# Valsartan attenuates LPS-induced ALI by modulating NF-κB and MAPK pathways

**DOI:** 10.3389/fphar.2024.1321095

**Published:** 2024-01-15

**Authors:** Mi Zhou, Ling Meng, Qinke He, Chunguang Ren, Changyi Li

**Affiliations:** ^1^ Department of Respiratory and Critical Care, Second Affiliated Hospital of Chongqing Medical University, Chongqing, China; ^2^ Laboratory of Developmental Biology, Department of Cell Biology and Genetics, School of Basic Medical Sciences, Chongqing Medical University, Chongqing, China

**Keywords:** valsartan, acute lung injury, LPS, MAPK, NF-κB

## Abstract

**Background:** Acute lung injury (ALI)/acute respiratory distress syndrome (ARDS) is a common respiratory disease characterized by persistent hypoxemia and an uncontrolled inflammatory response. Valsartan, an angiotensin II type 1 receptor antagonist, is clinically used to treat hypertension and has anti-inflammatory and antioxidant effects on gefitinib-induced pneumonia in rats. However, the potential therapeutic effects of valsartan on lipopolysaccharide (LPS)-induced ALI remain unclear. This study investigated the protective role of valsartan in LPS-induced ALI and its underlying mechanisms.

**Methods:** LPS-treated BEAS-2B cells and ALI mouse model were established. BEAS-2B cells were treated with LPS (10 μg/mL) for 24h, with or without valsartan (20, 40, and 80 µM). For ALI mouse models, LPS (5 mg/kg) was administered through intratracheal injection to treat the mice for 24h, and valsartan (10 or 30 mg/kg) was injected intraperitoneally twice 2 h before and 12 h after the LPS injection. Pulmonary functional parameters were examined by an EMKA pulmonary system. Hematoxylin and eosin staining, flow cytometry, CCK-8 assay, qRT-PCR, ELISA, immunofluorescence, Western blotting, and related commercial kits were used to assess the pathological damage to the lungs, neutrophil recruitment in the lung tissue and bronchoalveolar lavage fluid (BALF), cell viability, inflammation, oxidative activity, and mucus production, respectively. Potential mechanisms were further explored using network pharmacology and Western blotting.

**Results:** Valsartan rescued LPS-reduced cell viability of BEAS-2B cells, improved the pulmonary function, ameliorated pathological lung injury in mice with ALI, ameliorated LPS-induced neutrophil recruitment in BALF and lung tissue of mice, attenuated oxidative stress by increasing the level of SOD and decreasing that of MDA and GSSG, inhibited LPS-induced MUC5AC overproduction, decreased the LPS-induced increase in expression of pro-inflammatory cytokines/chemokines including TNF-α, IL-6, IL-1β, CXCL-1 and CXCL-2, and restored the expression of anti-inflammatory IL-10. Mechanistic studies showed that valsartan inhibits LPS-induced phosphorylation of nuclear factor-kappa B (NF-κΒ) and mitogen-activated protein kinases (MAPKs) including P38, extracellular signal-regulated kinase (ERK), and c-Jun N-terminal kinase (JNK) in both LPS-treated cells and the mouse model of ALI.

**Conclusion:** Valsartan protects against LPS-induced ALI by attenuating oxidative stress, reducing MUC5AC production, and attenuating the inflammatory response that may involve MAPK and NF-κΒ pathways.

## 1 Introduction

Acute lung injury (ALI)/acute respiratory distress syndrome (ARDS) is an acute inflammatory disease with a 30%–40% mortality rate and limited treatment options (Croasdell Lucchini et al., 2021; Wang et al., 2022; Li et al., 2023; Li et al., 2023). ALI is caused by various pathogenic factors, the most common of which are bacterial infections, particularly Gram-negative bacterial endotoxins such as lipopolysaccharide (LPS) (Li et al., 2023). LPS activates innate immune responses mainly through activating toll-like receptor 4 (TLR4), which in turn activates the downstream nuclear factor-kappa B (NF-κΒ) and mitogen-activated protein kinase (MAPK) signaling pathways (Wang et al., 2022; Li et al., 2023). NF-κΒ and MAPK signaling further induce the expression of inflammatory genes, oxidative stress, and mucus overproduction, thus promoting inflammation and pulmonary permeability, eventually leading to ALI (Gouda et al., 2018; Croasdell Lucchini et al., 2021; Li et al., 2023; Cortés-Ríos and Rodriguez-Fernandez, 2023). The airway epithelial barrier is the first line of defense against external damage, and its disruption causes leukocyte recruitment and pulmonary edema, thereby promoting ALI progression (Croasdell Lucchini et al., 2021). Thus, targeting bronchial epithelial cells has become an important therapeutic strategy for ALI (Gouda et al., 2018; Croasdell Lucchini et al., 2021), with LPS-treated human bronchial BEAS-2B epithelial cells and mice being widely used as models of ALI.

Valsartan is an angiotensin II type 1 receptor antagonist commonly used to treat hypertension (Cortés-Ríos and Rodriguez-Fernandez, 2023). Previous studies have shown that telmisartan, a novel angiotensin II type 1 receptor blocker, reduces mucin MUC5AC secretion in LPS-treated BEAS-2B cells (Chen et al., 2021) and alleviates LPS-induced pneumonia in rats by anti-inflammatory and anti-oxidative stress activity through peroxisome proliferator-activated receptor gamma (PPAR-γ)/NF-κΒ pathways (Zhou et al., 2022). These studies demonstrated that telmisartan improves LPS-induced ALI. Notably, telmisartan can also directly function as a PPAR-γ agonist, and PPAR-γ receptor activation could produce anti-inflammatory and anti-oxidative stress (Lan et al., 2023; Zhong et al., 2023). Thus, it remains unclear whether the protective effect of telmisartan against ALI is solely dependent on its angiotensin II type 1 receptor-blocking activity. Valsartan is more specific in inhibiting angiotensin II receptor type 1 than telmisartan and exhibits different pharmacological effects, For example, telmisartan promotes insulin secretion in rat islets, whereas valsartan does not (Liu et al., 2021), and has a better therapeutic effect in hypertension than valsartan (Takagi et al., 2013). Therefore, the effects of valsartan on ALI warrant further investigation. Here, we aimed to investigate whether valsartan protects against LPS-induced ALI through anti-inflammatory and anti-oxidative stress activity and inhibition of mucin MUC5AC secretion, as well as to explore the underlying mechanism using network pharmacology.

## 2 Materials and methods

### 2.1 Drugs and reagents

All following drugs and reagents were acquired commercially: Valsartan (Sigma-Aldrich, St. Louis, MO, United States); Lipopolysaccharide (Sigma-Aldrich, St. Louis, MO, United States); Cell Counting Kit-8 (CCK-8) (Biosharp, Guangzhou, China); Malondialdehyde (MDA) Content Assay Kit (Solarbio, Beijing, China); Oxidized Glutathione (GSSG) Content Assay Kit (Solarbio, Beijing, China); Superoxide Dismutase (SOD) Activity Assay Kit (Nanjing Jiancheng Bioengineering Institute, Nanjing, China); Antibodies against p-AKT, AKT, P38, p-JNK, JNK, ERK and MUC5AC (Zen-bio, Chengdu, China). Antibodies against p-P38, p-ERK, and Tubulin (Affinity Biosciences, Jiangsu, China). Antibodies against GAPDH and β-actin (ABclonal, Wuhan, China).

### 2.2 Cell culture and treatment

The human bronchial BEAS-2B epithelial cells were cultured in RPMI-1640 containing 1% penicillin/streptomycin and 10% fetal bovine serum (FBS) at 37°C and 5% CO2 in humidified air. The BEAS-2B cells were cultured to reach a confluence of 60%–70% before treatment with LPS/valsartan. LPS (10 μg/mL) and/or valsartan (20, 40, and 80 µM) were used for treating BEAS-2B cells for 24 h in this study.

### 2.3 Cell Counting Kit (CCK)-8 assay

BEAS-2B cells (5 × 10^3^/well) were seeded in the 96-well plates and cultured for 12 h before 24 h-starvation followed by treatment with different concentrations of valsartan (0, 20, 40, and 80 µM) or LPS (0, 10, 30, 50, and 100 μg/mL) for 24 h. Cells were then washed twice with sterile PBS and incubated with CCK-8 working solution (10 µL/well) at 37°C for 1.5 h. The absorption of each well was measured at 450 nm with a plate reader (Tecan).

### 2.4 Animals and experimental design

C57/6N mice (20–28 g) were purchased from Charles River (Beijing, China). Mice were kept under standard conditions (22°C ± 2°C; 12 h light/dark cycle) and were free to drink and eat. All animal studies were approved by the Institutional Animal Care and Use Committees of Chongqing Medical University. Mice were divided into four groups: control group (saline), LPS (5 mg/kg) group, LPS (5 mg/kg) + valsartan low-dose group (10 mg/kg), LPS (5 mg/kg) + valsartan high-dose group (30 mg/kg), n = 6 in each group. Mice were anesthetized and intratracheally instilled with saline or LPS. Valsartan (Sigma, United States) was intraperitoneally injected twice by 2 h prior to as well as 12 h after LPS administration. Mice were euthanized 24 h after LPS instillation. Bronchoalveolar lavage fluid (BALF) and lung tissue were collected and subjected to further measurements. The dosages for LPS and valsartan were chosen according to the previous studies (Alhazzani et al., 2021; Liang et al., 2022).

### 2.5 Whole body plethysmography for assessment of lung function

The method of whole body plethysmography (WBP) along with FinePoint software (Emka, France) is used to measure lung function indicators in awake, unrestrained mice. These indicators include respiratory rate (F), tidal volume (TV), ventilation per minute (MV), peak expiratory flow rate (PEF), and peak inspiratory flow rate (PIF).

### 2.6 Histological evaluation

Lung tissue of mice was fixed in 10% neutral buffered formalin for 48 h. Subsequently, lung tissues were dehydrated with gradient concentrations of alcohol. And tissues were subjected to paraffin embedding and sectioned to 4 µm. Tissue sections were then stained with hematoxylin and eosin (H&E) to detect pathological changes. The lung injury was quantitatively analyzed according to an official protocol from the American Thoracic Society (Matute-Bello et al., 2011), and 20 random high-power fields (×400 total magnification) in each group and scored according to Table 1.

**TABLE 1:** Lung injury score system.

Parameter	Score per field		
	0	1	2
A. Neutrophils in the alveolar space	none	1–5	5
B. Neutrophils in the interstitial space	none	1–5	5
C. Hyaline membranes	none	1	1
D. Proteinaceous debris filling the airspaces	none	1	1
E. Alveolar septal thickening	2x	2x–4x	4X

Score = [(20 × A) + (14 × B) + (7 × C) +(7 × D) + (2 × E)]/(number of fields × 100).

### 2.7 Collection of BALF

Mice were executed by intraperitoneal injection with a lethal dose of 2% sodium pentobarbital, the neck and trachea were completely exposed, and 0.5 mL of PBS was intratracheally injected into the lungs followed by BALF collection, and the lavage was repeated three times. BALF was then centrifuged at 1000 g at 4°C for 10 min, the supernatant was harvested for measuring the total protein in the BALF according to the instructions of the bicinchoninic acid (BCA) kit (CWBIO, China). The cell precipitates were resuspended in PBS, then numbered with a cell counter (RWD, Shenzhen, China), and used for further cellular analysis.

### 2.8 Wright-giemsa Stain

The BALF was collected, smeared, dried naturally, and fixed with methanol. The slide was then stained with Wright-Giemsa Stain (Solarbio, Beijing, China) for 3 min after fixing. Following the staining, the slide was observed under a microscope to manually count the neutrophils with purple segmented nucleus in the BALF.

### 2.9 Quantification of MDA, SOD, and GSSG activities

The mice lung tissues or BEAS-2B cells were collected and assayed for MDA, SOD, and GSSG according to the instructions of the relevant commercial kits (Solarbio, Beijing, China).

### 2.10 Quantitative real-time polymerase chain reaction (qRT-PCR)

Total RNA was extracted from the treated mice lung tissues or BEAS-2B cells with TRIzol reagent (ABclonal, Wuhan, China). Total RNA was reverse transcribed into cDNA with ABScript III RT Master Mix for qPCR with gDNA Remover (ABclonal, Wuhan, China), and cDNA was used as the template for the PCR reaction. Real-time PCR amplification was performed using 2X universal SYBR Green Fast qPCR Mix (ABclonal, Wuhan, China) with a Bio-Rad CFX96 system according to the manufacturer’s protocol. Relative expression was quantified by the 2^−ΔΔCt^ method. The GAPDH was used as an internal control. The primer sequences of genes measured in the RT-PCR assay are listed in Supplementary Table S1.

### 2.11 Flow cytometry analysis

Lung tissues were minced and digested in 1,640 medium containing 3% FBS and type I collagenase (1 mg/mL, Sigma-Aldrich, St. Louis, MO, United States of America) and deoxyribonuclease I (200 μg/mL, Sigma-Aldrich, St. Louis, MO, United States of America) for 30–60 min at 37°C, and the reaction was terminated by the addition of PBS containing EDTA (20 mM). The digested tissues were sieved through a 70 μm nylon filter to get single-cell suspensions. The resuspended BALF cells and lung tissue single-cell suspensions were numbered with a cell counter (RWD, Shenzhen, China). The cells were diluted to 1–2 × 10^6^/100 μL, incubated with a non-specific binding blocker of anti-mouse CD16/CD32 antibody (2.4G2), and the cell surface antigens were stained with the following, fluorescent-labeled antibodies obtained from BD Bioscience (BD), BioLegend (BL). Flow-through fluorescent antibodies used were: FITC-anti-mouse CD45 (30-F11, BL, 103108, 0.5 mg/mL); PE-anti-mouse Ly6G (1A8, BD, 551461, 0.2 mg/mL); APC-anti-mouse CD11b (A1/70, BL, 101212, 0.2 mg/mL), stained in 100ul cell suspension on ice with light protection, and then terminated with FBS-containing PBS as a staining buffer, followed by centrifugation at 500 g for 5min, and resuspension. The stained cells were analyzed on CytoFLEX (BECKMAN COULTER) and the data were analyzed using FlowJo software.

### 2.12 Immunofluorescence (IF)

Paraffin sections were prepared and stained as previously described (Zeng et al., 2023). Briefly, sections were treated with 0.1% Triton X-100, then washed three times with TBST, and blocked with 5% BSA for 30 min. Subsequently, the sections were incubated with an anti-myeloperoxidase (Mpo) antibody (1:200, ABclonal, A24531, China) at 4°C overnight. Following three washes with TBST, the samples were incubated with anti-HRP Goat Anti-Rabbit IgG antibody (1:100, ABclonal, AS014, China) for 1 h at room temperature in the dark. Then the sections were prepared with a mounting medium containing DAPI after TBST washes. The images were then captured under a Leica DM6B fluorescence microscope and analyzed using ImageJ software.

### 2.13 Western blotting

For Western blot analysis of proteins extracted from mice lung tissues and BEAS-2B cells, samples were lysed with radioimmunoprecipitation assay buffer, and protein levels were quantified using a BCA protein assay kit. Protein denaturation was performed at 100°C for 10min. Each well was loaded with approximately 30 μg of total protein and separated on sodium dodecyl sulfate -denatured polyacrylamide gels with specific concentrations (12% gel for Cyclophilin B, 7% gel for MUC5AC, and 10% gel for the remaining proteins). The proteins were then transferred to polyvinylidene fluoride membranes. And membranes were blocked with 5% skim milk (or 5% BSA for detecting phosphorylated proteins) for 2 h at room temperature. The primary antibodies used for Western blotting were anti-MUC5AC (1:1000), anti-P38 (1:1000), anti-p-P38 (1:1000), anti-JNK (1:1000), anti-p-JNK (1:1000), anti-ERK (1:1000), anti-p-ERK (1:1000), anti-p-P65 (1:1000), P65 (1:1000), anti-GAPDH (1:5000), anti-Tubulin (1:3000), anti-Cyclophilin B (1:1000) and anti-β-actin (1:10000). The membranes were sequentially incubated with above mentioned primary antibodies and secondary antibodies. The blots were further detected using ECL chemiluminescence, and ImageJ software was used to quantify the bands.

### 2.14 Network pharmacology

The 2D structure of valsartan was obtained from PubChem (https://pubchem.ncbi.nlm.nih.gov/) by entering the keyword “valsartan”. Based on the 2D structure of valsartan, the drug targets of valsartan were obtained from the SwissTargetPrediction database (http://www.swisstargetprediction.ch/). The targets of Acute Lung Injury were also obtained by entering the keyword “Acute Lung Injury” in the GeneCards database (https://www.genecards.org/). The overlapping targets of valsartan-ALI were identified by using bioinformatics (https://www.bioinformatics.com.cn/) and the corresponding Venn diagram was obtained. The common targets were then imported into the STRING database, in which the protein category was set as *H. sapiens*, and the minimum required interaction score was “medium confidence (0.4)” and “hide disconnected nodes in the network” to obtain the protein-protein interaction (PPI) network. The GO and KEGG enrichment analysis diagrams were obtained with common targets by using the David database (https://david.ncifcrf.gov/).

### 2.15 Statistical analysis

GraphPad Prism software was used for data analysis. All data were displayed as mean ± standard deviation (SD), and differences between groups were analyzed by one-way ANOVA analysis with *post hoc* Dunnett’s or Tukey’s test that were indicated in the figure legends. The *p*-value of <0.05 was considered to be statistically significant.

## 3 Results

### 3.1 Valsartan attenuates LPS-induced epithelial cell cytotoxicity

**FIGURE 1:**
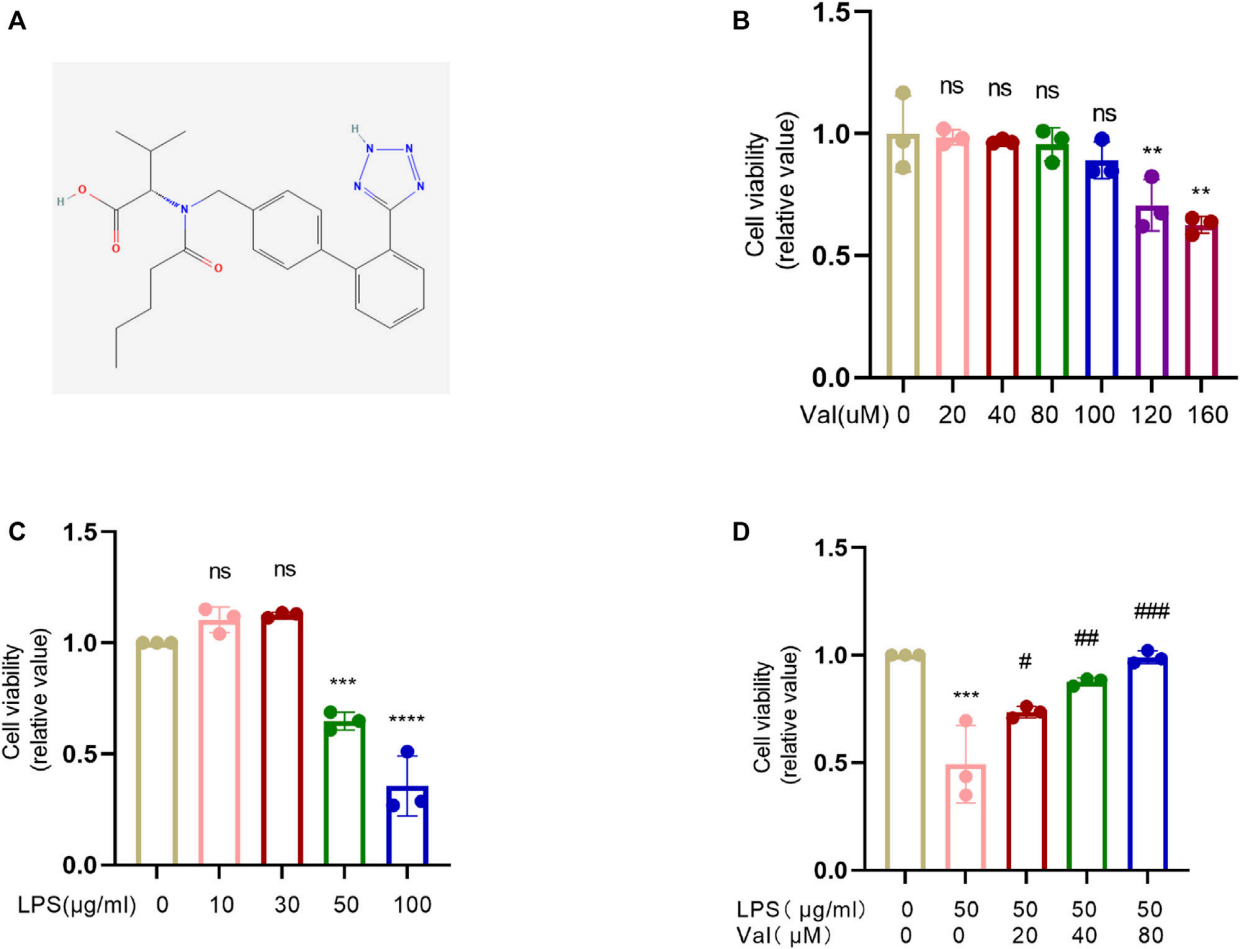
Valsartan protects cell viability against LPS treatment in BEAS-2B cells. **(A)** The 2D chemical structure of valsartan. **(B–D)** The BEAS-2B cells were incubated with valsartan and/or LPS at indicated concentrations for 24h, and the cell viability was measured with CCK-8 assay. Each data point represents a biological replicate **(B–D)**, and the experiments in **(B–D)** were repeated three times. One-Way ANOVA (Dunnett’s test for **(B,C)**, and Tukey’s test for **(D)**. ***p < 0.001, ****p < 0.0001, *versus* control group; ^#^ *p* < 0.05, ^##^ *p* < 0.01, ^###^ *p* < 0.001, *versus* LPS group. ns, not statistically significant. Val, valsartan; LPS, lipopolysaccharide.

The 2D structure of valsartan was obtained from PubChem (Figure 1A). The effect of valsartan on the viability of bronchial BEAS-2B cells was measured using the CCK8 assay. The results showed that treatment with 0–80 µM valsartan did not have any impact on the viability of BEAS-2B cells. However, there was a dosage-dependent reduction in cell viability upon treatment with valsartan in dosages ≥100 µM (Figure 1B). Therefore, we selected 80 µM as the maximum dosage to assess its protective effects against LPS-induced cell death. We stimulated BEAS-2B cells with LPS to assess the effect of different concentrations of LPS on cell viability. We observed that only 50 or 100 μg/mL LPS significantly reduced cell viability (Figure 1C). To examine whether valsartan protects from LPS-mediated BEAS-2B cell damage, we challenged the BEAS-2B cells with 50 μg/mL LPS with or without 20, 40, or 80 µM valsartan for 24 h. The results showed that valsartan restored LPS-induced cell viability in a dose-dependent manner (Figure 1D). Therefore, we chose valsartan concentrations of 20,40, and 80 µM for the follow-up research.

### 3.2 Valsartan ameliorates LPS-induced acute lung injury in mice

**FIGURE 2:**
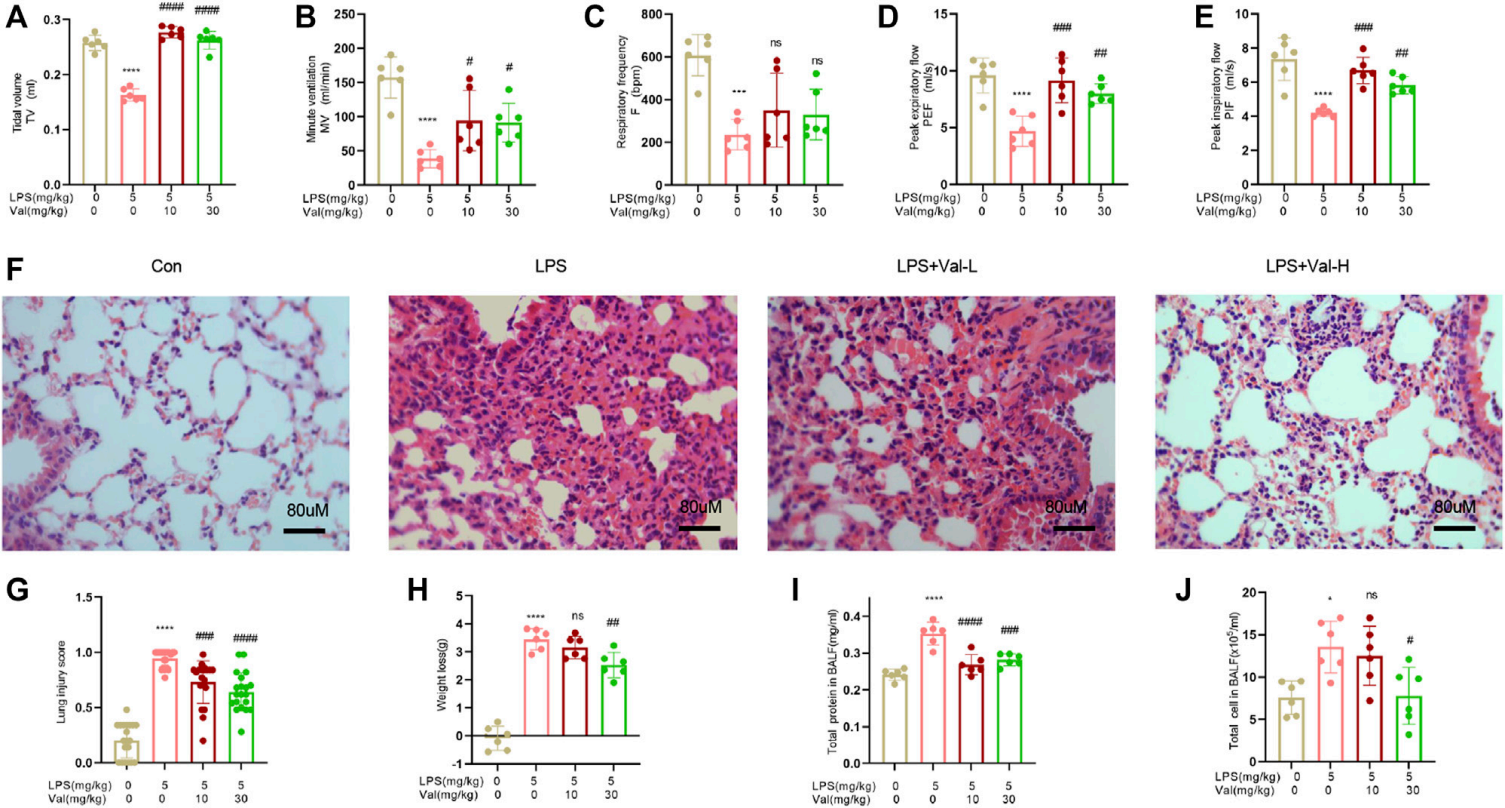
Valsartan ameliorates LPS-induced acute lung injury in mice. **(A–E)** The whole body plethysmography (WBP) method was used to detect the indicators of lung function, including tidal volume (TV), ventilation per minute (MV), respiratory rate (F), peak expiratory flow rate (PEF), and peak inspiratory flow rate (PIF) in LPS-induced ALI mice. **(F)** Representative images of HE staining of lung tissue of PBS or LPS-treated mice with or without valsartan treatment. Magnification, ×400. Scale bar = 80 µm. **(G)** Quantitative analysis of lung injury in F. **(H–J)** Measurements of weight loss, total protein and cell counts in BALF of mice treated as in A (n = 6). Each data point represents an individual mouse **(A–J)**, and the experiments in **(A–J)** were repeated three times. One-Way ANOVA (Tukey’s test) for **(A-E) and (G-J)**. *p < 0.05, ****p < 0.0001, *versus* control group; ^#^ *p* < 0.05, ^##^ *p* < 0.01, ^###^ *p* < 0.001, ^####^ *p* < 0.0001, *versus* LPS group. LPS + Val-L, LPS (5 mg/kg) + valsartan at low-dose (10 mg/kg); LPS + Val-H, LPS (5 mg/kg) + valsartan at high-dose (30 mg/kg); BALF, bronchoalveolar lavage fluid.

To evaluate the protective effects of valsartan against LPS-induced ALI in mice, we first utilized an EMKA pulmonary system to measure various pulmonary functional parameters, such as respiratory rate (F), tidal volume (TV), ventilation per minute (MV), peak expiratory flow rate (PEF), and peak inspiratory flow rate (PIF). Our findings revealed that the LPS group exhibited a decrease in F, TV, MV, PEF, and PIF (Figures 2A–E). However, treatment with valsartan significantly resotored these parameters (Figures 2A–E). Therefore, the results suggested that valsartan alleviated LPS-induced pulmonary dysfunction in mice. We then performed H&E staining to evaluate pathological changes in lung tissues. Compared to the control group, LPS stimulated robust lung tissue damage, accompanied by neutrophil infiltration, alveolar wall thickening, and pulmonary edema. Notably, the LPS-induced lung tissue damage was significantly reduced by valsartan treatment in a dose-dependent manner (Figures 2F, G). Similarly, valsartan treatment significantly improved LPS-induced weight loss (Figure 2H) and attenuated the increase in total protein and cell counts in BALF of LPS-treated mice (Figures 2I, J). These results demonstrate the protective role of valsartan in LPS-induced ALI.

### 3.3 Valsartan ameliorates LPS-induced neutrophil recruitment in BALF and lung tissue of mice

**FIGURE 3:**
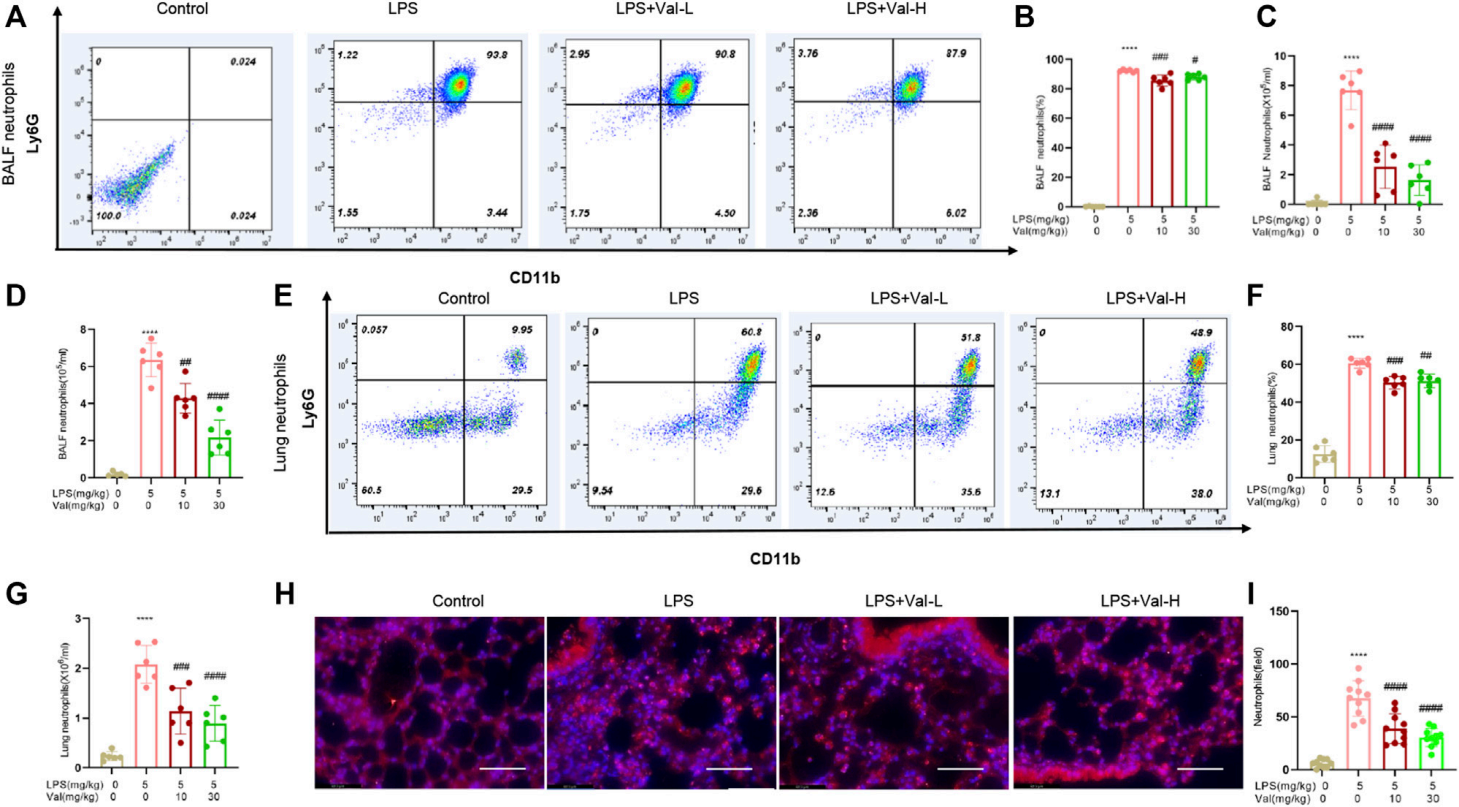
Valsartan ameliorates LPS-induced neutrophil recruitment in BALF and lung tissue of mice. **(A–C and E–G)** Flow cytometry detection of neutrophils in BALF **(A–C)** and lung tissues of ALI mice **(E–G)**. **(D)** Quantification of Giemsa-stained neutrophil in BALF. **(H,I)** Representative images **(H)** and quantification **(H)** of immunofluorescence staining with anti-MPO antibody in lung tissue. Magnification, ×400. Scale bar = 125 µm. Each data point represents an individual mouse **(A–G)** and an individual observation field **(I)**. The experiments in **(A–I)** were repeated three times. One-Way ANOVA (Tukey’s test) for **(B–D, F, G and I)** ****p < 0.0001, *versus* control group; ^#^ *p* < 0.05, ^##^ *p* < 0.01, ^###^ *p* < 0.001, ^####^ *p* < 0.0001 *versus* LPS group.

The effect of valsartan on LPS-induced neutrophil recruitment in the lung of ALI mouse model was determined using flow cytometry, Wright-Giemsa stain and immunofluorescence staining of mpo. The results showed that valsartan significantly ameliorated LPS-induced neutrophil recruitment in both BALF and lung tissue (Figures 3A–I).

### 3.4 Valsartan ameliorates LPS-induced inflammatory cytokine expression in human BEAS-2B epithelial cells and murine lung tissue

**FIGURE 4:**
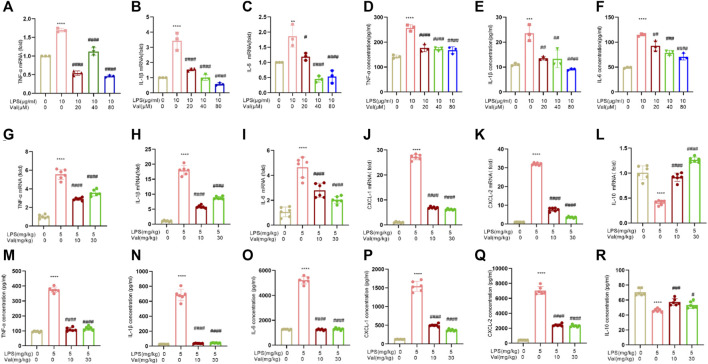
Valsartan ameliorates LPS-induced inflammatory cytokine expression in human BEAS-2B epithelial cells and mouse lung tissue. **(A–R)** qRT-PCR and ELISA analysis of inflammatory cytokine in LPS-treated BEAS-2B cells **(A–F)** and ALI mouse lung tissue **(G–R)**. The experiments in **(A–R)** were repeated three times. Each data point represents a biological replicate in **(A–F)** and an individual mouse in **(G–R)** (n = 6). One-Way ANOVA (Tukey’s test) for **(A-R)**. **p < 0.01, ****p < 0.0001, *versus* control group; ^#^ *p* < 0.05, ^####^ *p* < 0.0001 *versus* LPS group.

We examined the effect of valsartan on the mRNA and protein expression of inflammatory factors in LPS-treated BEAS-2B cells and lung tissue of mice. The results showed that the mRNA and protein expression levels of inflammatory factors TNF-α, IL-6, and IL-1β were increased in BEAS-2B cells treated with LPS. However, treatment with valsartan at concentrations ranging from 20 to 80 µM improved this LPS-induced overexpression (Figures 4A–F). In addition, in lung tissues of ALI mice that were treated with LPS, the mRNA and protein levels of inflammatory factors TNF-α, IL-6, IL-1β, CXCL-1, and CXCL-2 were found to be elevated, while the expression level of anti-inflammatory factor IL-10 was reduced. However, when treated with valsartan, the release of LPS-induced inflammatory factors was reduced while the release of anti-inflammatory factors was increased (Figures 4G–R). These data demonstrate that valsartan attenuates LPS-induced inflammatory responses in both human bronchial BEAS-2B epithelial cells and lung tissue of mice.

### 3.5 Valsartan attenuates oxidative stress in LPS-treated BEAS-2B cells and lung tissue of mice

**FIGURE 5:**
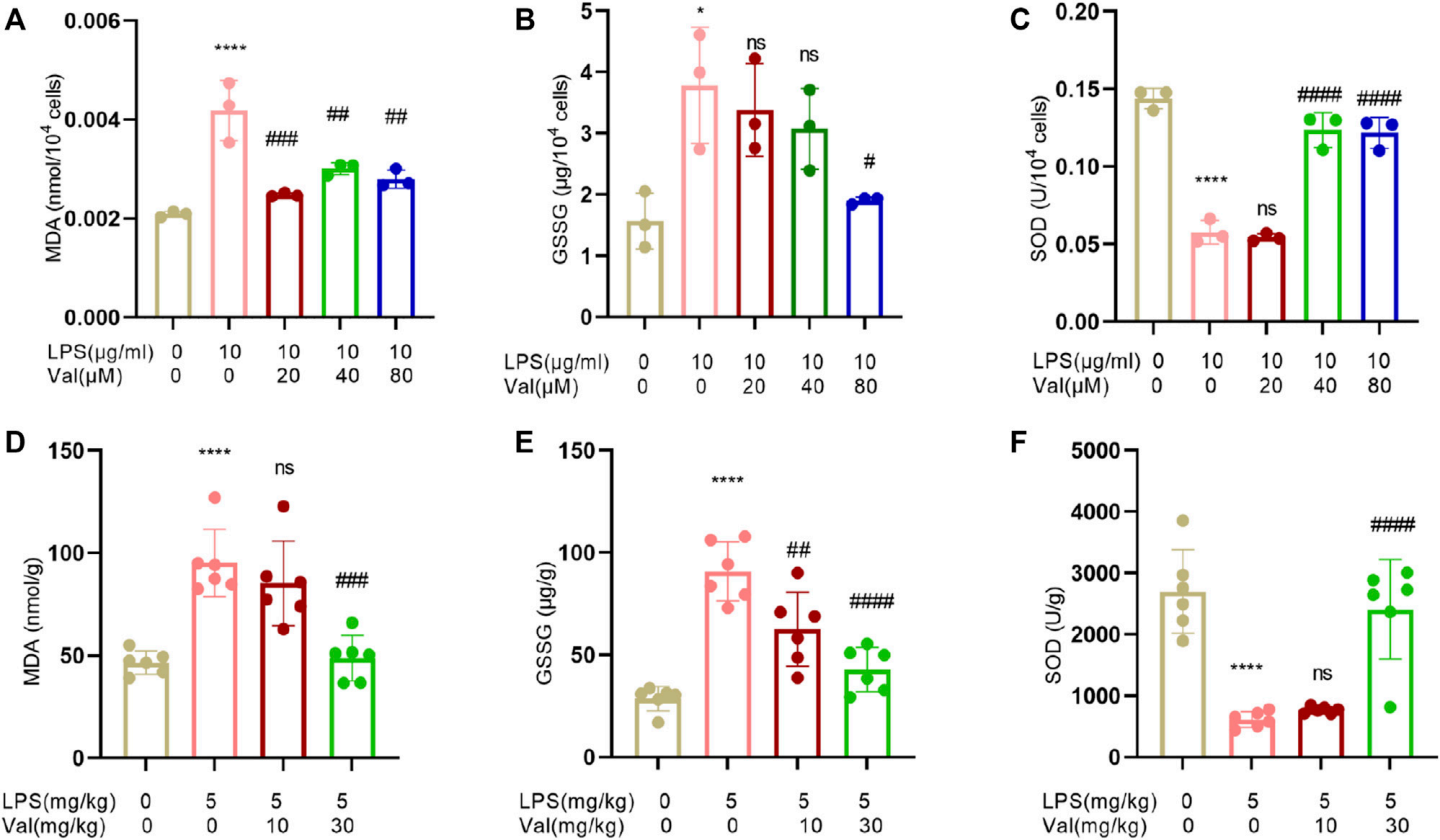
Valsartan attenuates oxidative stress in LPS-treated BEAS-2B cells and lung tissue in mice. **(A–F)** Quantitative analysis of MDA, GSSG and SOD in LPS-treated BEAS-2B cells **(A**–**C)** and ALI mouse lung tissue **(D–F)**. The experiments in **(A–F)** were repeated four times. Each data point represents a biological replicate in **(A-C)** and an individual mouse in **(D–F)** (n = 6). One-Way ANOVA (Tukey’s test) for **(A–F)**. *p < 0.05, ****p < 0.0001, *versus* control group; ^#^ *p* < 0.05, ^##^ *p* < 0.01, ^###^ *p* < 0.001, ^####^ *p* < 0.0001, *versus* LPS group.

Oxidative stress is usually activated in LPS-induced ALI and contributes to ALI progression (Bezerra et al., 2023). The effects of valsartan on LPS-induced oxidative stress in ALI were assessed by measuring MDA, GSSG, and SOD levels. The results showed that valsartan treatment reversed the LPS-induced increase in MDA and GSSG levels, as well as the reduction in SOD levels in both BEAS-2B cells (Figures 5A–C) and mouse lung tissue (Figures 5D–F). These results indicate that valsartan attenuates oxidative stress in LPS models of ALI.

### 3.6 Valsartan inhibits MUC5AC production in LPS-treated BEAS-2B cells and lung tissue of mice

**FIGURE 6:**
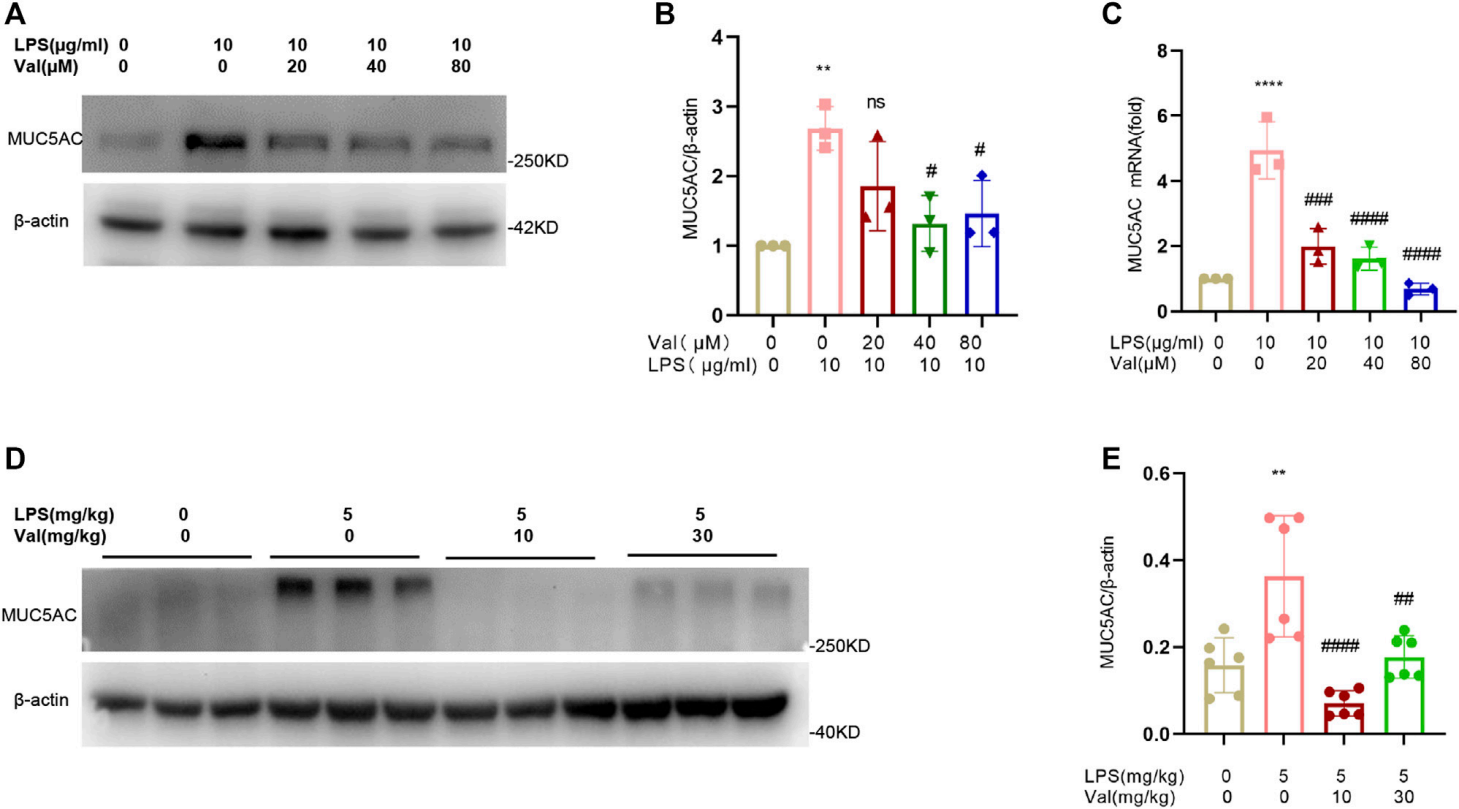
Valsartan inhibits MUC5AC production in LPS-treated BEAS-2B cells and lung tissue in mice. **(A–C)** Representative blots **(A)** and quantification of MUC5AC proteins **(B)** and mRNAs **(C)** in LPS-treated BEAS-2B cells. **(D,E)** Representative blots **(D)** and quantification of MUC5AC proteins **(E)** in the lung tissue of LPS-induced ALI mouse model. The experiments in **(A–E)** were repeated three times. Each data point represents a biological replicate in **(A-C)** and an individual mouse in **(D–E)** (n = 6). One-Way ANOVA (Tukey’s test) for **(B, C, E)** **p < 0.01, ****p < 0.0001, *versus* control group; ^#^ *p* < 0.05, ^##^ *p* < 0.01, ^###^ *p* < 0.001, ^####^ *p* < 0.0001, *versus* LPS group.

Excessive secretion of respiratory mucus is an important characteristic of ALI, and an increase in Mucin 5AC (MUC5AC), the main component of respiratory secretions, can further exacerbate lung injury (Chen et al., 2021). We measured the mRNA and protein levels of MUC5AC in BEAS-2B epithelial cells after treatment with LPS and valsartan. Valsartan significantly attenuated LPS-induced MUC5AC expression at both transcriptional and translational levels (Figures 6A–C). Similarly, valsartan suppressed the expression of MUC5AC protein in the lung tissue of the ALI mouse model (Figures 6D, E). These data suggest that valsartan inhibits LPS-induced mucus overproduction.

### 3.7 Valsartan modulates NF-κB and MAPK pathways in LPS-treated BEAS-2B cells and mice

**FIGURE 7:**
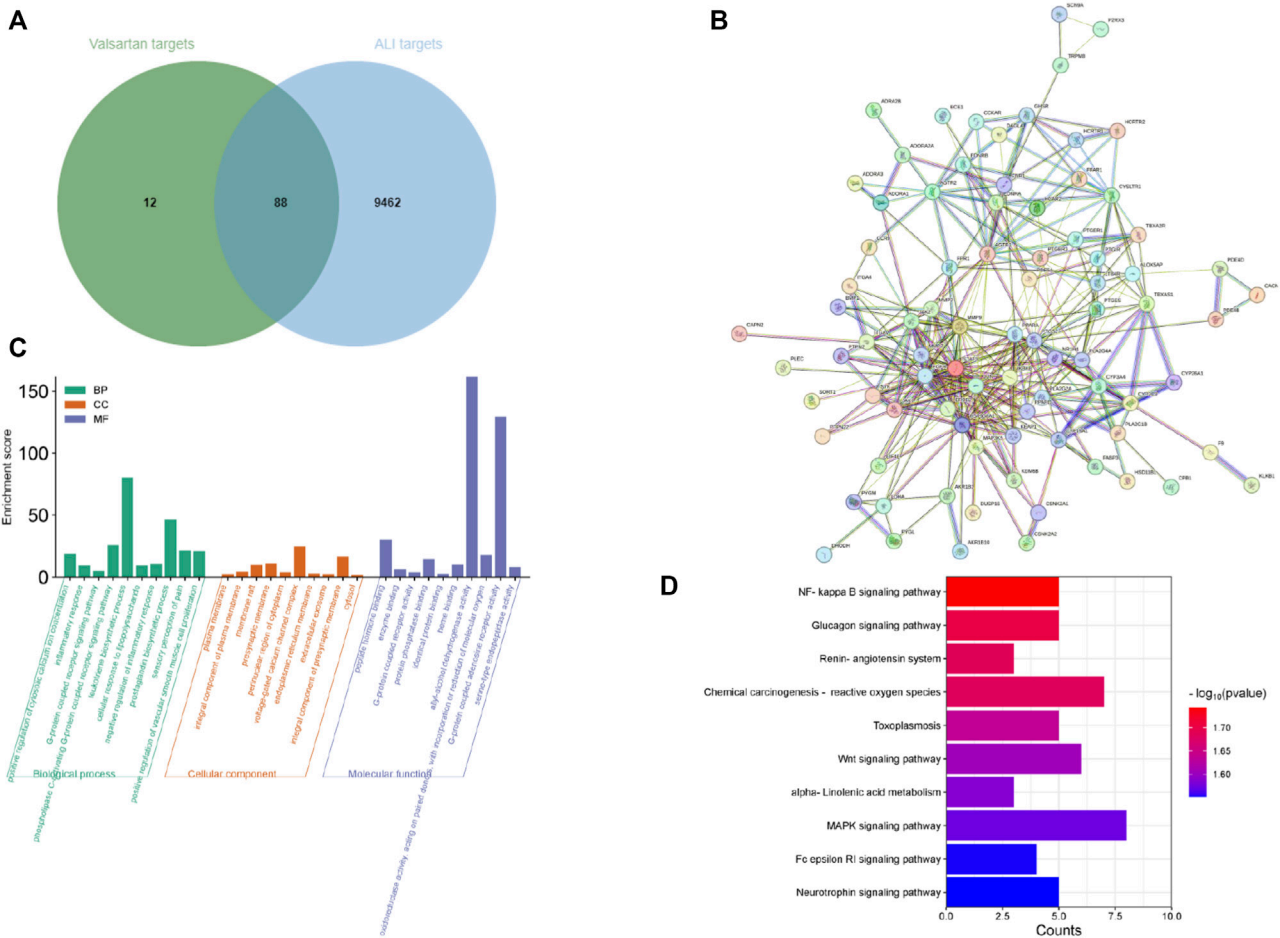
Network pharmacological analysis of valsartan in ALI. **(A)** The Venn diagram of valsartan and ALI targets. **(B)** PPI network of the 88 overlapping targets between valsartan and ALI targets. **(C,D)** GO function enrichment analysis **(C)** and KEGG pathway enrichment analysis **(D)** of the 88 overlapping targets between valsartan and ALI targets.

After demonstrating that valsartan protects against LPS-induced ALI in human lung epithelial cells and in a mouse model, we investigated the underlying molecular mechanisms of action of valsartan in ALI. We used network pharmacology to predict valsartan targets in ALI. The 2D structure of valsartan from PubChem (Figure 1A) was entered into the SwissTargetPrediction database, from which 100 potential targets of valsartan were identified (Supplementary Data Sheet S1). A total of 9,550 potential ALI targets were predicted using the GeneCards database (Supplementary Data Sheet S2). We put the obtained drug-disease targets into the Bioinformatics website and constructed a Venn diagram visualization (Figure 7A), and 88 ALI-specific valsartan targets were identified (Supplementary Data Sheet S3) and imported into the STRING database to construct the PPI network. The constructed PPI network contained 87 nodes and 336 edges with an average node degree of 7.72 and a PPI enrichment *p*-value of <1.0^e−16^ (Figure 7B). GO functional enrichment and KEGG pathway analyses were performed on these 88 valsartan targets, with a statistical significance of *p* < 0.05 (Supplementary Data Sheet S4–S7). GO function analysis revealed the enrichment of biological processes, including inflammatory response, G-protein coupled receptor signaling pathway, and leukotriene biosynthetic process, which were highly associated with LPS-TLR4 signaling and ALI (Figure 7C). Consistent with this, the molecular functions of protein phosphatase binding and oxidoreductase activity were mostly regulated by valsartan (Figure 7C). Thus, these results of GO function analysis suggest that valsartan plays an important role in the treatment of ALI through its anti-inflammatory and antioxidative stress effects.

**FIGURE 8:**
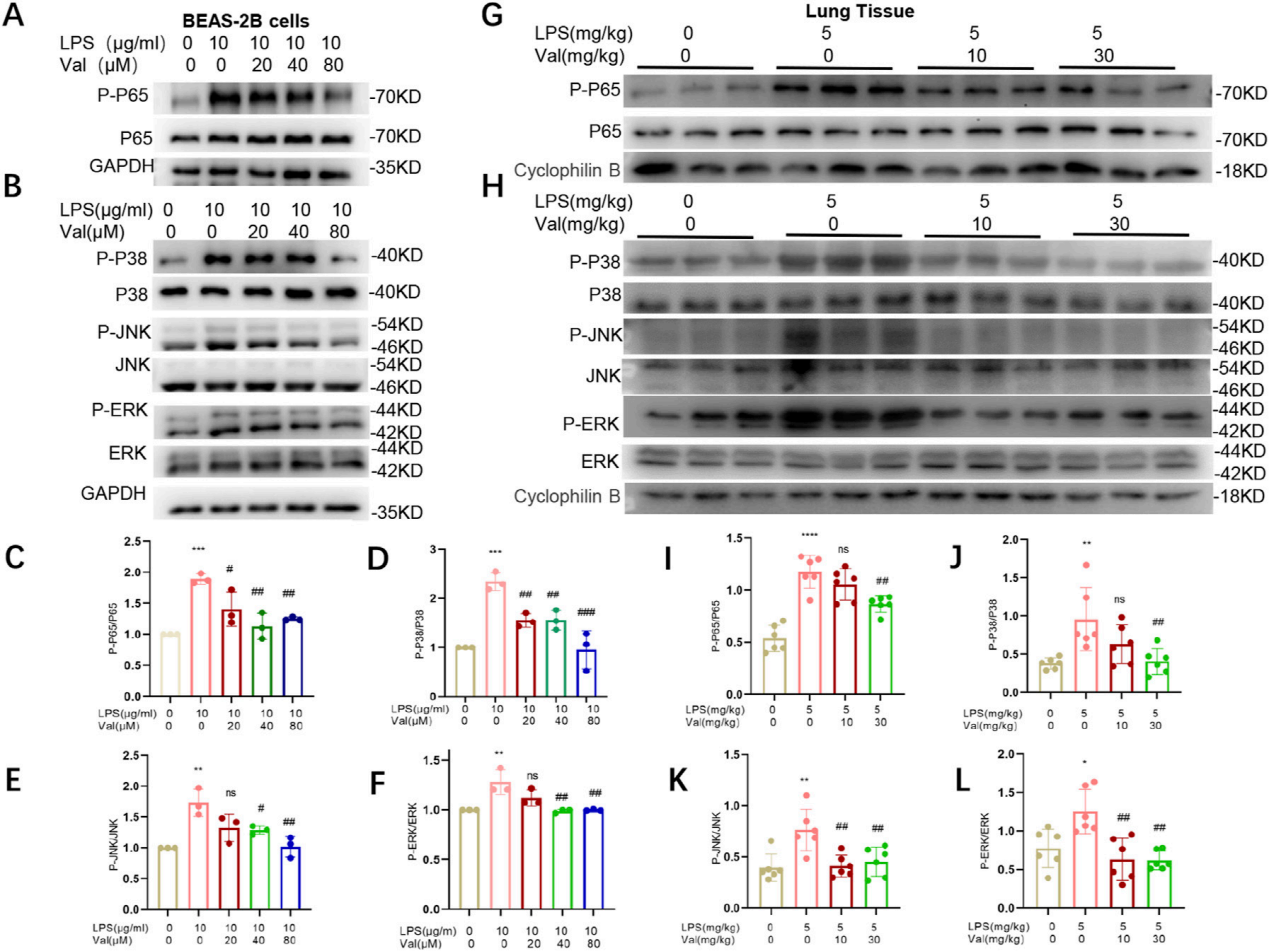
Valsartan modulates NF-κB and MAPK pathways in LPS-treated BEAS-2B cells and mice. **(A-F)** Representative blots **(A–B)** and quantification **(C-F)** of P-P65, P65 and P-P38, P38, P-JNK, JNK, P-ERK, and ERK in LPS-treated BEAS-2B cells. **(G-L)** Representative blots **(G–H)** and quantification **(I–L)** of P-P65, P65 and P-P38, P38, P-JNK, JNK, P-ERK, and ERK in the lung tissue of LPS-induced ALI mouse model. The experiments in **(A–L)** were repeated three times. Each data point represents a biological replicate in **(C–F)** and an individual mouse in **(I–L)** (n = 6). One-way ANOVA (Tukey’s test) for **(C–F)** and **(I–L)**. *p < 0.05, **p < 0.01, ***p < 0.0001, *versus* control group; ^#^ *p* < 0.05, ^##^ *p* < 0.01, ^###^ *p* < 0.001, *versus* LPS group.

NF-κB and MAPK signaling pathways are classical downstream pathways of LPS-TLR4 in mediating inflammation and lung injury (Liyanage et al., 2023; Yue et al., 2023). Notably, these two pathways were among the 74 enriched pathways in the KEGG analysis (*p* values of 0.018 and 0.027 for NF-κB and MAPK, respectively) (Figure 7D). We further investigated whether valsartan protects against LPS-induced ALI by modulating MAPK signaling and NF-κB pathways by performing *in vivo* and *in vitro* experiments. Valsartan attenuated the LPS-mediated increase in the phosphorylation of p65, P38, ERK, and JNK in BEAS-2B cells in a dose-dependent manner (Figures 8A–F). Further, valsartan inhibited the phosphorylation of p65 and MAPKs in the LPS-induced ALI mouse model (Figures 8G–L). These findings suggest that valsartan possibly ameliorates LPS-induced ALI by inhibiting MAPK and NF-κΒ phosphorylation.

## 4 Discussion

**FIGURE 9:**
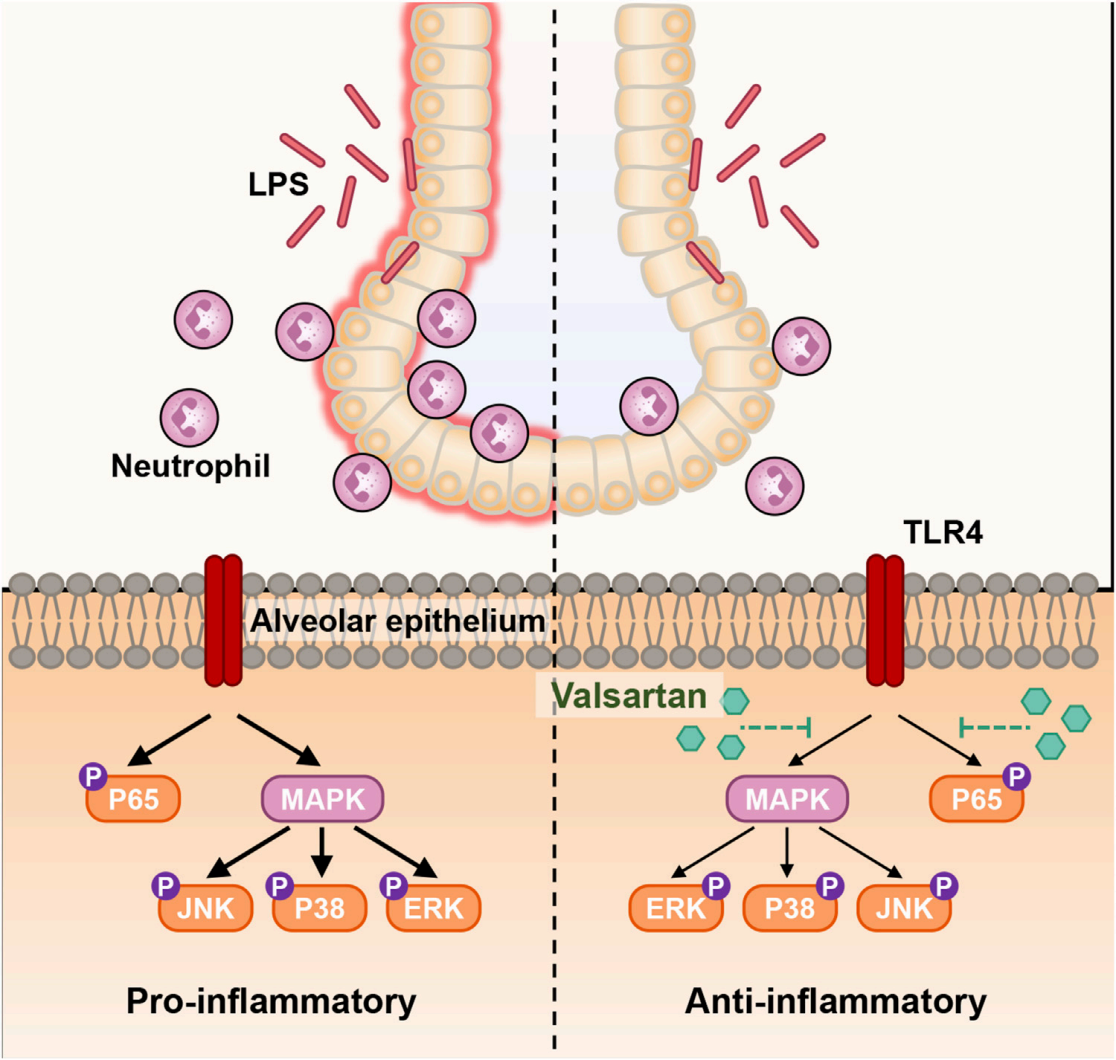
Schematic diagram of the molecular mechanism of valsartan attenuates LPS-induced ALI. In the LPS-induced BEAS-2B cell and mouse ALI models, LPS activates the natural immune response through various molecular mechanisms, including activation of TLR4, which in turn activates the downstream NF-κΒ and MAPK signaling pathways. Activation of NF-κΒ and MAPK signaling further induces recruitment of neutrophils into mouse and lung tissue and BALF the excessive release of inflammatory factors, oxidative stress, and mucus overproduction in mouse lung tissue and BEAS-2B cells, which leads to inflammation and accelerated infection, and ultimate ALI. Valsartan suppresses the LPS-TLR4 signaling by modulating the NF-κΒ and MAPK pathways.

Here, we investigated the protective role of valsartan in LPS-induced ALI and its underlying mechanisms using two classical models of ALI, including LPS-induced human epithelial BEAS-2B cells and mice. Our results demonstrated that valsartan protects against LPS-induced ALI. Valsartan was shown to ameliorate LPS-induced epithelial cell damage, reduce lung permeability, limit pulmonary recruitment of neutrophils, and decrease the expression of inflammatory cytokines, oxidative stress, and mucosal protein production. Furthermore, we observed that valsartan attenuated LPS-induced phosphorylation of p65 and MAPKs, such as P38, ERK, and JNK. These findings suggest that valsartan can downregulate NF-κΒ and MAPK pathways, thereby exhibiting anti-inflammatory and antioxidant activities that protect against ALI (Figure 9).

Alveolar epithelial cells, neutrophils, and macrophages are all important players in the pathogenesis of ALI/ARDS (Aggarwal et al., 2014; Williams and Chambers, 2014; Hiemstra et al., 2015; Hu et al., 2016). Experimental modulations involving anti-inflammatory, anti-oxidative stress and reduced secretion of MUC5AC can improve ALI pathogenesis (Zhang et al., 2016; Jangam et al., 2023). Our study found that valsartan treatment reduces the production and secretion of inflammatory cytokines (IL-6, TNF-a, IL-1β) and MUC5AC in both LPS-treated human BEAS-2B cells and mouse ALI models. Additionally, valsartan can reduce the production of chemokines (CXCL-1/2) and increase the expression of the anti-inflammatory factor, IL-10, in LPS-induced ALI mice. Furthermore, valsartan ameliorates LPS-induced oxidative stress by decreasing the levels of MDA and GSSG while increasing SOD levels. The release of chemokines in the lung during ALI drives neutrophil infiltration, which amplifies the severity of ALI by promoting pulmonary permeability and immune responses (Abraham, 2003; Miyoshi et al., 2013; Moreno-Castaño et al., 2023; Yu et al., 2023). Consistently, the downregulation of two major pulmonary neutrophilic chemokines CXCL-1/2 secretion by valsartan was accompanied with a reduction in neutrophil pulmonary recruitment. The consistency between the BEAS2B cells and mouse ALI model, and the well-known important roles of epithelial cells in sensing the stimulus, including LPS, and mediating immune cell infiltration, strongly suggest that valsartan ameliorates the severity of ALI through its action on alveolar epithelial cells. Nevertheless, a direct effect of valsartan on the other immune cells such as neutrophils or macrophages in the mouse ALI model, could not be ruled out. Notably, a recent study also showed that valsartan mitigated sepsis-induced lung damage via modulating macrophage (Zhang et al., 2016; Jangam et al., 2023). Additionally, previous studies have demonstrated that activation of the MAPK signaling pathway (including ERK, JNK, and p38 cascades) and NF-κB signaling pathway promote MUC5AC expression as well as inflammatory cytokines/chemokines expression in human airway epithelial cells (Ha et al., 2008; Nie et al., 2012; Tao et al., 2023; Zhou et al., 2023). Therefore, valsartan may inhibit the expression of MUC5AC and cytokines/chemokines by downregulating the MAPK and NF-κB signaling pathways in LPS-induced ALI model in this study.

In mammals, the MAPK family plays a crucial role in various cellular functions, including inflammation, stress response, cell proliferation, and apoptosis (Efferth and Oesch, 2021; Li et al., 2022). NF-κΒ is a major regulator of inflammation, and the severity and lethality of pneumonia- or sepsis-induced ALI/ARDS is primarily related to the NF-κΒ-mediated “cytokine storm” (Heine and Zamyatina, 2022). The NF-κB and MAPK signaling pathways have been shown to act downstream of LPS-TLR4 in mediating inflammation and lung injury (Matute-Bello et al., 2011; Zeng et al., 2023). Our results from network pharmacology and KEGG analysis revealed that the NF-κB and MAPK signaling pathways were among the predicted enriched pathways regulated by valsartan in LPS-induced ALI (Figure 7). We performed further experiments and confirmed that valsartan inhibits LPS-induced P38, JNK, ERK, and P65 phosphorylation in both BEAS2B cells and the lung tissue of ALI mouse model in a dose-dependent manner (Figure 8). Notably, our finding demonstrates that MAPK and NF-κB signaling are involved in the modulation of valsartan on ALI. However, the current results are insufficient to fully support the argument that valsartan regulates ALI in an NF-κB and MAPK-dependent manner, which warrants further investigation. Additionally, a large body of literature has shown that LPS-induced activation of TLR4 is an early signaling event in LPS-induced ALI models, initiating various signaling pathways, including NF-κΒ and MAPK, further leading to the production of inflammatory mediators (Na et al., 2019; Liu et al., 2022; Yue et al., 2023). Here, we demonstrate regulatory effects of valsartan on NF-κΒ and MAPKs. Whether valsartan regulates the expression or activities of TLR4 requires further investigation.

Valsartan is an angiotensin II type 1 receptor antagonist that is commonly used to clinically control blood pressure. Previous studies have indicated that it has anti-inflammatory and anti-oxidative effects. Valsartan prevents gefitinib-induced lung inflammation and oxidative stress, modulates plasma metabolites in rats, and attenuates gefitinib-induced cardiac hypertrophy in rat by modulating angiotensin II-mediated oxidative stress and the JNK/P38 pathway (Nie et al., 2012; Tao et al., 2023). Additionally, telmisartan, another angiotensin II type 1 receptor antagonist, has been found to attenuate LPS-induced MUC5AC production by upregulating cytokine signaling inhibitory factor 1 (SOCS1) (Chen et al., 2021). However, it remains unclear whether and how valsartan affects LPS-induced ALI. In this study, we demonstrated another potential effect of valsartan in an experimental mouse model of lung injury. It is worth noting that ALI/ARDS in humans is tipically caused by a combination of intra- and extra-pulmonary pathogenic factors (Zaki et al., 2022). Therefore, a single pathogenic LPS-induced mouse model of ALI cannot replicate all the pathological features of human ALI/ARDS. However, our study suggests that Valsartan treatment can be used to reduce the progression of ALI and control blood pressure in ALI patients who also have hypertension. It is important to note that most ALI/ARDS patients experience multi-organ damage, such as hypovolemic shock, renal failure, and electrolyte disorders. Valsartan treatment may result in hypotension, transient deterioration of renal function, hyperkalemia, and other side effects. Therefore, it is essential to monitor blood pressure, renal function, and electrolytes when using valsartan to treat ALI/ARDS in clinical settings. In conclusion, our *in vitro* and *in vivo* experiments have proposed a new therapeutic role for valsartan in the prevention of ALI and provided insights into the development of drugs for ALI prevention.

## Data Availability

The original contributions presented in the study are included in the article/Supplementary Material, further inquiries can be directed to the corresponding authors.
