# Supplementary figures and images for: Valsartan attenuates LPS-induced ALI by modulating NF-κB and MAPK pathways (part 1 of 4)
Source: Front Pharmacol. 2024 Jan 15;15:1321095. doi: 10.3389/fphar.2024.1321095 (PMC10822936; doi:10.3389/fphar.2024.1321095)

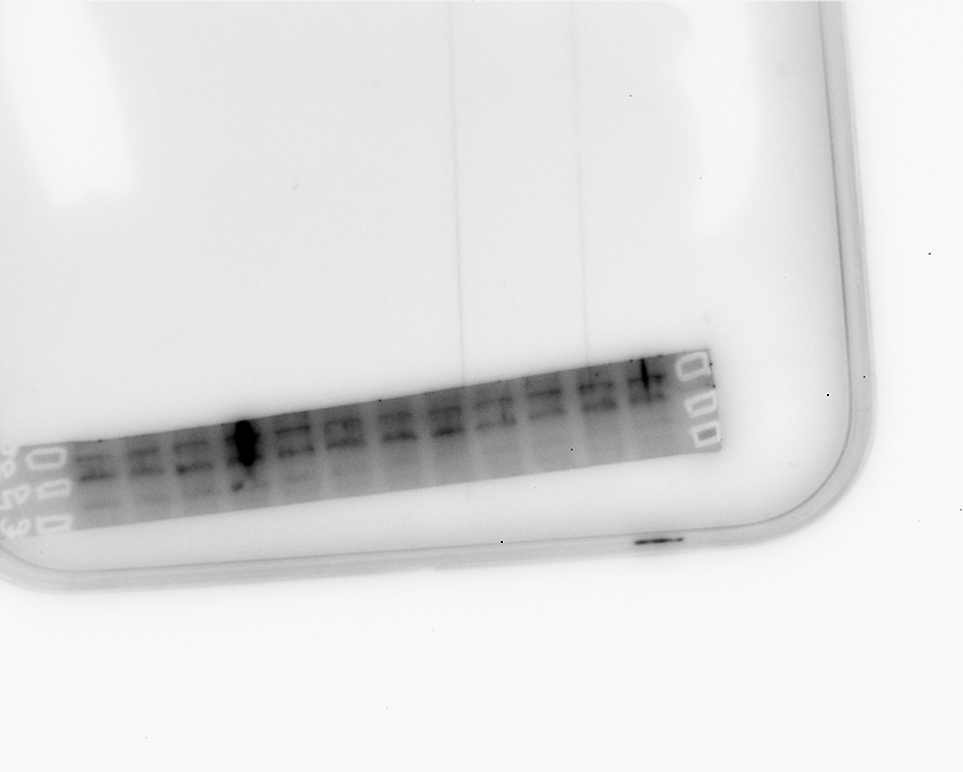

Supplement: Supplementary file 2 [file DataSheet3.ZIP › JNK1/JNK 1.tif]

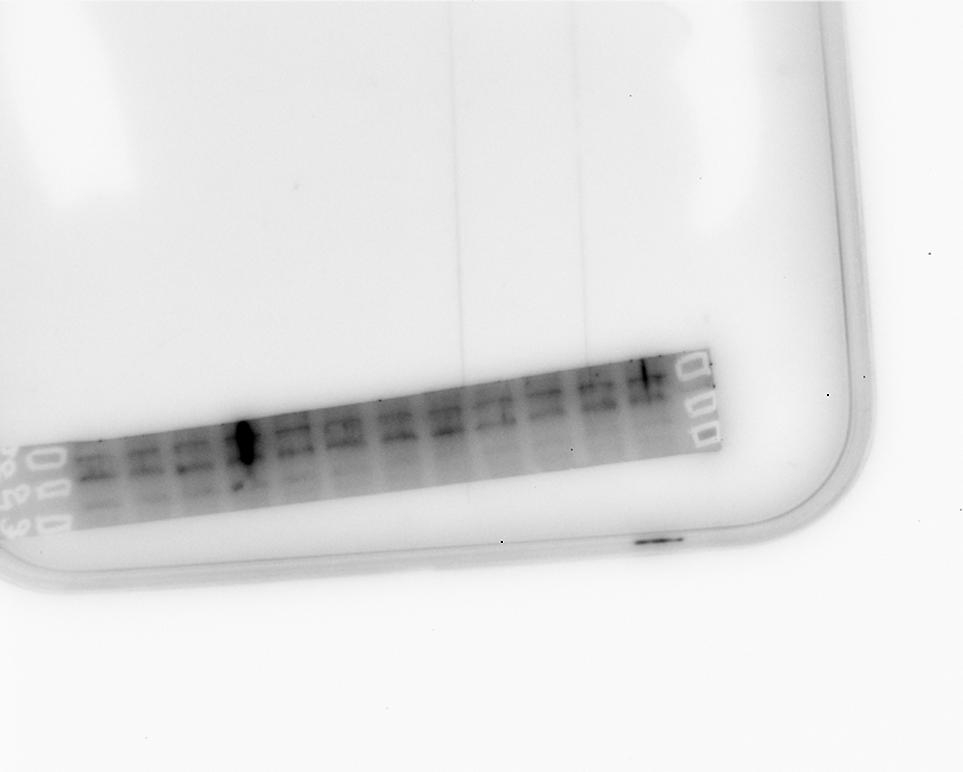

Supplement: Supplementary file 2 [file DataSheet3.ZIP › JNK1/JNK 2.tif]

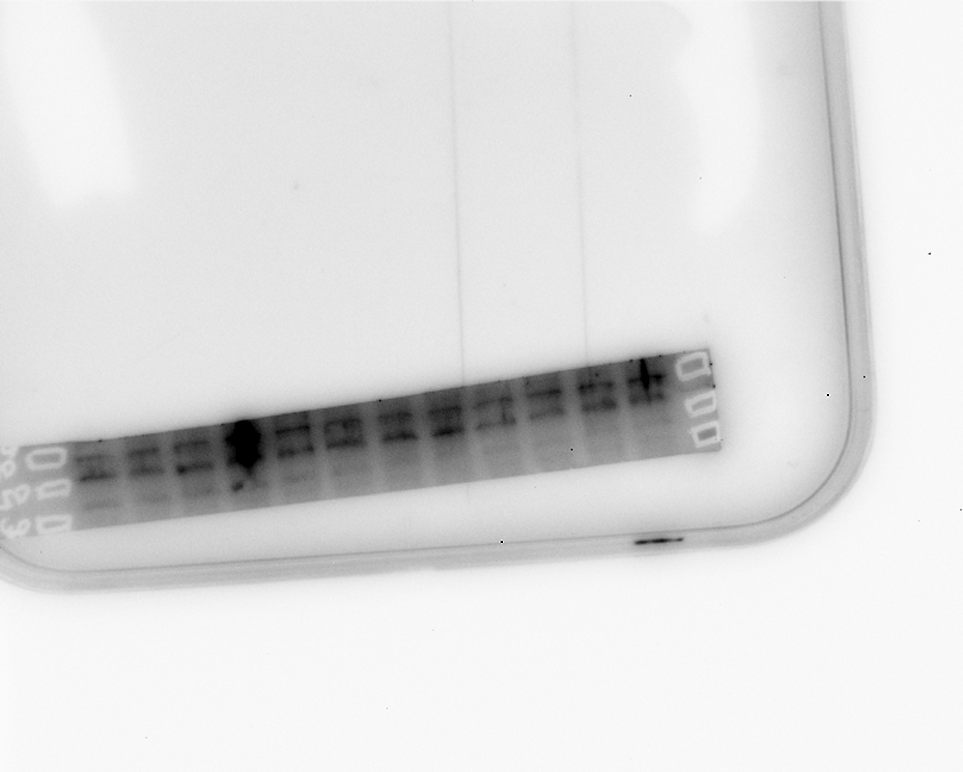

Supplement: Supplementary file 2 [file DataSheet3.ZIP › JNK1/JNK 3.tif]

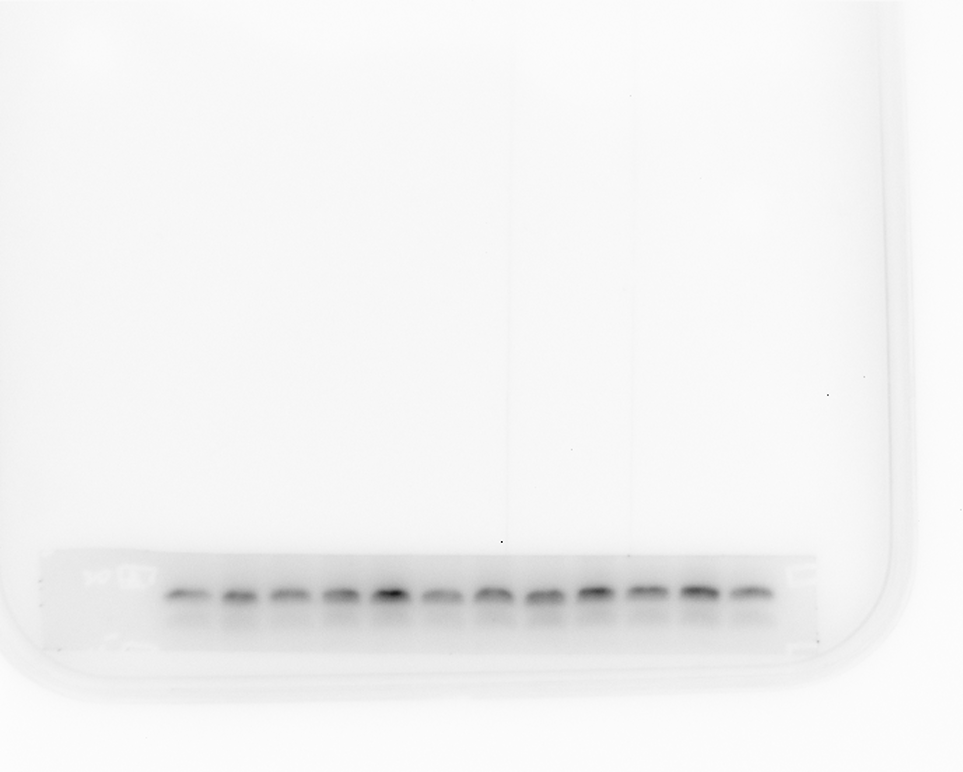

Supplement: Supplementary file 2 [file DataSheet3.ZIP › JNK1/JNK CYPB 1.tif]

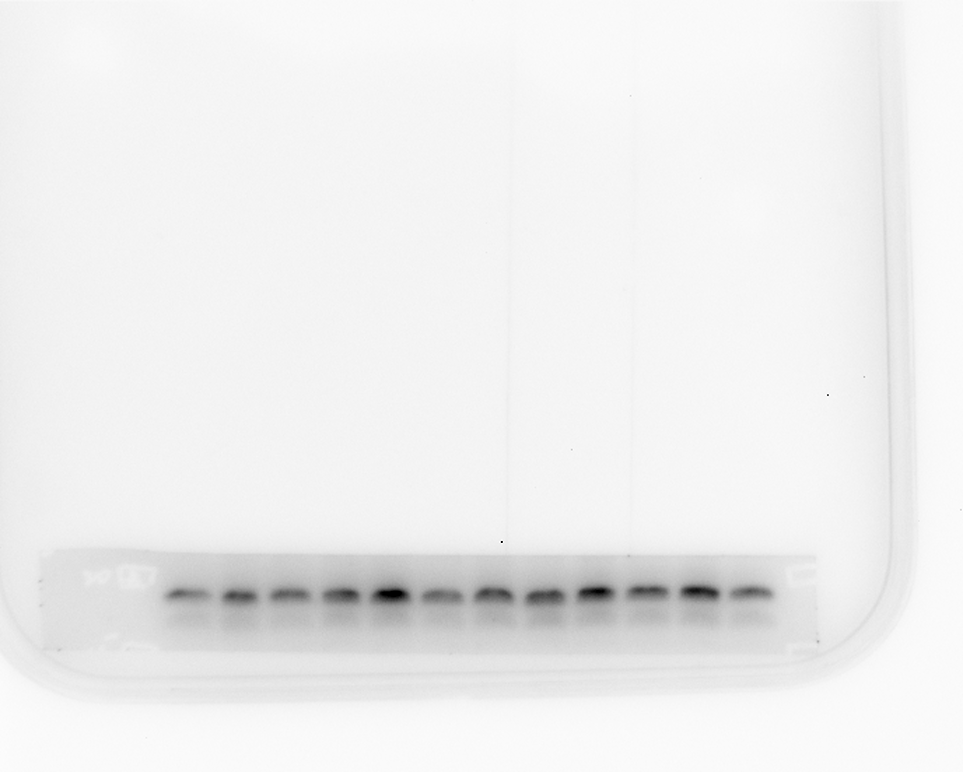

Supplement: Supplementary file 2 [file DataSheet3.ZIP › JNK1/JNK CYPB 2.tif]

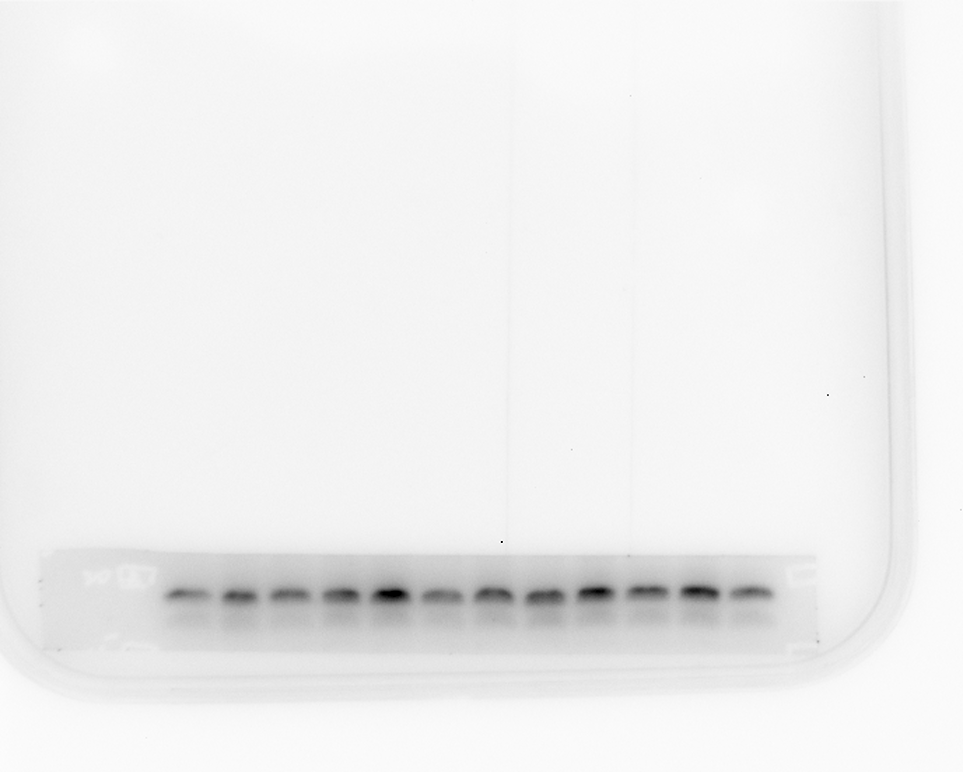

Supplement: Supplementary file 2 [file DataSheet3.ZIP › JNK1/JNK CYPB 4.tif]

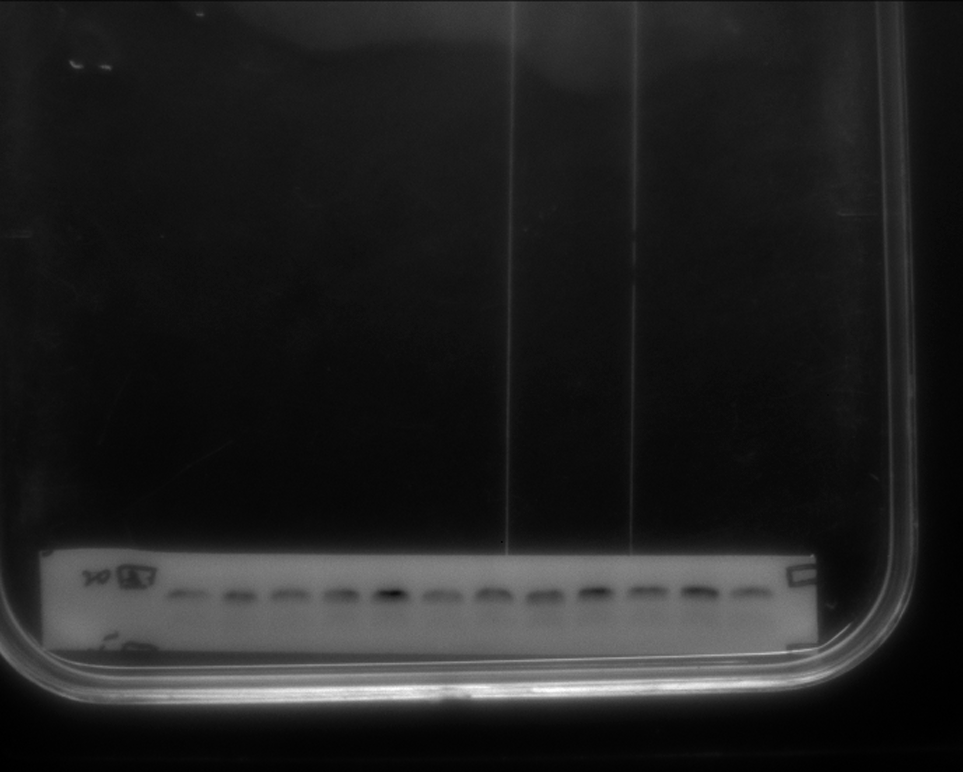

Supplement: Supplementary file 2 [file DataSheet3.ZIP › JNK1/JNK CYPB q.tif]

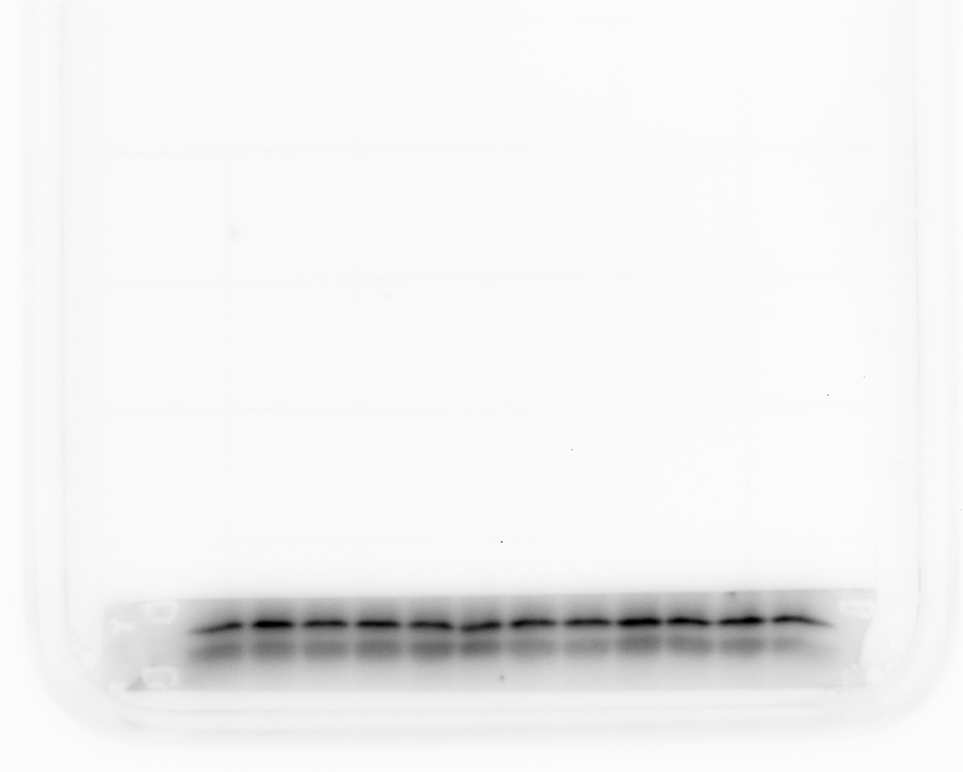

Supplement: Supplementary file 2 [file DataSheet3.ZIP › JNK1/p-JNK CYPB 1.tif]

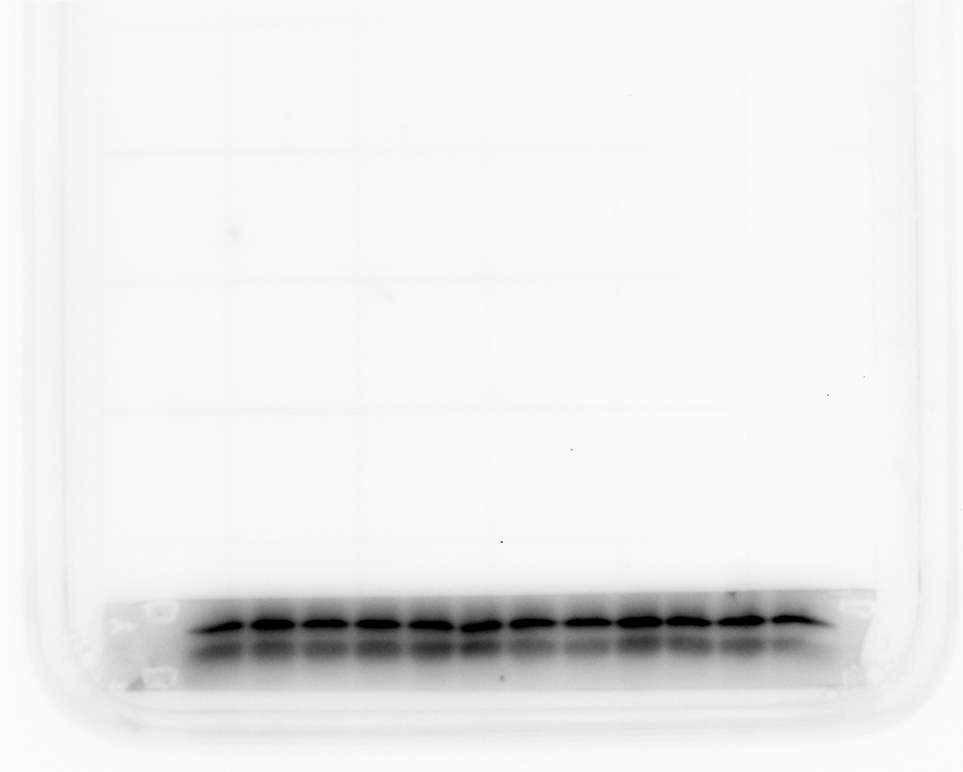

Supplement: Supplementary file 2 [file DataSheet3.ZIP › JNK1/p-JNK CYPB 2.tif]

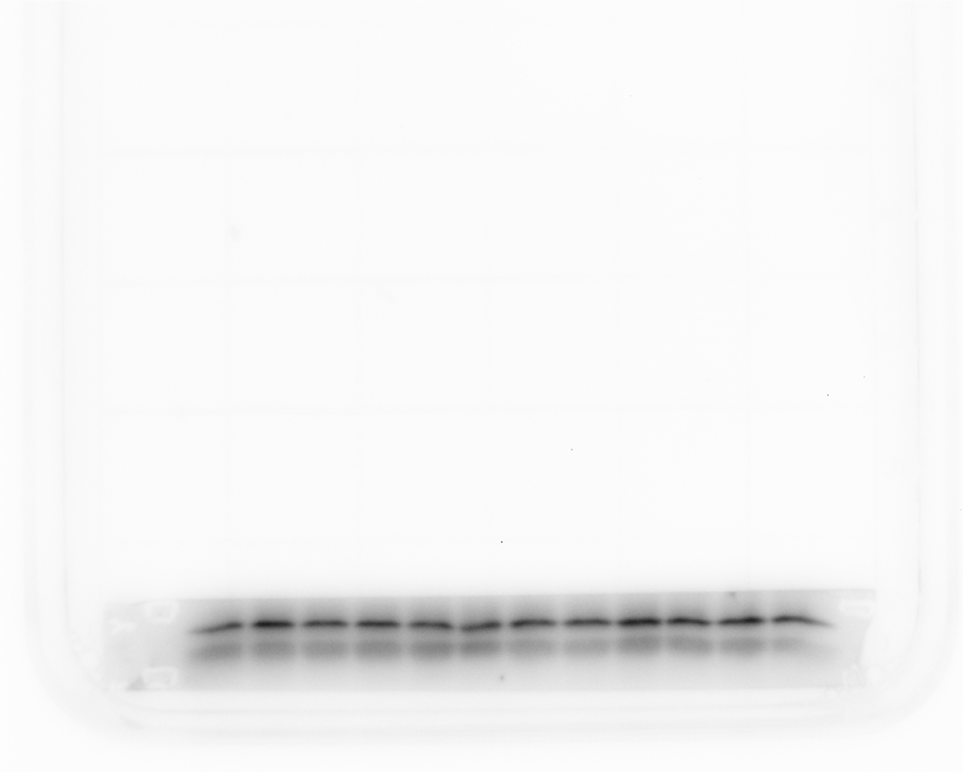

Supplement: Supplementary file 2 [file DataSheet3.ZIP › JNK1/p-JNK CYPB 3.tif]

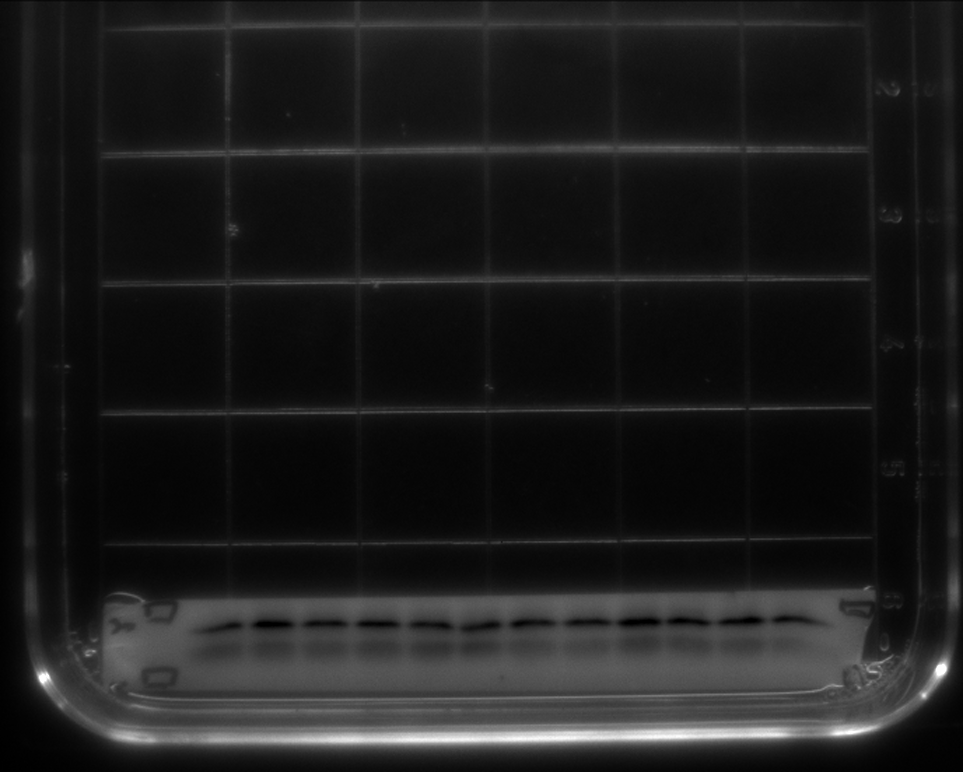

Supplement: Supplementary file 2 [file DataSheet3.ZIP › JNK1/p-JNK CYPB q.tif]

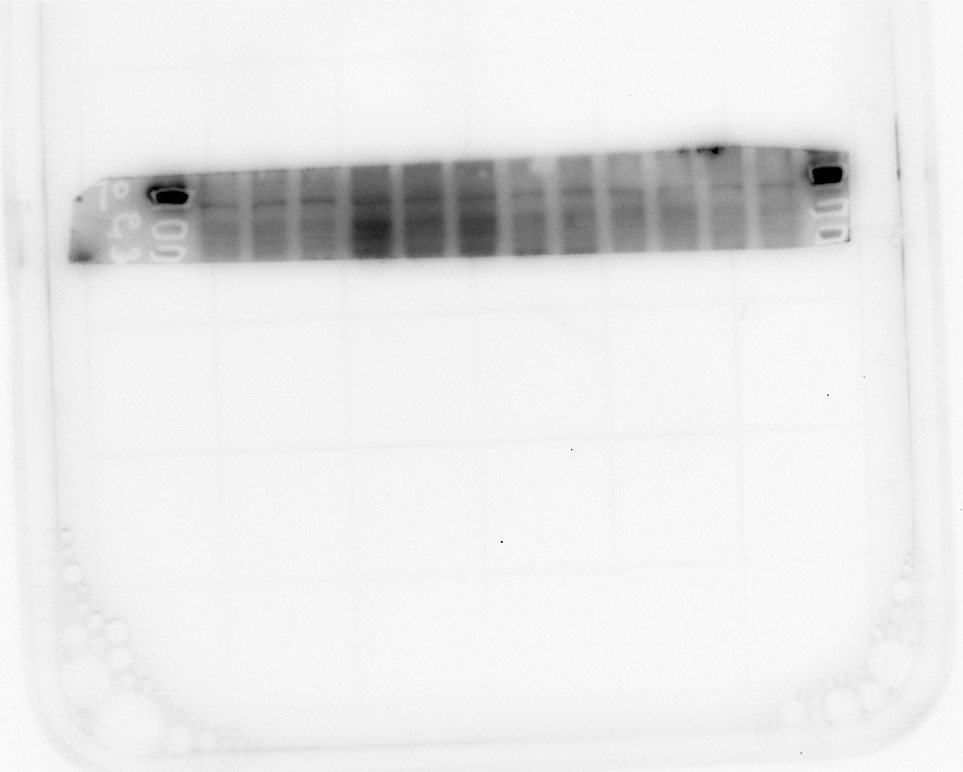

Supplement: Supplementary file 2 [file DataSheet3.ZIP › JNK1/p-JNK 1.tif]

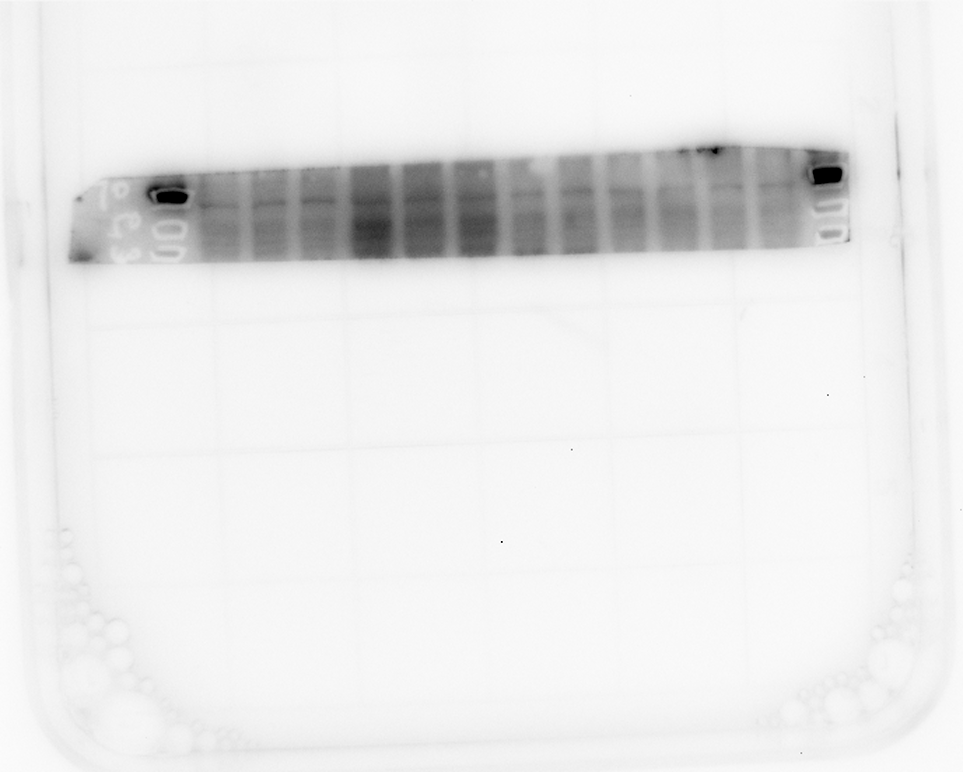

Supplement: Supplementary file 2 [file DataSheet3.ZIP › JNK1/p-JNK 2.tif]

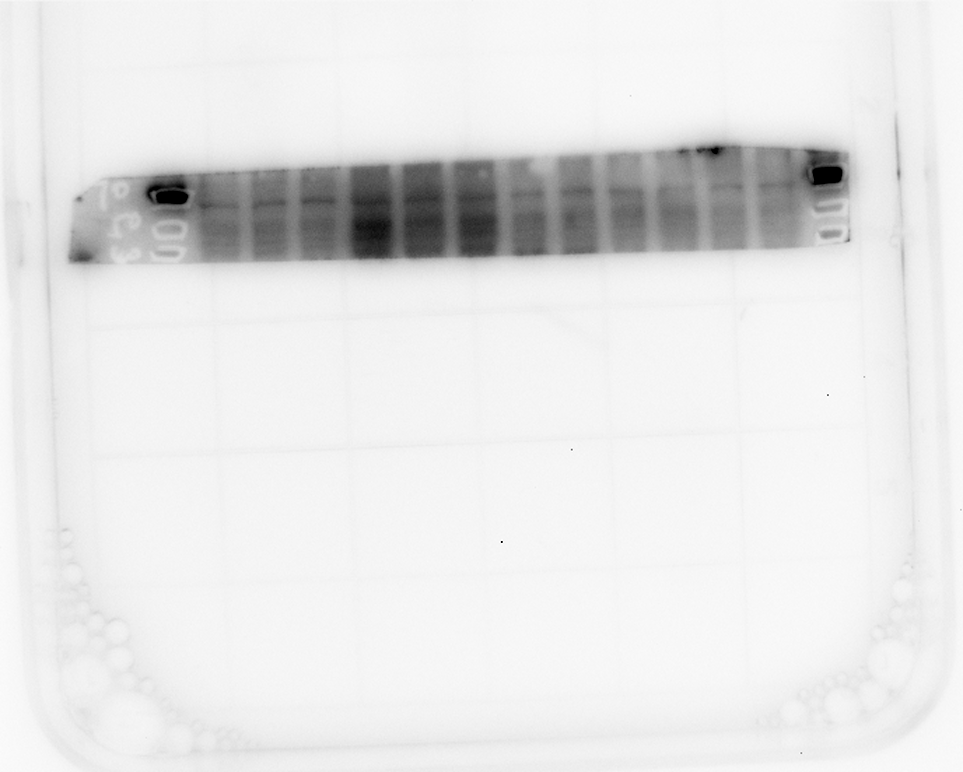

Supplement: Supplementary file 2 [file DataSheet3.ZIP › JNK1/p-JNK 3.tif]

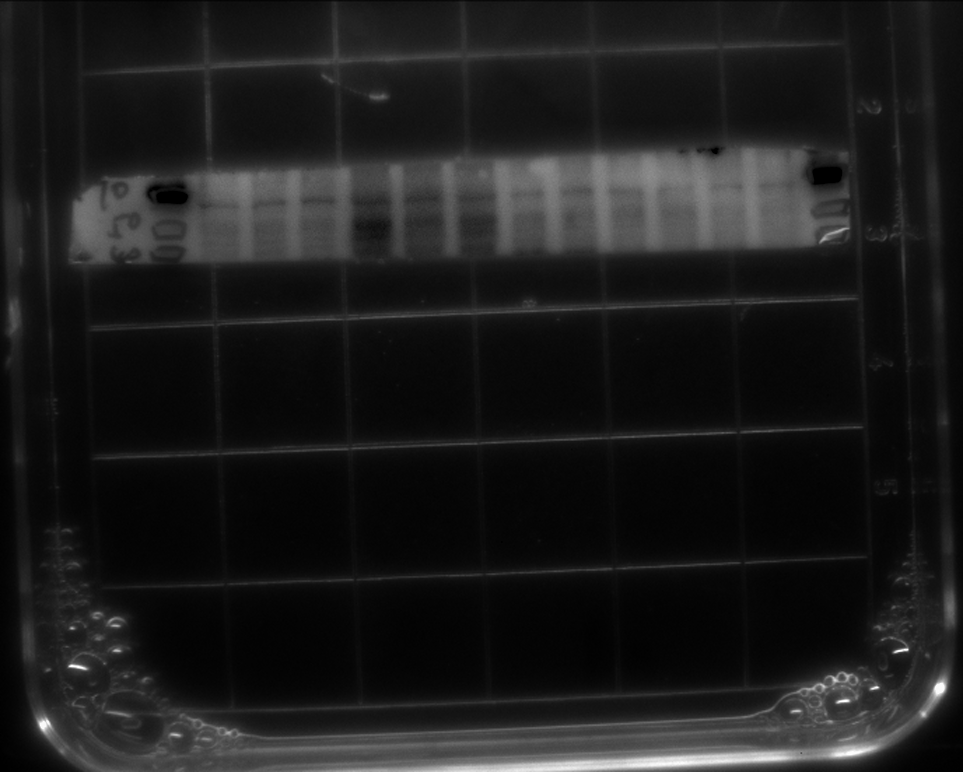

Supplement: Supplementary file 2 [file DataSheet3.ZIP › JNK1/p-JNK q.tif]

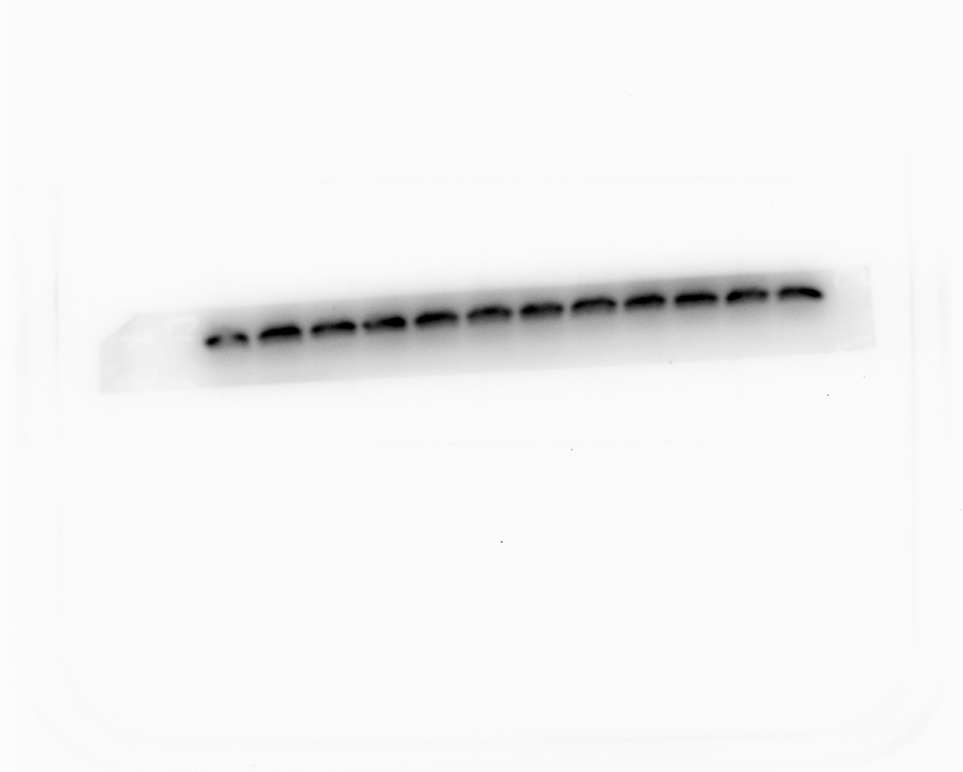

Supplement: Supplementary file 2 [file DataSheet3.ZIP › JNK2/CYPB 5.tif]

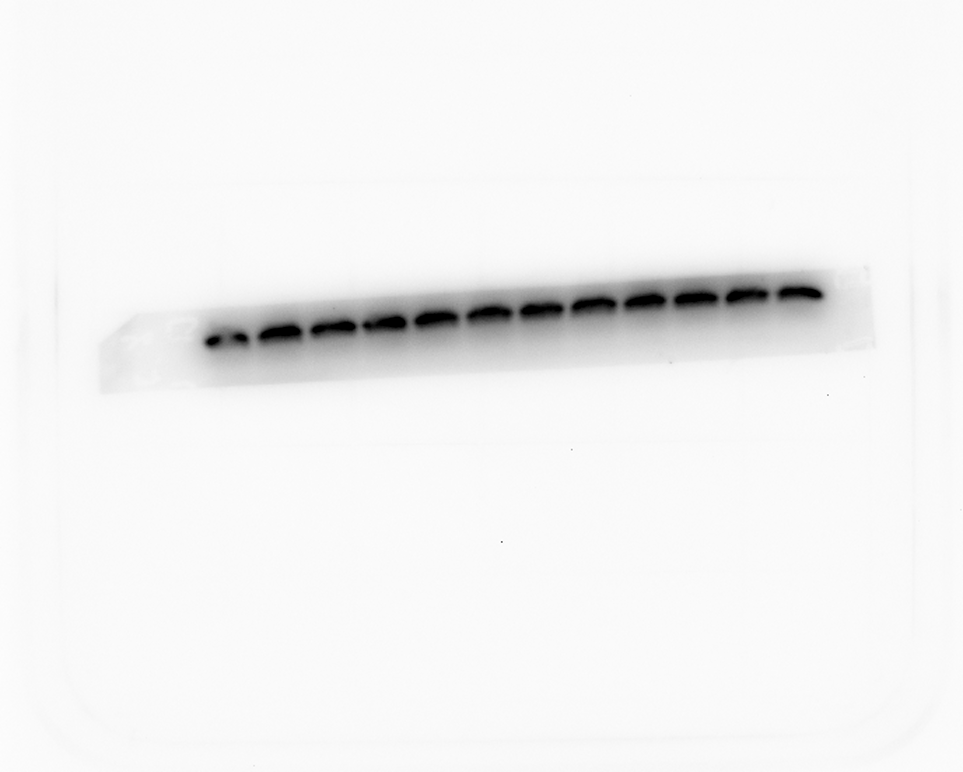

Supplement: Supplementary file 2 [file DataSheet3.ZIP › JNK2/CYPB 6.tif]

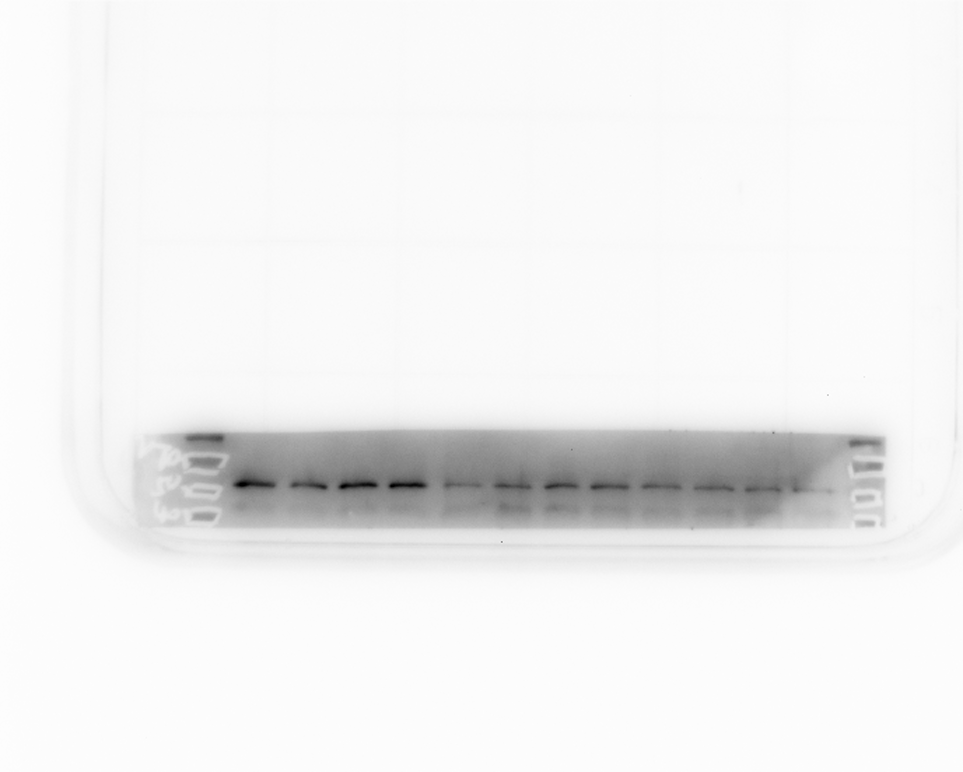

Supplement: Supplementary file 2 [file DataSheet3.ZIP › JNK2/JNK 1.tif]

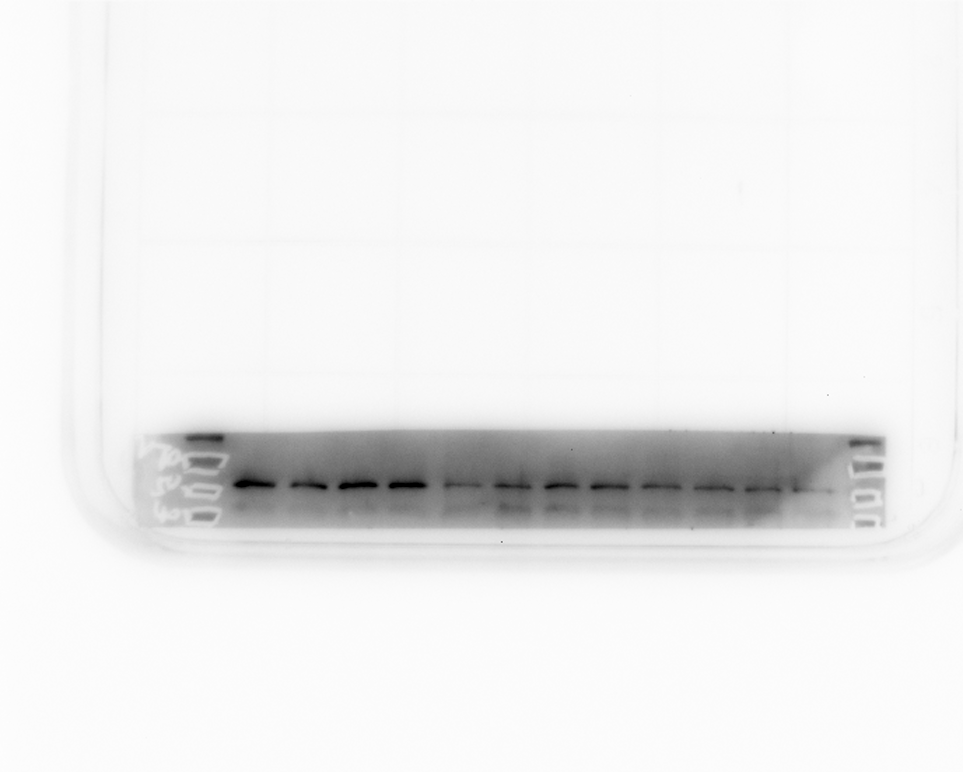

Supplement: Supplementary file 2 [file DataSheet3.ZIP › JNK2/JNK 3.tif]

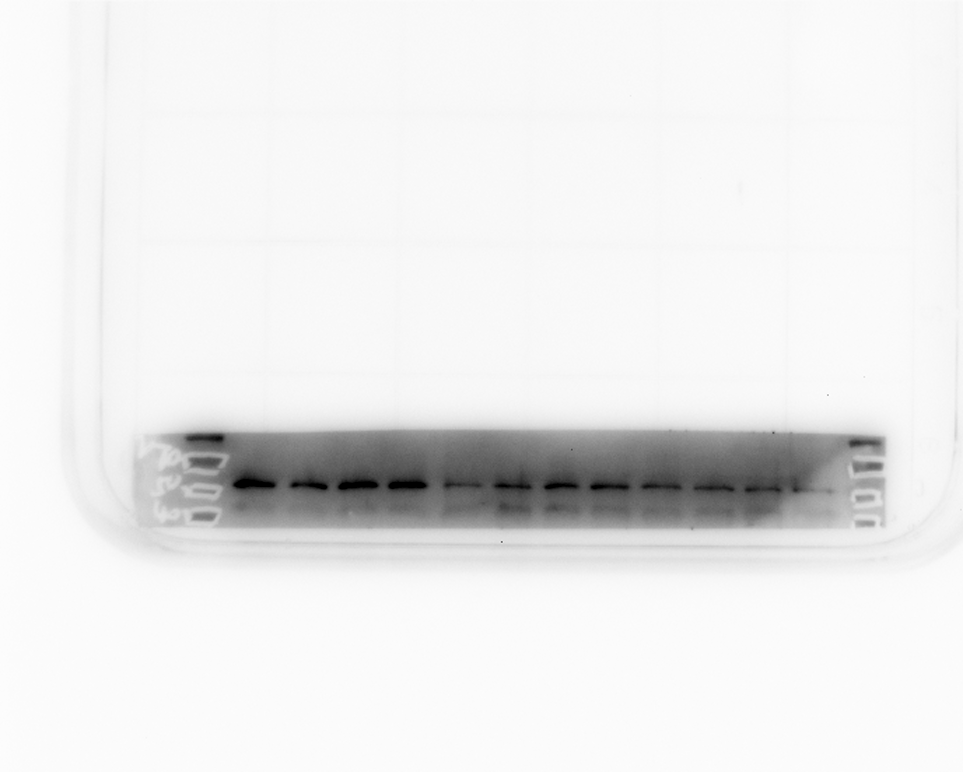

Supplement: Supplementary file 2 [file DataSheet3.ZIP › JNK2/JNK 4.tif]

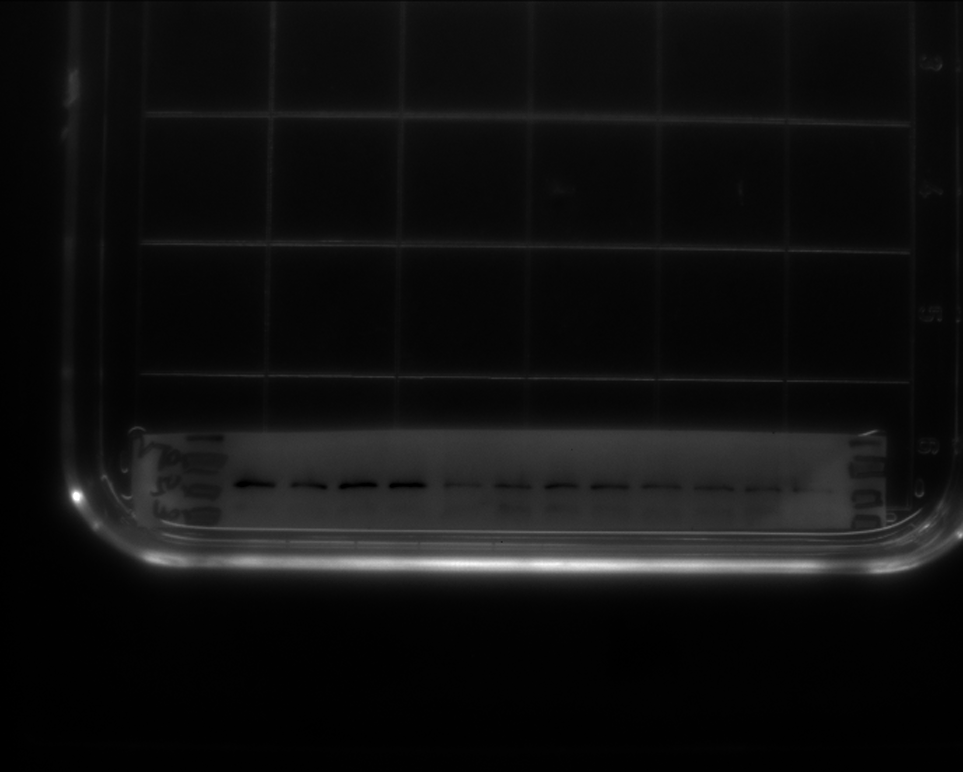

Supplement: Supplementary file 2 [file DataSheet3.ZIP › JNK2/JNK q.tif]

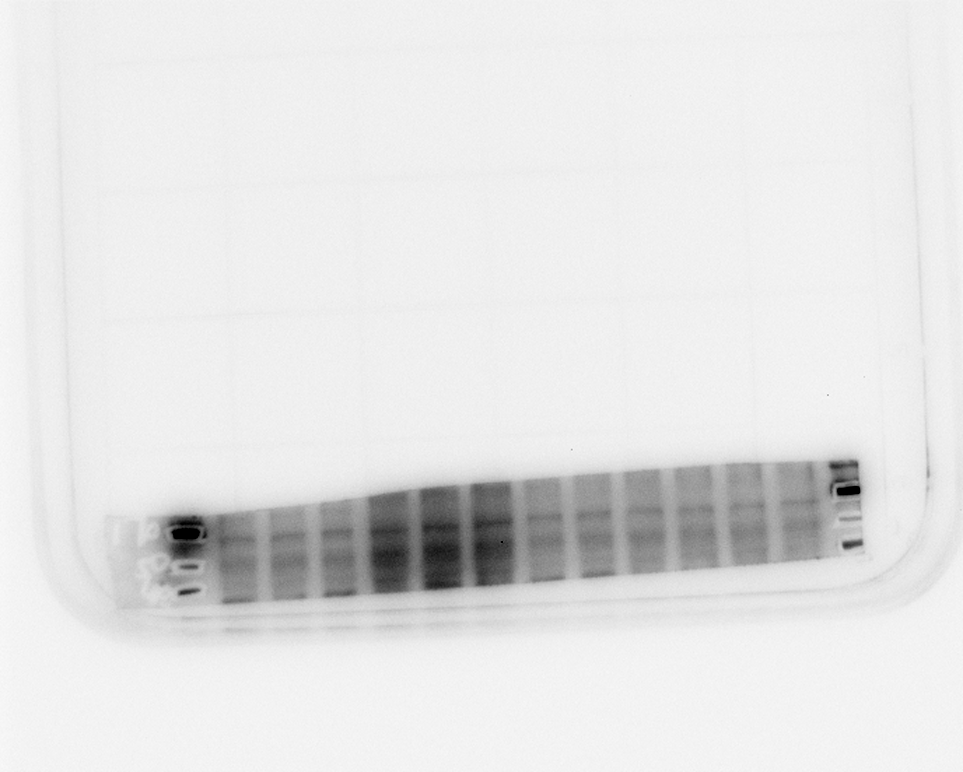

Supplement: Supplementary file 2 [file DataSheet3.ZIP › JNK2/p-JNK--2.tif]

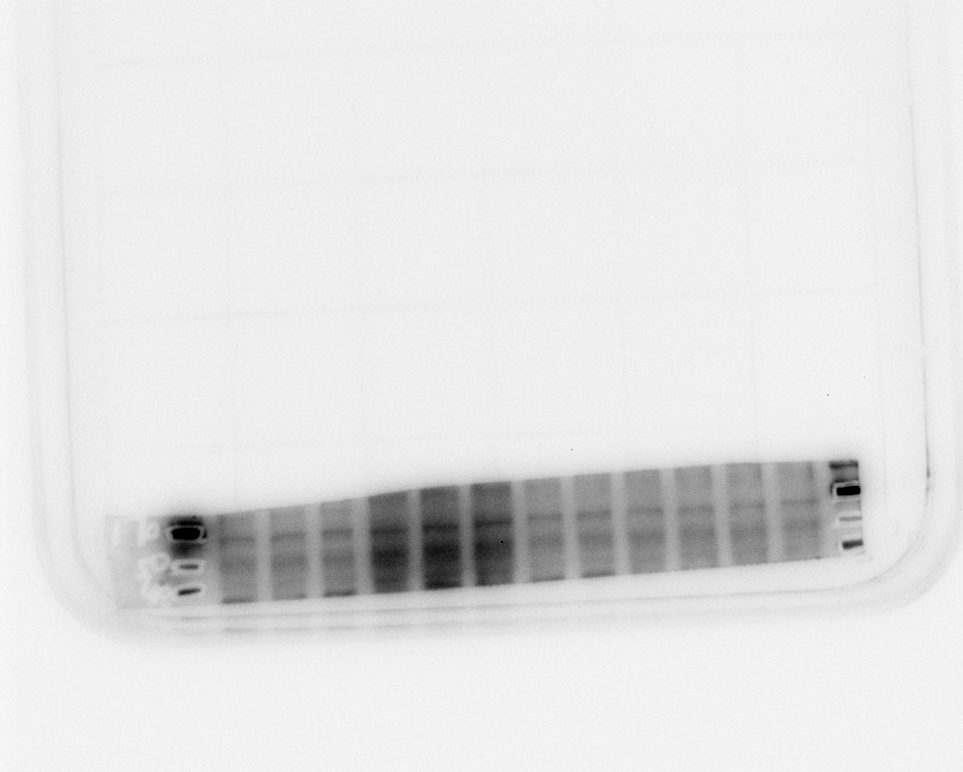

Supplement: Supplementary file 2 [file DataSheet3.ZIP › JNK2/p-JNK--4.tif]

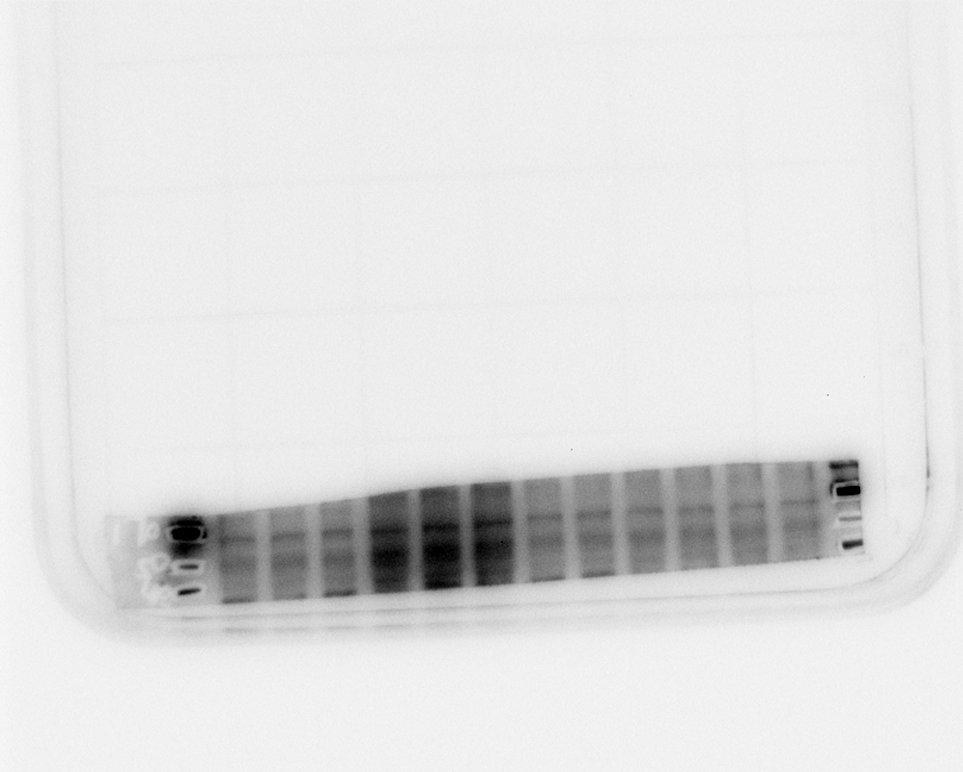

Supplement: Supplementary file 2 [file DataSheet3.ZIP › JNK2/p-JNK--5.tif]

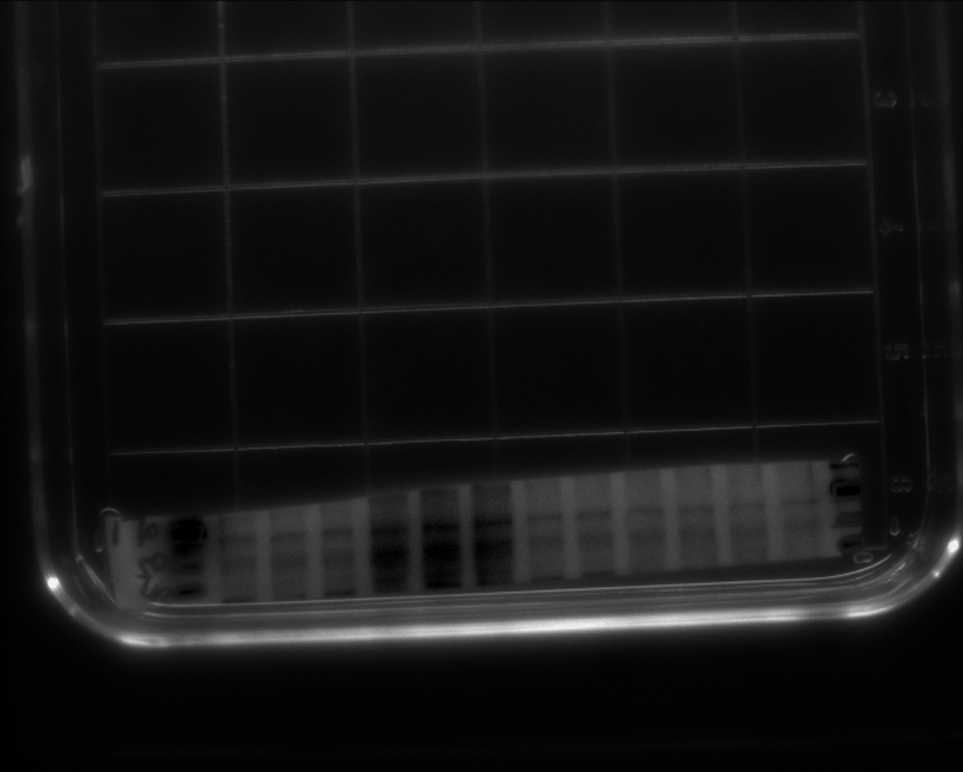

Supplement: Supplementary file 2 [file DataSheet3.ZIP › JNK2/p-JNK--q.tif]

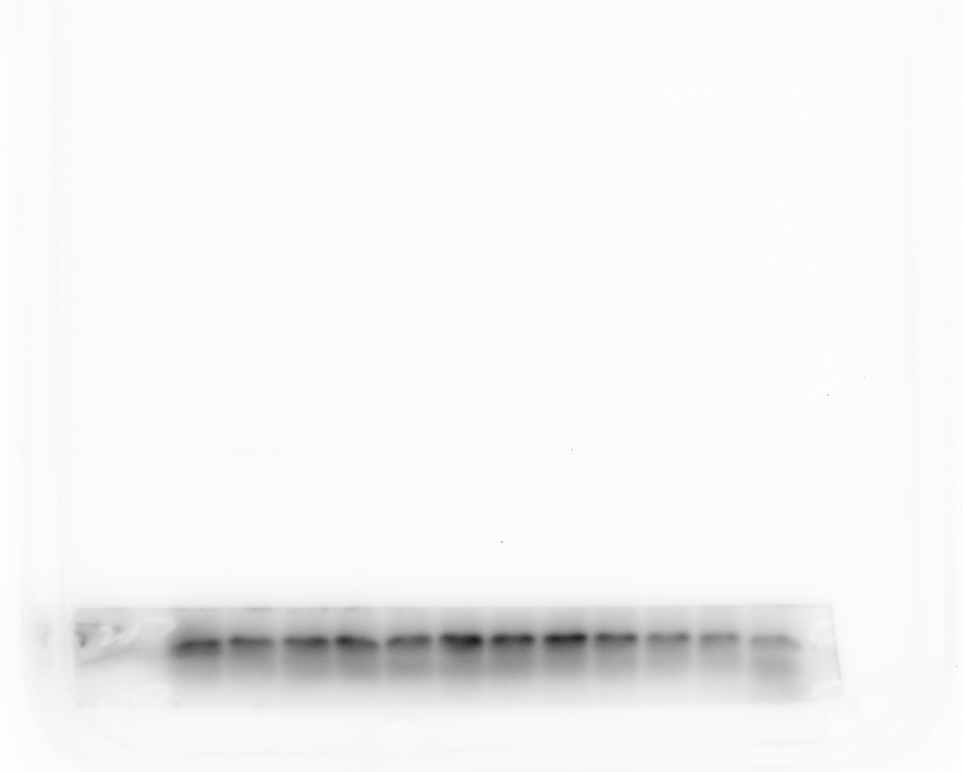

Supplement: Supplementary file 2 [file DataSheet3.ZIP › JNK2/P-jnkCYPB 1.tif]

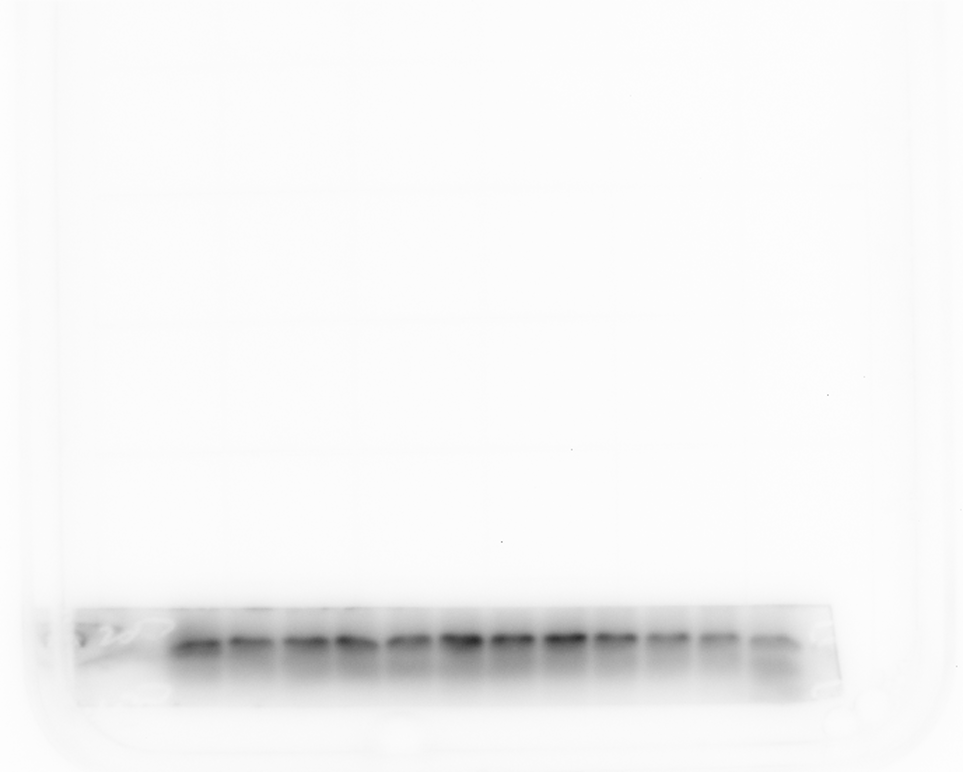

Supplement: Supplementary file 2 [file DataSheet3.ZIP › JNK2/P-jnkCYPB 2.tif]

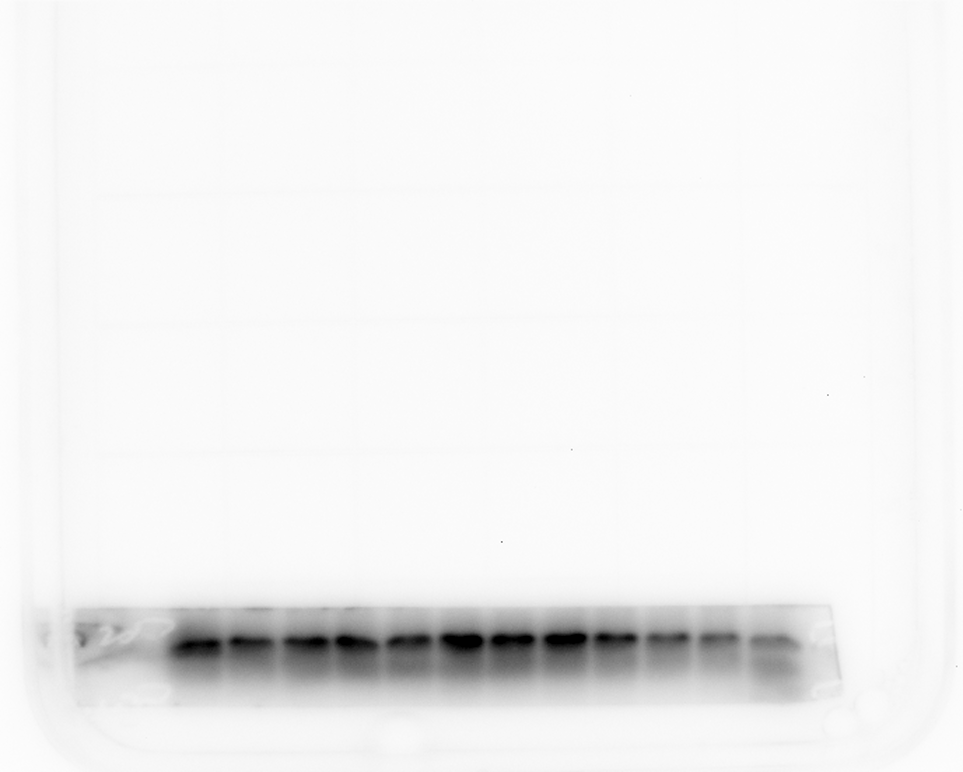

Supplement: Supplementary file 2 [file DataSheet3.ZIP › JNK2/P-jnkCYPB 7.tif]

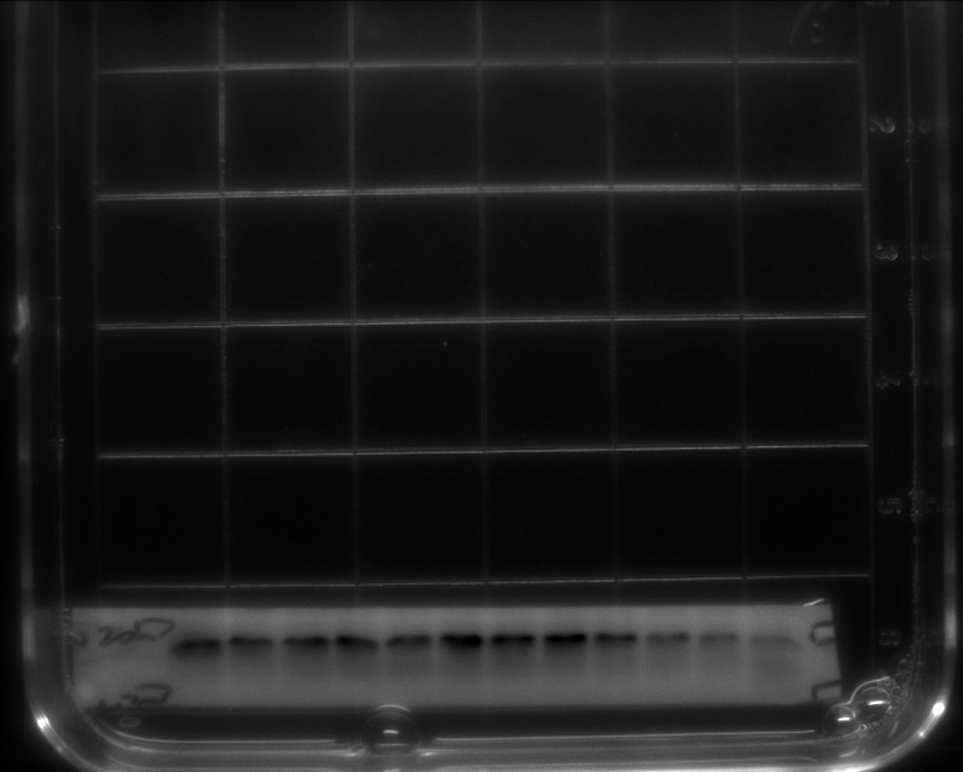

Supplement: Supplementary file 2 [file DataSheet3.ZIP › JNK2/P-jnkCYPB q.tif]

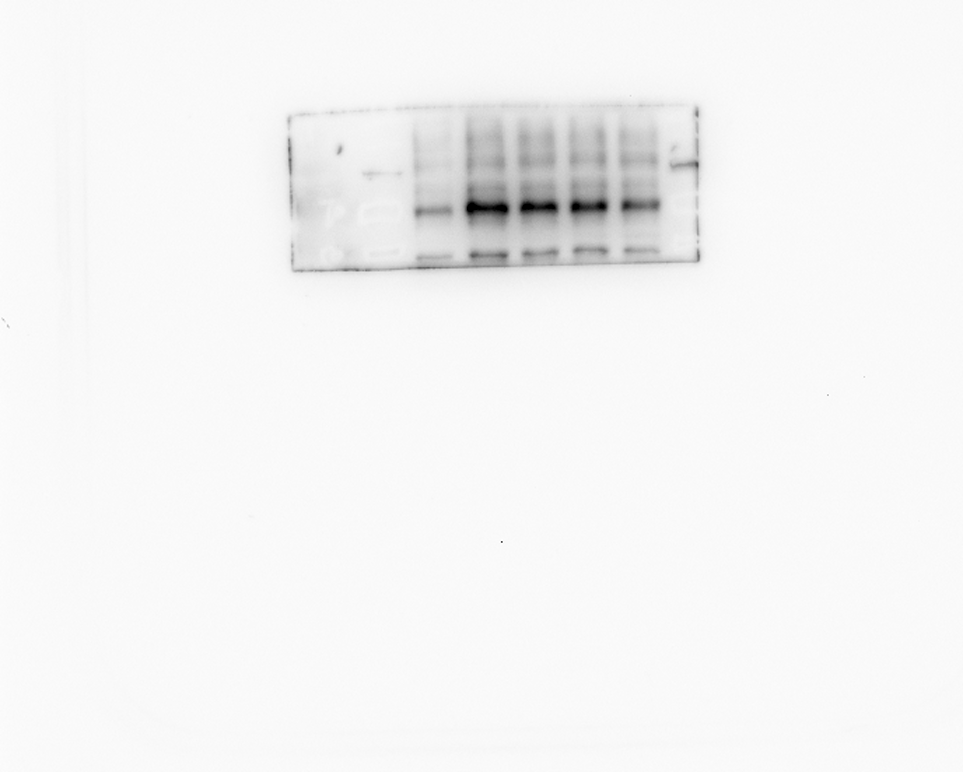

Supplement: Supplementary file 3 [file DataSheet11.ZIP › 1/p-p65 1.tif]

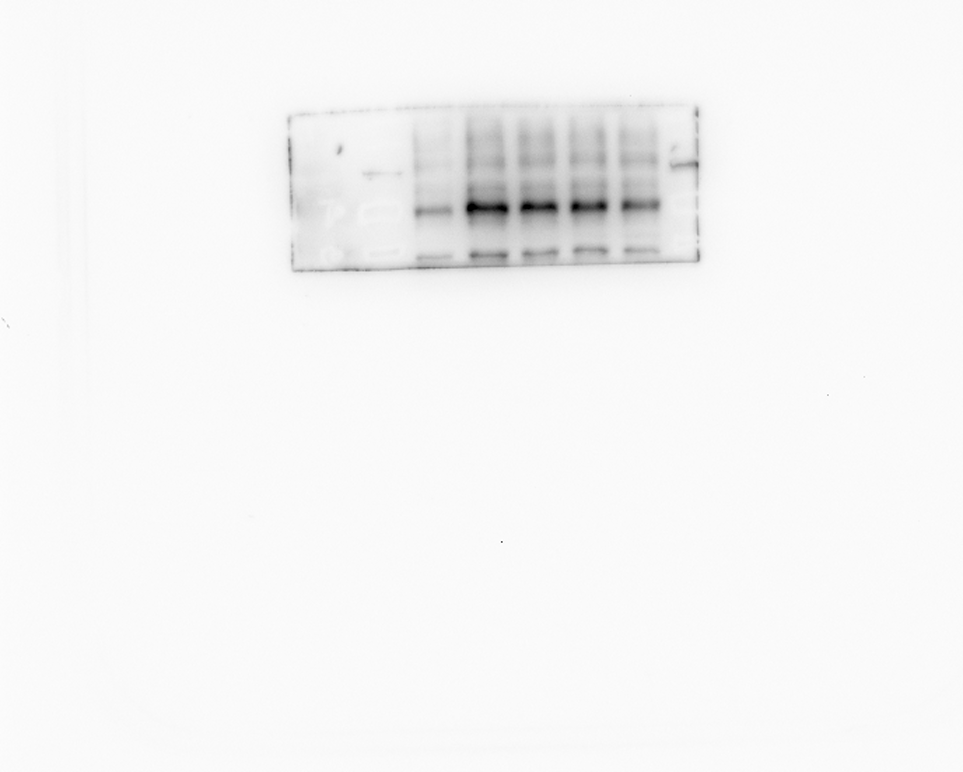

Supplement: Supplementary file 3 [file DataSheet11.ZIP › 1/p-p65 2.tif]

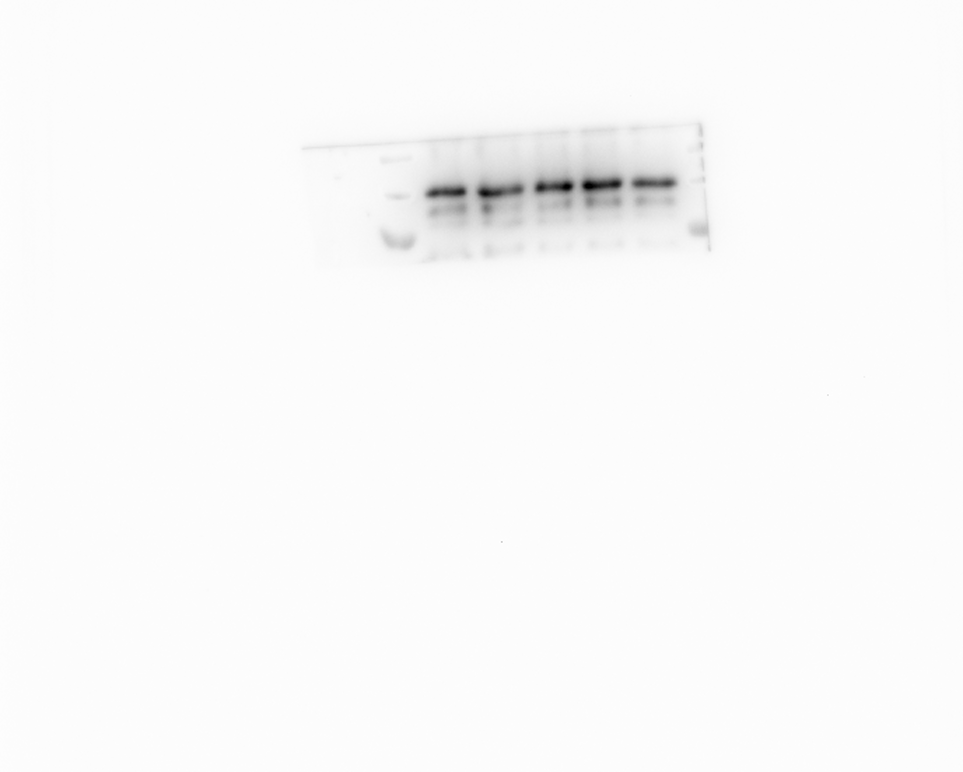

Supplement: Supplementary file 3 [file DataSheet11.ZIP › 1/p-p65 GAPDH 1.tif]

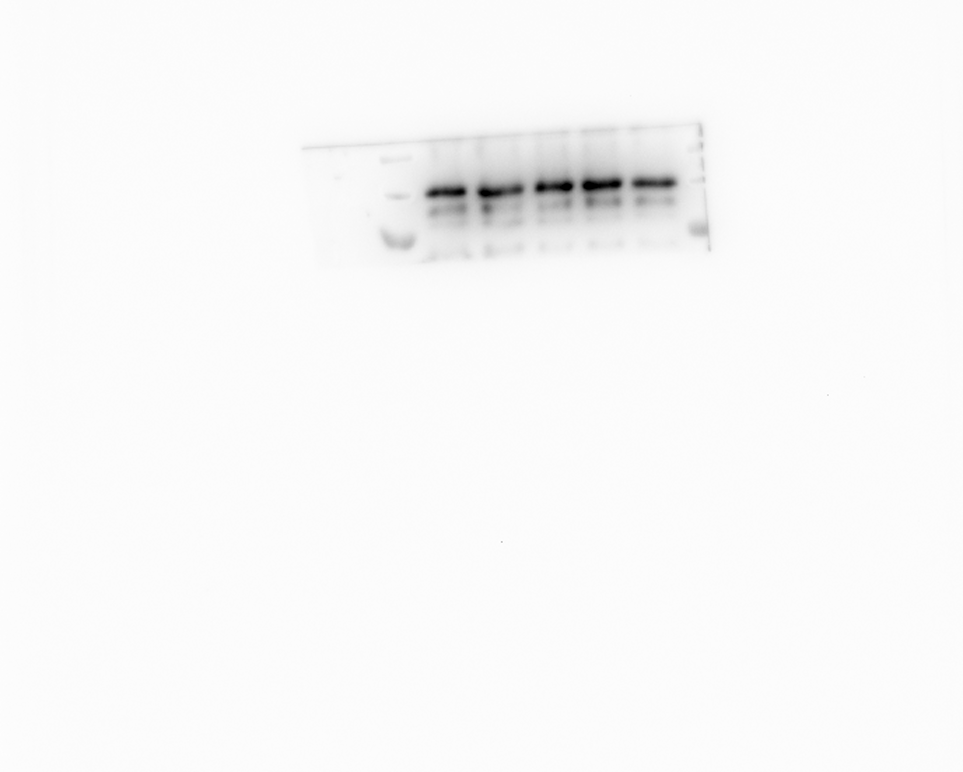

Supplement: Supplementary file 3 [file DataSheet11.ZIP › 1/p-p65 GAPDH 3.tif]

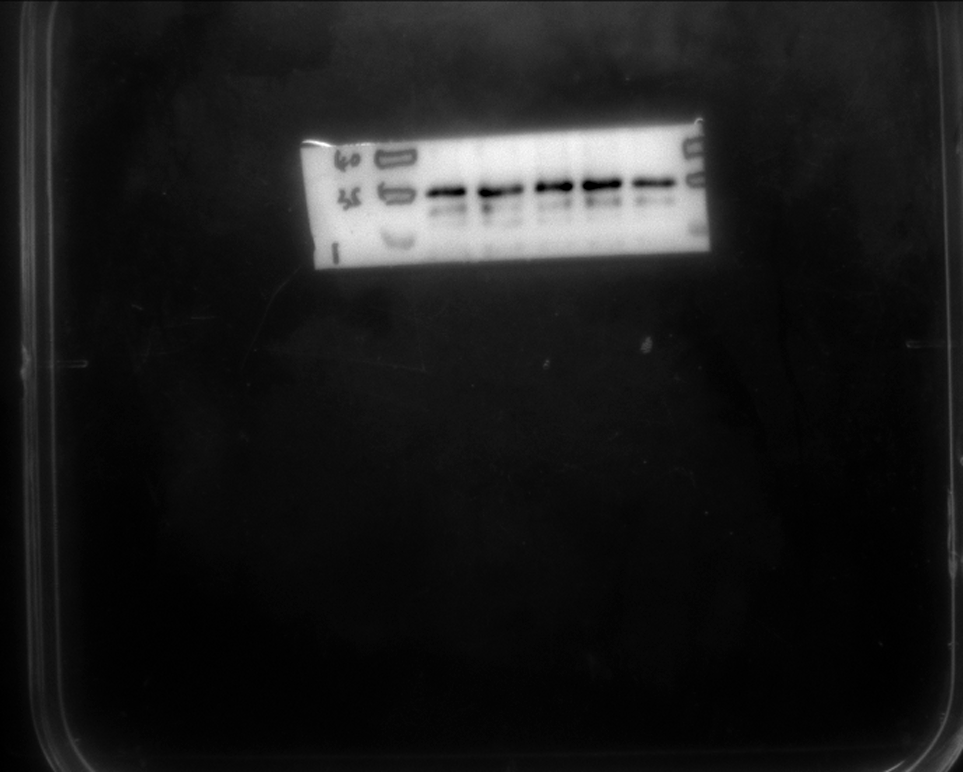

Supplement: Supplementary file 3 [file DataSheet11.ZIP › 1/p-p65 GAPDH q.tif]

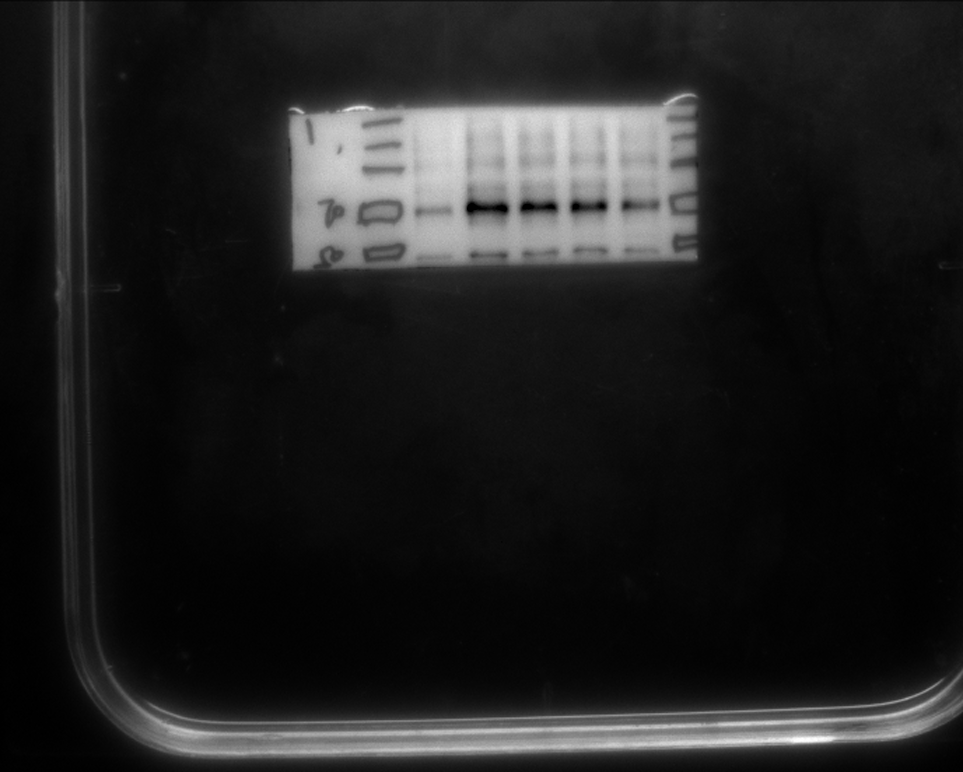

Supplement: Supplementary file 3 [file DataSheet11.ZIP › 1/p-p65.tif]

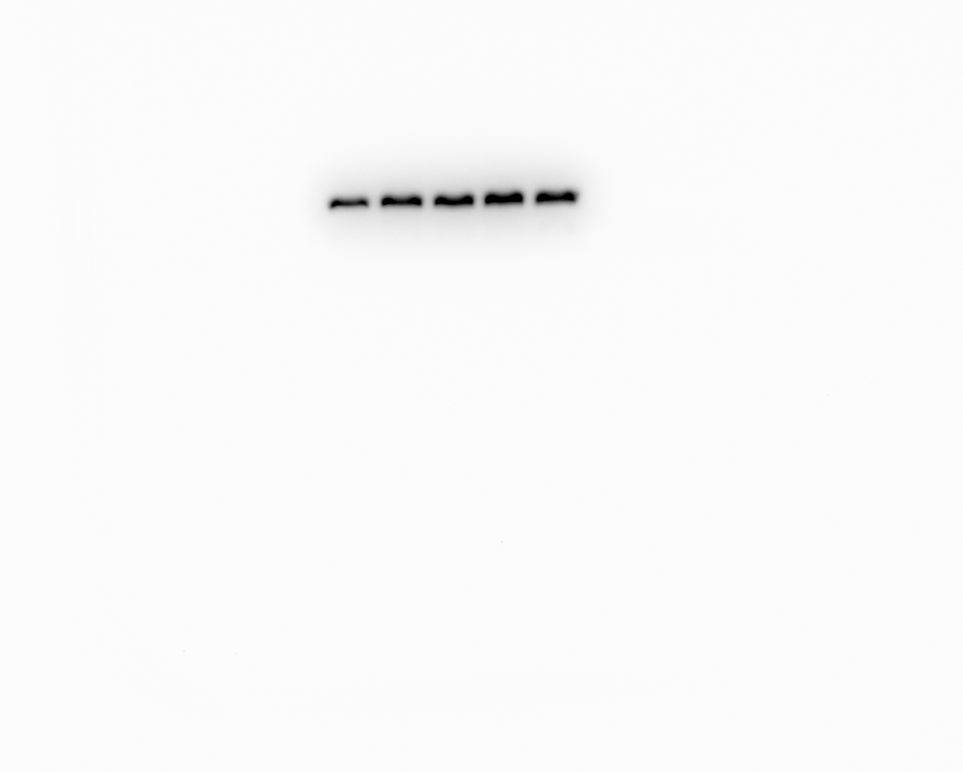

Supplement: Supplementary file 3 [file DataSheet11.ZIP › 1/p65 1.tif]

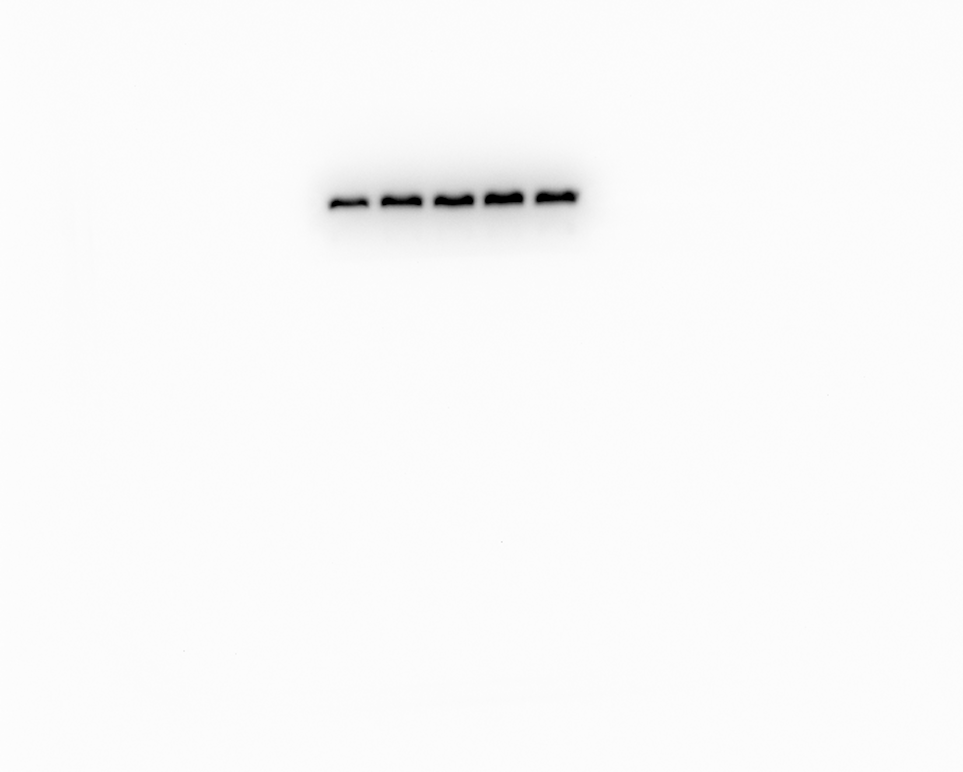

Supplement: Supplementary file 3 [file DataSheet11.ZIP › 1/p65 2.tif]

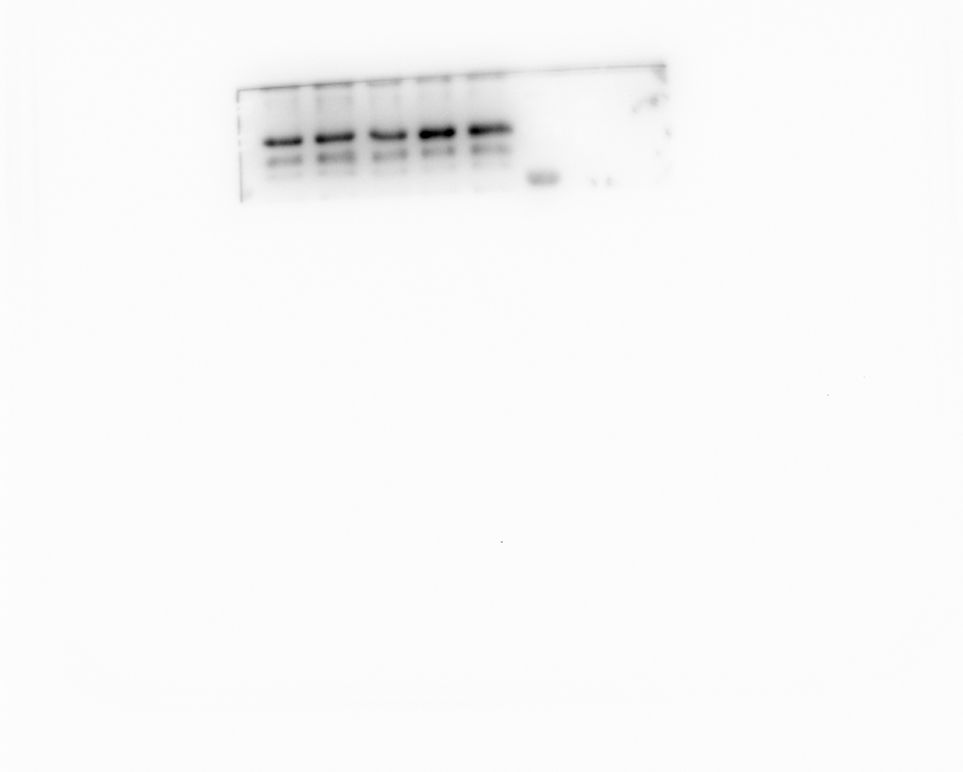

Supplement: Supplementary file 3 [file DataSheet11.ZIP › 1/p65 GAPDH 2.tif]

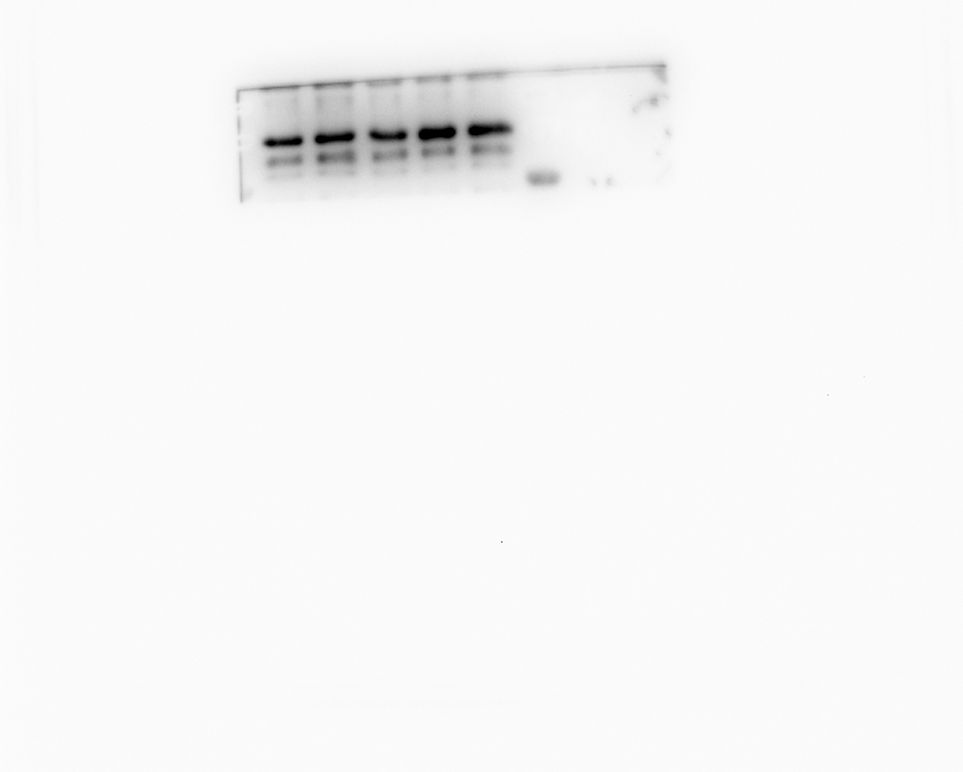

Supplement: Supplementary file 3 [file DataSheet11.ZIP › 1/p65 GAPDH 3.tif]

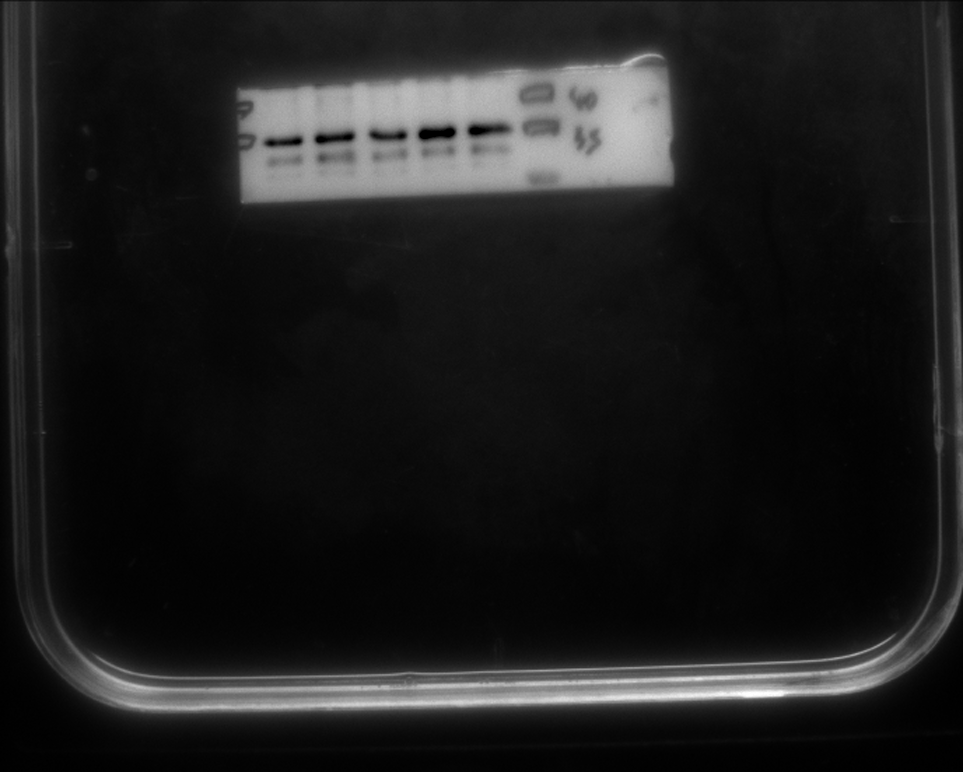

Supplement: Supplementary file 3 [file DataSheet11.ZIP › 1/p65 GAPDH q.tif]

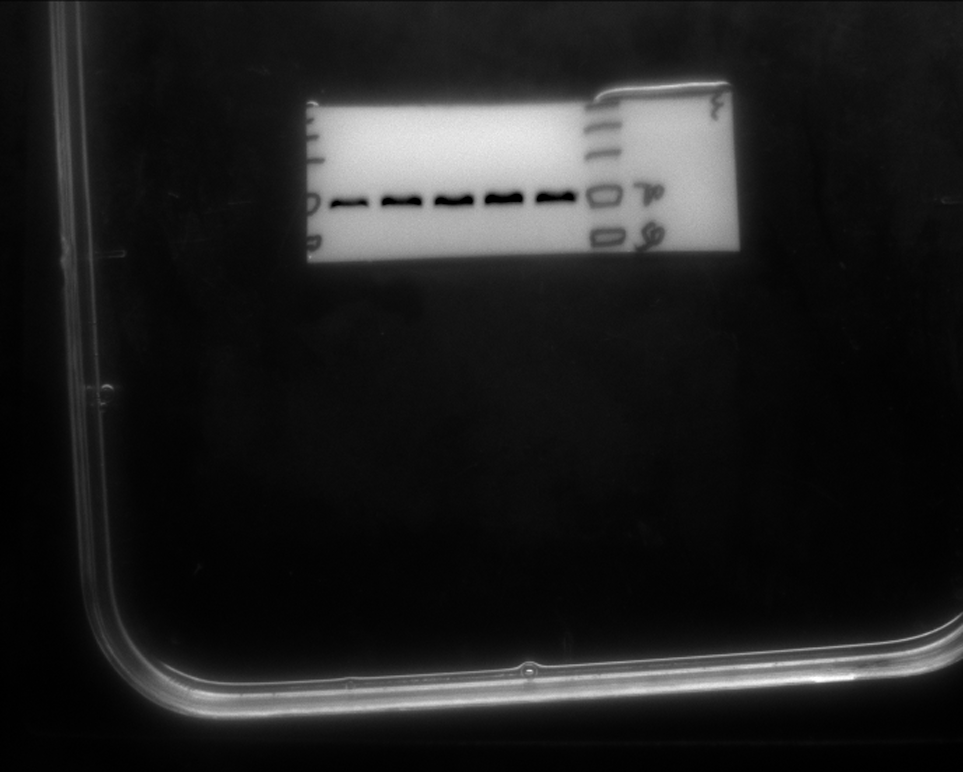

Supplement: Supplementary file 3 [file DataSheet11.ZIP › 1/p65 q.tif]

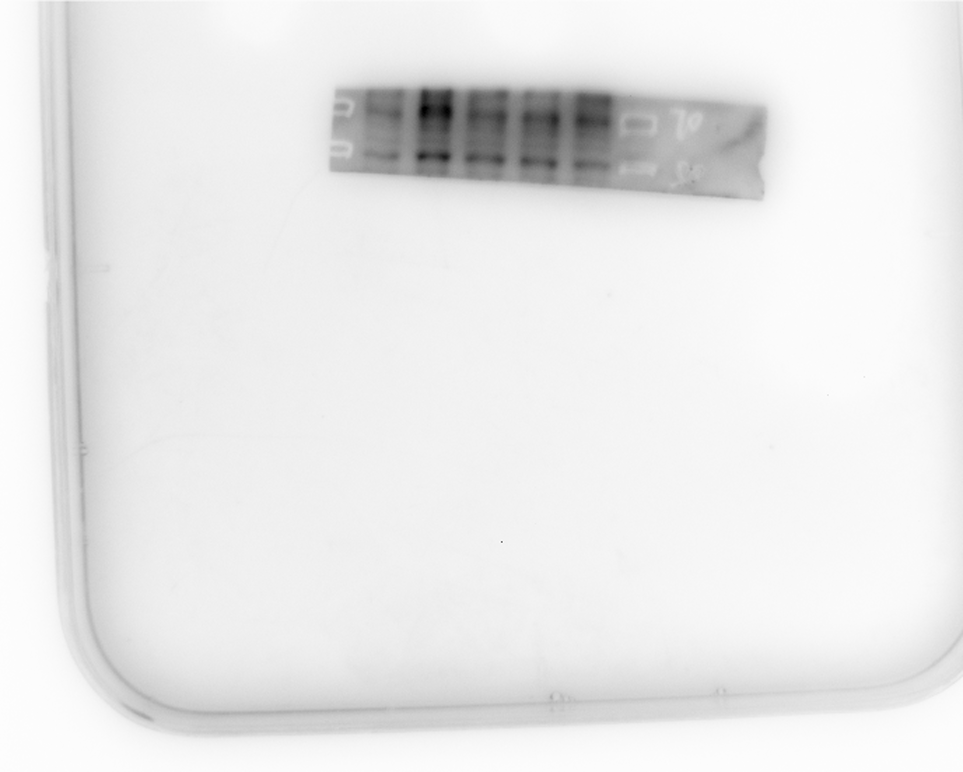

Supplement: Supplementary file 3 [file DataSheet11.ZIP › 2/p-p65 3.tif]

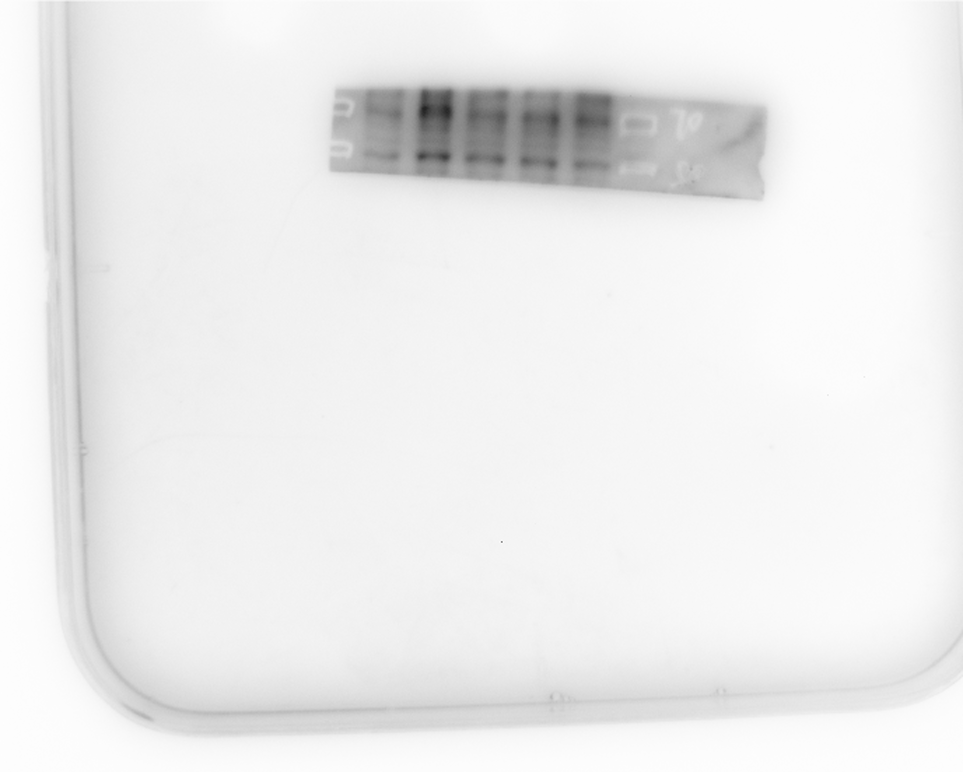

Supplement: Supplementary file 3 [file DataSheet11.ZIP › 2/p-p65 4.tif]

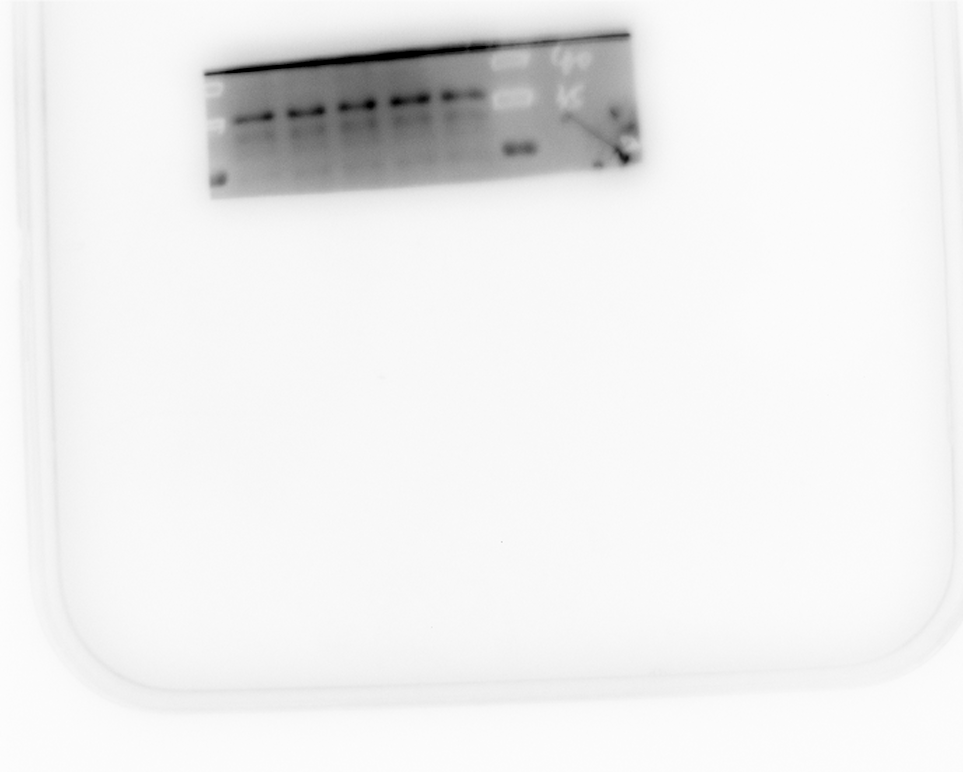

Supplement: Supplementary file 3 [file DataSheet11.ZIP › 2/p-p65 GAPDH 1.tif]

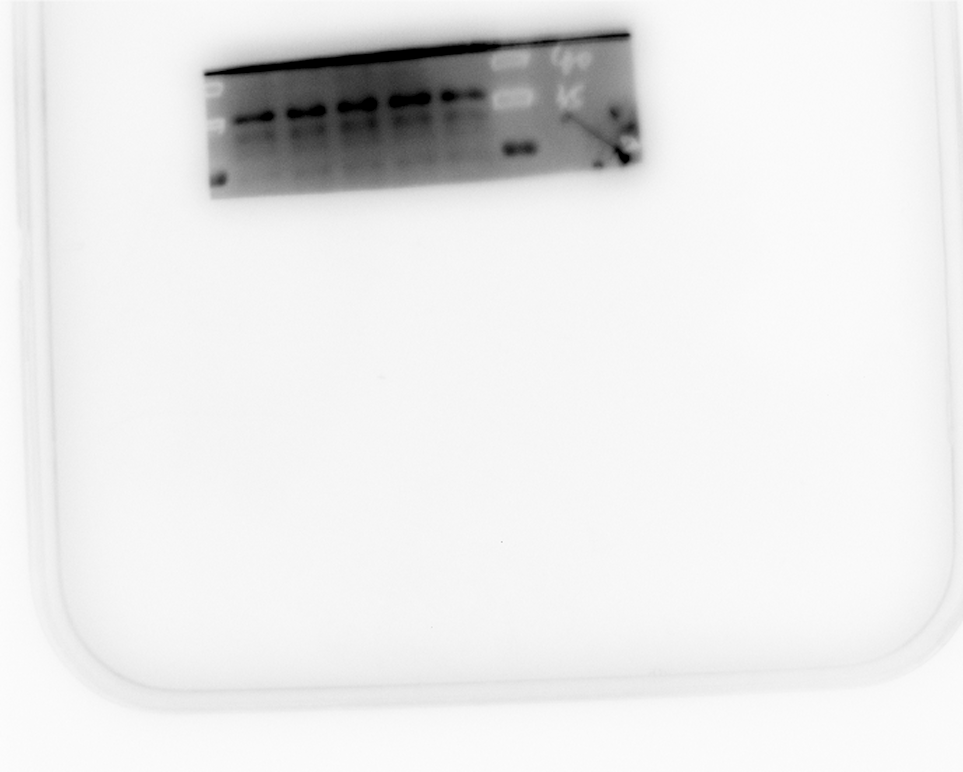

Supplement: Supplementary file 3 [file DataSheet11.ZIP › 2/p-p65 GAPDH 2.tif]

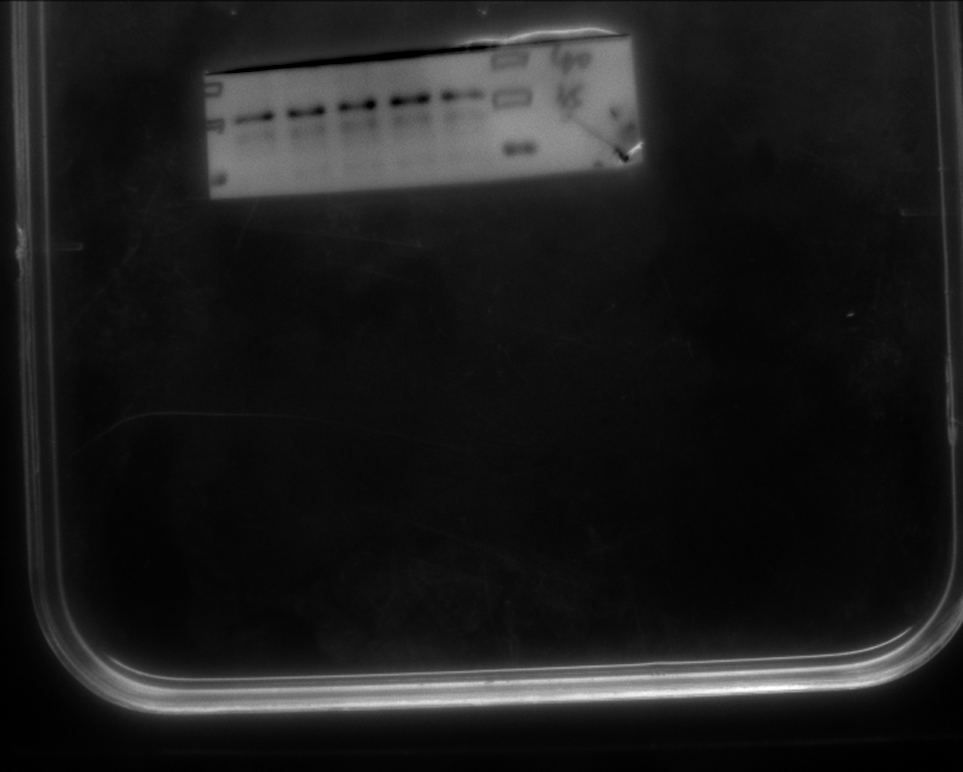

Supplement: Supplementary file 3 [file DataSheet11.ZIP › 2/p-p65 GAPDH q.tif]

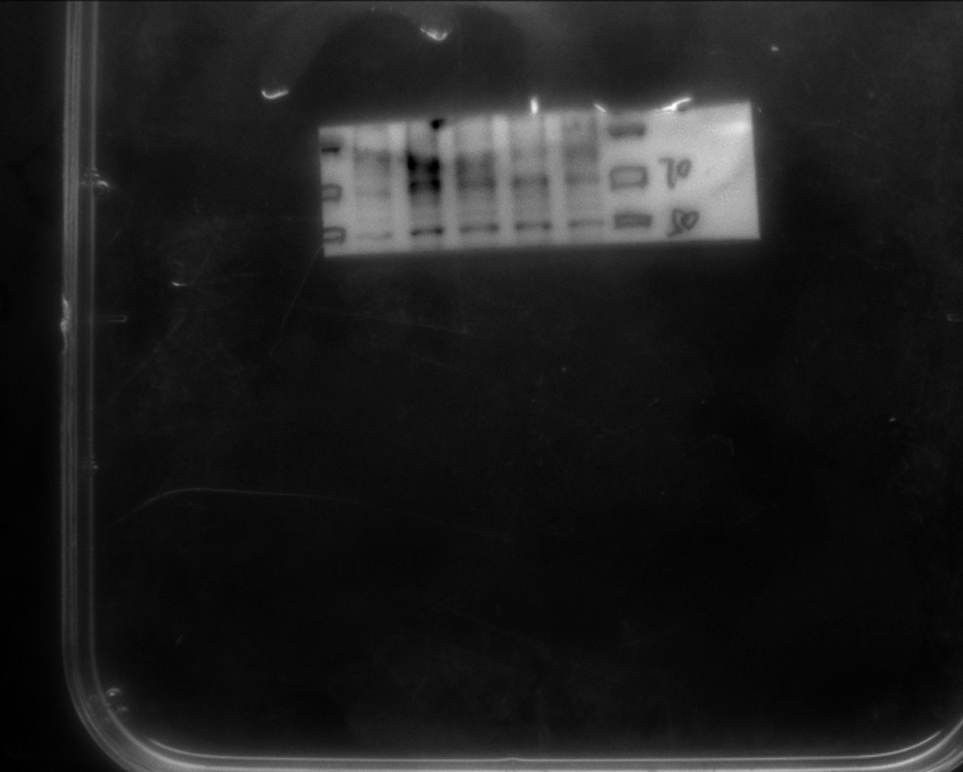

Supplement: Supplementary file 3 [file DataSheet11.ZIP › 2/p-p65 q.tif]

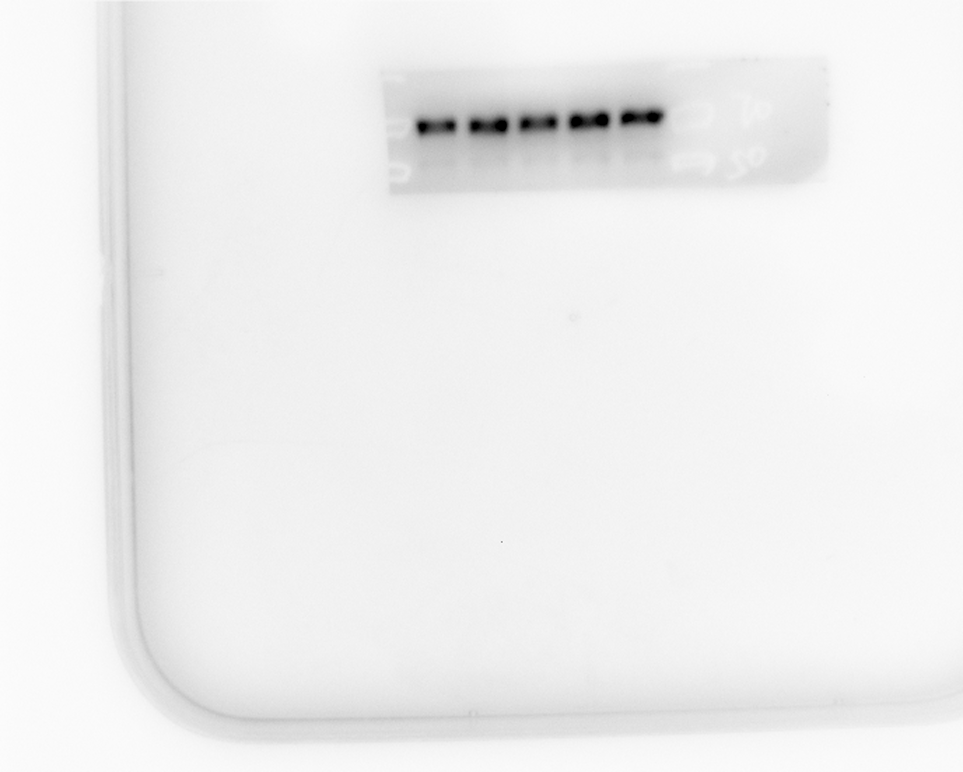

Supplement: Supplementary file 3 [file DataSheet11.ZIP › 2/p65 1.tif]

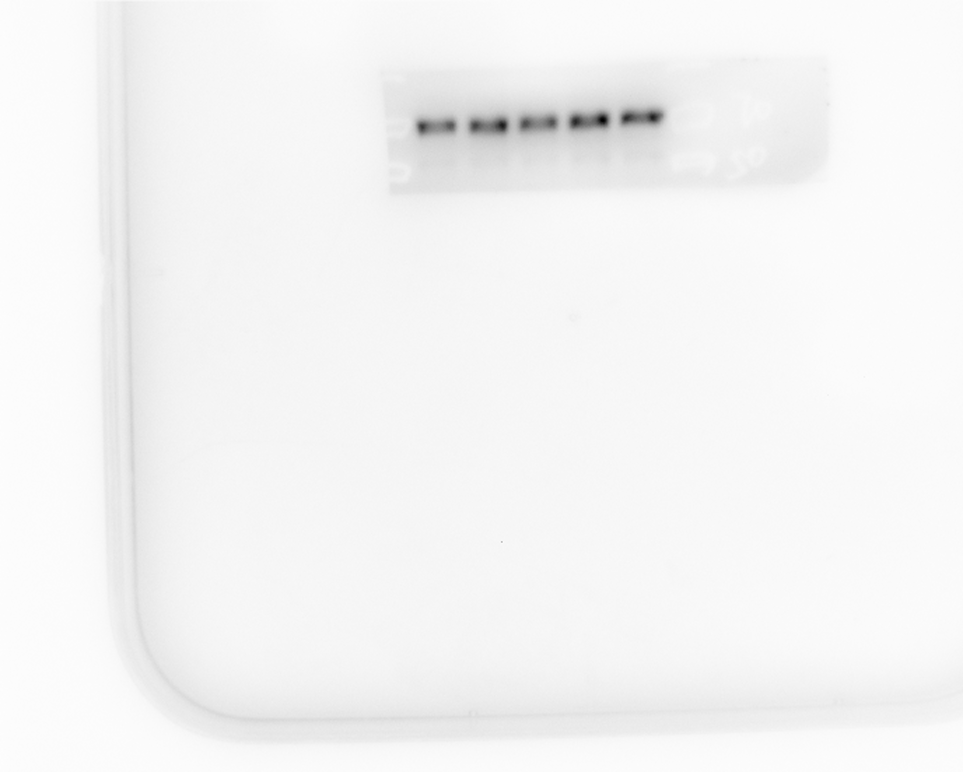

Supplement: Supplementary file 3 [file DataSheet11.ZIP › 2/p65 2.tif]

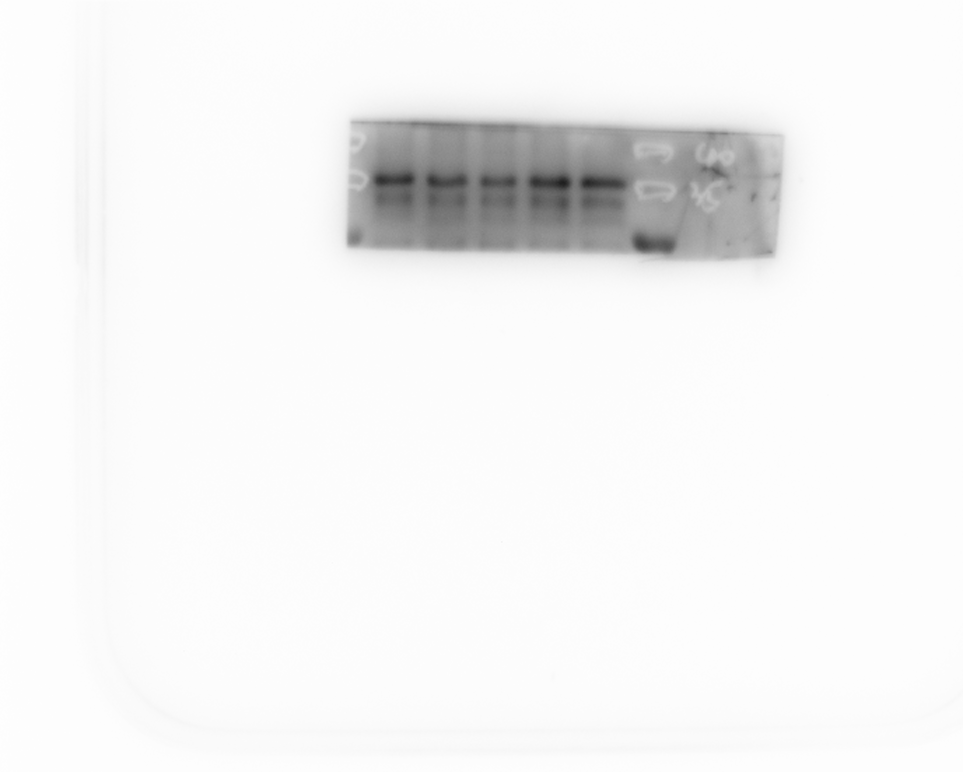

Supplement: Supplementary file 3 [file DataSheet11.ZIP › 2/p65 GAPDH 1.tif]

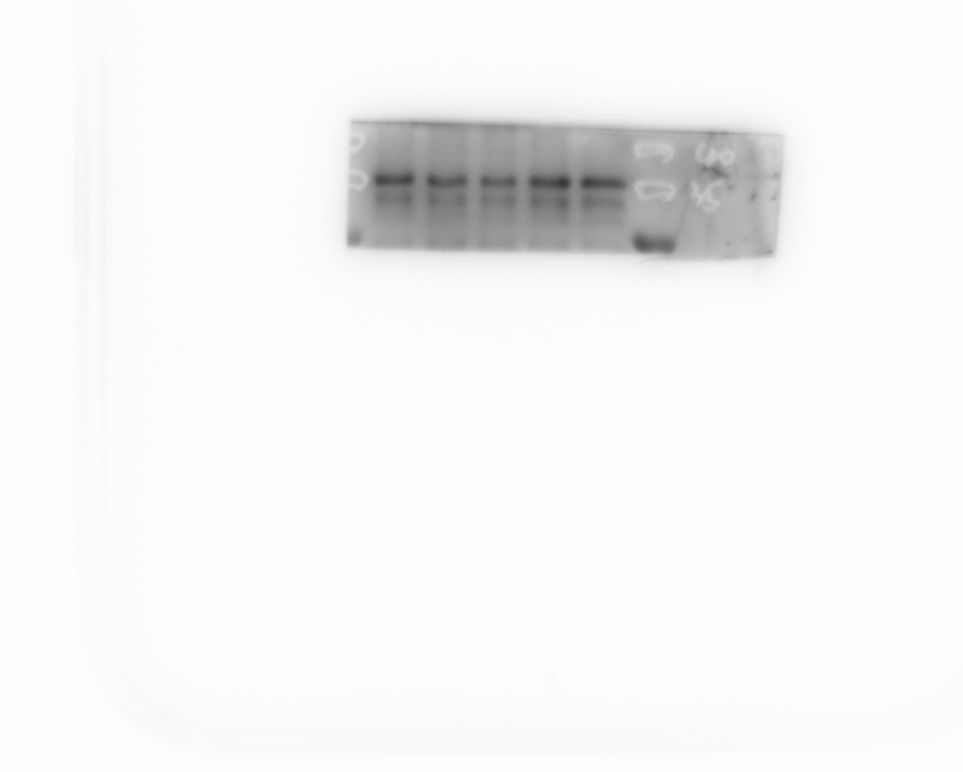

Supplement: Supplementary file 3 [file DataSheet11.ZIP › 2/p65 GAPDH 2.tif]

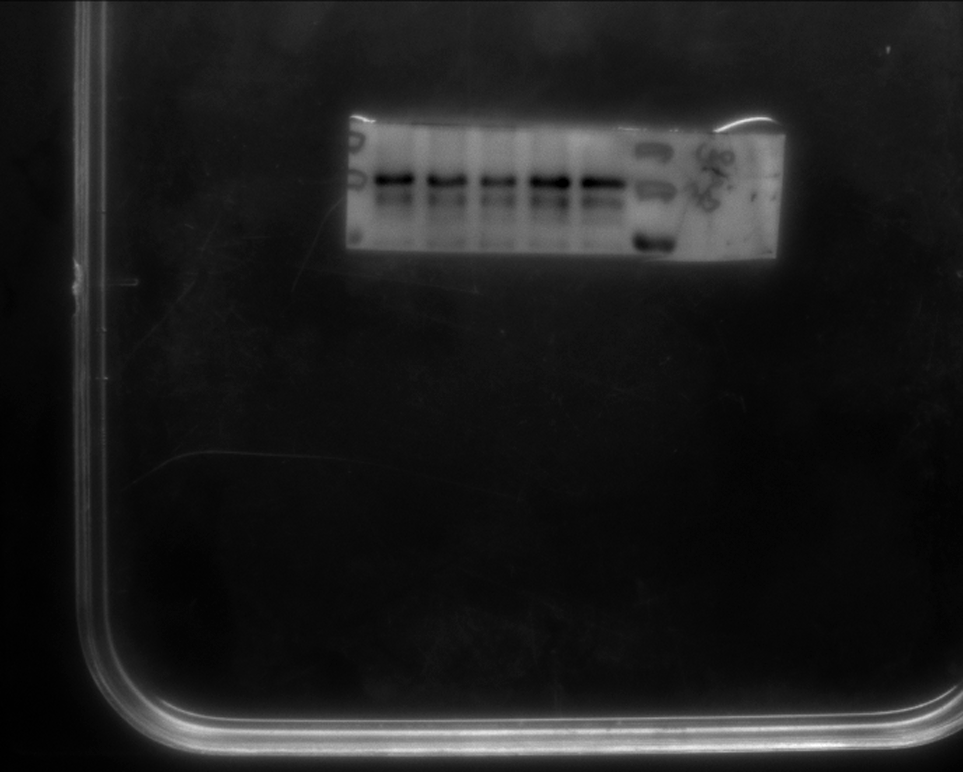

Supplement: Supplementary file 3 [file DataSheet11.ZIP › 2/p65 GAPDH q.tif]

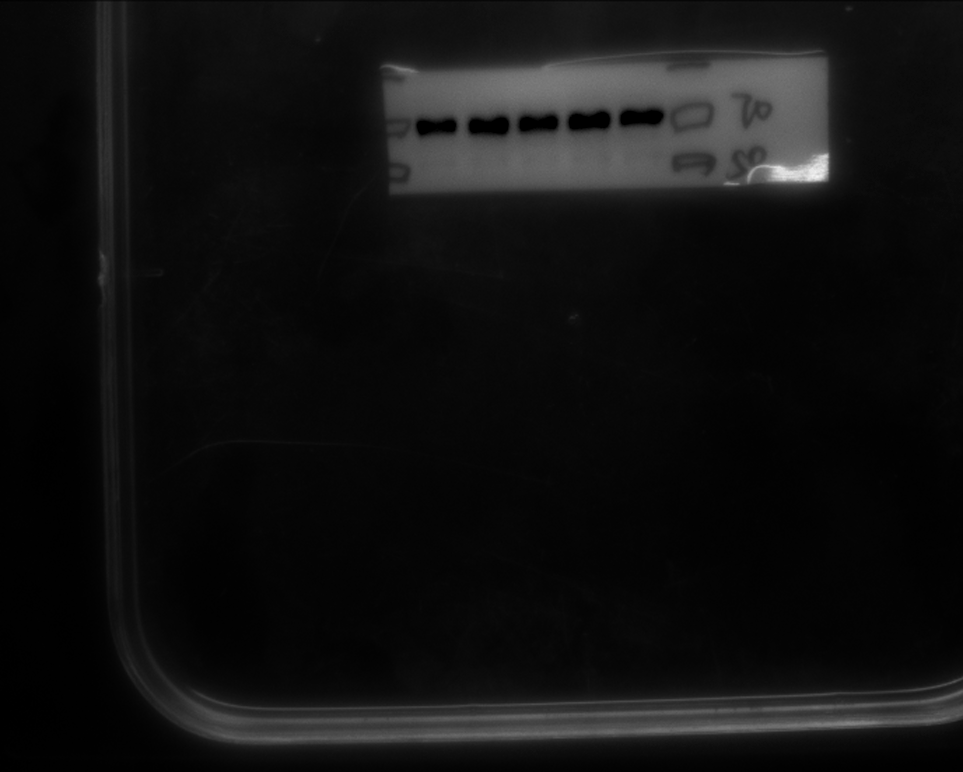

Supplement: Supplementary file 3 [file DataSheet11.ZIP › 2/p65 q.tif]

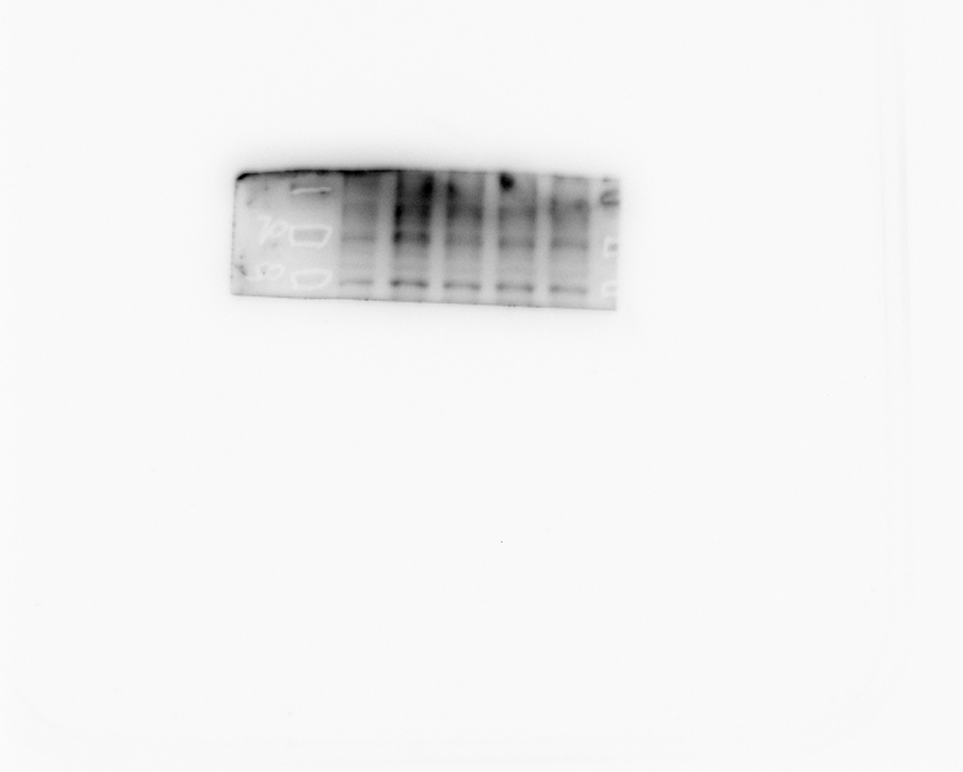

Supplement: Supplementary file 3 [file DataSheet11.ZIP › 3/p-p65 1.tif]

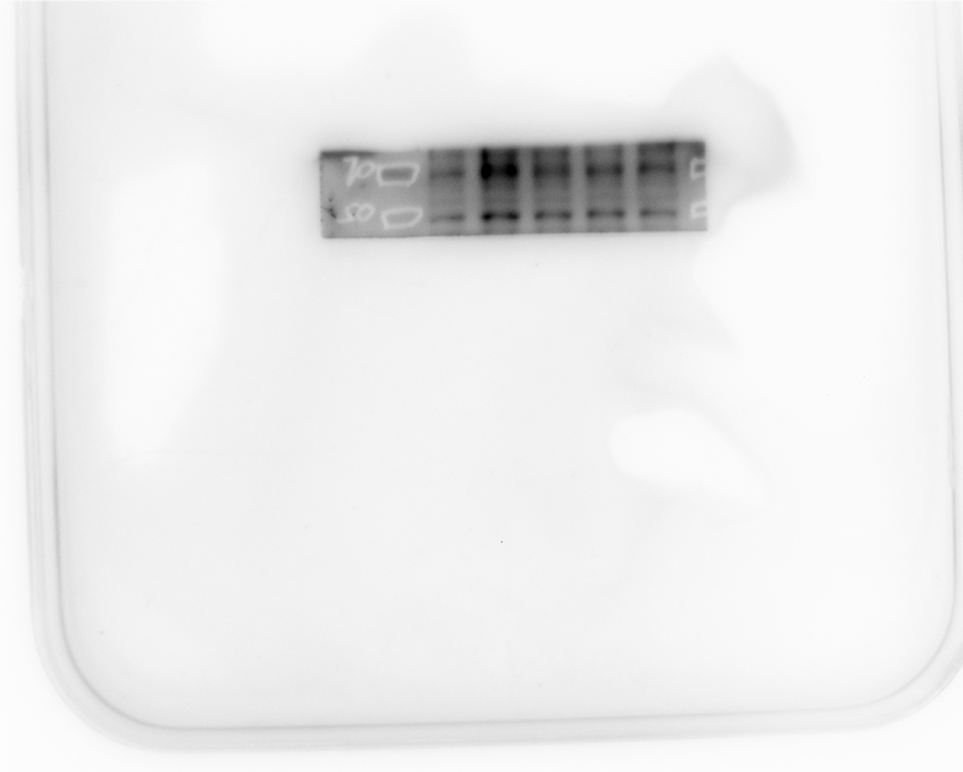

Supplement: Supplementary file 3 [file DataSheet11.ZIP › 3/p-p65 2.tif]

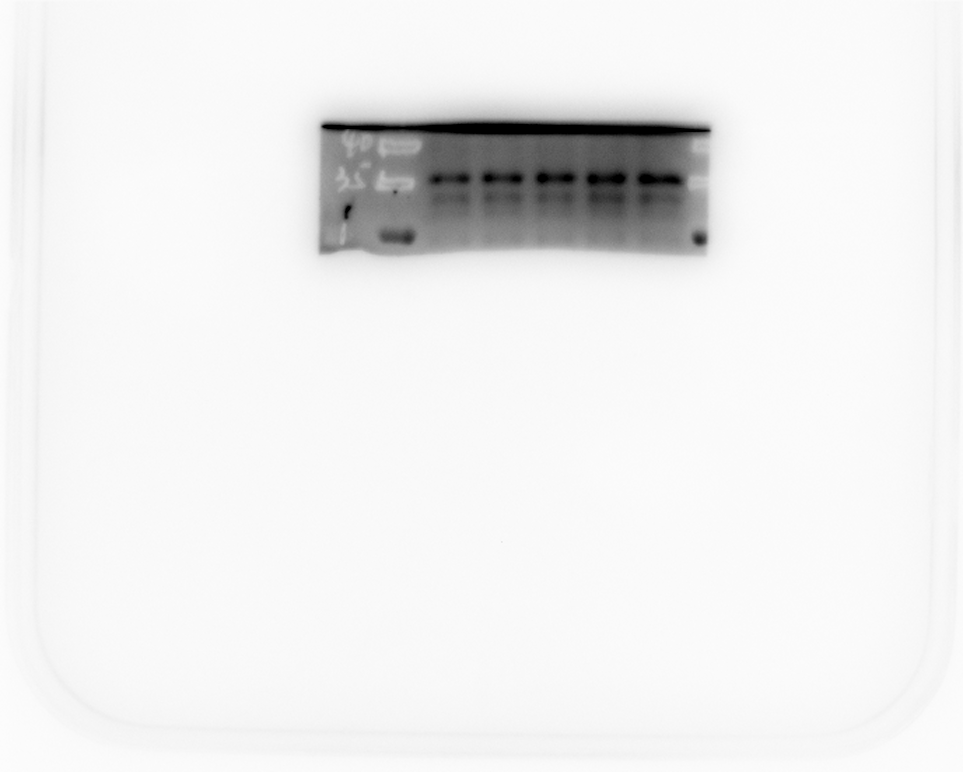

Supplement: Supplementary file 3 [file DataSheet11.ZIP › 3/p-p65 GAPDH 1.tif]

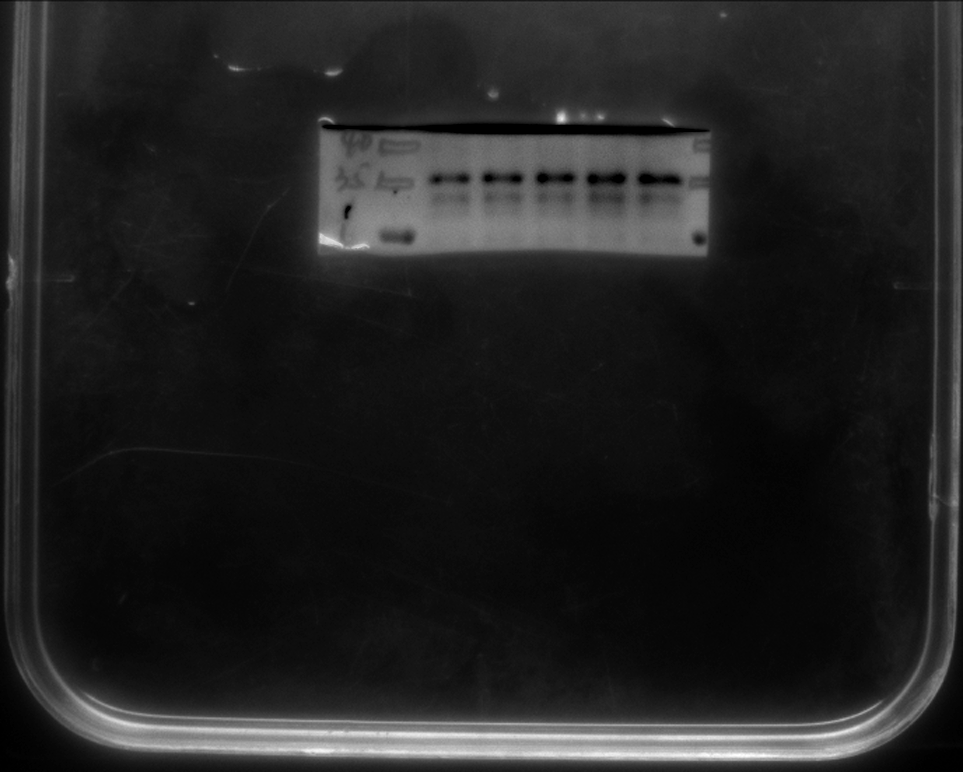

Supplement: Supplementary file 3 [file DataSheet11.ZIP › 3/p-p65 GAPDH q.tif]

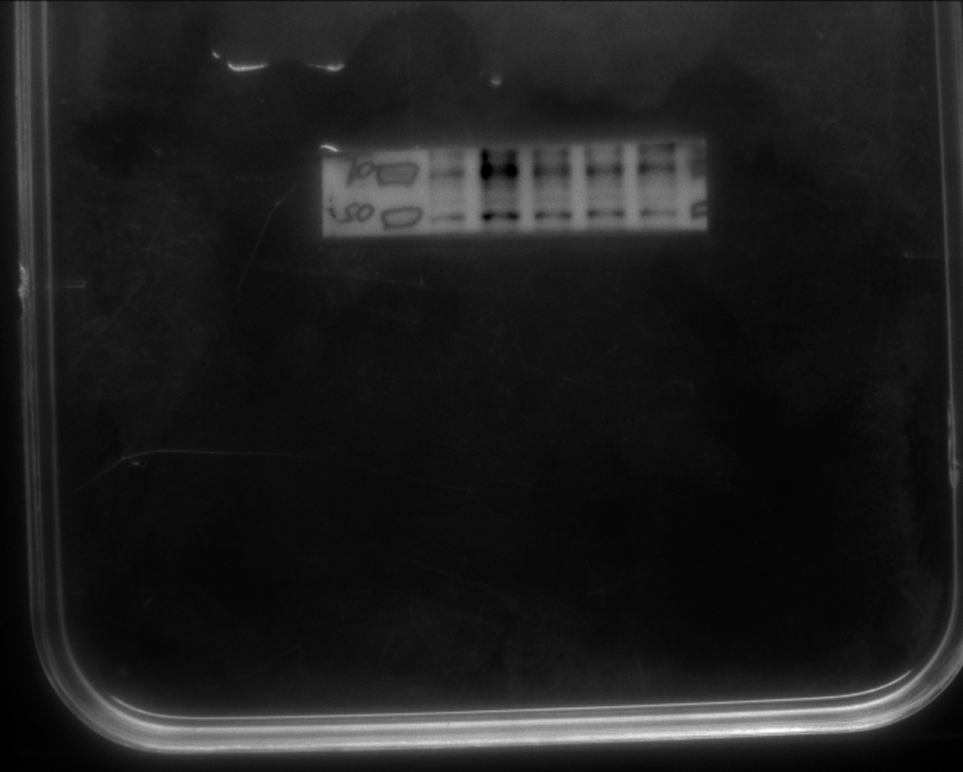

Supplement: Supplementary file 3 [file DataSheet11.ZIP › 3/p-p65 q 1.tif]

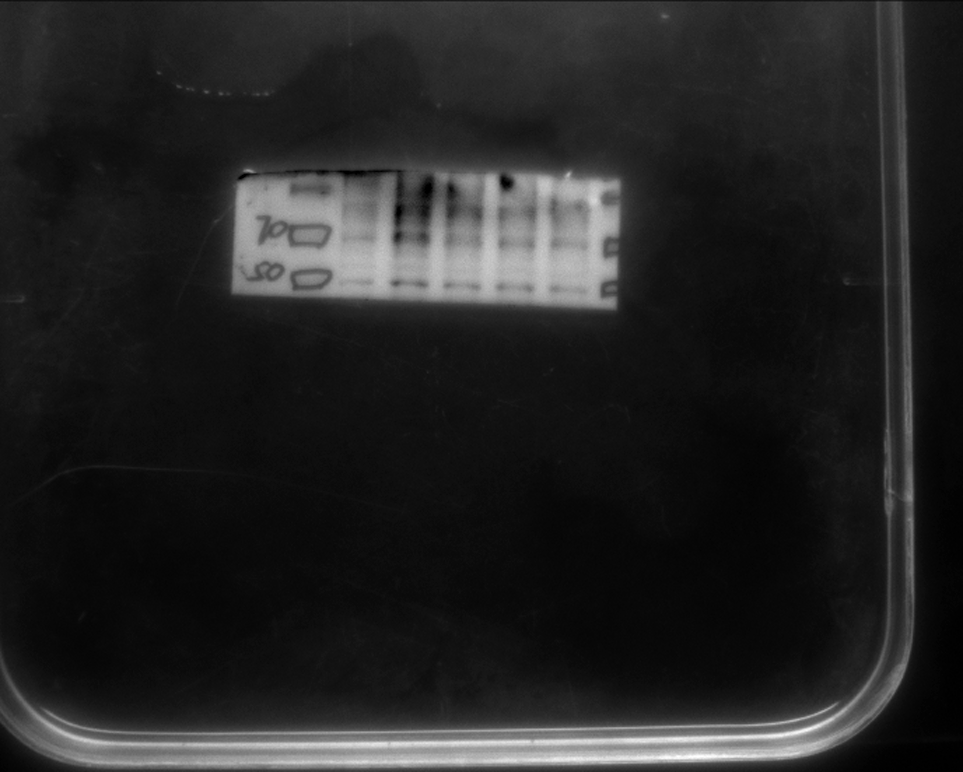

Supplement: Supplementary file 3 [file DataSheet11.ZIP › 3/p-p65 q.tif]

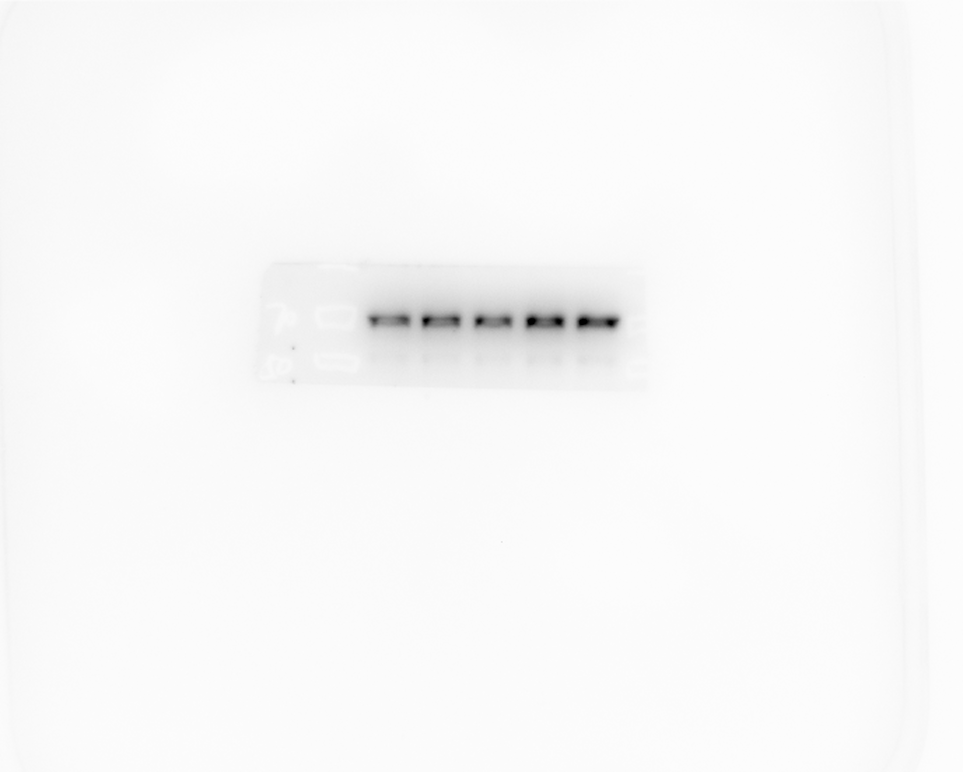

Supplement: Supplementary file 3 [file DataSheet11.ZIP › 3/p65 1.tif]

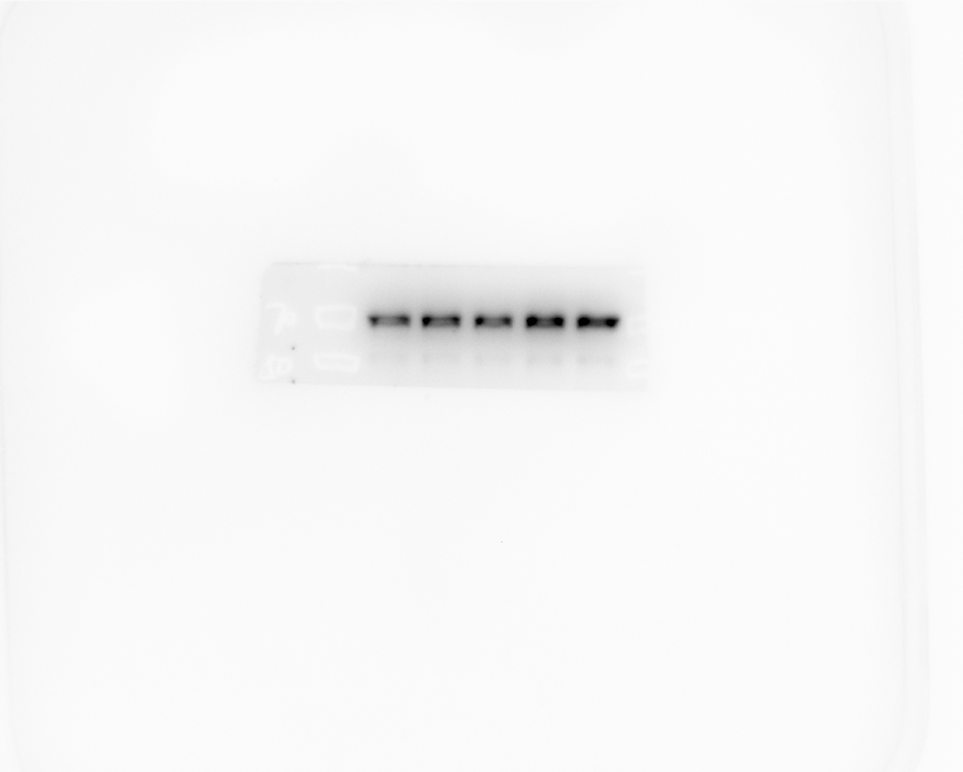

Supplement: Supplementary file 3 [file DataSheet11.ZIP › 3/p65 2.tif]

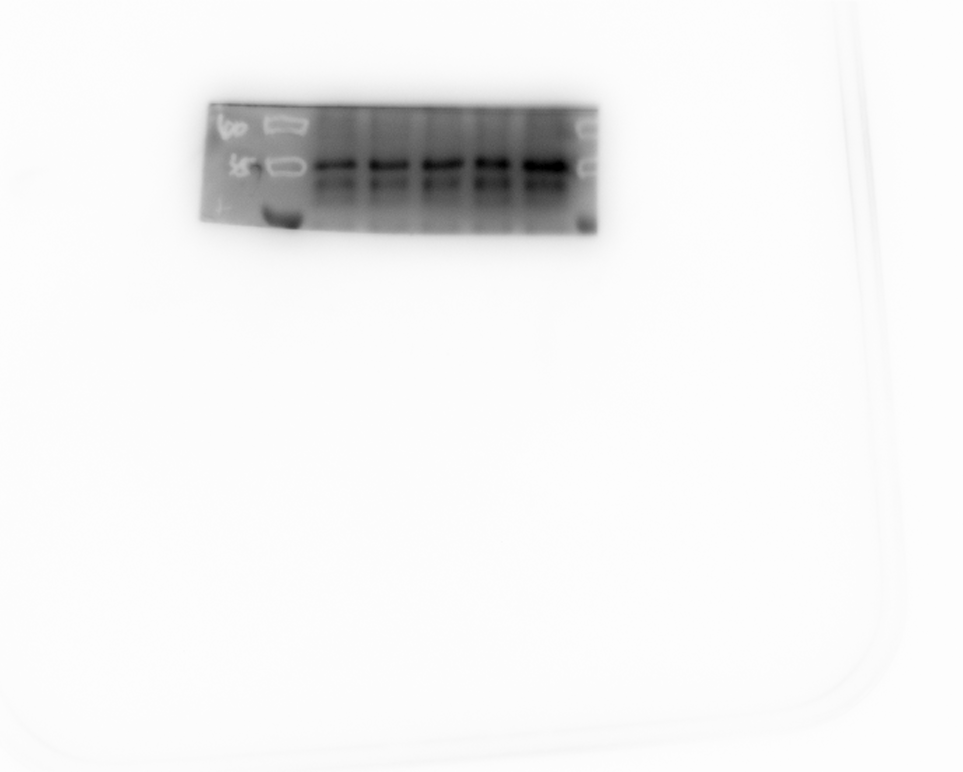

Supplement: Supplementary file 3 [file DataSheet11.ZIP › 3/p65 GAPDH 1.tif]

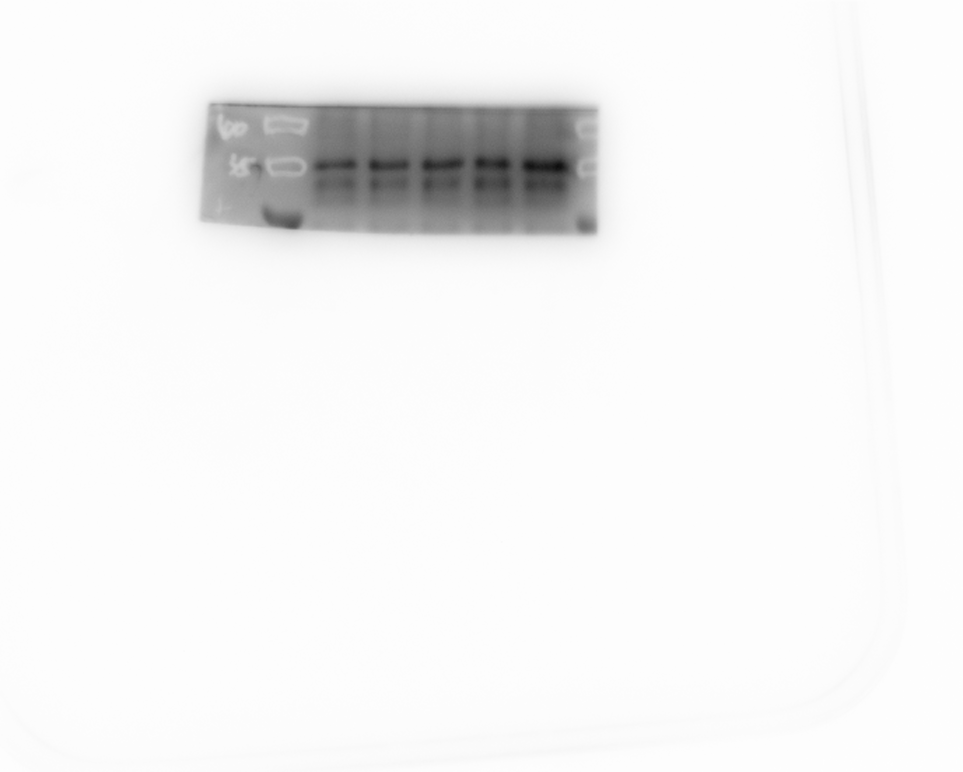

Supplement: Supplementary file 3 [file DataSheet11.ZIP › 3/p65 GAPDH 2.tif]

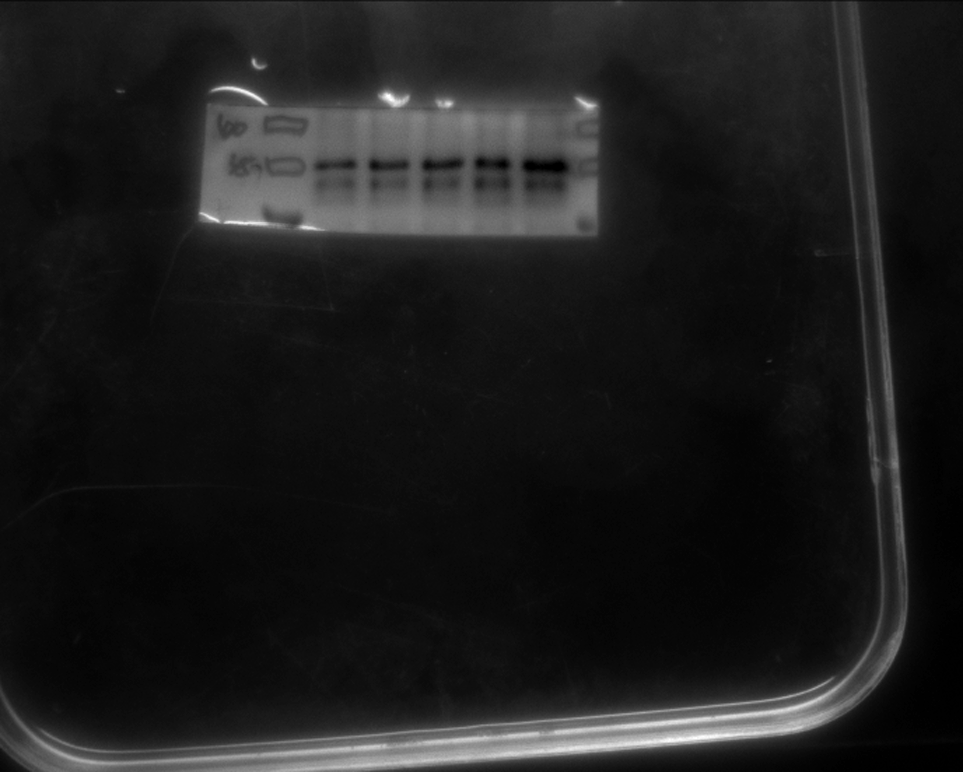

Supplement: Supplementary file 3 [file DataSheet11.ZIP › 3/p65 GAPDH q.tif]

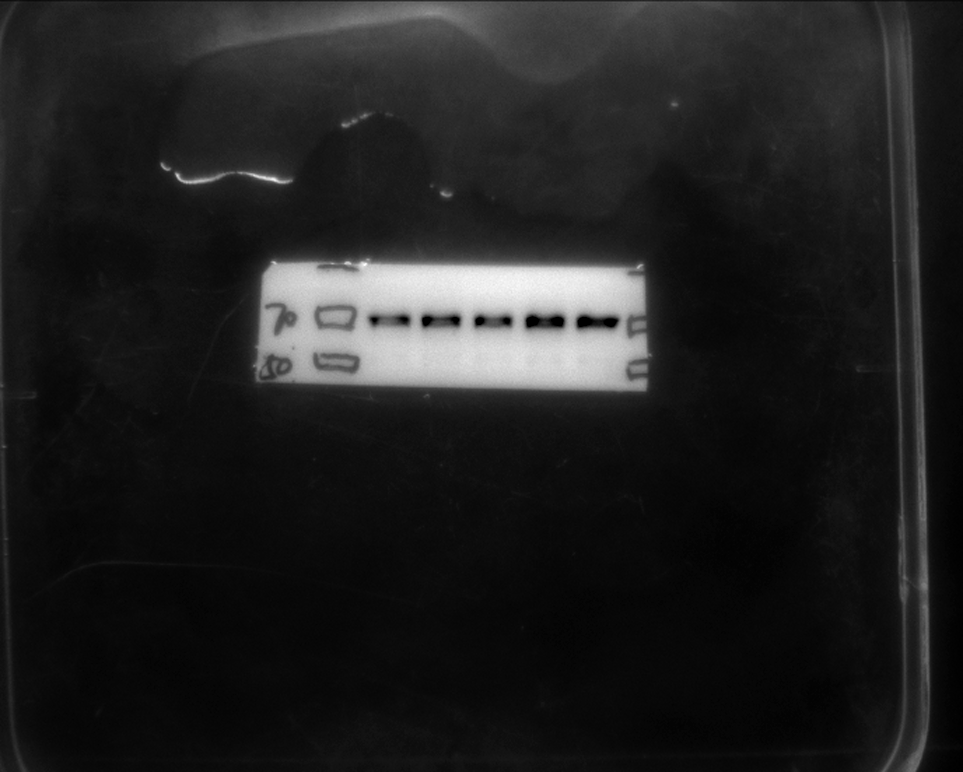

Supplement: Supplementary file 3 [file DataSheet11.ZIP › 3/p65 q.tif]

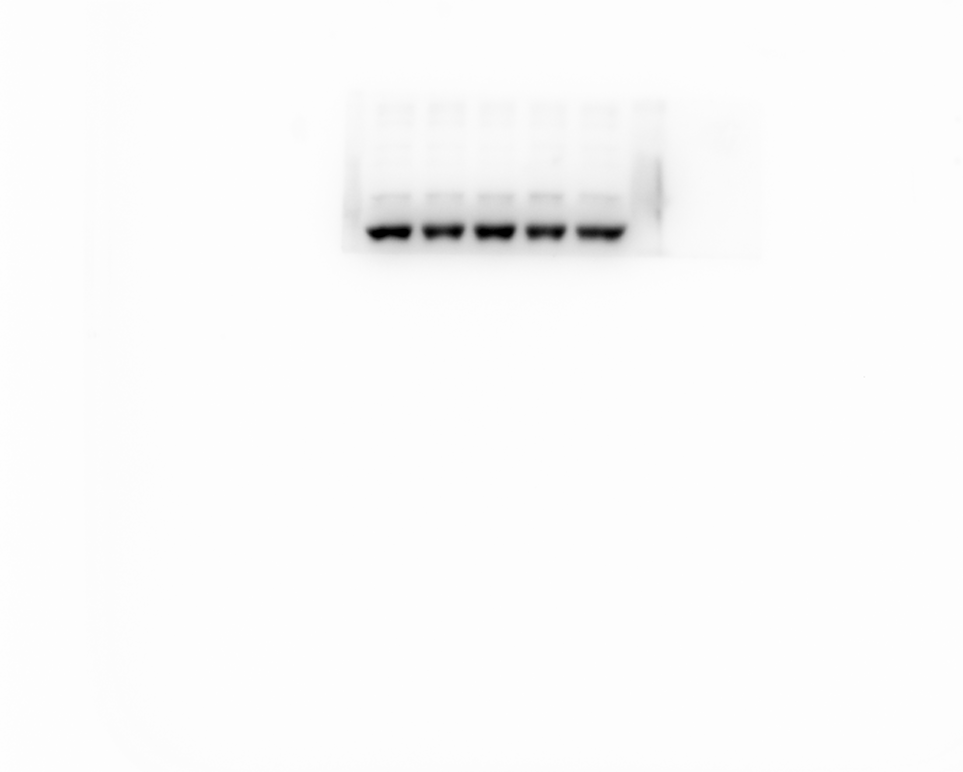

Supplement: Supplementary file 4 [file DataSheet8.ZIP › JNK1/JNK 1.tif]

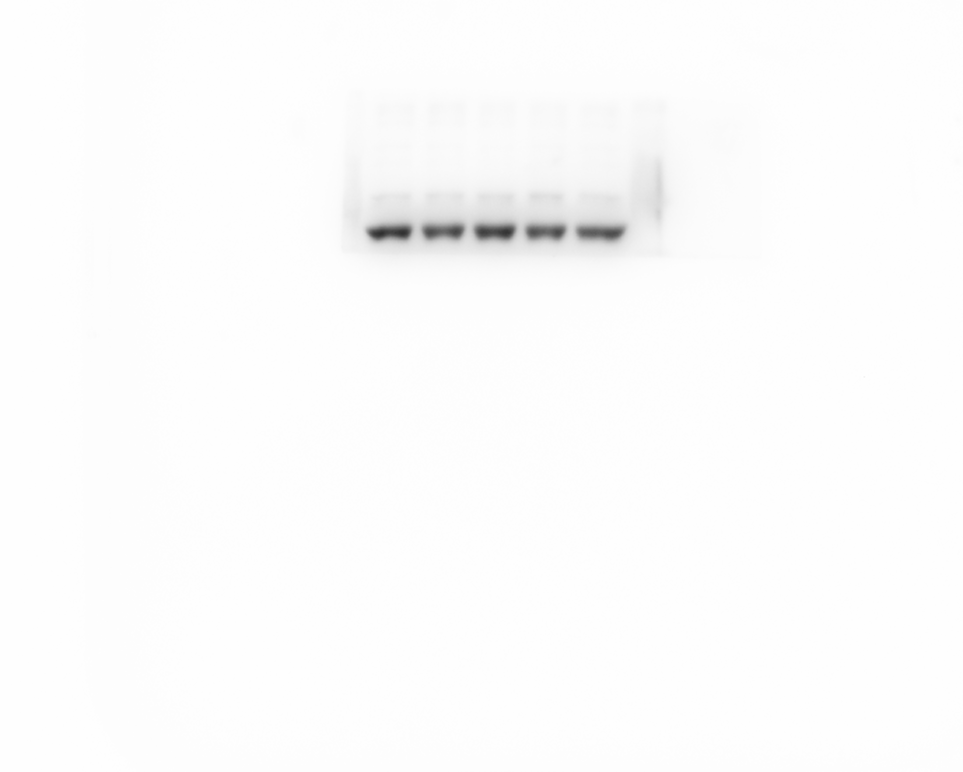

Supplement: Supplementary file 4 [file DataSheet8.ZIP › JNK1/JNK 2.tif]

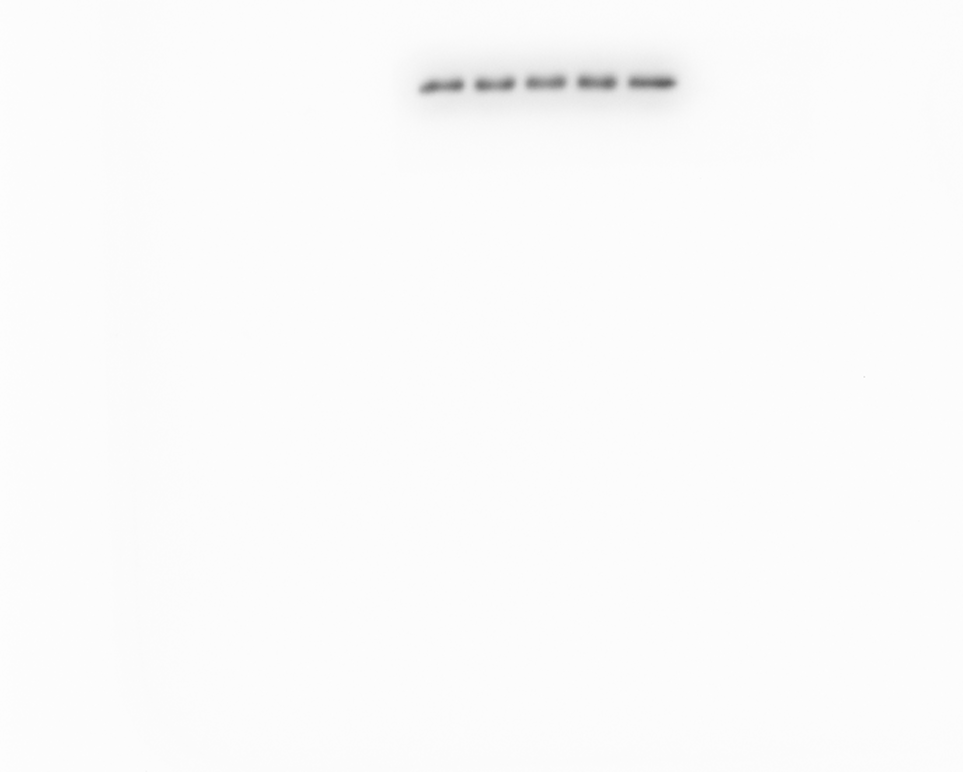

Supplement: Supplementary file 4 [file DataSheet8.ZIP › JNK1/JNK GAPDH 1.tif]

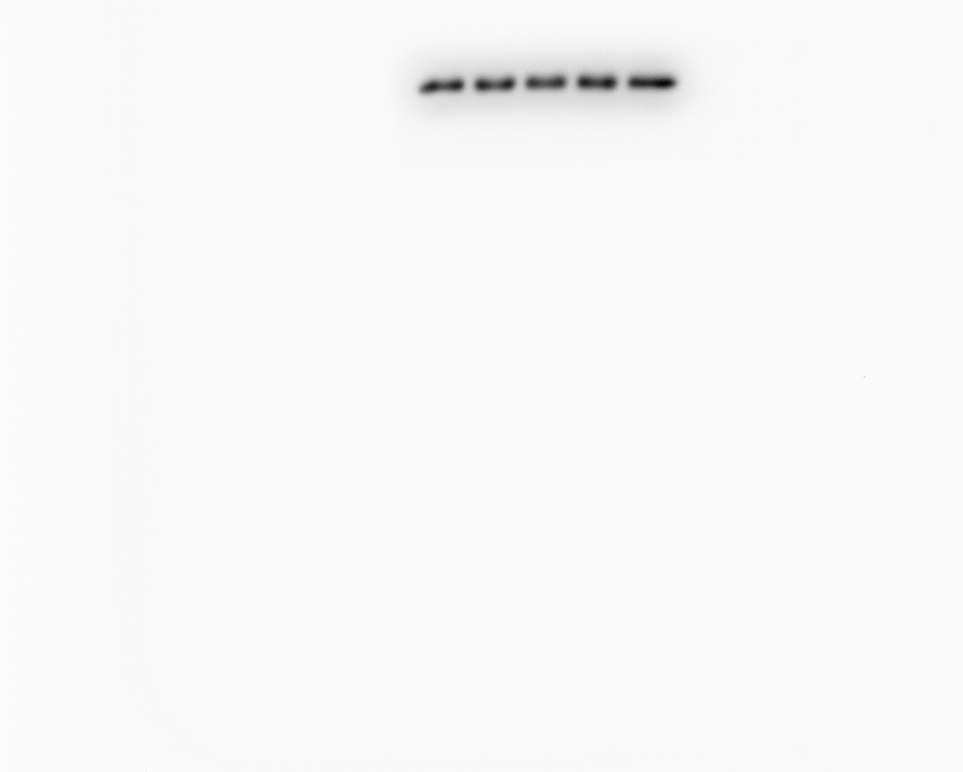

Supplement: Supplementary file 4 [file DataSheet8.ZIP › JNK1/JNK GAPDH 2.tif]

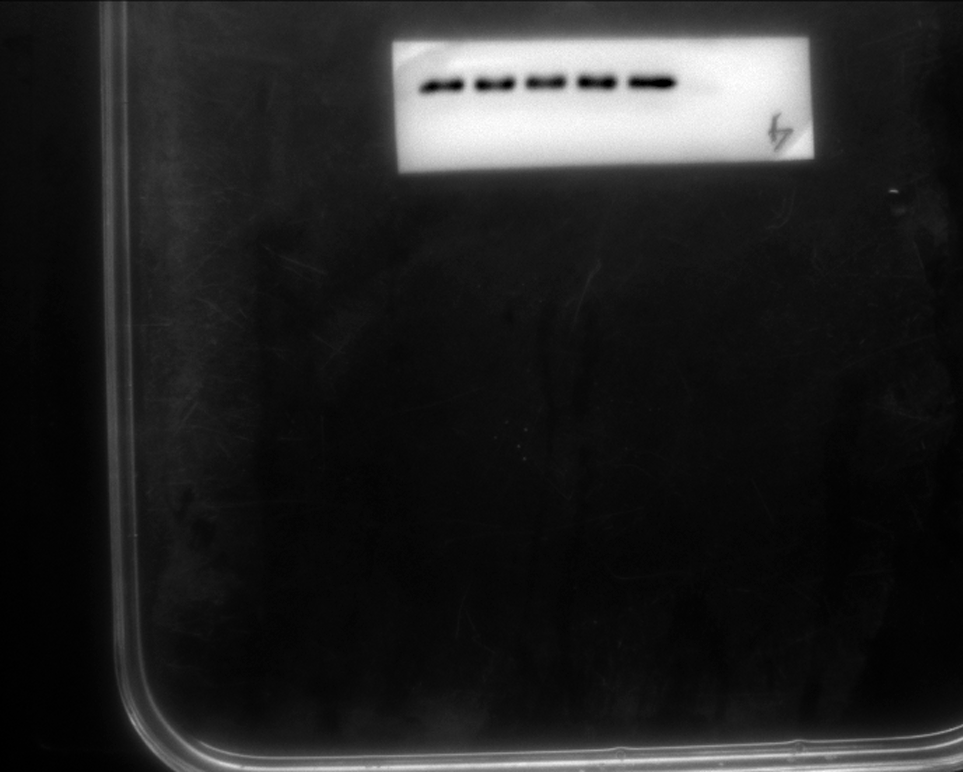

Supplement: Supplementary file 4 [file DataSheet8.ZIP › JNK1/JNK GAPDH q.tif]

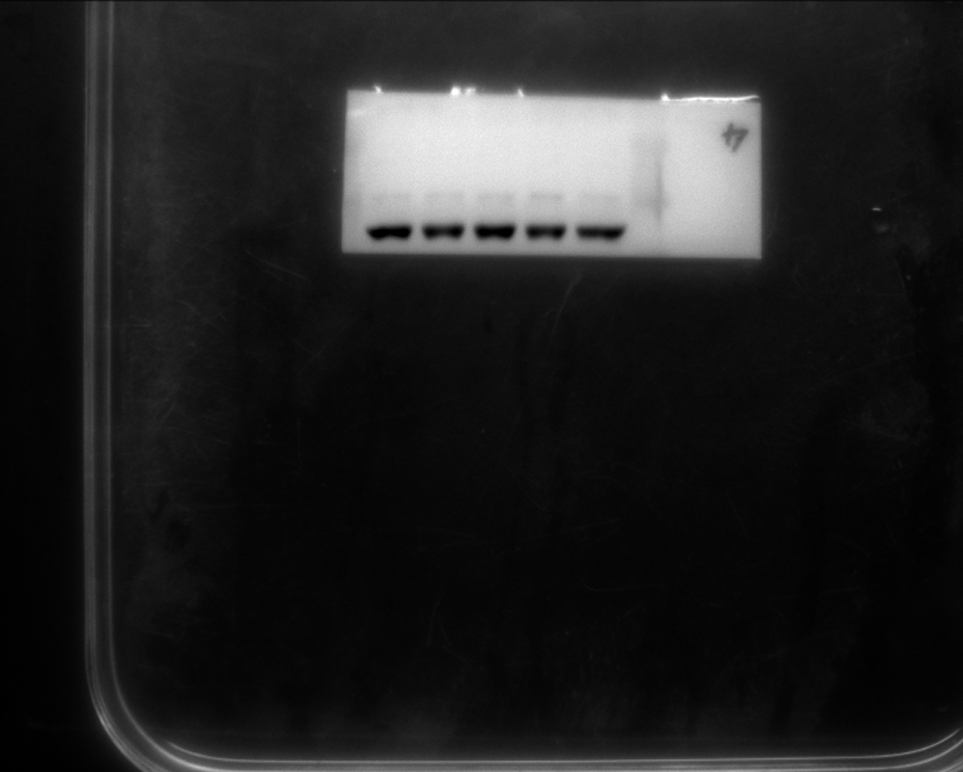

Supplement: Supplementary file 4 [file DataSheet8.ZIP › JNK1/JNK q.tif]

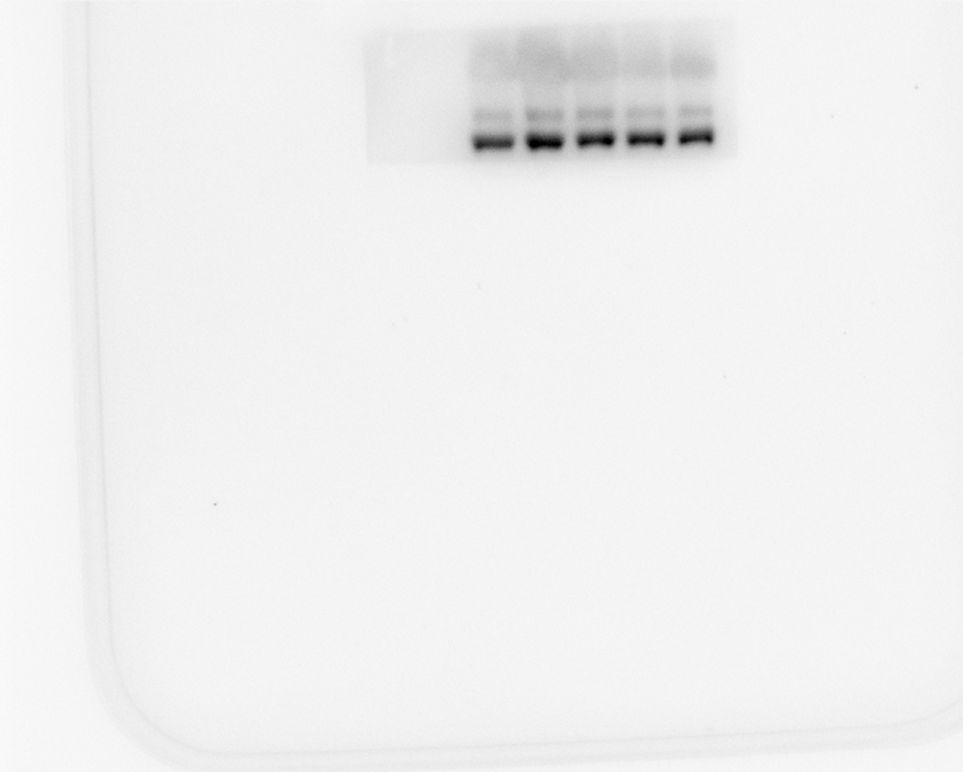

Supplement: Supplementary file 4 [file DataSheet8.ZIP › JNK1/p-JNK 1.tif]

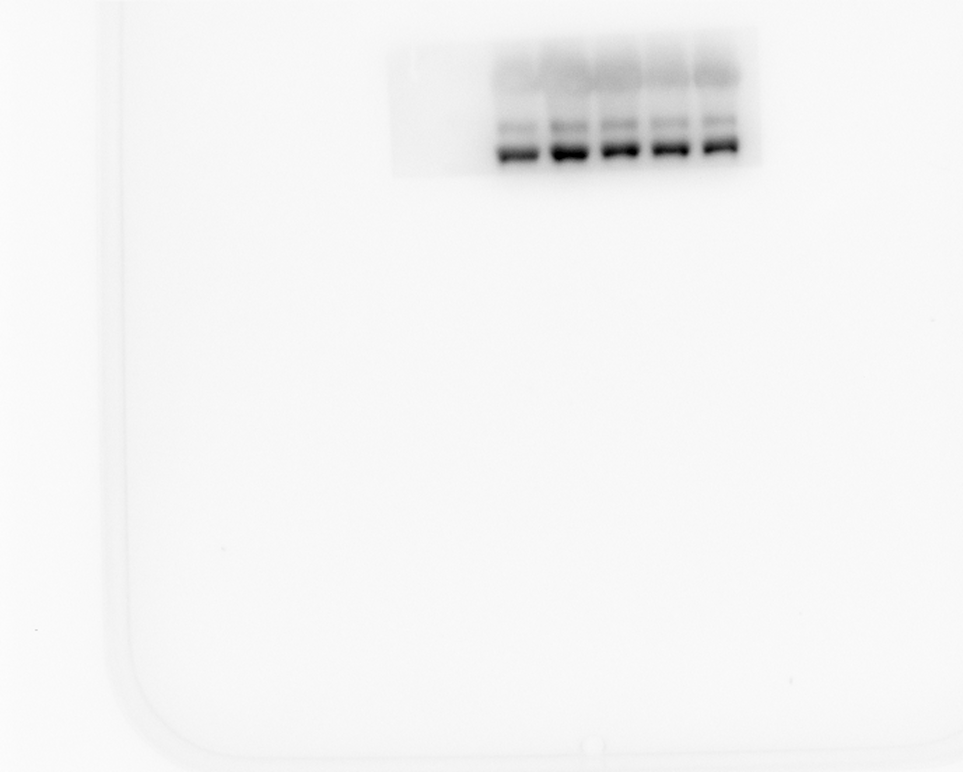

Supplement: Supplementary file 4 [file DataSheet8.ZIP › JNK1/p-JNK 2.tif]

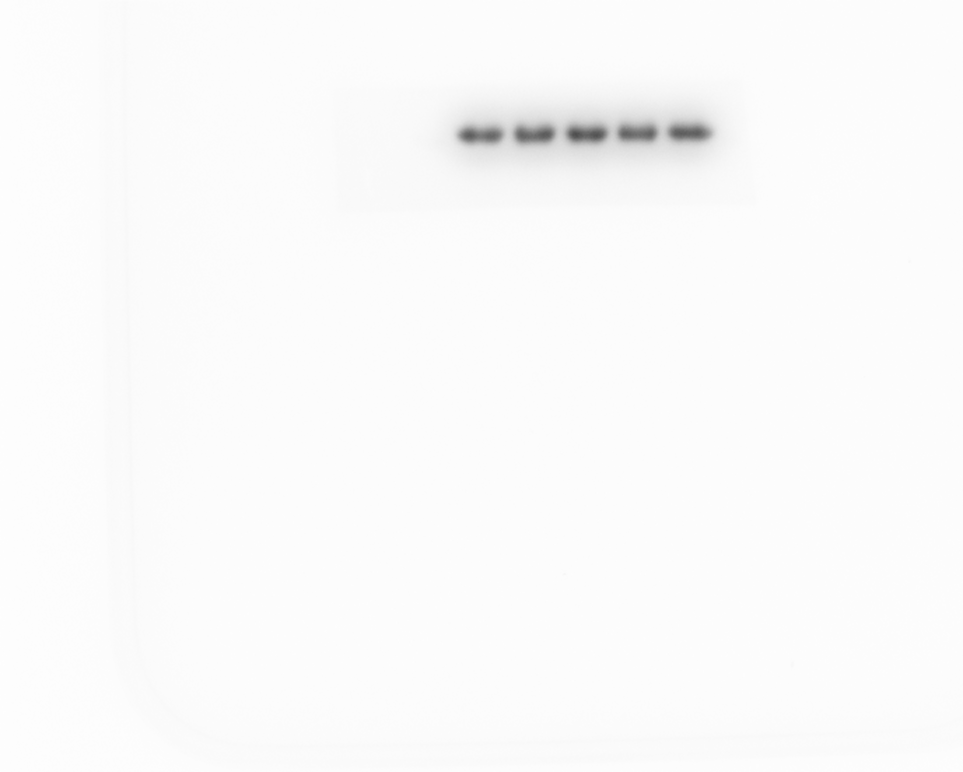

Supplement: Supplementary file 4 [file DataSheet8.ZIP › JNK1/p-JNK GAPDH 1.tif]

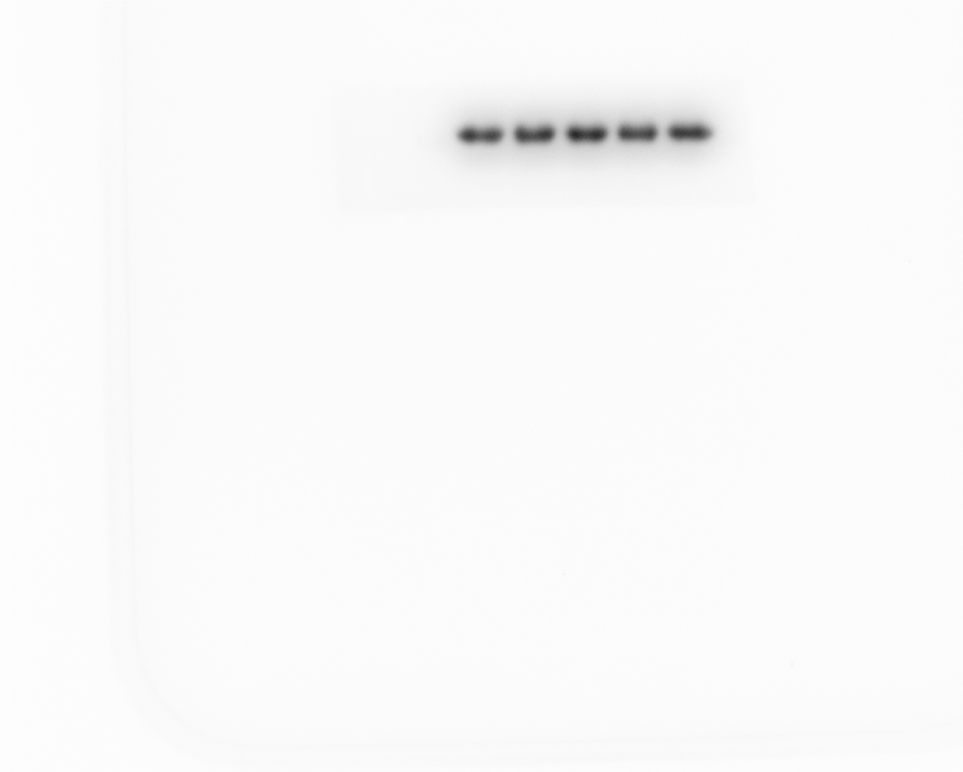

Supplement: Supplementary file 4 [file DataSheet8.ZIP › JNK1/p-JNK GAPDH 2.tif]

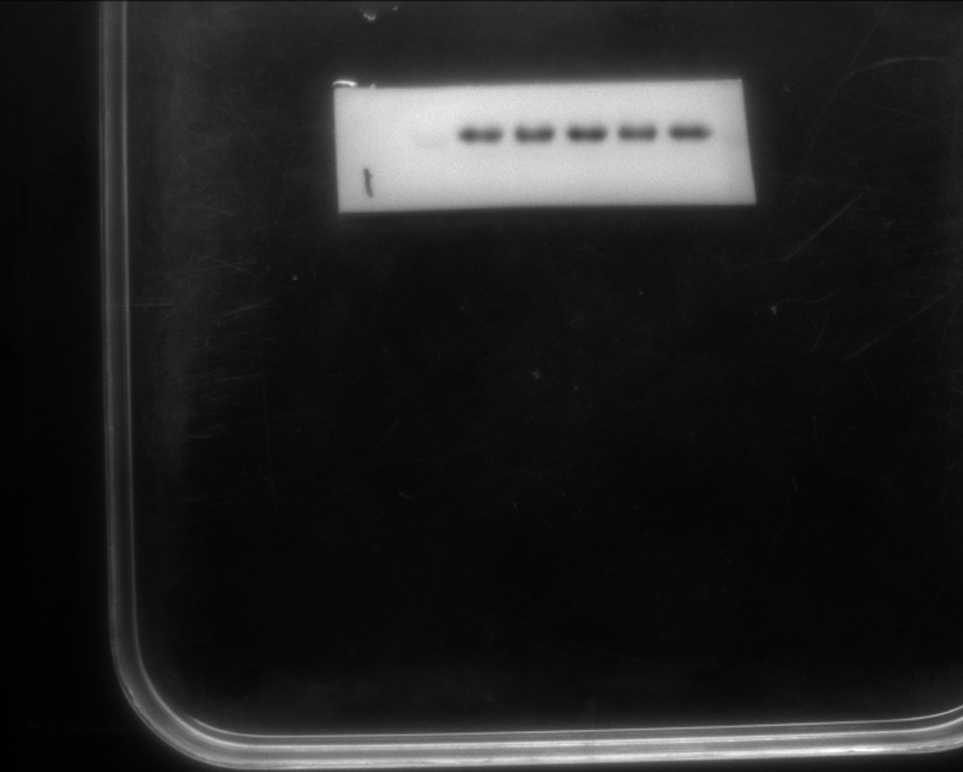

Supplement: Supplementary file 4 [file DataSheet8.ZIP › JNK1/p-JNK GAPDH q.tif]

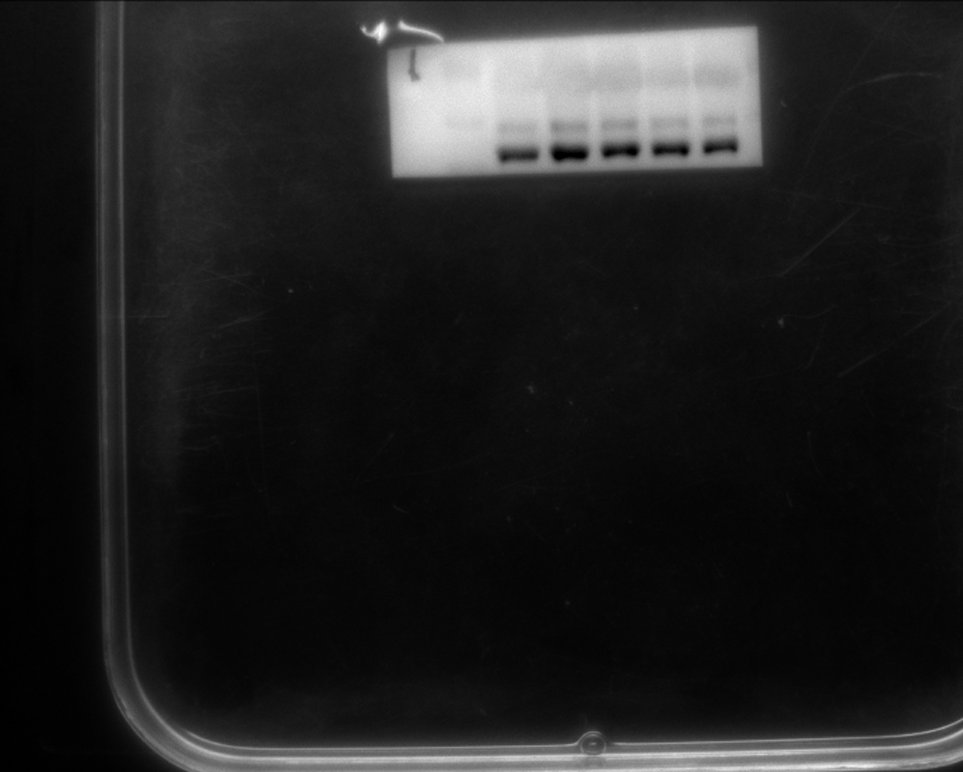

Supplement: Supplementary file 4 [file DataSheet8.ZIP › JNK1/p-JNK q.tif]

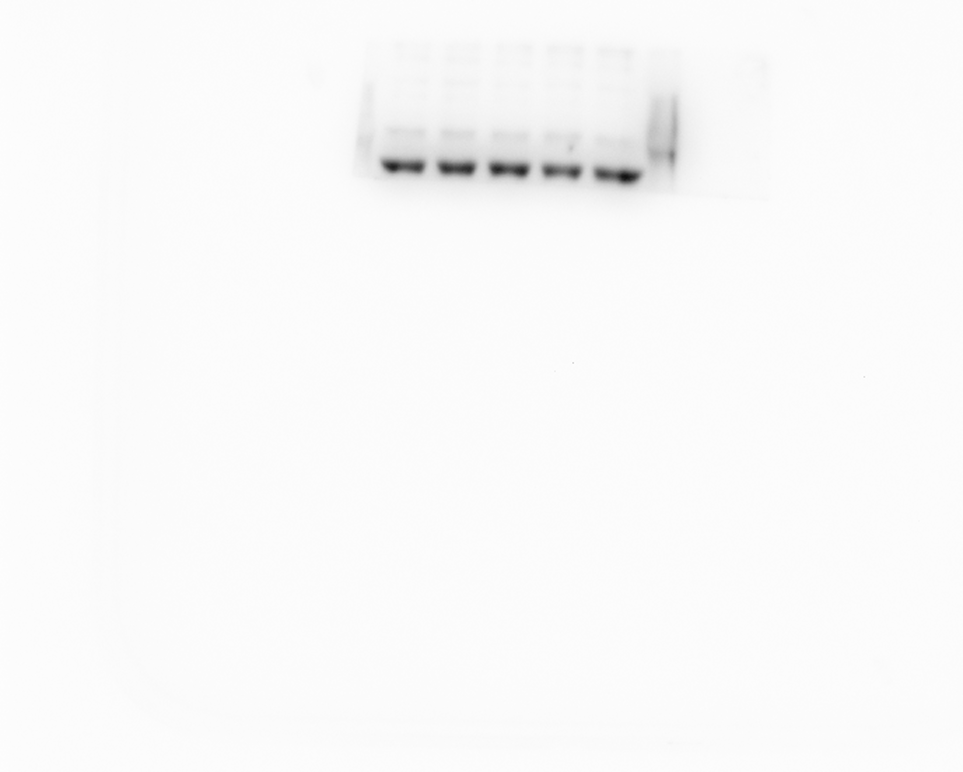

Supplement: Supplementary file 4 [file DataSheet8.ZIP › JNK2/JNK 1.tif]

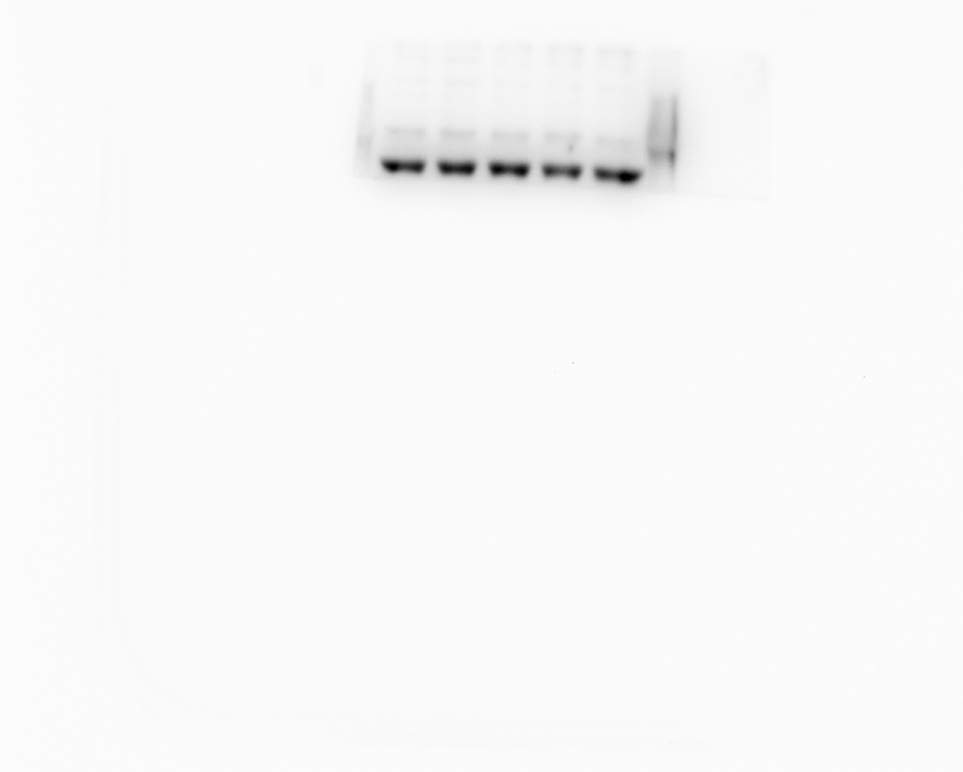

Supplement: Supplementary file 4 [file DataSheet8.ZIP › JNK2/JNK 2.tif]

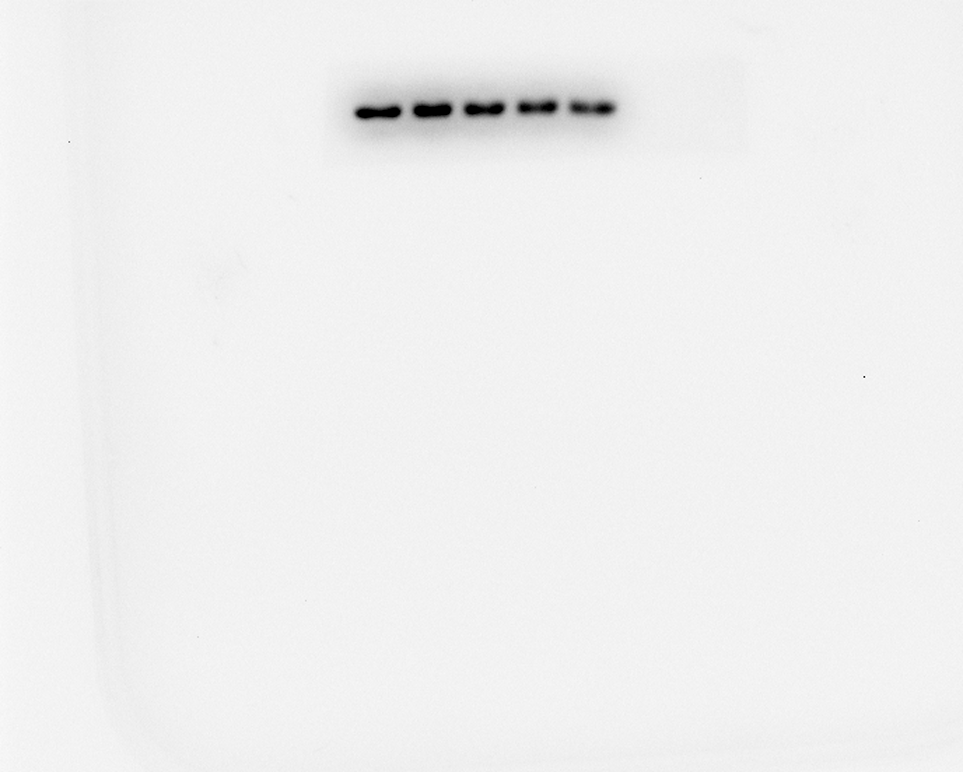

Supplement: Supplementary file 4 [file DataSheet8.ZIP › JNK2/JNK GAPDH 3.tif]

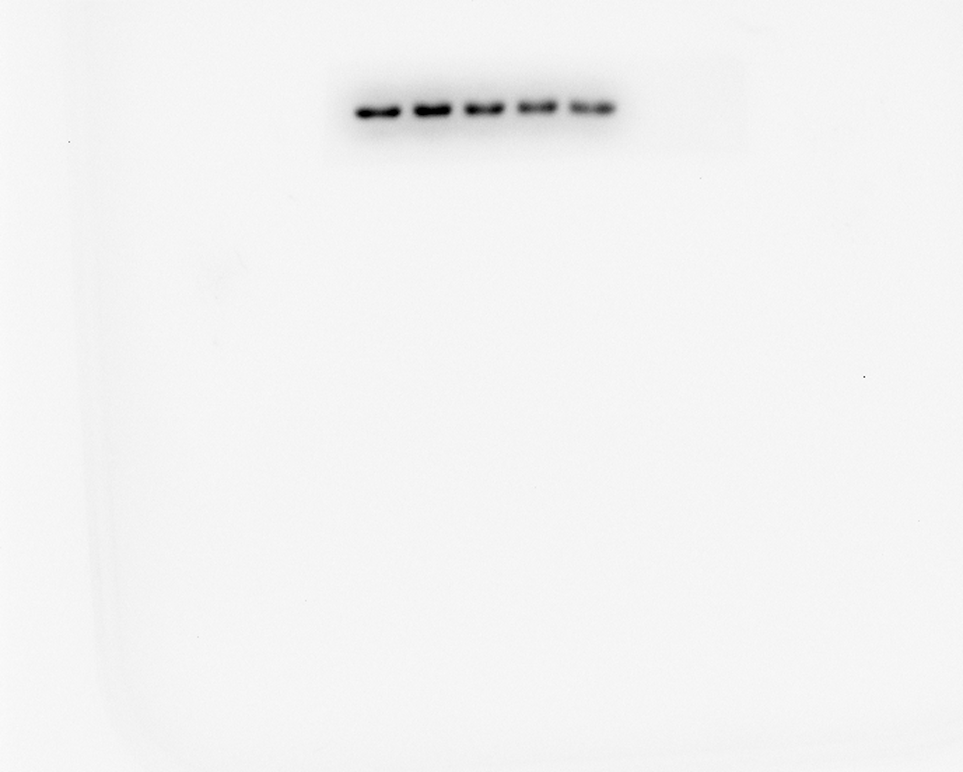

Supplement: Supplementary file 4 [file DataSheet8.ZIP › JNK2/JNK GAPDH 5.tif]

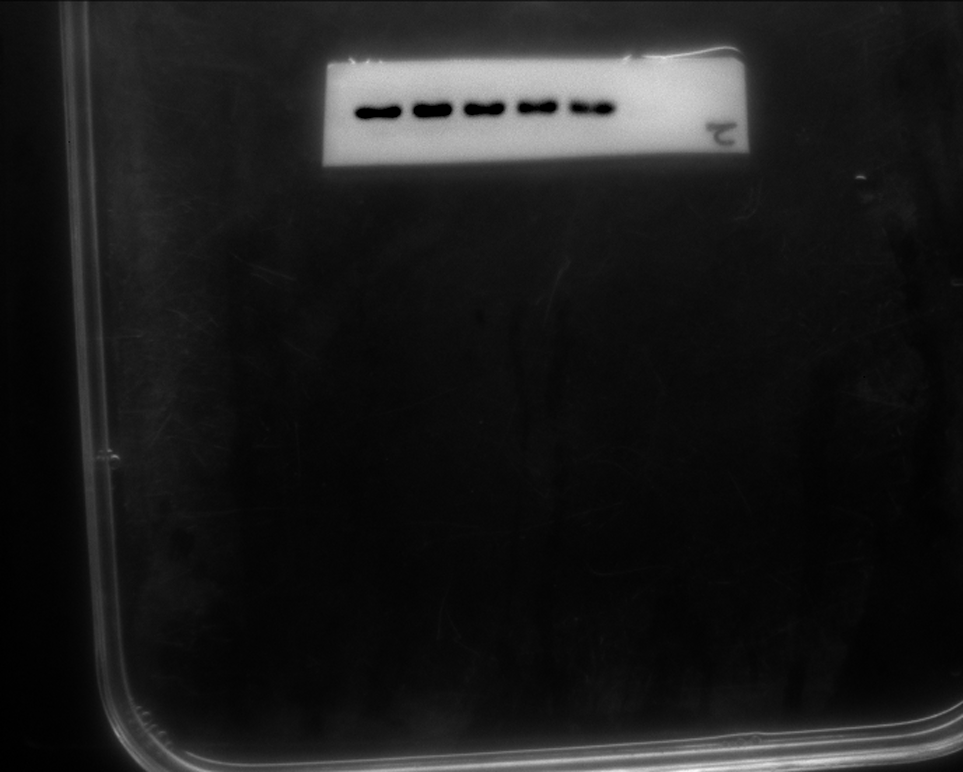

Supplement: Supplementary file 4 [file DataSheet8.ZIP › JNK2/JNK GAPDH q.tif]

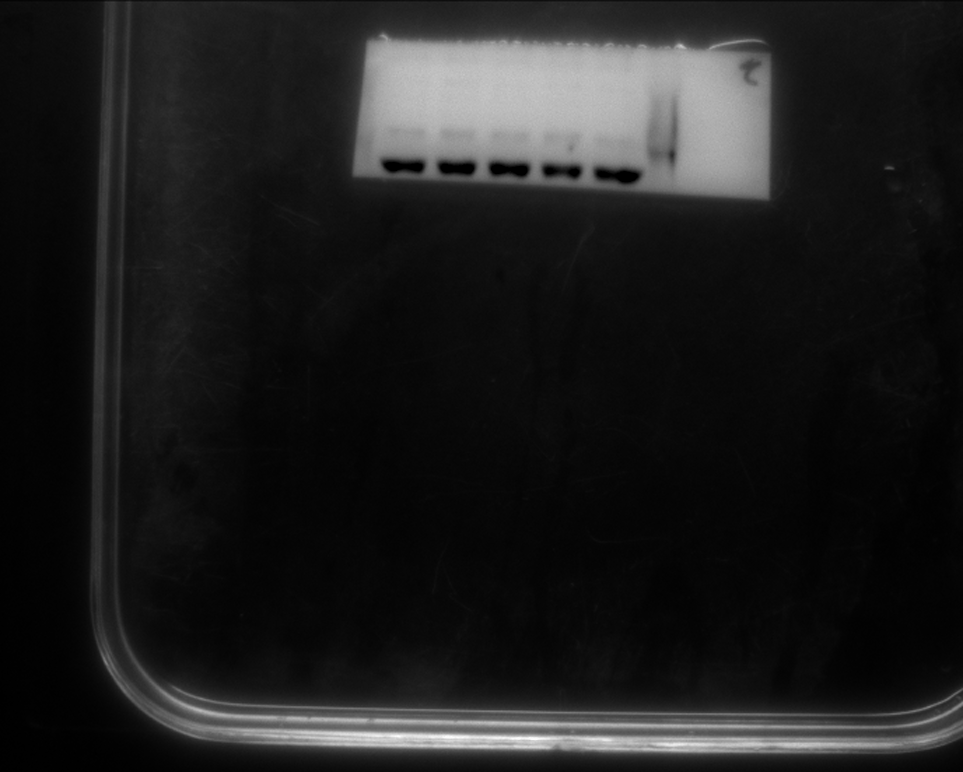

Supplement: Supplementary file 4 [file DataSheet8.ZIP › JNK2/JNK q.tif]

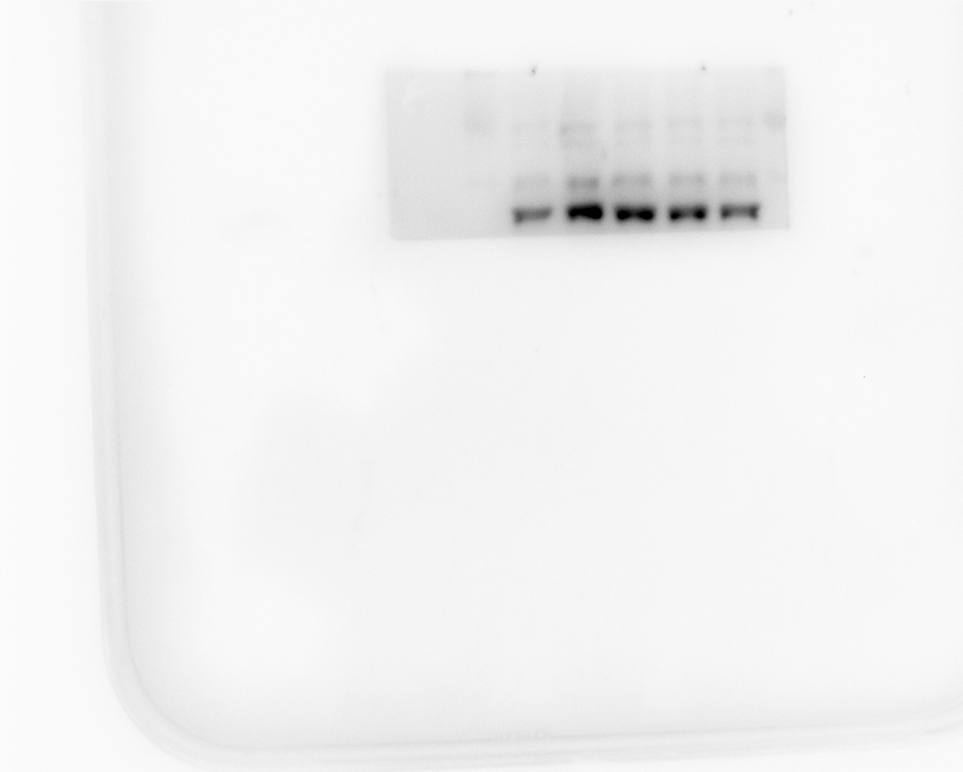

Supplement: Supplementary file 4 [file DataSheet8.ZIP › JNK2/p-JNK 1.tif]

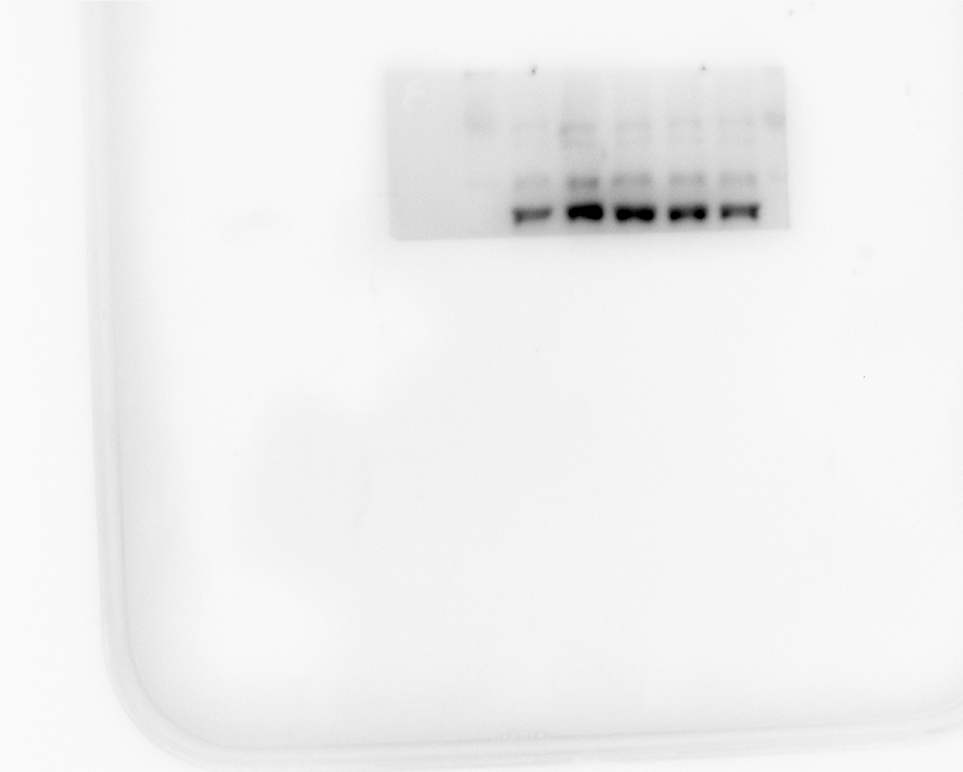

Supplement: Supplementary file 4 [file DataSheet8.ZIP › JNK2/p-JNK 2.tif]

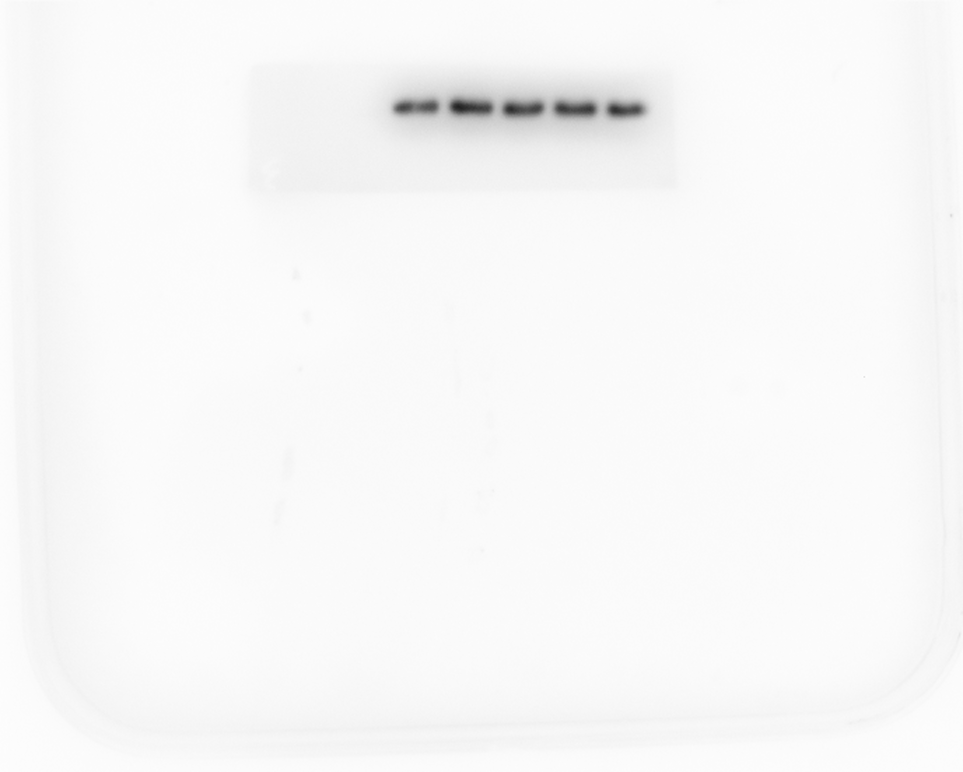

Supplement: Supplementary file 4 [file DataSheet8.ZIP › JNK2/p-JNK GAPDH 2.tif]

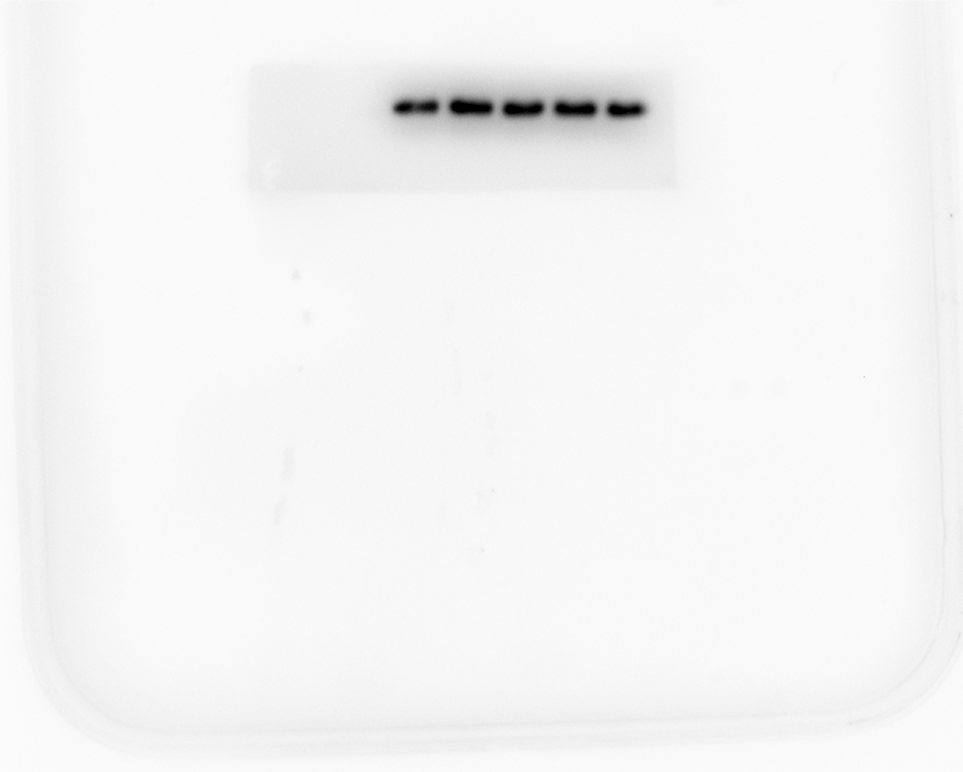

Supplement: Supplementary file 4 [file DataSheet8.ZIP › JNK2/p-JNK GAPDH 3.tif]

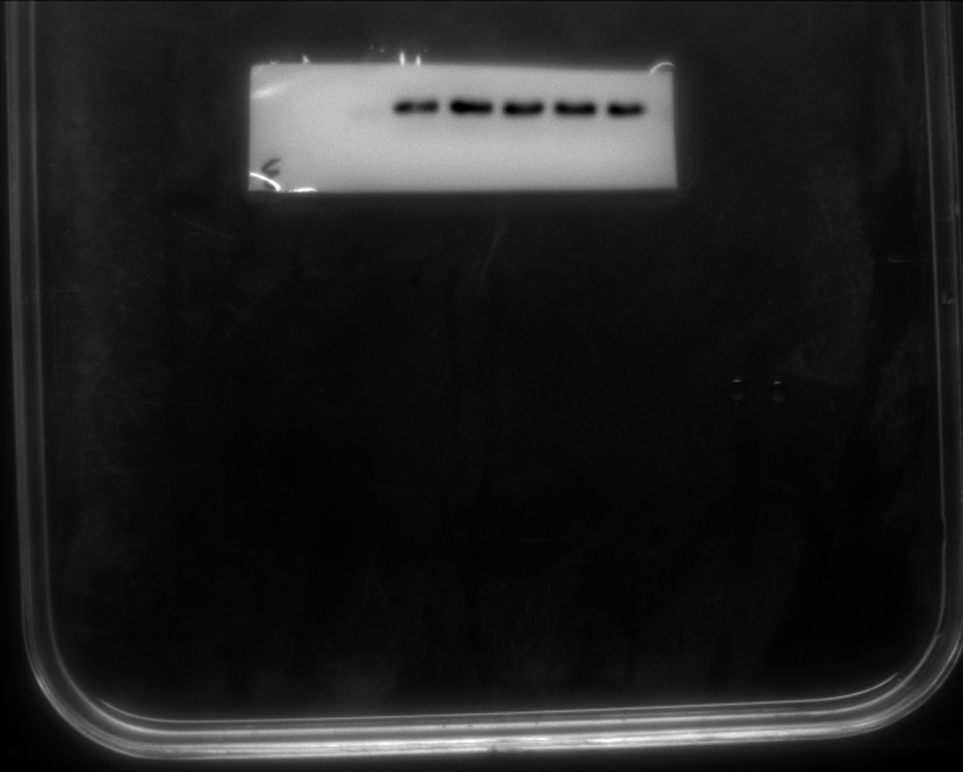

Supplement: Supplementary file 4 [file DataSheet8.ZIP › JNK2/p-JNK GAPDH q.tif]

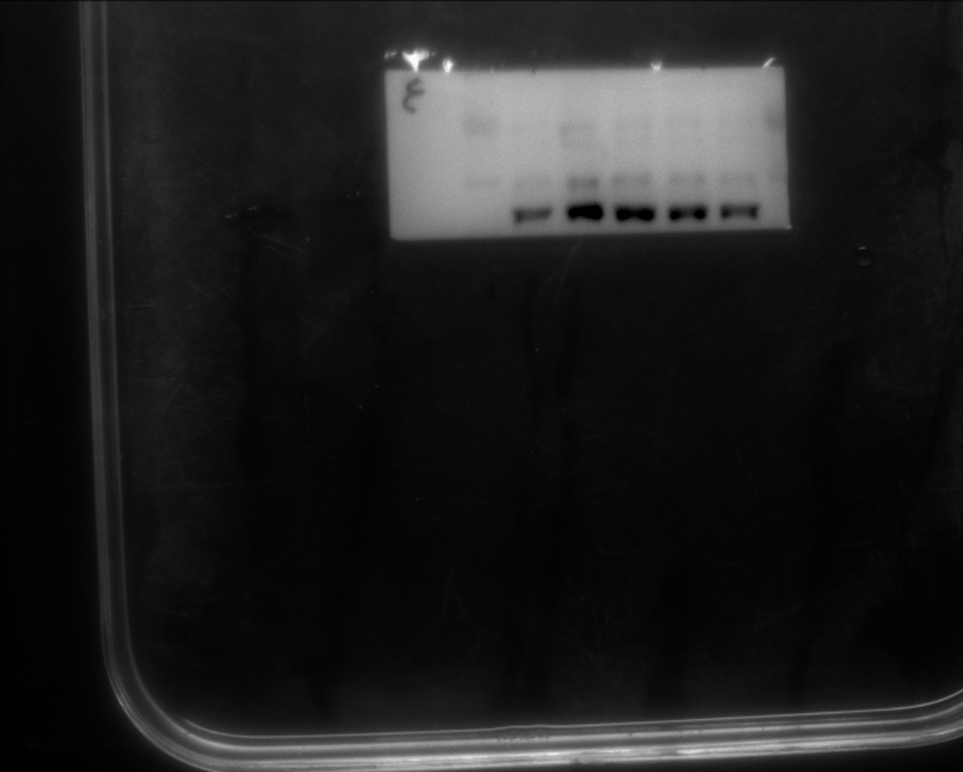

Supplement: Supplementary file 4 [file DataSheet8.ZIP › JNK2/p-JNK q.tif]

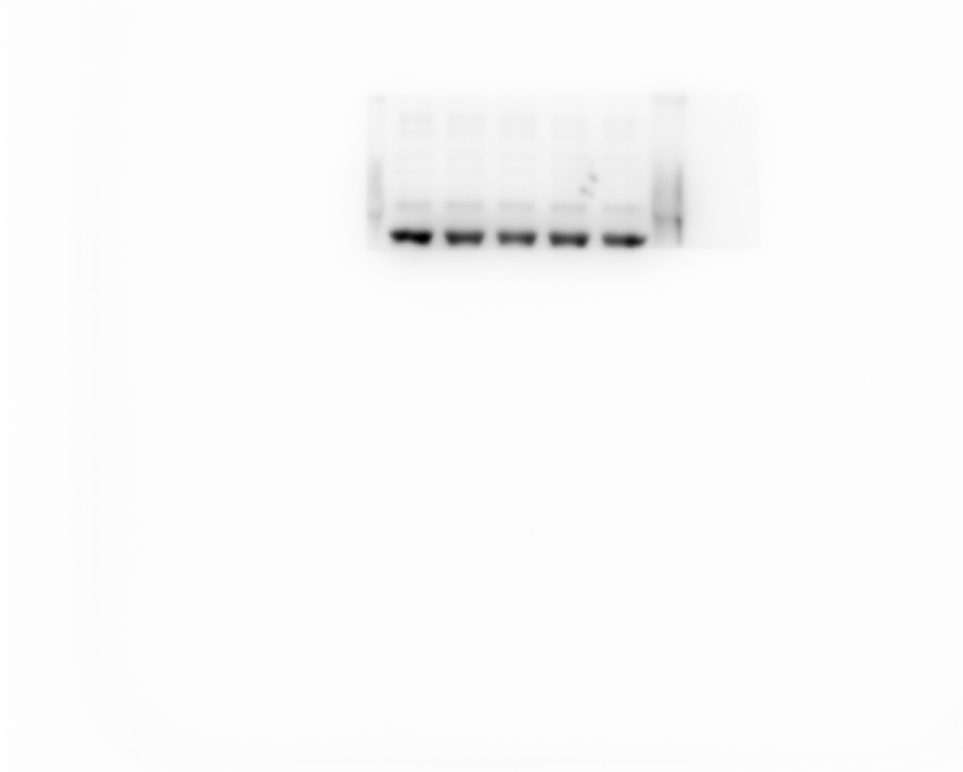

Supplement: Supplementary file 4 [file DataSheet8.ZIP › JNK3/JNK 1.tif]

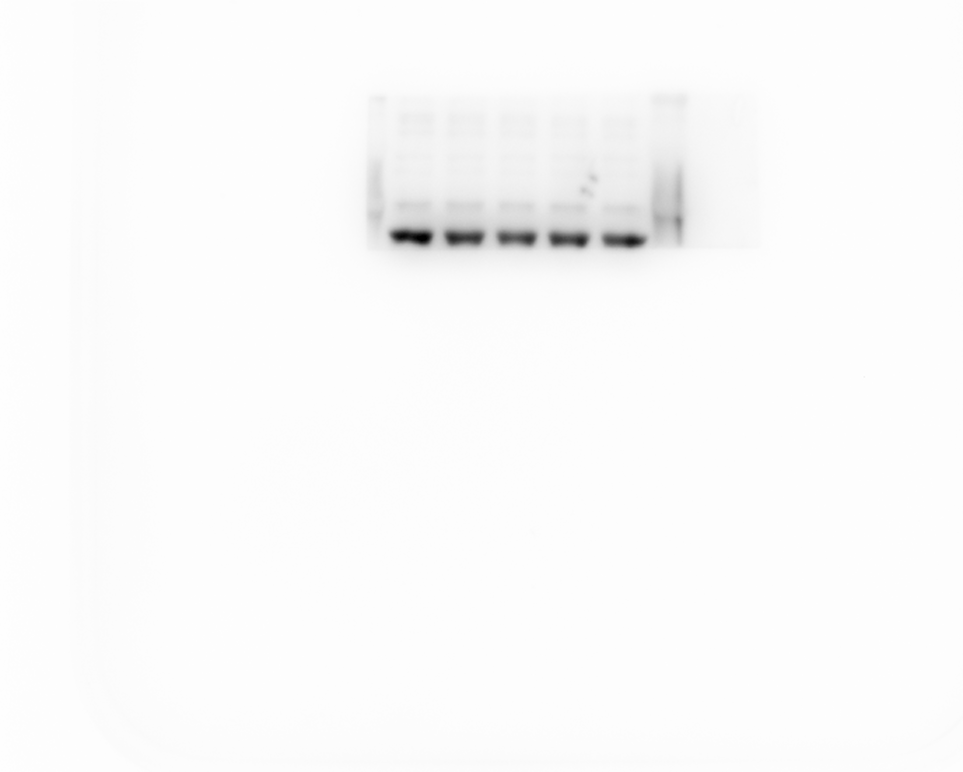

Supplement: Supplementary file 4 [file DataSheet8.ZIP › JNK3/JNK 2.tif]

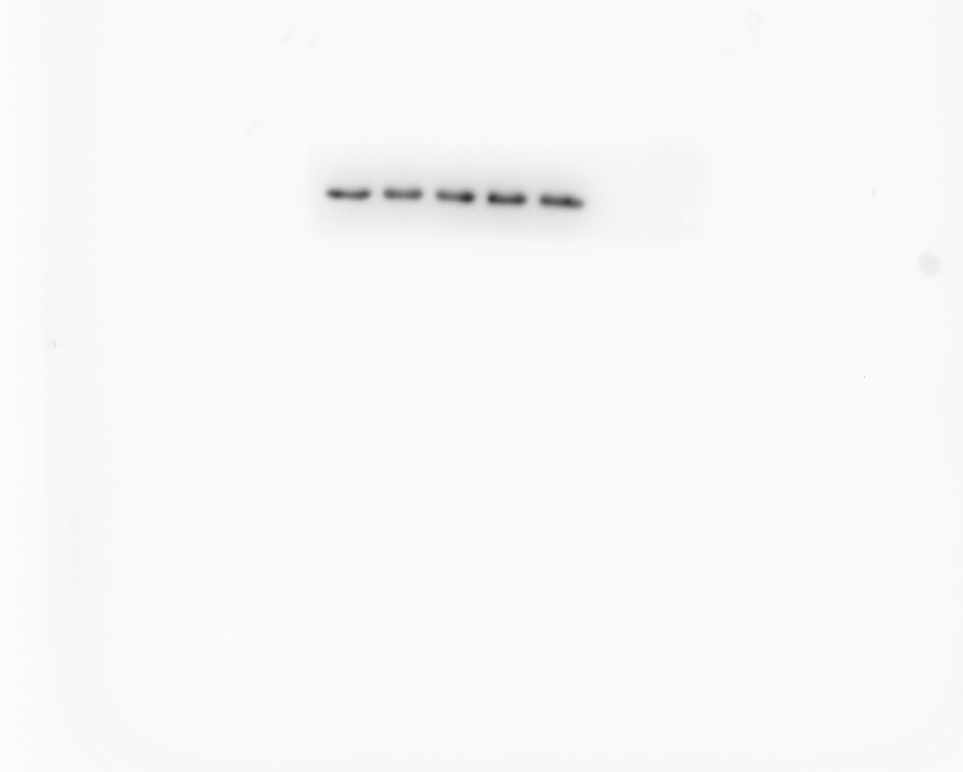

Supplement: Supplementary file 4 [file DataSheet8.ZIP › JNK3/JNK GAPDH 1.tif]

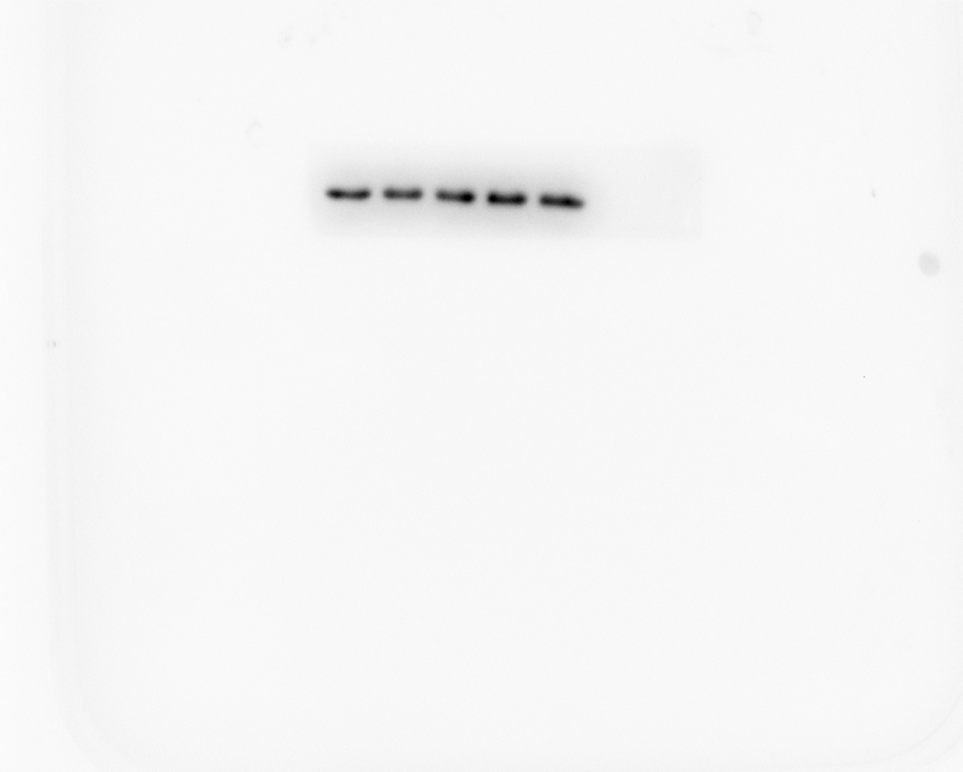

Supplement: Supplementary file 4 [file DataSheet8.ZIP › JNK3/JNK GAPDH 2.tif]

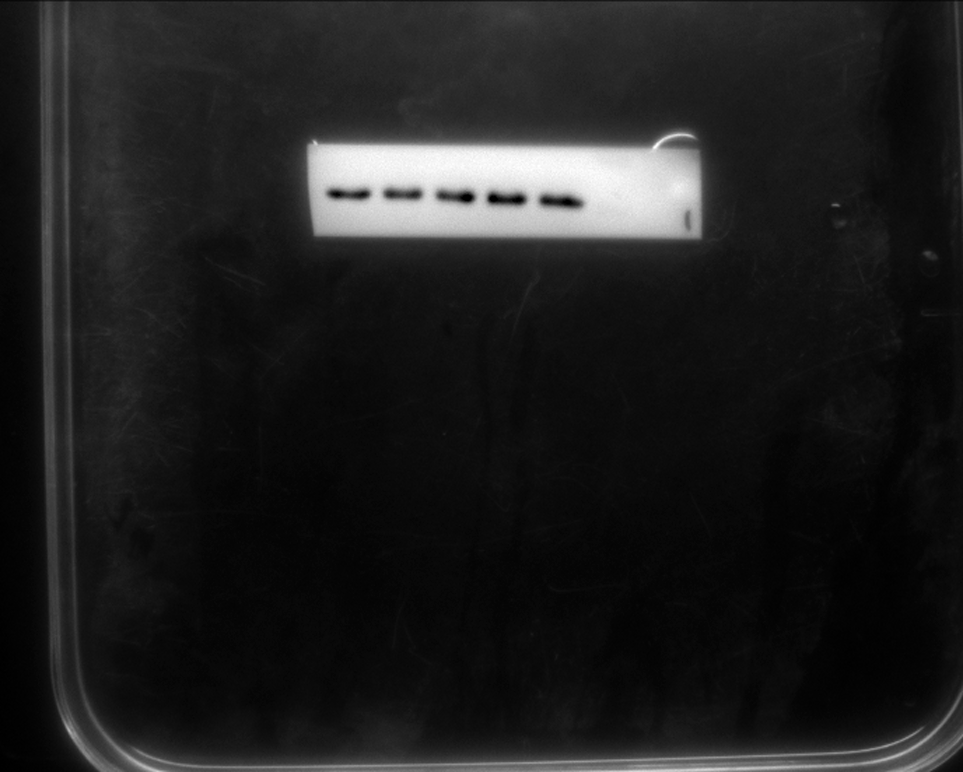

Supplement: Supplementary file 4 [file DataSheet8.ZIP › JNK3/JNK GAPDH q.tif]

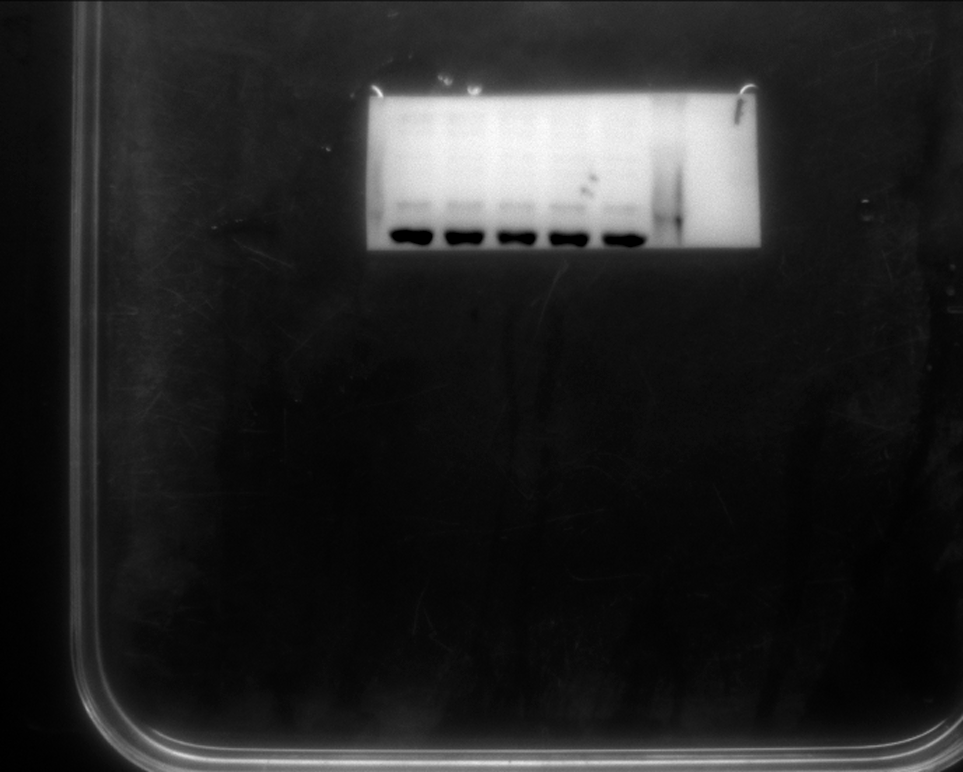

Supplement: Supplementary file 4 [file DataSheet8.ZIP › JNK3/JNK q.tif]

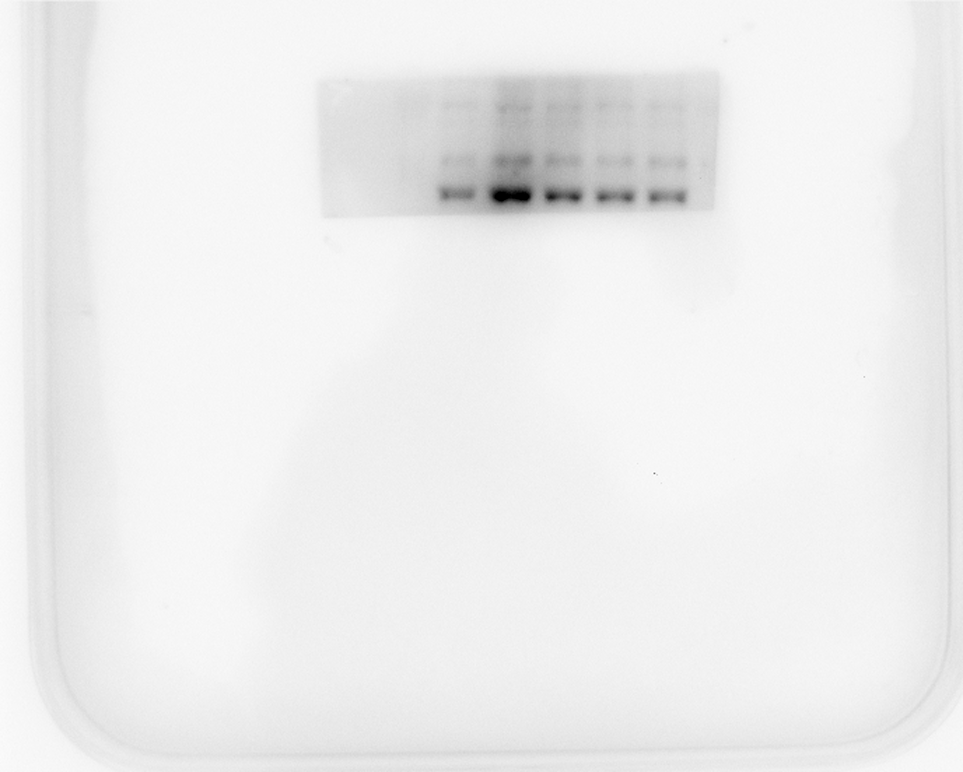

Supplement: Supplementary file 4 [file DataSheet8.ZIP › JNK3/p-JNK 1.tif]

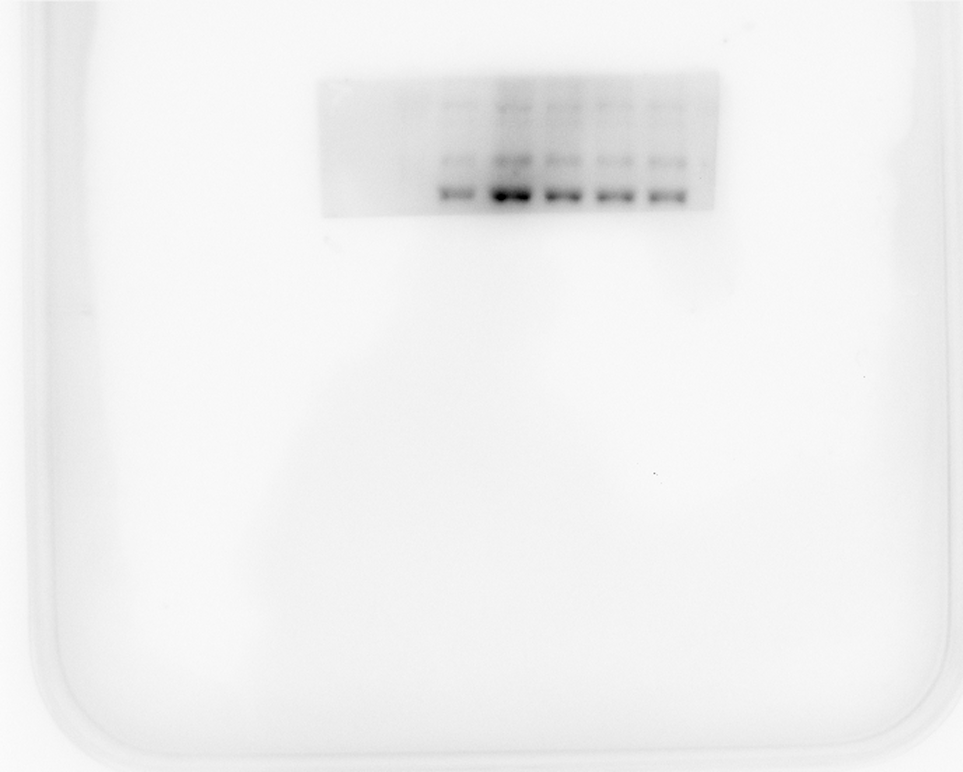

Supplement: Supplementary file 4 [file DataSheet8.ZIP › JNK3/p-JNK 2.tif]

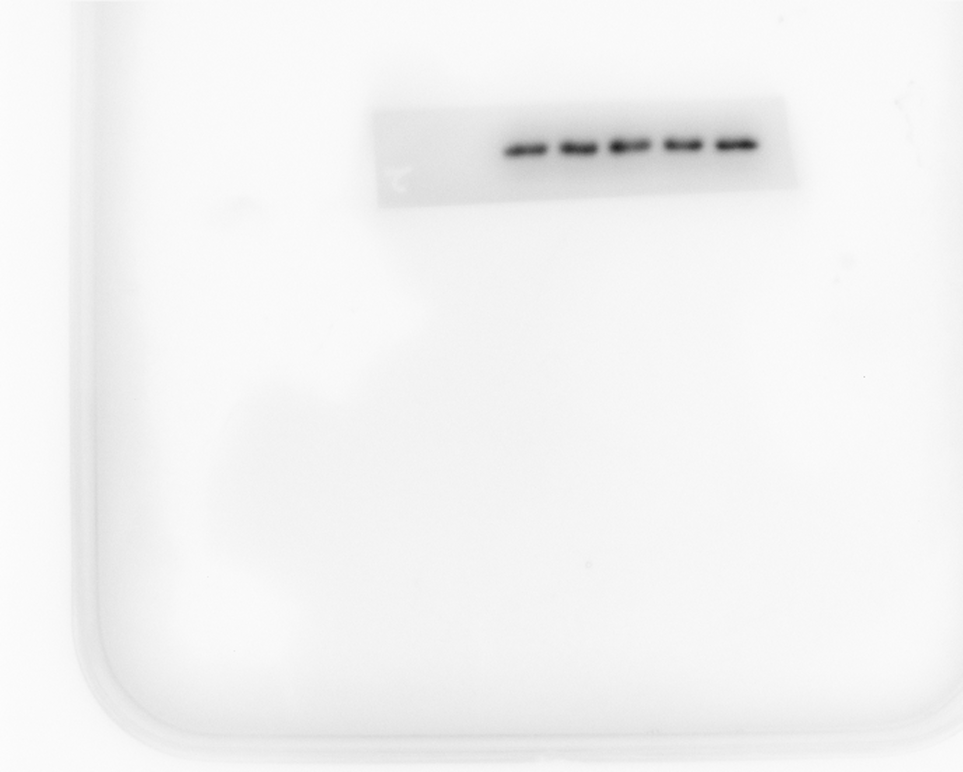

Supplement: Supplementary file 4 [file DataSheet8.ZIP › JNK3/p-JNK GAPDH 1.tif]

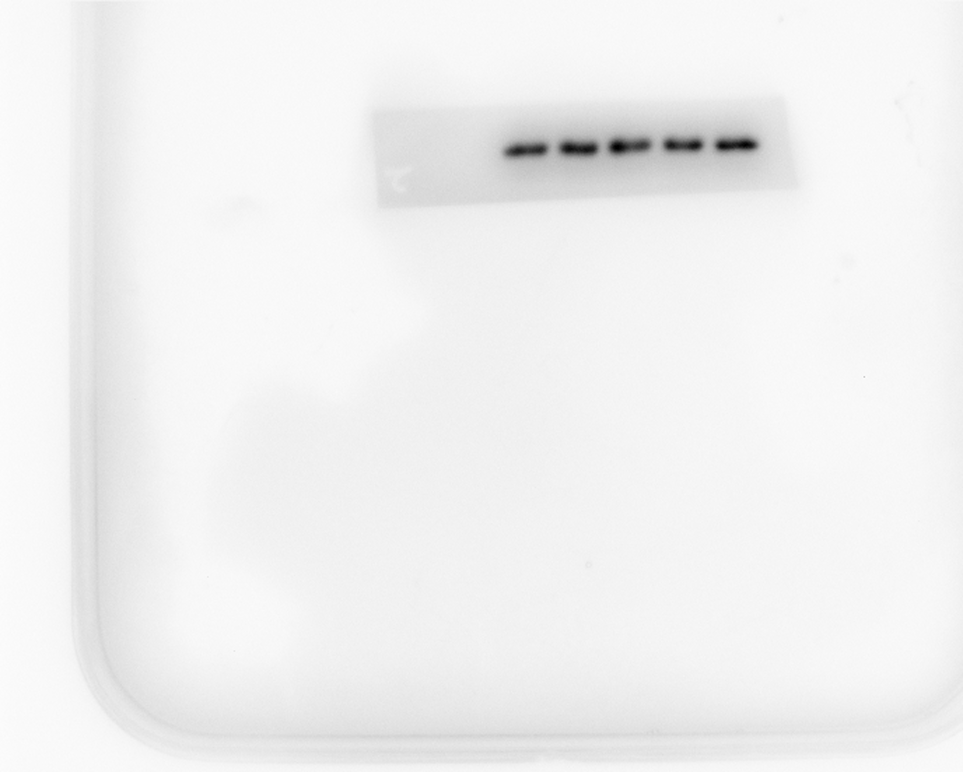

Supplement: Supplementary file 4 [file DataSheet8.ZIP › JNK3/p-JNK GAPDH 2.tif]

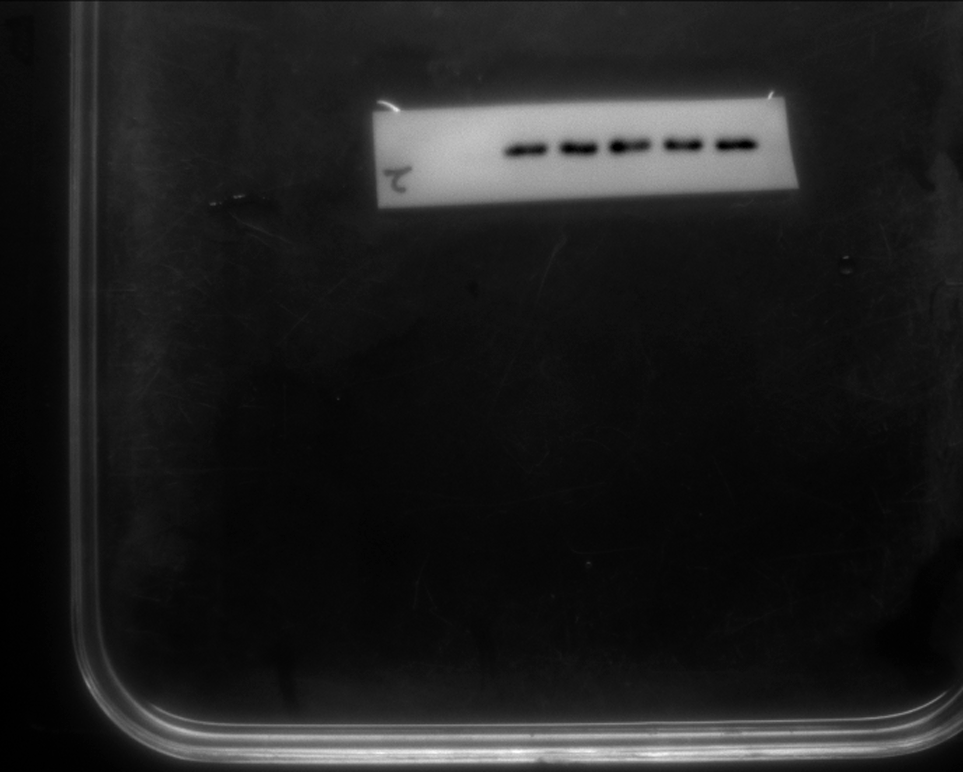

Supplement: Supplementary file 4 [file DataSheet8.ZIP › JNK3/p-JNK GAPDH q.tif]
