# Supplementary figures and images for: Valsartan attenuates LPS-induced ALI by modulating NF-κB and MAPK pathways (part 2 of 4)
Source: Front Pharmacol. 2024 Jan 15;15:1321095. doi: 10.3389/fphar.2024.1321095 (PMC10822936; doi:10.3389/fphar.2024.1321095)

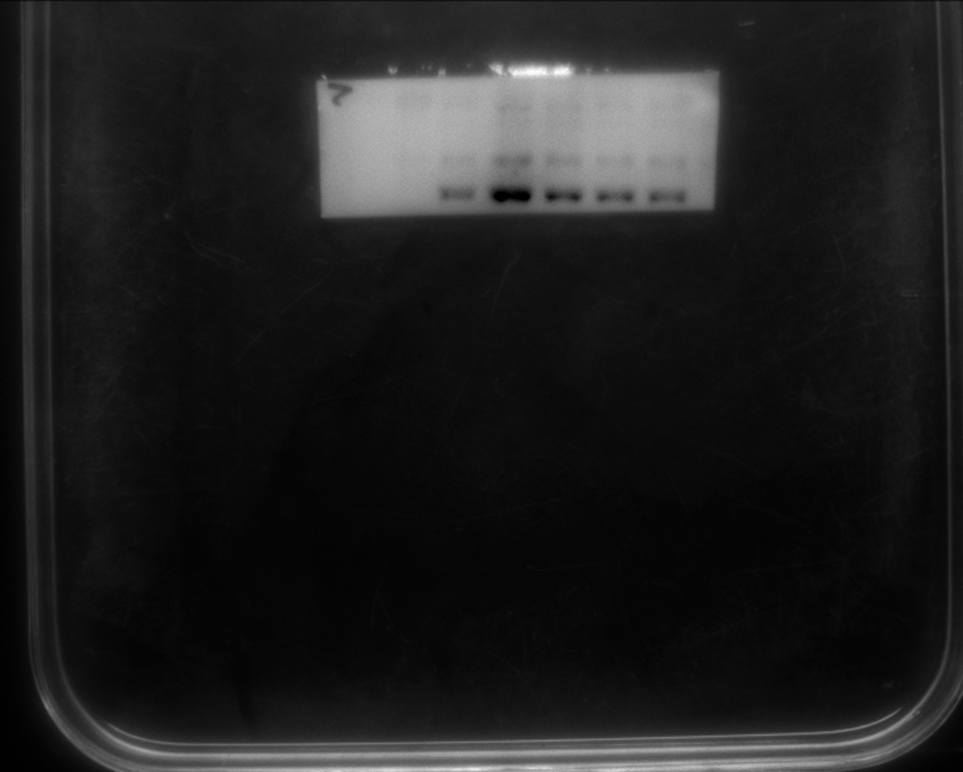

Supplement: Supplementary file 4 [file DataSheet8.ZIP › JNK3/p-JNK q.tif]

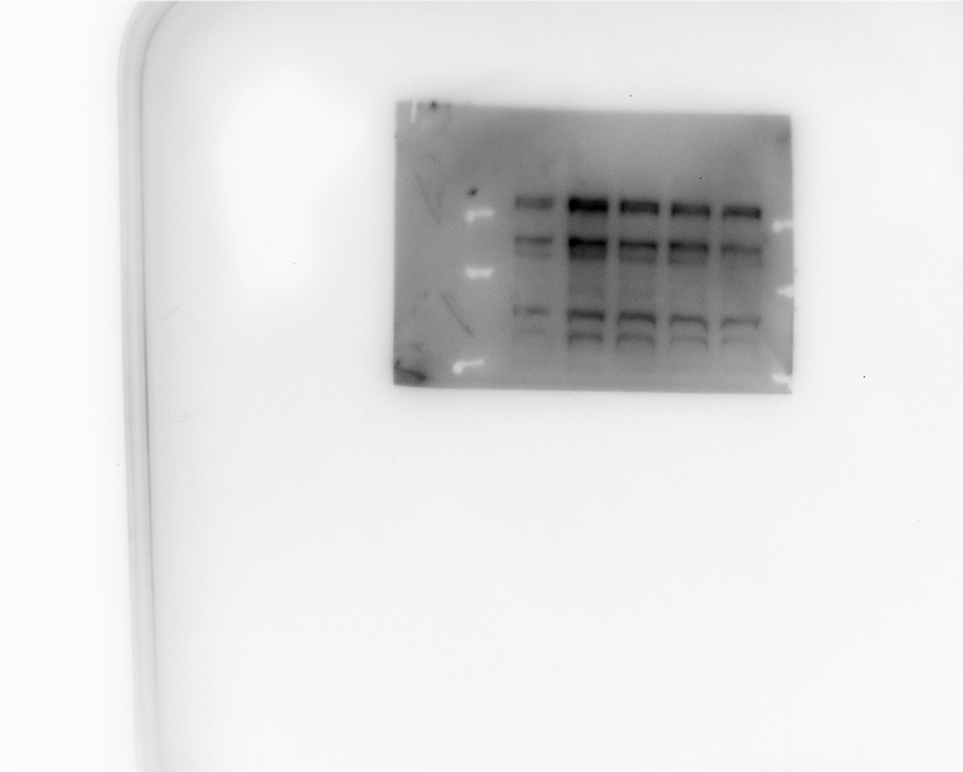

Supplement: Supplementary file 5 [file DataSheet9.ZIP › 1/muc5ac 1.tif]

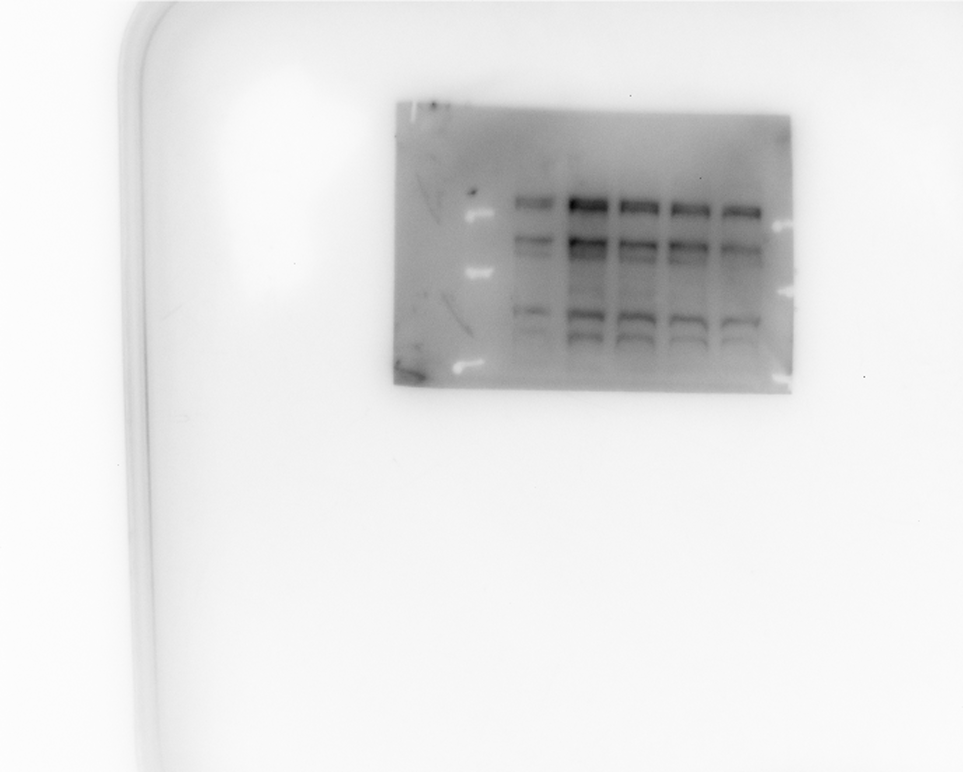

Supplement: Supplementary file 5 [file DataSheet9.ZIP › 1/muc5ac 2.tif]

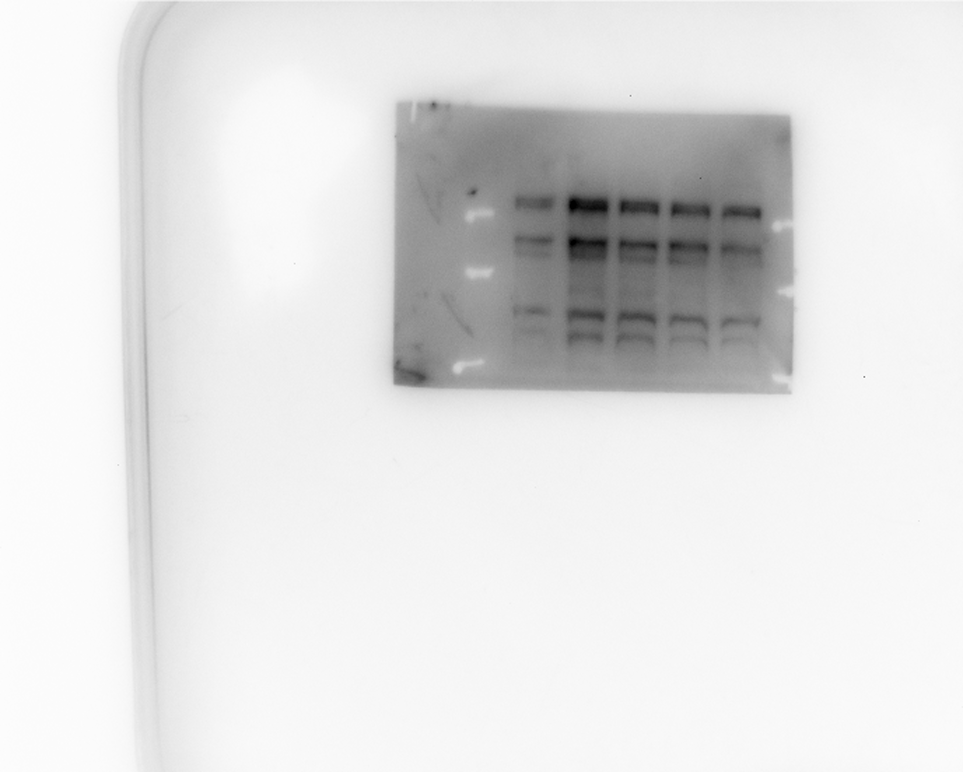

Supplement: Supplementary file 5 [file DataSheet9.ZIP › 1/muc5ac 3.tif]

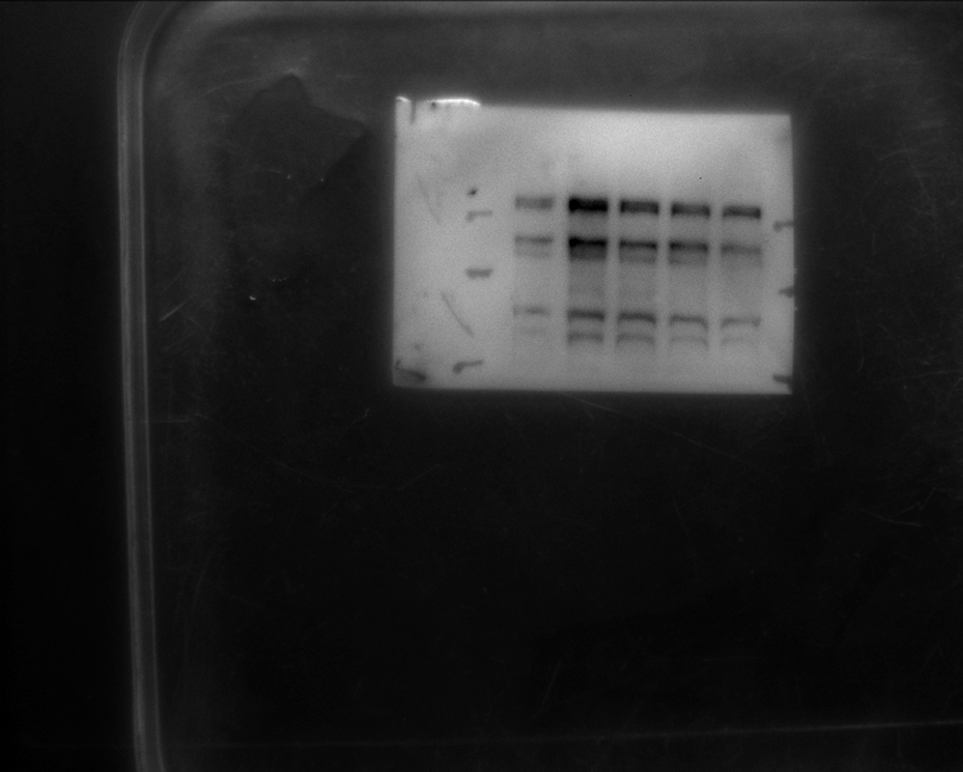

Supplement: Supplementary file 5 [file DataSheet9.ZIP › 1/muc5ac q.tif]

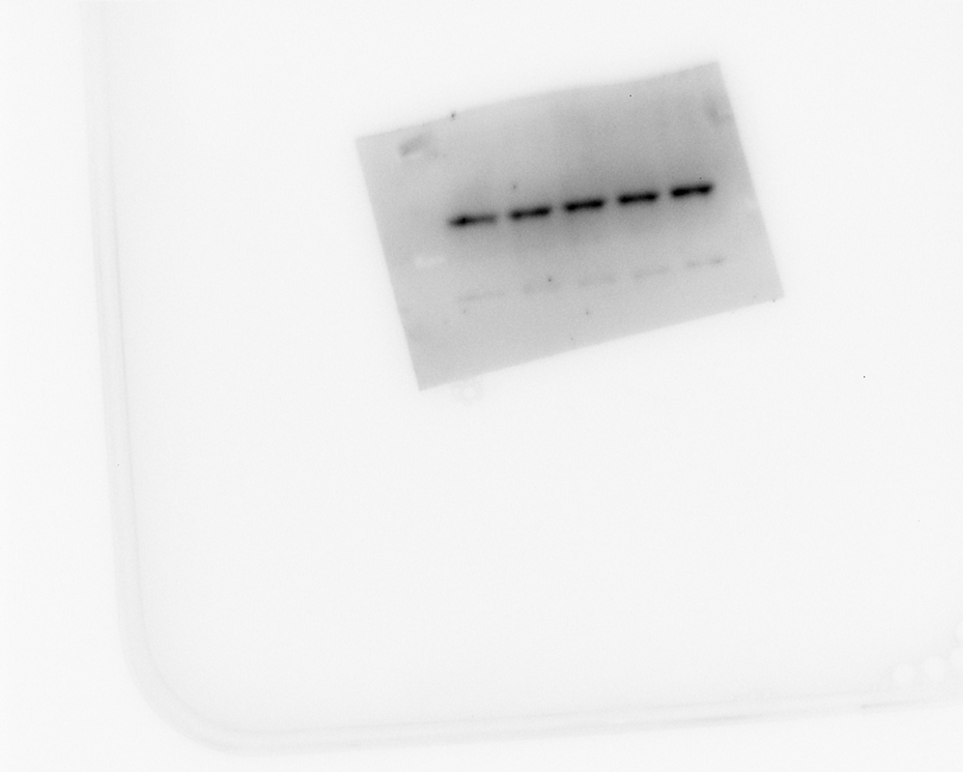

Supplement: Supplementary file 5 [file DataSheet9.ZIP › 1/muc5ac tublin 1.tif]

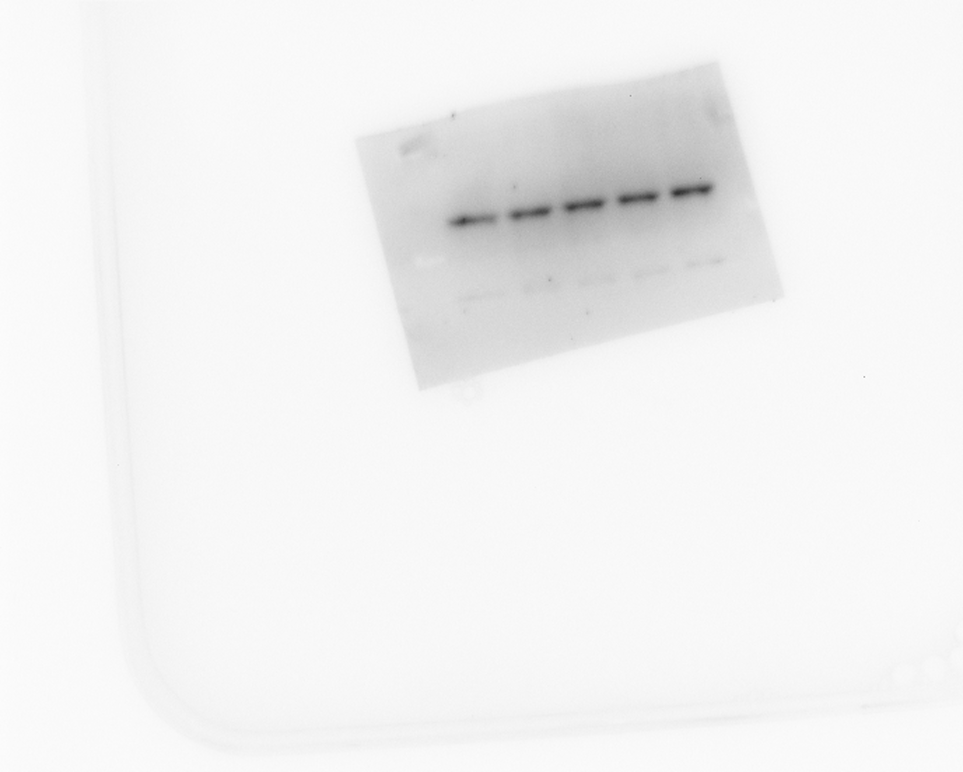

Supplement: Supplementary file 5 [file DataSheet9.ZIP › 1/muc5ac tublin 2.tif]

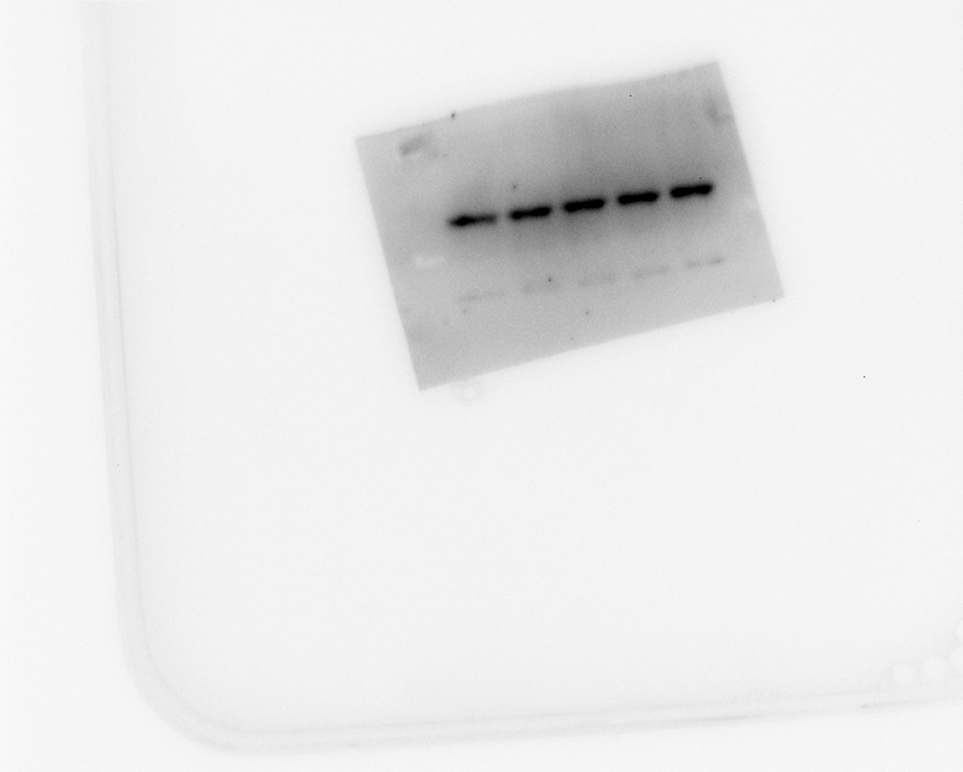

Supplement: Supplementary file 5 [file DataSheet9.ZIP › 1/muc5ac tublin 3.tif]

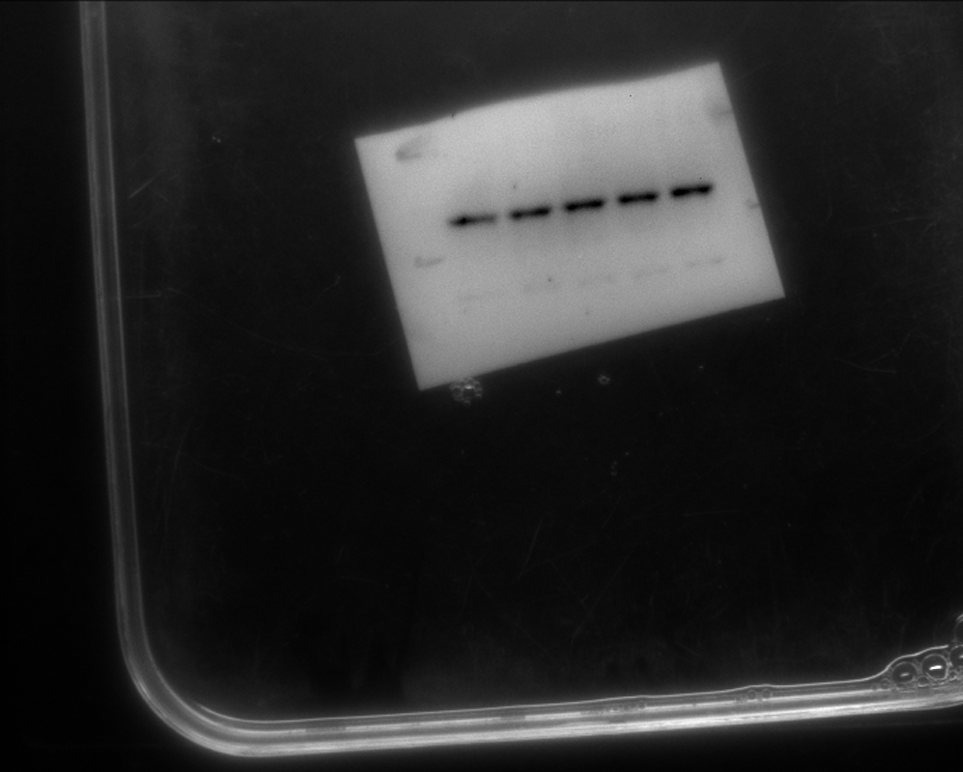

Supplement: Supplementary file 5 [file DataSheet9.ZIP › 1/muc5ac tublin q.tif]

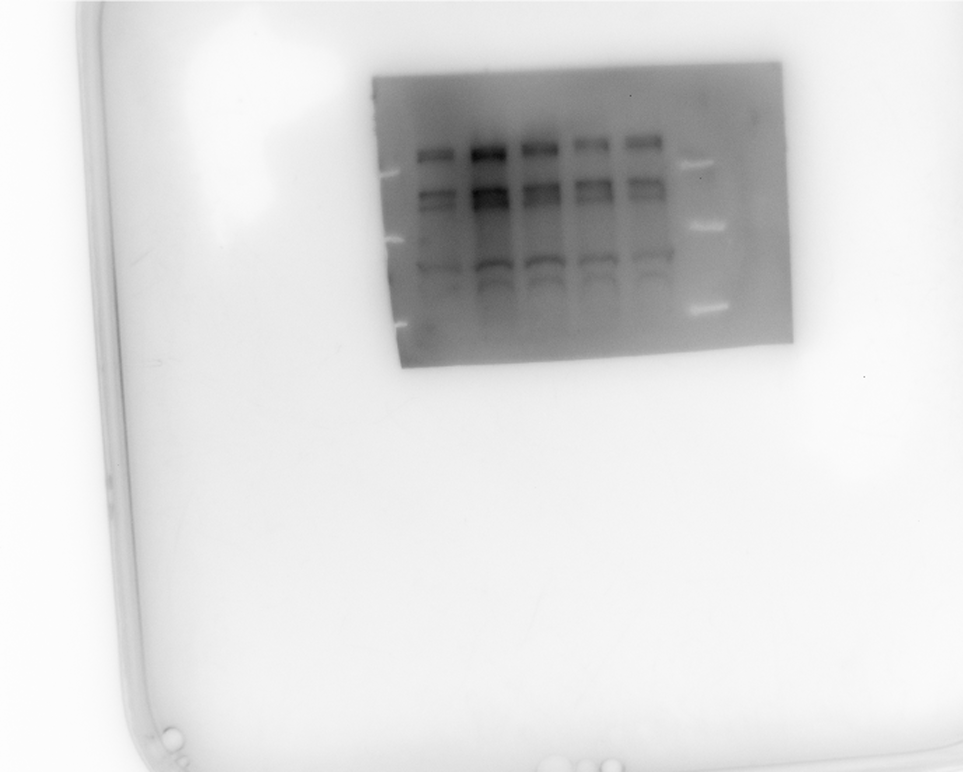

Supplement: Supplementary file 5 [file DataSheet9.ZIP › 2/muc5ac 1.tif]

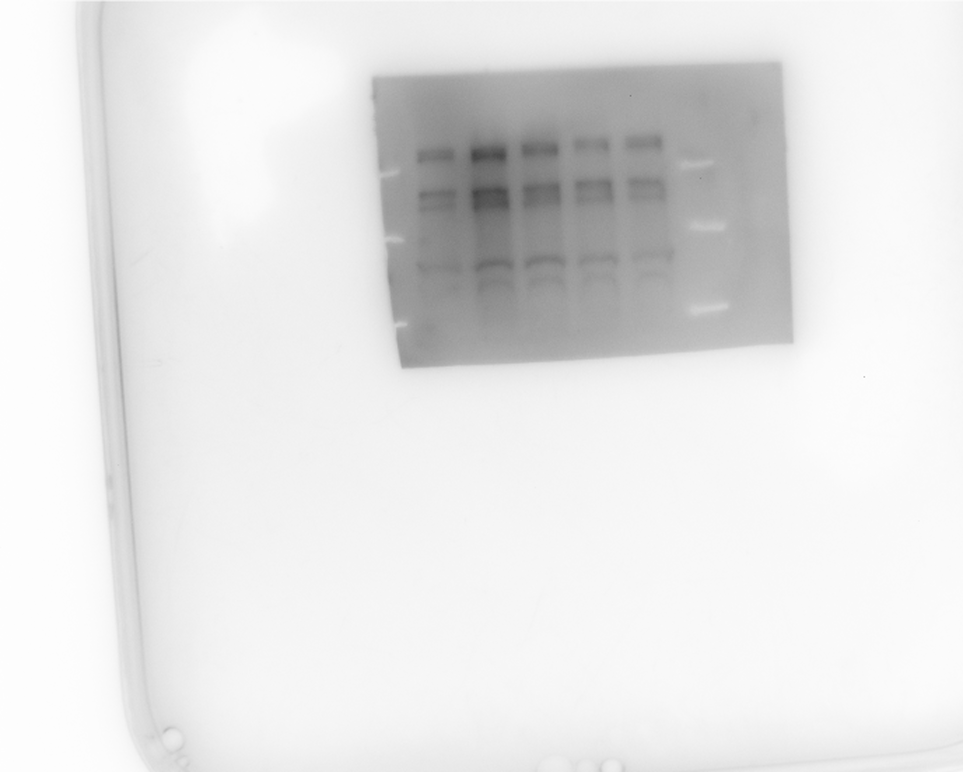

Supplement: Supplementary file 5 [file DataSheet9.ZIP › 2/muc5ac 2.tif]

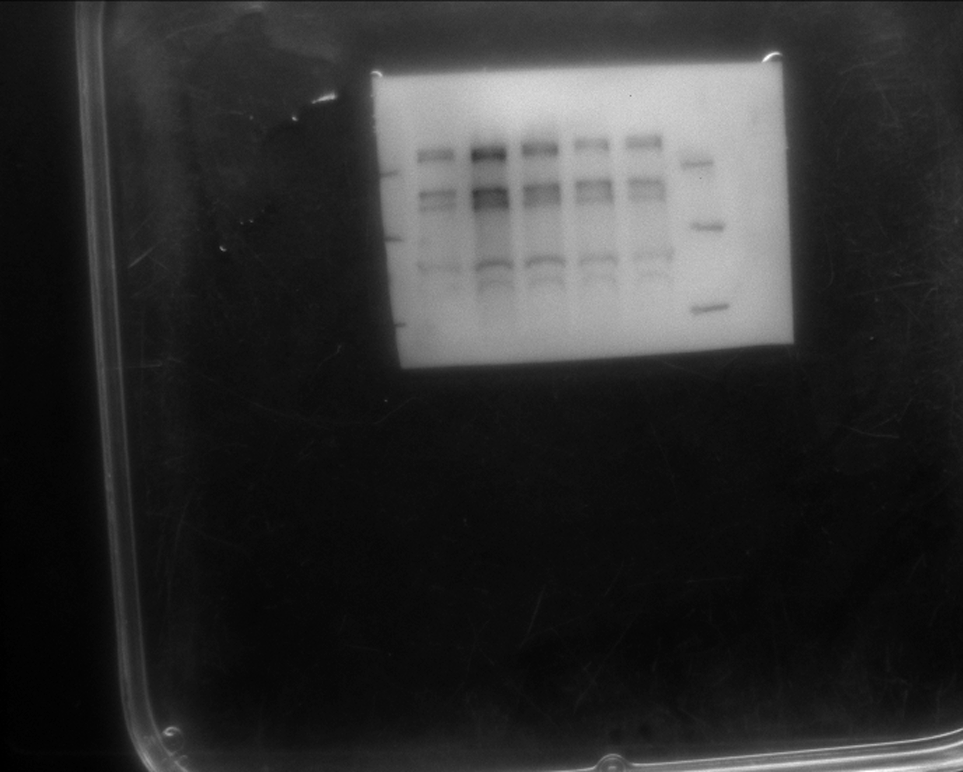

Supplement: Supplementary file 5 [file DataSheet9.ZIP › 2/muc5ac q.tif]

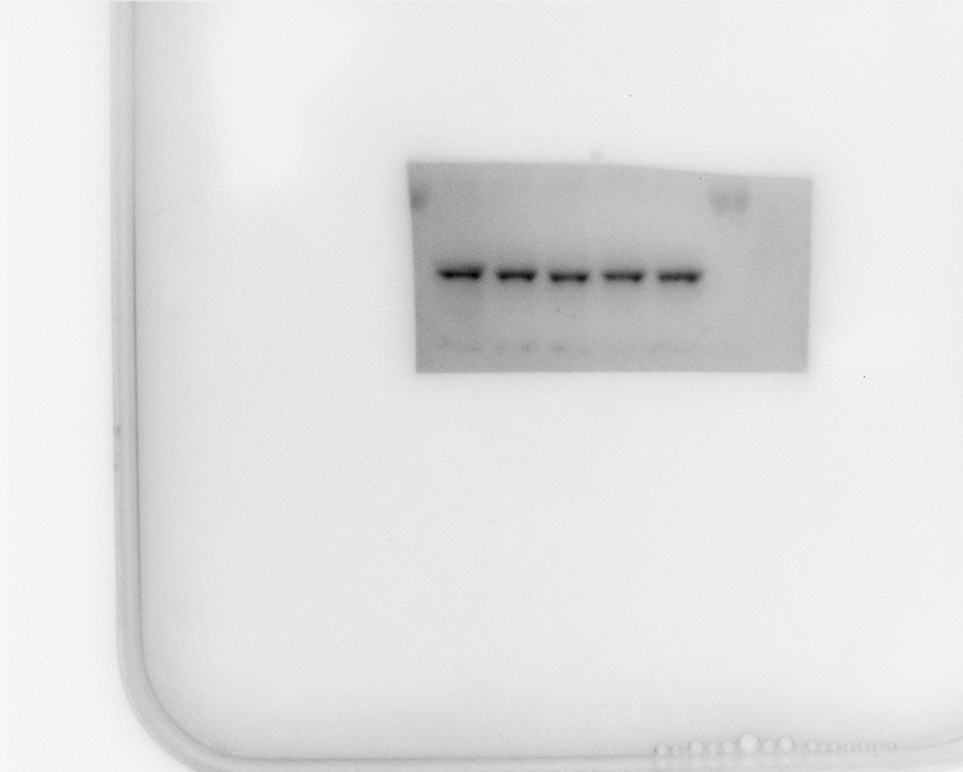

Supplement: Supplementary file 5 [file DataSheet9.ZIP › 2/muc5ac tublin 1.tif]

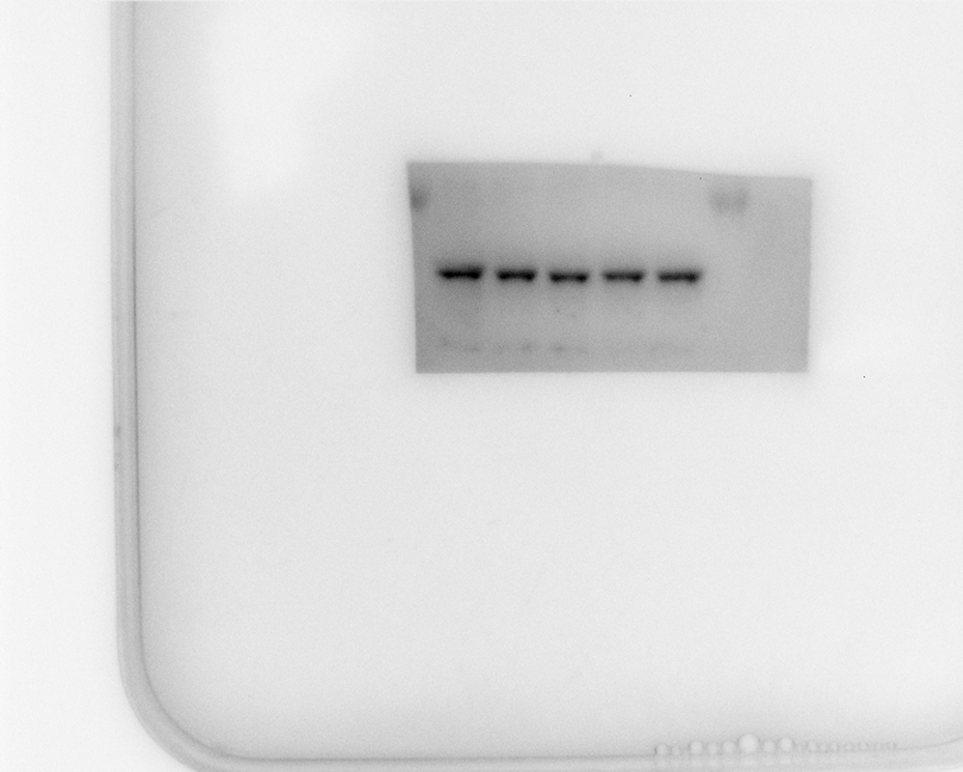

Supplement: Supplementary file 5 [file DataSheet9.ZIP › 2/muc5ac tublin 2.tif]

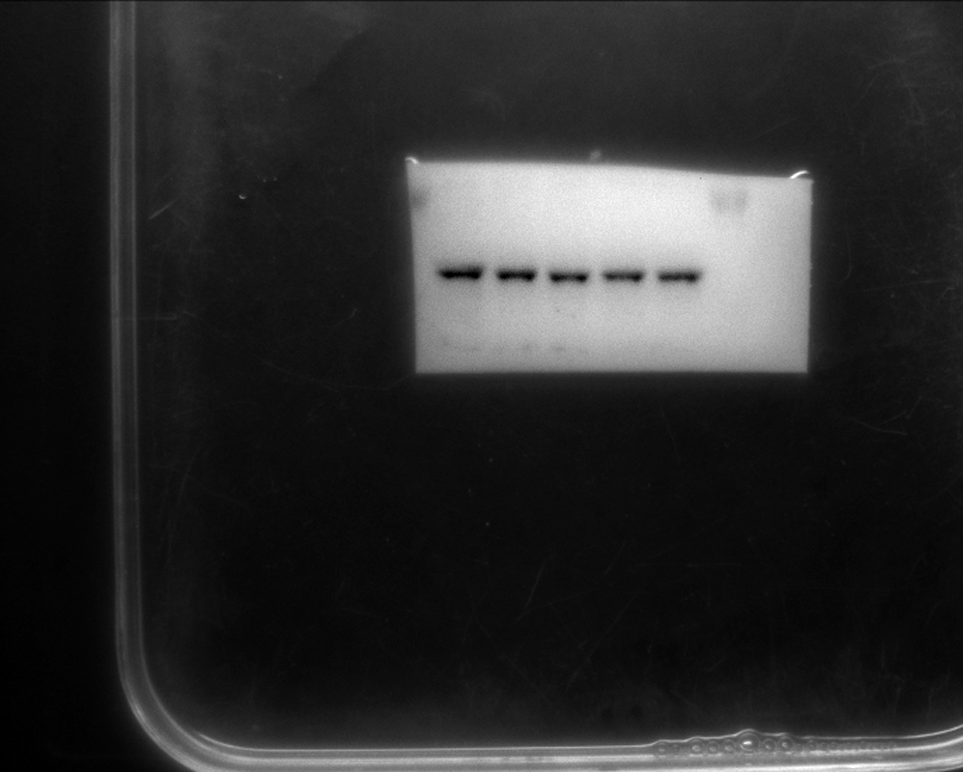

Supplement: Supplementary file 5 [file DataSheet9.ZIP › 2/muc5ac tublin q.tif]

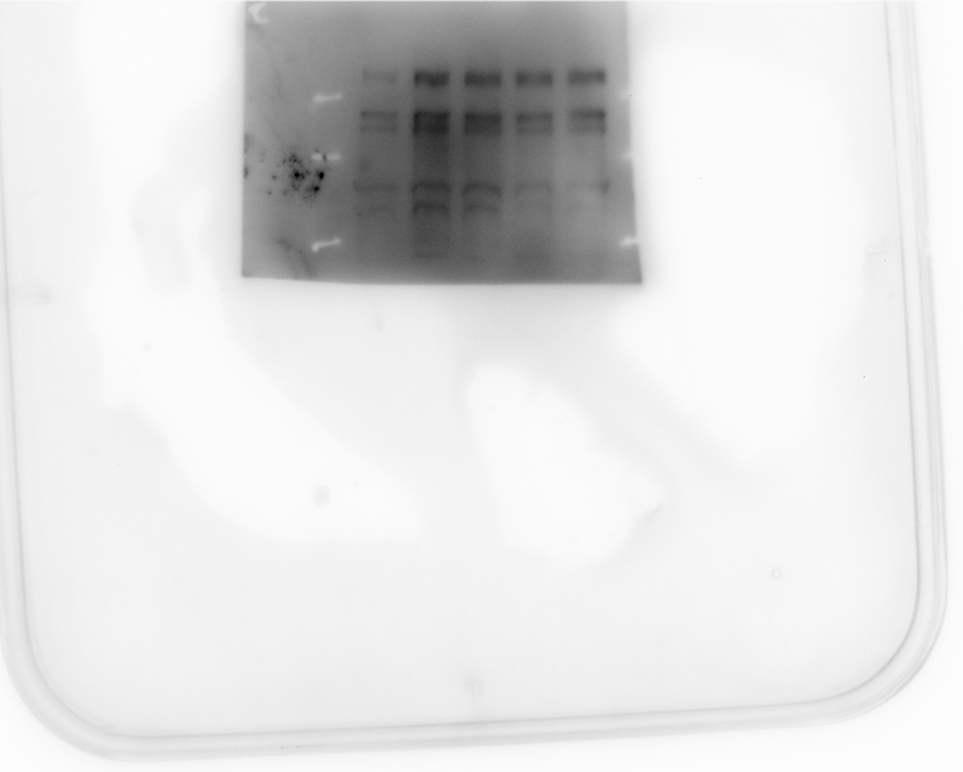

Supplement: Supplementary file 5 [file DataSheet9.ZIP › 3/muc5ac 1.tif]

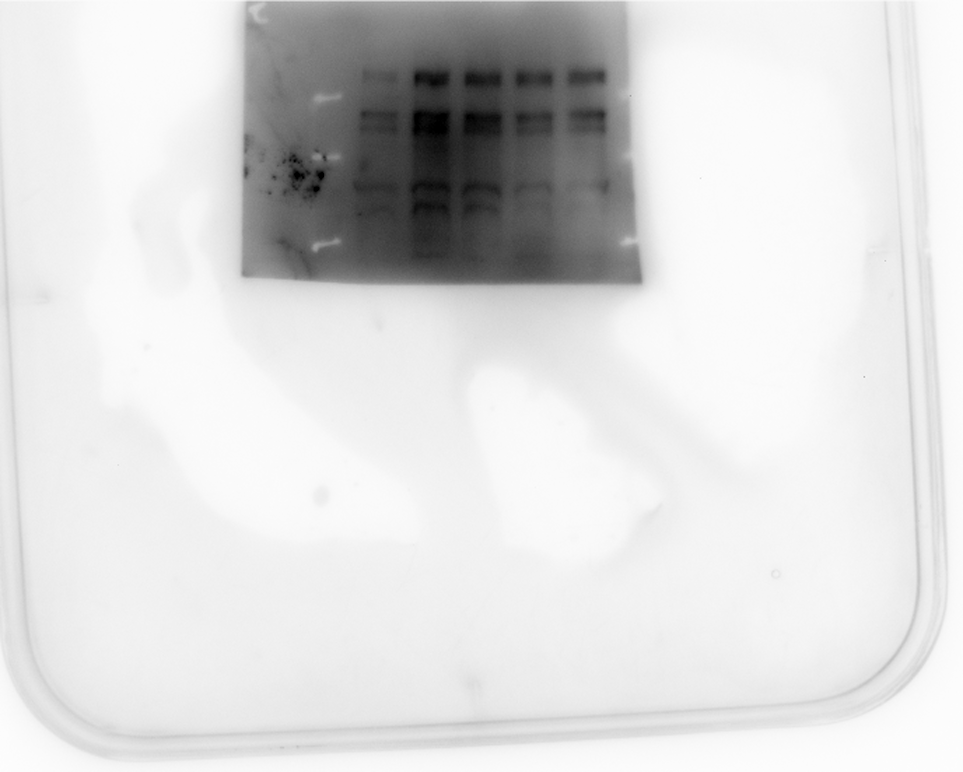

Supplement: Supplementary file 5 [file DataSheet9.ZIP › 3/muc5ac 2.tif]

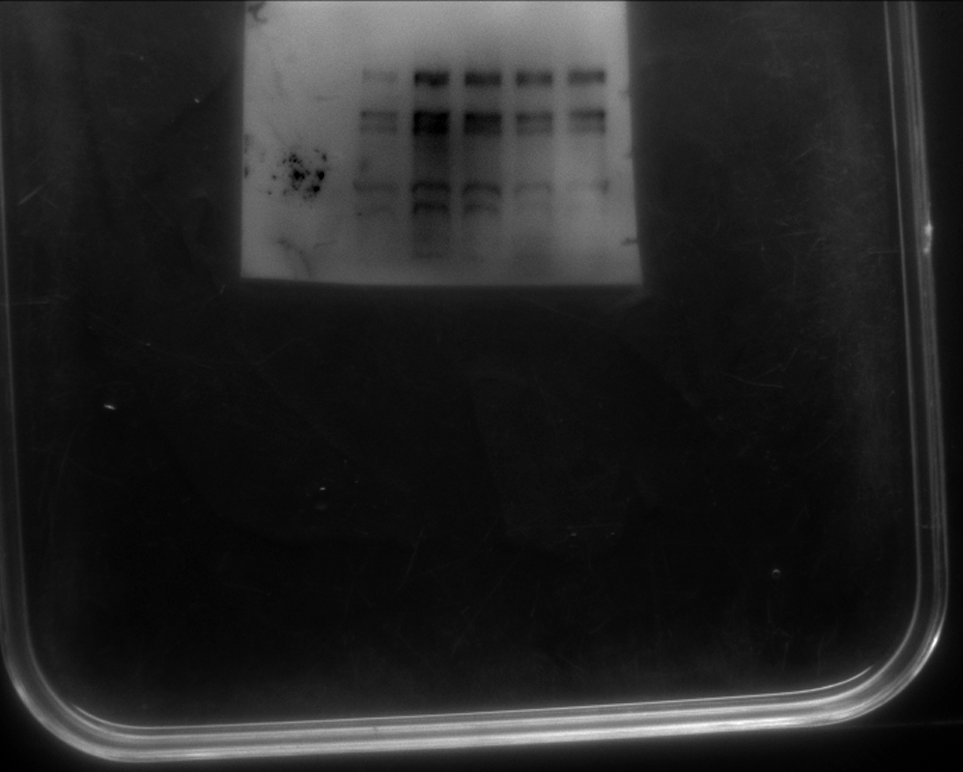

Supplement: Supplementary file 5 [file DataSheet9.ZIP › 3/muc5ac q.tif]

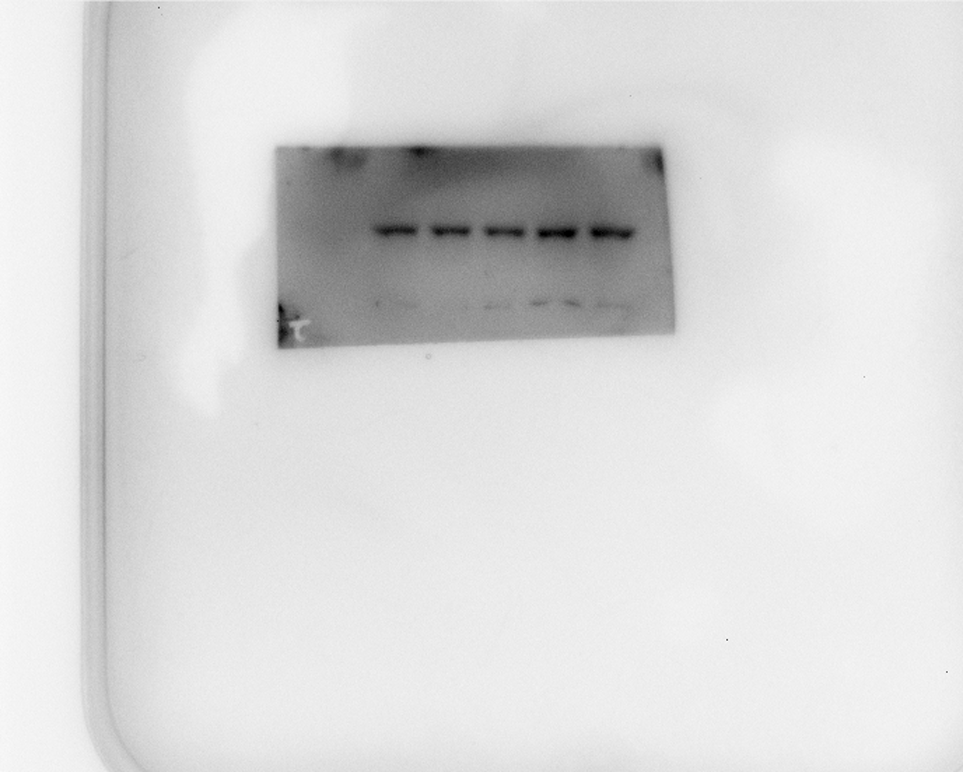

Supplement: Supplementary file 5 [file DataSheet9.ZIP › 3/muc5ac tublin 1.tif]

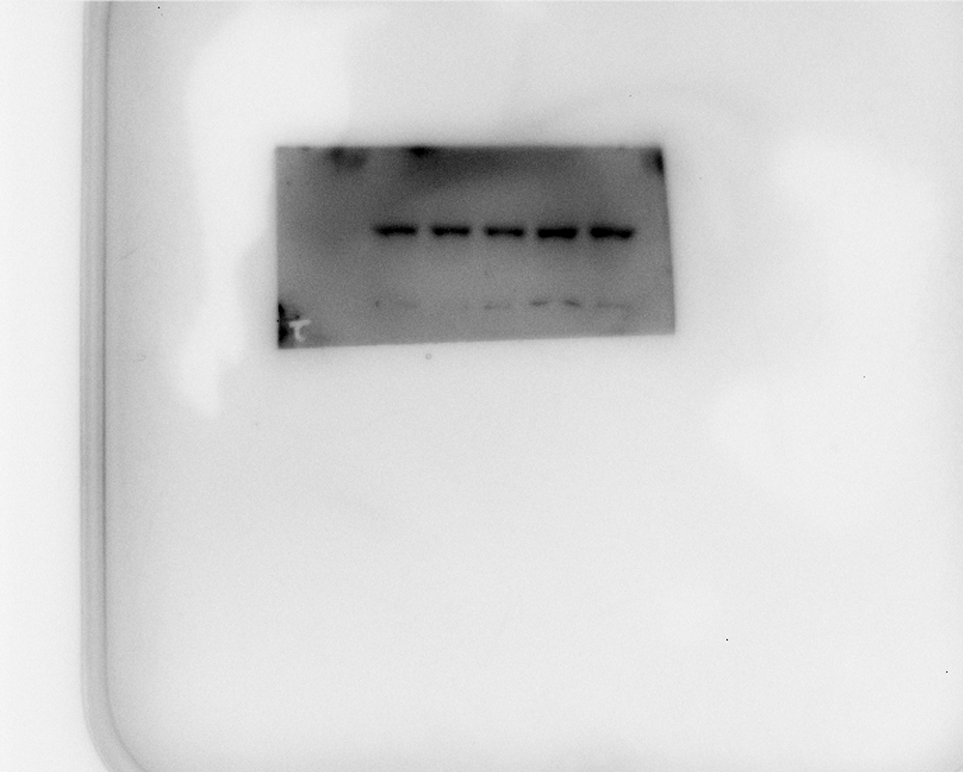

Supplement: Supplementary file 5 [file DataSheet9.ZIP › 3/muc5ac tublin 2.tif]

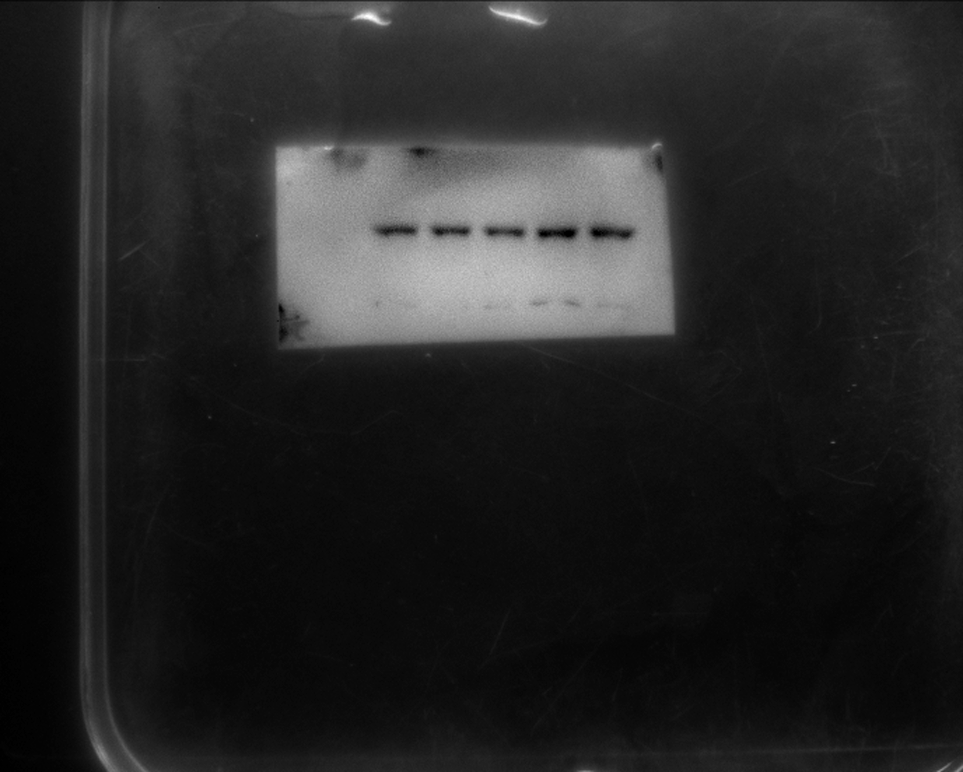

Supplement: Supplementary file 5 [file DataSheet9.ZIP › 3/muc5ac tublin.tif]

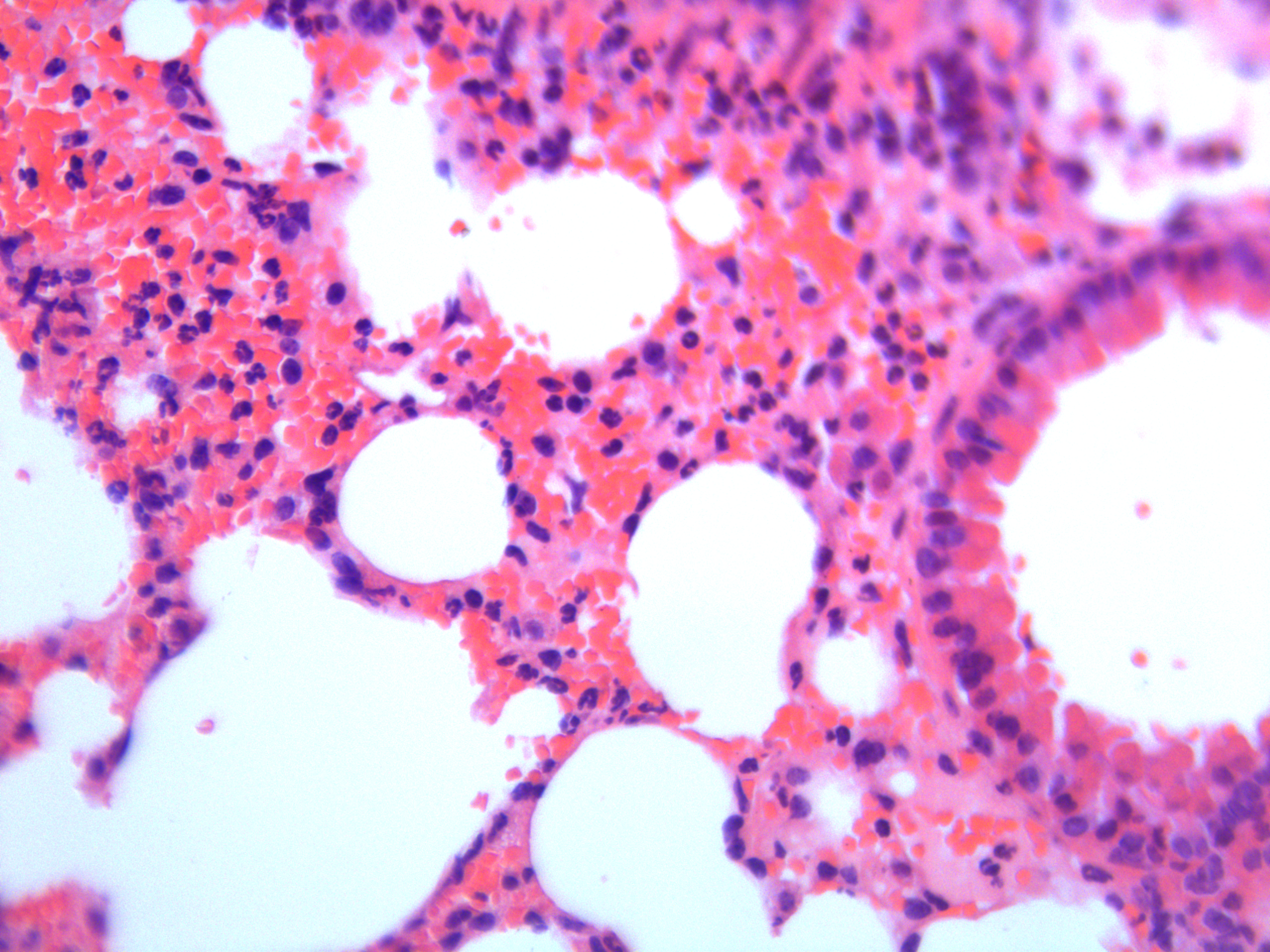

Supplement: Supplementary file 6 [file Image6.TIF]

## Slide 1
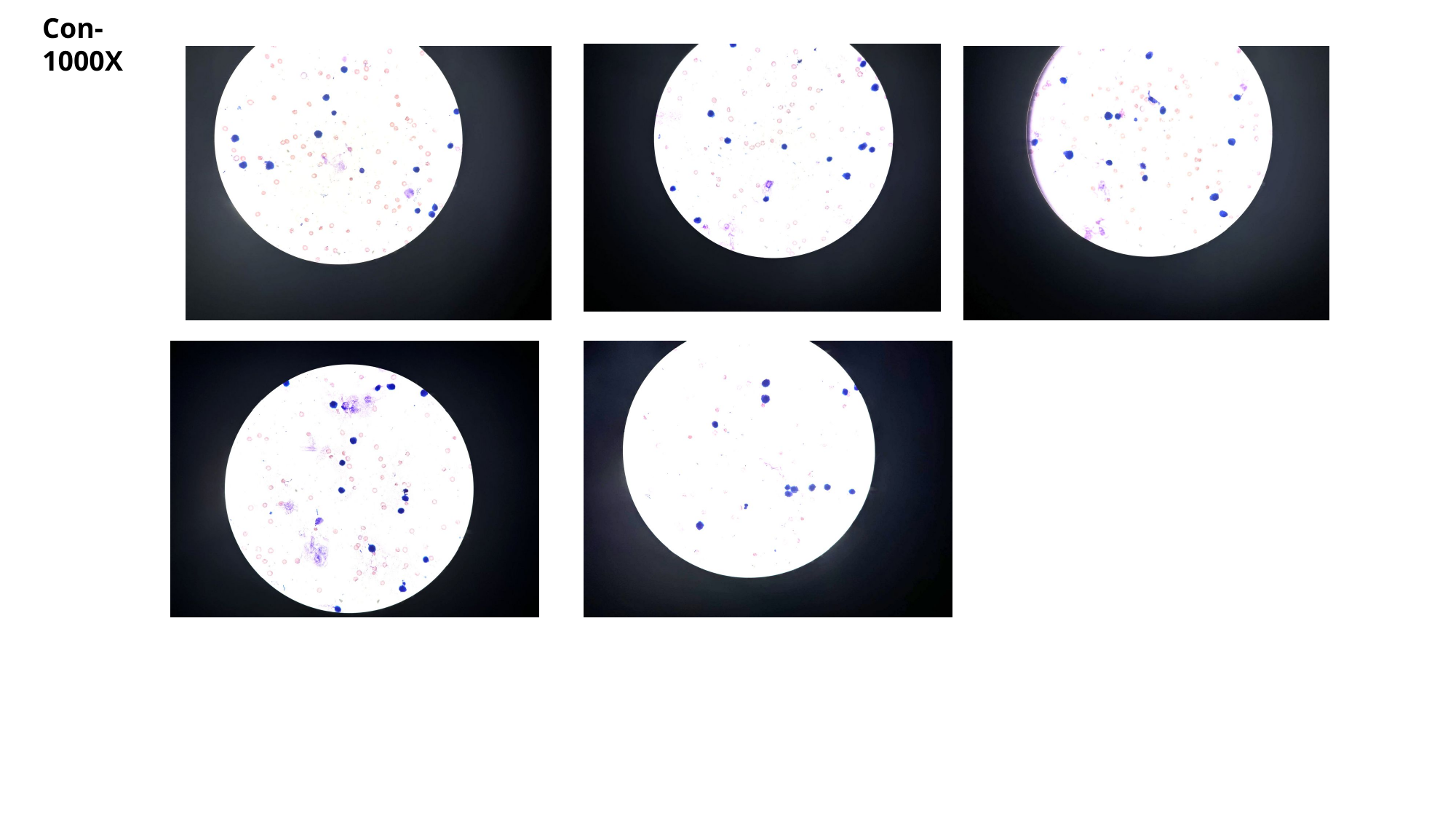

Con-1000X

## Slide 2
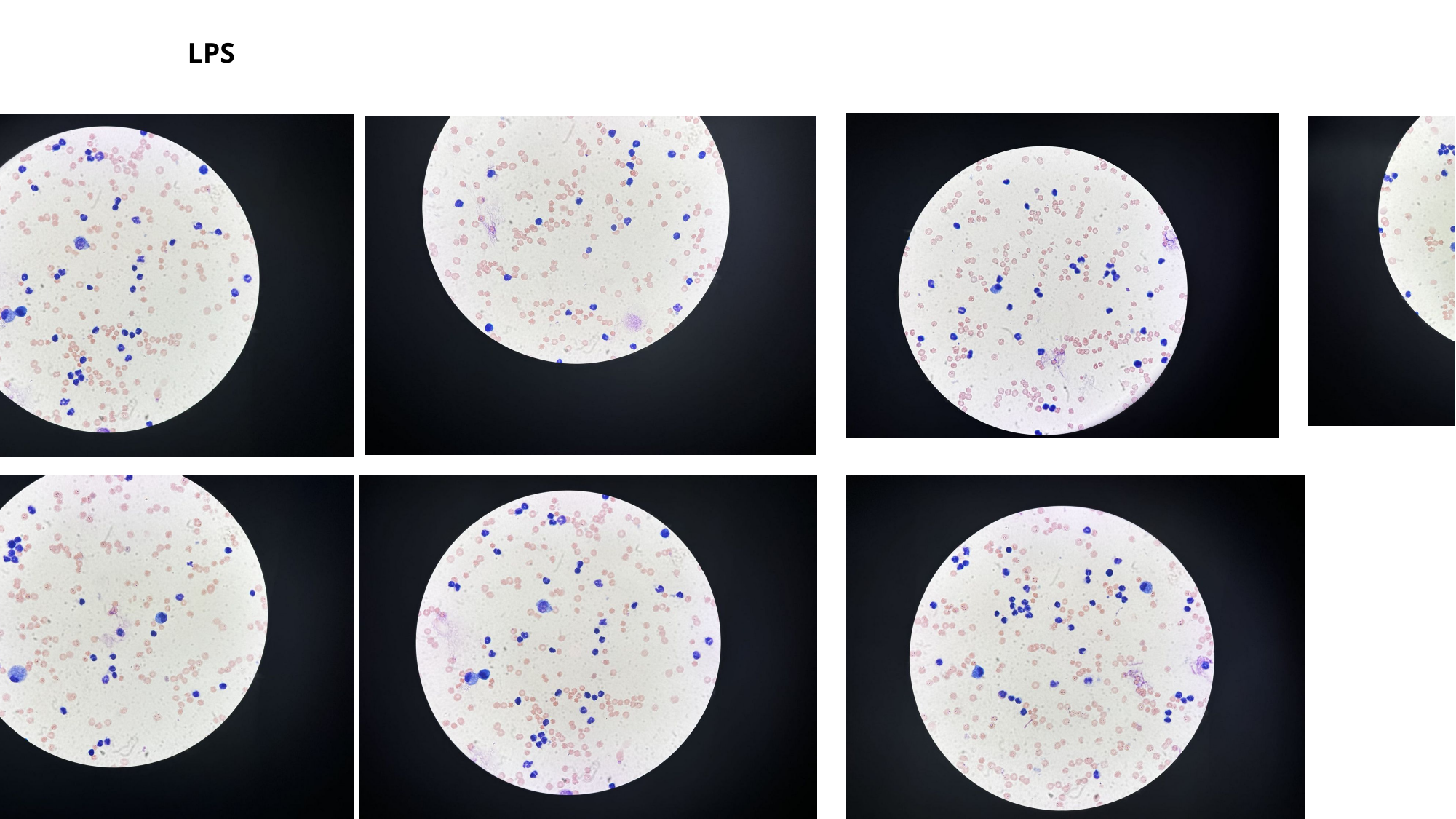

LPS

## Slide 3
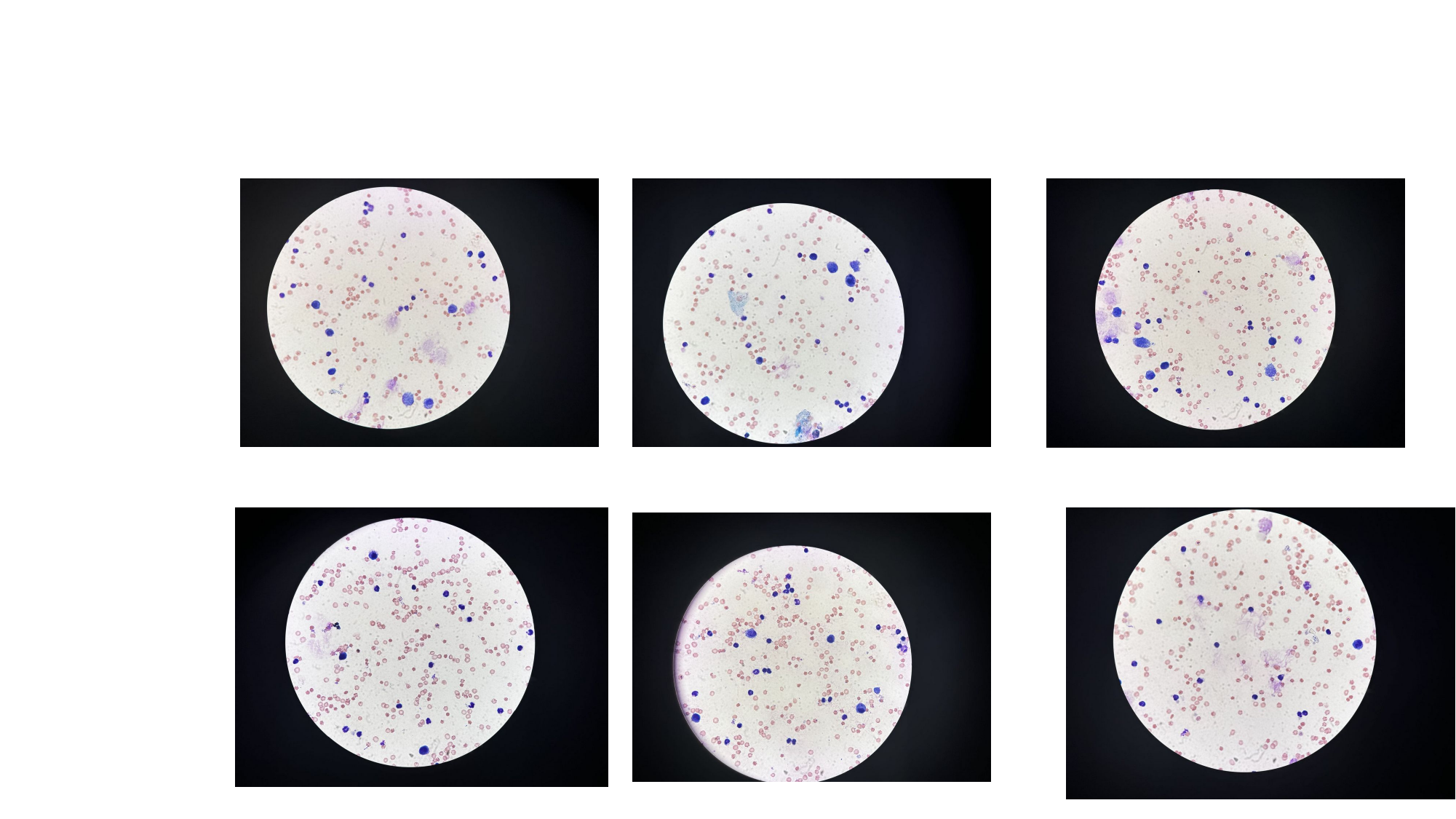

Val-Low

## Slide 4
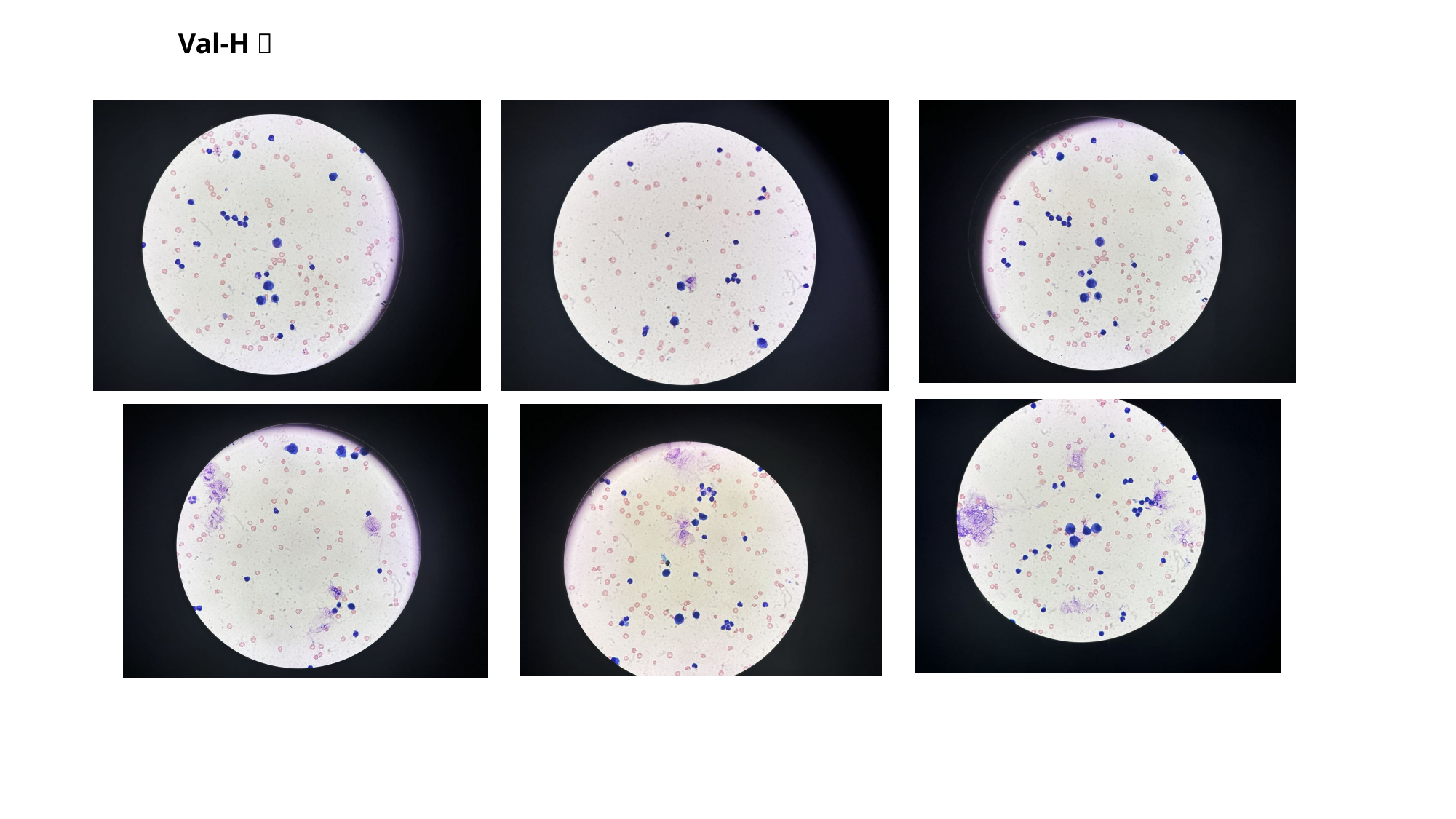

Val-H：

Supplement: Supplementary file 7 [file Presentation1.PPTX]

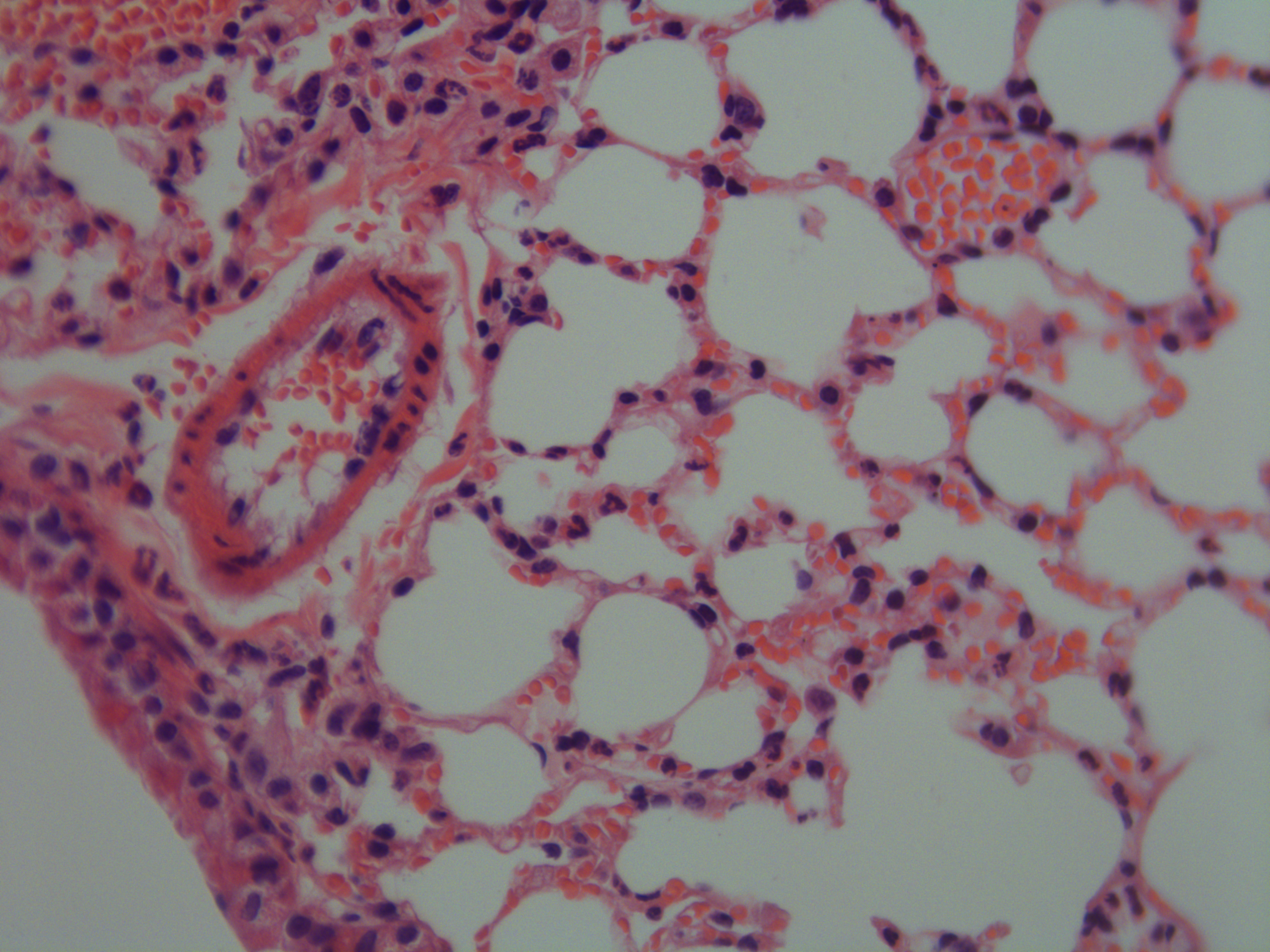

Supplement: Supplementary file 8 [file Image14.TIF]

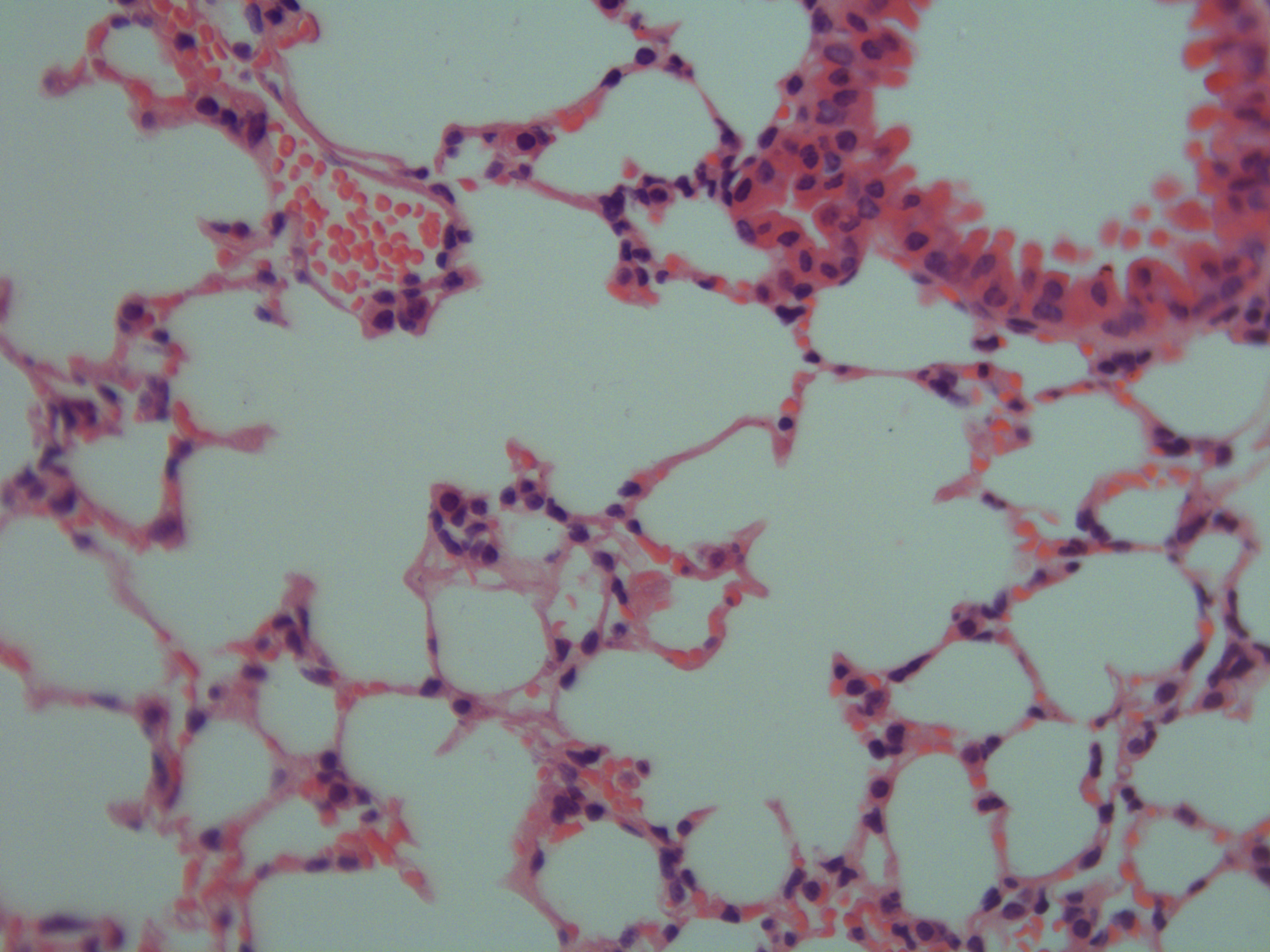

Supplement: Supplementary file 9 [file Image3.TIF]

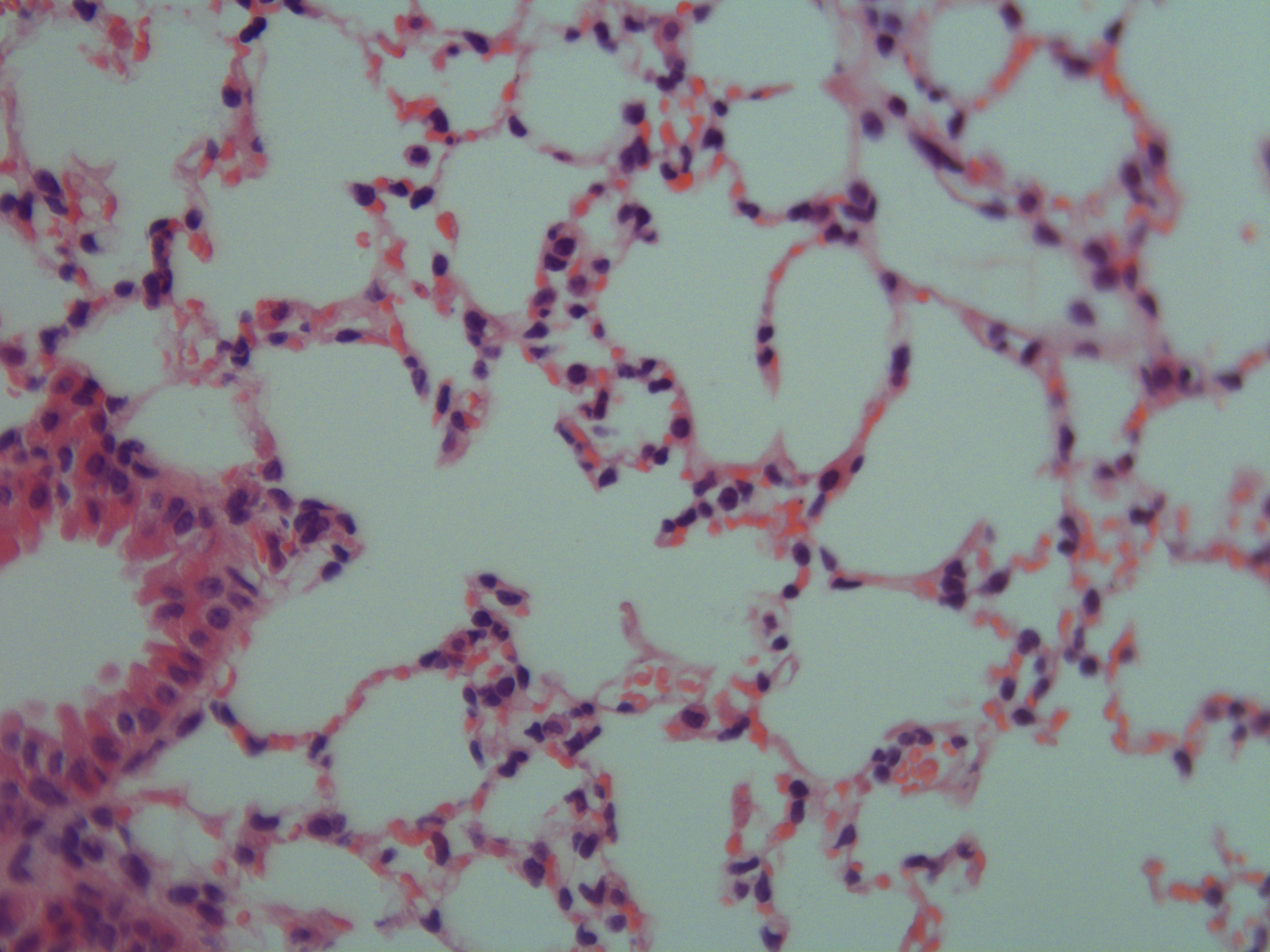

Supplement: Supplementary file 10 [file Image4.TIF]

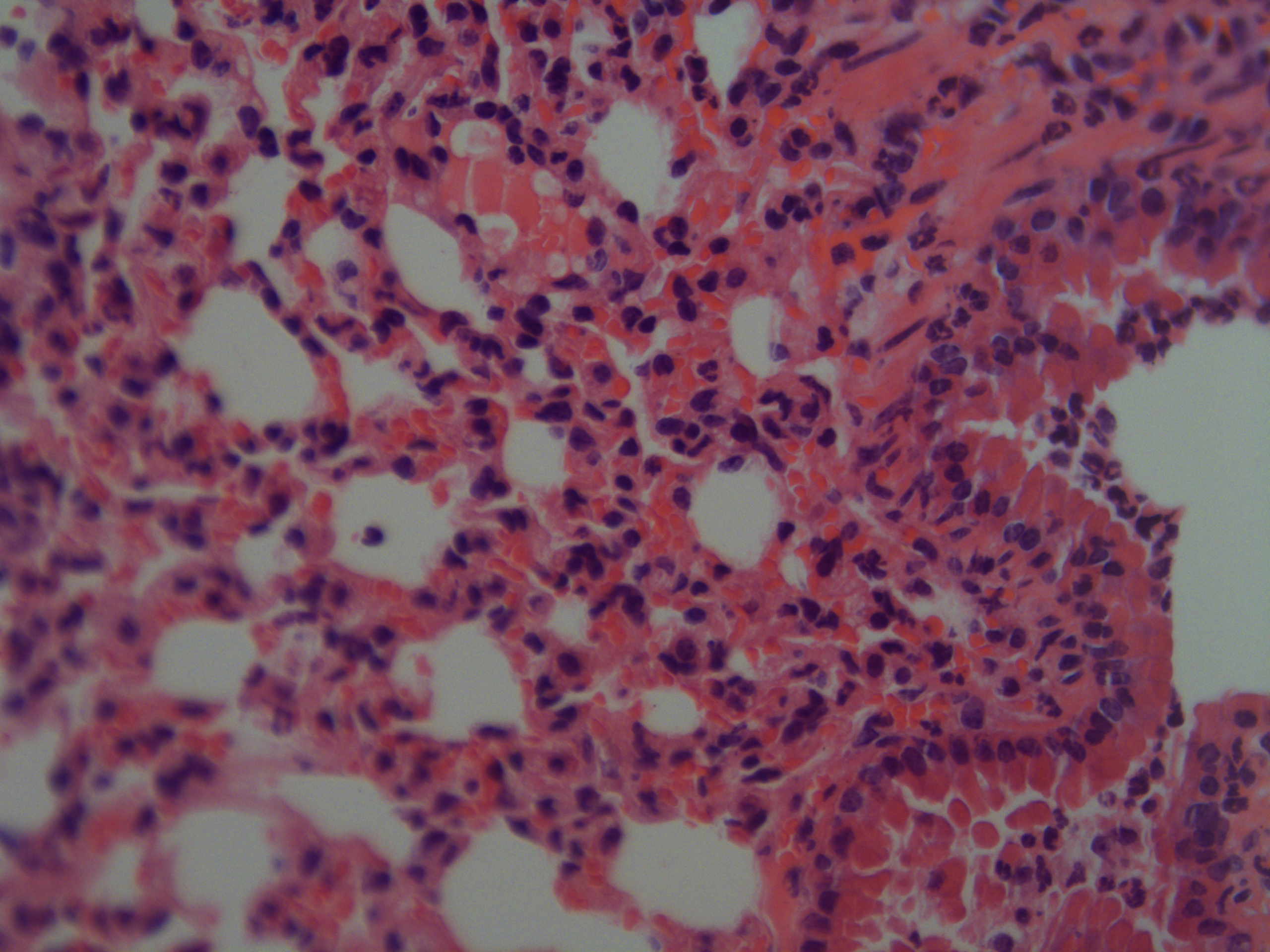

Supplement: Supplementary file 11 [file Image9.TIF]

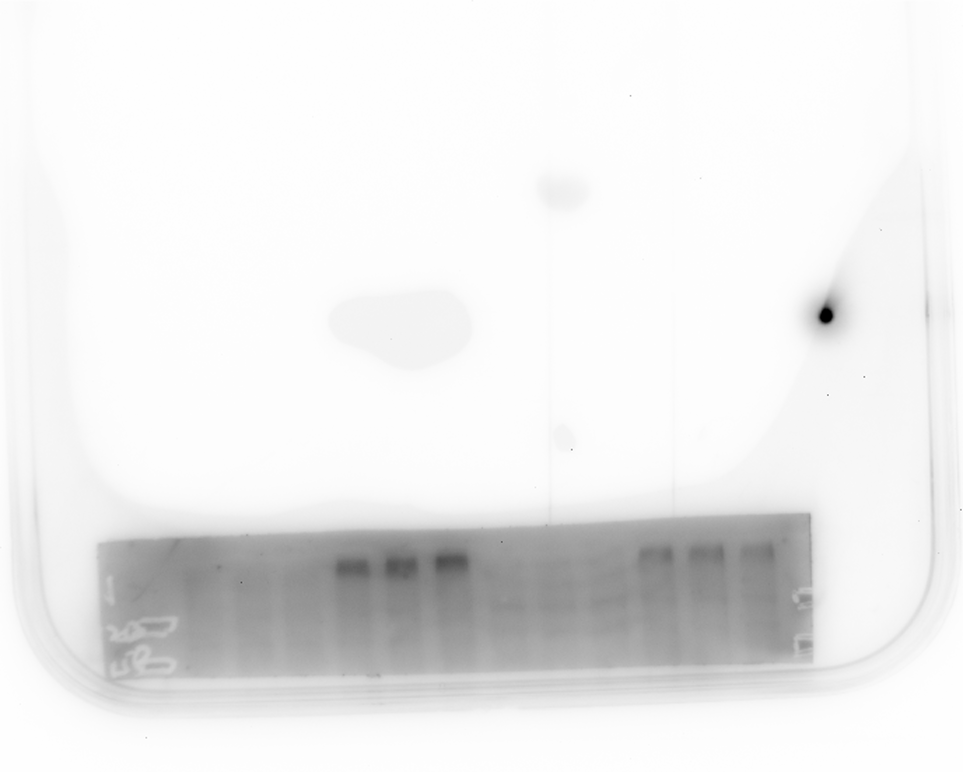

Supplement: Supplementary file 12 [file DataSheet4.ZIP › MUC5AC1/MUC5ac 1.tif]

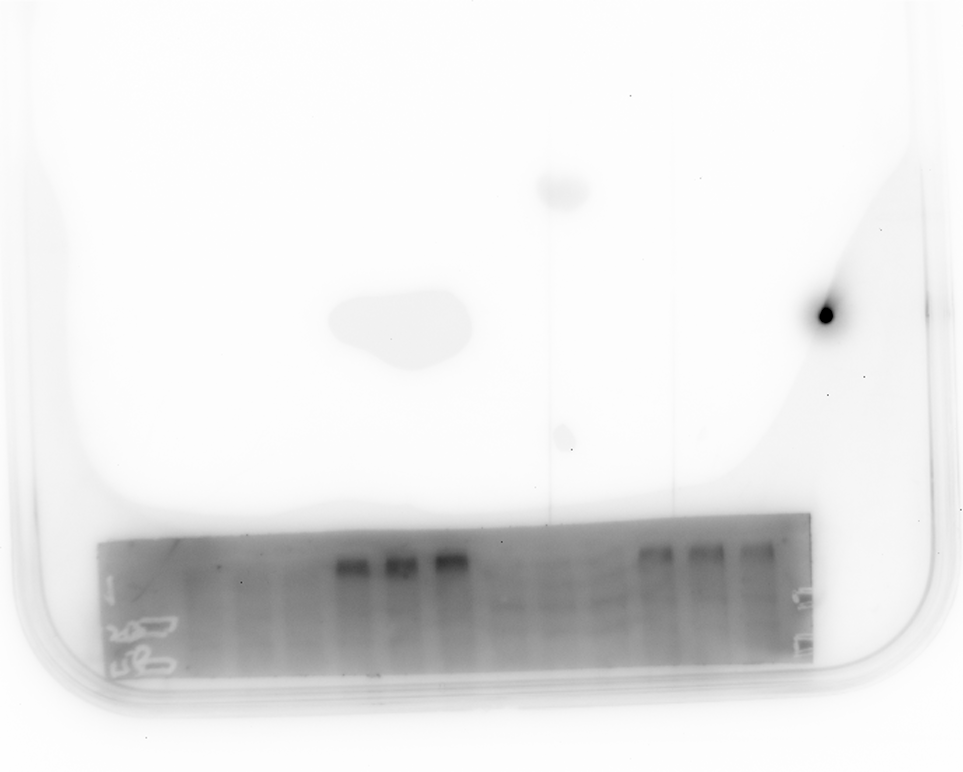

Supplement: Supplementary file 12 [file DataSheet4.ZIP › MUC5AC1/MUC5ac 2.tif]

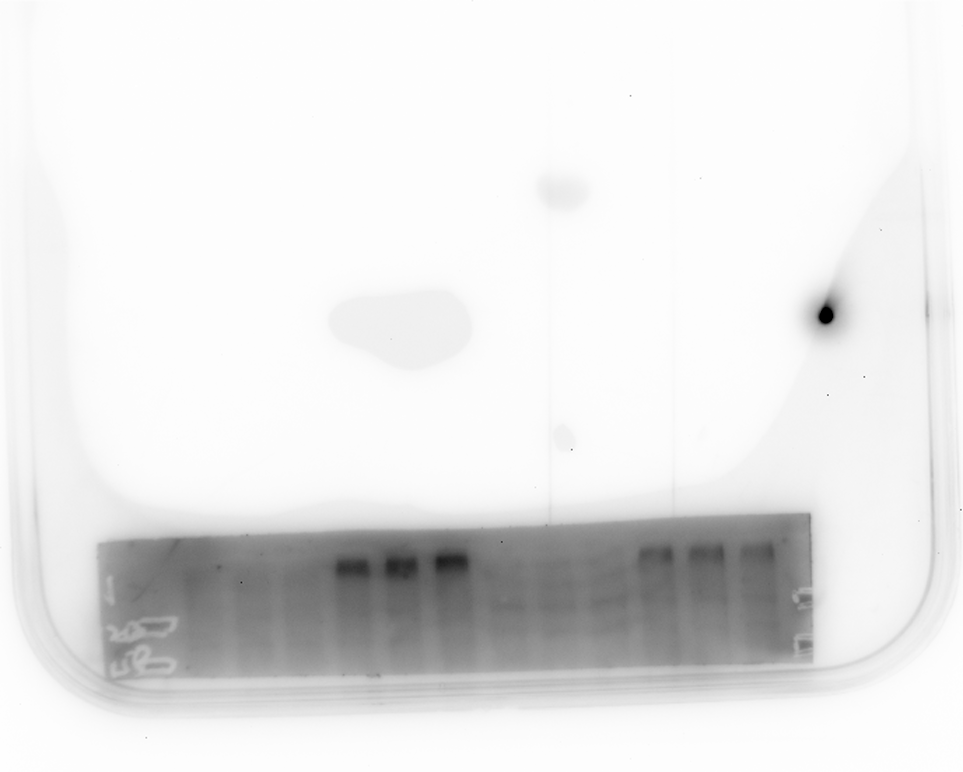

Supplement: Supplementary file 12 [file DataSheet4.ZIP › MUC5AC1/MUC5ac 3.tif]

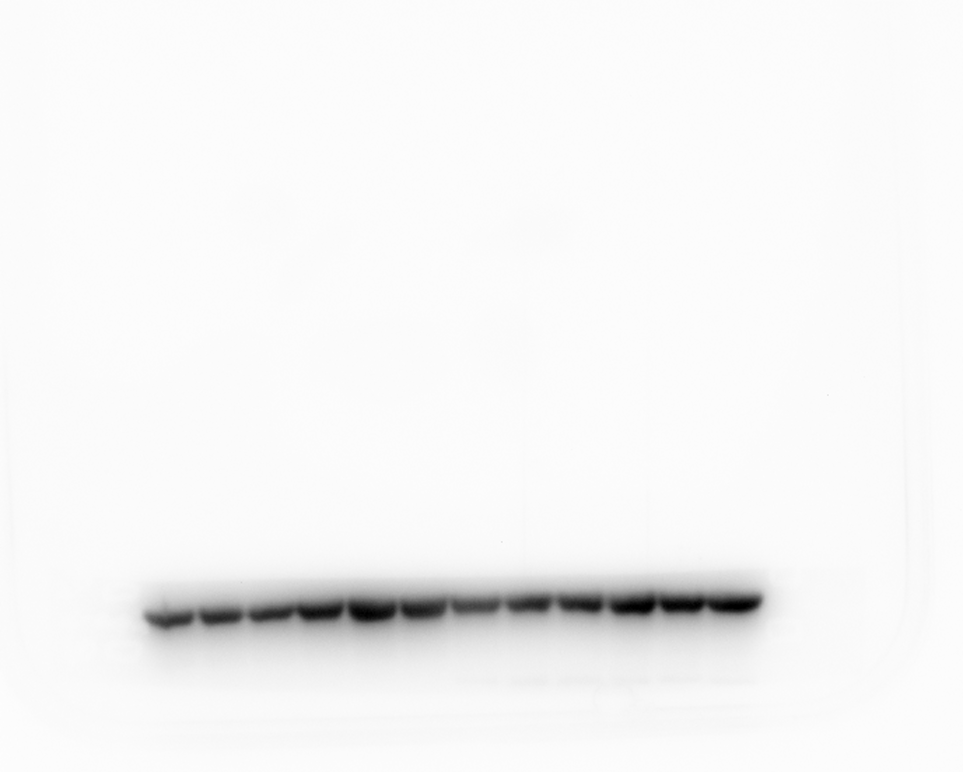

Supplement: Supplementary file 12 [file DataSheet4.ZIP › MUC5AC1/MUC5ac b-actin 2.tif]

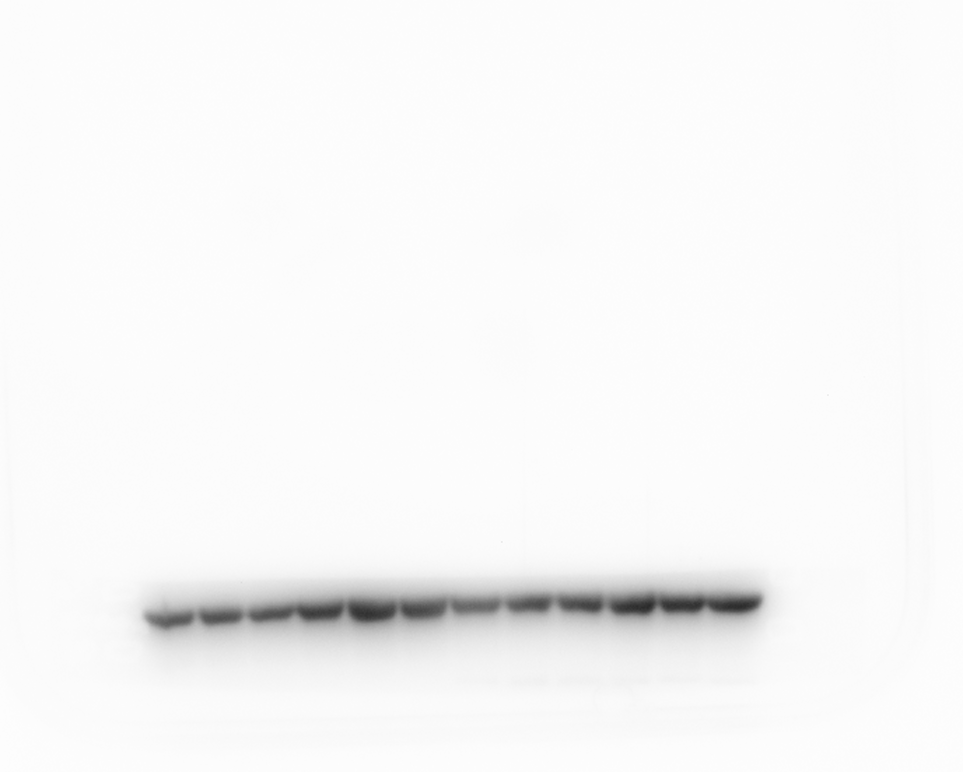

Supplement: Supplementary file 12 [file DataSheet4.ZIP › MUC5AC1/MUC5ac b-actin 3.tif]

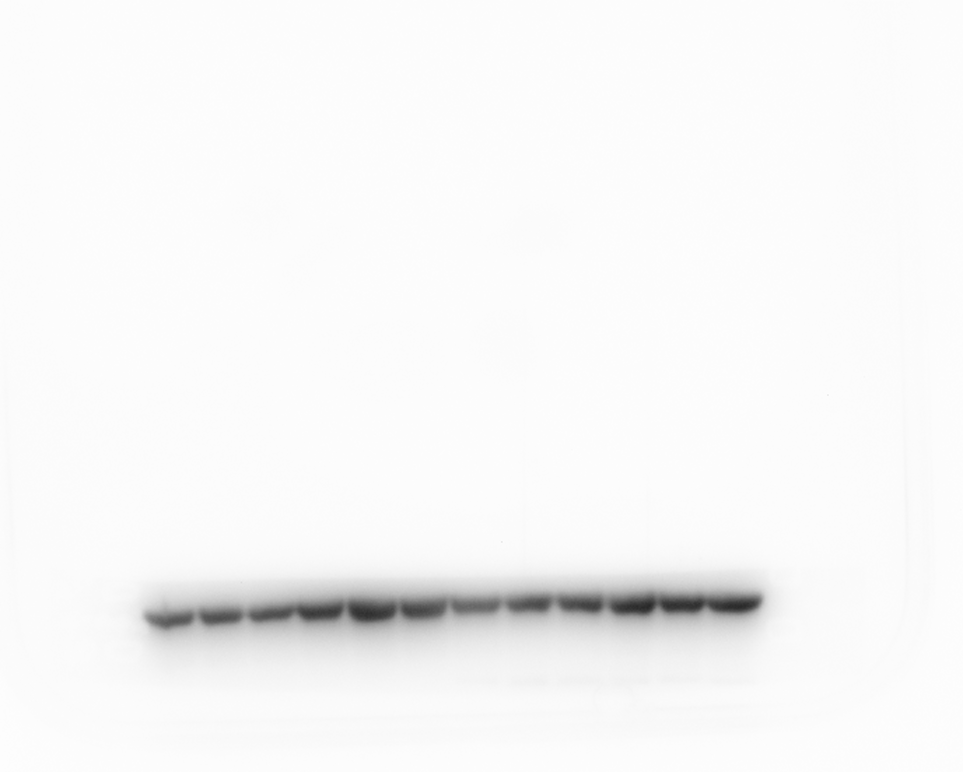

Supplement: Supplementary file 12 [file DataSheet4.ZIP › MUC5AC1/MUC5ac b-actin 4.tif]

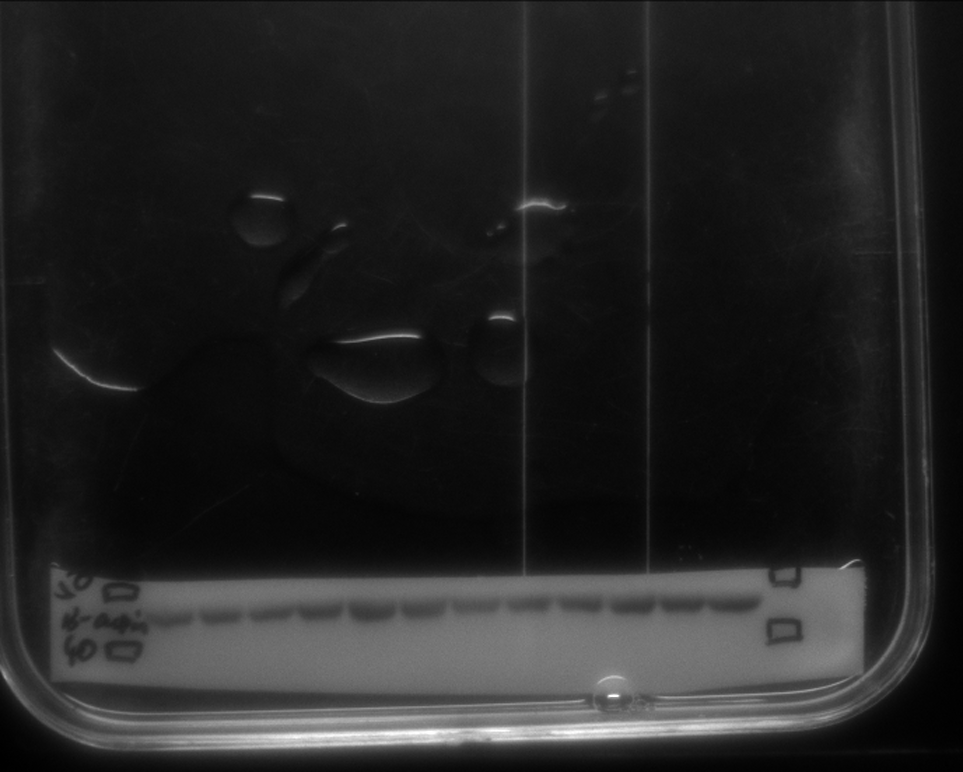

Supplement: Supplementary file 12 [file DataSheet4.ZIP › MUC5AC1/MUC5ac b-actin q.tif]

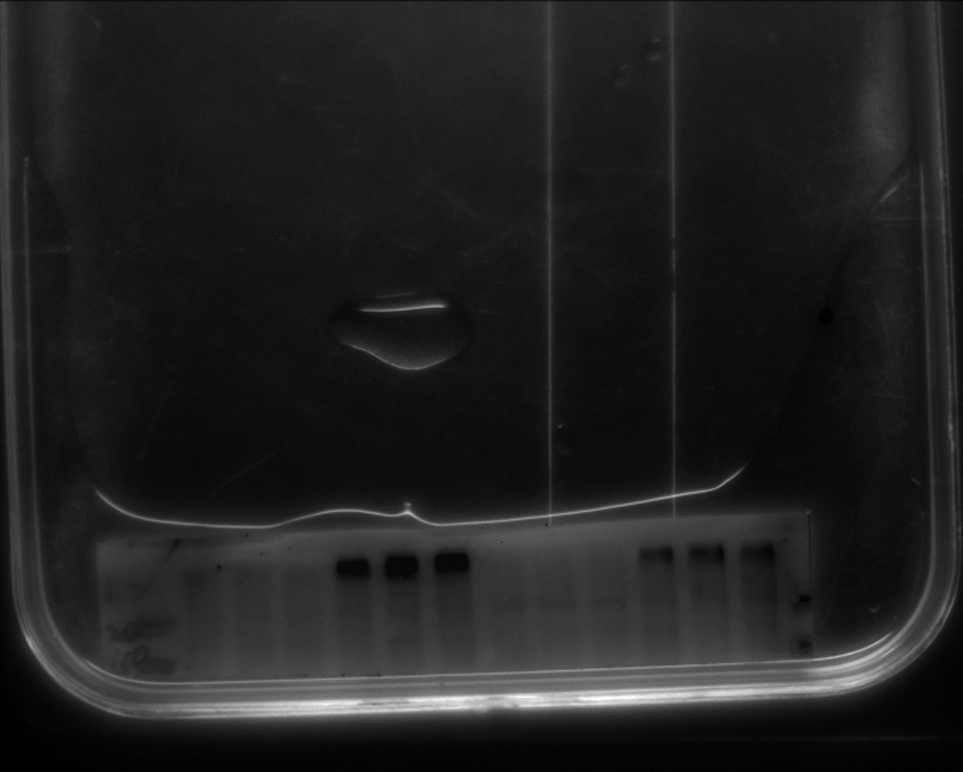

Supplement: Supplementary file 12 [file DataSheet4.ZIP › MUC5AC1/MUC5ac gb.tif]

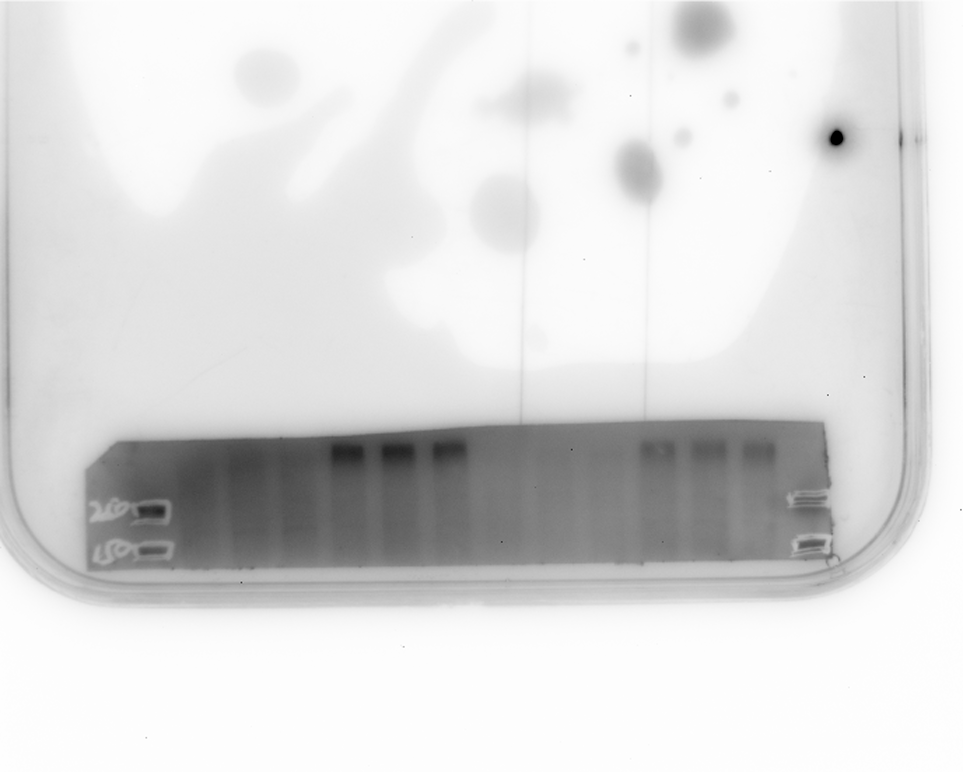

Supplement: Supplementary file 12 [file DataSheet4.ZIP › MUC5AC2/MUC5ac 1.tif]

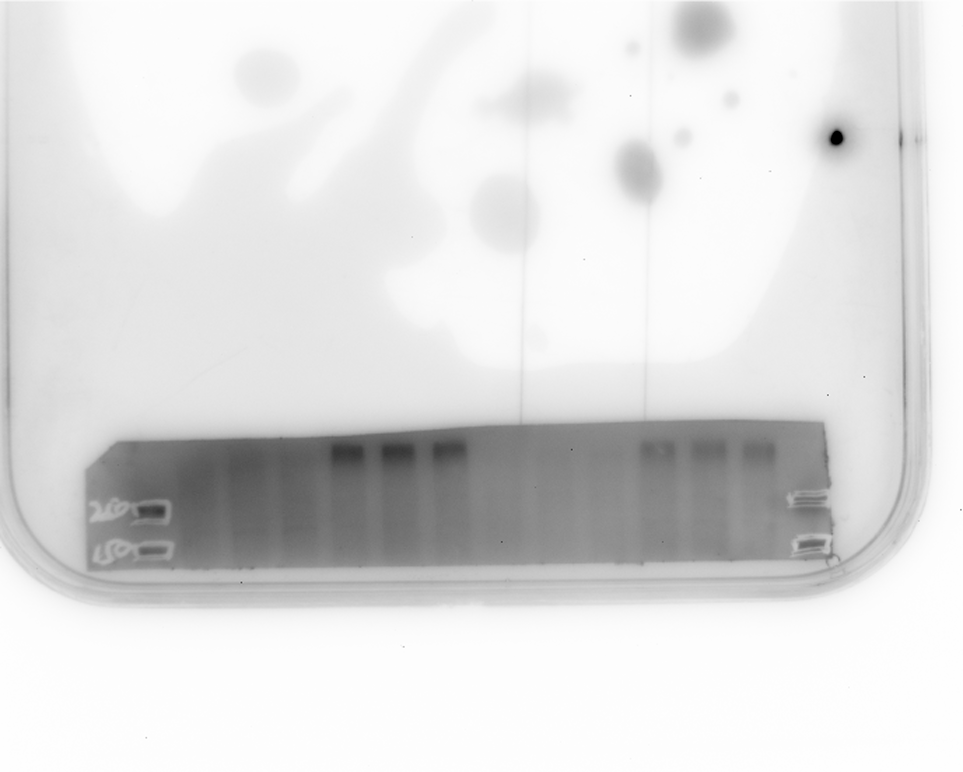

Supplement: Supplementary file 12 [file DataSheet4.ZIP › MUC5AC2/MUC5ac 2.tif]

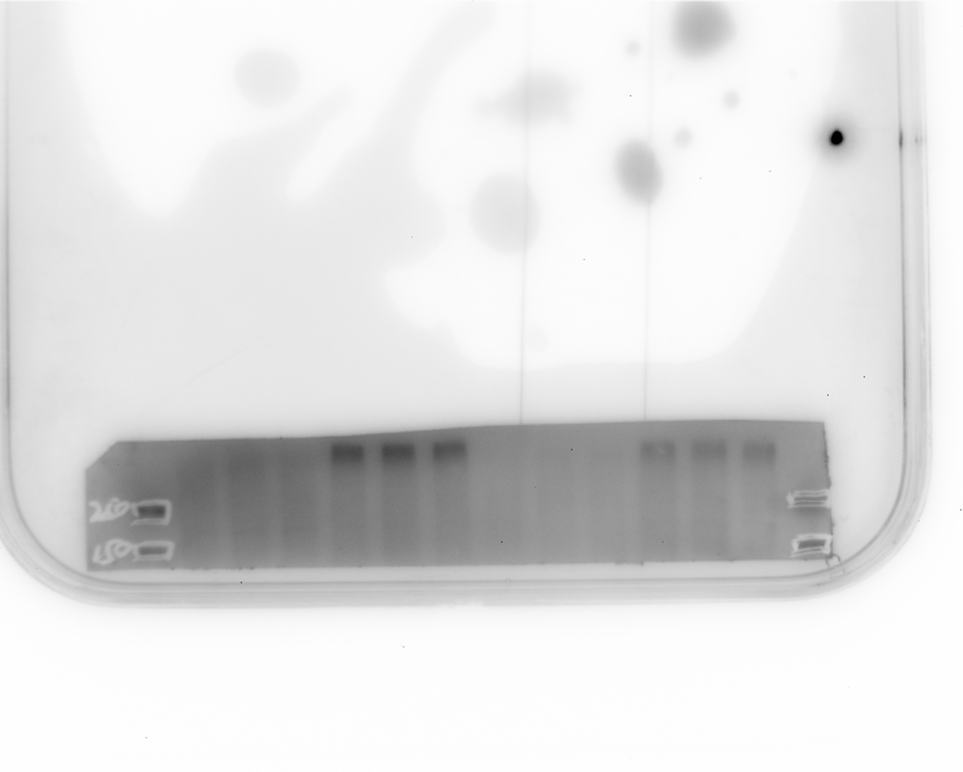

Supplement: Supplementary file 12 [file DataSheet4.ZIP › MUC5AC2/MUC5ac 3.tif]

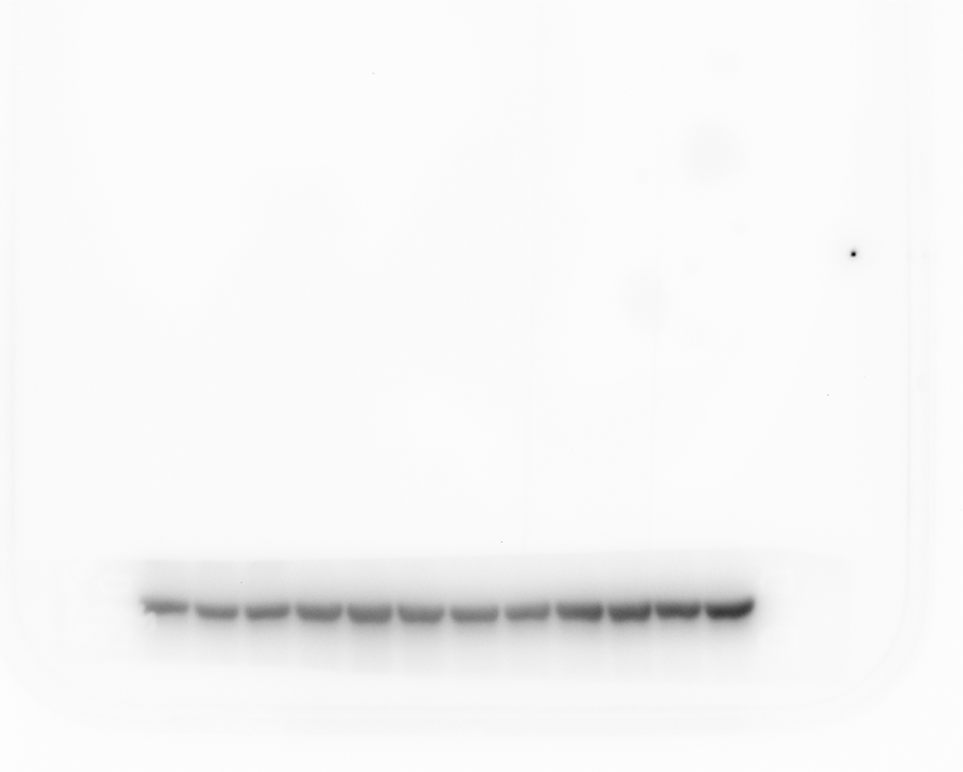

Supplement: Supplementary file 12 [file DataSheet4.ZIP › MUC5AC2/MUC5ac b-actin 1.tif]

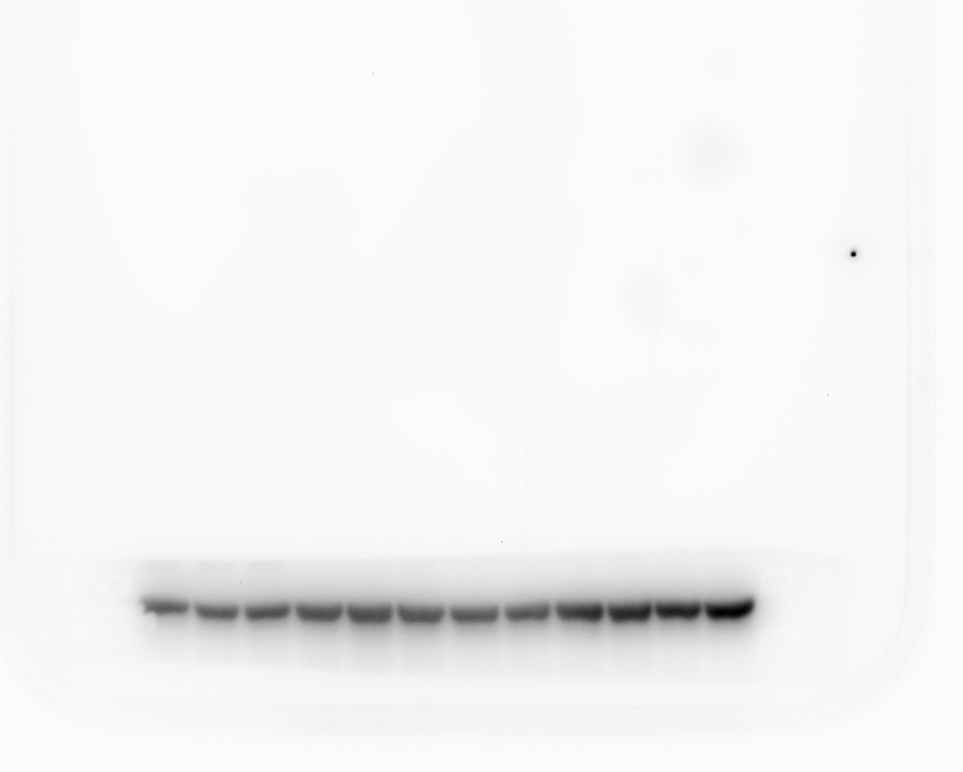

Supplement: Supplementary file 12 [file DataSheet4.ZIP › MUC5AC2/MUC5ac b-actin 2.tif]

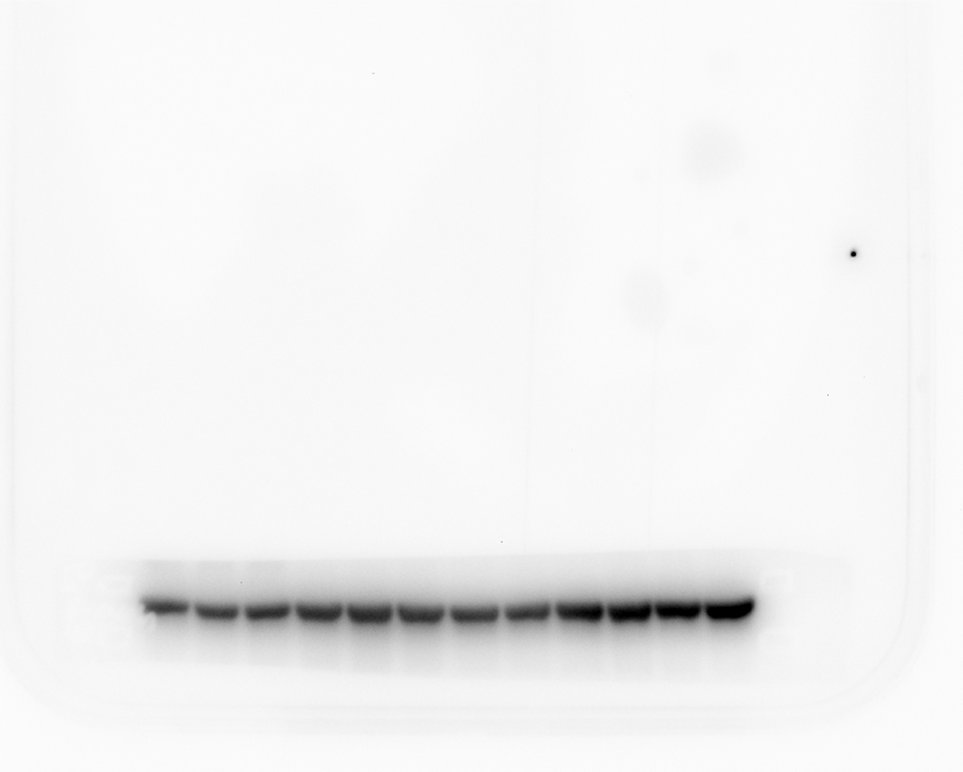

Supplement: Supplementary file 12 [file DataSheet4.ZIP › MUC5AC2/MUC5ac b-actin 3.tif]

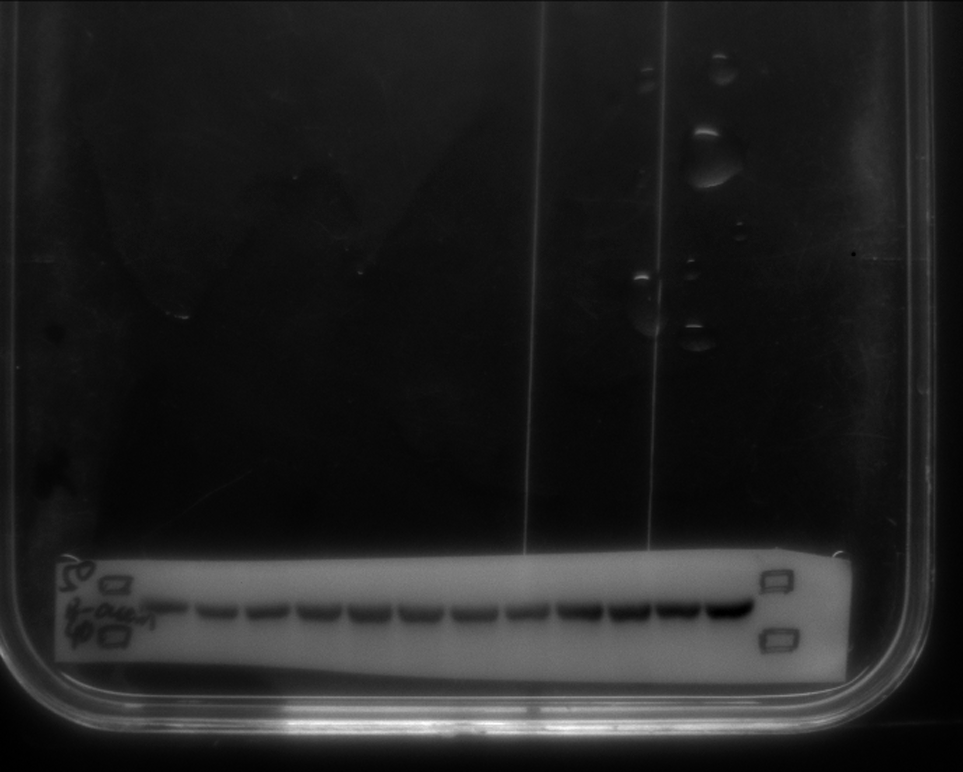

Supplement: Supplementary file 12 [file DataSheet4.ZIP › MUC5AC2/MUC5ac b-actin.tif]

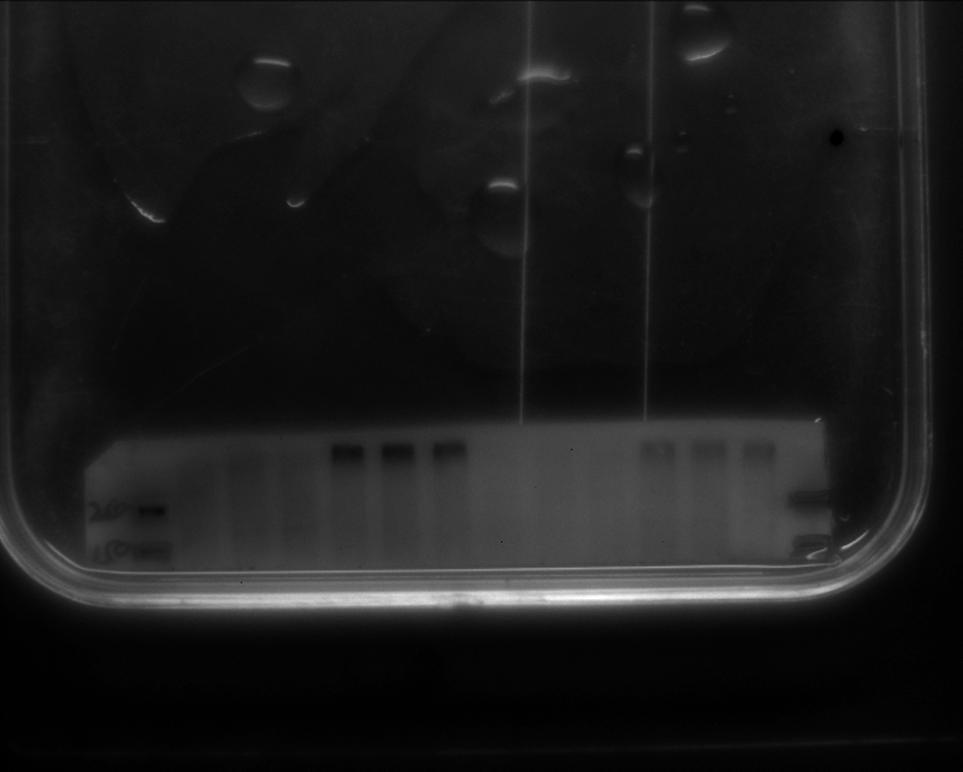

Supplement: Supplementary file 12 [file DataSheet4.ZIP › MUC5AC2/MUC5ac q.tif]

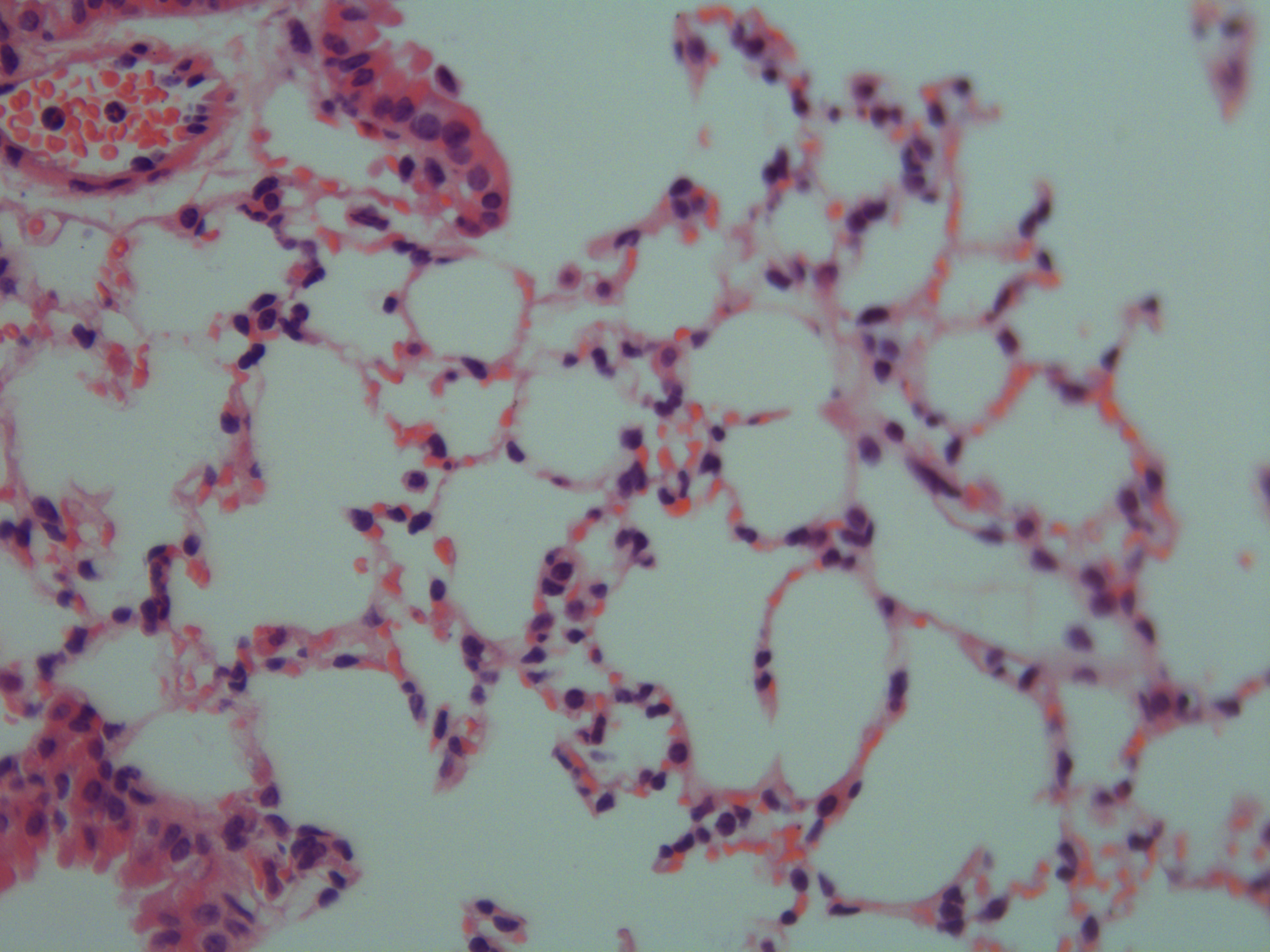

Supplement: Supplementary file 15 [file Image2.TIF]

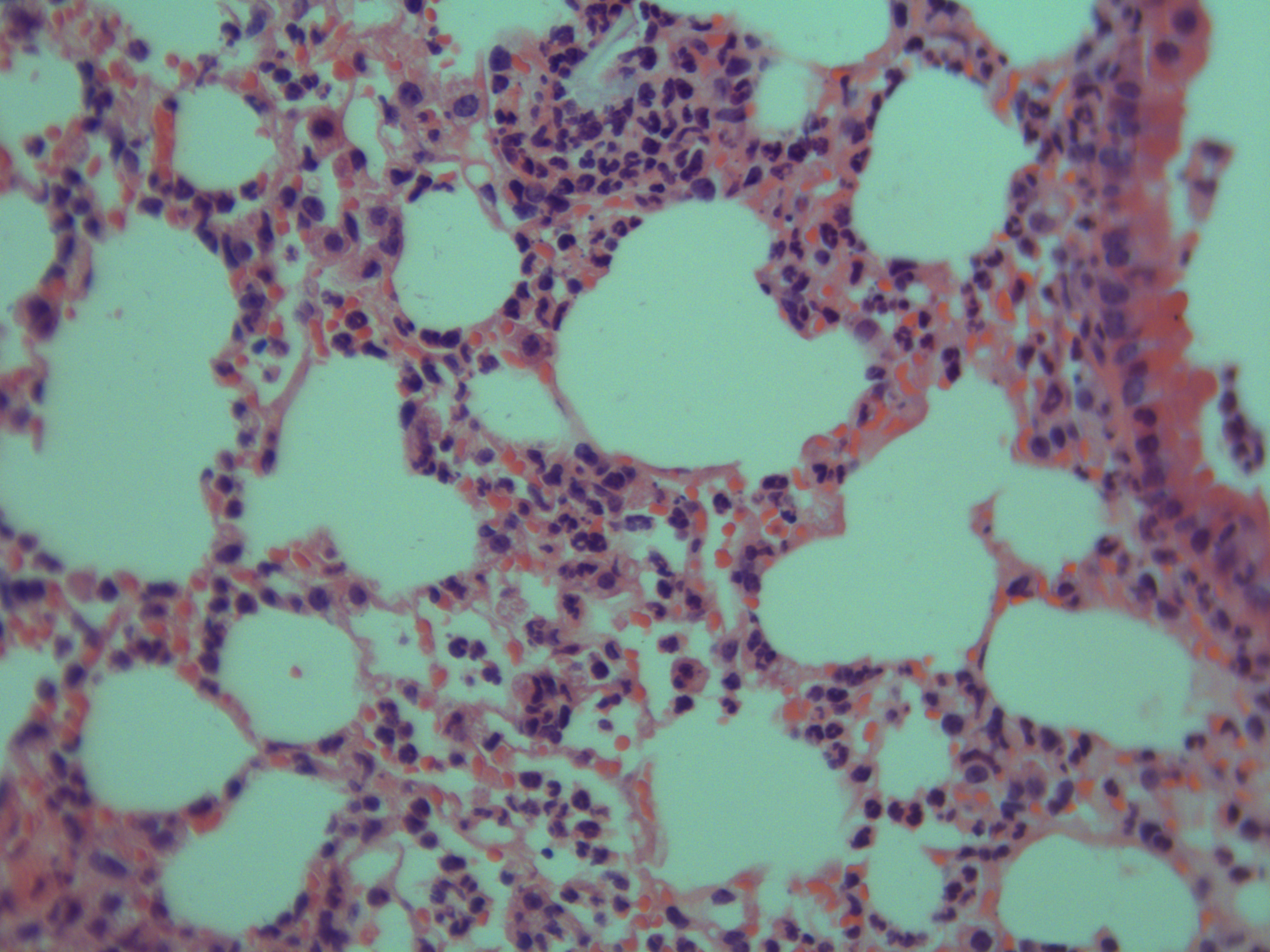

Supplement: Supplementary file 16 [file Image13.TIF]

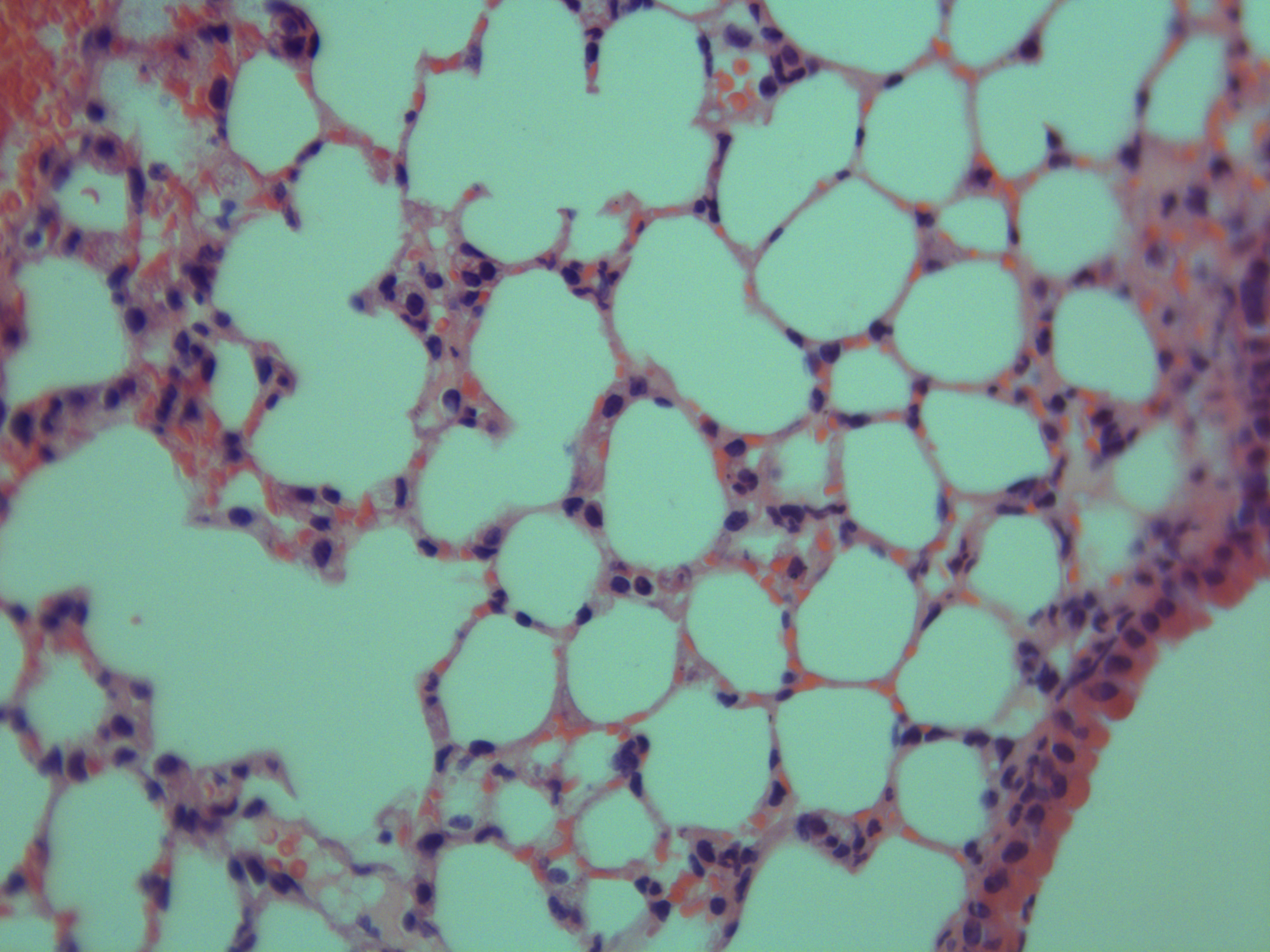

Supplement: Supplementary file 17 [file Image11.TIF]

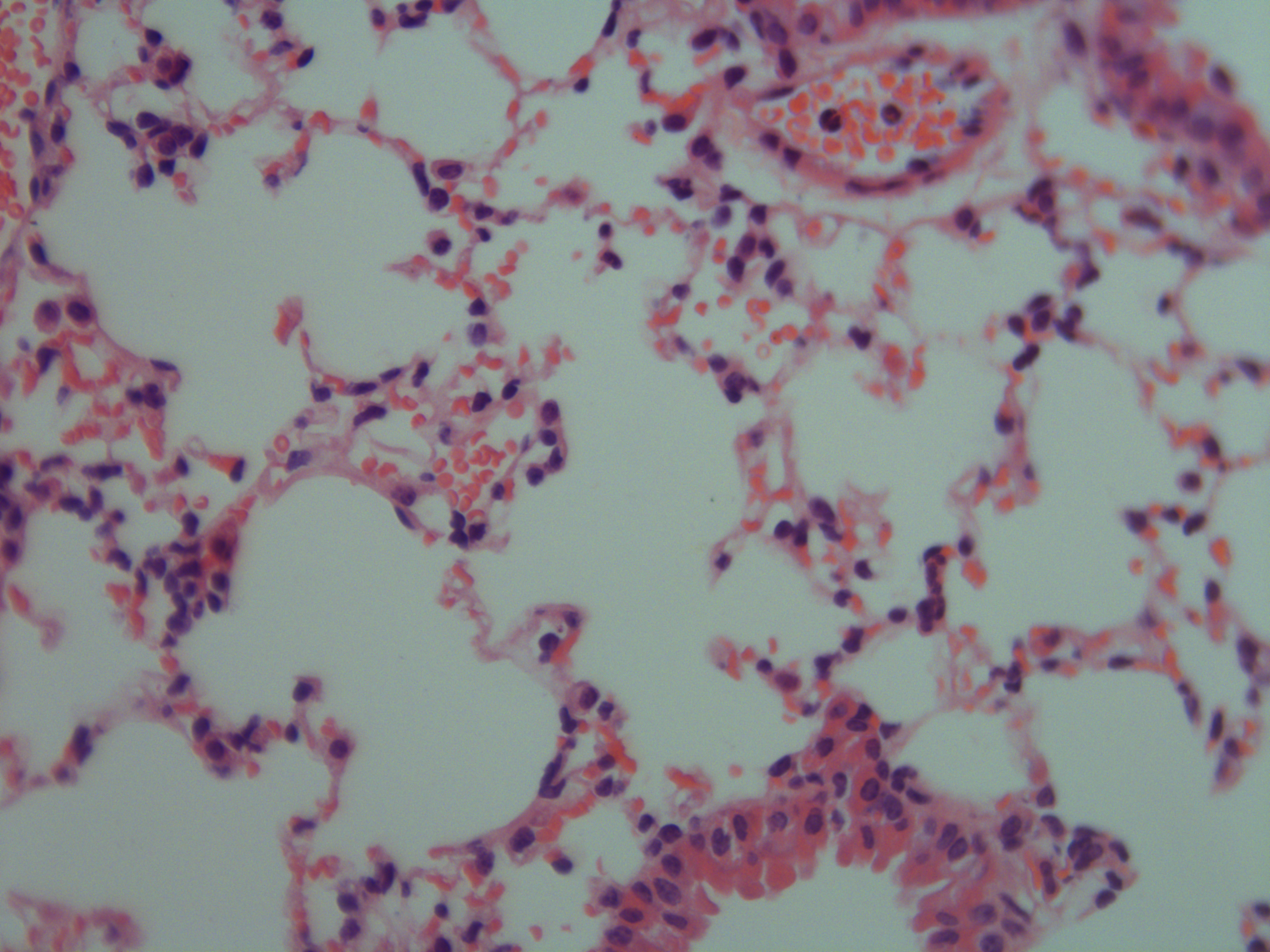

Supplement: Supplementary file 18 [file Image1.TIF]

## Slide 1
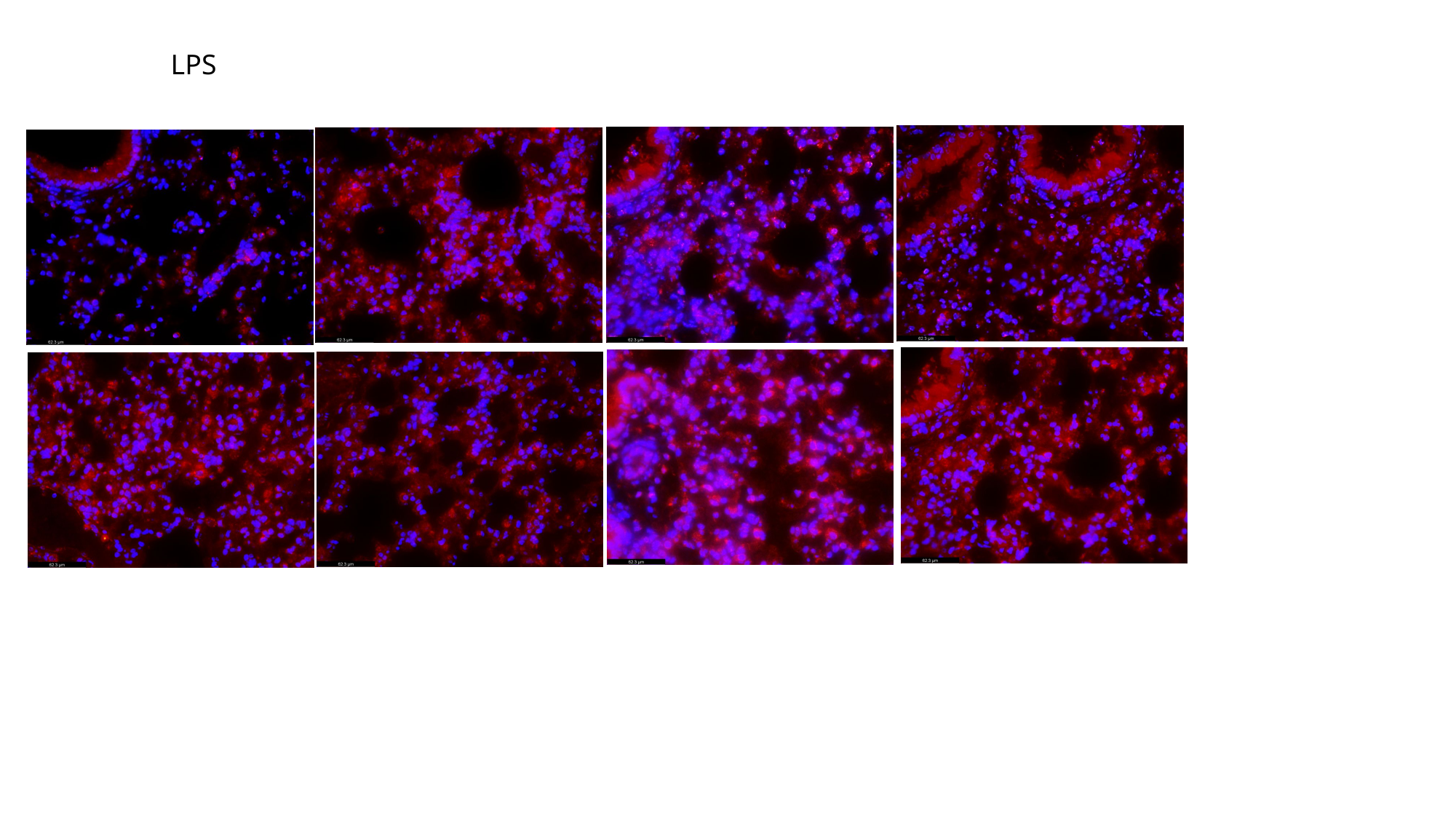

LPS

Supplement: Supplementary file 19 [file Presentation4.PPTX]

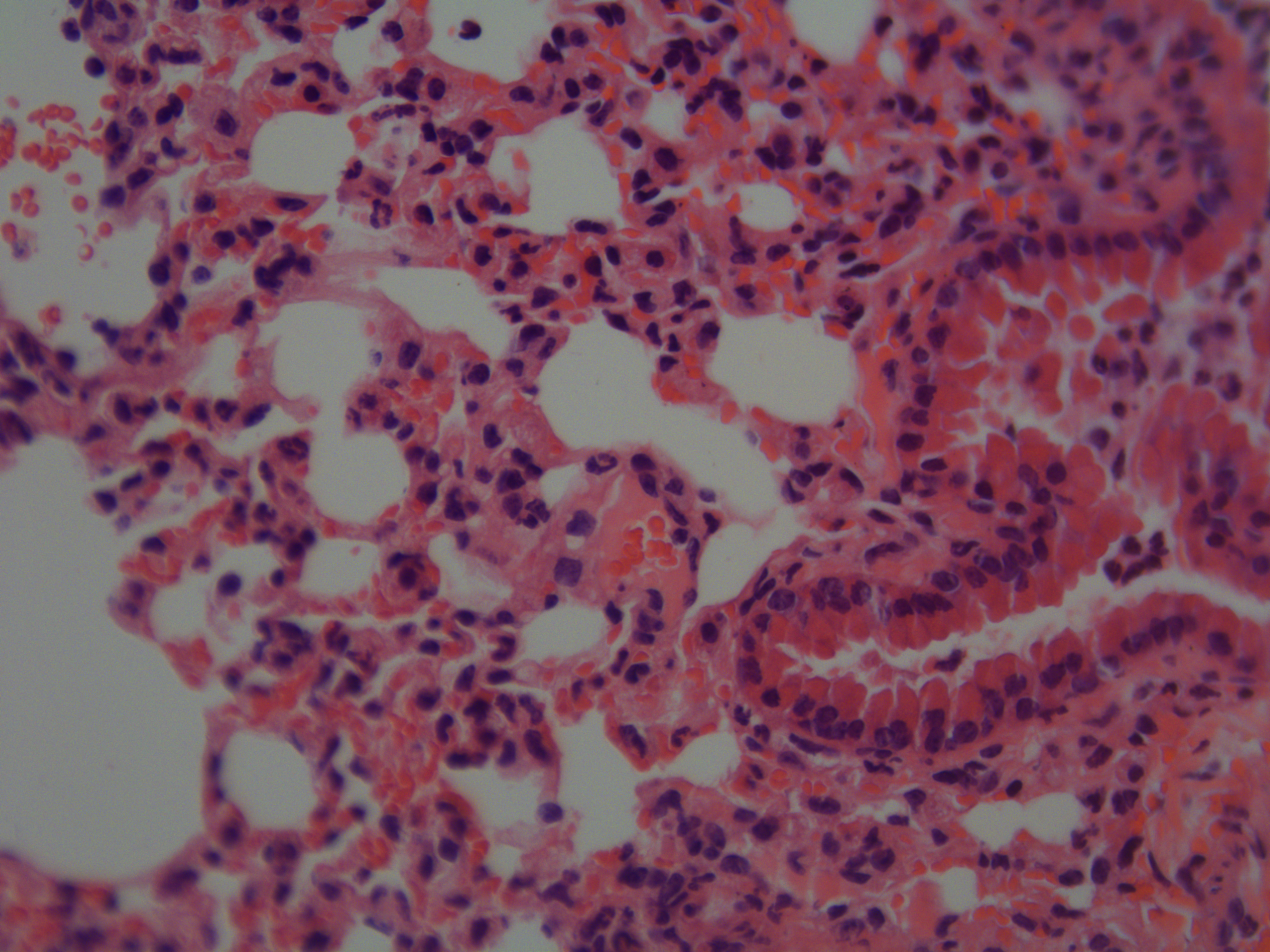

Supplement: Supplementary file 20 [file Image10.TIF]

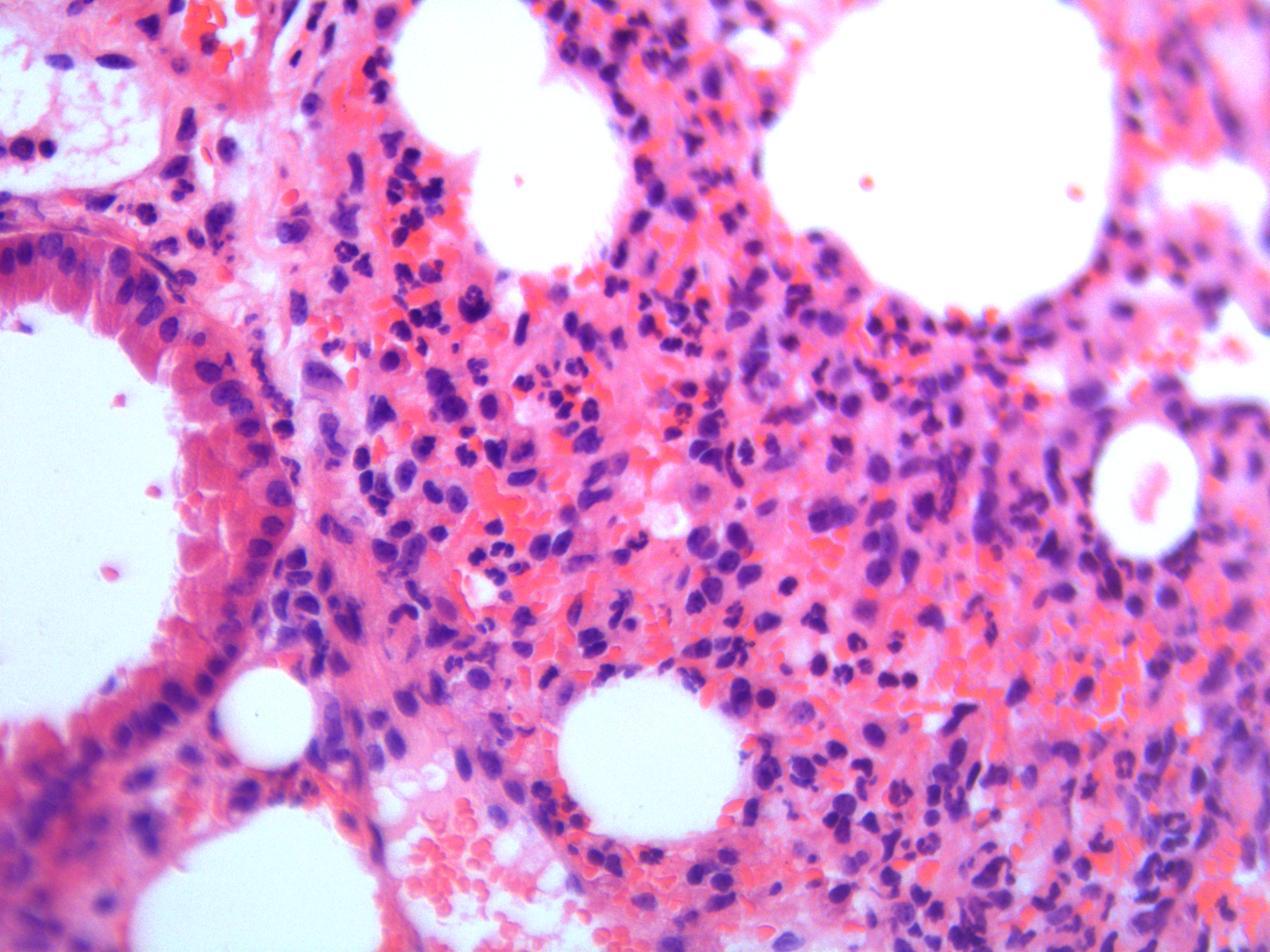

Supplement: Supplementary file 21 [file Image7.TIF]

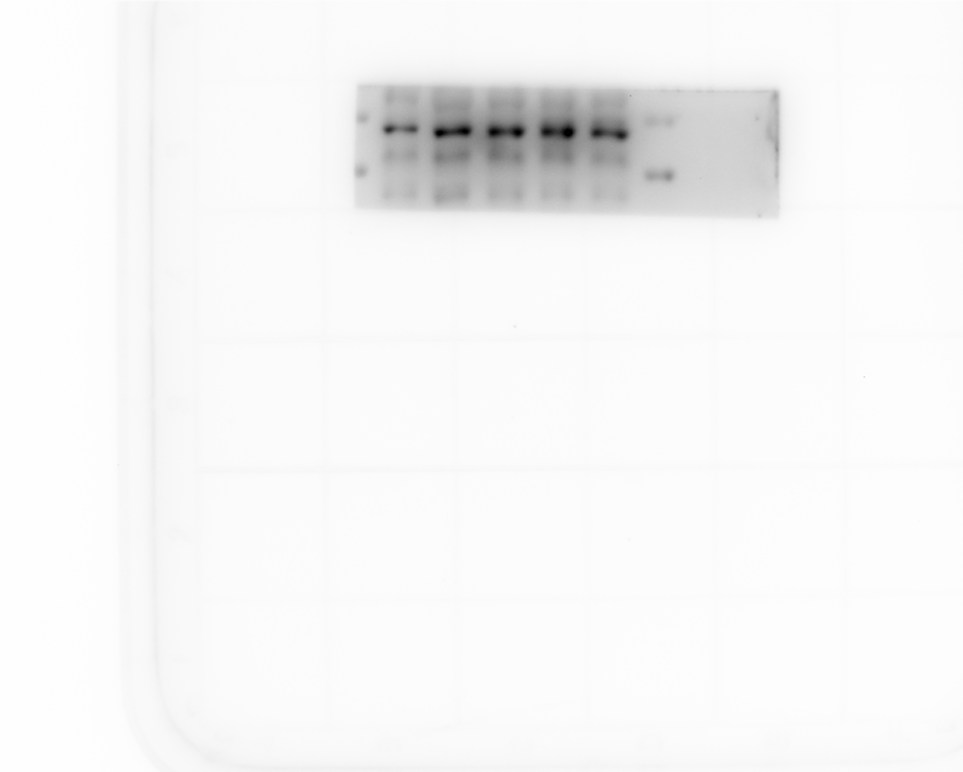

Supplement: Supplementary file 22 [file DataSheet10.ZIP › 2/p-p38 1.tif]

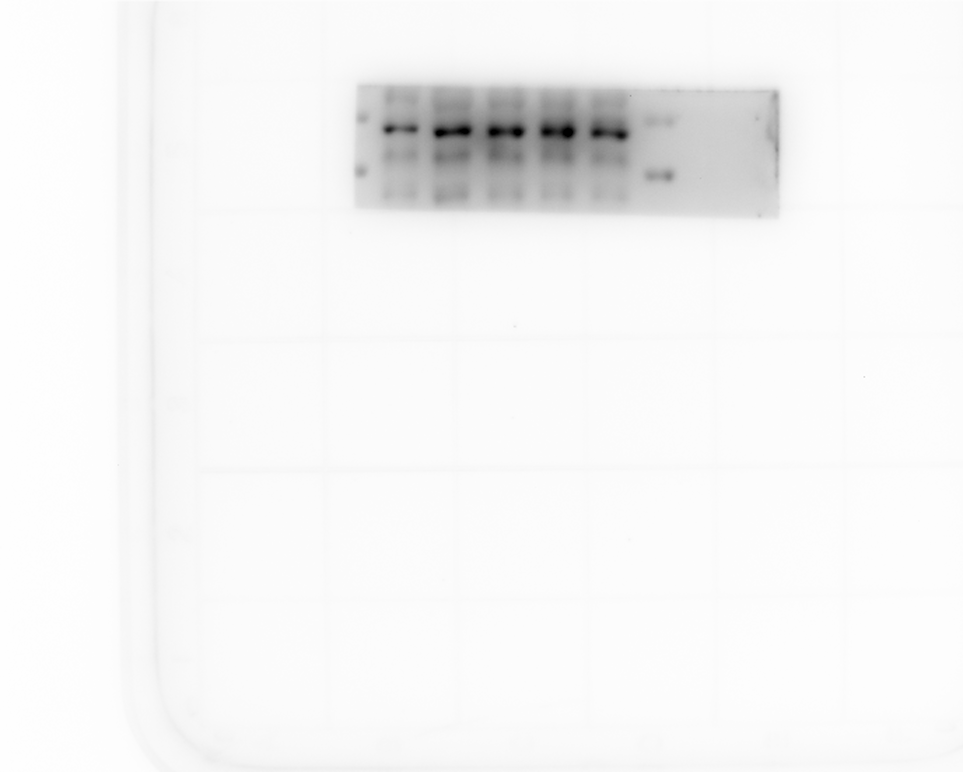

Supplement: Supplementary file 22 [file DataSheet10.ZIP › 2/p-p38 2.tif]

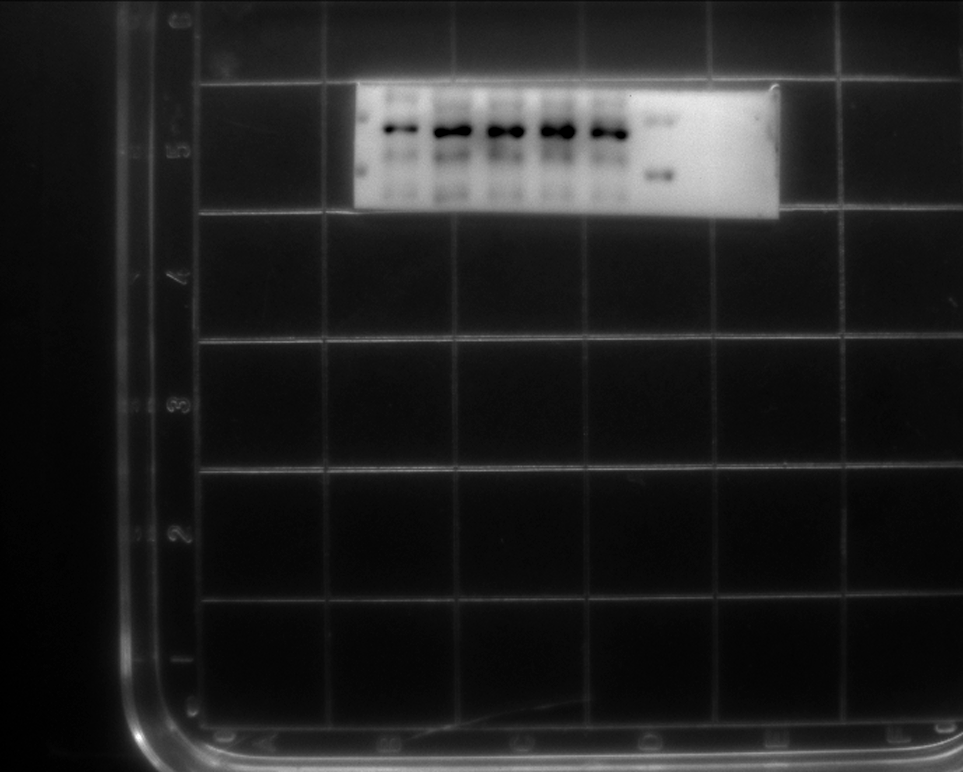

Supplement: Supplementary file 22 [file DataSheet10.ZIP › 2/p-p38 q.tif]

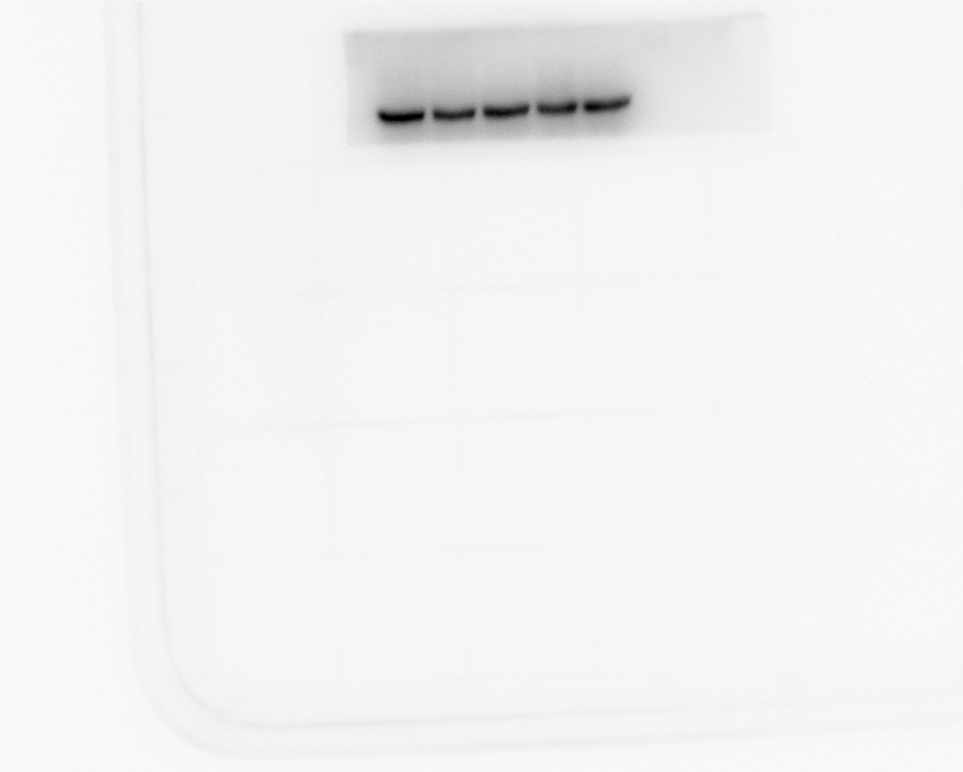

Supplement: Supplementary file 22 [file DataSheet10.ZIP › 2/p-p38 tublin 1.tif]

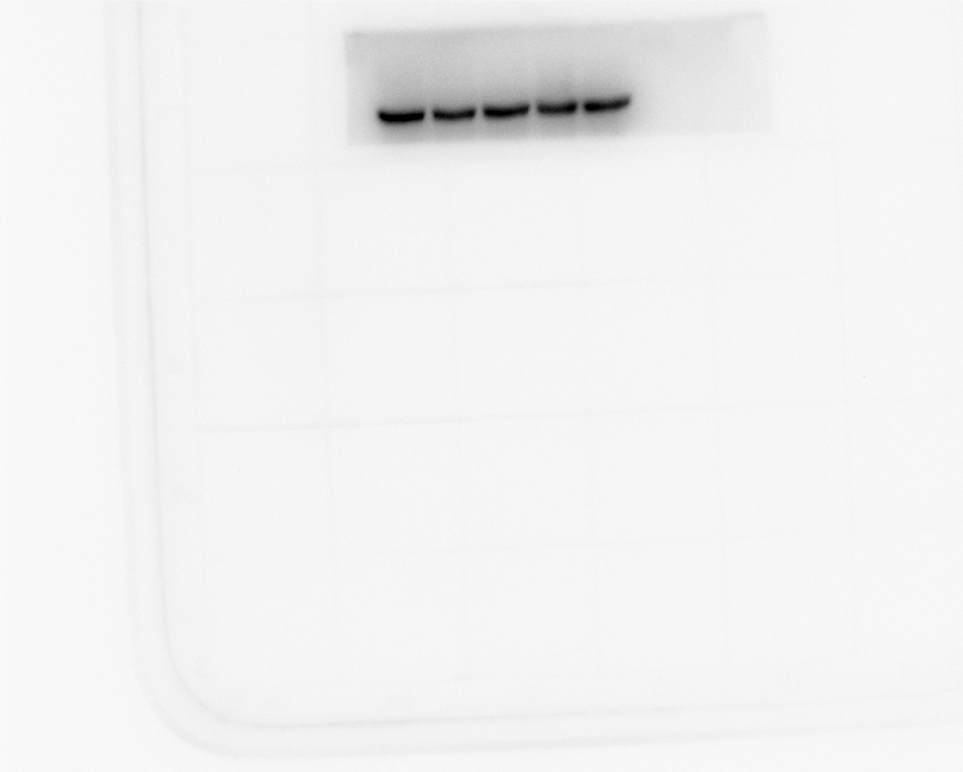

Supplement: Supplementary file 22 [file DataSheet10.ZIP › 2/p-p38 tublin 2.tif]

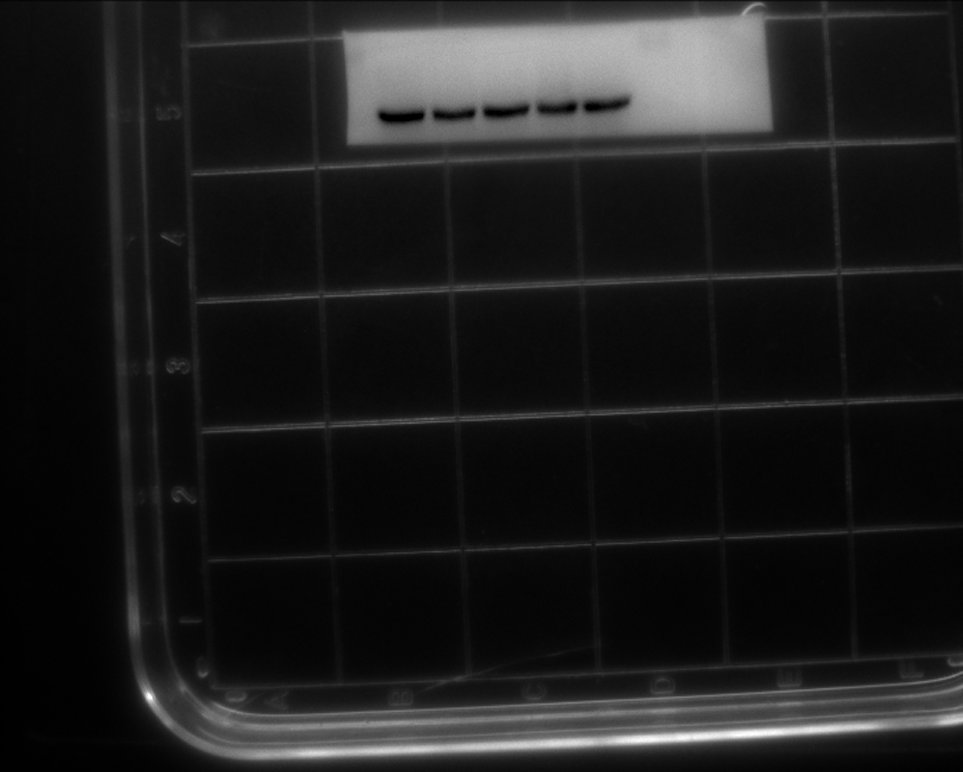

Supplement: Supplementary file 22 [file DataSheet10.ZIP › 2/p-p38 tublin q.tif]

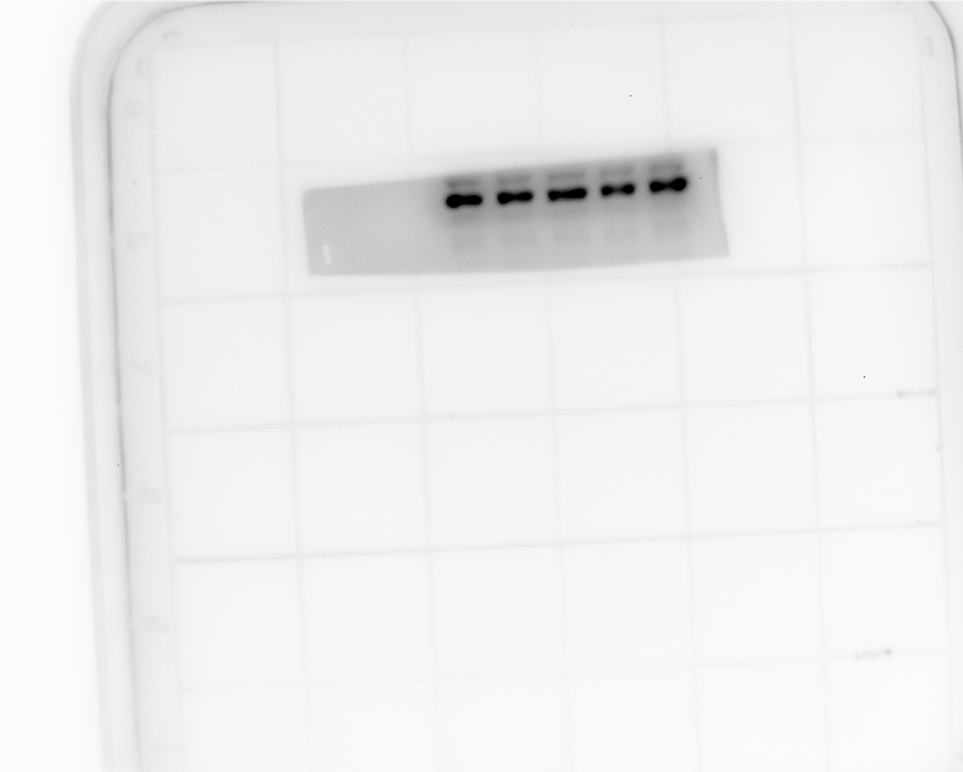

Supplement: Supplementary file 22 [file DataSheet10.ZIP › 2/p38 1.tif]

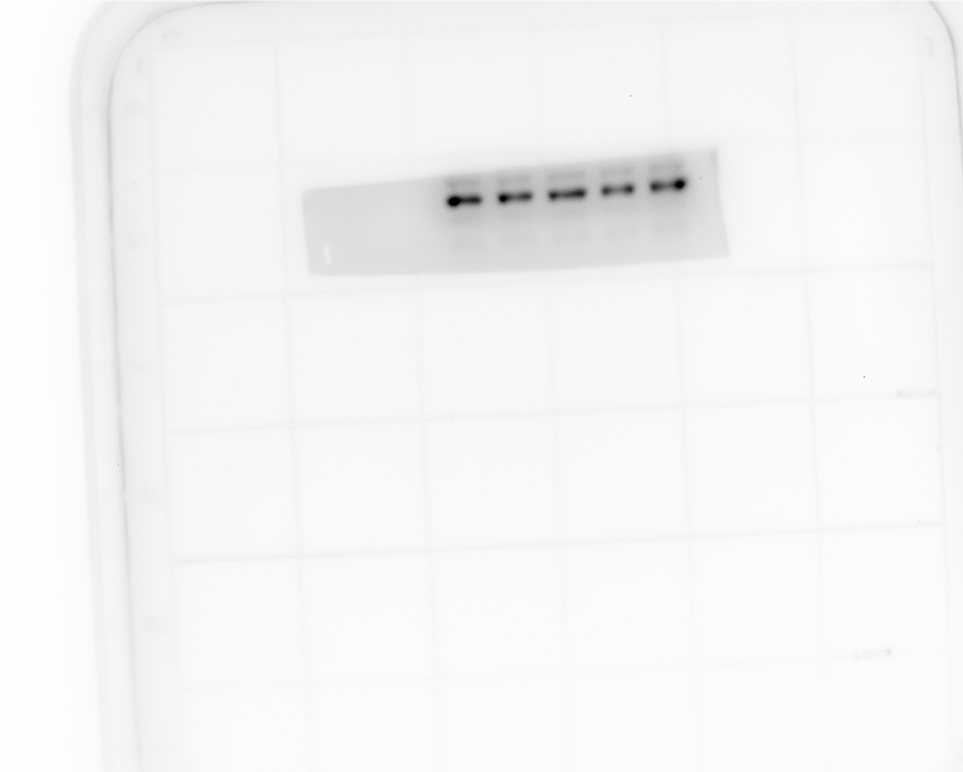

Supplement: Supplementary file 22 [file DataSheet10.ZIP › 2/p38 2.tif]

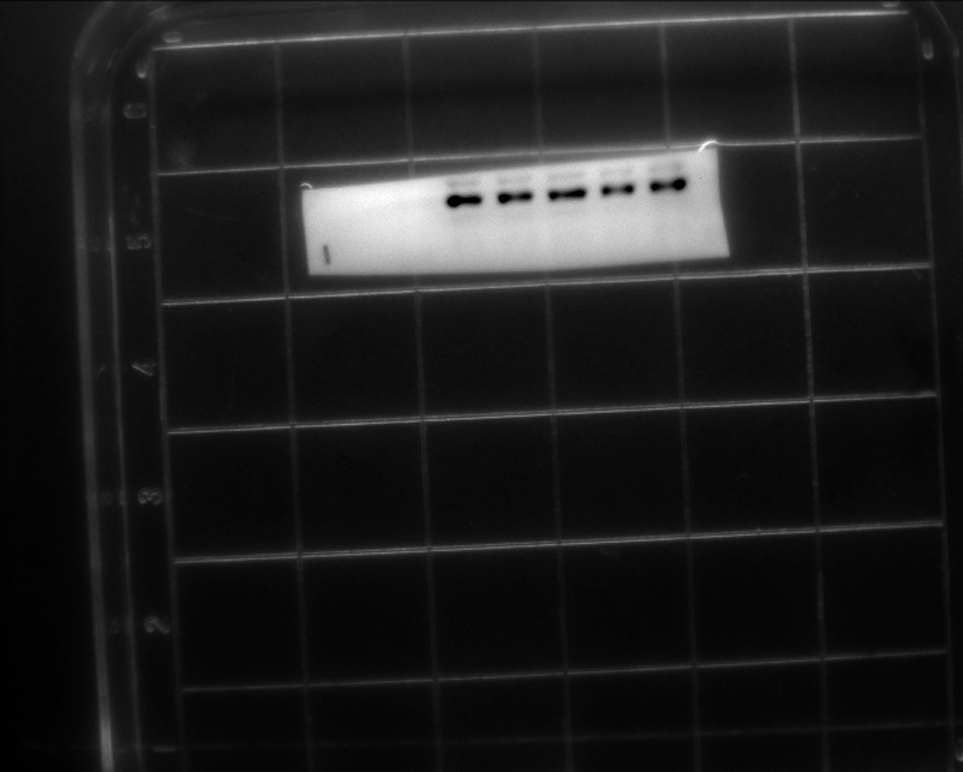

Supplement: Supplementary file 22 [file DataSheet10.ZIP › 2/p38 q.tif]

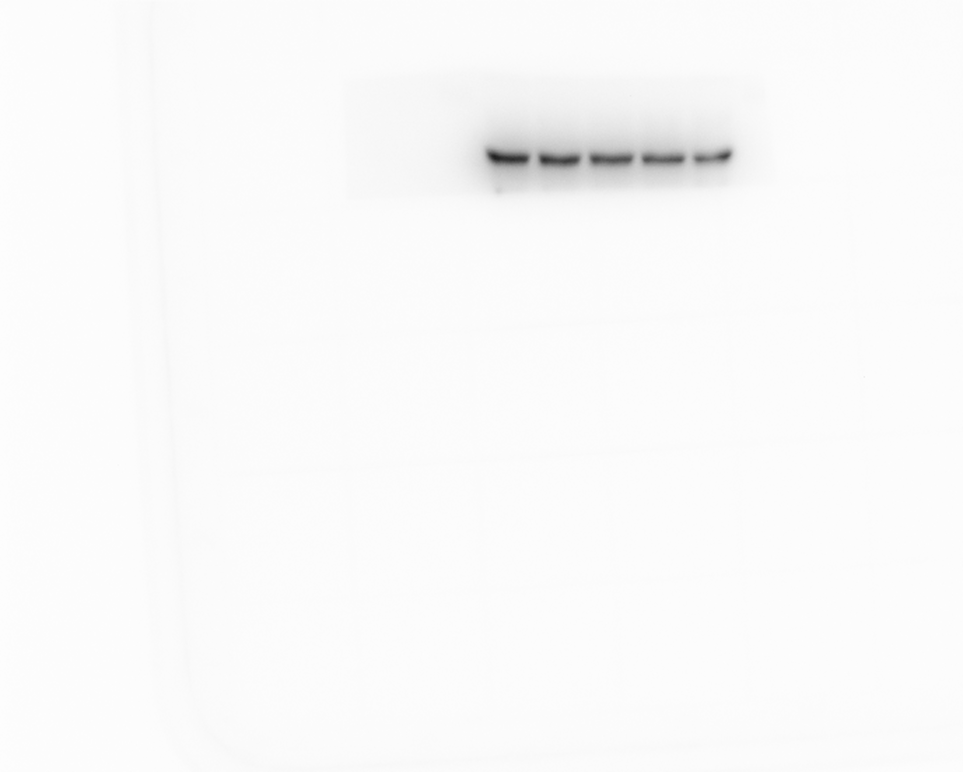

Supplement: Supplementary file 22 [file DataSheet10.ZIP › 2/p38 tublin 1.tif]

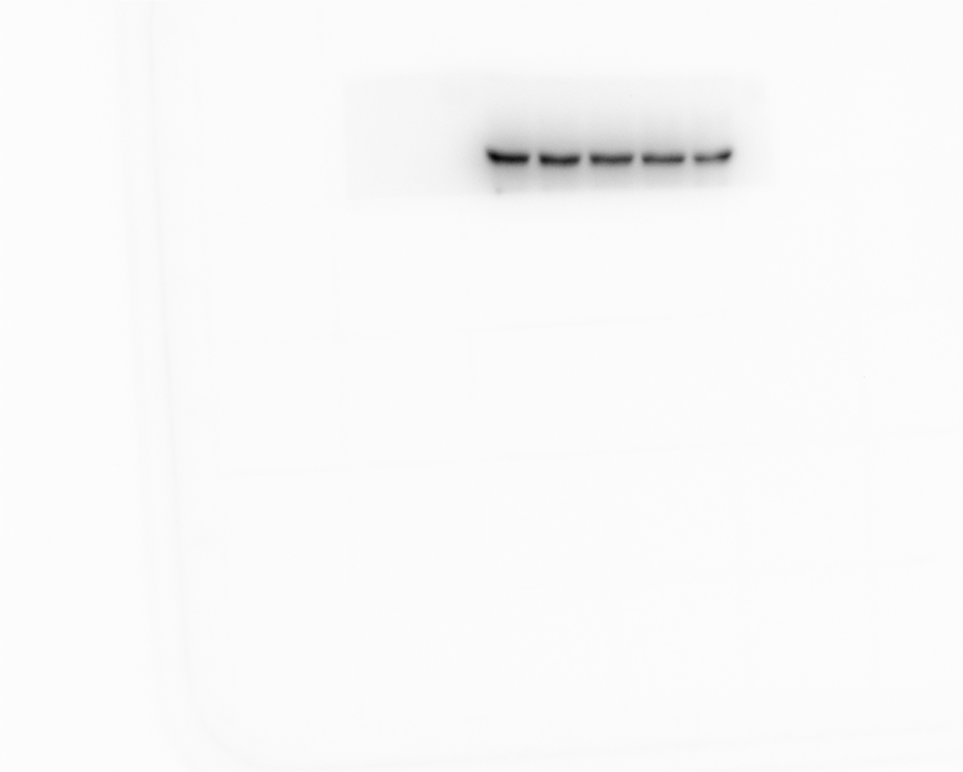

Supplement: Supplementary file 22 [file DataSheet10.ZIP › 2/p38 tublin 2.tif]

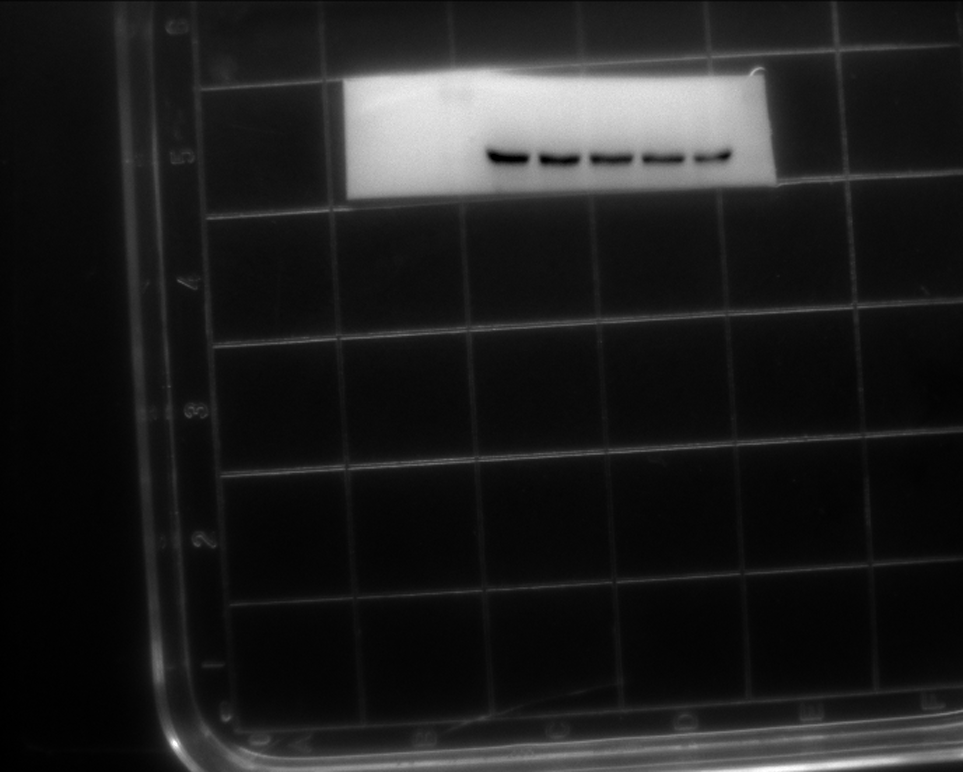

Supplement: Supplementary file 22 [file DataSheet10.ZIP › 2/p38 tublin q.tif]

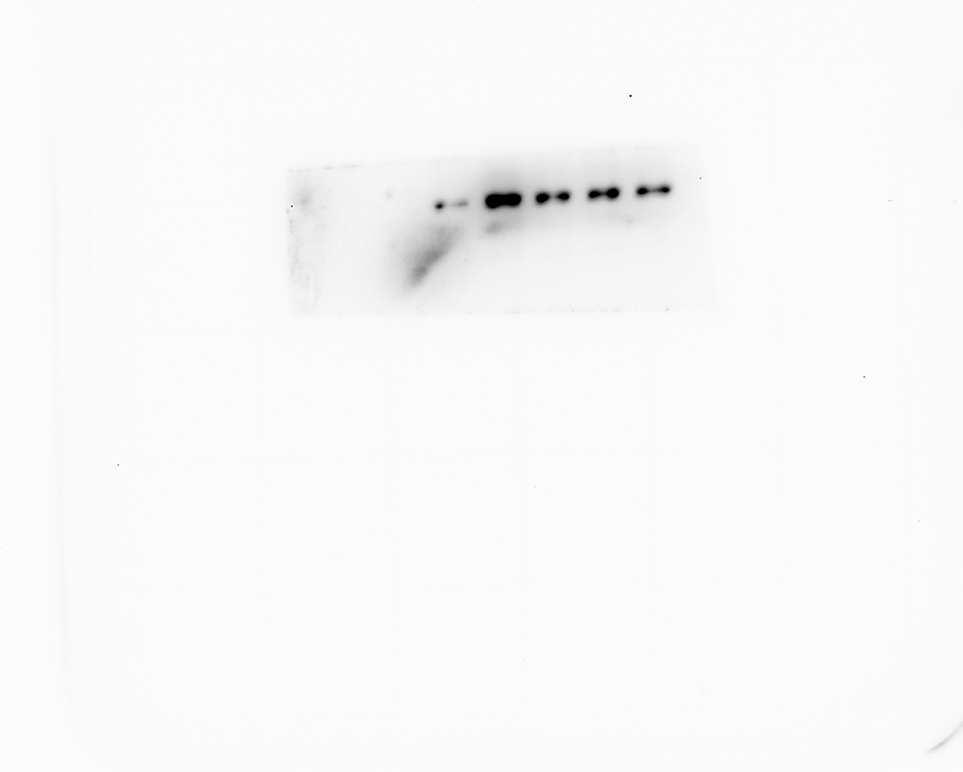

Supplement: Supplementary file 22 [file DataSheet10.ZIP › 3/p-p38 1.tif]

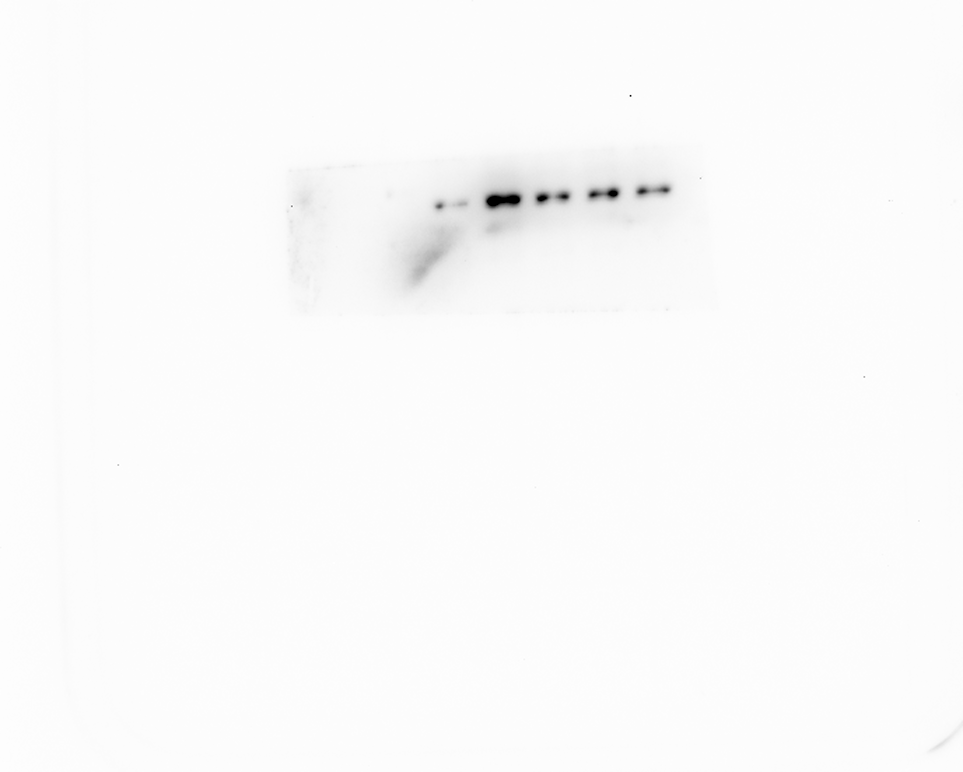

Supplement: Supplementary file 22 [file DataSheet10.ZIP › 3/p-p38 2.tif]

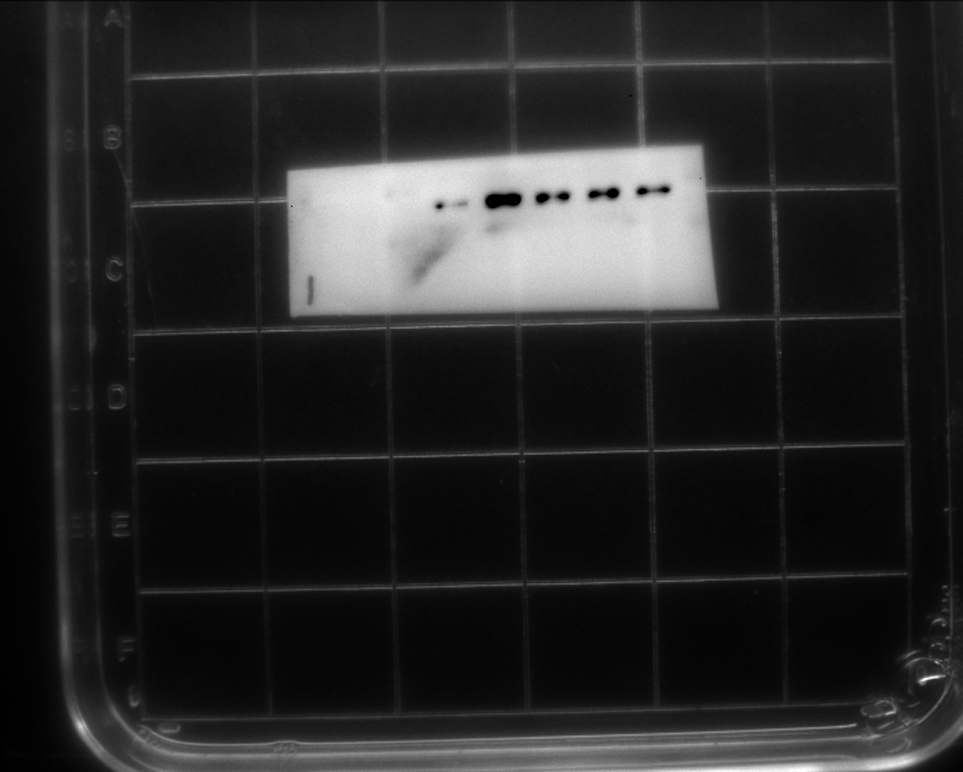

Supplement: Supplementary file 22 [file DataSheet10.ZIP › 3/p-p38 q.tif]

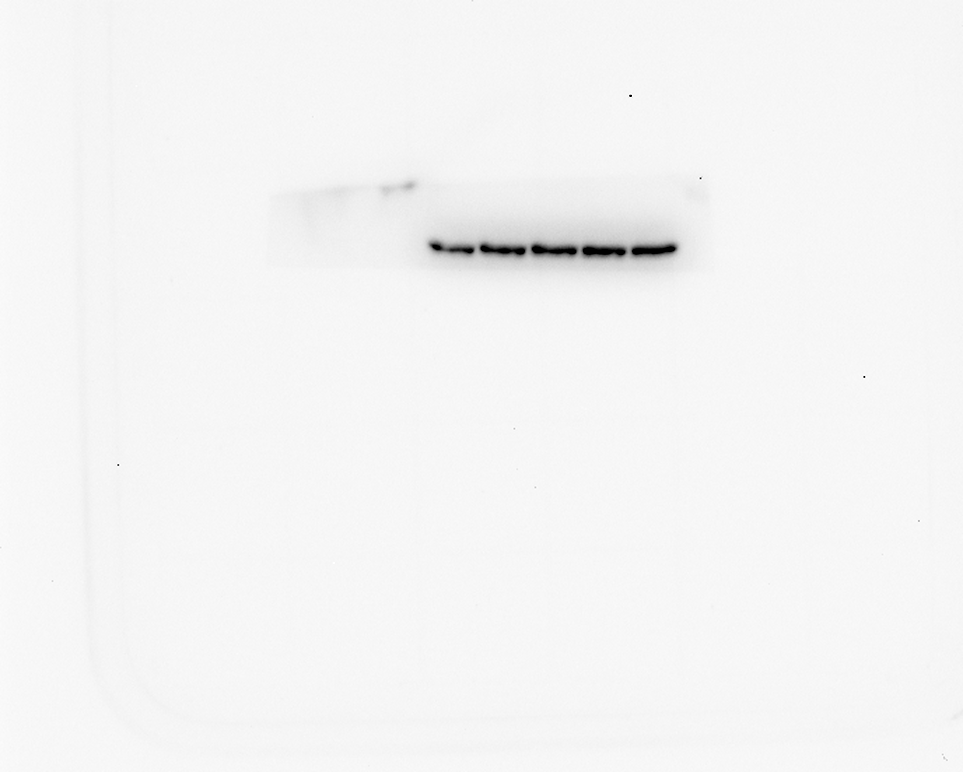

Supplement: Supplementary file 22 [file DataSheet10.ZIP › 3/p-p38 tublin 1.tif]

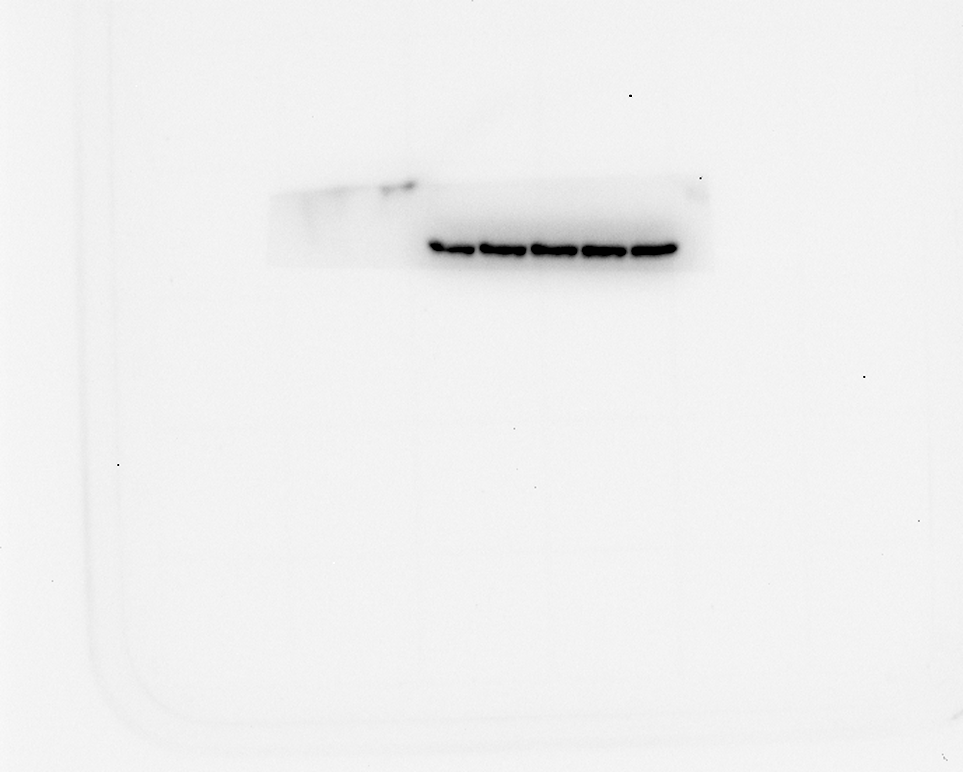

Supplement: Supplementary file 22 [file DataSheet10.ZIP › 3/p-p38 tublin 2.tif]

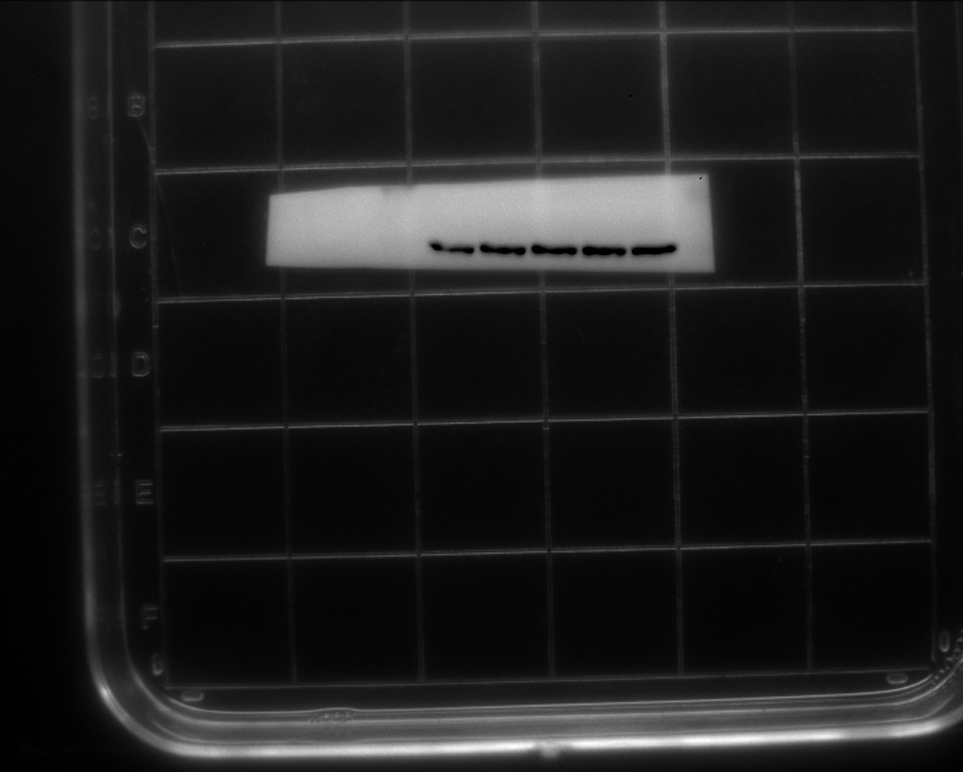

Supplement: Supplementary file 22 [file DataSheet10.ZIP › 3/p-p38 tublin q.tif]

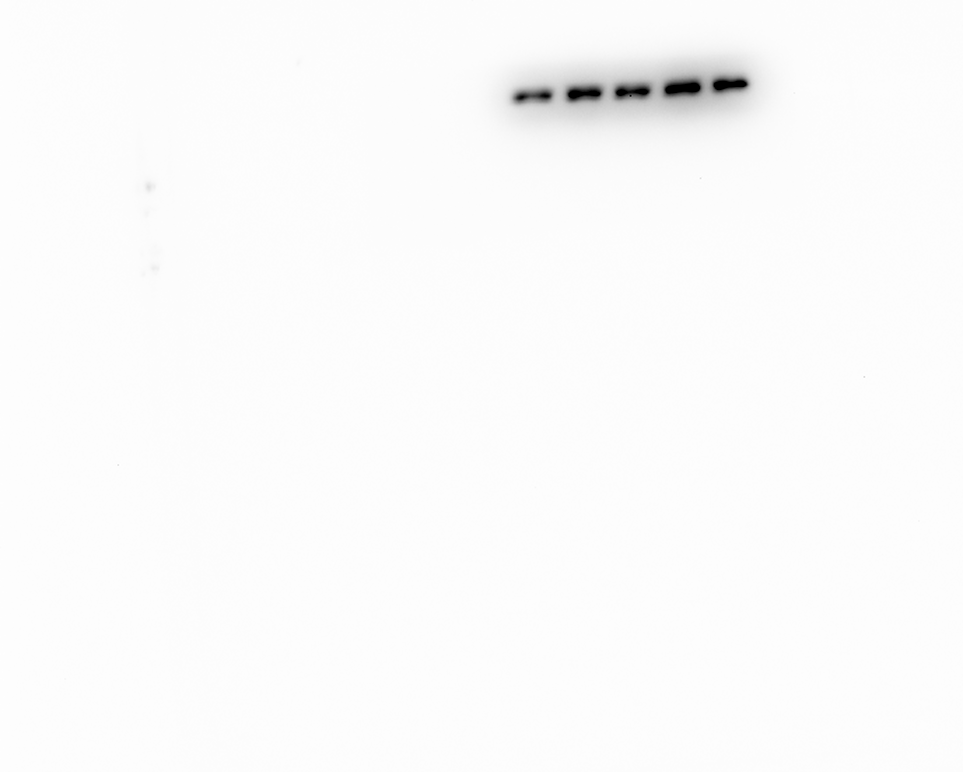

Supplement: Supplementary file 22 [file DataSheet10.ZIP › 3/p38 1.tif]

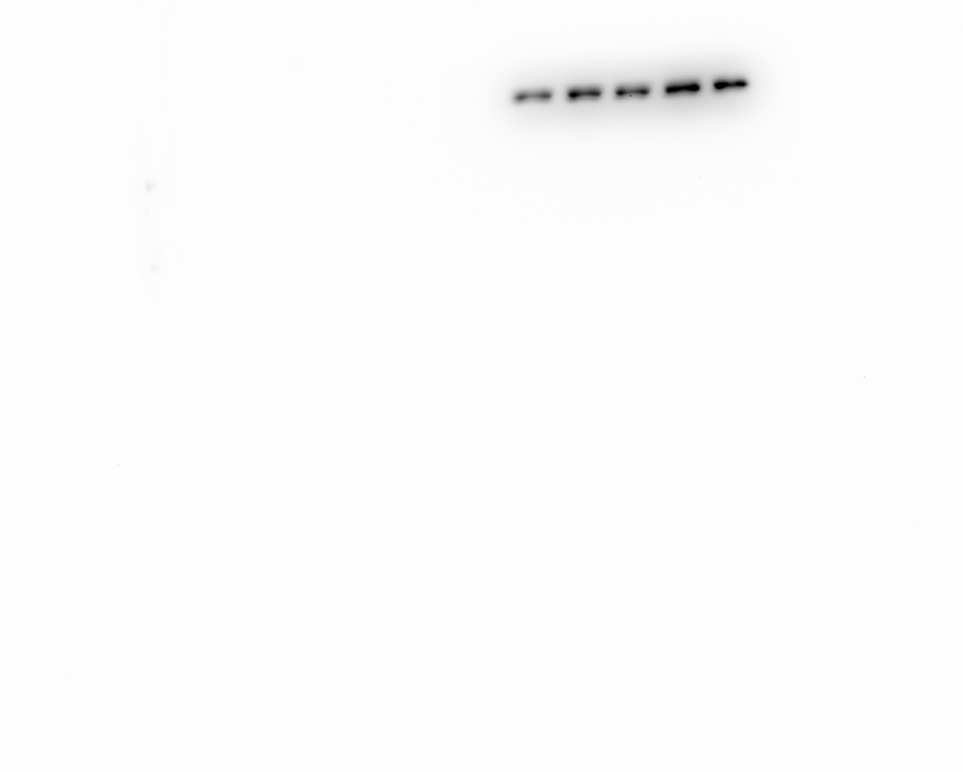

Supplement: Supplementary file 22 [file DataSheet10.ZIP › 3/p38 2.tif]

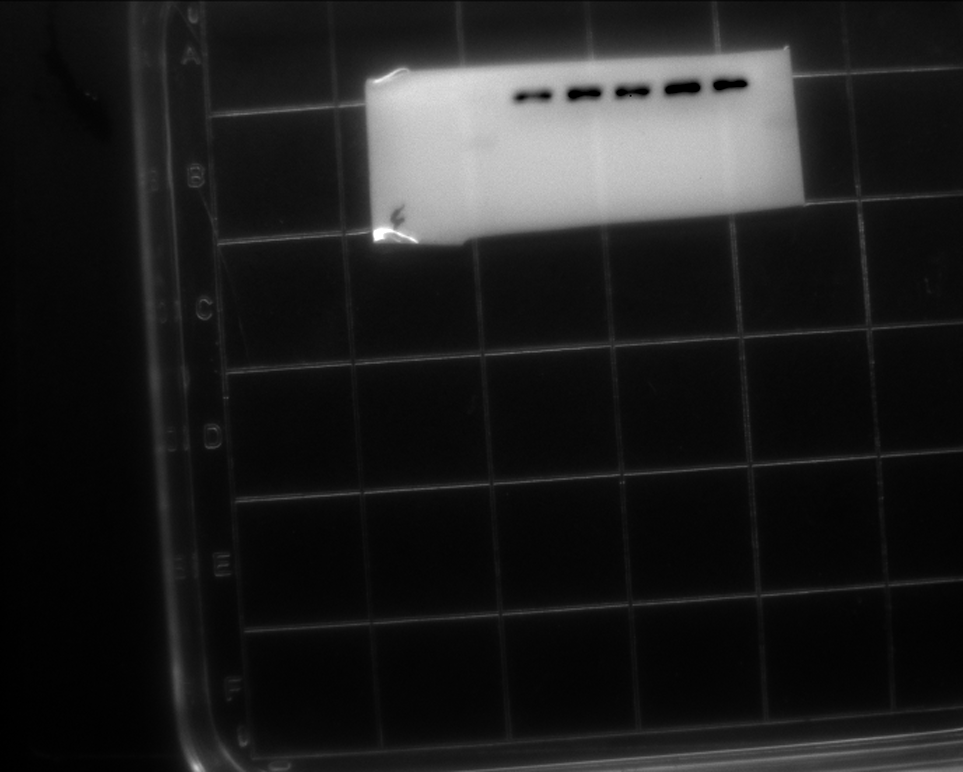

Supplement: Supplementary file 22 [file DataSheet10.ZIP › 3/p38 q.tif]

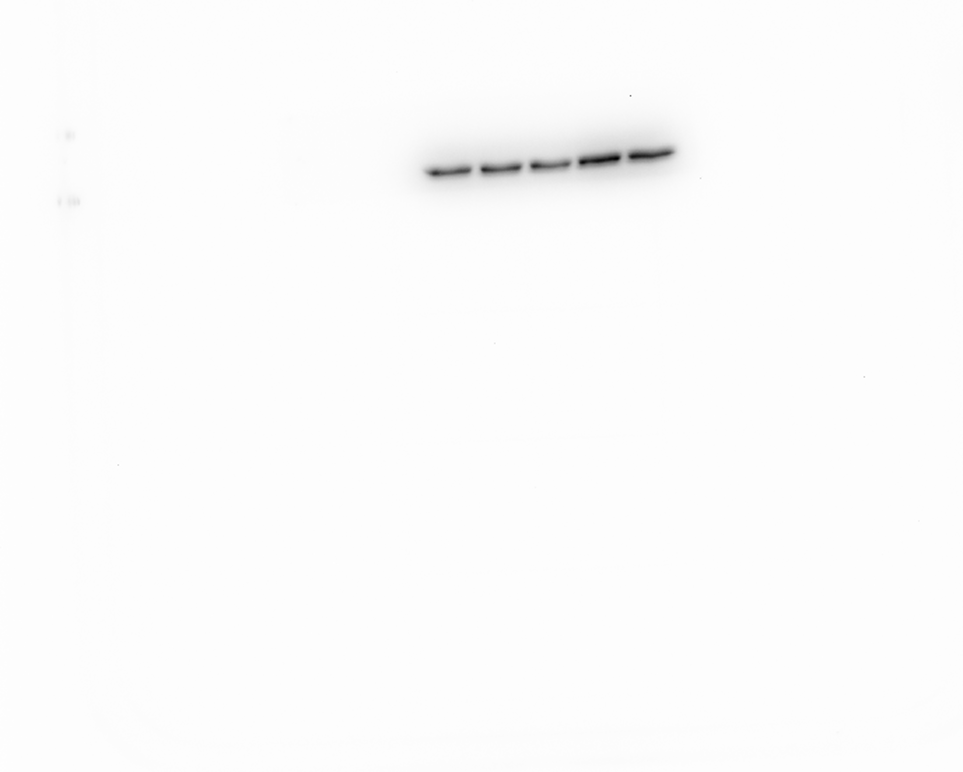

Supplement: Supplementary file 22 [file DataSheet10.ZIP › 3/p38 tublin 1.tif]

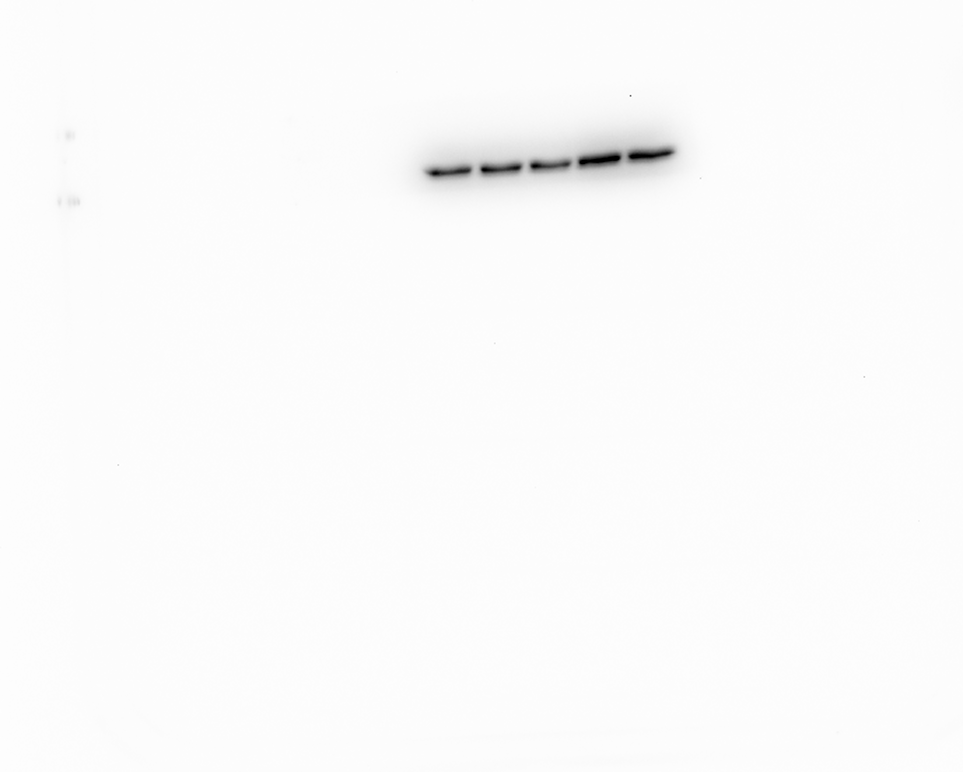

Supplement: Supplementary file 22 [file DataSheet10.ZIP › 3/p38 tublin 2.tif]

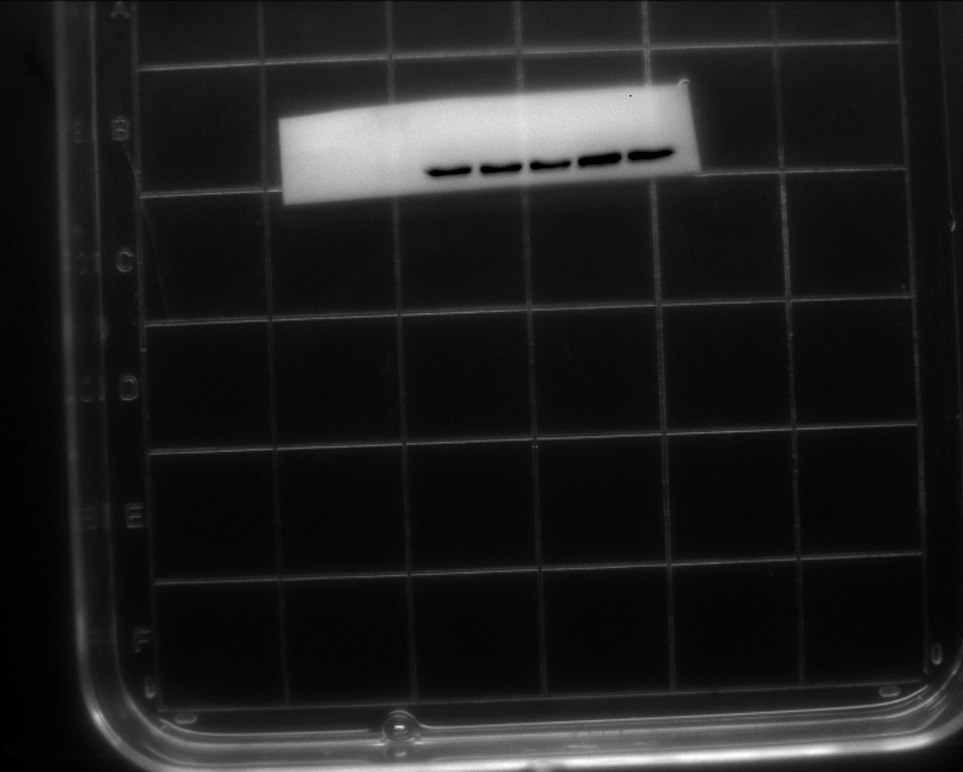

Supplement: Supplementary file 22 [file DataSheet10.ZIP › 3/p38 tublin q.tif]

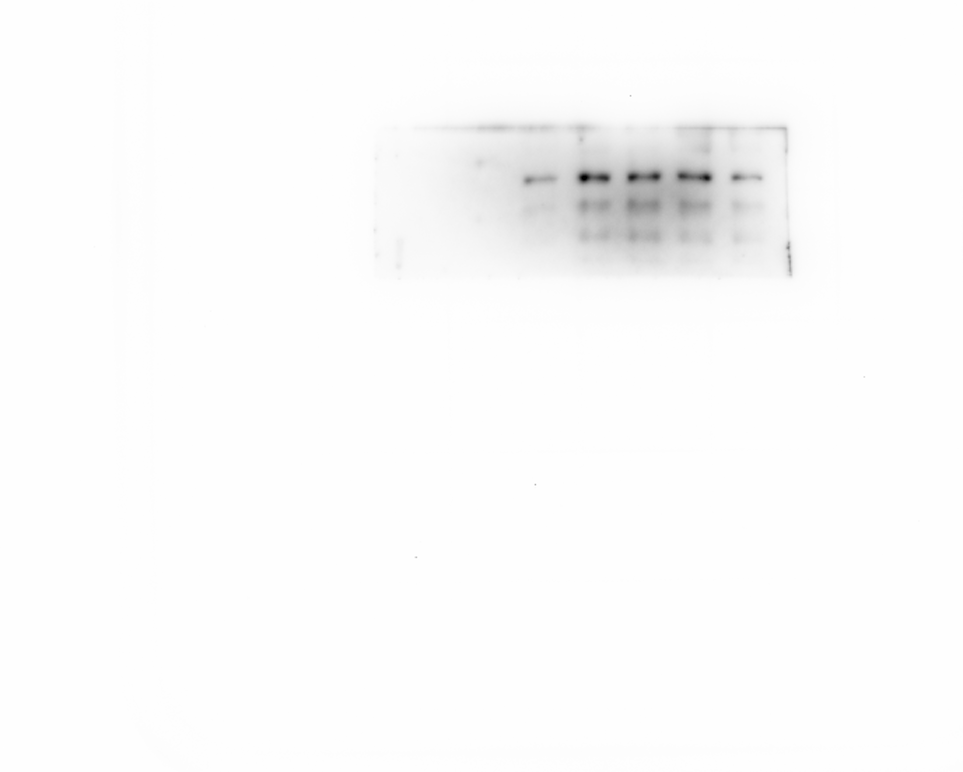

Supplement: Supplementary file 22 [file DataSheet10.ZIP › p38-1/p-p38 1.tif]

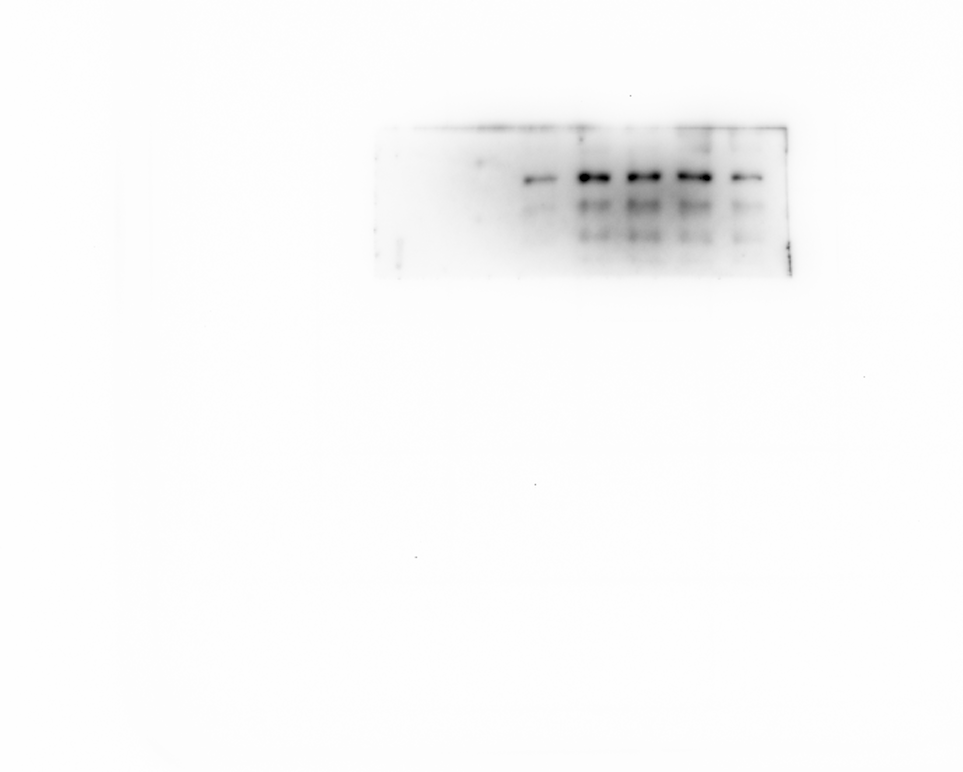

Supplement: Supplementary file 22 [file DataSheet10.ZIP › p38-1/p-p38 2.tif]

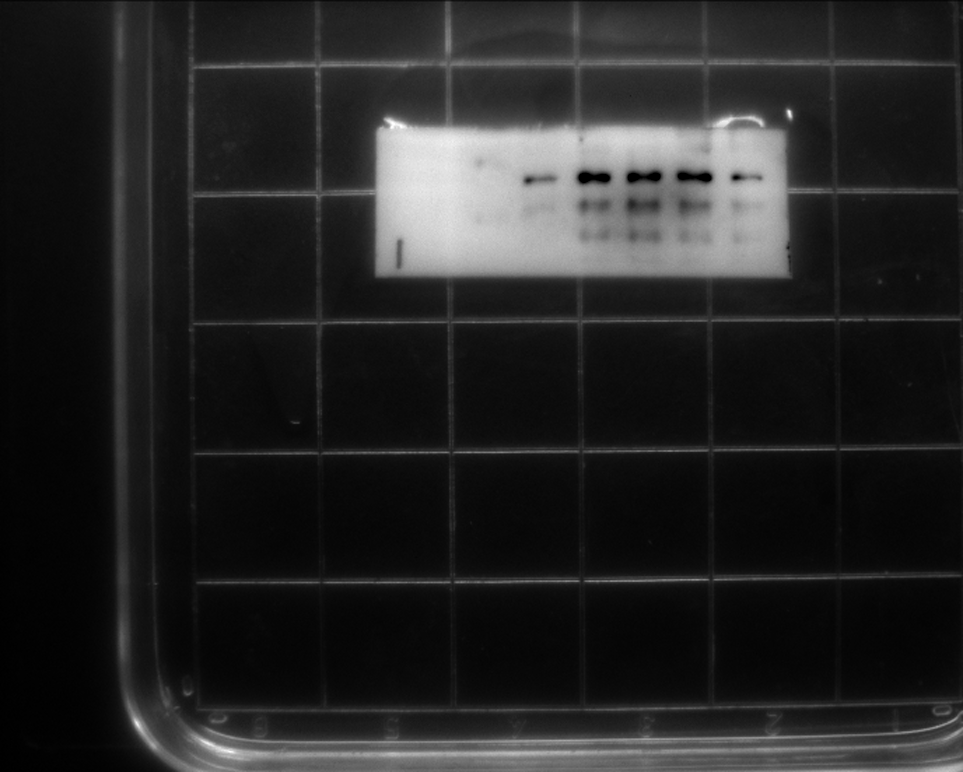

Supplement: Supplementary file 22 [file DataSheet10.ZIP › p38-1/p-p38 q.tif]

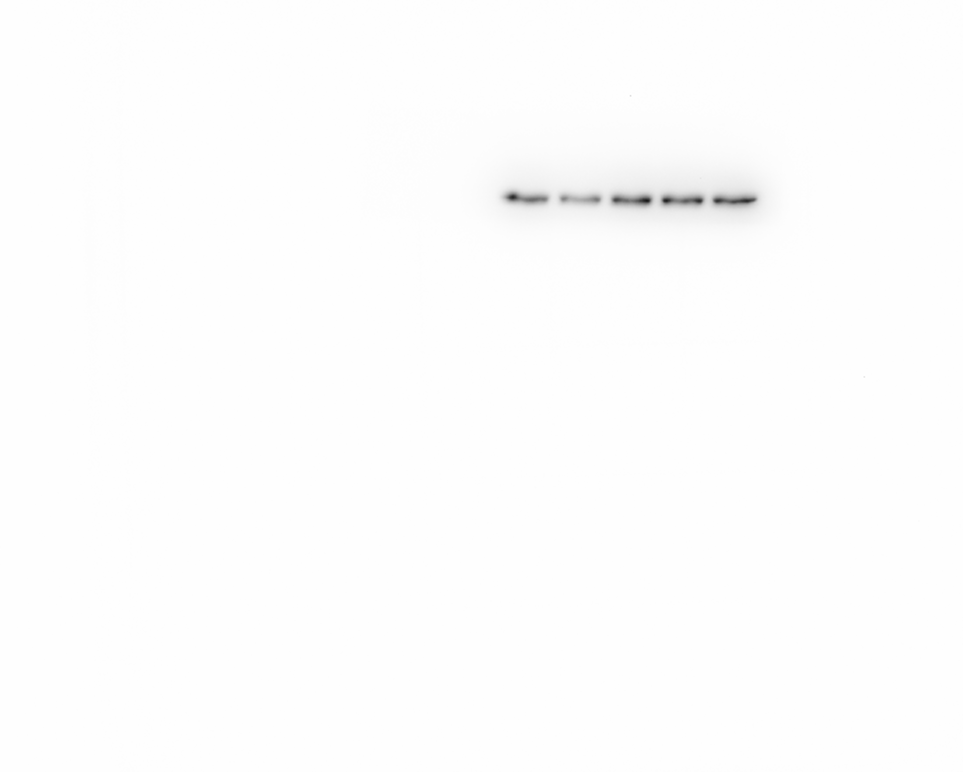

Supplement: Supplementary file 22 [file DataSheet10.ZIP › p38-1/p-p38 tublin1.tif]

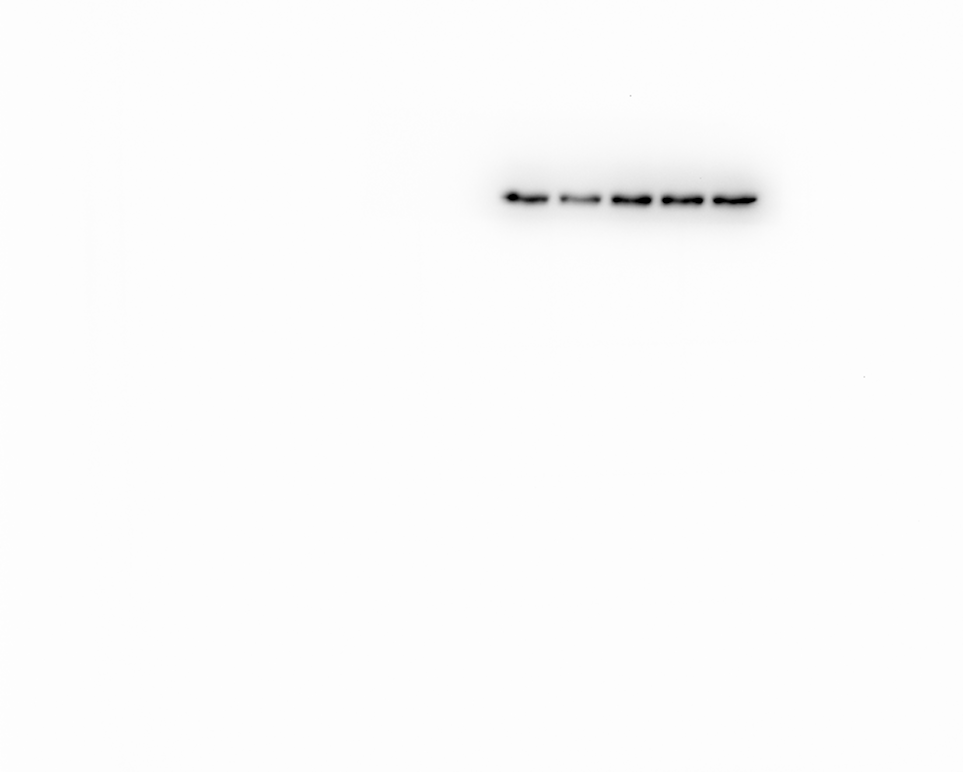

Supplement: Supplementary file 22 [file DataSheet10.ZIP › p38-1/p-p38 tublin2.tif]

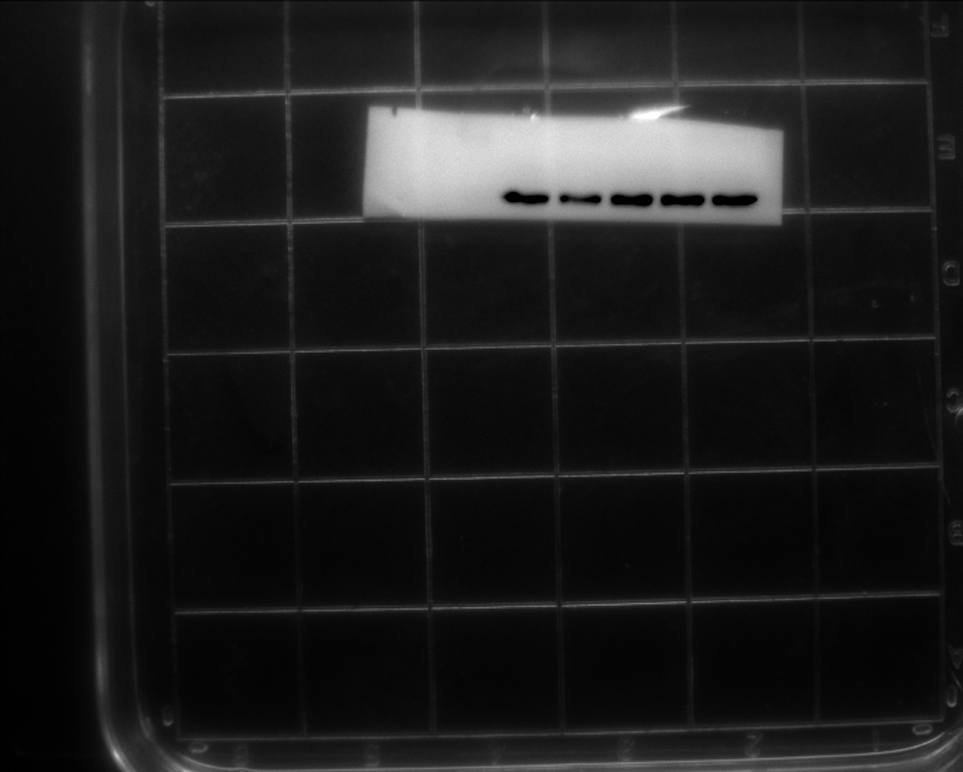

Supplement: Supplementary file 22 [file DataSheet10.ZIP › p38-1/p-p38 tublinq.tif]

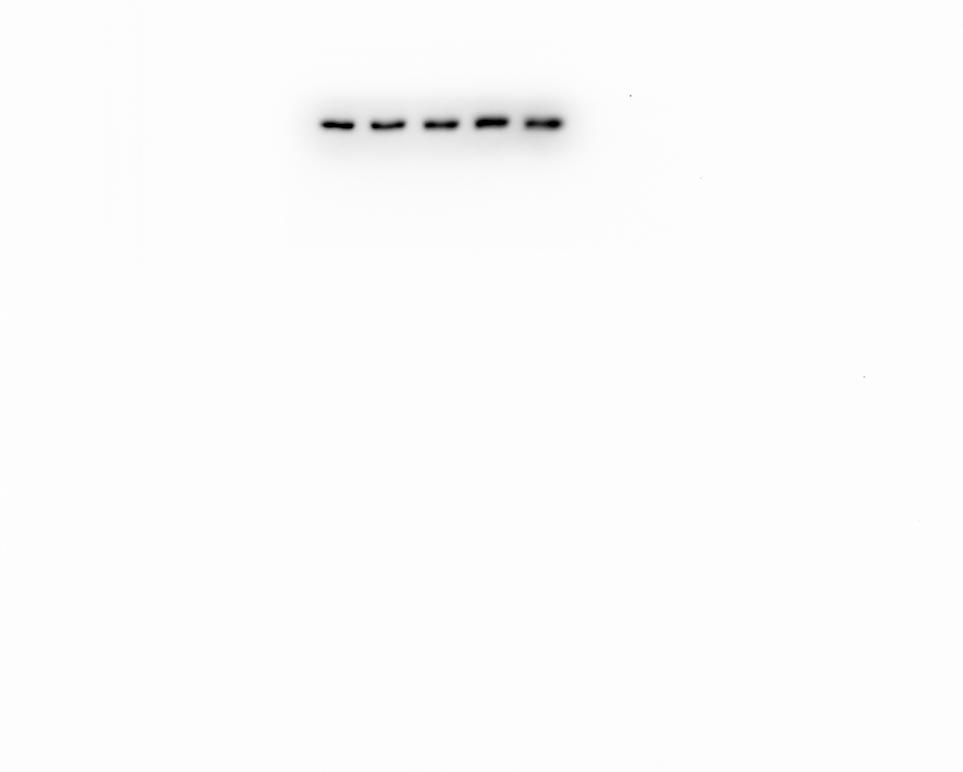

Supplement: Supplementary file 22 [file DataSheet10.ZIP › p38-1/p38 1.tif]

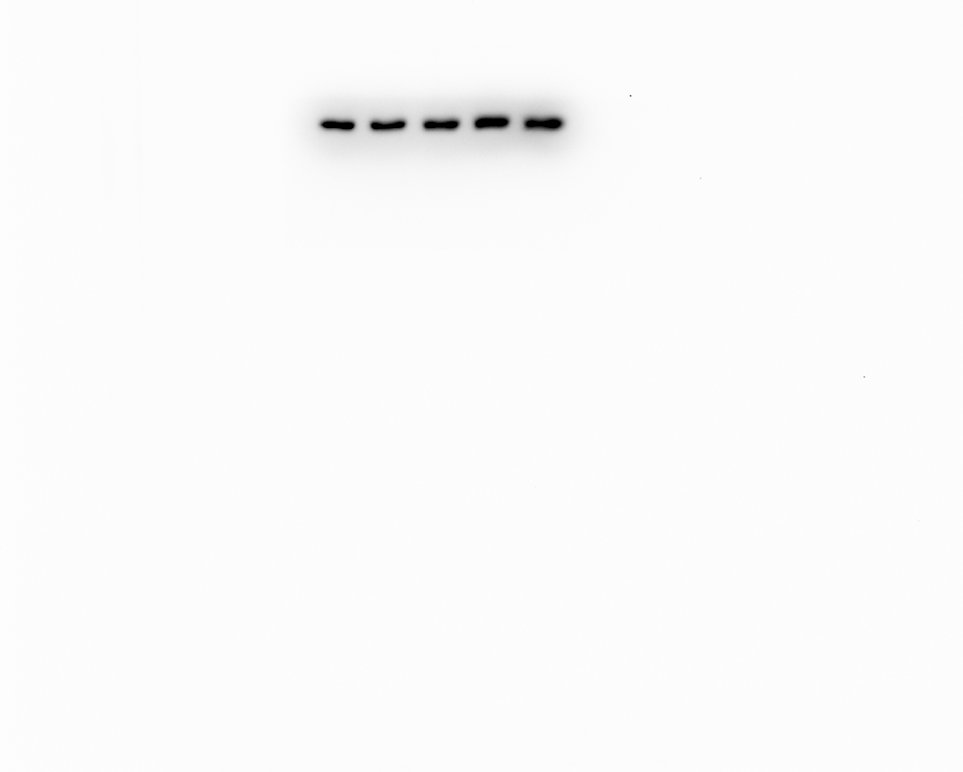

Supplement: Supplementary file 22 [file DataSheet10.ZIP › p38-1/p38 2.tif]

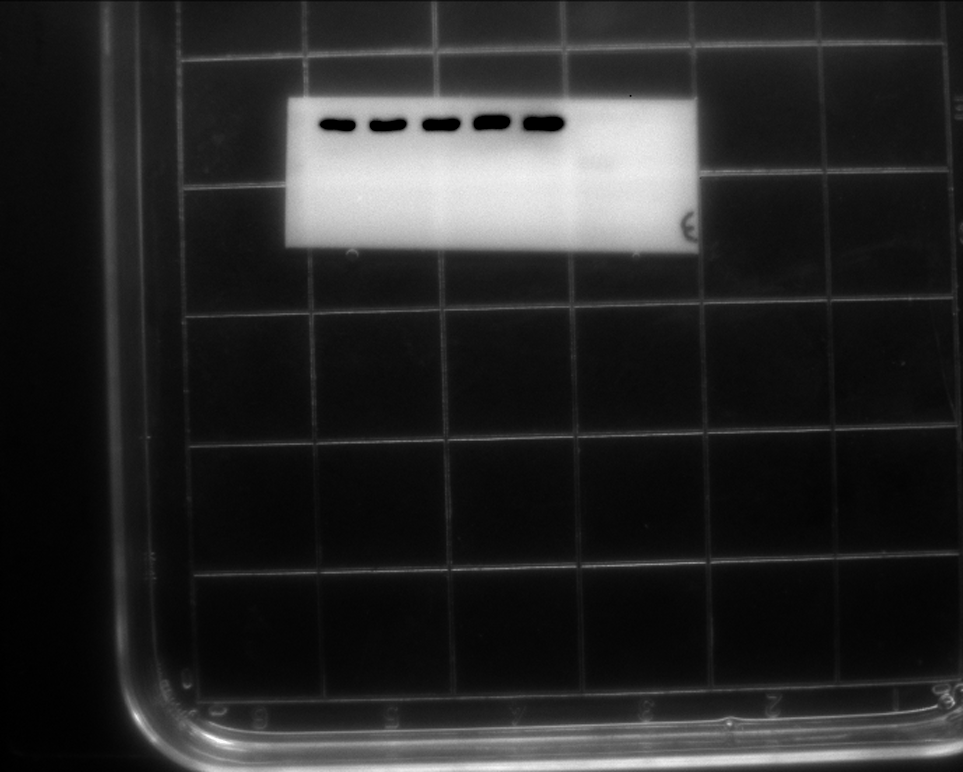

Supplement: Supplementary file 22 [file DataSheet10.ZIP › p38-1/p38 q.tif]

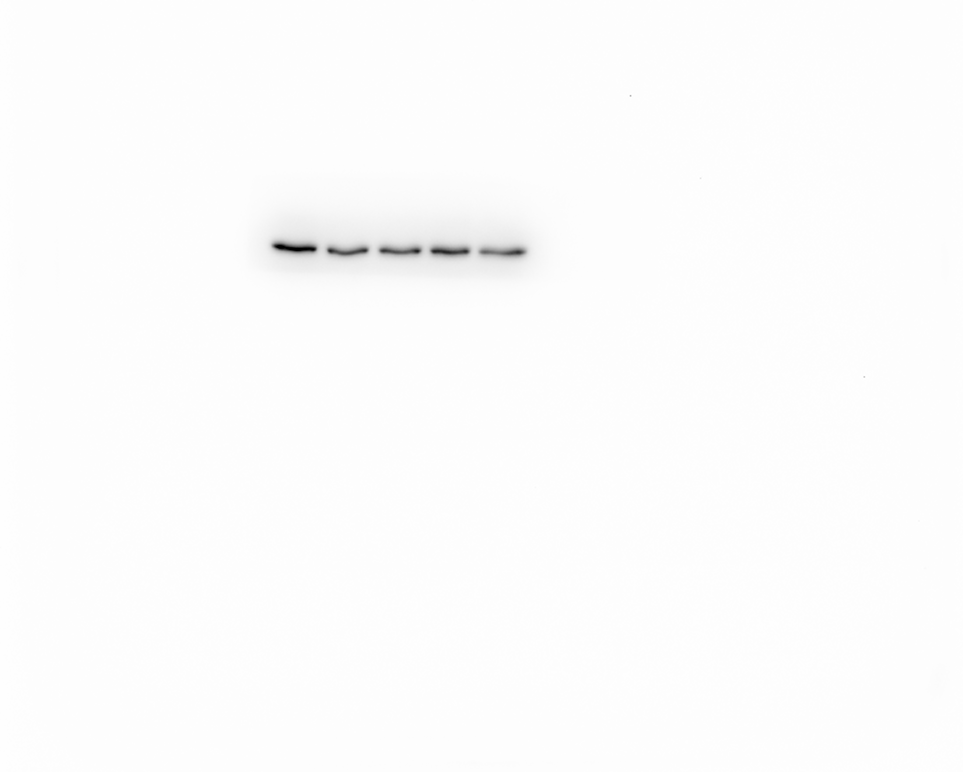

Supplement: Supplementary file 22 [file DataSheet10.ZIP › p38-1/p38 tublin 1.tif]

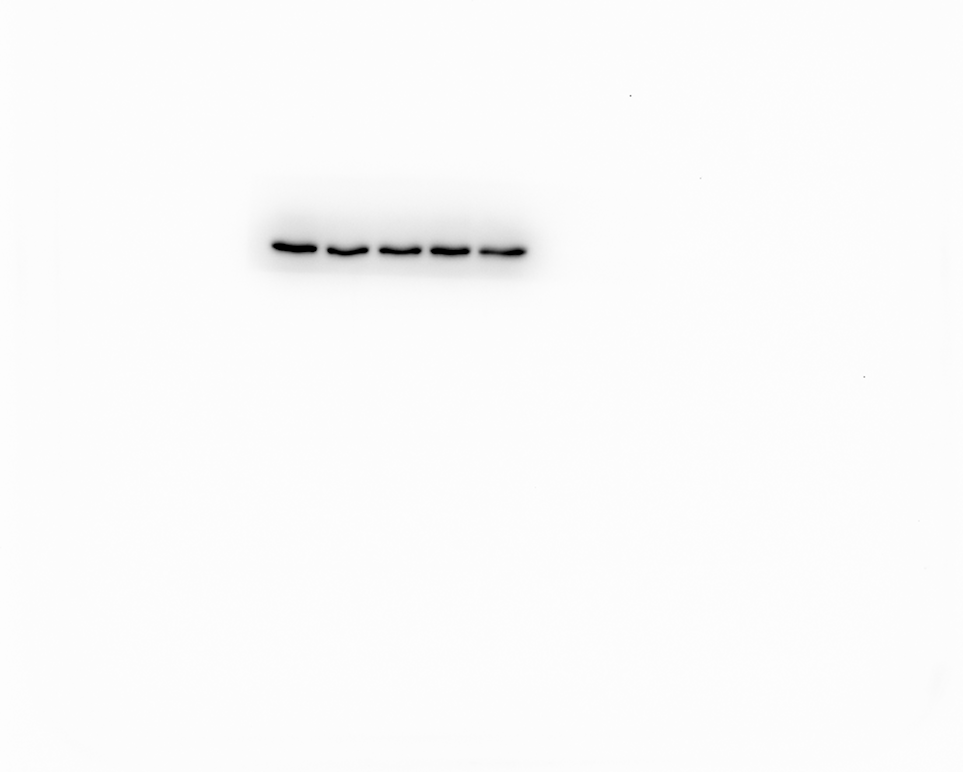

Supplement: Supplementary file 22 [file DataSheet10.ZIP › p38-1/p38 tublin 2.tif]

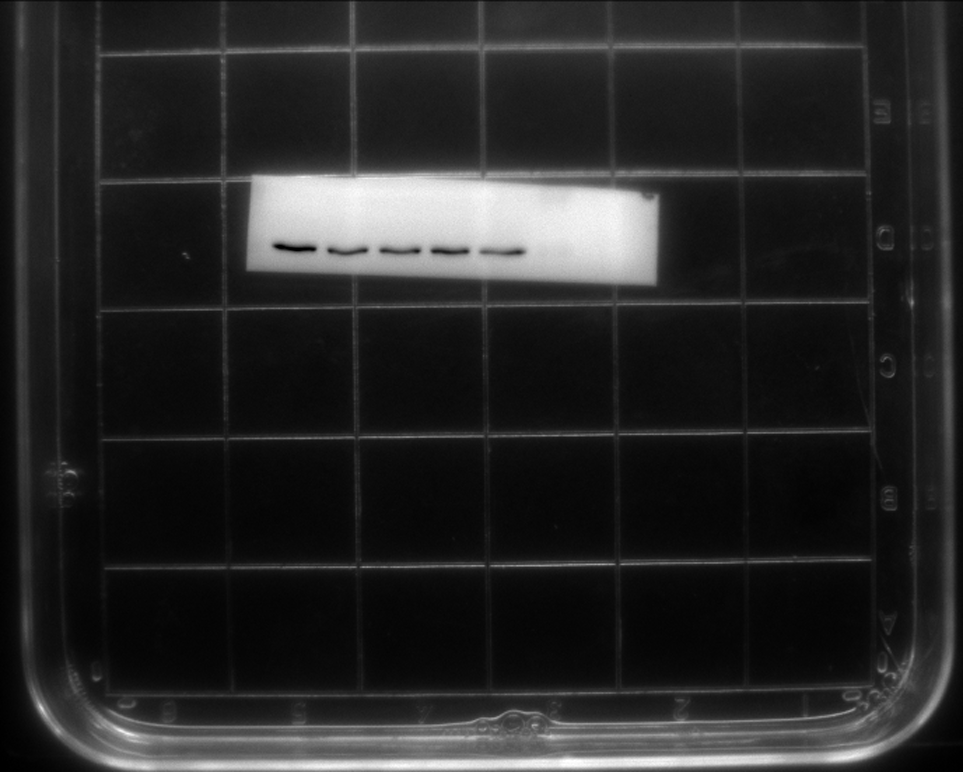

Supplement: Supplementary file 22 [file DataSheet10.ZIP › p38-1/p38 tublin q.tif]

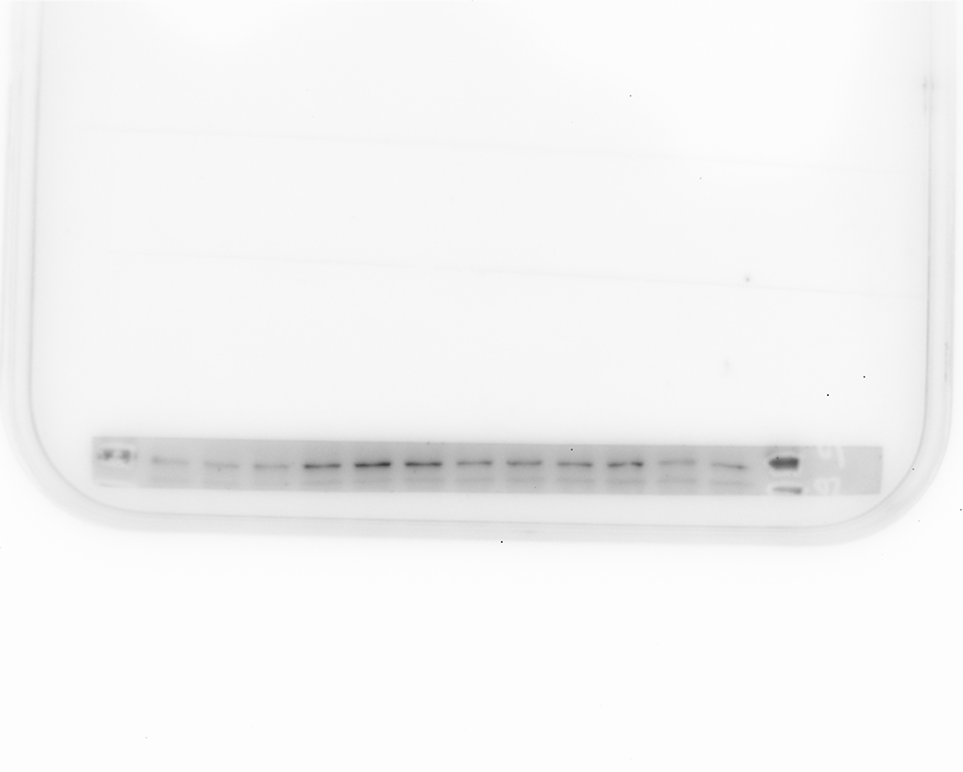

Supplement: Supplementary file 23 [file DataSheet6.ZIP › P65-1/p-p65 1.tif]

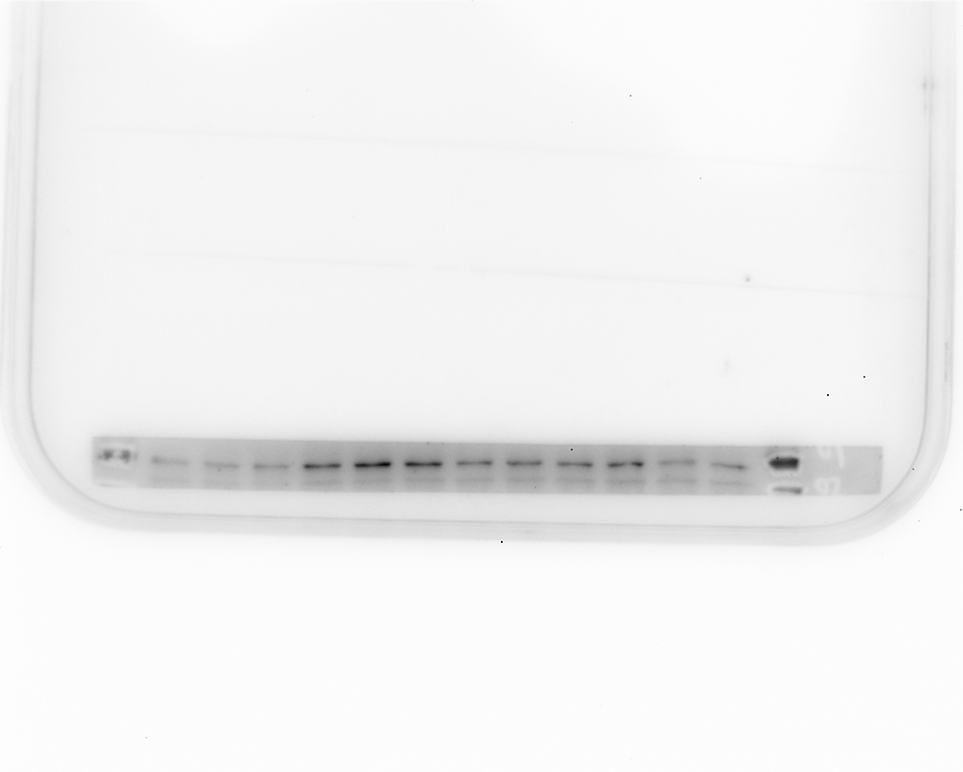

Supplement: Supplementary file 23 [file DataSheet6.ZIP › P65-1/p-p65 2.tif]

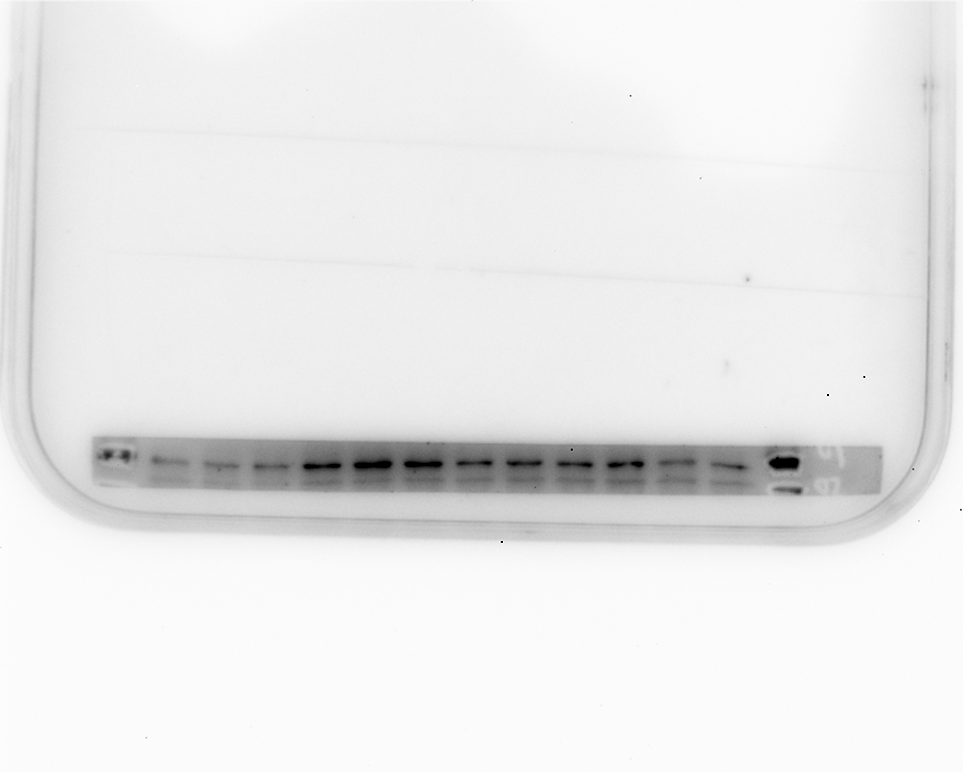

Supplement: Supplementary file 23 [file DataSheet6.ZIP › P65-1/p-p65 6.tif]

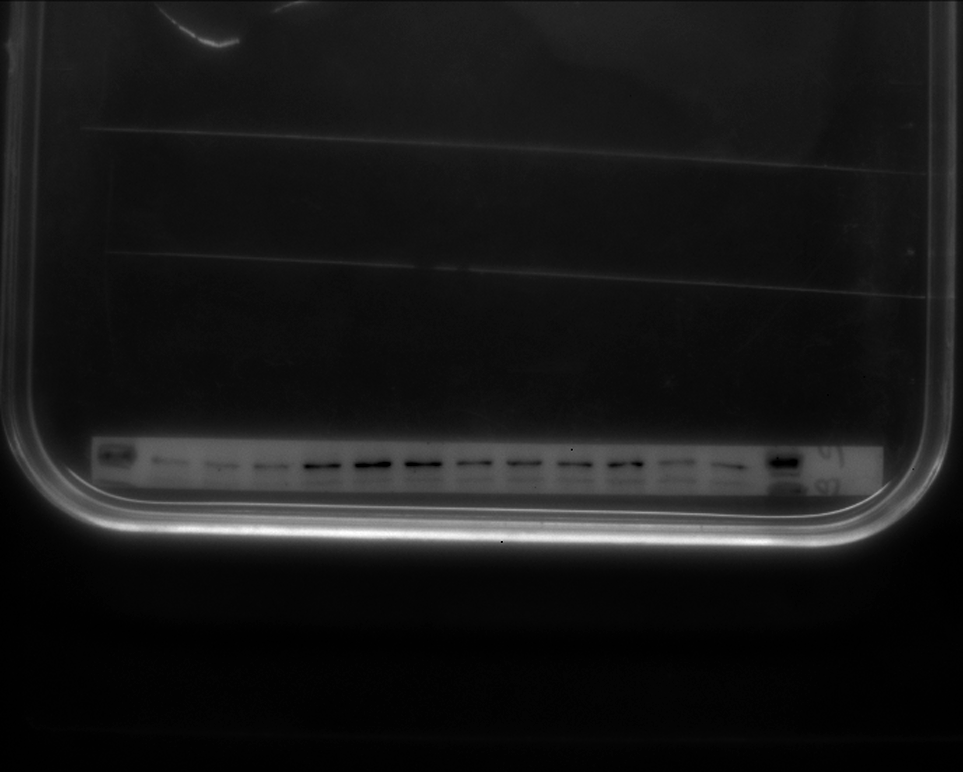

Supplement: Supplementary file 23 [file DataSheet6.ZIP › P65-1/p-p65 q.tif]

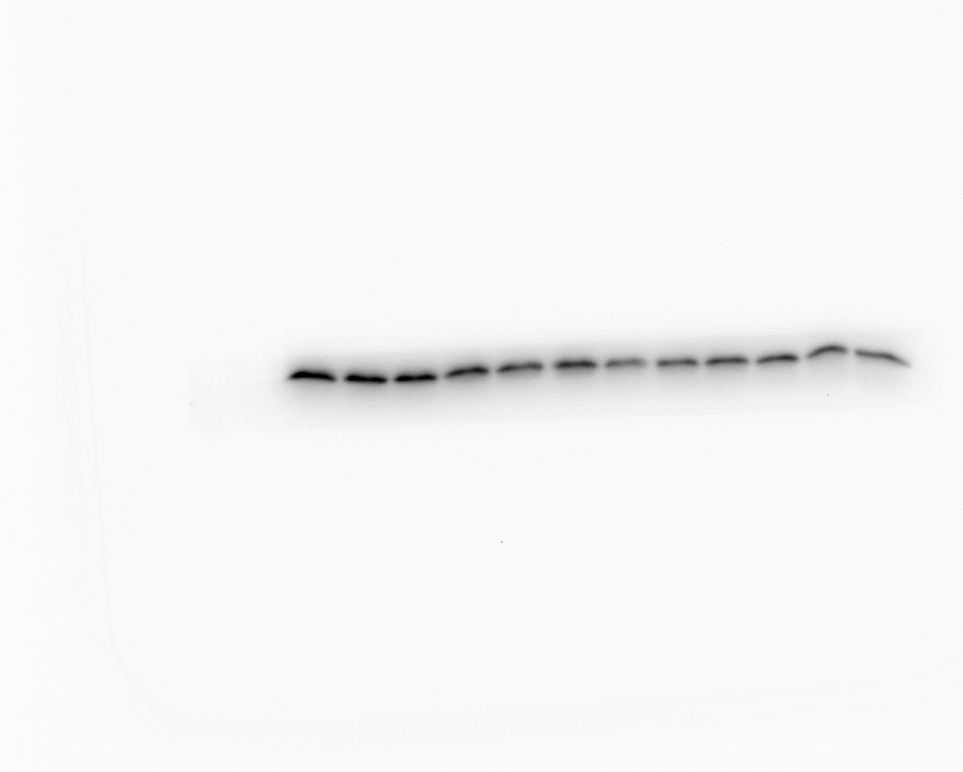

Supplement: Supplementary file 23 [file DataSheet6.ZIP › P65-1/p-p65CYPB1.tif]

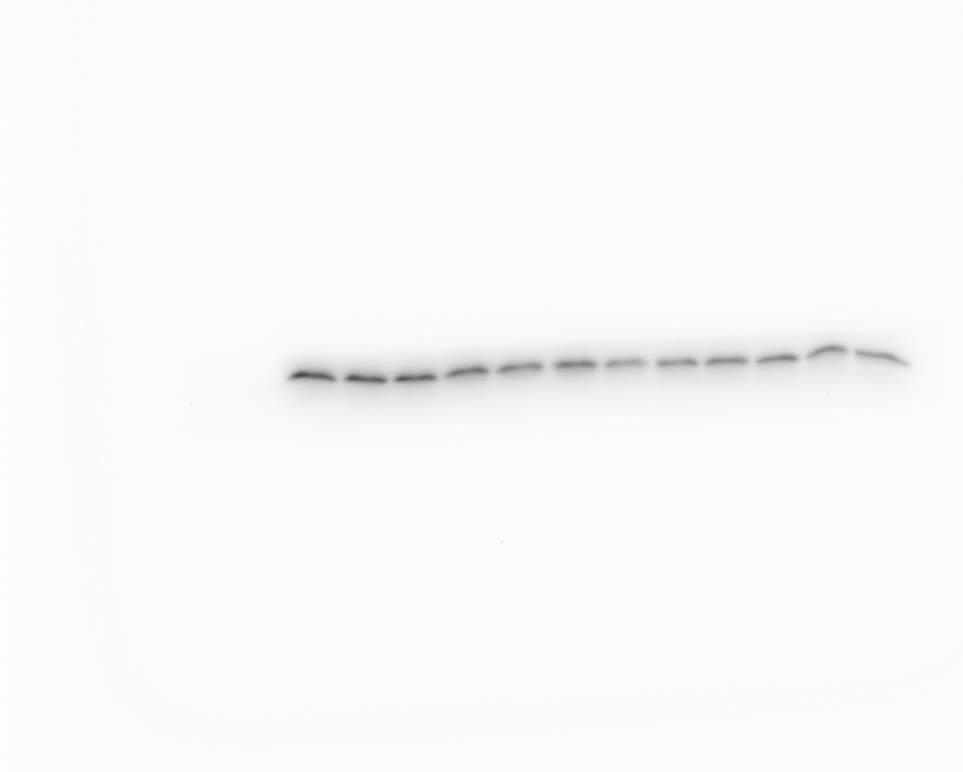

Supplement: Supplementary file 23 [file DataSheet6.ZIP › P65-1/p-p65CYPB2.tif]

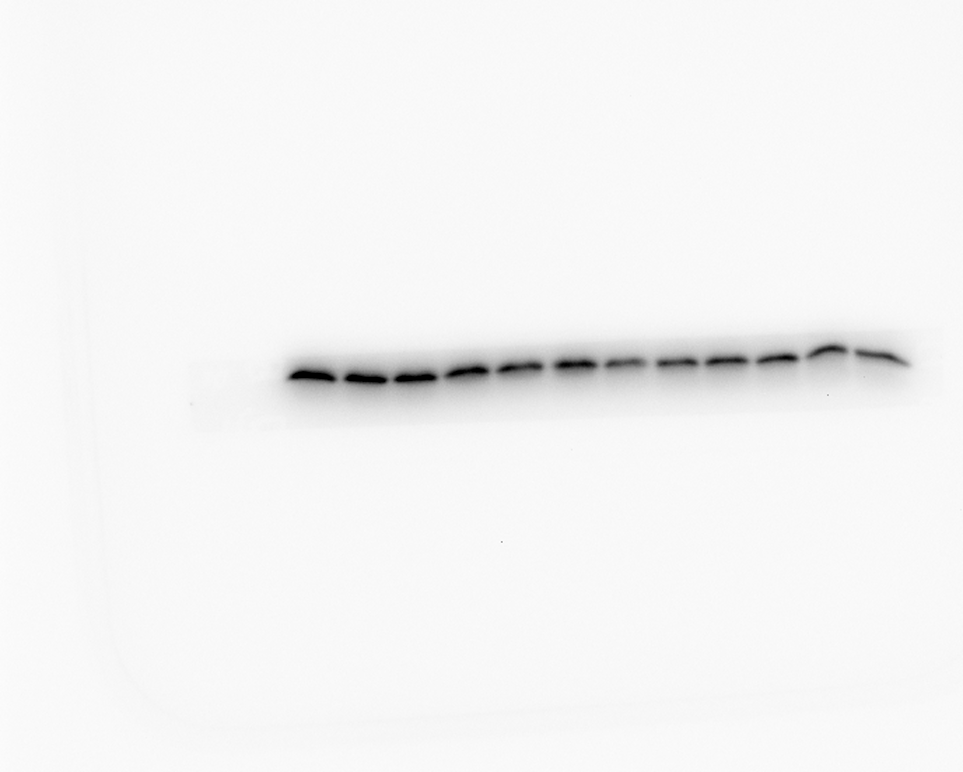

Supplement: Supplementary file 23 [file DataSheet6.ZIP › P65-1/p-p65CYPB3.tif]

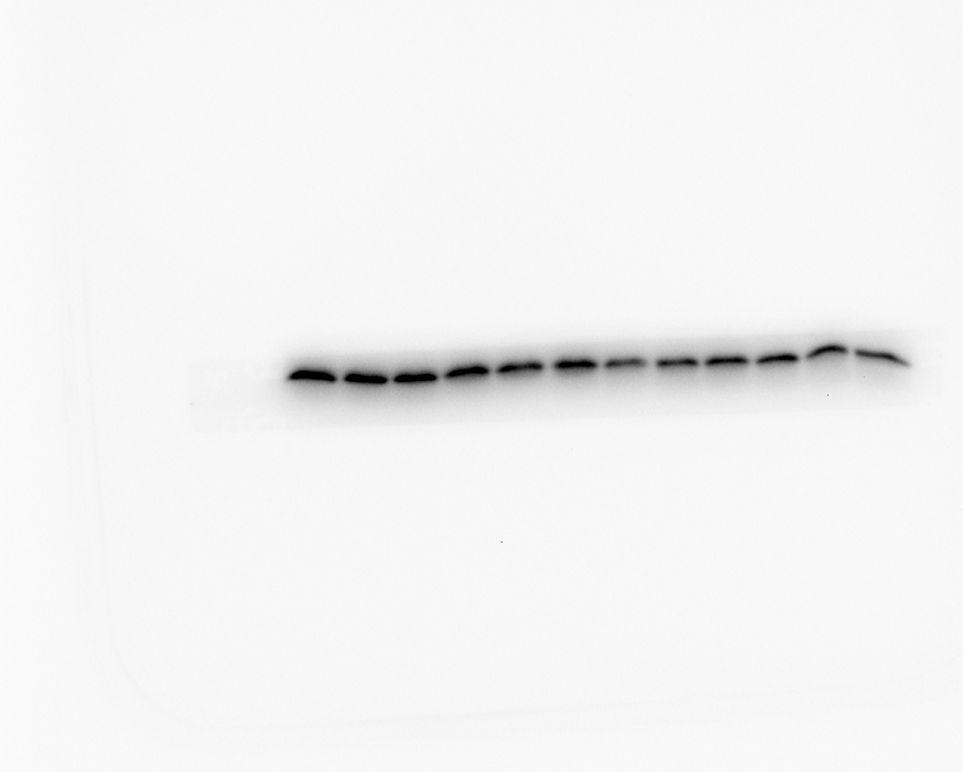

Supplement: Supplementary file 23 [file DataSheet6.ZIP › P65-1/p-p65CYPB4.tif]

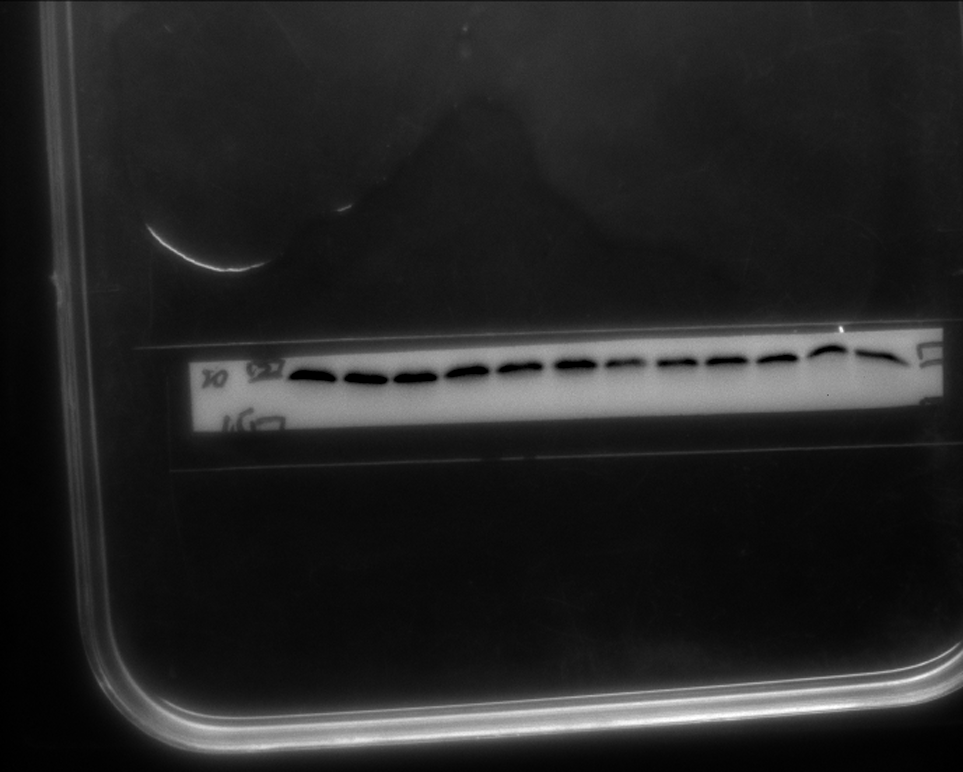

Supplement: Supplementary file 23 [file DataSheet6.ZIP › P65-1/p-p65CYPBq.tif]

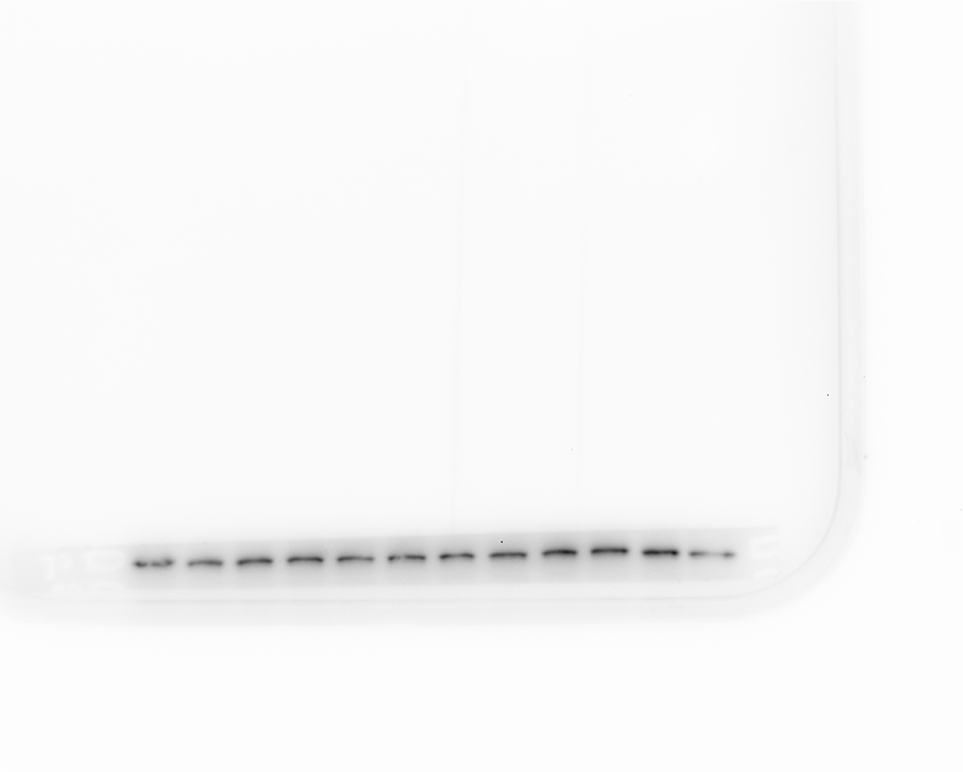

Supplement: Supplementary file 23 [file DataSheet6.ZIP › P65-1/p65 1.tif]

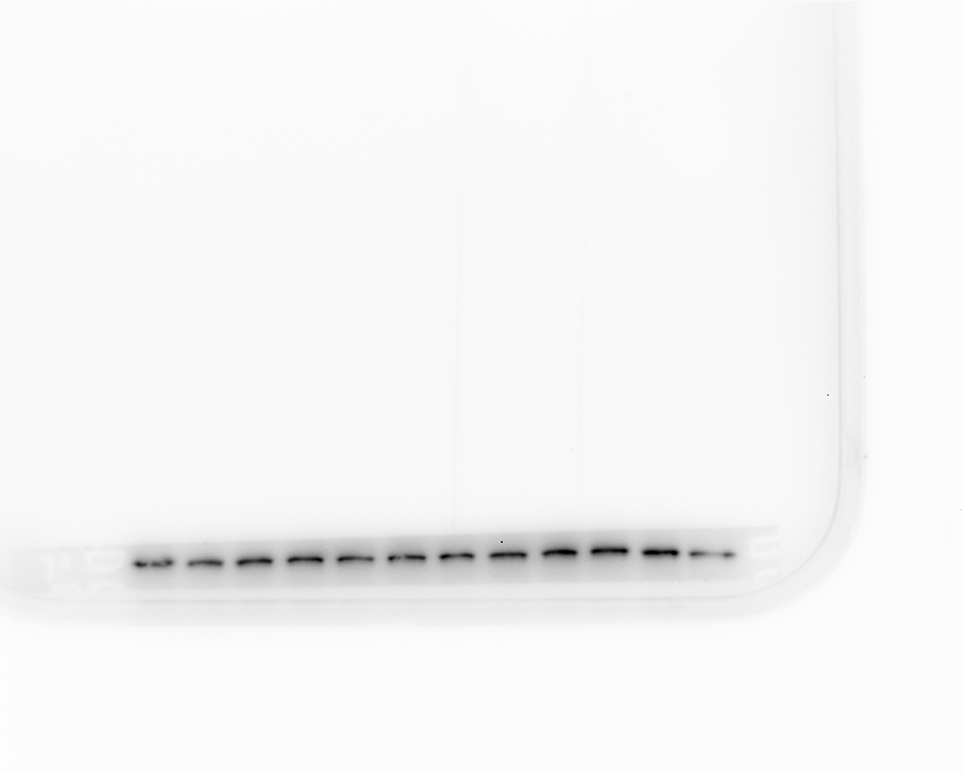

Supplement: Supplementary file 23 [file DataSheet6.ZIP › P65-1/p65 2.tif]

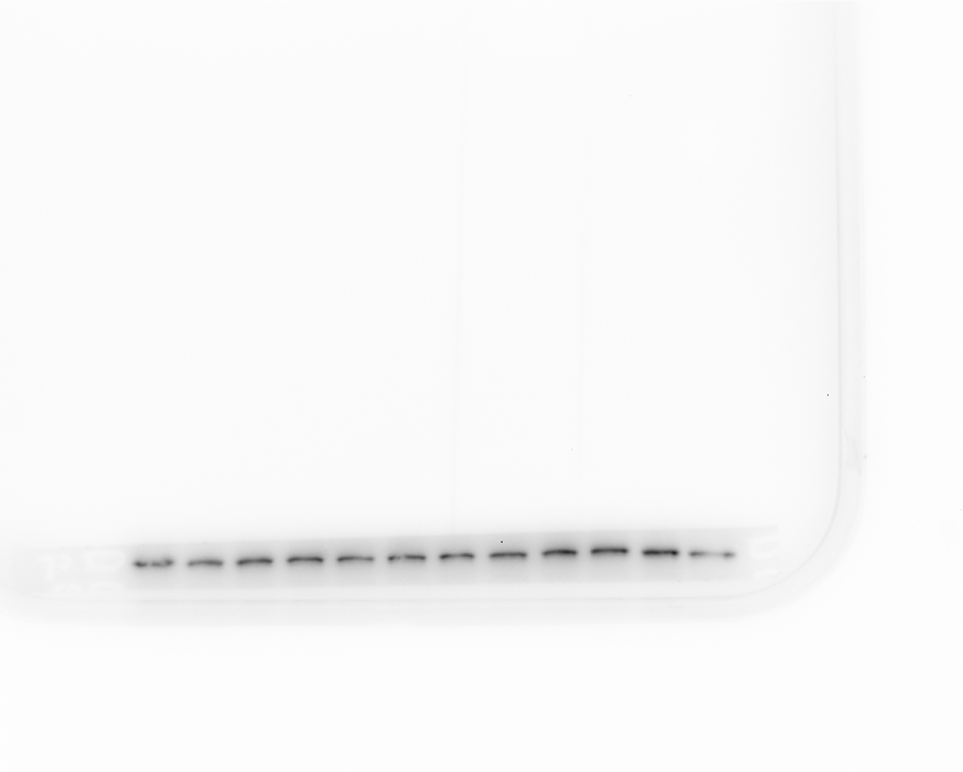

Supplement: Supplementary file 23 [file DataSheet6.ZIP › P65-1/p65 3.tif]

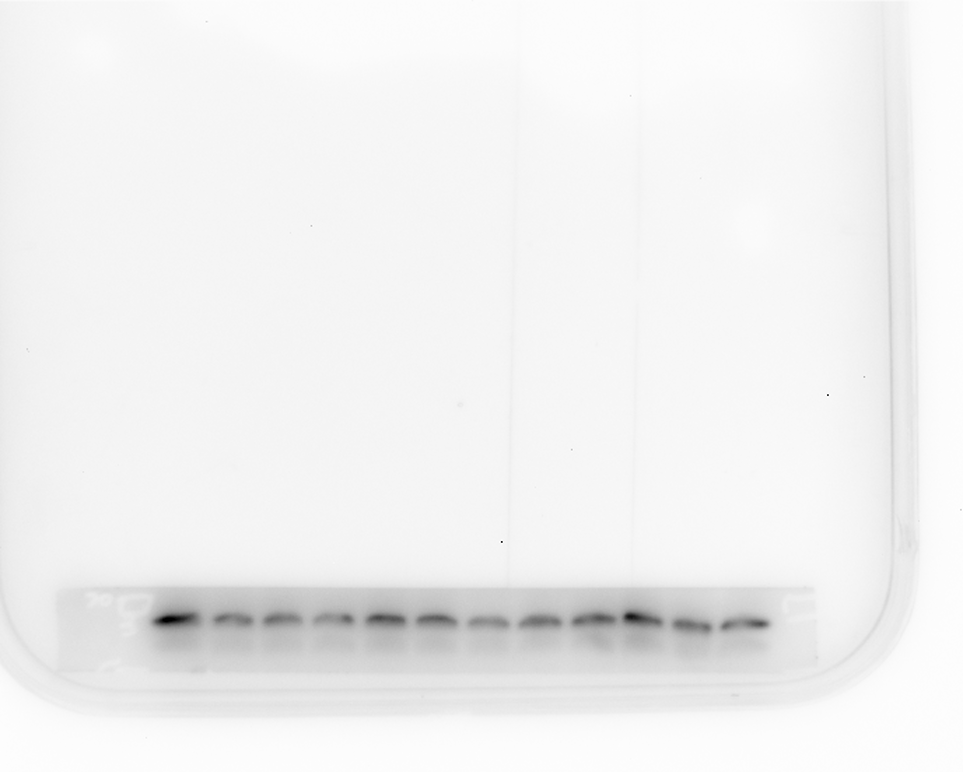

Supplement: Supplementary file 23 [file DataSheet6.ZIP › P65-1/p65 CYPB 1.tif]

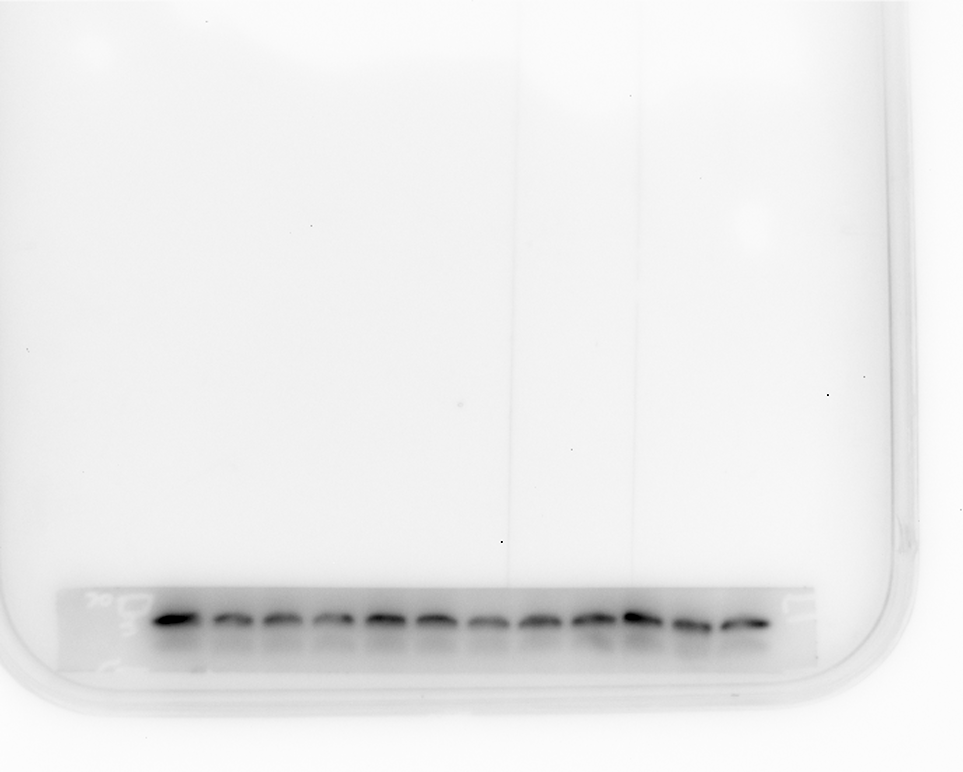

Supplement: Supplementary file 23 [file DataSheet6.ZIP › P65-1/p65 CYPB 2.tif]
