# Supplementary material for: Valsartan attenuates LPS-induced ALI by modulating NF-κB and MAPK pathways
Source: Front Pharmacol. 2024 Jan 15;15:1321095. doi: 10.3389/fphar.2024.1321095 (PMC10822936; doi:10.3389/fphar.2024.1321095)
Supplement: Supplementary file 13 [file DataSheet13.ZIP › Supplementary Table 1.docx]

Supplementary Material

Supplementary Table 1: List of primers and their sequences used in qPCR.

| Gene | Forward primer | Reverse primer |
| --- | --- | --- |
| Human-TNF-α | CTCTTCTGCCTGCTGCACTTTG | ATGGGCTACAGGCTTGTCACTC |
| Human-IL-6 | TGGTCTTTTGGAGTTTGAGGTA | AGGTTTCTGACCAGAAGAAGGA |
| Human-IL-1β | TCCCTTCATCTTTGAAGAAGA | GAGGCCCCAAGGCCACAGG |
| Human-MUC5AC | CCACTGGTTCTATGGCAACACC | GCCGAAGTCCAGGCTGTGCG |
| Human-GAPDH | CTGACTTCAACAGCGACACC | TGCTGTAGCCAAATTCGTTGT |
| Mouse-TNF-α | CAGGCGGTGCCTATGTCTC | CGATCACCCCGAAGTTCAGTAG |
| Mouse-IL-6 | GGCGGATCGGATGTTGTGAT | GGACCCCAGACAATCGGTTG |
| Mouse-IL-1β | GTGGCAGCTACCTGTGTCTT | GGAGCCTGTAGTGCAGTTGT |
| Mouse-CXCL-1 | CATGGCTGGGATTCACCTCA | CCTCGCGACCATTCTTGAGT |
| Mouse-CXCL-2 | TCAATGCCTGAAGACCCTGC | AGGCAAACTTTTTGACCGCC |
| IL-10 | CCCTTTGCTATGGTGTCCTT | TGGTTTCTCTTCCCAAGACC |
| Mouse-Actb | GTGACGTTGACATCCGTAAAGA | GCCGGACTCATCGTACTCC |
