# Supplementary material for: Valsartan attenuates LPS-induced ALI by modulating NF-κB and MAPK pathways
Source: Front Pharmacol. 2024 Jan 15;15:1321095. doi: 10.3389/fphar.2024.1321095 (PMC10822936; doi:10.3389/fphar.2024.1321095)
Supplement: Supplementary file 14 [file DataSheet1.ZIP › gating strategies.pptx]

## Slide 1
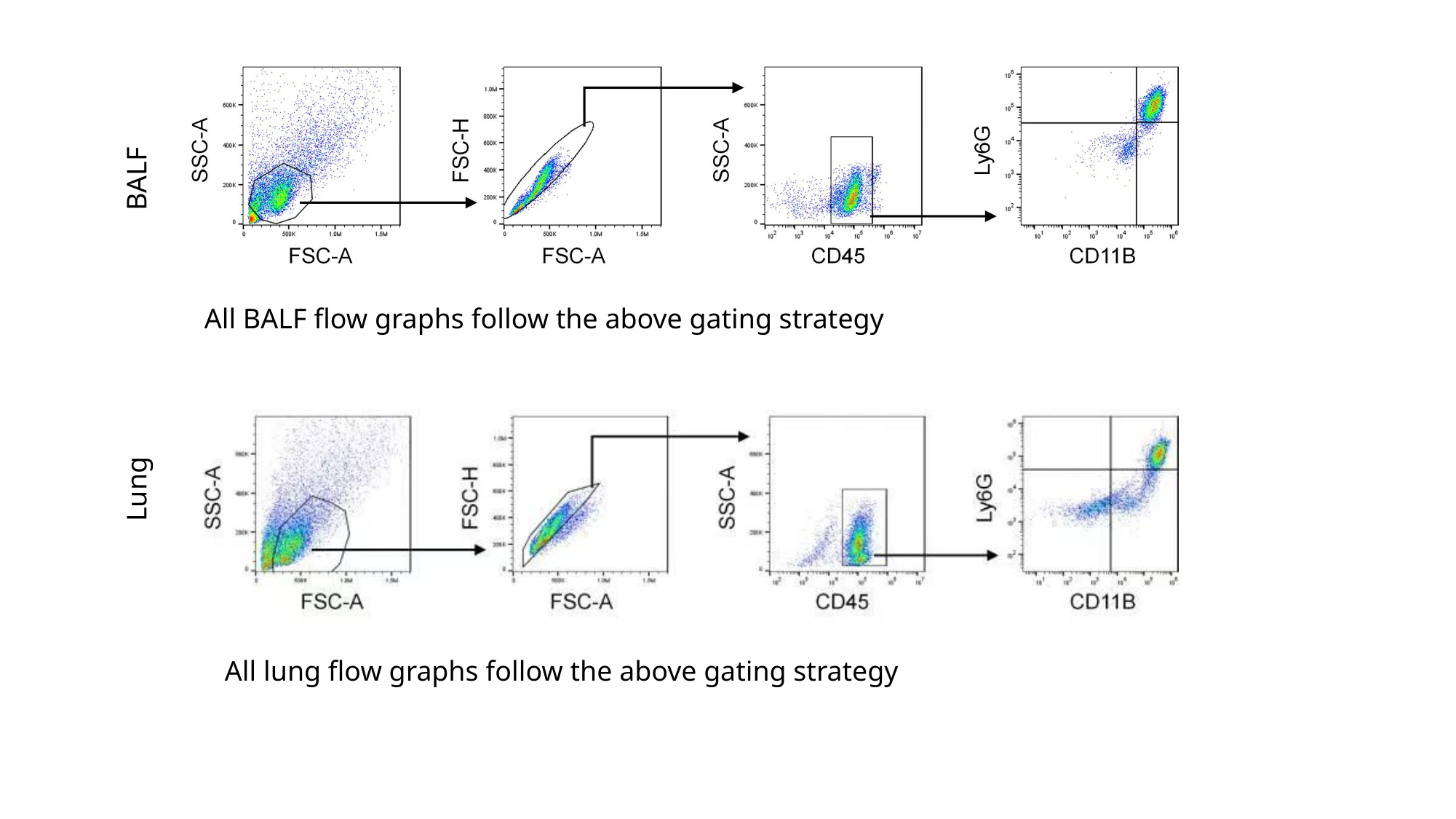

BALF
All BALF flow graphs follow the above gating strategy
Lung
All lung flow graphs follow the above gating strategy
