# Supplementary figures and images for: Valsartan attenuates LPS-induced ALI by modulating NF-κB and MAPK pathways (part 3 of 4)
Source: Front Pharmacol. 2024 Jan 15;15:1321095. doi: 10.3389/fphar.2024.1321095 (PMC10822936; doi:10.3389/fphar.2024.1321095)

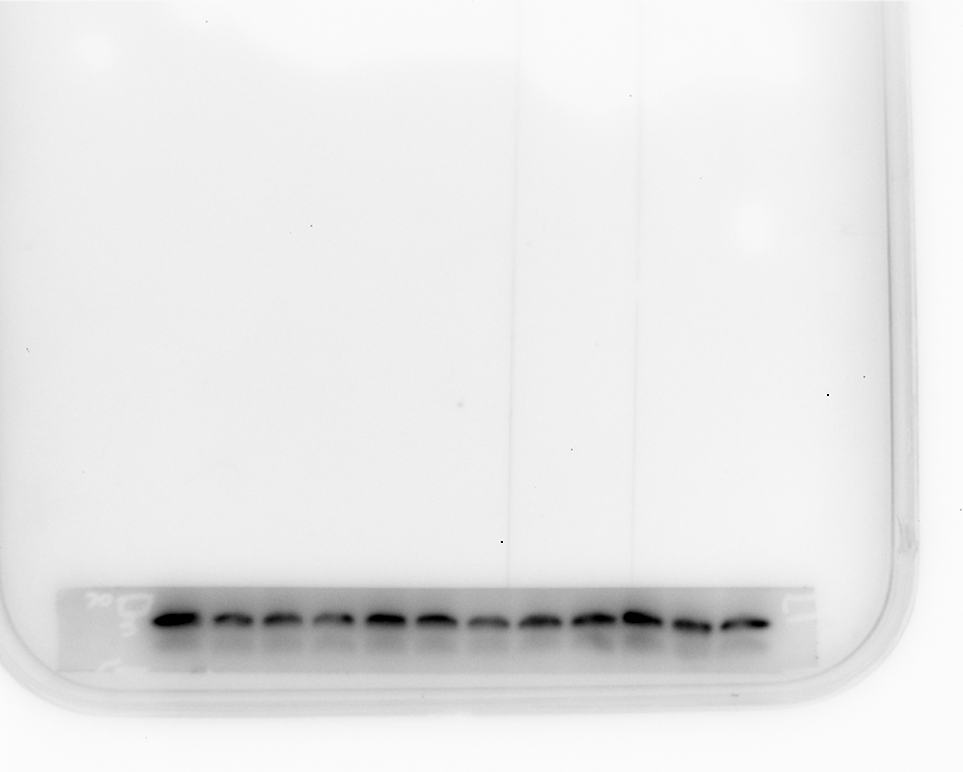

Supplement: Supplementary file 23 [file DataSheet6.ZIP › P65-1/p65 CYPB 4.tif]

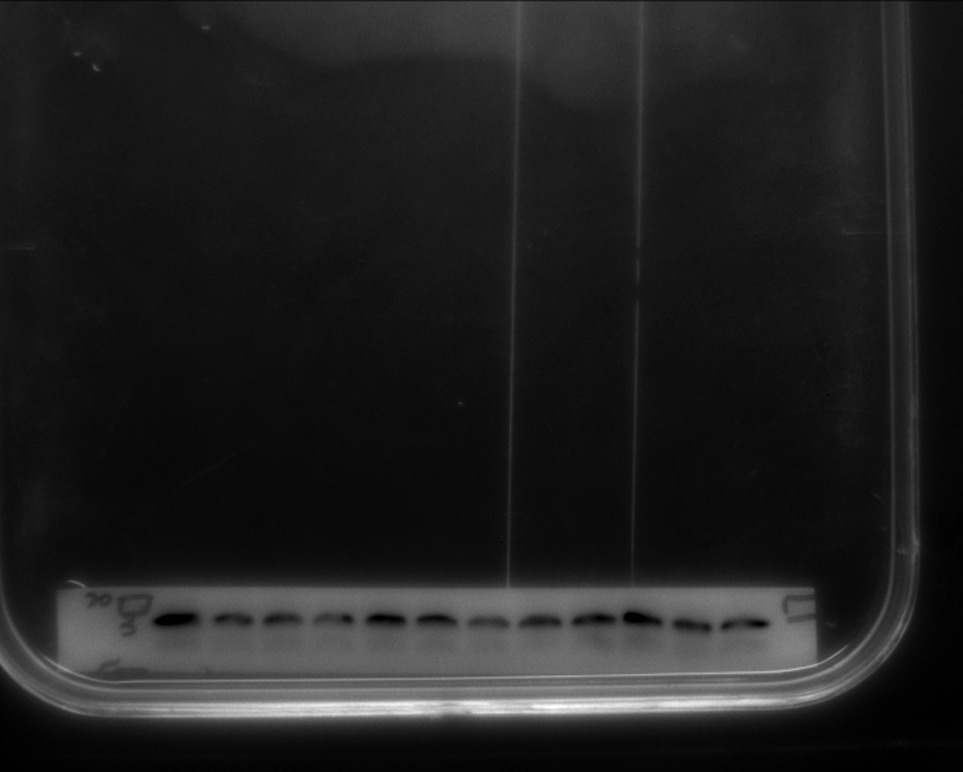

Supplement: Supplementary file 23 [file DataSheet6.ZIP › P65-1/p65 CYPB q.tif]

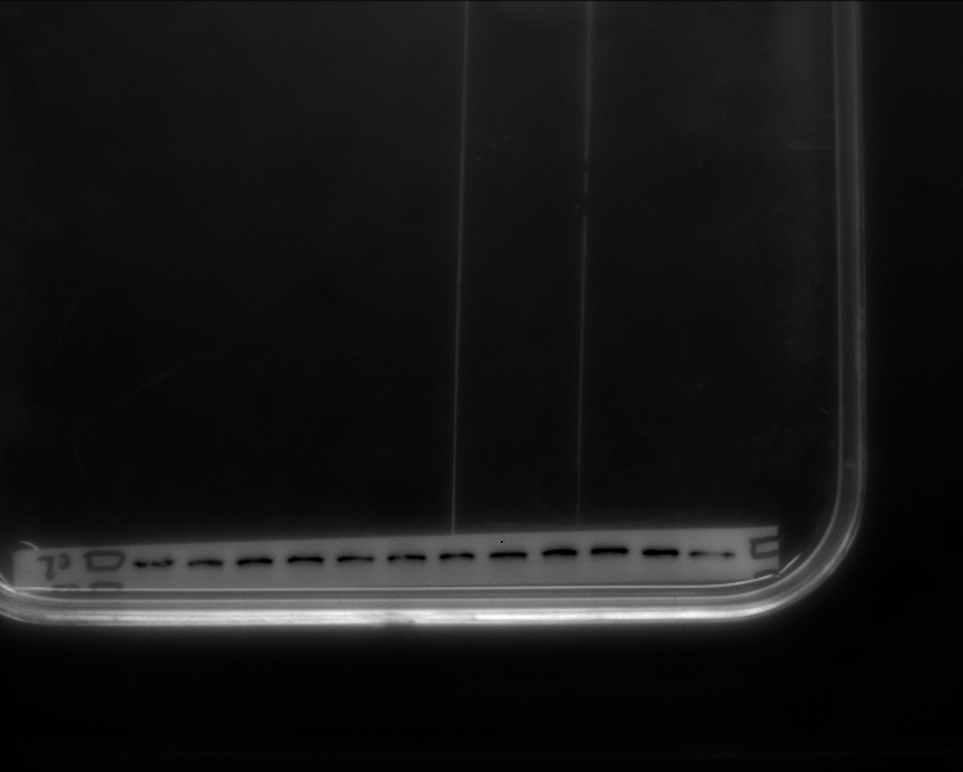

Supplement: Supplementary file 23 [file DataSheet6.ZIP › P65-1/p65 q.tif]

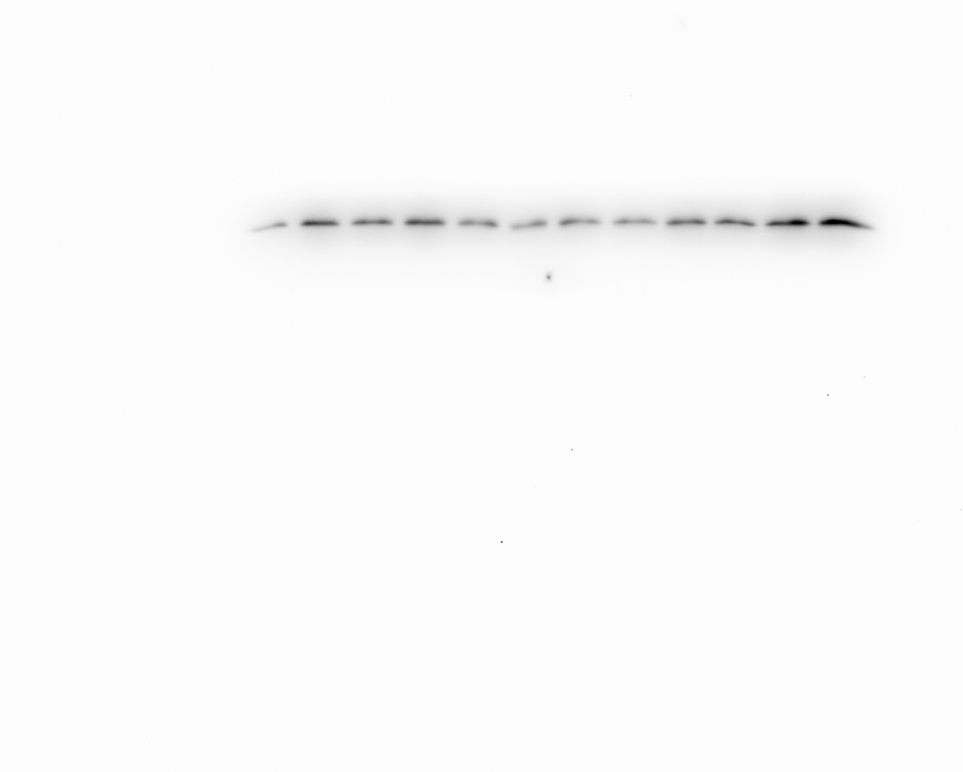

Supplement: Supplementary file 23 [file DataSheet6.ZIP › P65-2/CYPB1.tif]

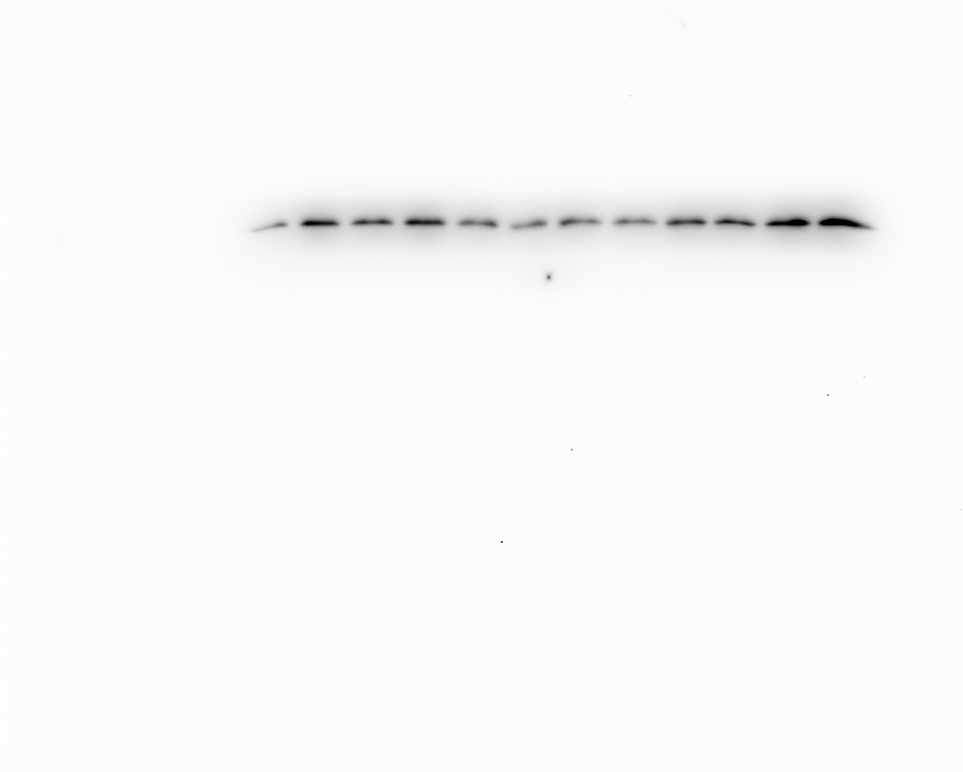

Supplement: Supplementary file 23 [file DataSheet6.ZIP › P65-2/CYPB2.tif]

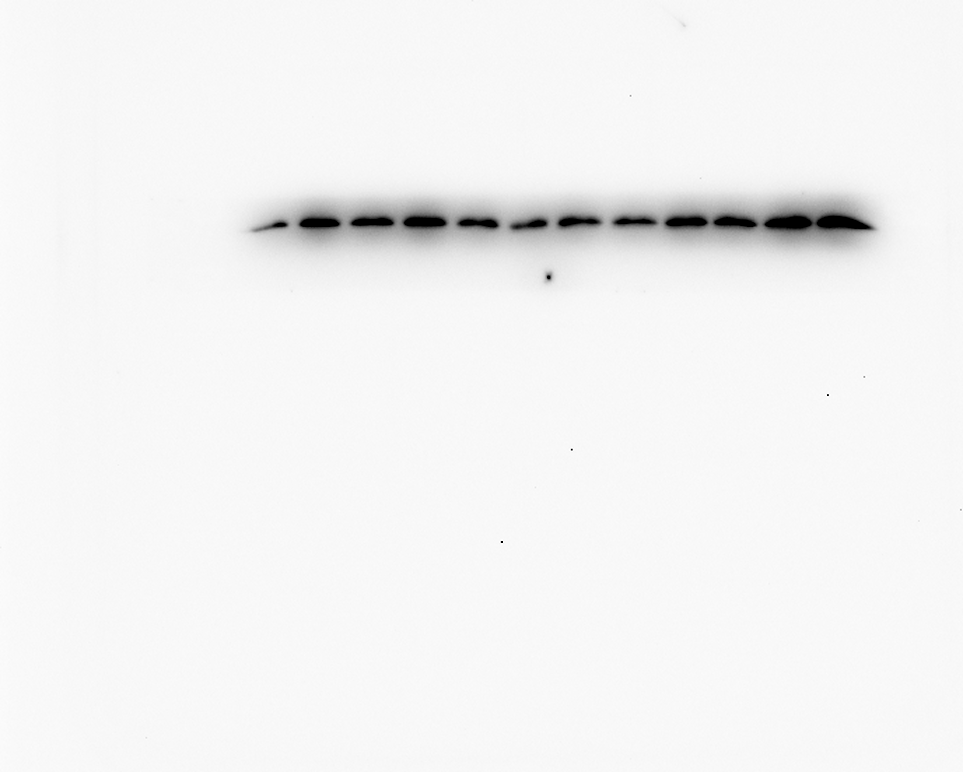

Supplement: Supplementary file 23 [file DataSheet6.ZIP › P65-2/CYPBgb.tif]

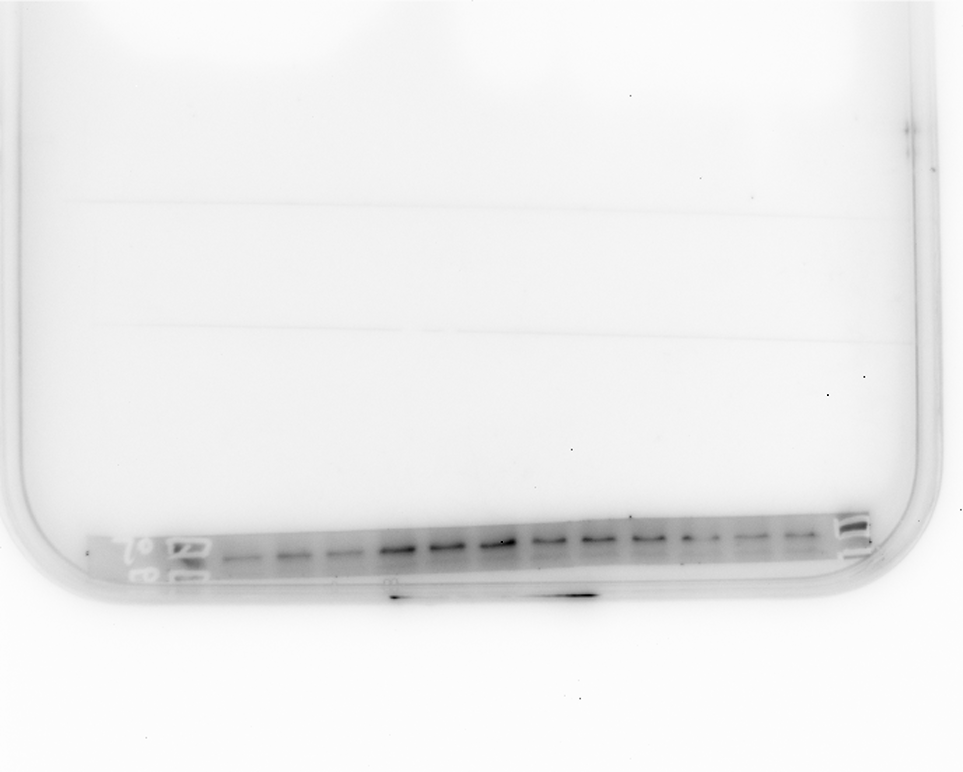

Supplement: Supplementary file 23 [file DataSheet6.ZIP › P65-2/p-p65 2.tif]

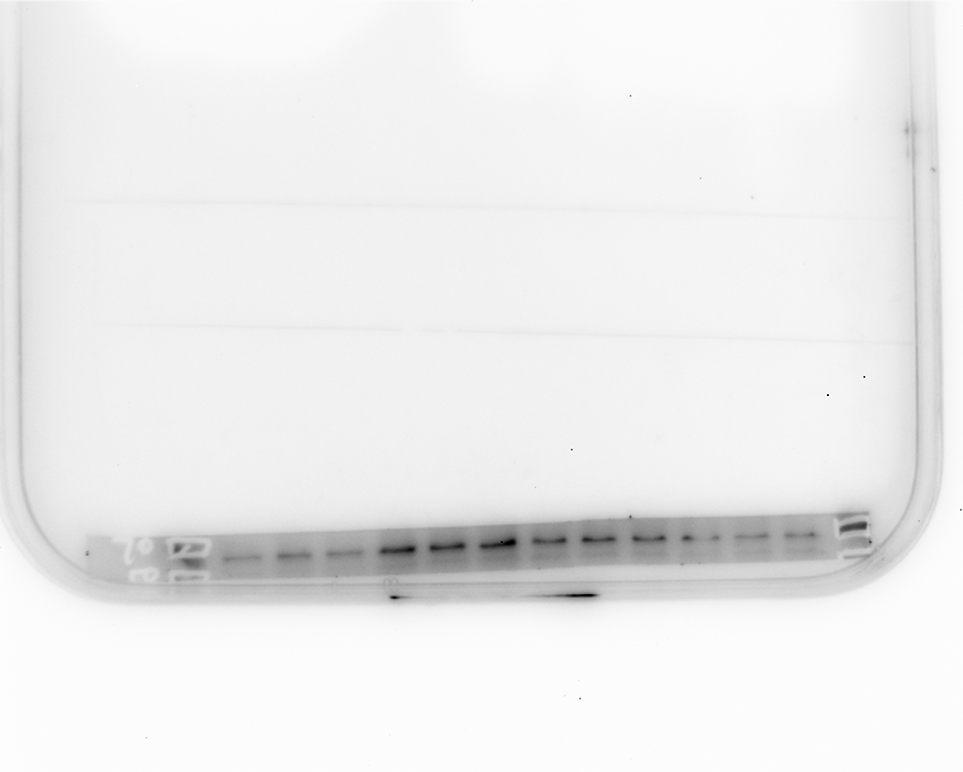

Supplement: Supplementary file 23 [file DataSheet6.ZIP › P65-2/p-p65 5.tif]

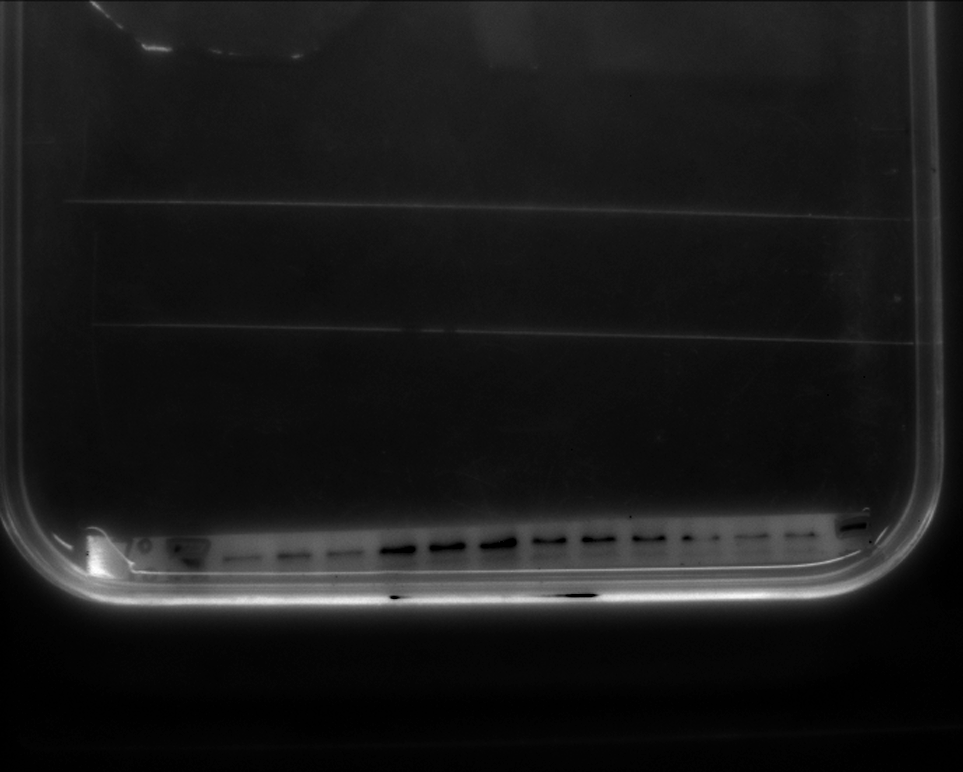

Supplement: Supplementary file 23 [file DataSheet6.ZIP › P65-2/p-p65 q.tif]

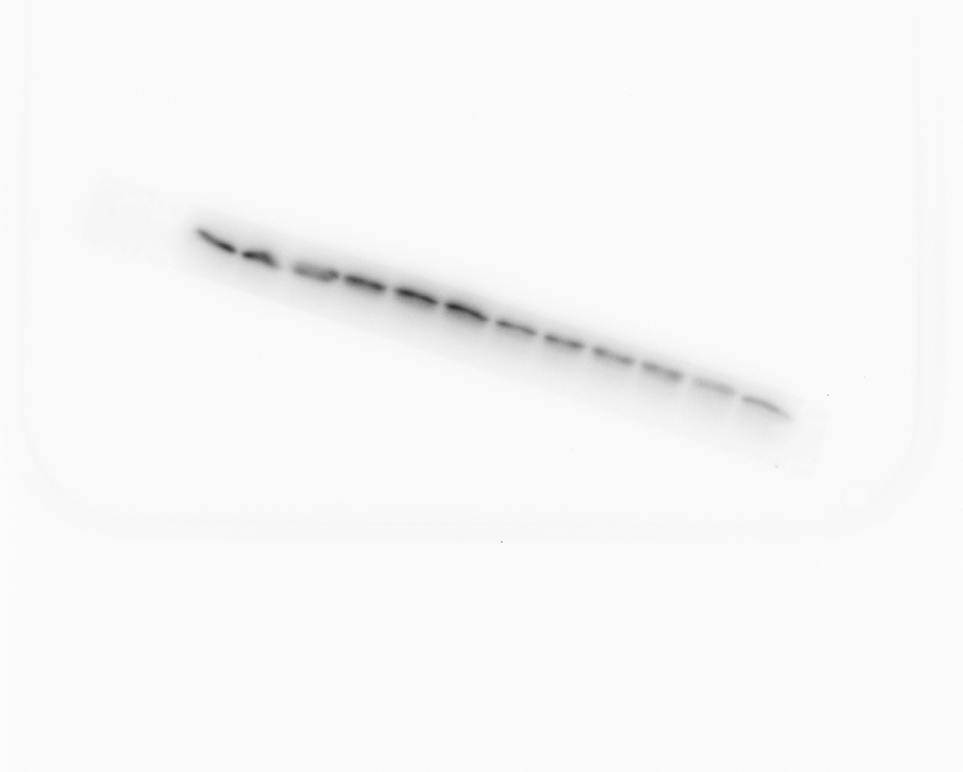

Supplement: Supplementary file 23 [file DataSheet6.ZIP › P65-2/p-p65CYPB 2.tif]

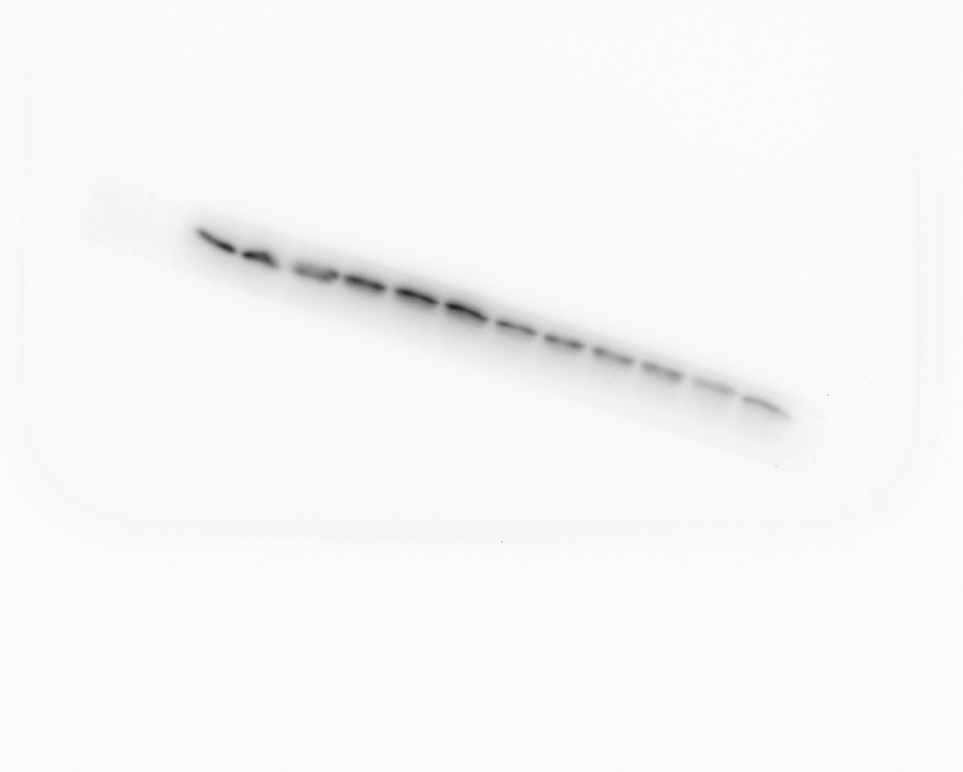

Supplement: Supplementary file 23 [file DataSheet6.ZIP › P65-2/p-p65CYPB 3.tif]

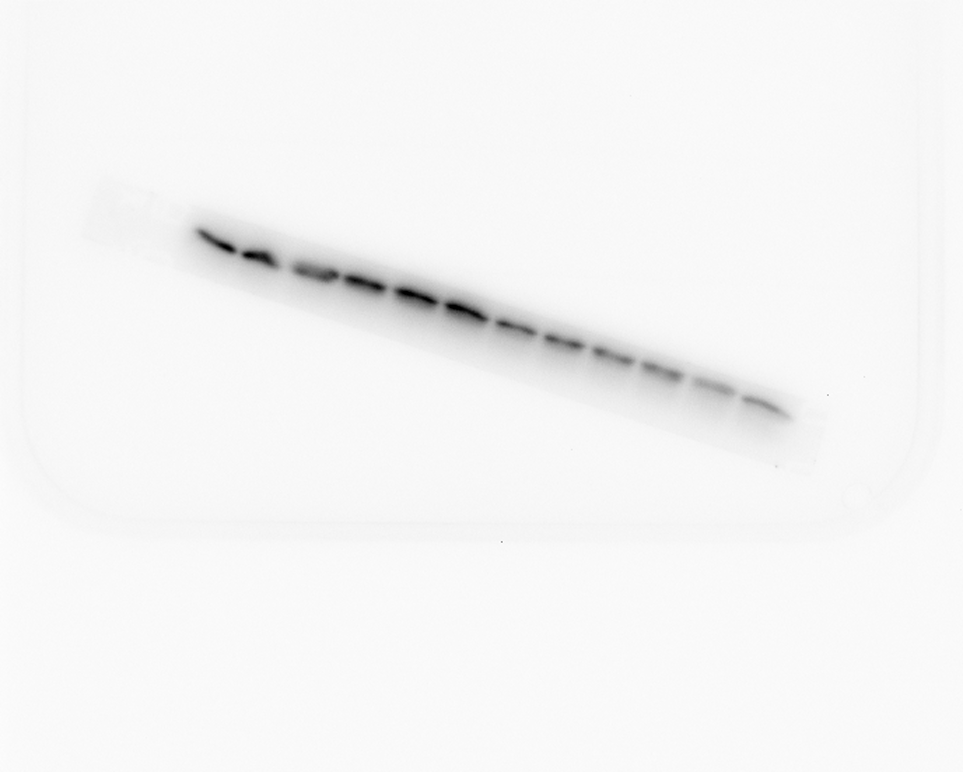

Supplement: Supplementary file 23 [file DataSheet6.ZIP › P65-2/p-p65CYPB.tif]

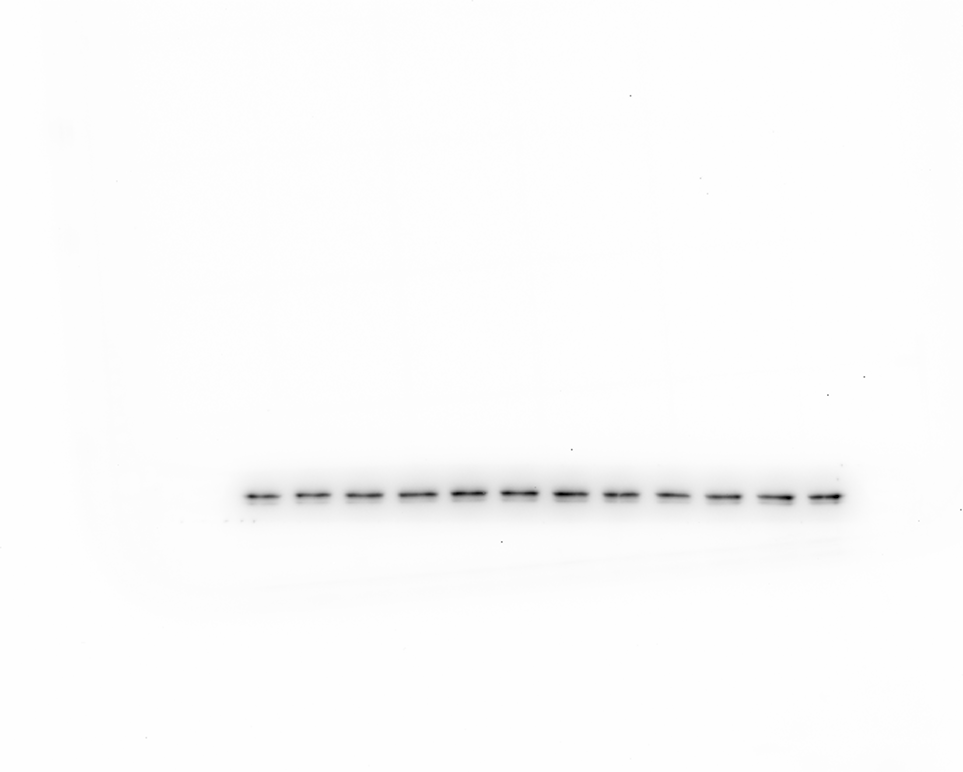

Supplement: Supplementary file 23 [file DataSheet6.ZIP › P65-2/p65 3.tif]

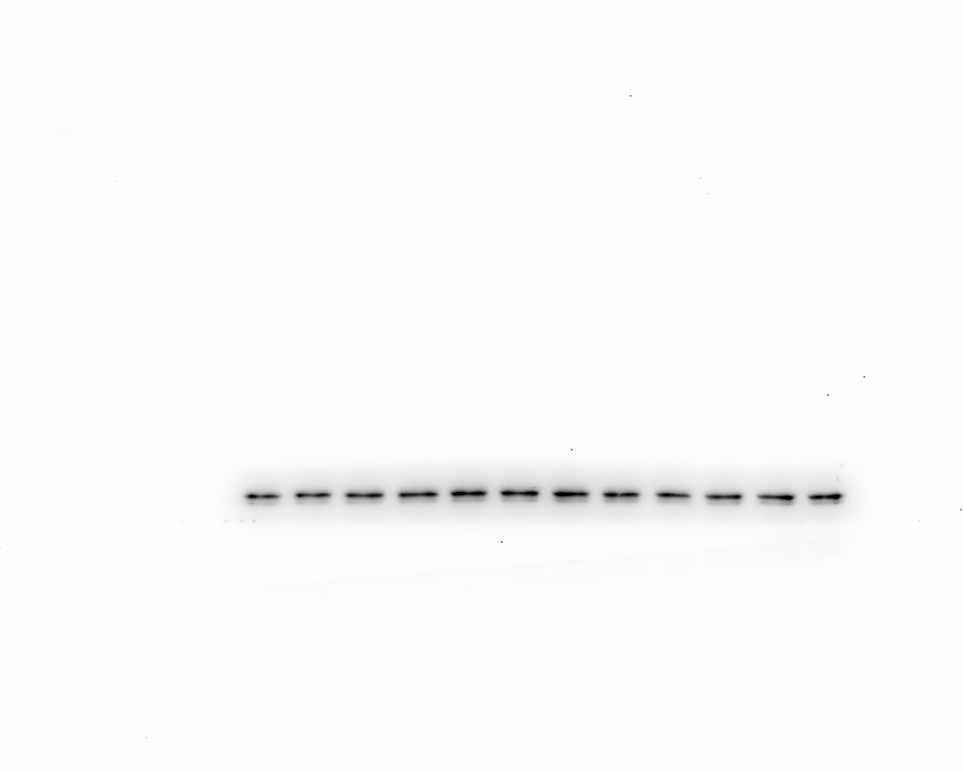

Supplement: Supplementary file 23 [file DataSheet6.ZIP › P65-2/p65 4.tif]

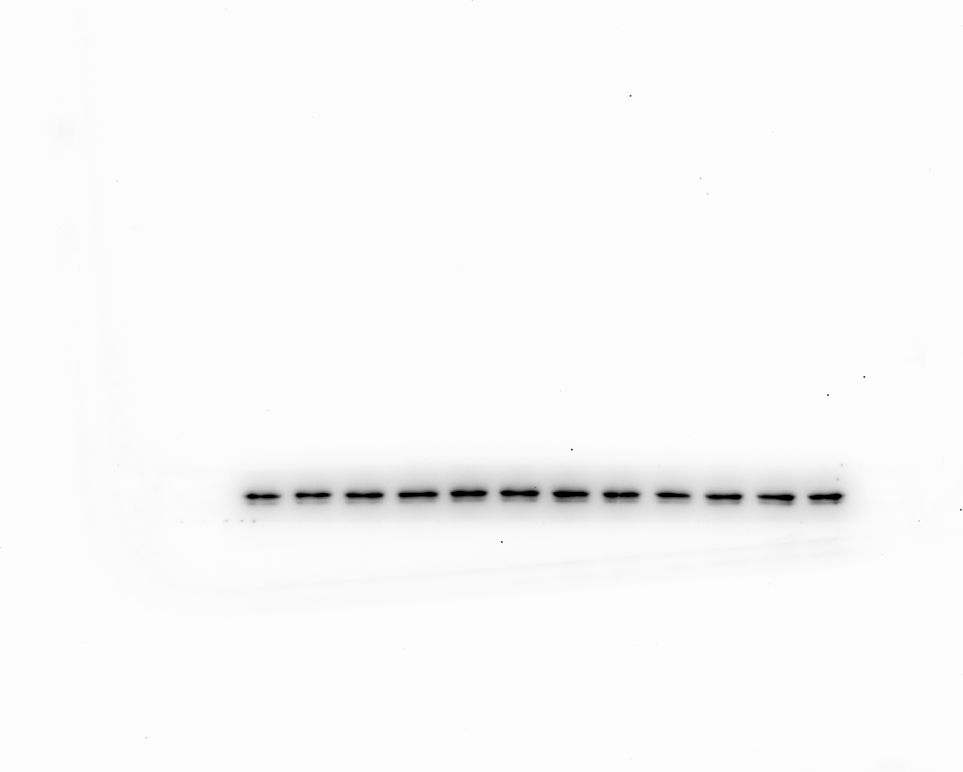

Supplement: Supplementary file 23 [file DataSheet6.ZIP › P65-2/p65 5.tif]

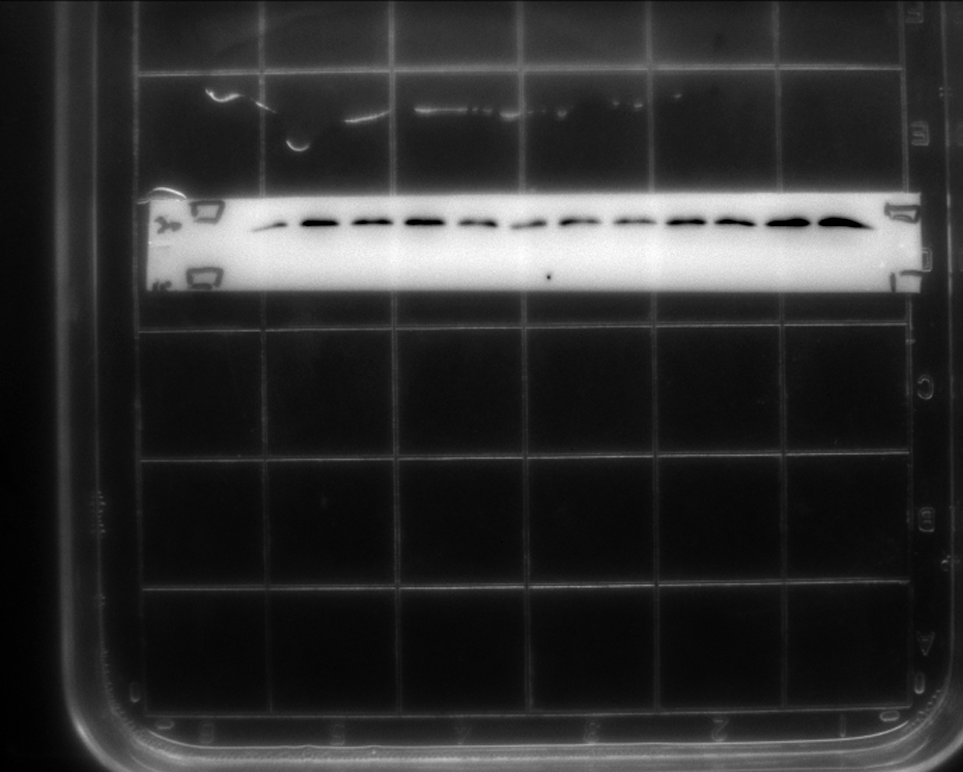

Supplement: Supplementary file 23 [file DataSheet6.ZIP › P65-2/p65 CYPBq.tif]

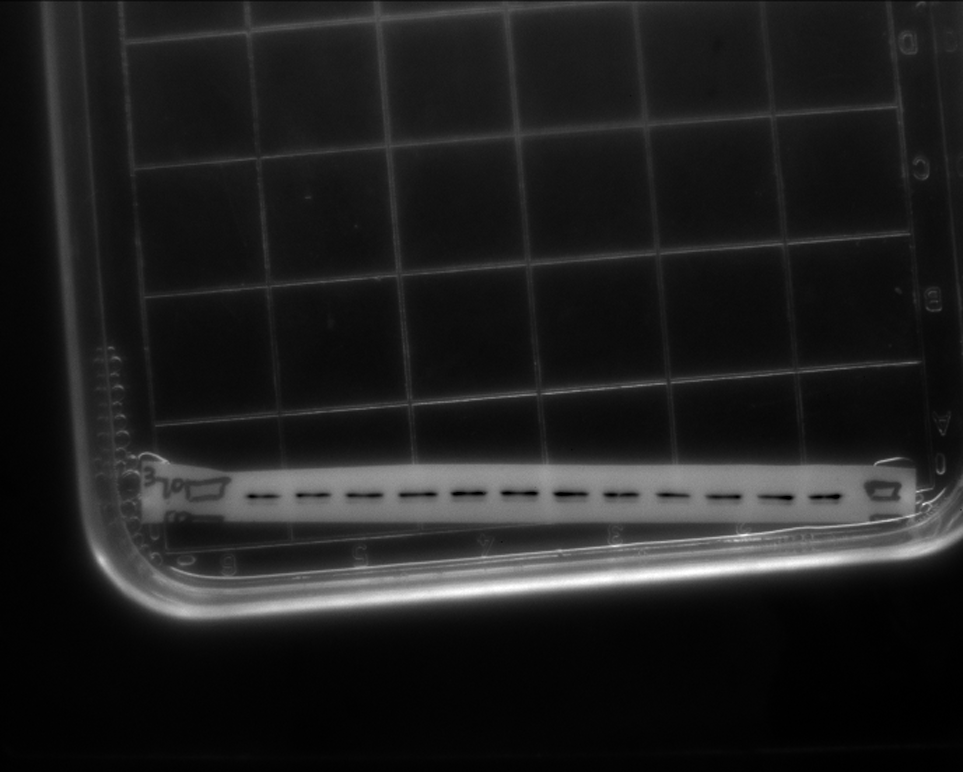

Supplement: Supplementary file 23 [file DataSheet6.ZIP › P65-2/p65 q 1.tif]

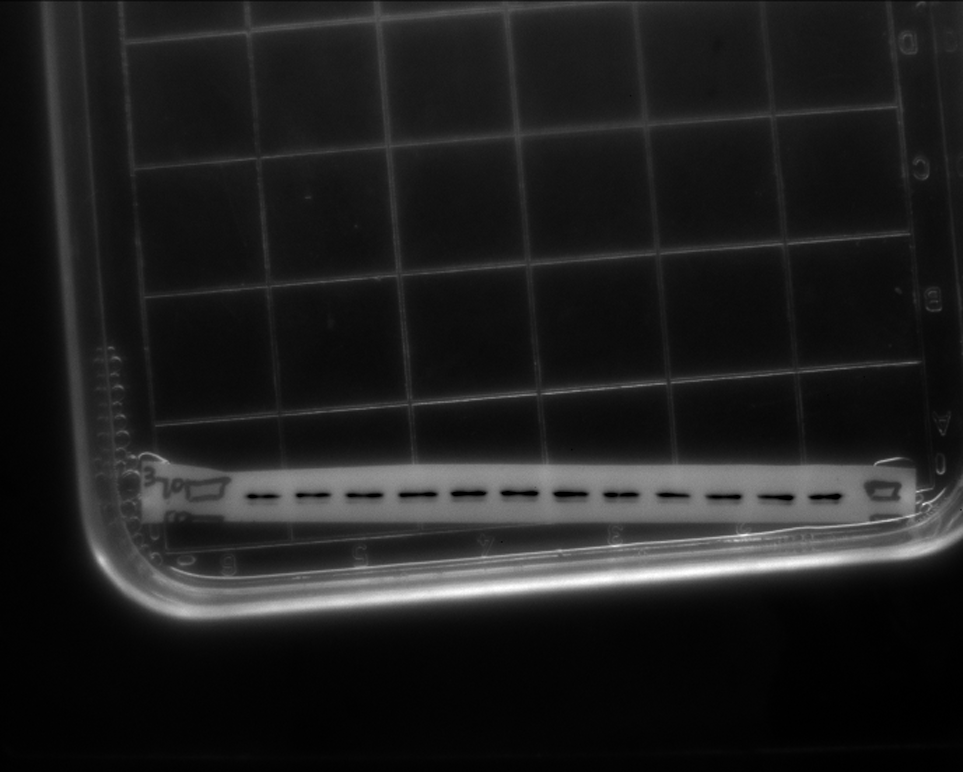

Supplement: Supplementary file 23 [file DataSheet6.ZIP › P65-2/p65 q.tif]

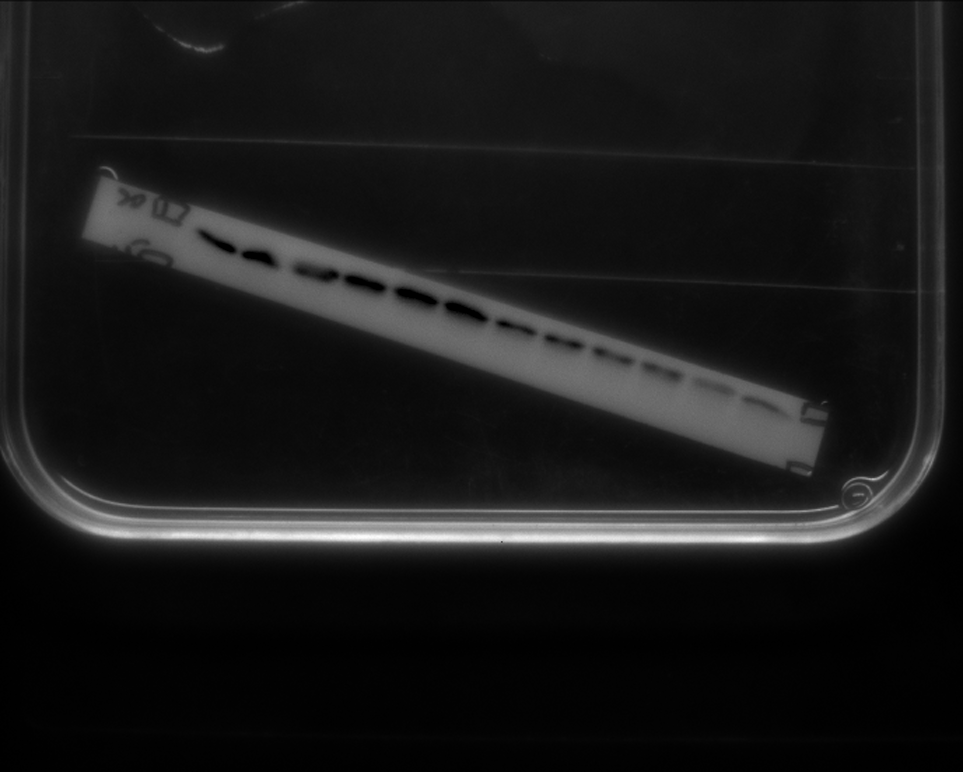

Supplement: Supplementary file 23 [file DataSheet6.ZIP › P65-2/pp65CYPB q.tif]

## Slide 1
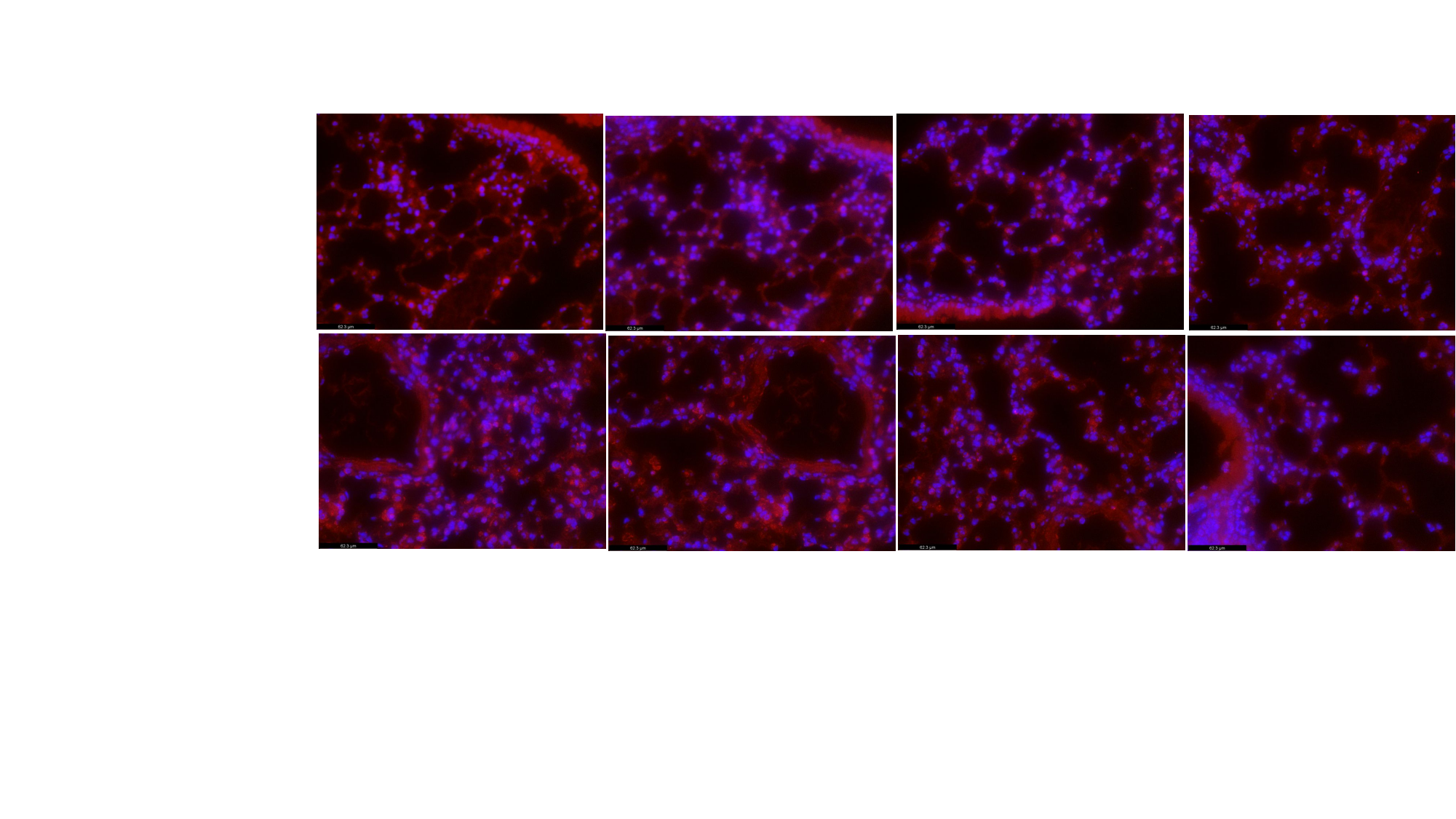

Supplement: Supplementary file 24 [file Presentation6.PPTX]

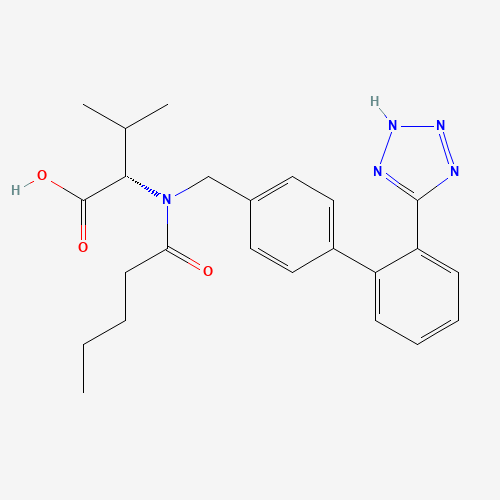

Supplement: Supplementary file 25 [file DataSheet12.ZIP › 1Obtaining the chemical structural of valsartan/Valsartan_500.png]

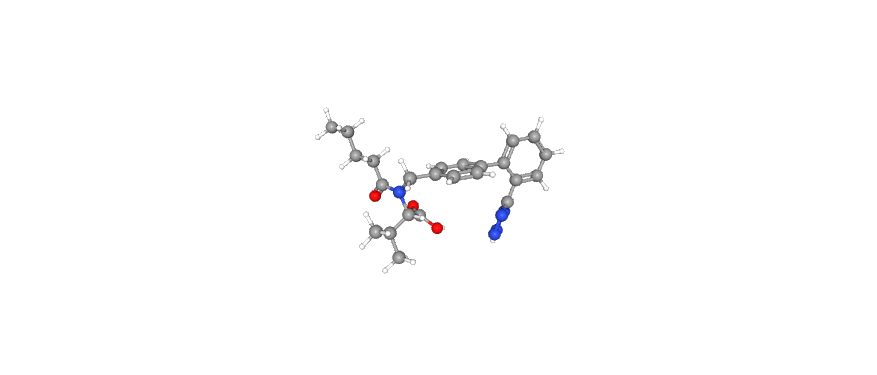

Supplement: Supplementary file 25 [file DataSheet12.ZIP › 1Obtaining the chemical structural of valsartan/Valsartan_877×375_3D_Conformer.png]

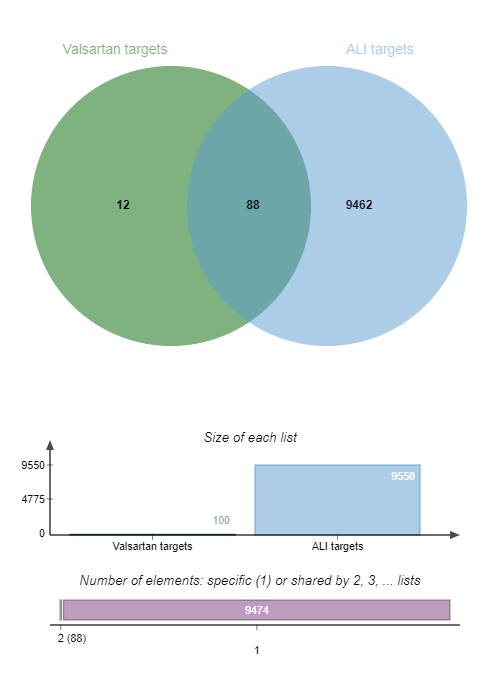

Supplement: Supplementary file 25 [file DataSheet12.ZIP › 4、ALI-Drug tagets/jVenn_chart.png]

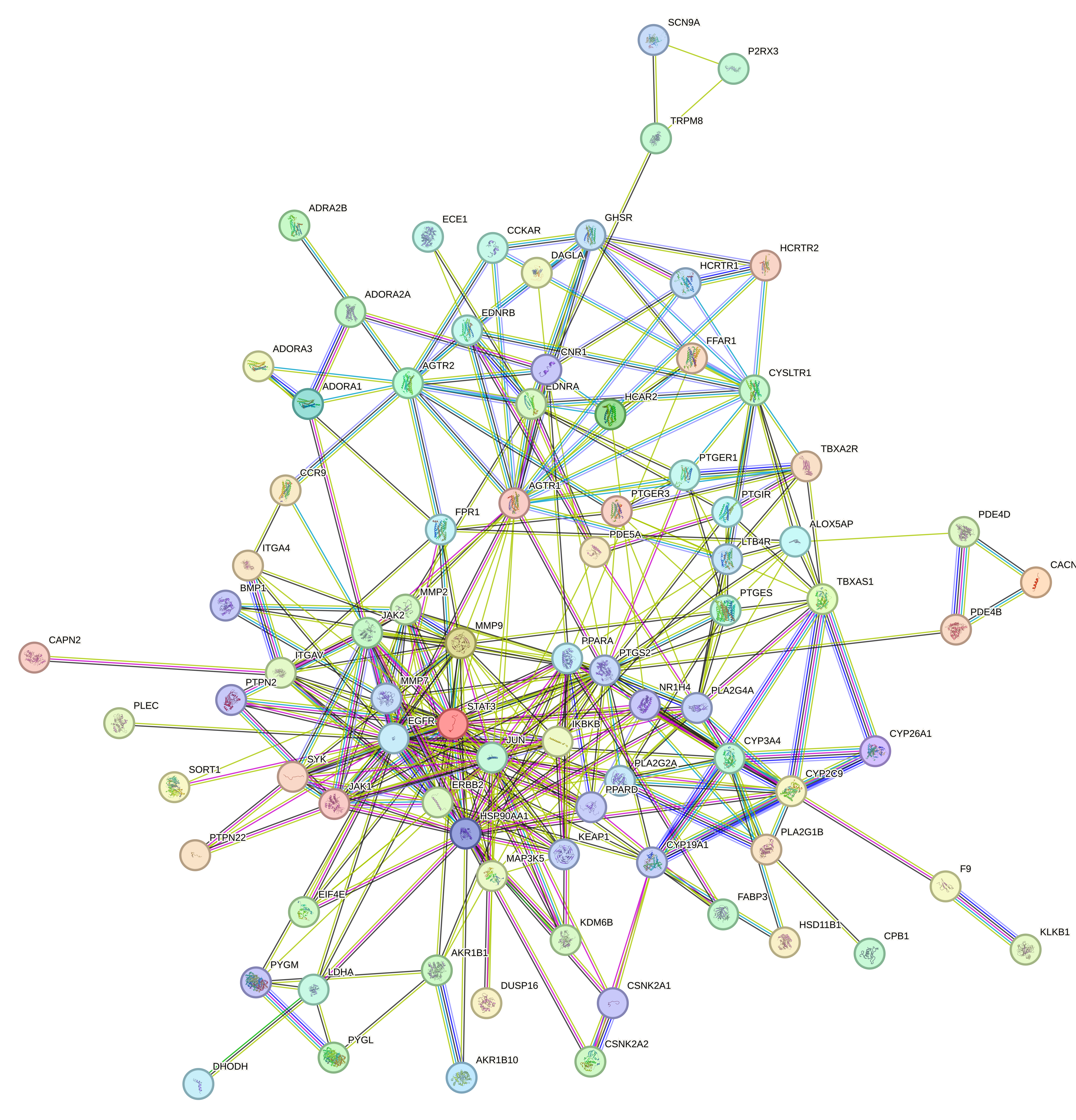

Supplement: Supplementary file 25 [file DataSheet12.ZIP › 5、String/string_hires_image (3).png]

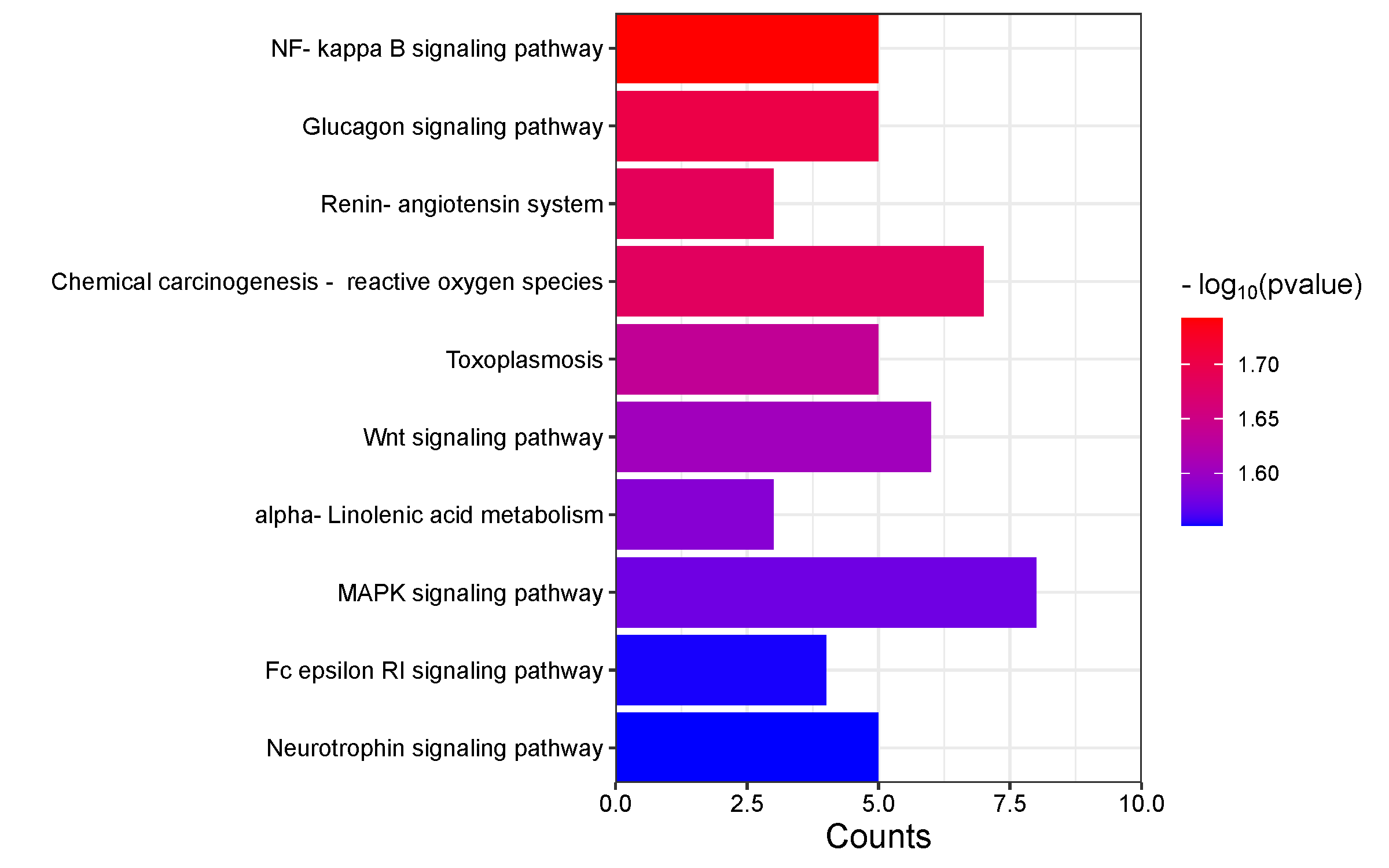

Supplement: Supplementary file 25 [file DataSheet12.ZIP › 6、KEGG/1b5b9f9c5e89fddf.png]

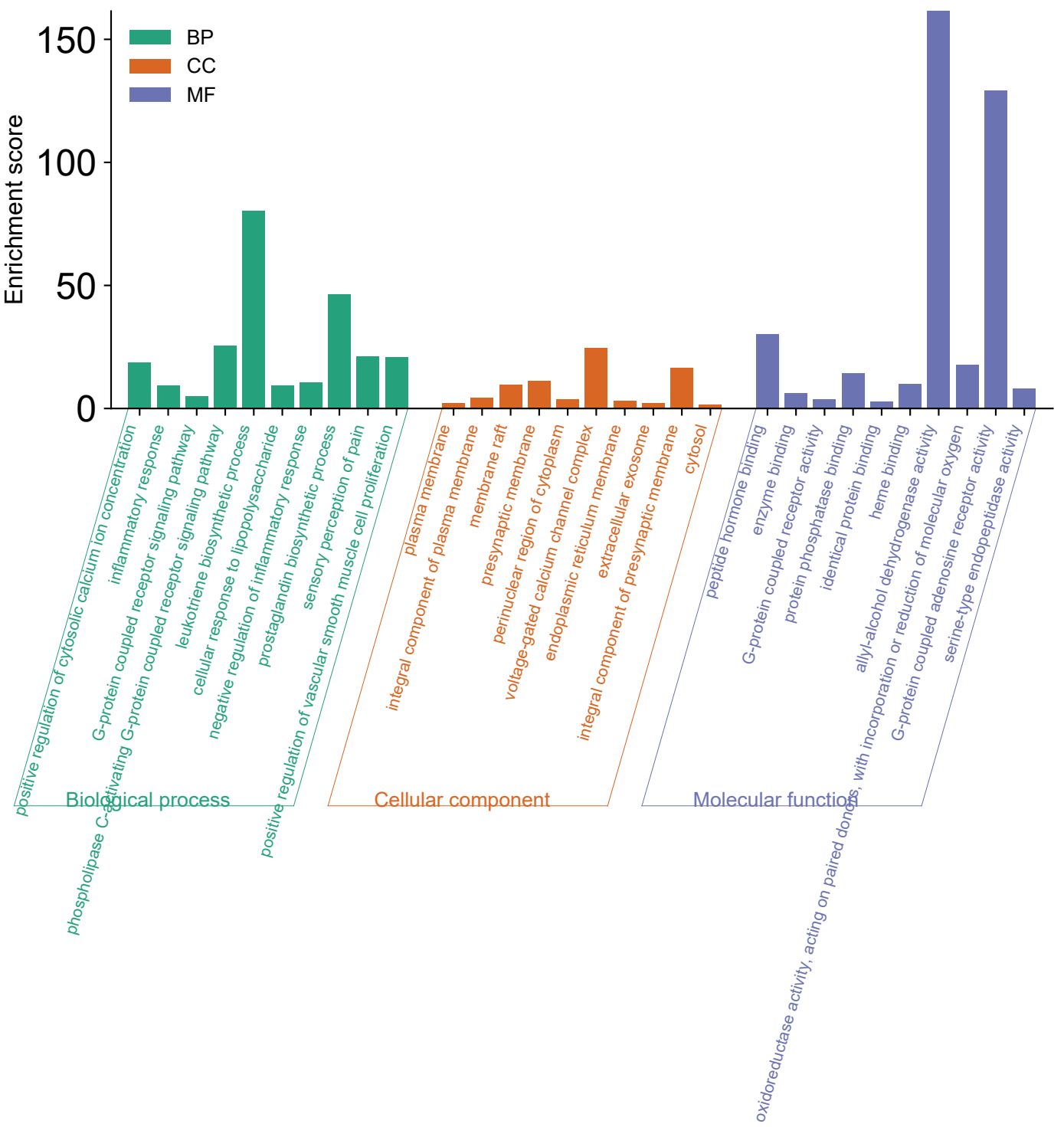

Supplement: Supplementary file 25 [file DataSheet12.ZIP › 7GO/419843203a79ce25.pdf]

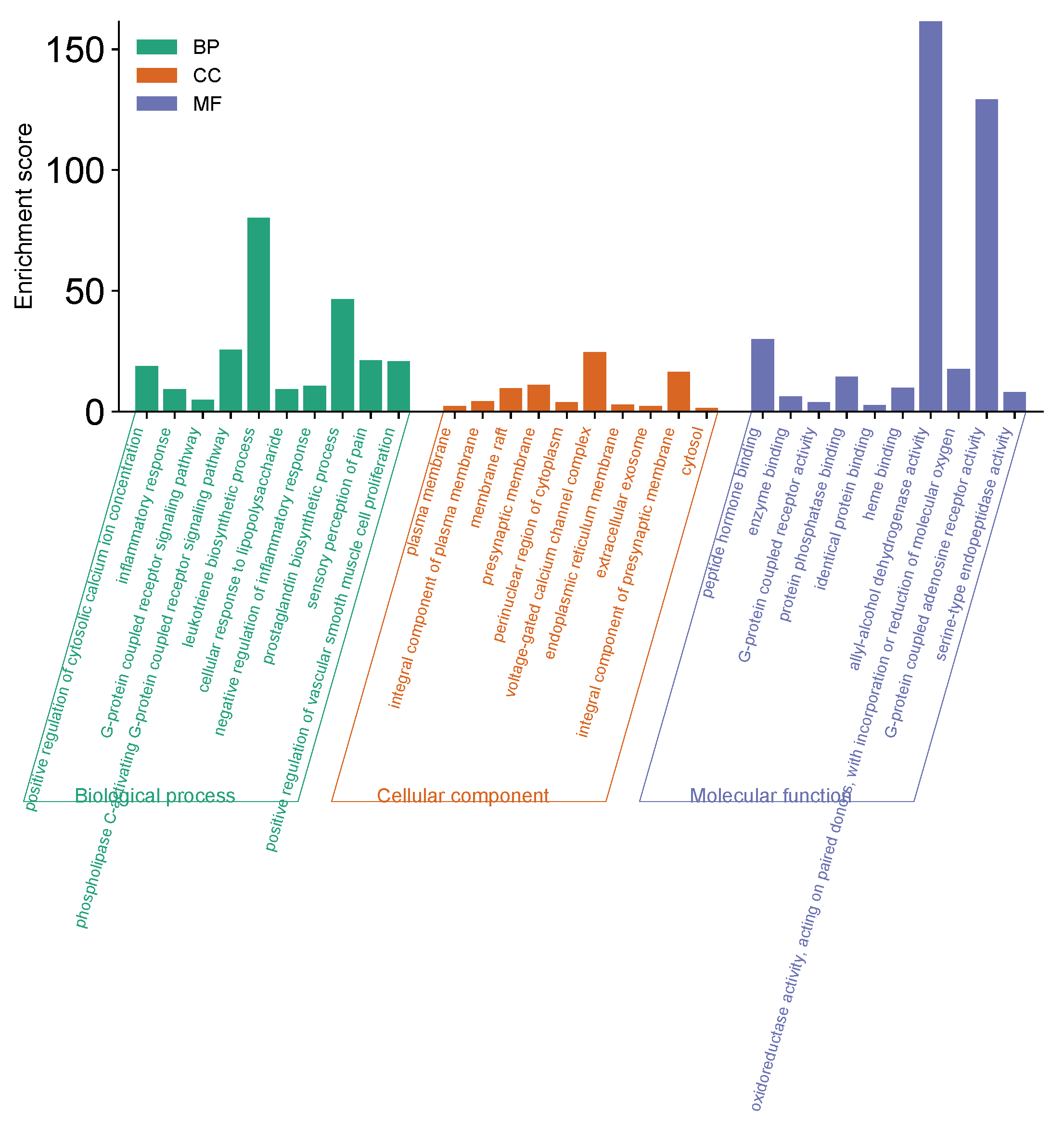

Supplement: Supplementary file 25 [file DataSheet12.ZIP › 7GO/419843203a79ce25.png]

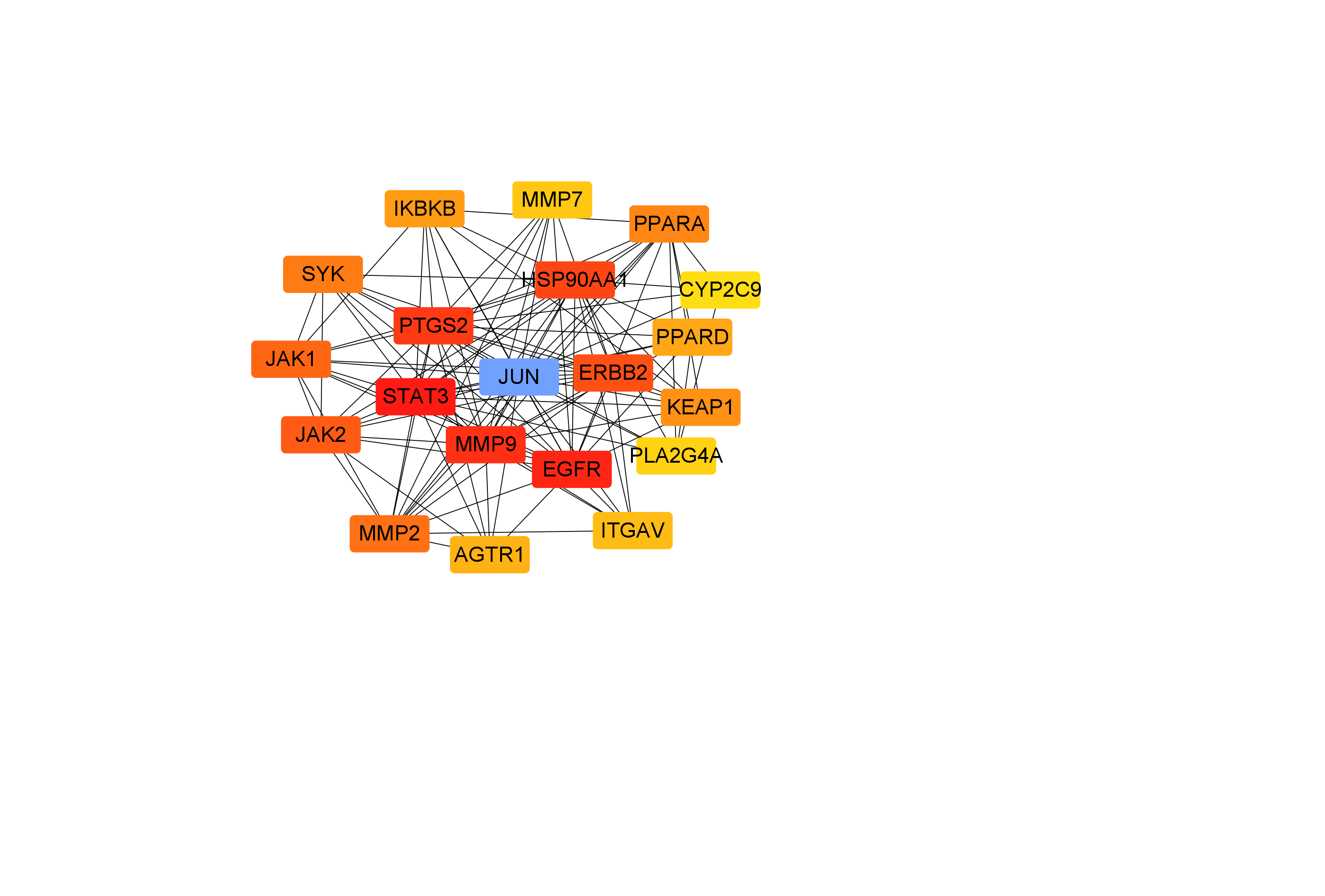

Supplement: Supplementary file 25 [file DataSheet12.ZIP › 8.cytoscape/cytohubba 20.png]

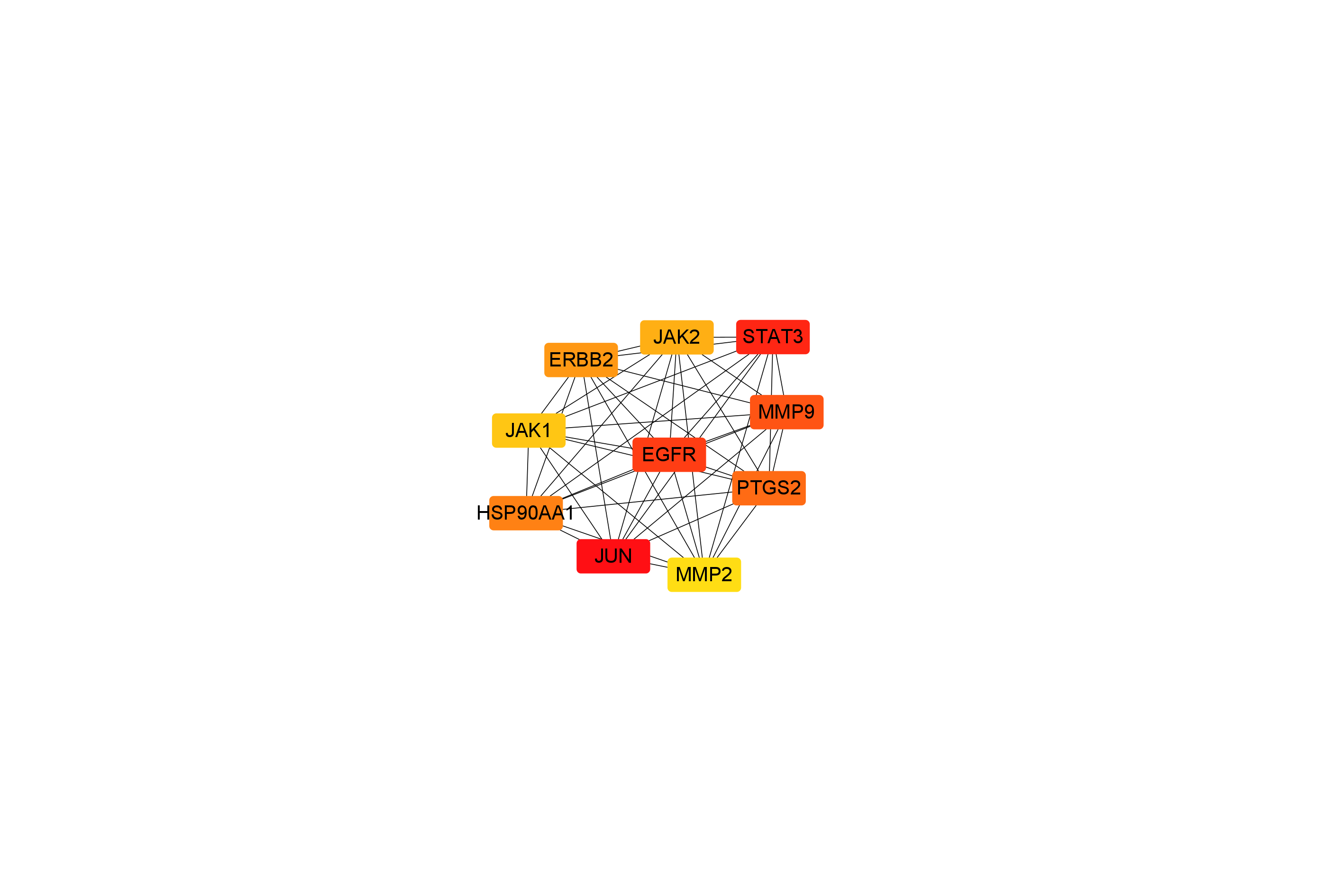

Supplement: Supplementary file 25 [file DataSheet12.ZIP › 8.cytoscape/cytohubba.png]

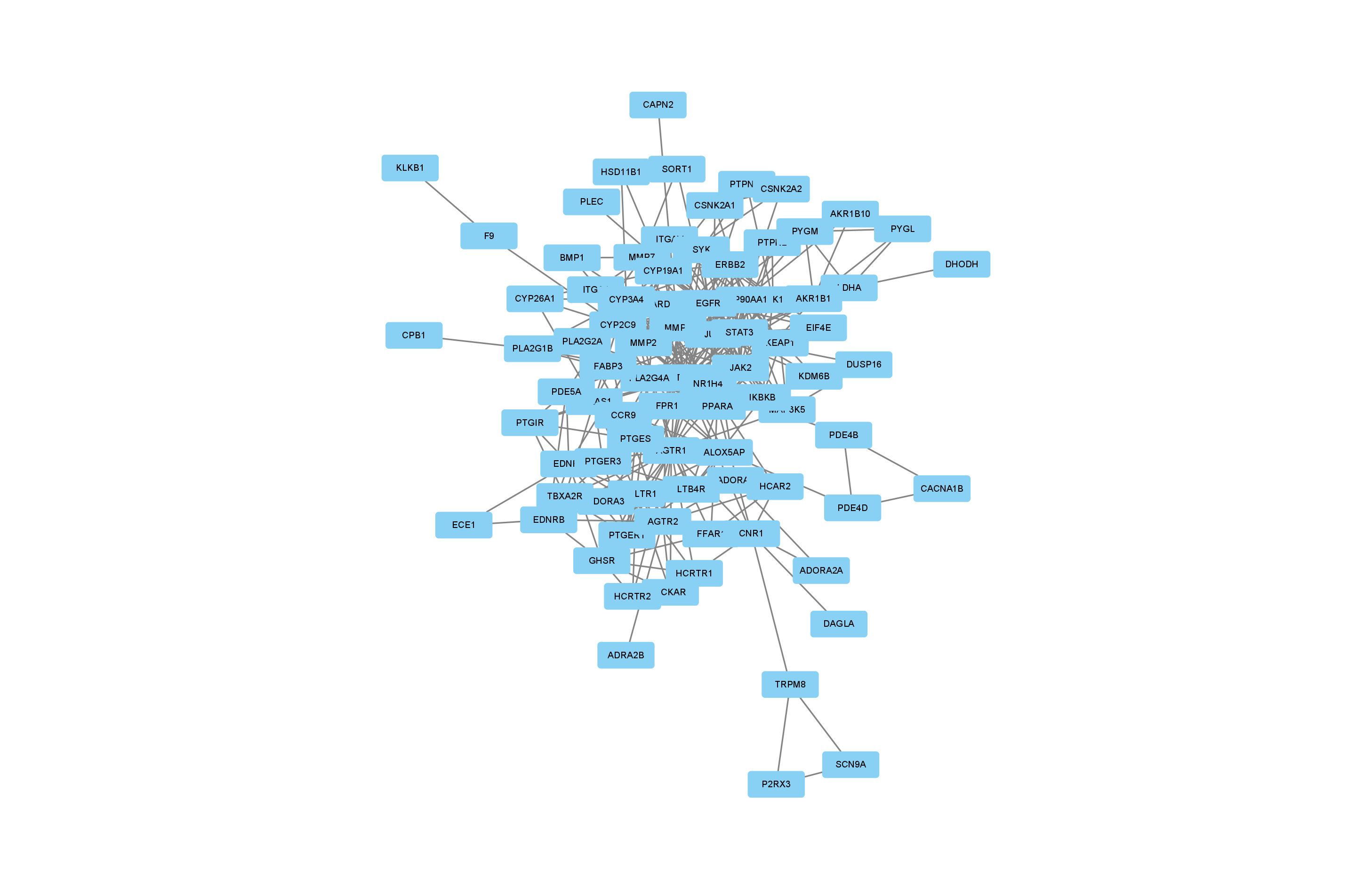

Supplement: Supplementary file 25 [file DataSheet12.ZIP › 8.cytoscape/string 图.png]

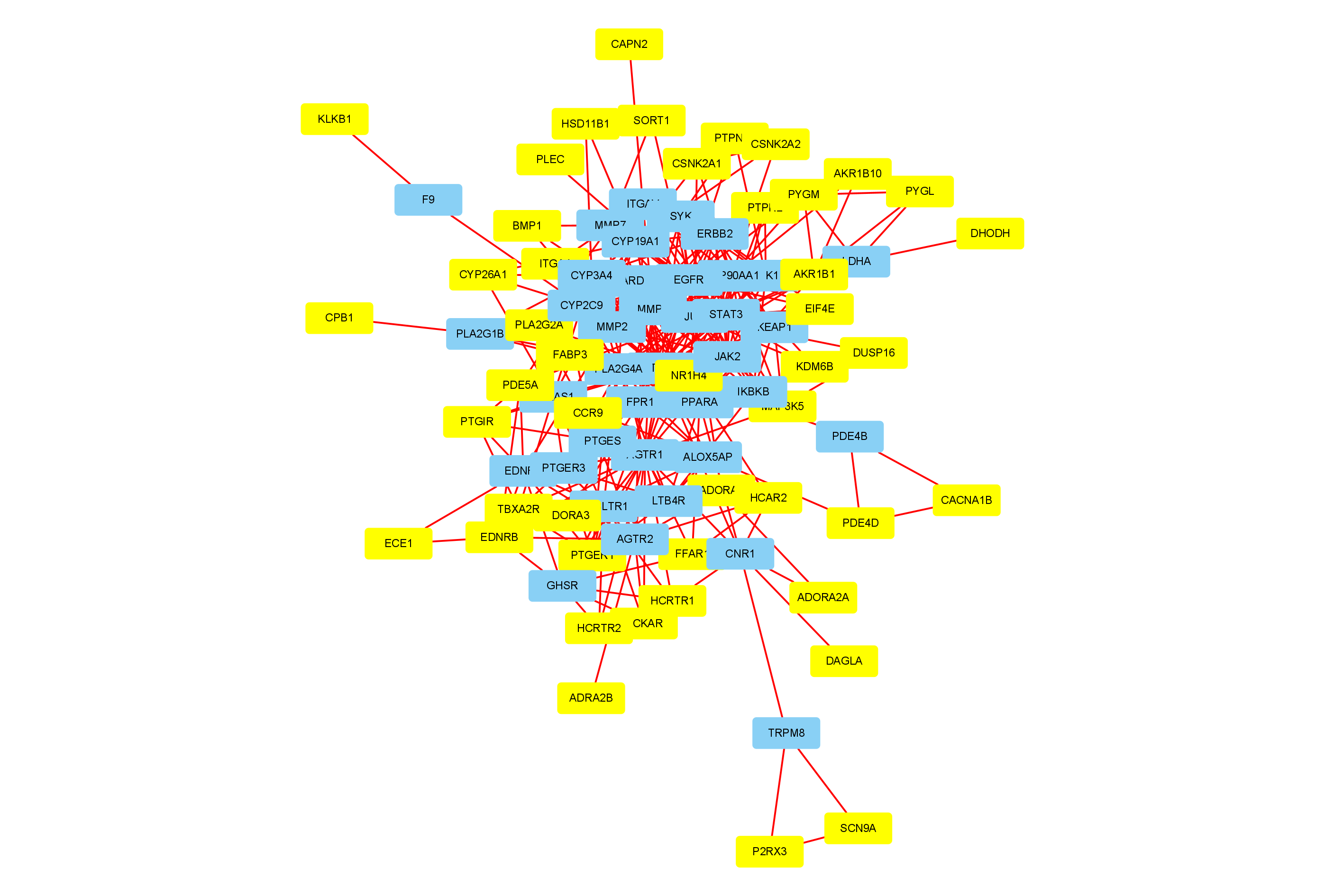

Supplement: Supplementary file 25 [file DataSheet12.ZIP › 8.cytoscape/string_interactions_short%20(2).tsv.png]

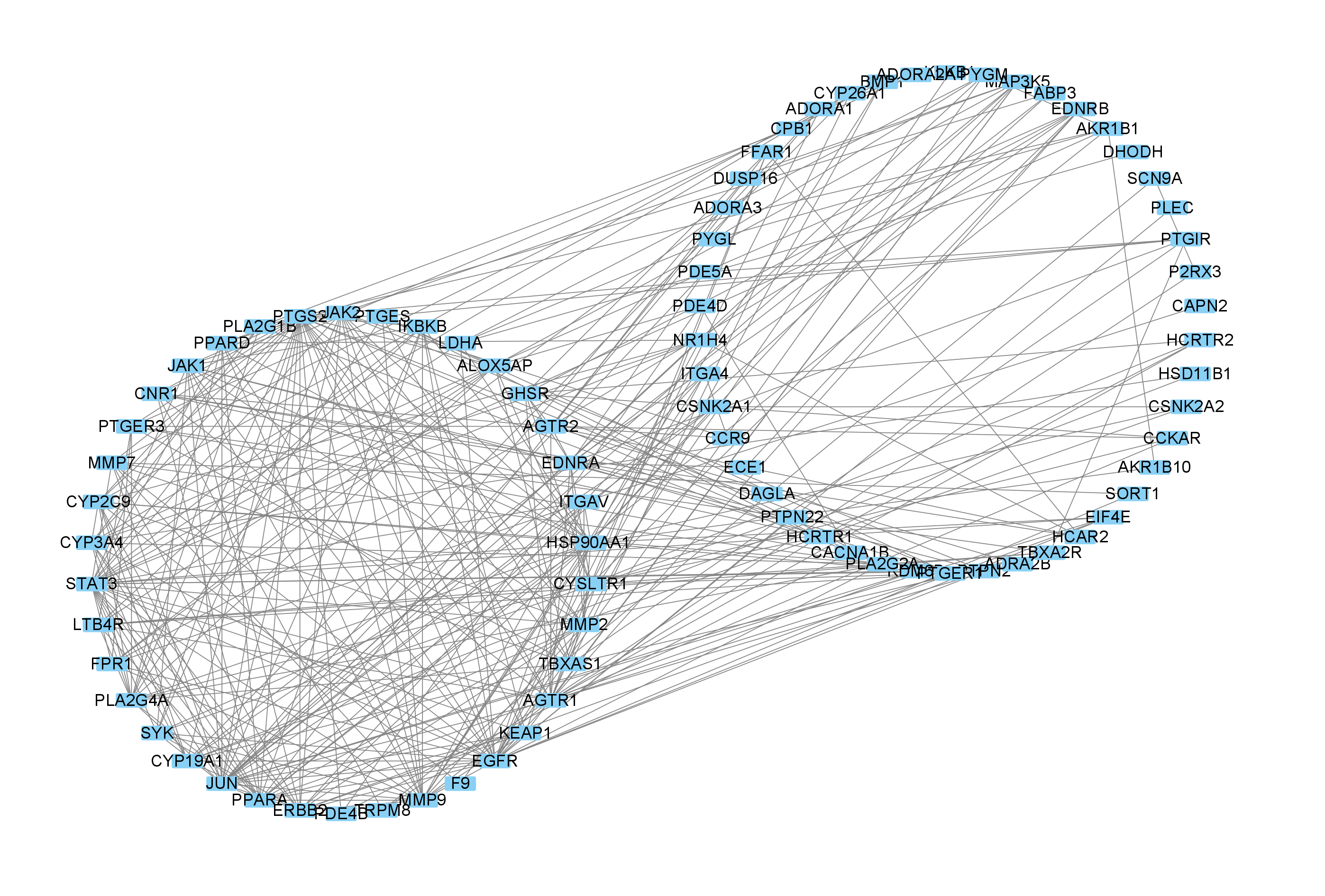

Supplement: Supplementary file 25 [file DataSheet12.ZIP › 8.cytoscape/string_interactions_short%20(3).tsv.png]

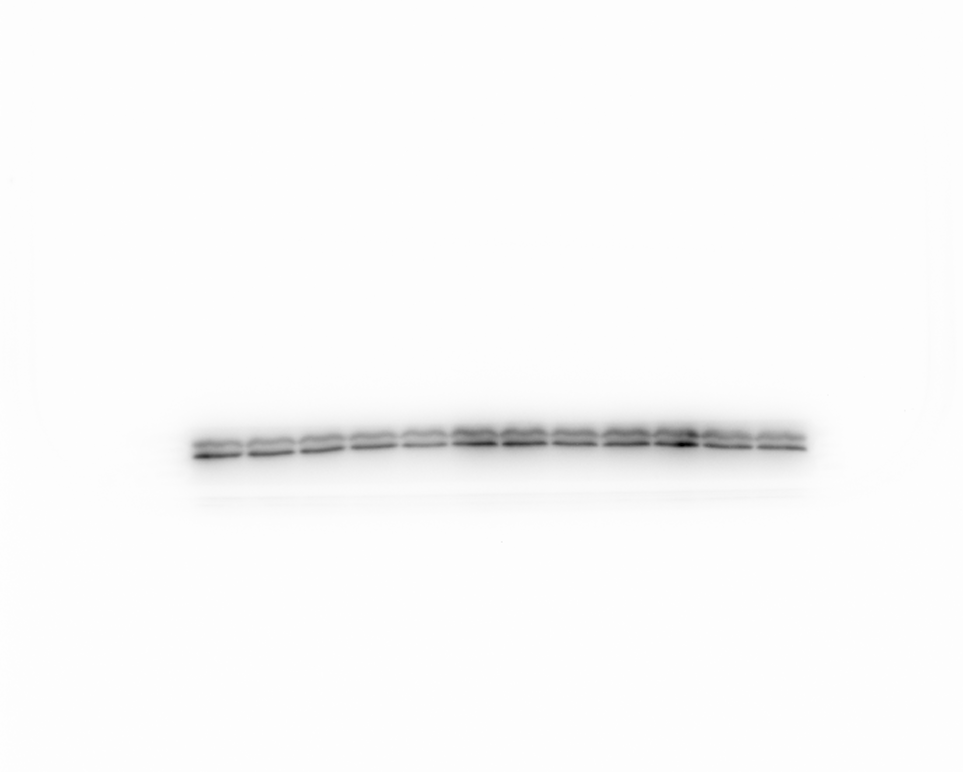

Supplement: Supplementary file 26 [file DataSheet2.ZIP › ERK1/ERK 1.tif]

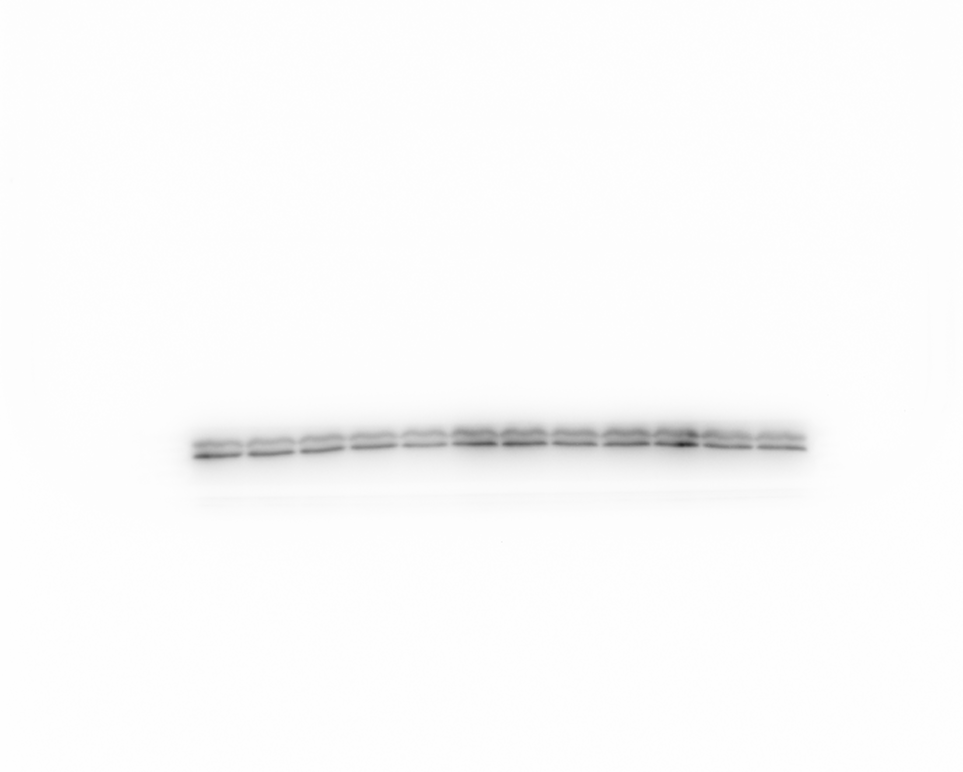

Supplement: Supplementary file 26 [file DataSheet2.ZIP › ERK1/ERK 2.tif]

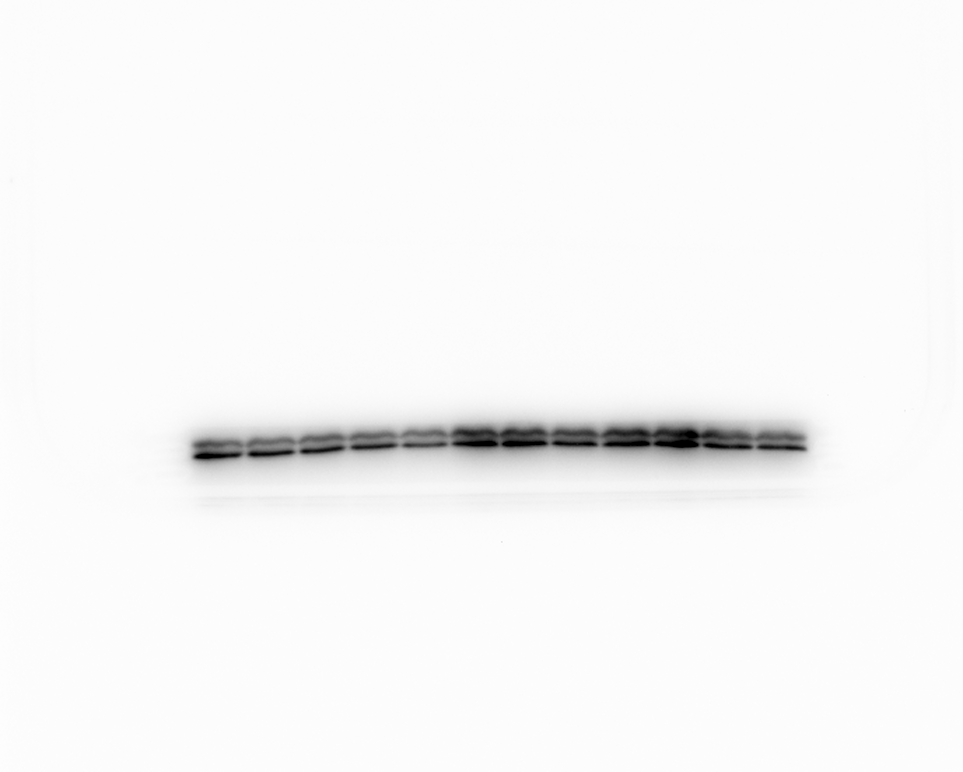

Supplement: Supplementary file 26 [file DataSheet2.ZIP › ERK1/ERK 3.tif]

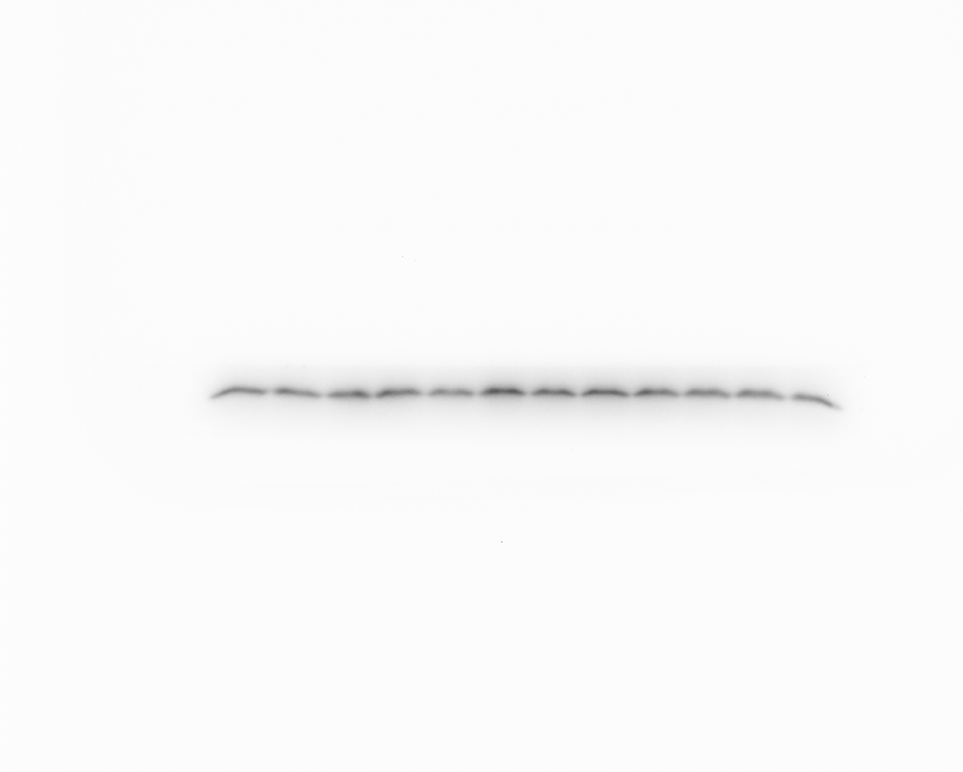

Supplement: Supplementary file 26 [file DataSheet2.ZIP › ERK1/ERK CYPB 1.tif]

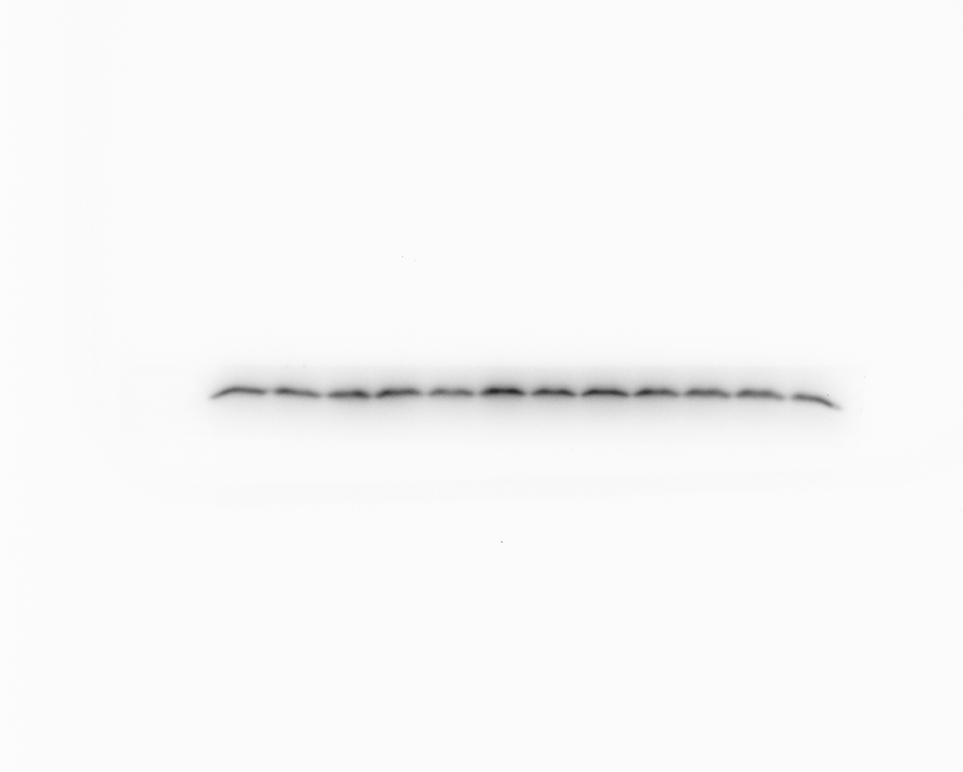

Supplement: Supplementary file 26 [file DataSheet2.ZIP › ERK1/ERK CYPB 2.tif]

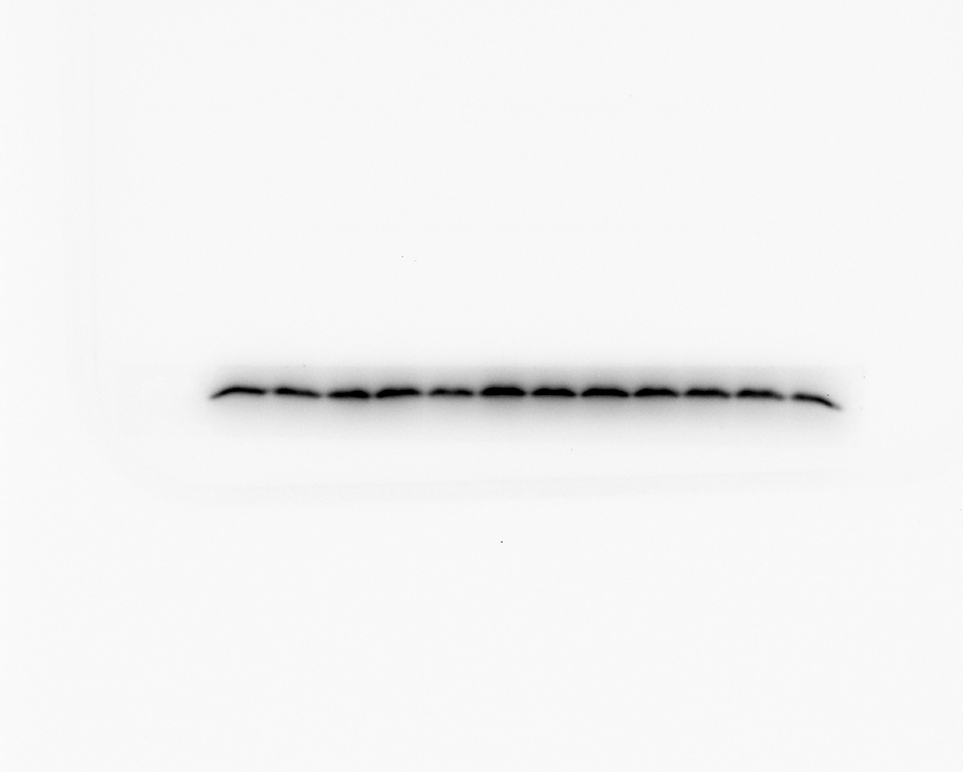

Supplement: Supplementary file 26 [file DataSheet2.ZIP › ERK1/ERK CYPB 3.tif]

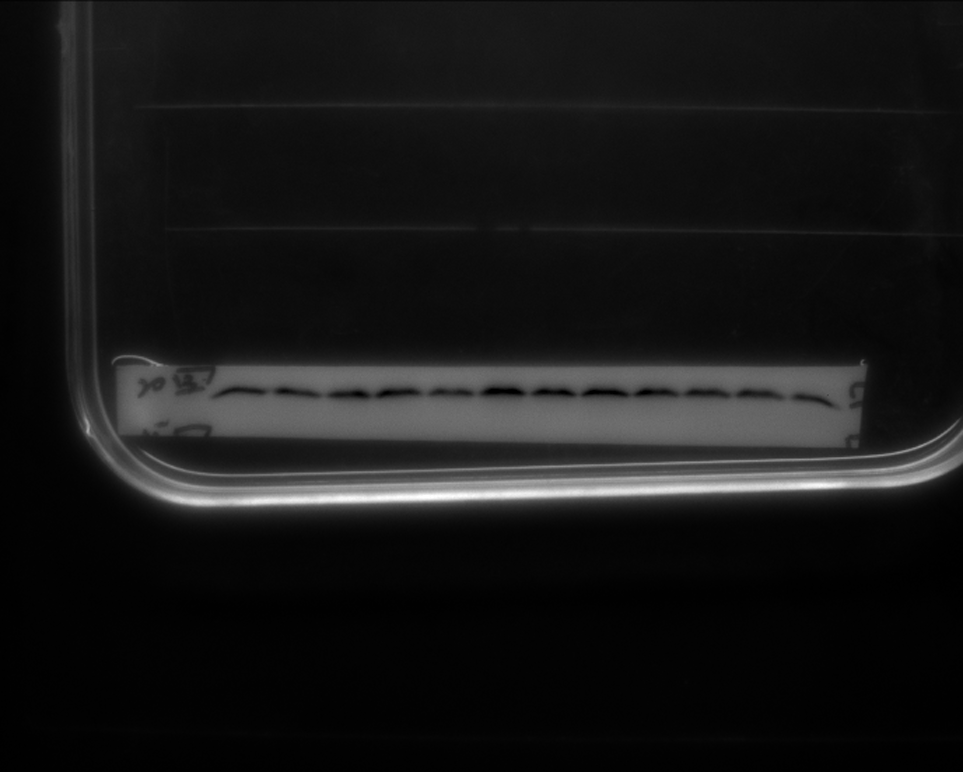

Supplement: Supplementary file 26 [file DataSheet2.ZIP › ERK1/ERK CYPB.tif]

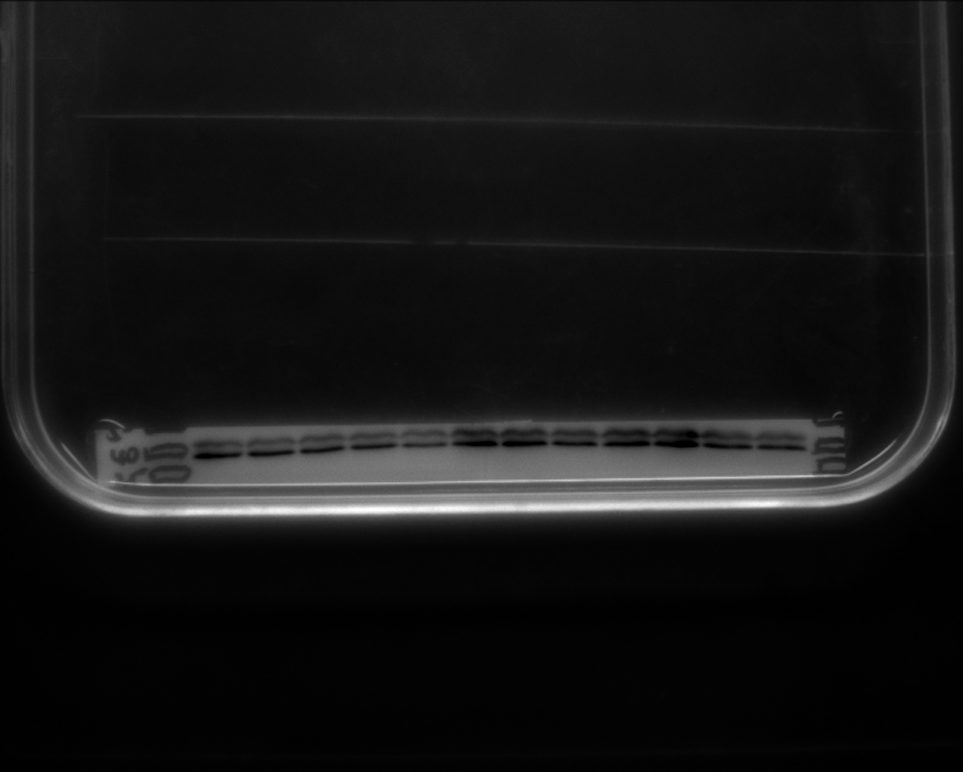

Supplement: Supplementary file 26 [file DataSheet2.ZIP › ERK1/ERK q.tif]

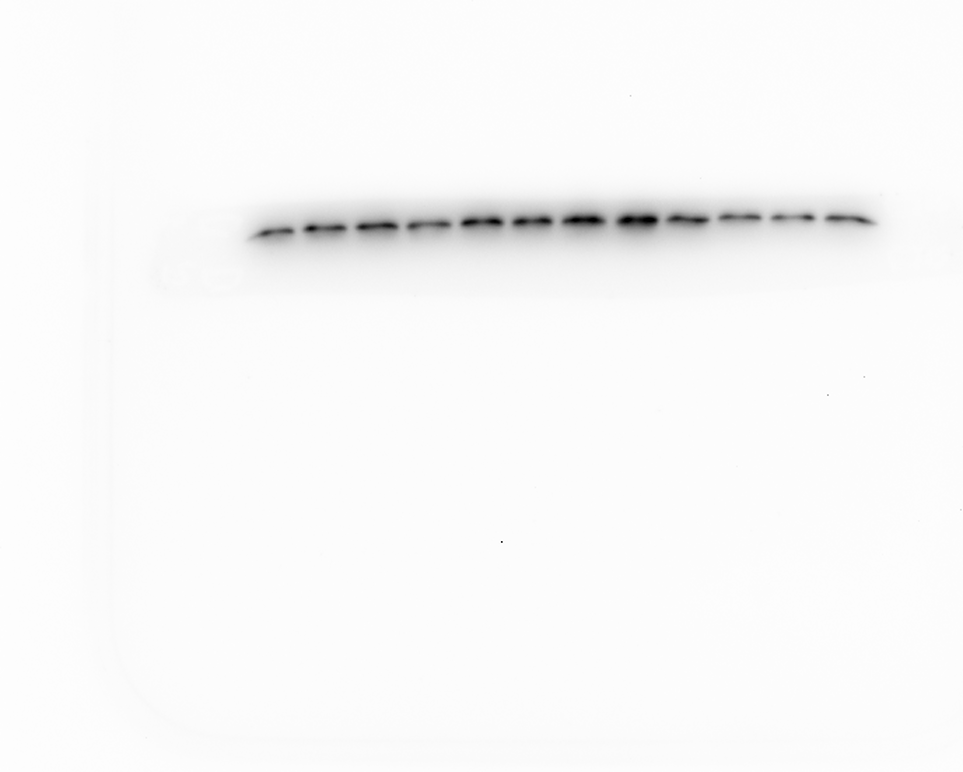

Supplement: Supplementary file 26 [file DataSheet2.ZIP › ERK1/p-ERK CYPB 1.tif]

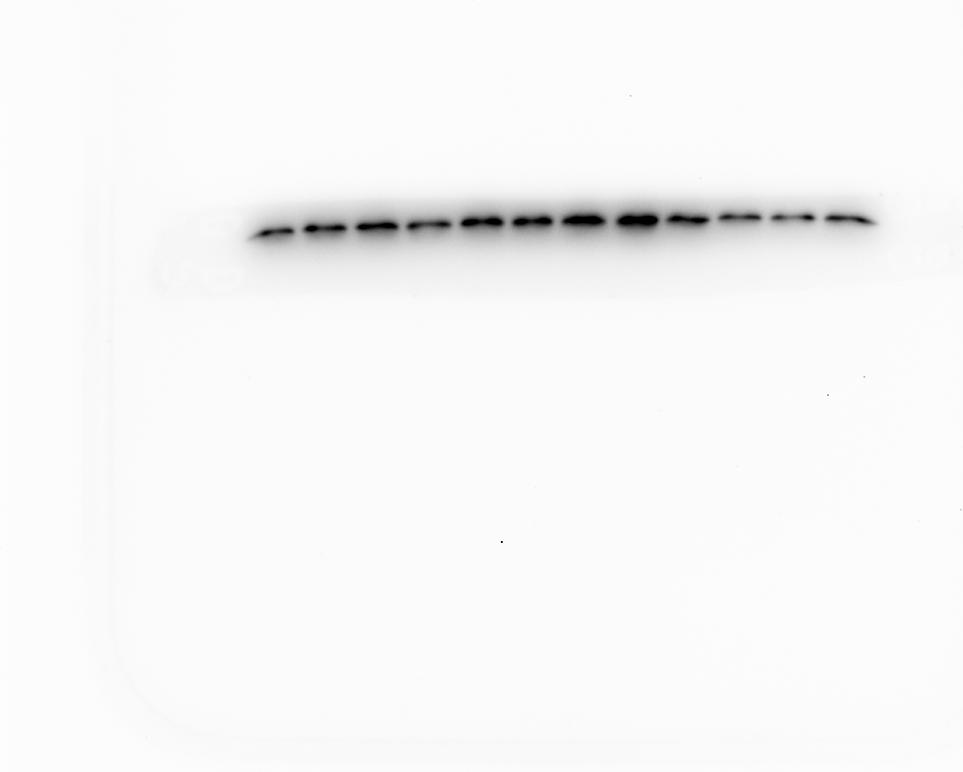

Supplement: Supplementary file 26 [file DataSheet2.ZIP › ERK1/p-ERK CYPB 2.tif]

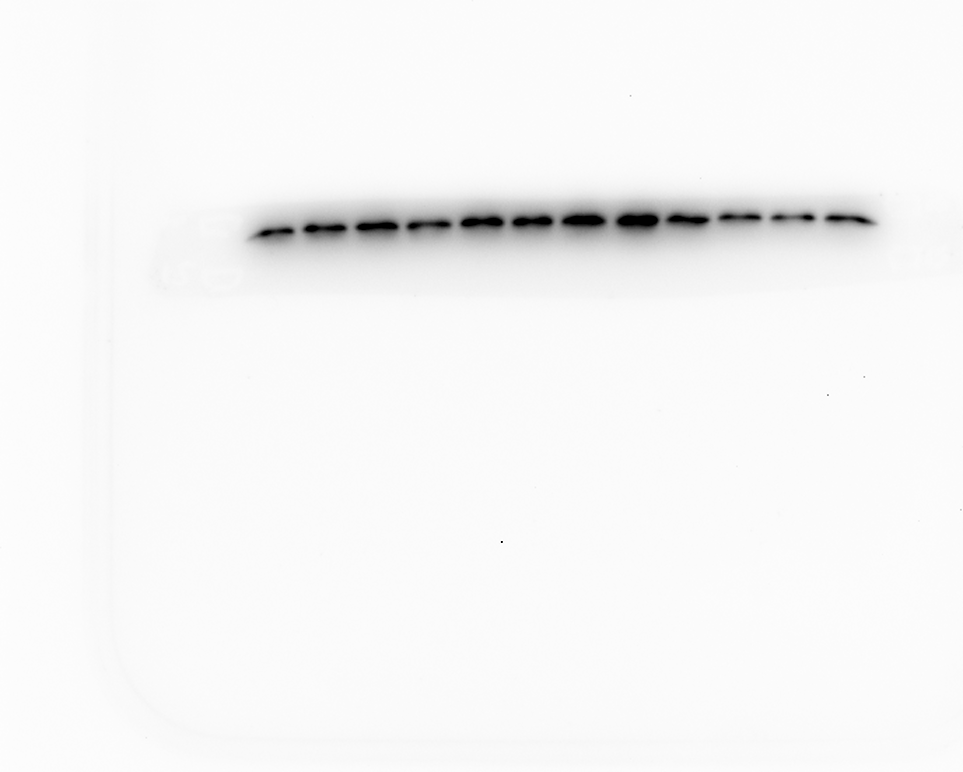

Supplement: Supplementary file 26 [file DataSheet2.ZIP › ERK1/p-ERK CYPB 3.tif]

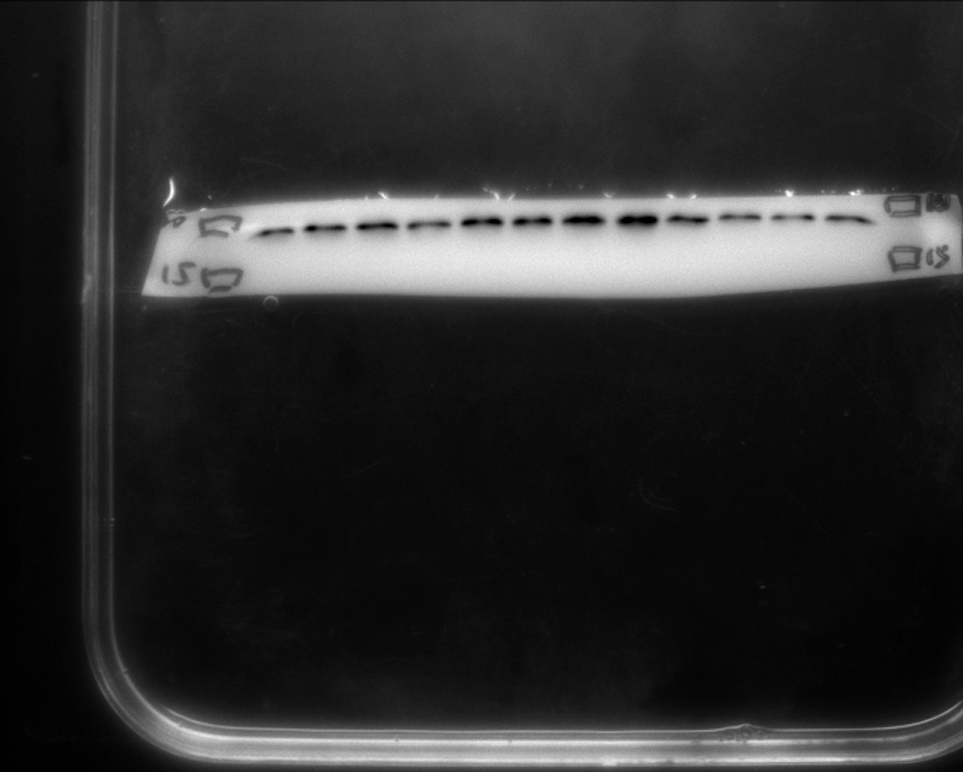

Supplement: Supplementary file 26 [file DataSheet2.ZIP › ERK1/p-ERK CYPB q.tif]

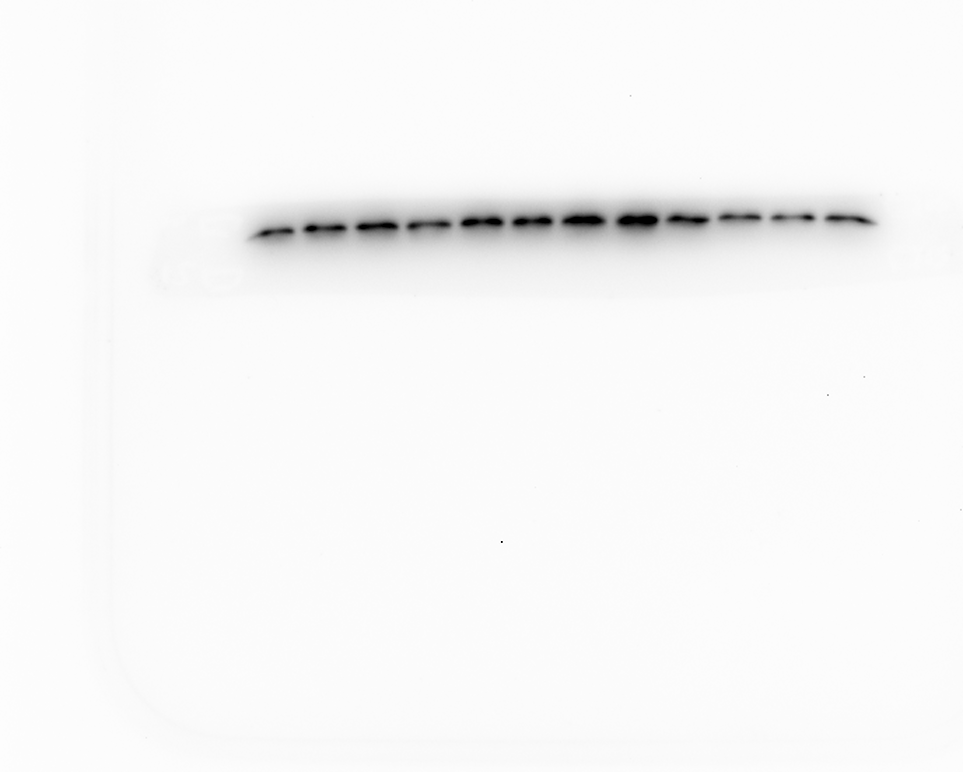

Supplement: Supplementary file 26 [file DataSheet2.ZIP › ERK1/p-ERK CYPB.tif]

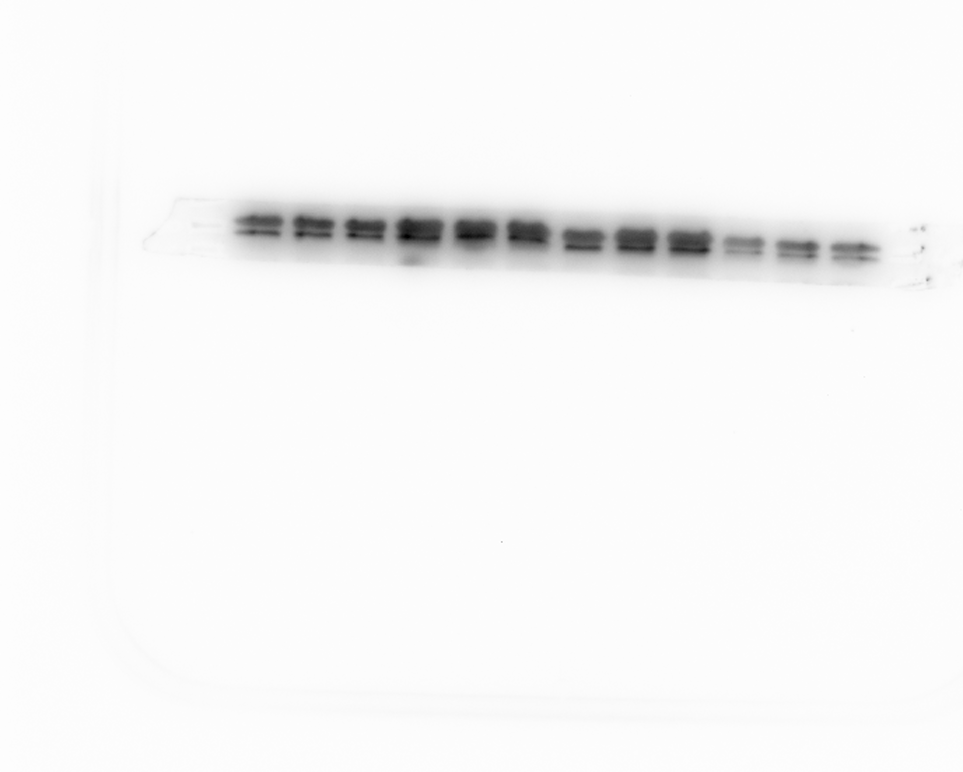

Supplement: Supplementary file 26 [file DataSheet2.ZIP › ERK1/p-ERK1.tif]

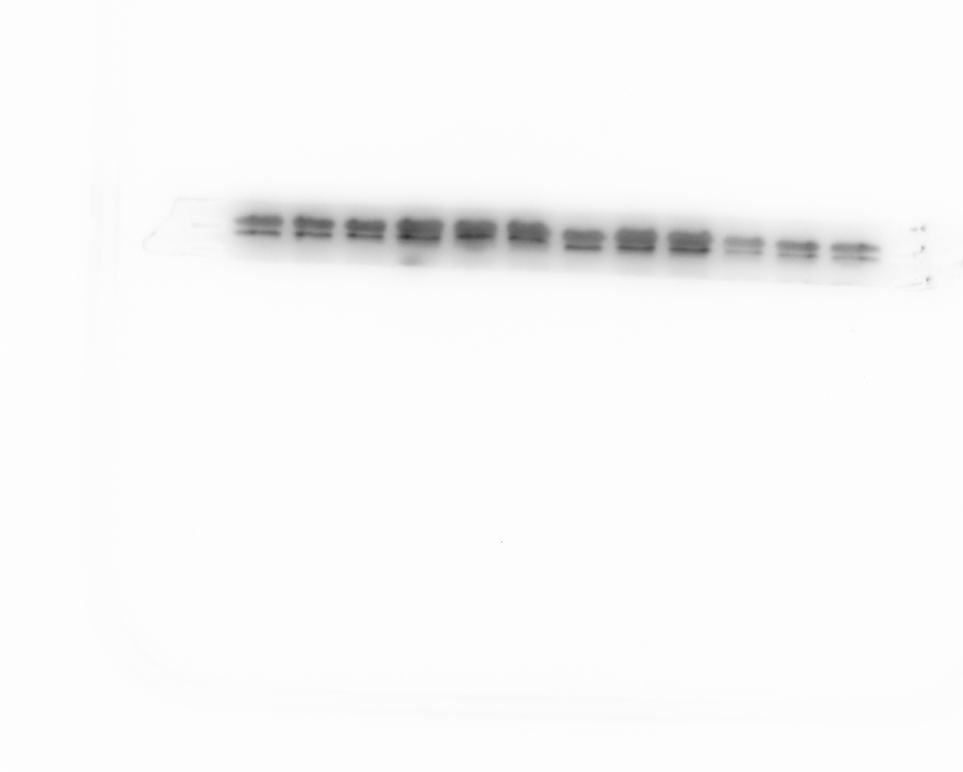

Supplement: Supplementary file 26 [file DataSheet2.ZIP › ERK1/p-ERK6.tif]

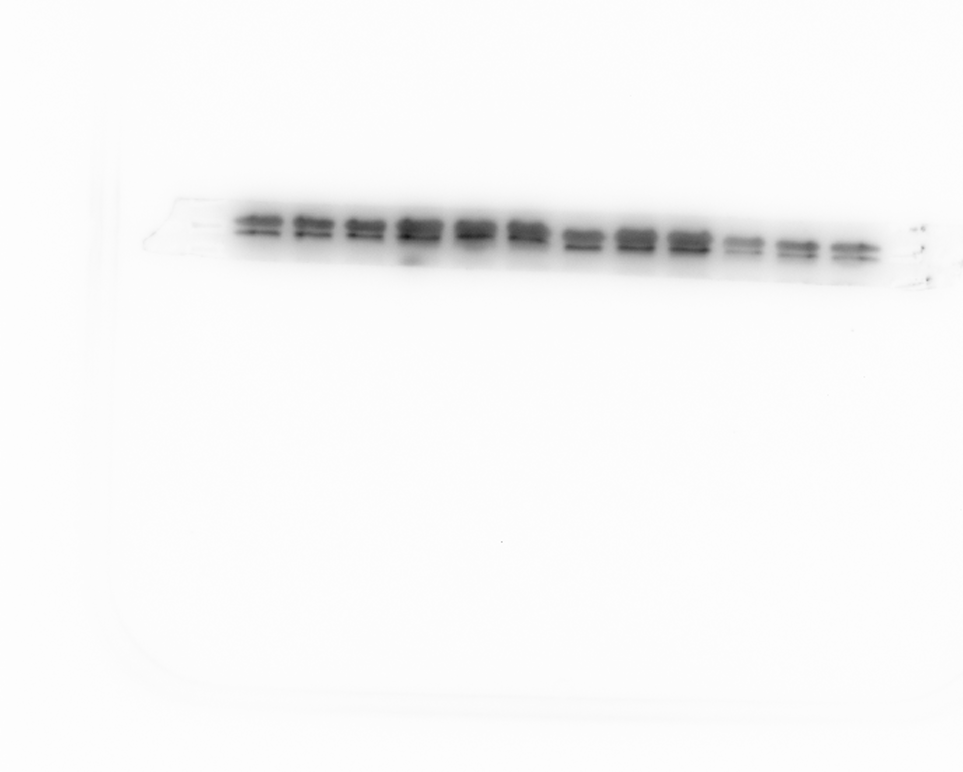

Supplement: Supplementary file 26 [file DataSheet2.ZIP › ERK1/p-ERK7.tif]

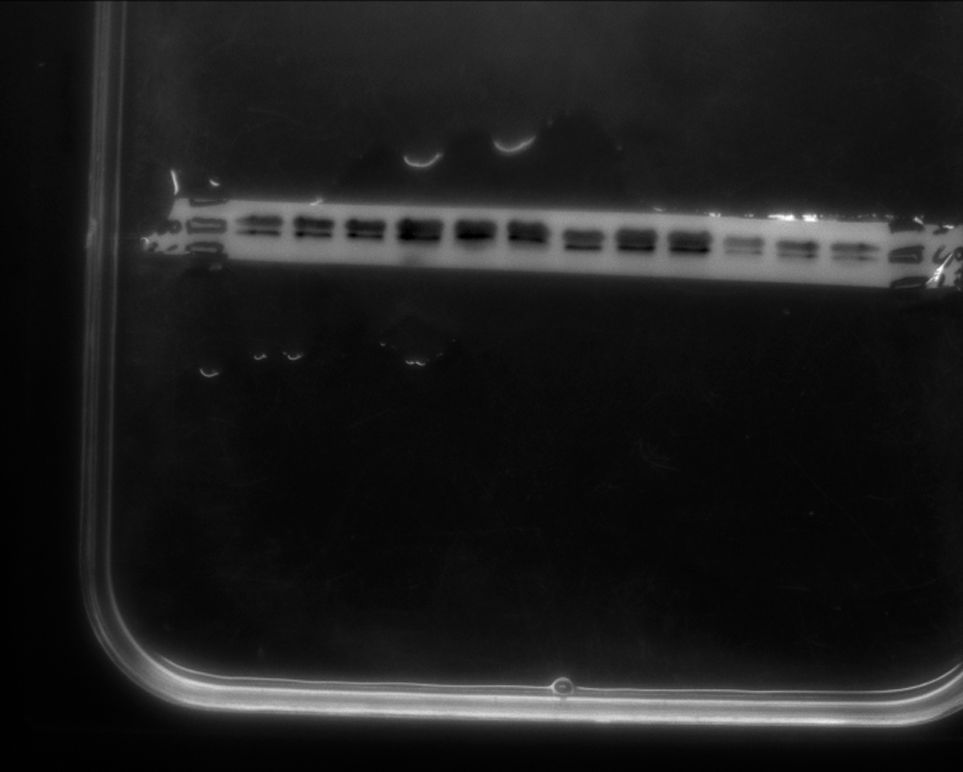

Supplement: Supplementary file 26 [file DataSheet2.ZIP › ERK1/p-ERKq.tif]

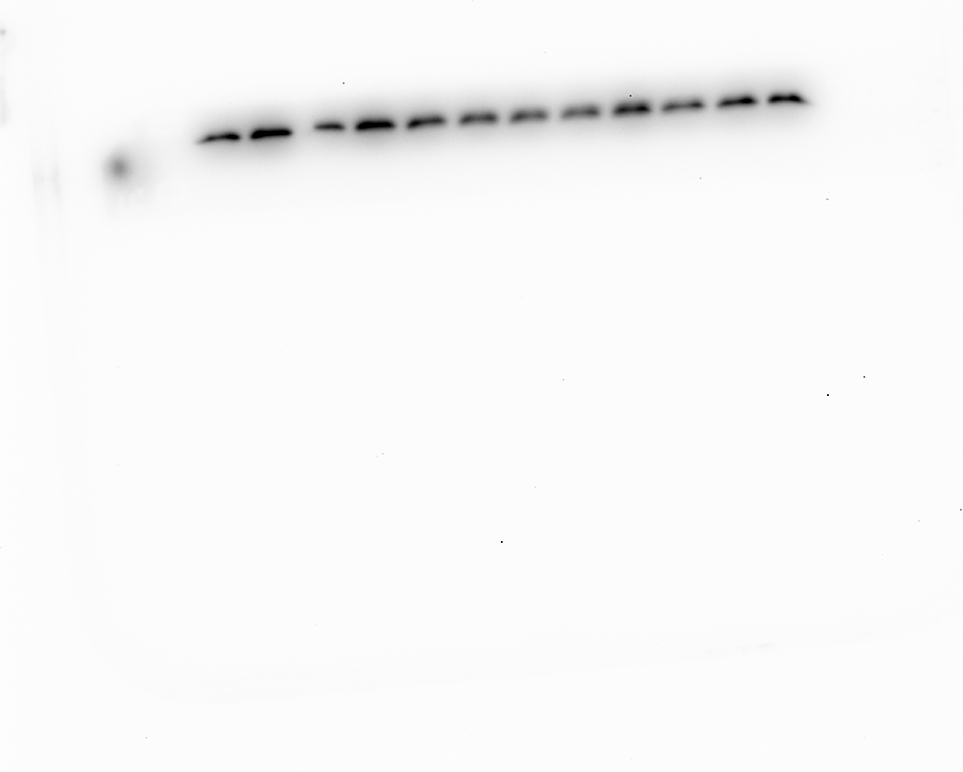

Supplement: Supplementary file 26 [file DataSheet2.ZIP › ERK2/ERK CYPB1.tif]

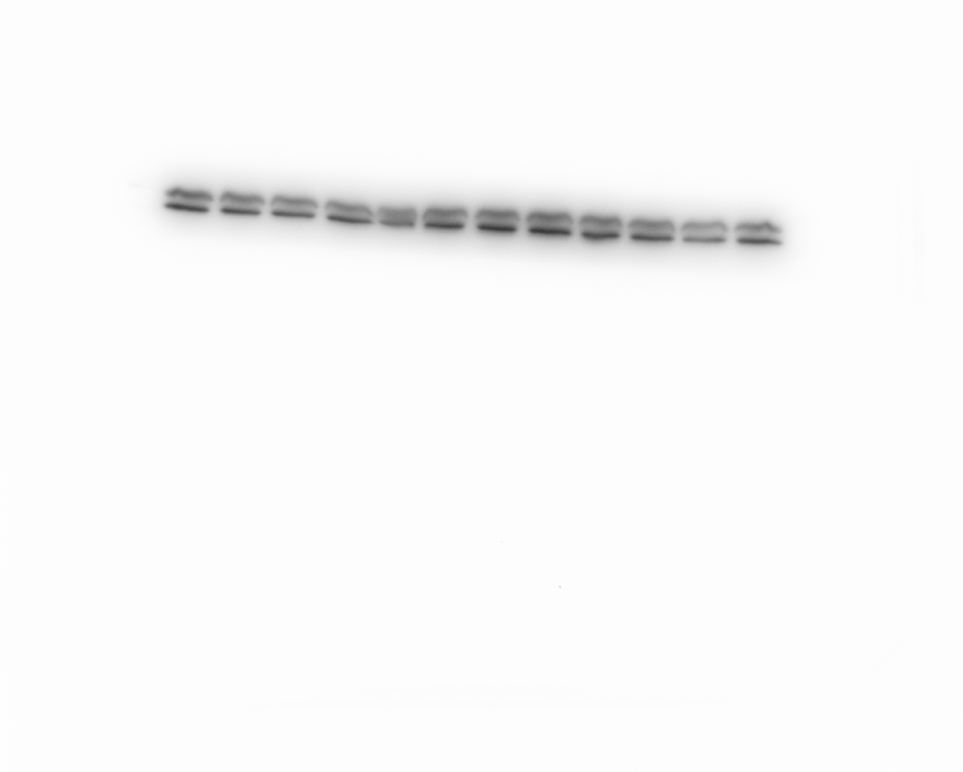

Supplement: Supplementary file 26 [file DataSheet2.ZIP › ERK2/ERKq 1.tif]

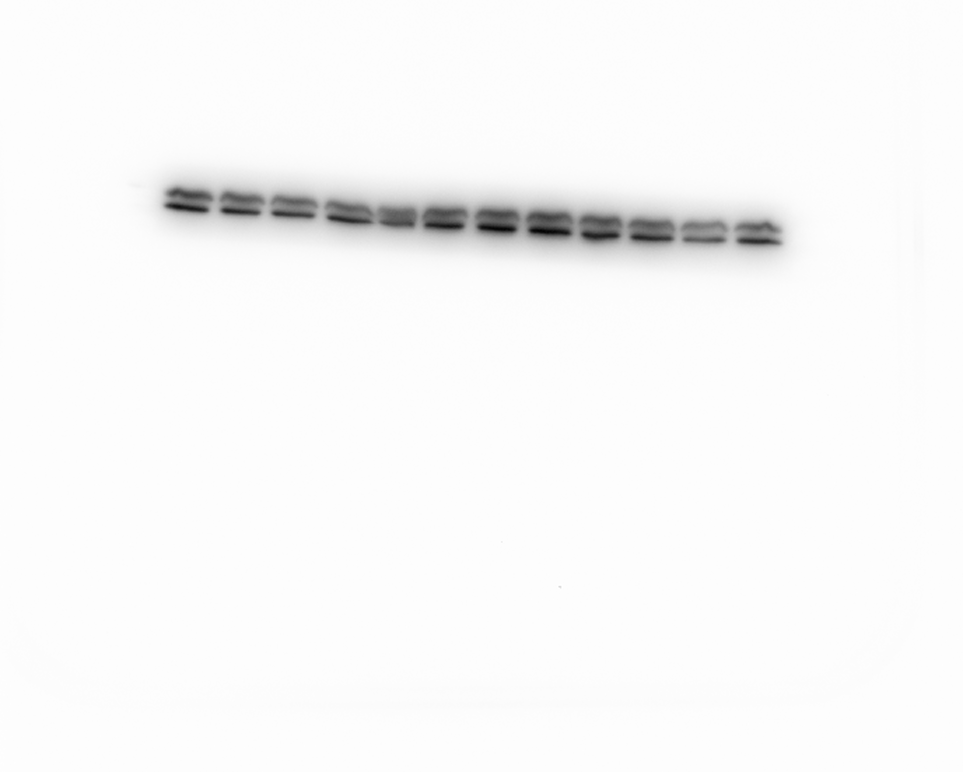

Supplement: Supplementary file 26 [file DataSheet2.ZIP › ERK2/ERKq 2.tif]

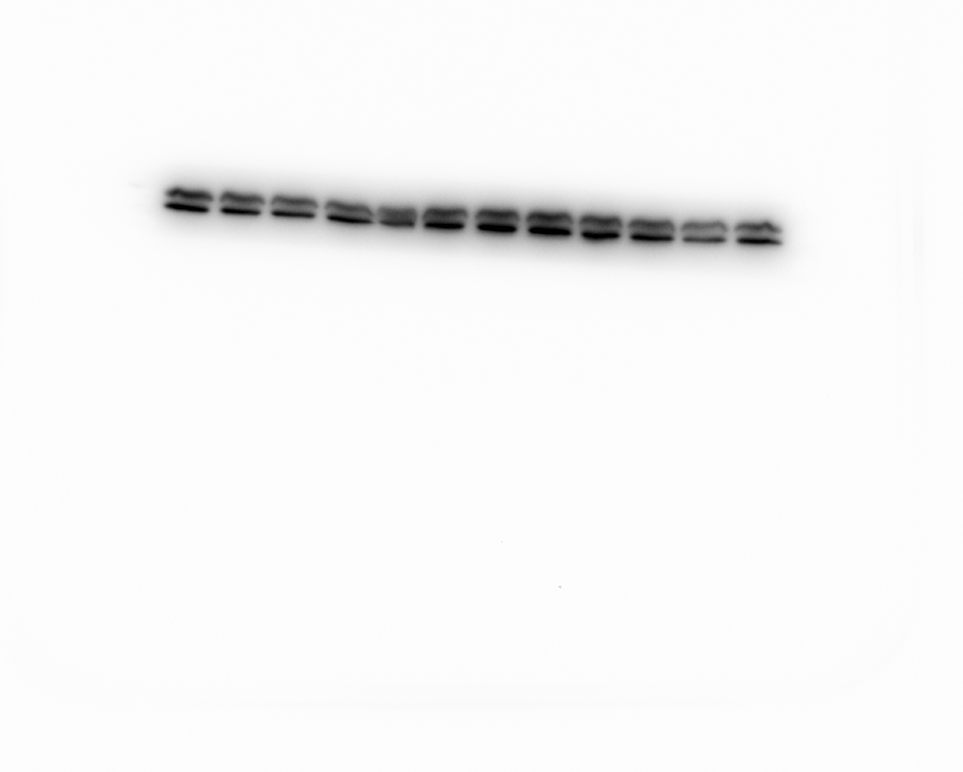

Supplement: Supplementary file 26 [file DataSheet2.ZIP › ERK2/ERKq 3.tif]

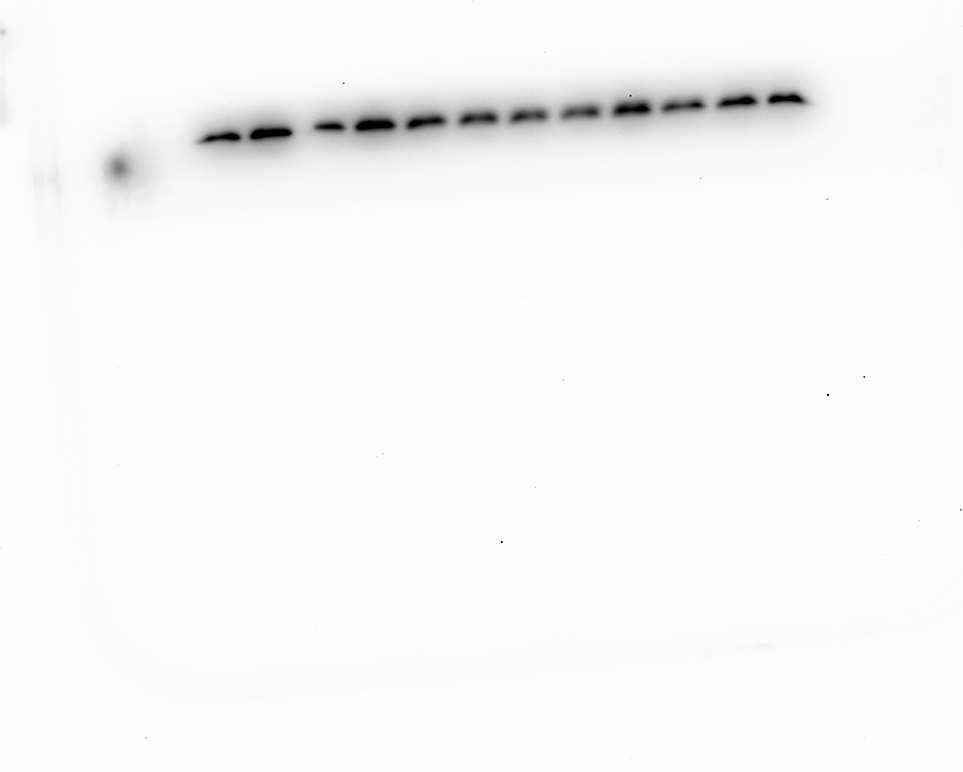

Supplement: Supplementary file 26 [file DataSheet2.ZIP › ERK2/ERKq CYPB 2.tif]

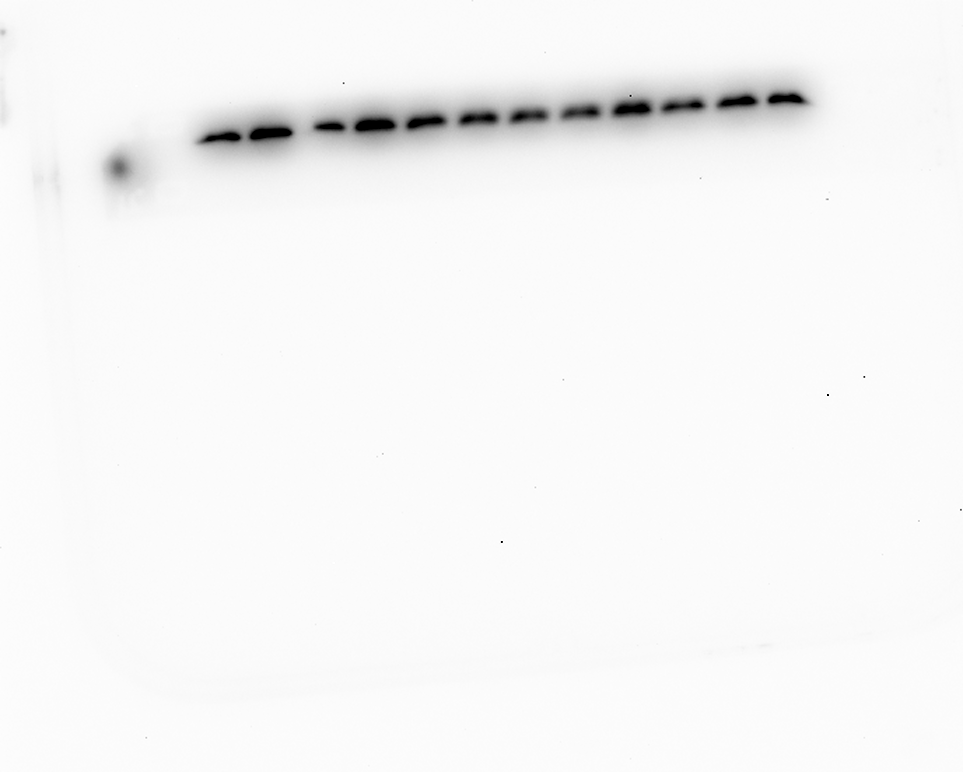

Supplement: Supplementary file 26 [file DataSheet2.ZIP › ERK2/ERKq CYPB 3.tif]

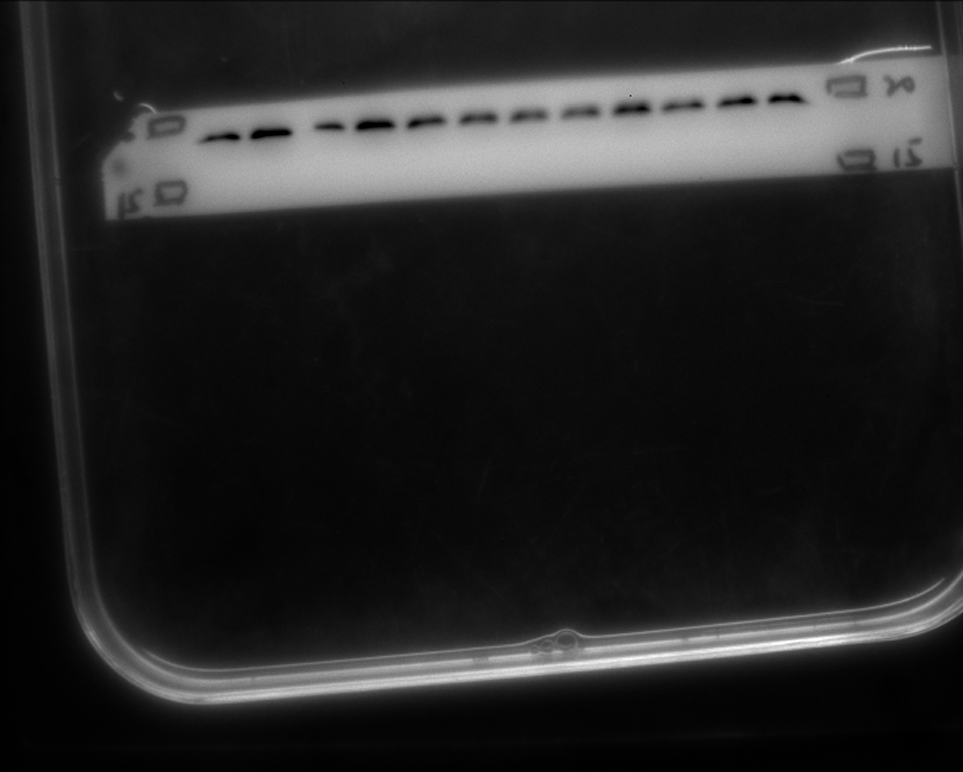

Supplement: Supplementary file 26 [file DataSheet2.ZIP › ERK2/ERKq CYPB.tif]

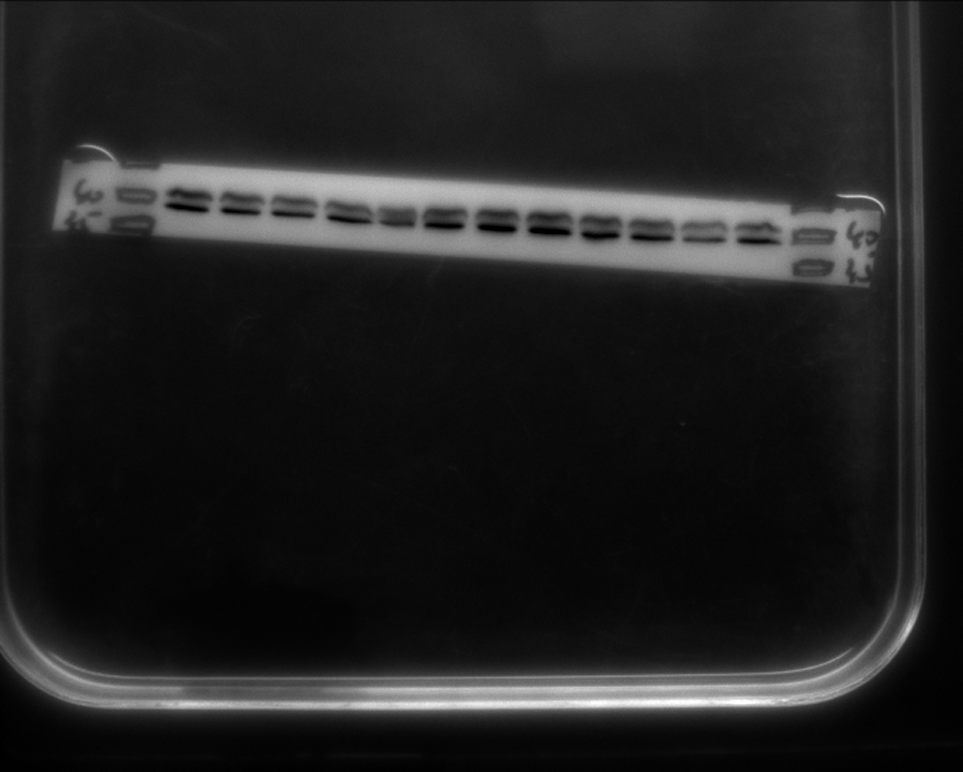

Supplement: Supplementary file 26 [file DataSheet2.ZIP › ERK2/ERKq.tif]

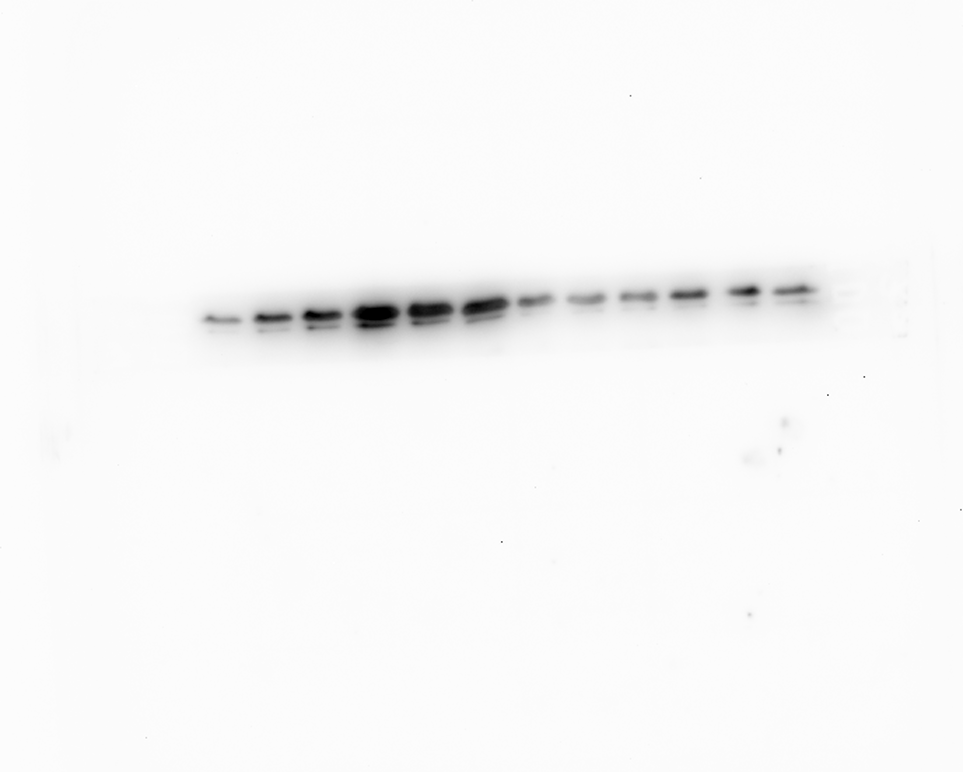

Supplement: Supplementary file 26 [file DataSheet2.ZIP › ERK2/p-ERK 1.tif]

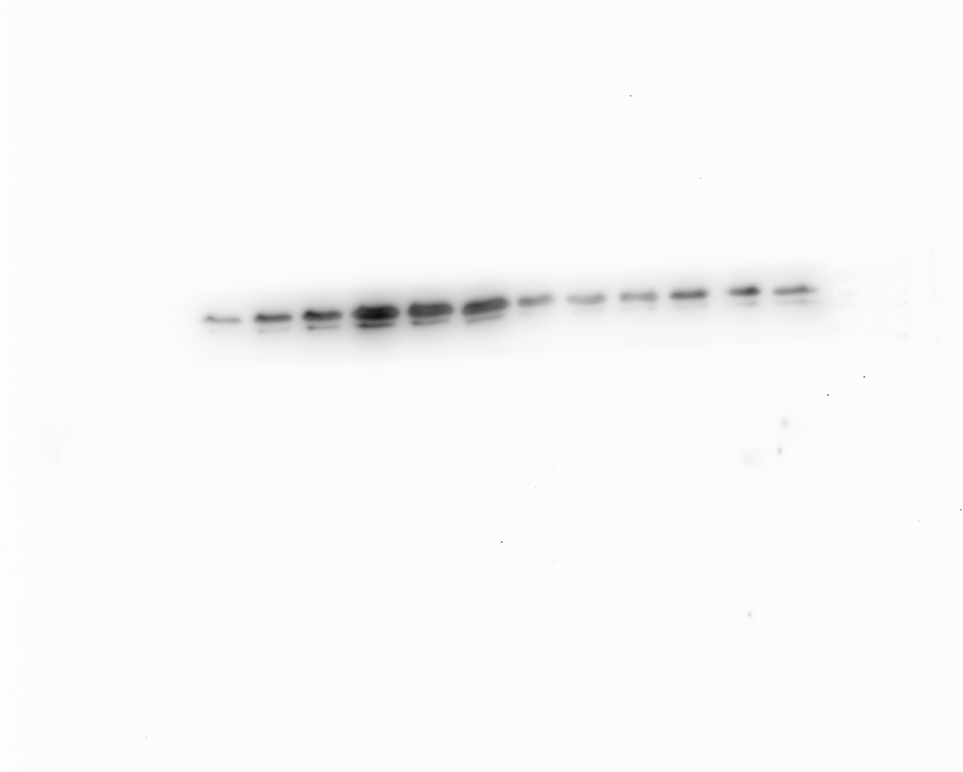

Supplement: Supplementary file 26 [file DataSheet2.ZIP › ERK2/p-ERK 2.tif]

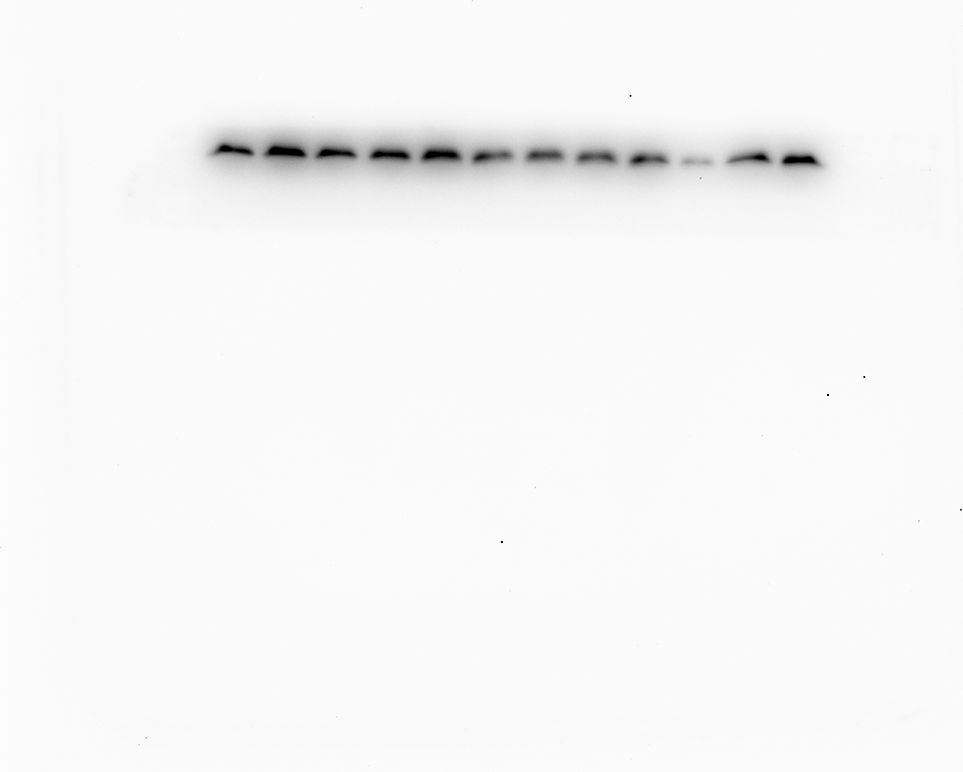

Supplement: Supplementary file 26 [file DataSheet2.ZIP › ERK2/p-ERK CYPB 1.tif]

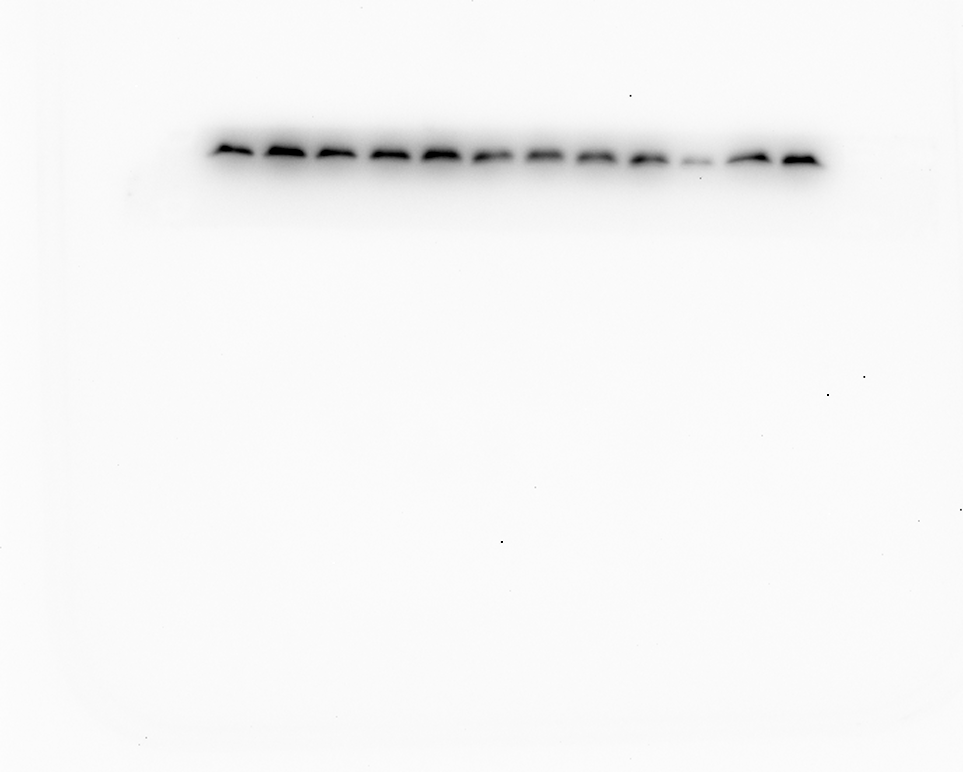

Supplement: Supplementary file 26 [file DataSheet2.ZIP › ERK2/p-ERK CYPB 4.tif]

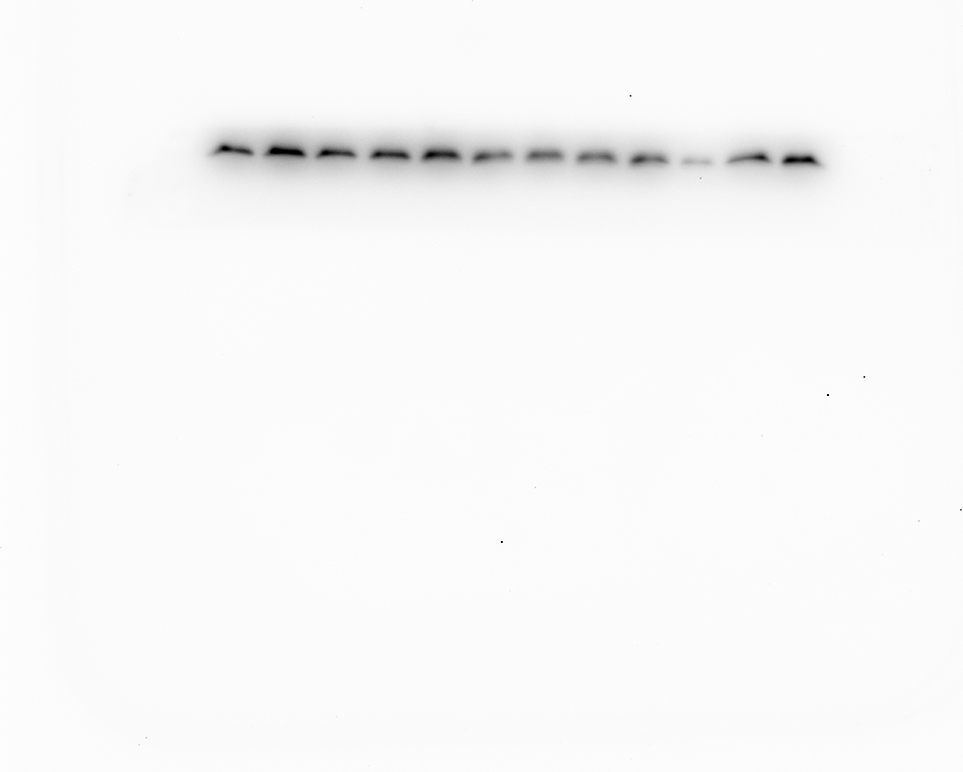

Supplement: Supplementary file 26 [file DataSheet2.ZIP › ERK2/p-ERK CYPB 6.tif]

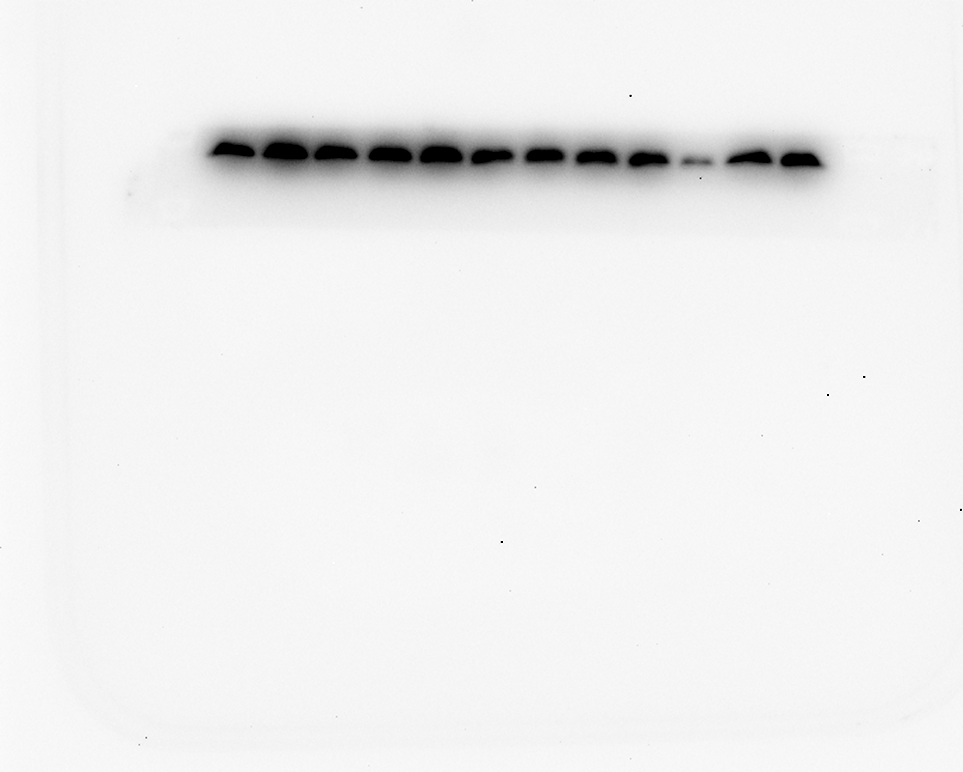

Supplement: Supplementary file 26 [file DataSheet2.ZIP › ERK2/p-ERK CYPB gb.tif]

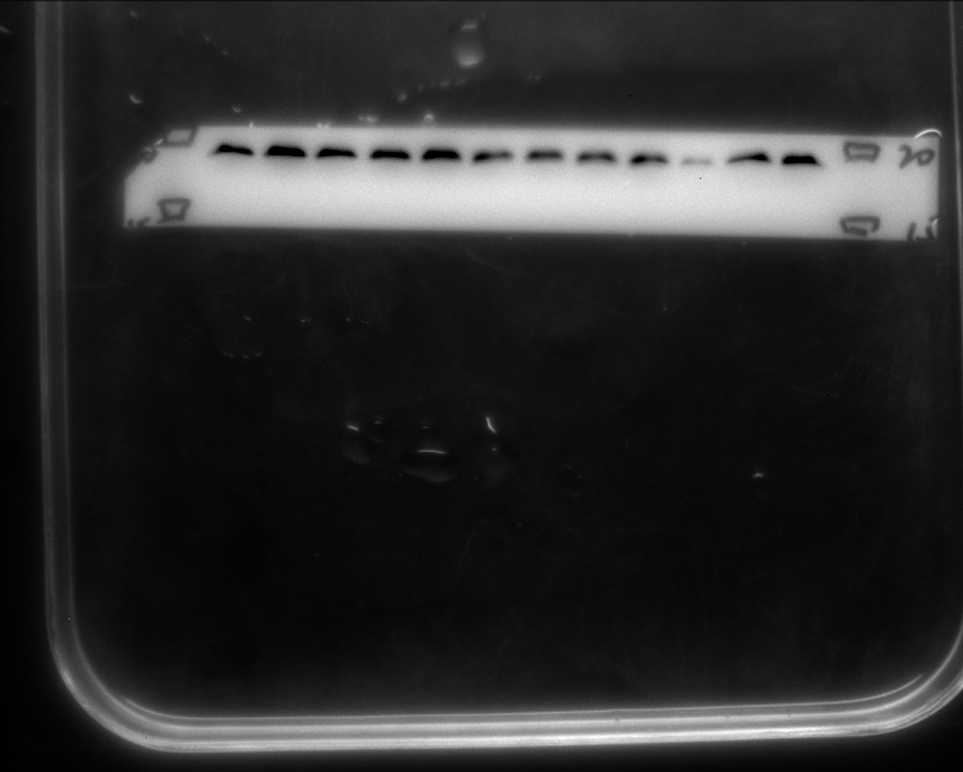

Supplement: Supplementary file 26 [file DataSheet2.ZIP › ERK2/p-ERK CYPB q.tif]

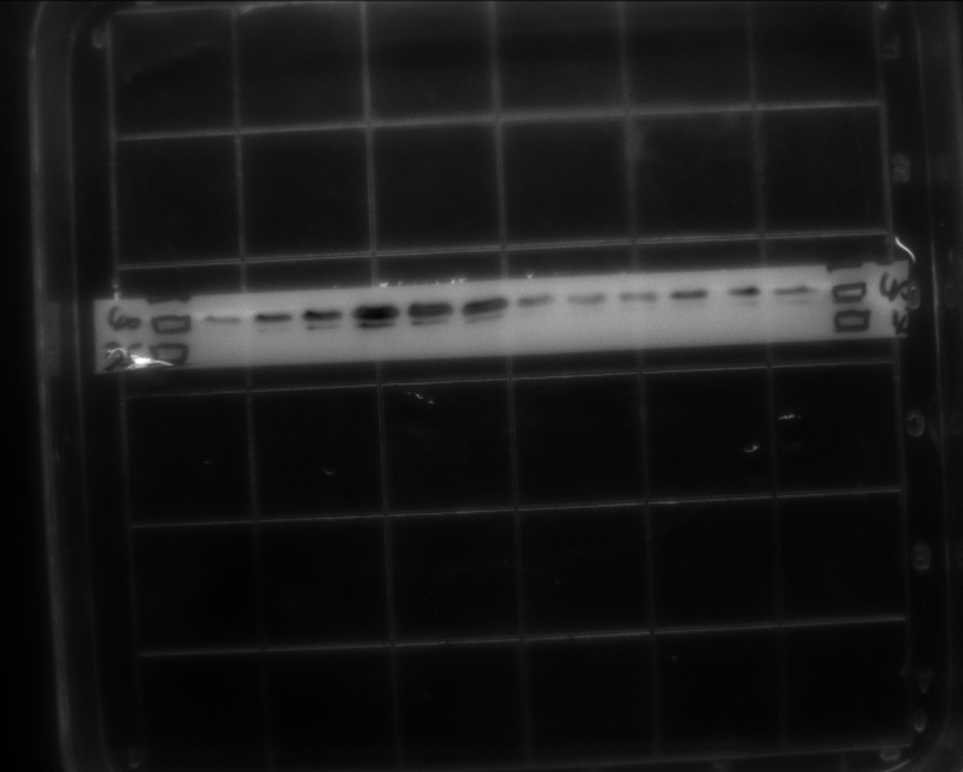

Supplement: Supplementary file 26 [file DataSheet2.ZIP › ERK2/p-ERK q.tif]

## Slide 1
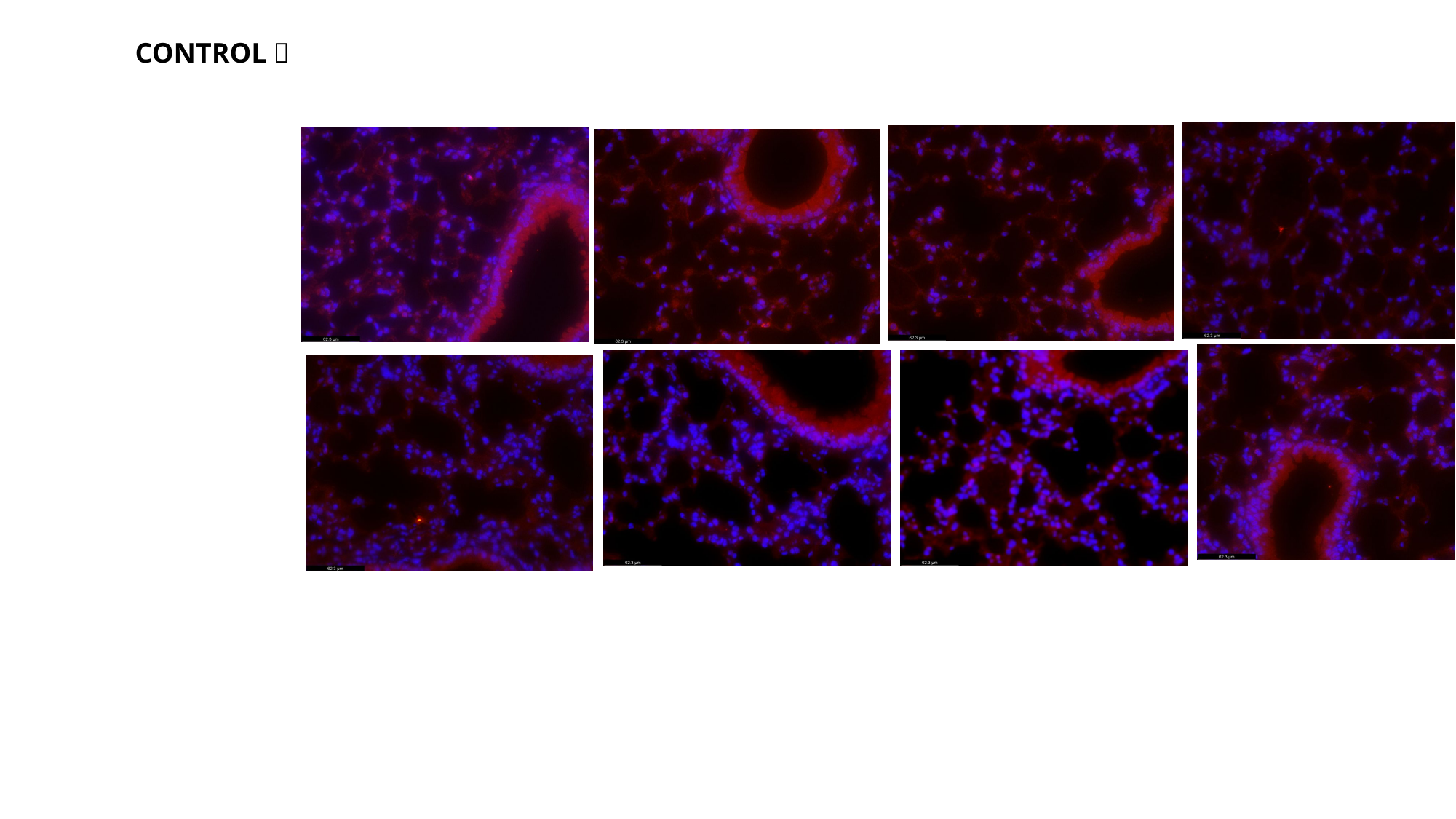

CONTROL：

Supplement: Supplementary file 28 [file Presentation3.PPTX]

## Slide 1
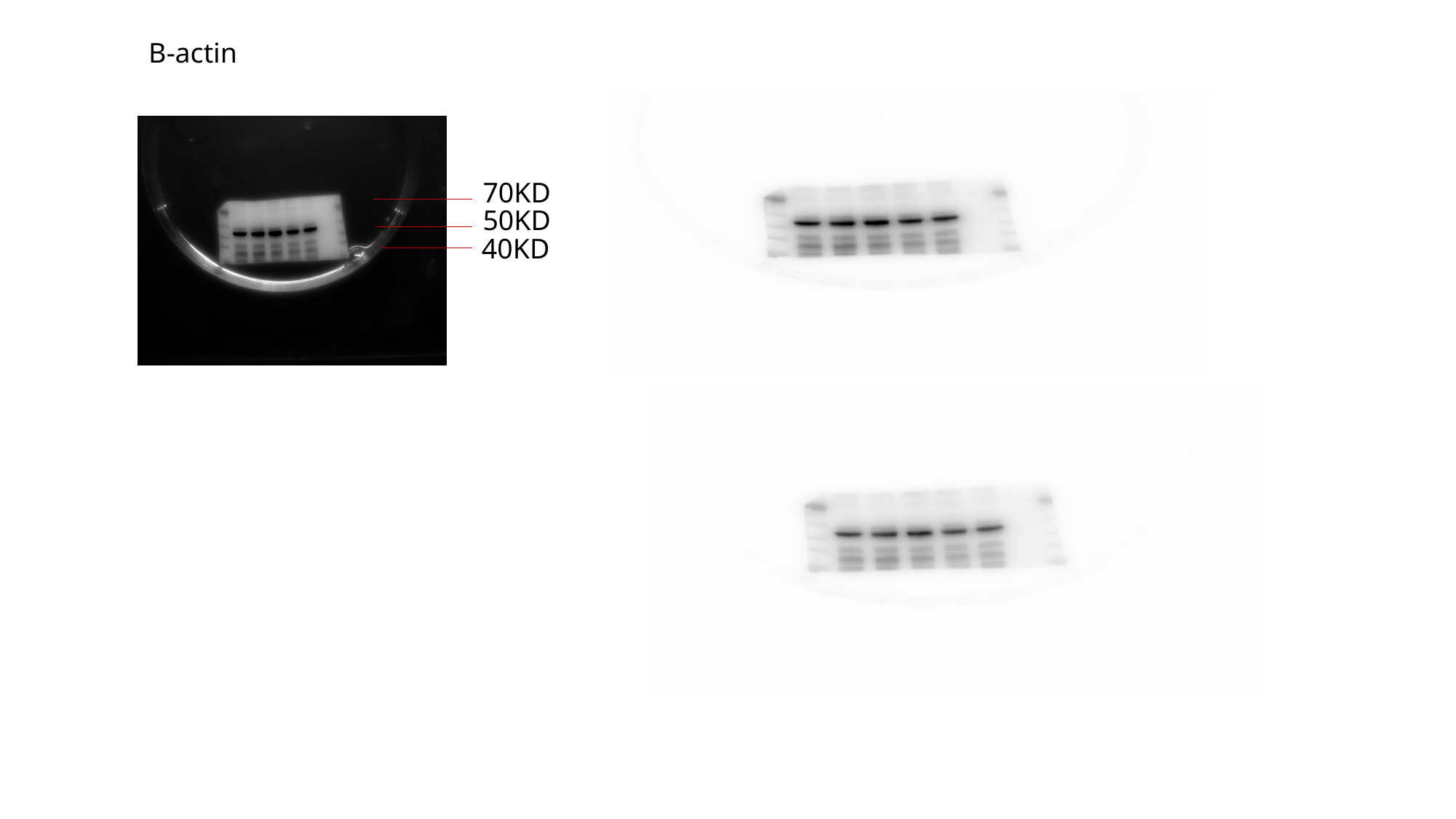

Β-actin
70KD
50KD
40KD

Supplement: Supplementary file 29 [file Presentation2.PPTX]

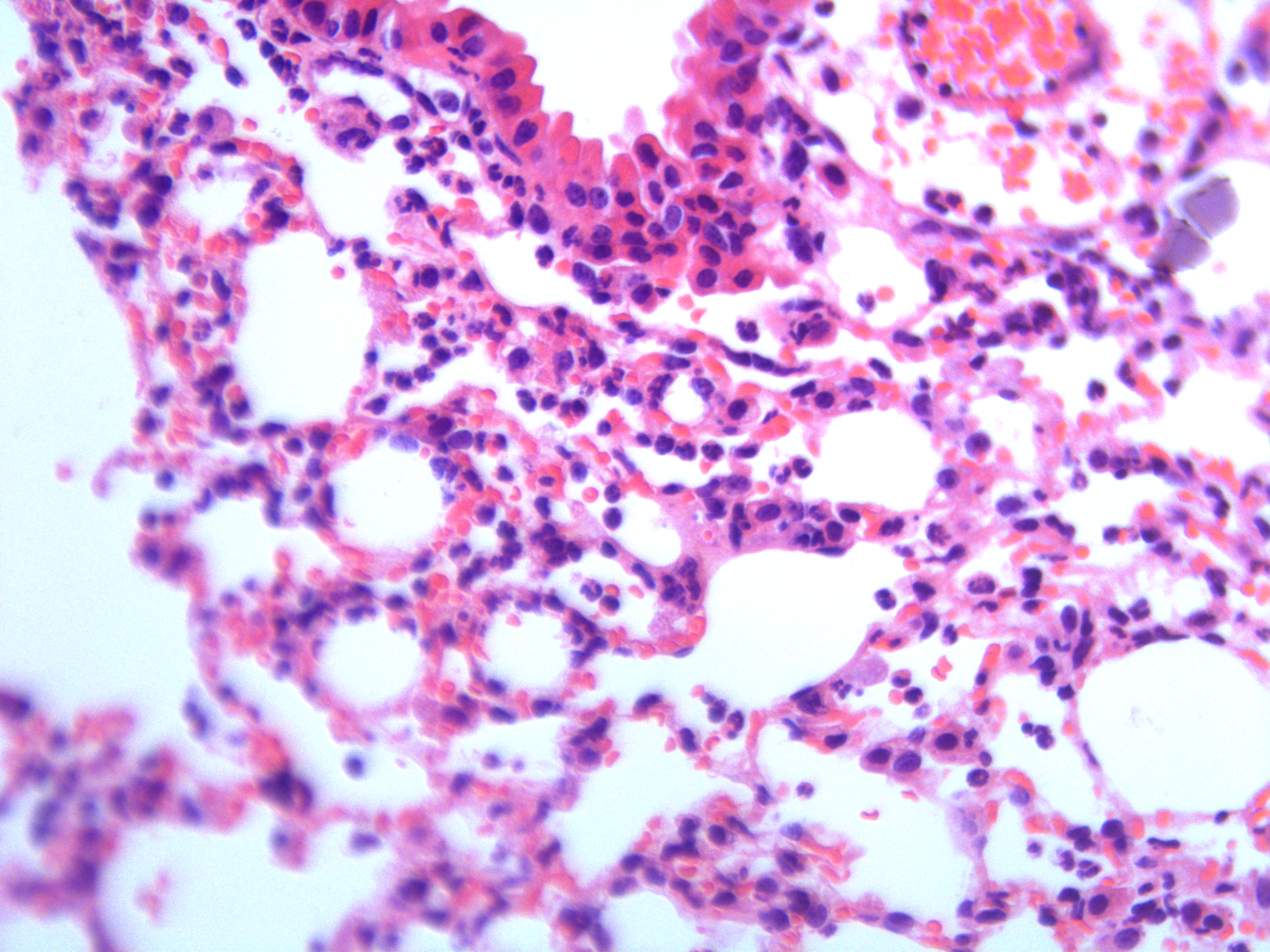

Supplement: Supplementary file 30 [file Image8.TIF]

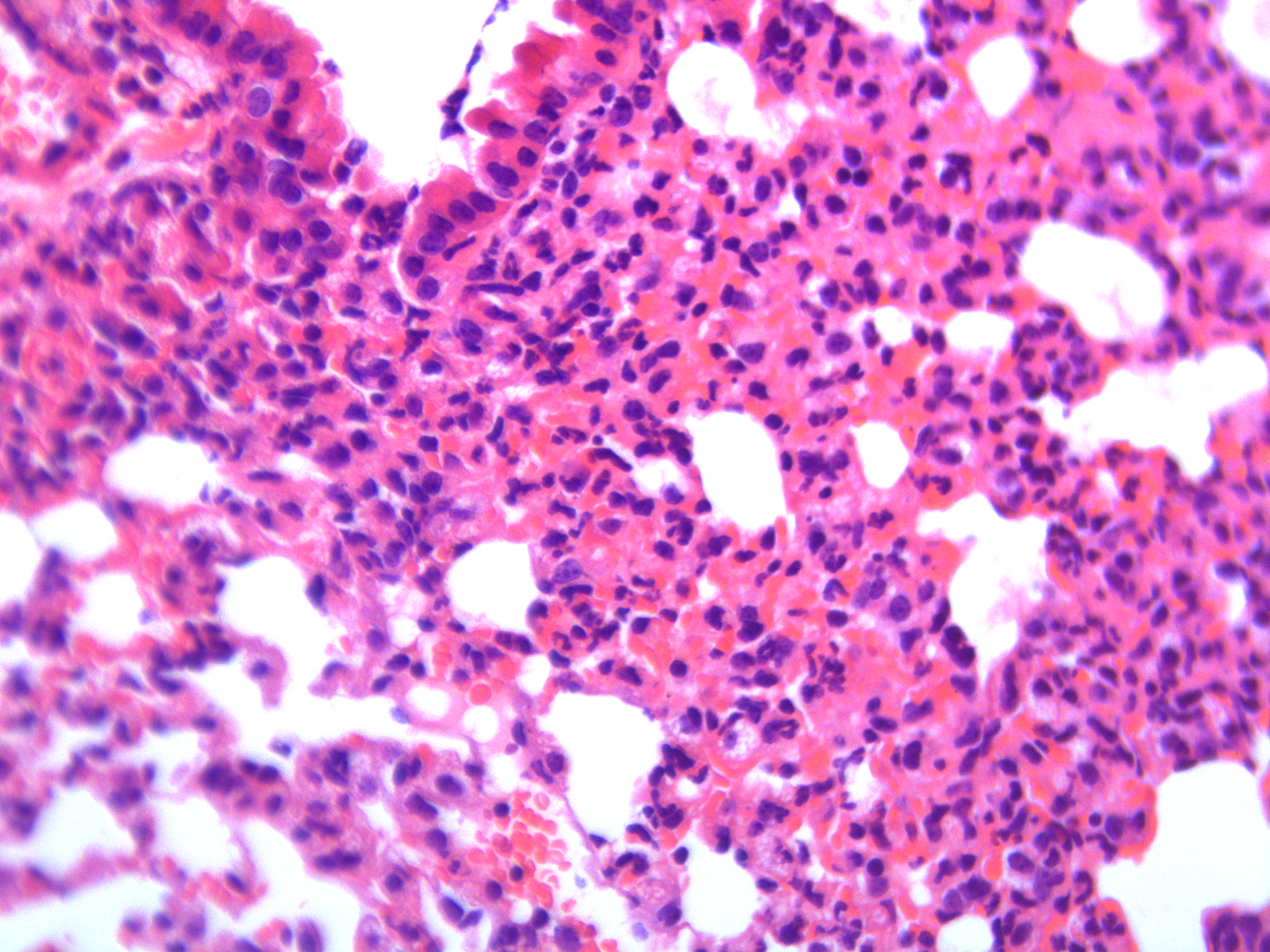

Supplement: Supplementary file 31 [file Image5.TIF]

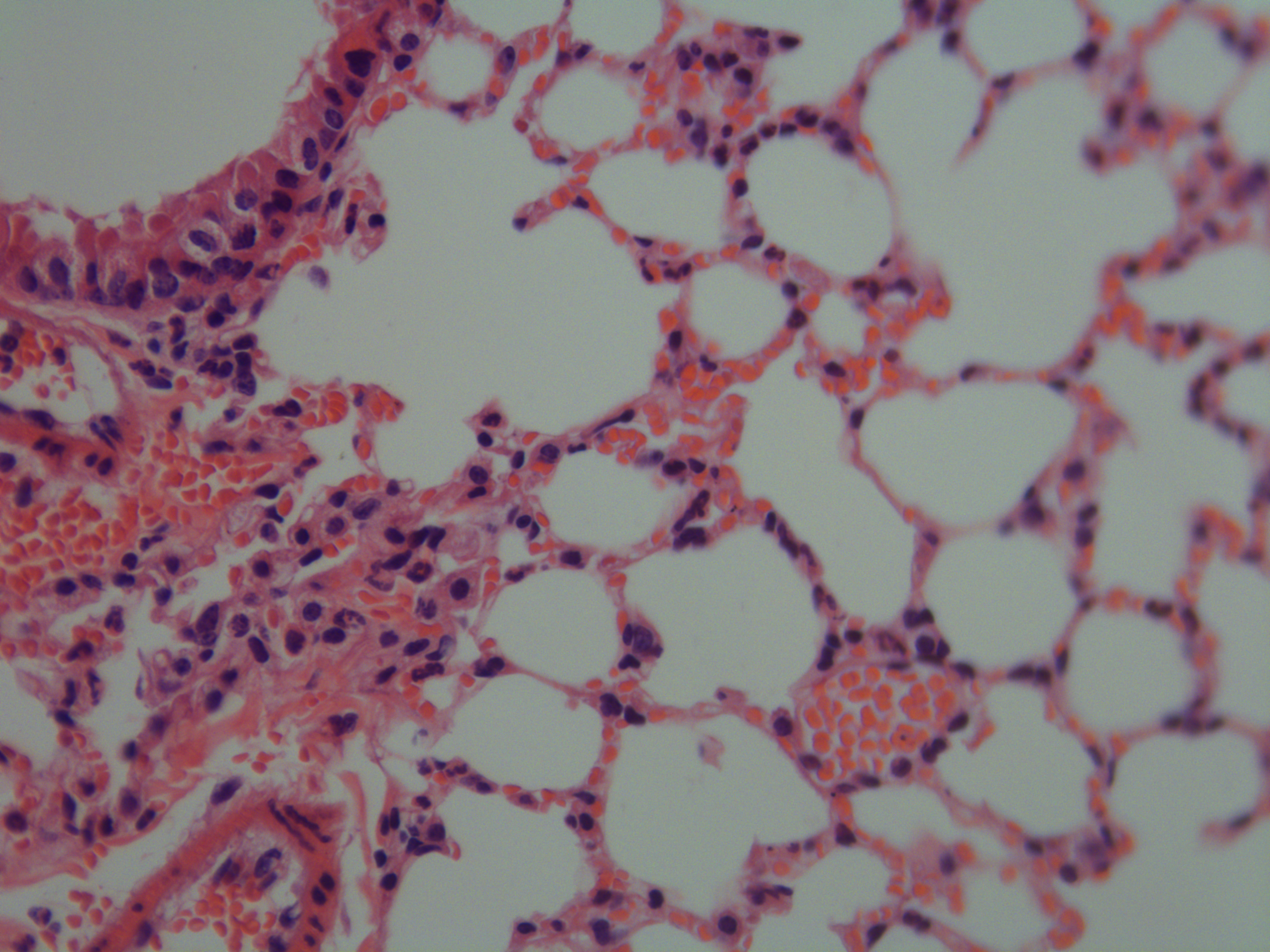

Supplement: Supplementary file 32 [file Image15.TIF]

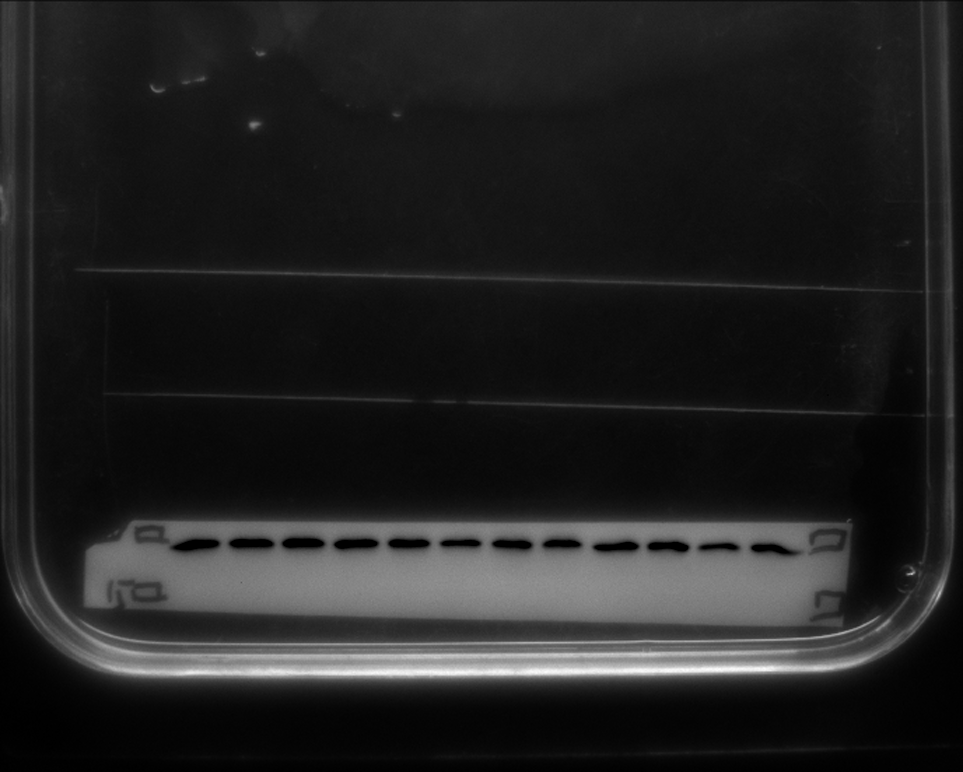

Supplement: Supplementary file 33 [file DataSheet5.ZIP › P38-1/p-p38 CYPB q 2.tif]

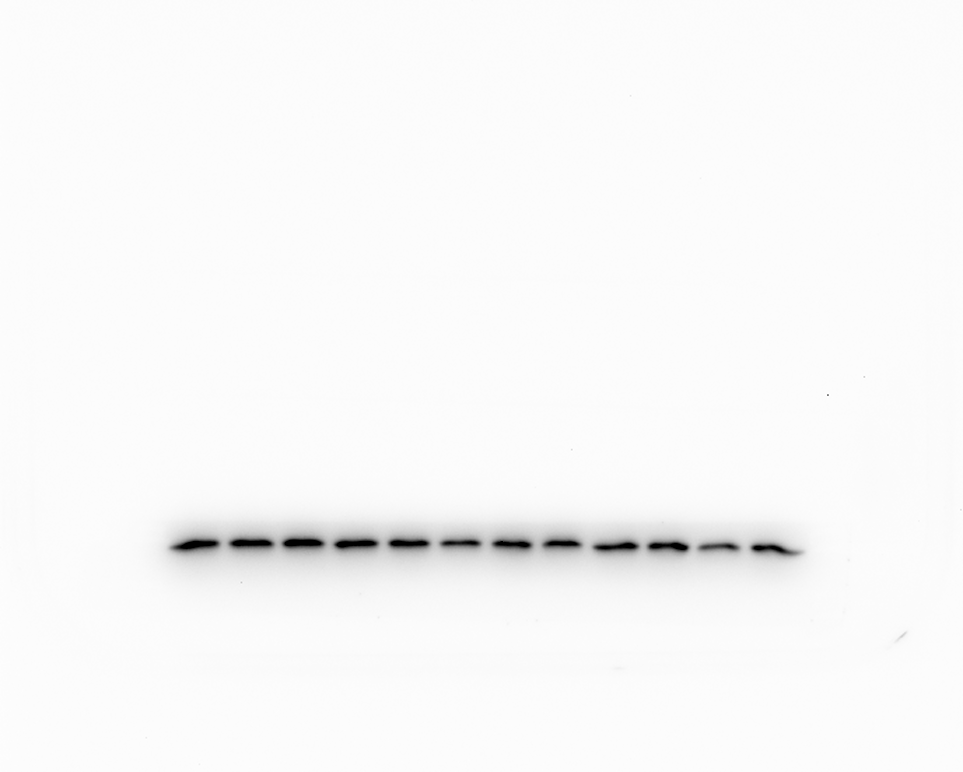

Supplement: Supplementary file 33 [file DataSheet5.ZIP › P38-1/p-p38 CYPB5.tif]

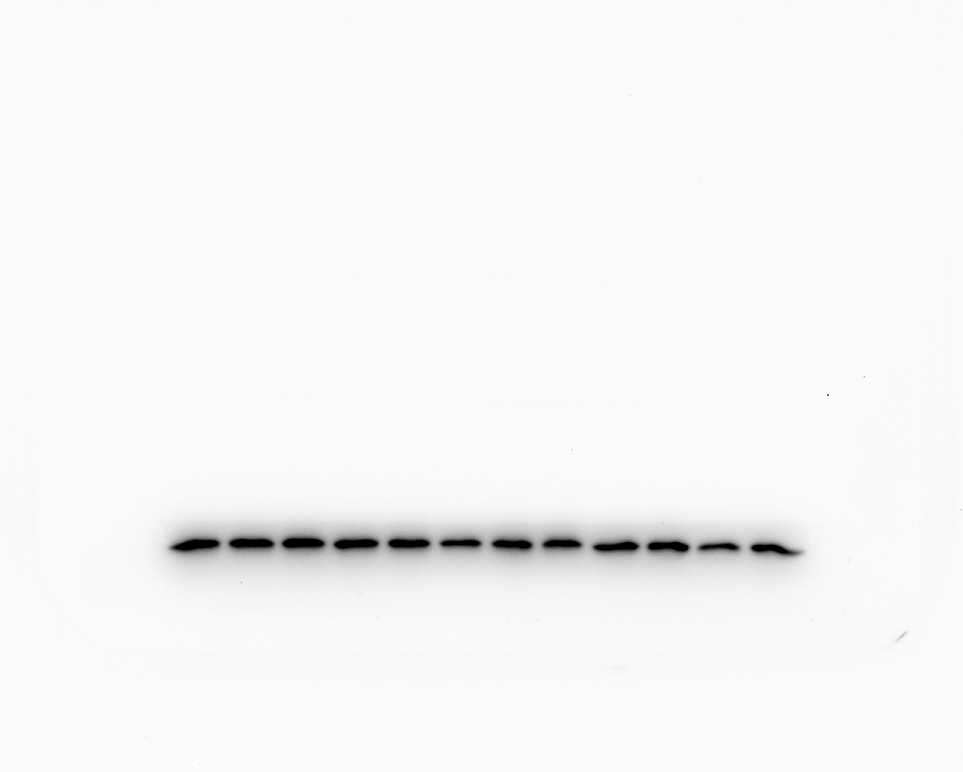

Supplement: Supplementary file 33 [file DataSheet5.ZIP › P38-1/p-p38 CYPBgb.tif]

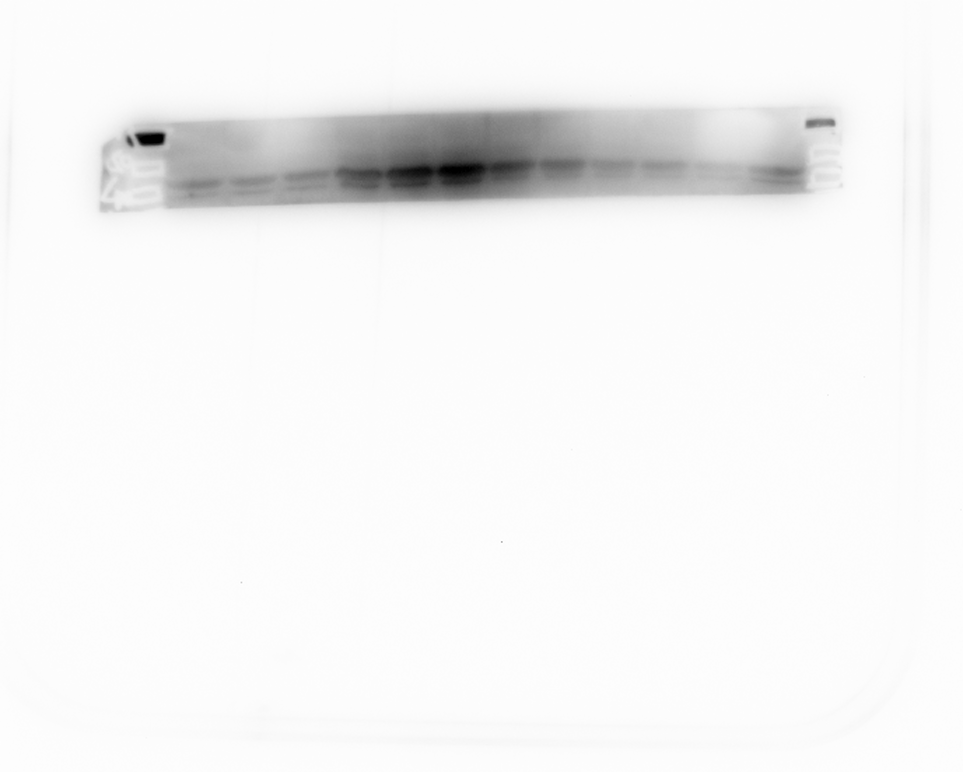

Supplement: Supplementary file 33 [file DataSheet5.ZIP › P38-1/p-p38-1.tif]

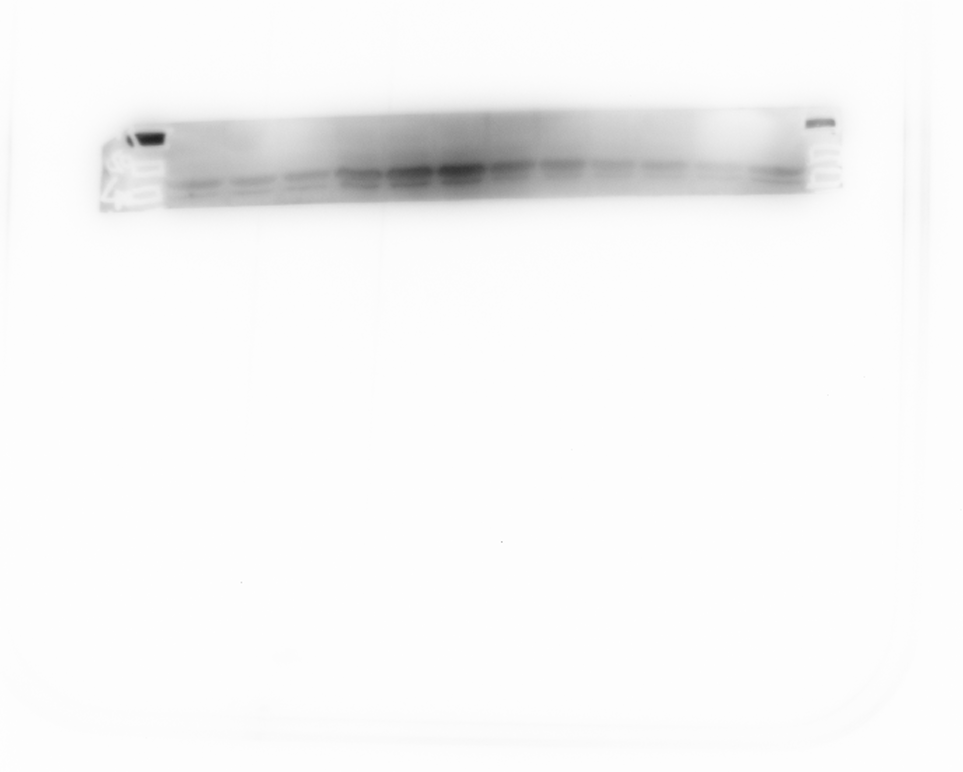

Supplement: Supplementary file 33 [file DataSheet5.ZIP › P38-1/p-p38-2.tif]

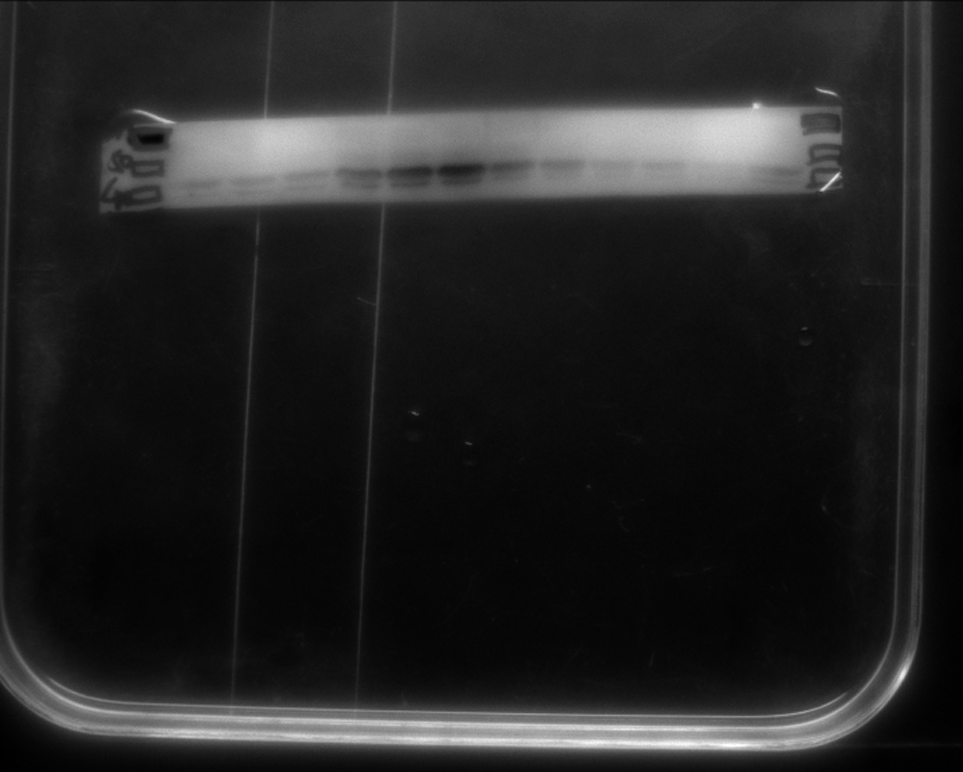

Supplement: Supplementary file 33 [file DataSheet5.ZIP › P38-1/p-p38-q.tif]

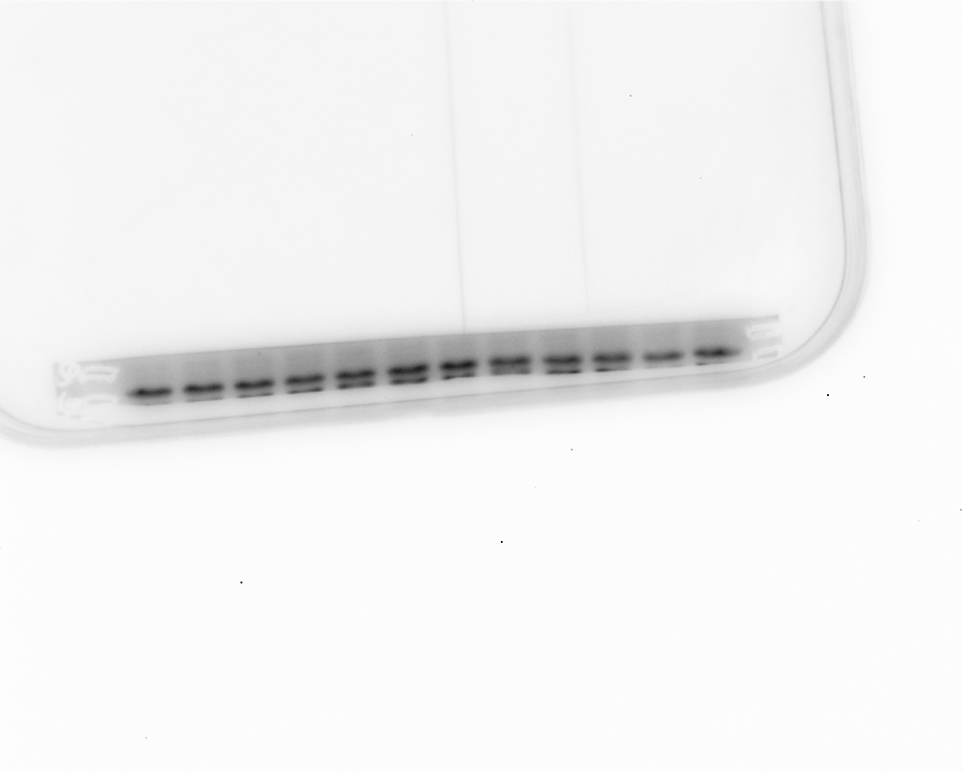

Supplement: Supplementary file 33 [file DataSheet5.ZIP › P38-1/p38 1.tif]

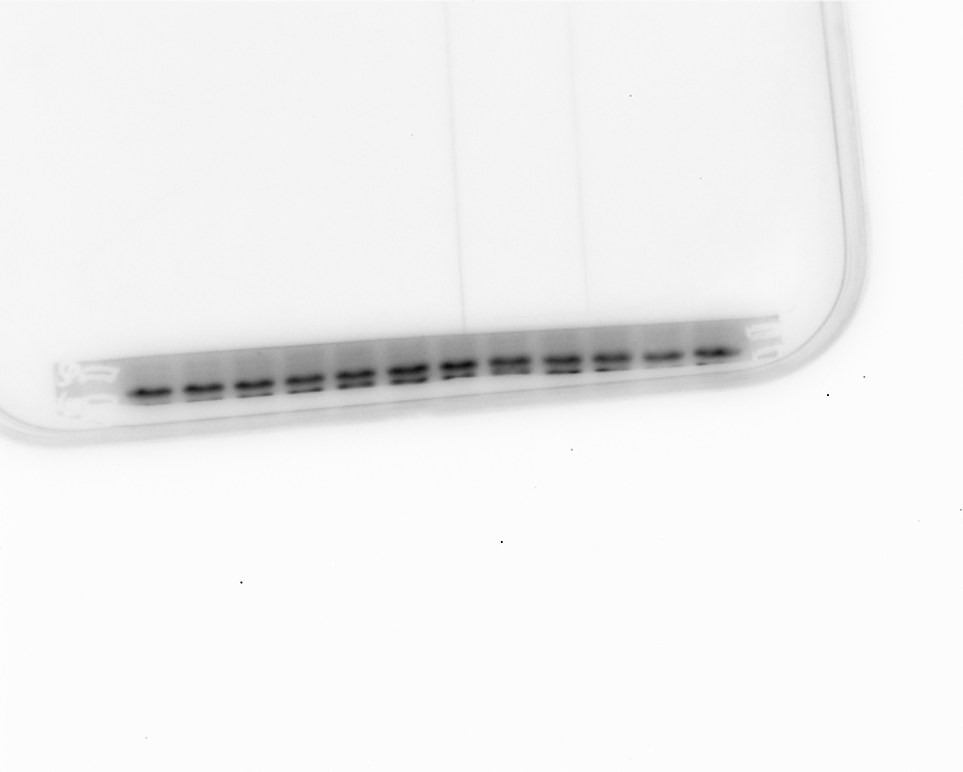

Supplement: Supplementary file 33 [file DataSheet5.ZIP › P38-1/p38 2.tif]

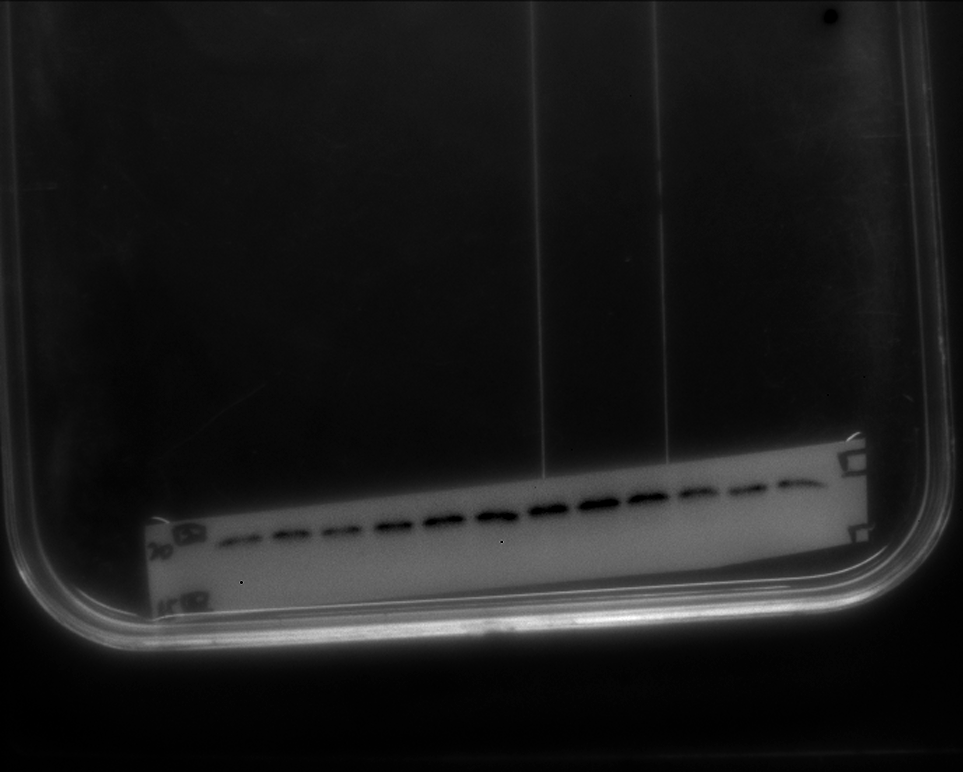

Supplement: Supplementary file 33 [file DataSheet5.ZIP › P38-1/p38 CYPB q.tif]

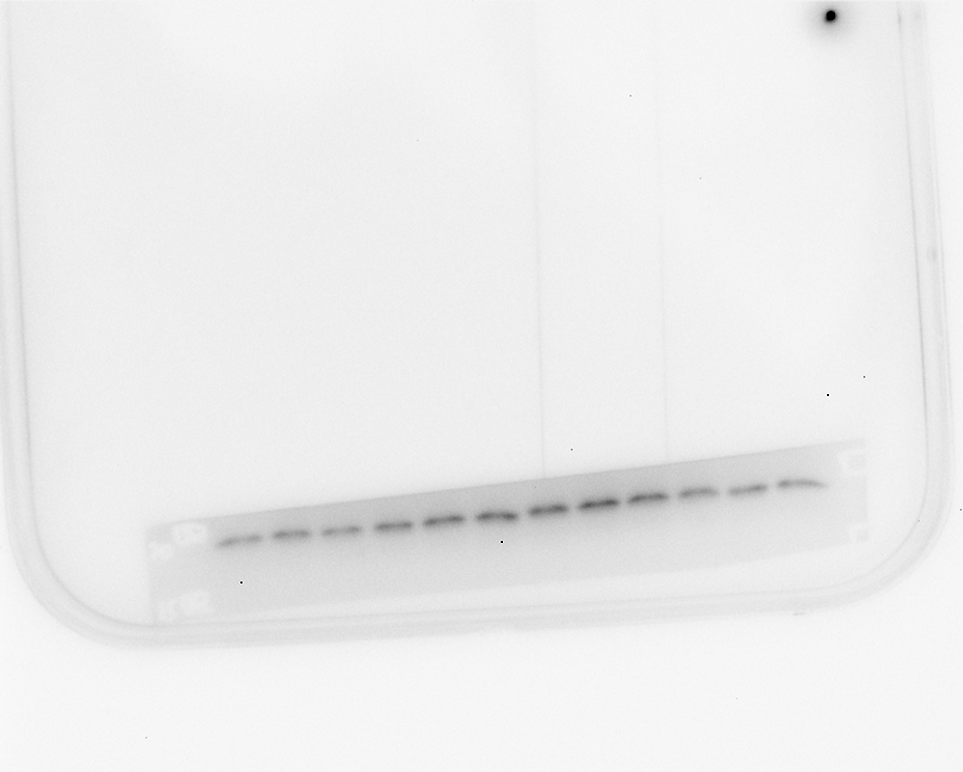

Supplement: Supplementary file 33 [file DataSheet5.ZIP › P38-1/p38 CYPB2.tif]

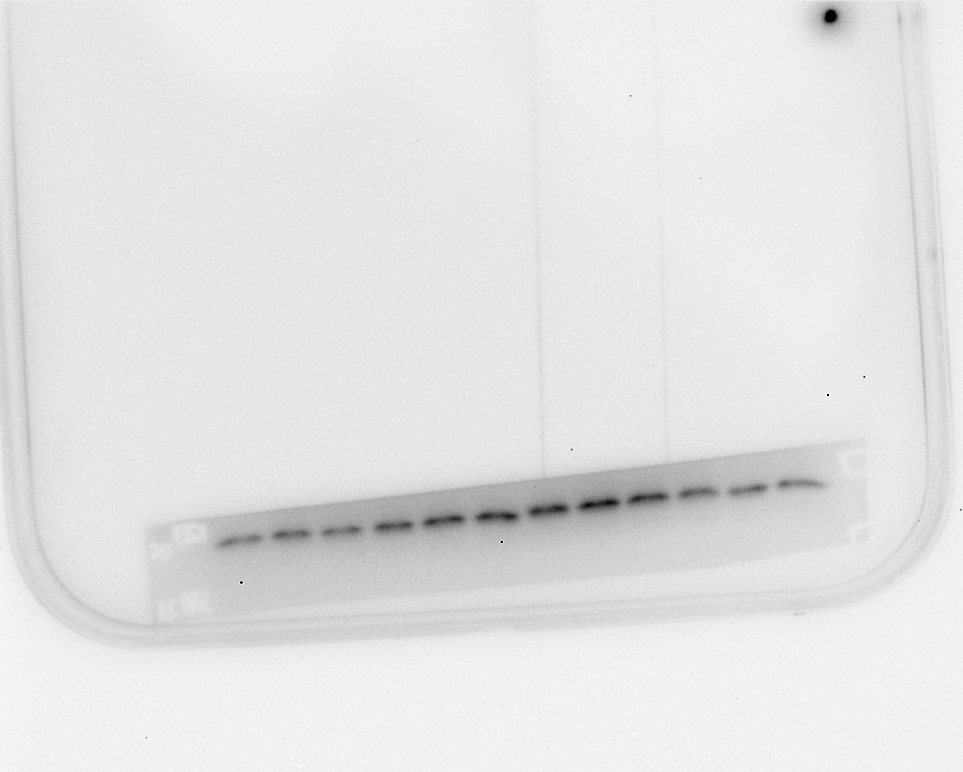

Supplement: Supplementary file 33 [file DataSheet5.ZIP › P38-1/p38 CYPB3.tif]

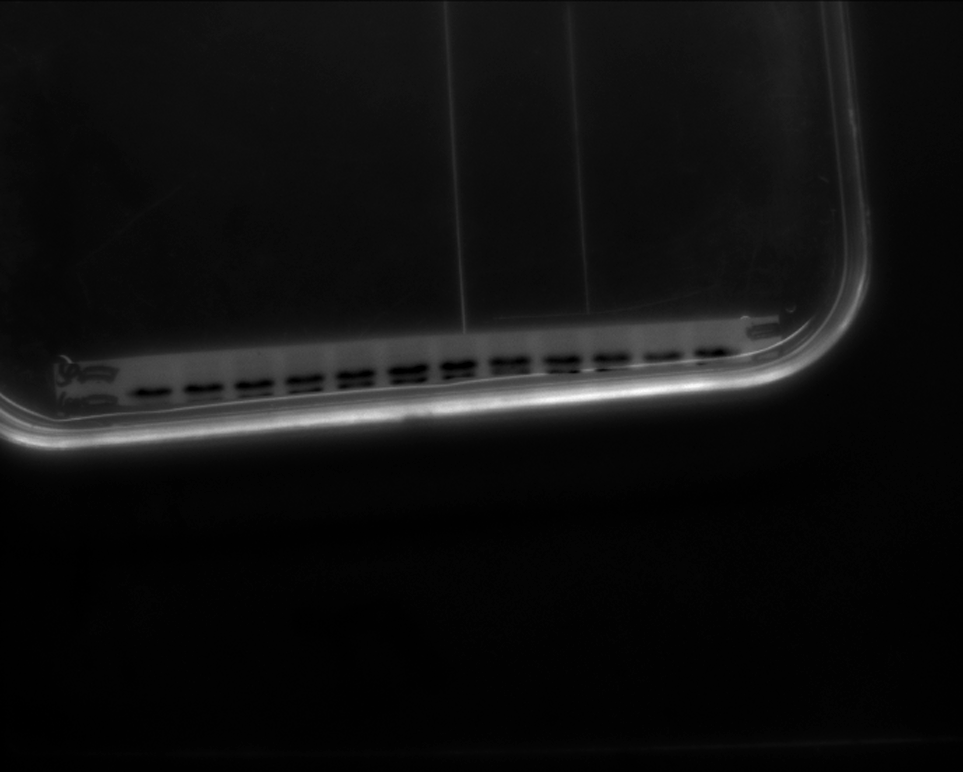

Supplement: Supplementary file 33 [file DataSheet5.ZIP › P38-1/p38 q.tif]

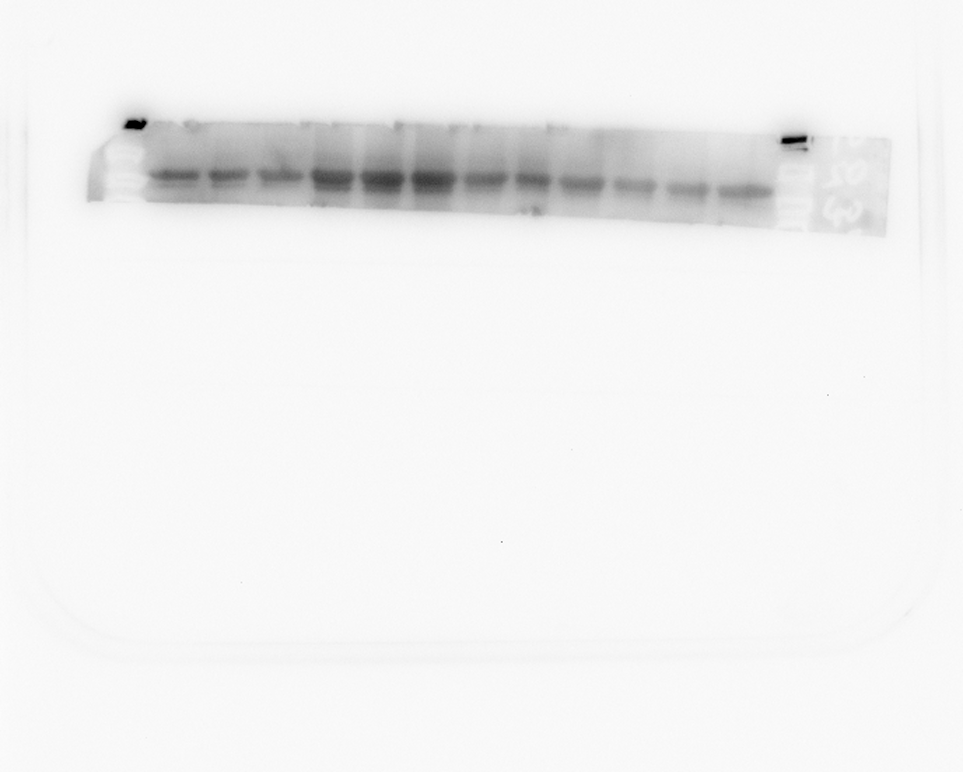

Supplement: Supplementary file 33 [file DataSheet5.ZIP › P38-2/p-p38 1.tif]

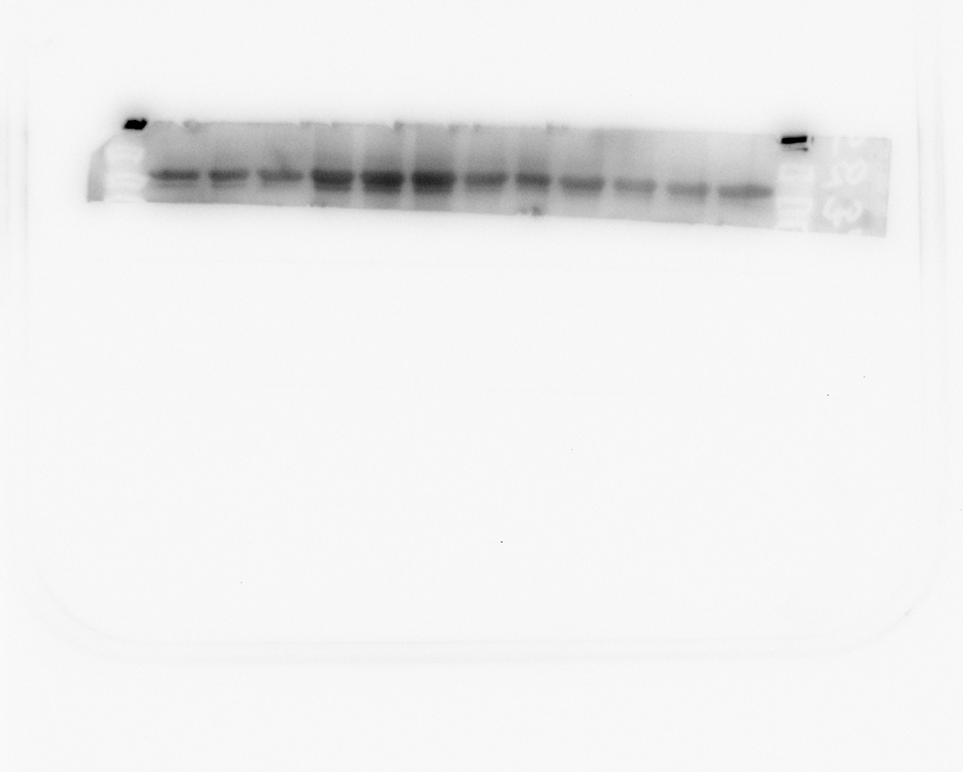

Supplement: Supplementary file 33 [file DataSheet5.ZIP › P38-2/p-p38 2.tif]

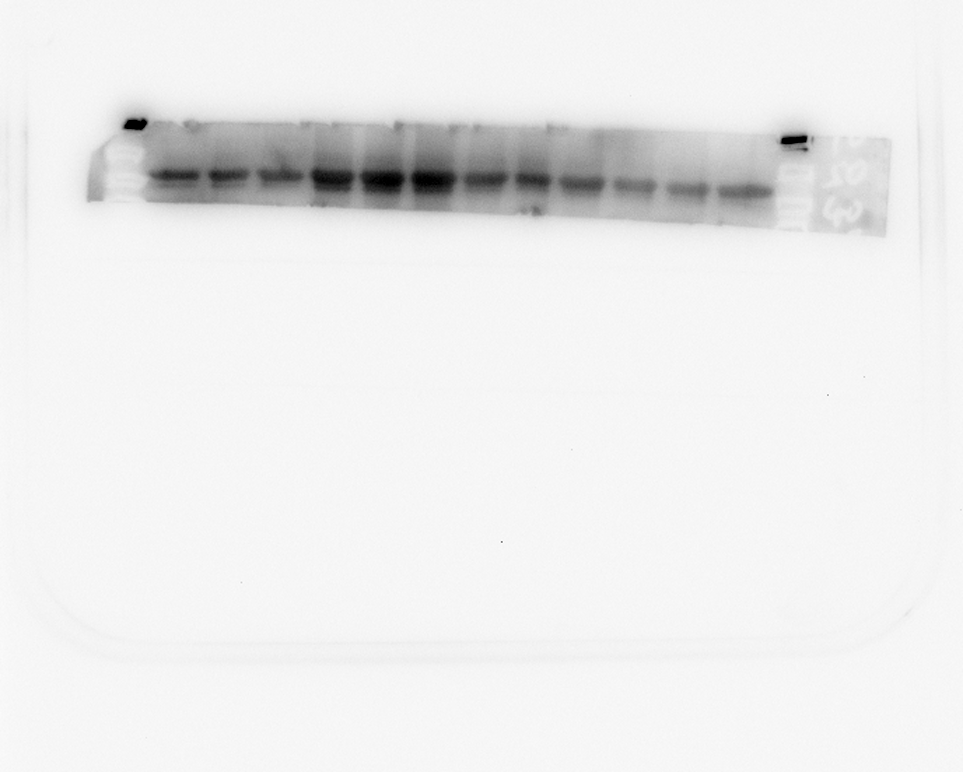

Supplement: Supplementary file 33 [file DataSheet5.ZIP › P38-2/p-p38 3.tif]

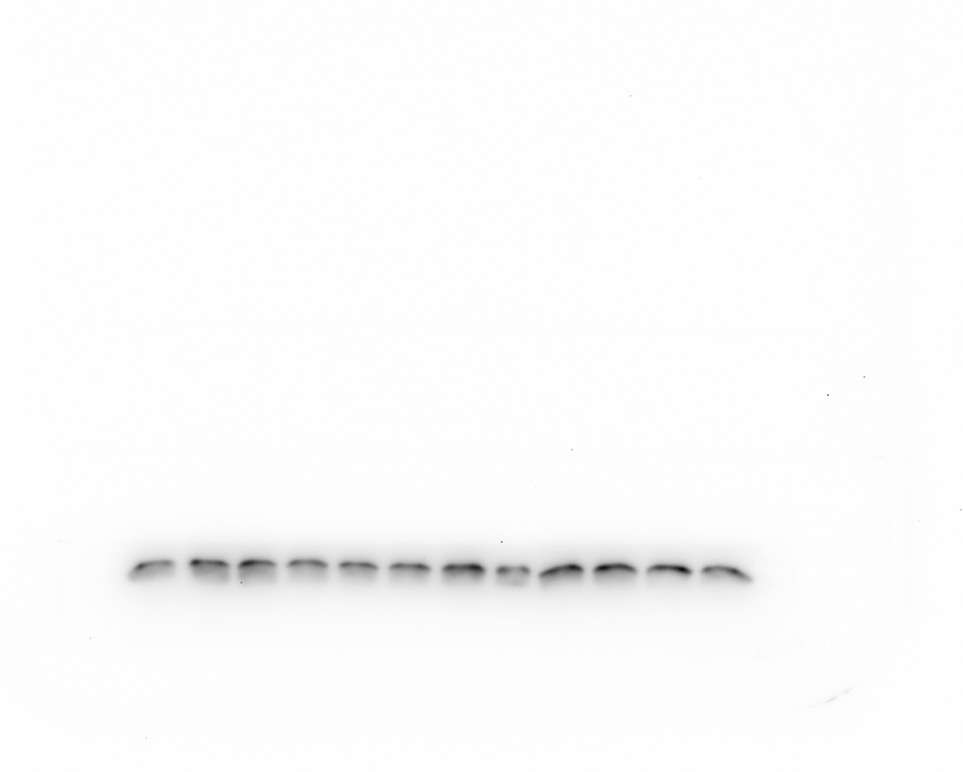

Supplement: Supplementary file 33 [file DataSheet5.ZIP › P38-2/p-p38 CYPB1.tif]

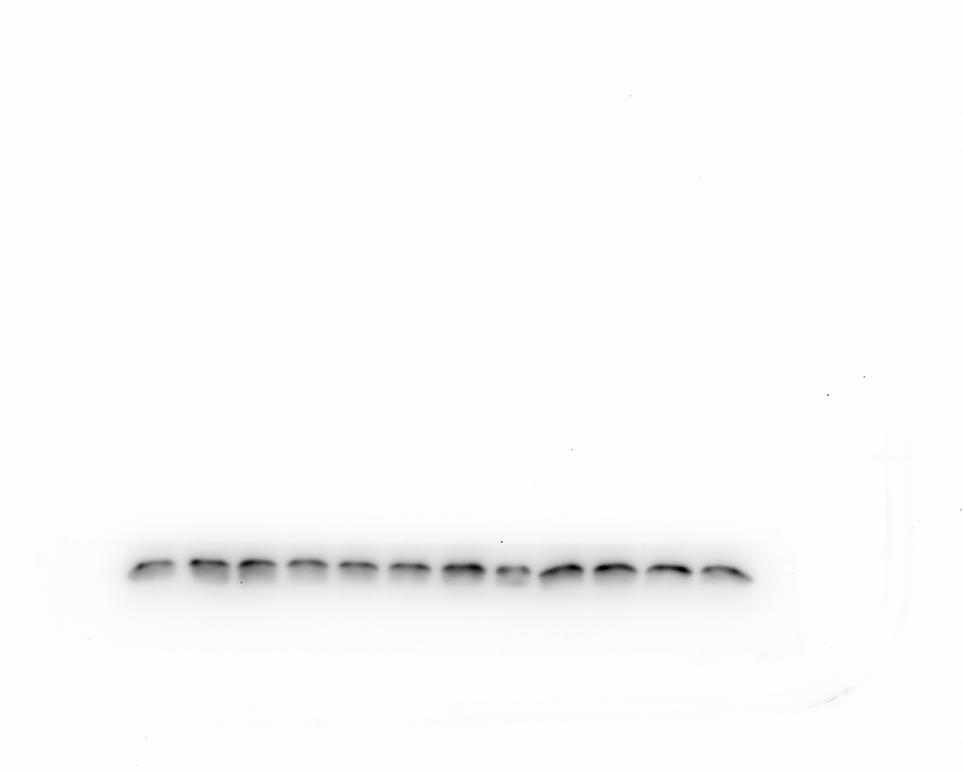

Supplement: Supplementary file 33 [file DataSheet5.ZIP › P38-2/p-p38 CYPB2.tif]

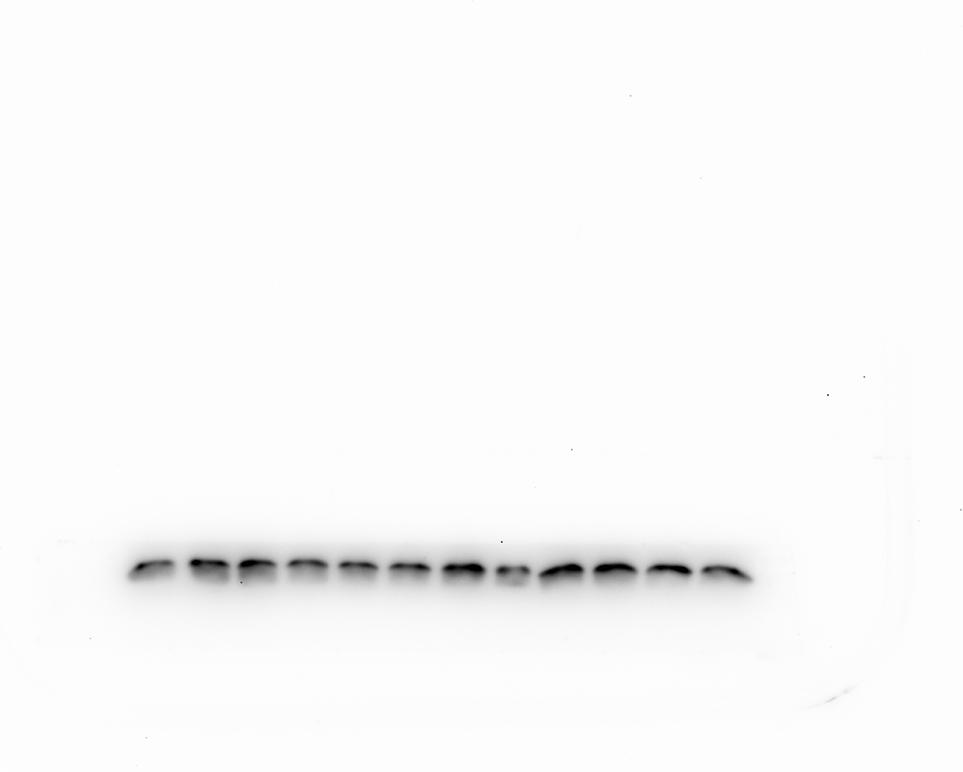

Supplement: Supplementary file 33 [file DataSheet5.ZIP › P38-2/p-p38 CYPB3.tif]

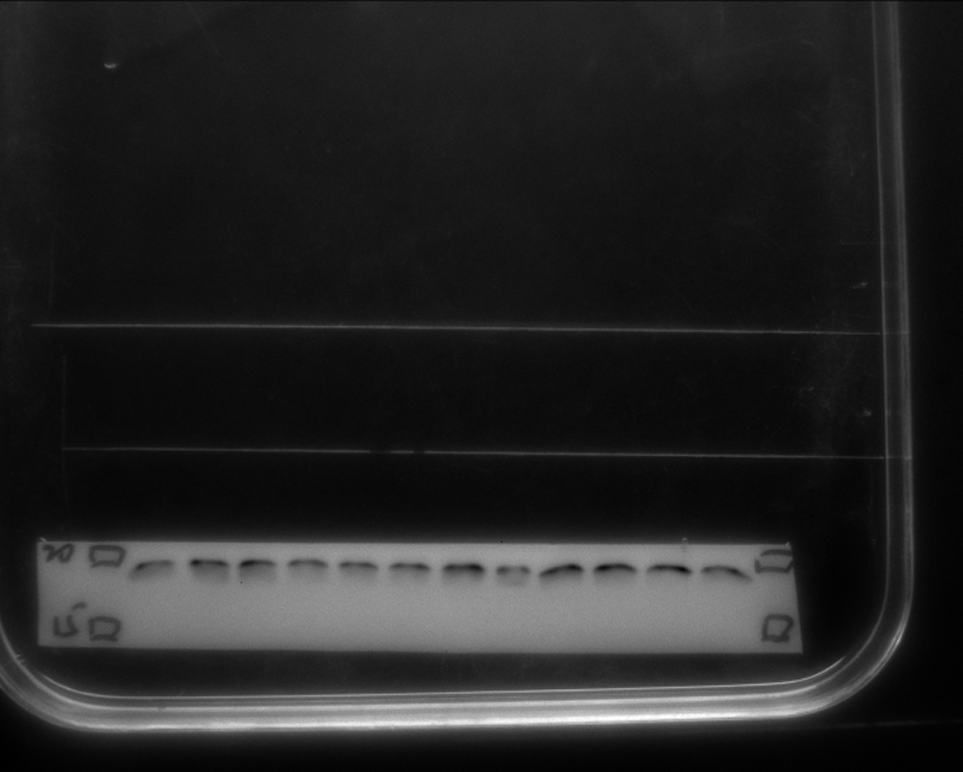

Supplement: Supplementary file 33 [file DataSheet5.ZIP › P38-2/p-p38 CYPBq.tif]

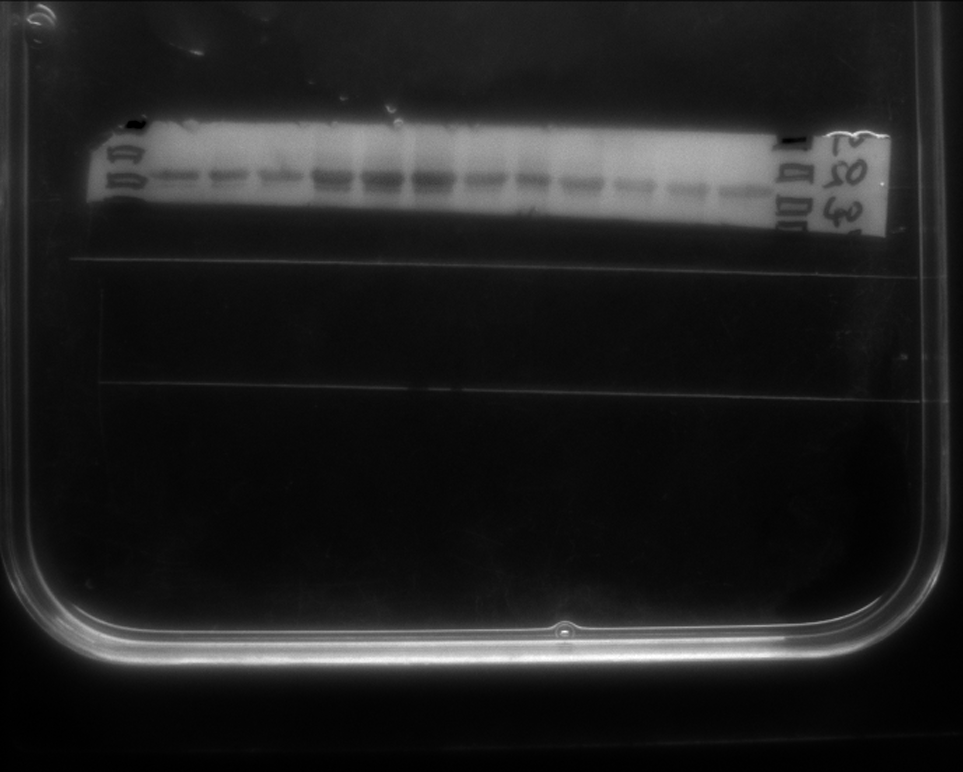

Supplement: Supplementary file 33 [file DataSheet5.ZIP › P38-2/p-p38 q.tif]

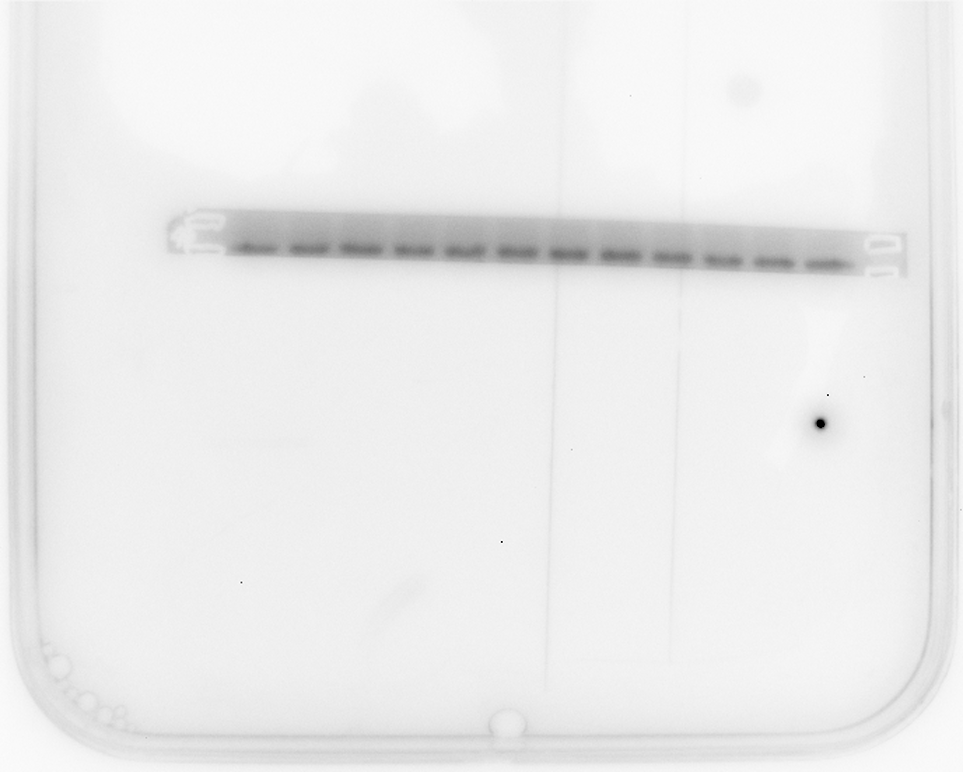

Supplement: Supplementary file 33 [file DataSheet5.ZIP › P38-2/p38 1.tif]

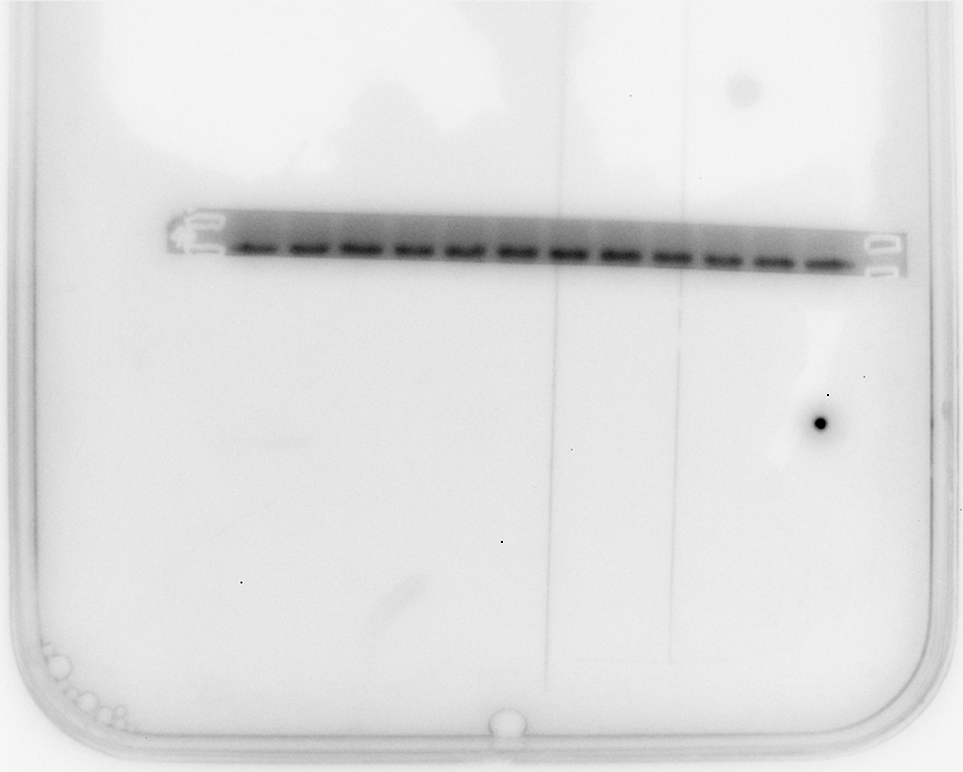

Supplement: Supplementary file 33 [file DataSheet5.ZIP › P38-2/p38 2.tif]

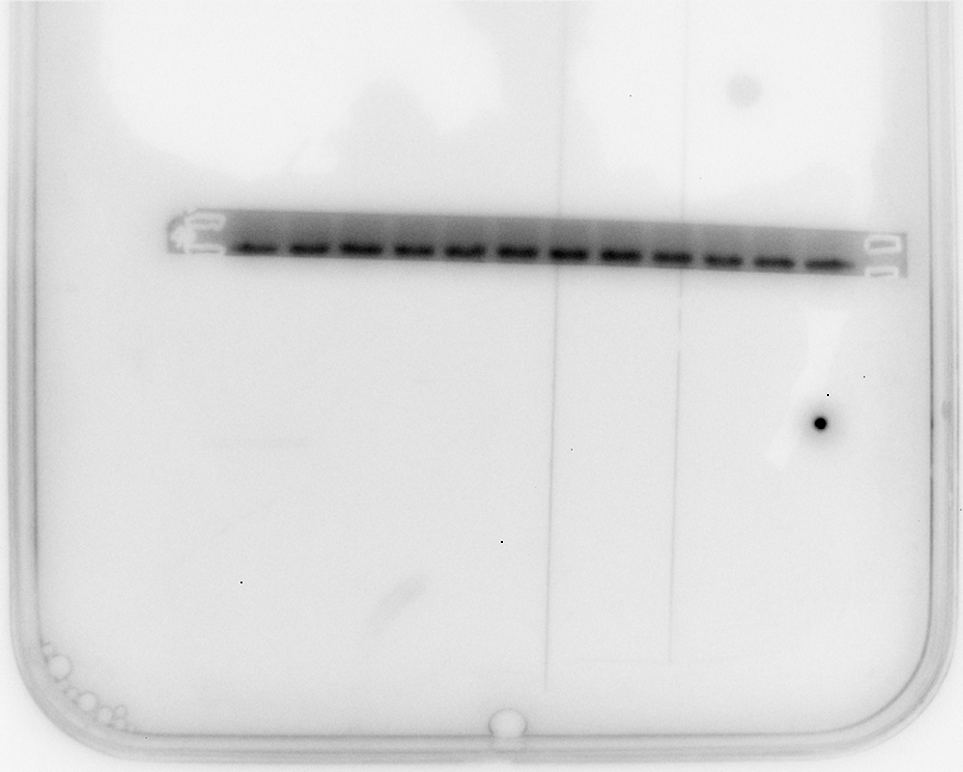

Supplement: Supplementary file 33 [file DataSheet5.ZIP › P38-2/p38 3.tif]

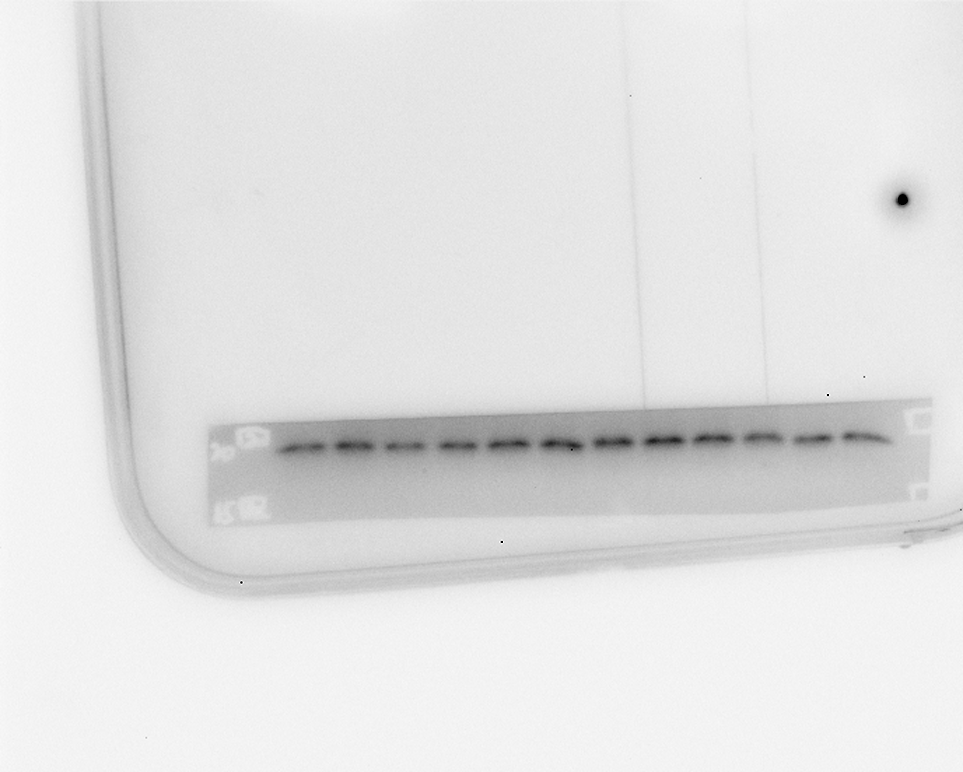

Supplement: Supplementary file 33 [file DataSheet5.ZIP › P38-2/p38 CYPB 1.tif]

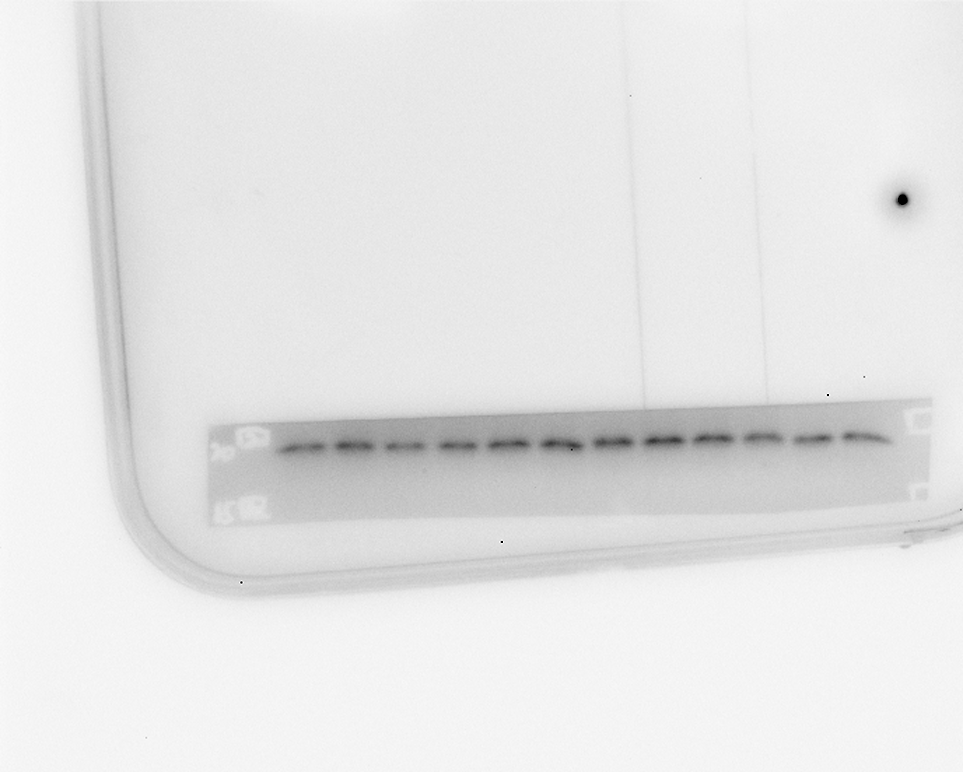

Supplement: Supplementary file 33 [file DataSheet5.ZIP › P38-2/p38 CYPB 2.tif]

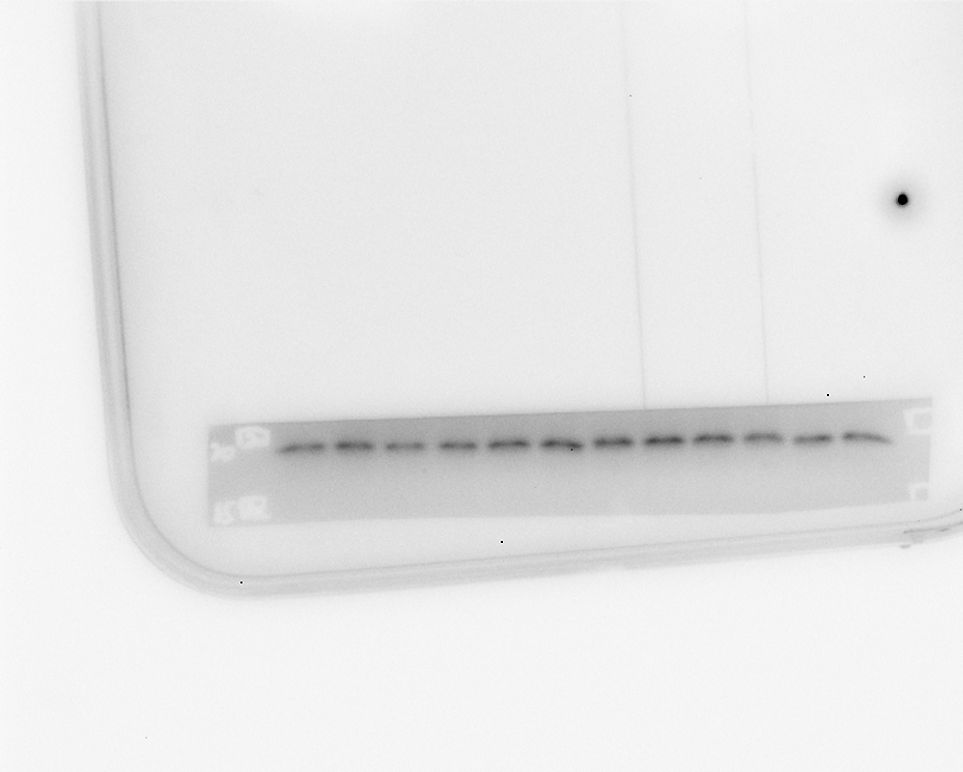

Supplement: Supplementary file 33 [file DataSheet5.ZIP › P38-2/p38 CYPB 3.tif]

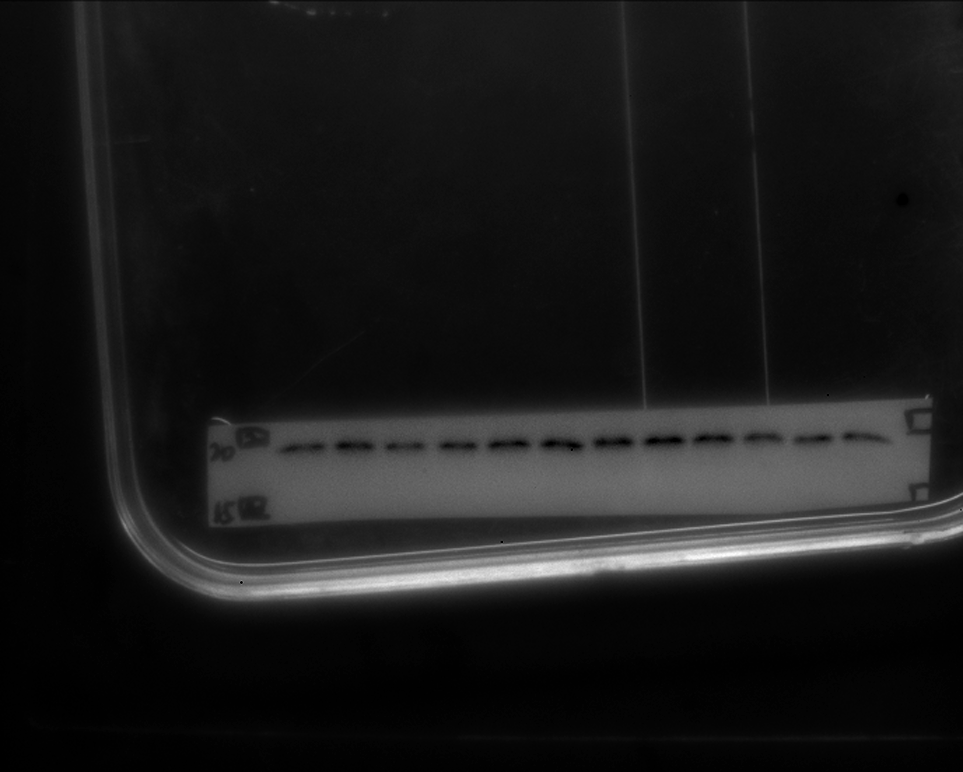

Supplement: Supplementary file 33 [file DataSheet5.ZIP › P38-2/p38 CYPB.tif]

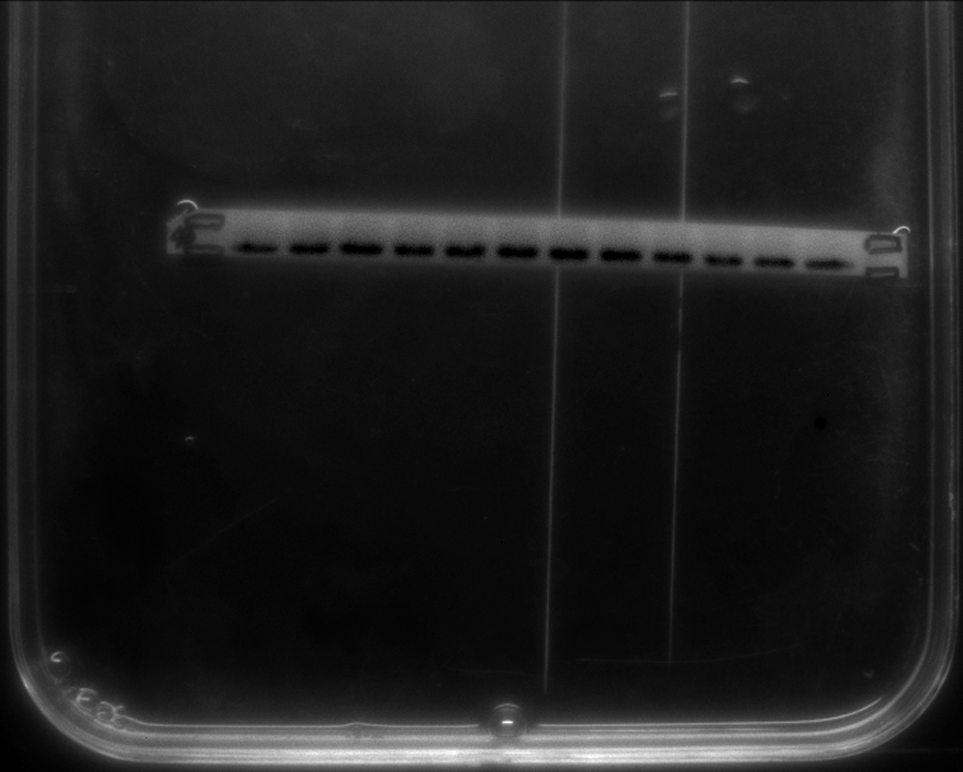

Supplement: Supplementary file 33 [file DataSheet5.ZIP › P38-2/p38 q.tif]

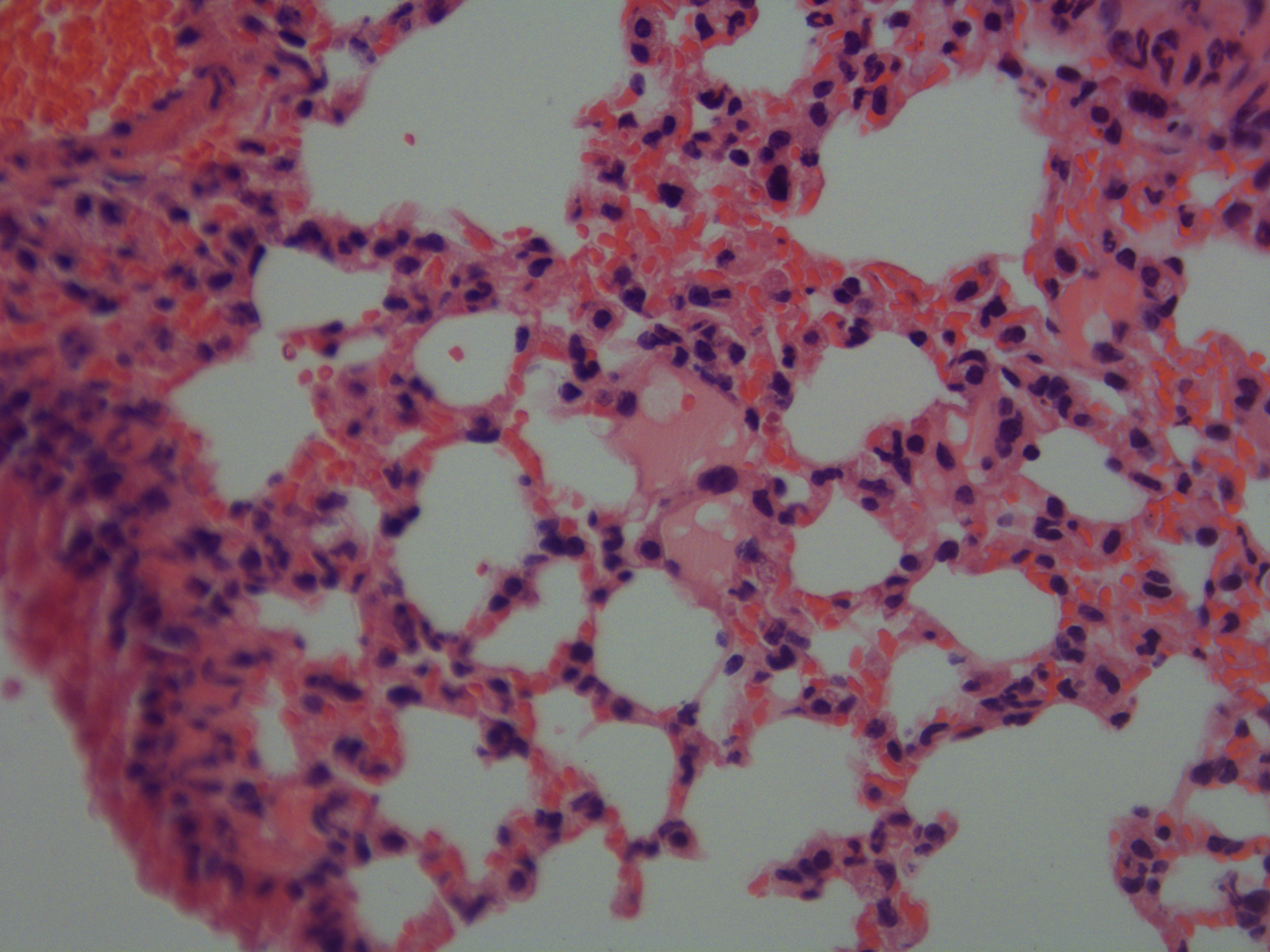

Supplement: Supplementary file 34 [file Image12.TIF]

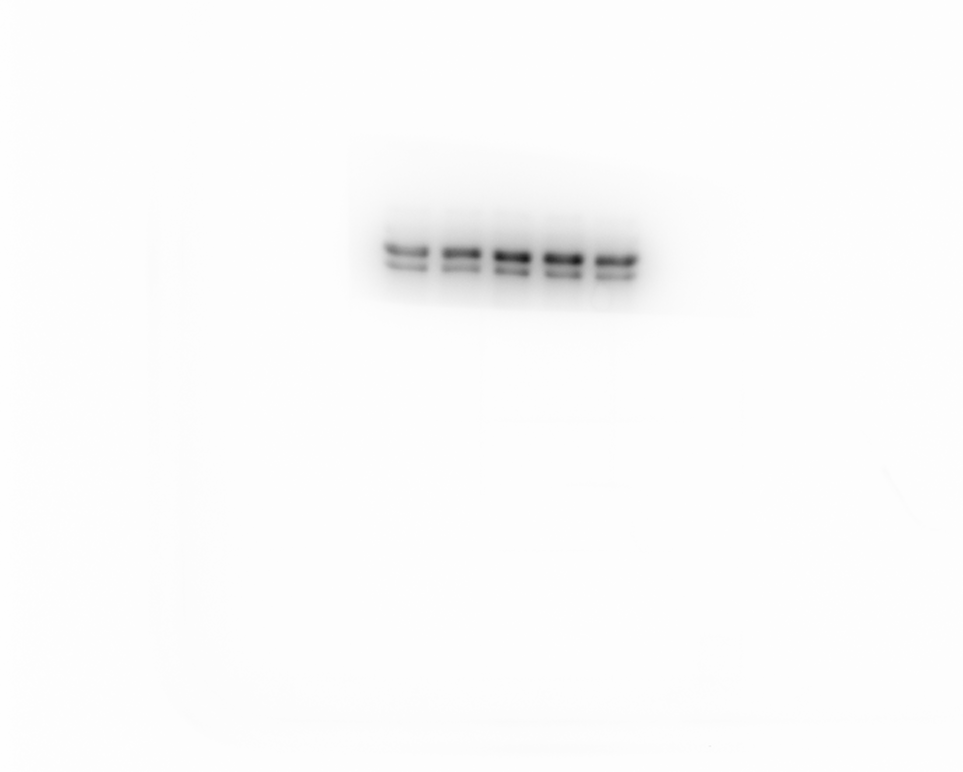

Supplement: Supplementary file 35 [file DataSheet7.ZIP › ERK-1/ERK 1.tif]
