# Supplementary figures and images for: Valsartan attenuates LPS-induced ALI by modulating NF-κB and MAPK pathways (part 4 of 4)
Source: Front Pharmacol. 2024 Jan 15;15:1321095. doi: 10.3389/fphar.2024.1321095 (PMC10822936; doi:10.3389/fphar.2024.1321095)

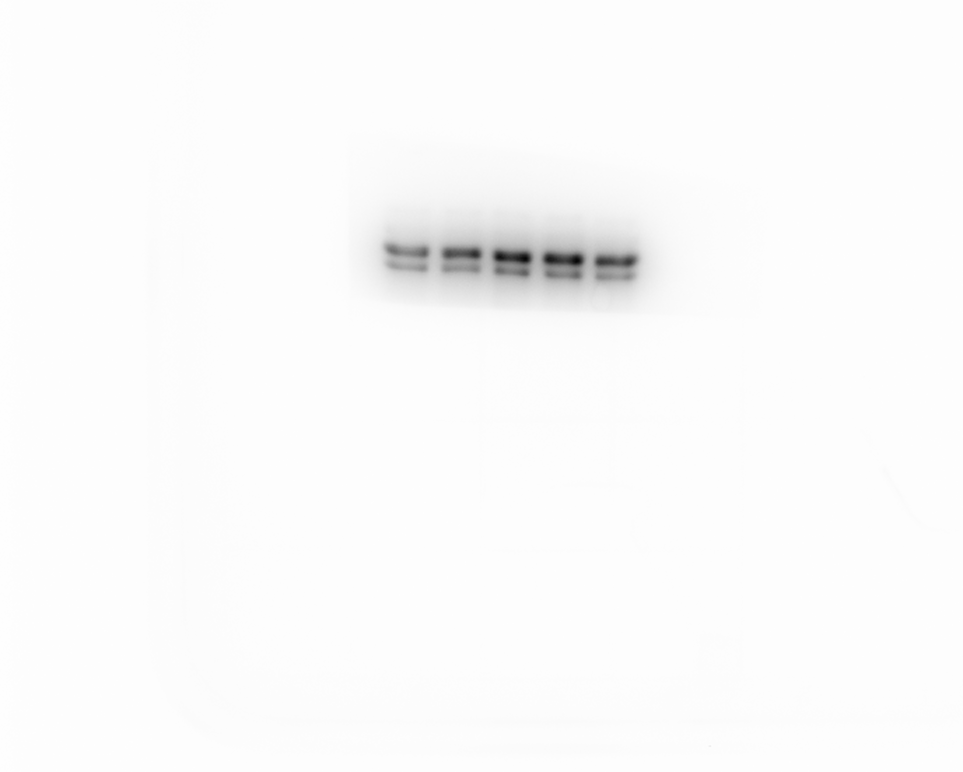

Supplement: Supplementary file 35 [file DataSheet7.ZIP › ERK-1/ERK 2.tif]

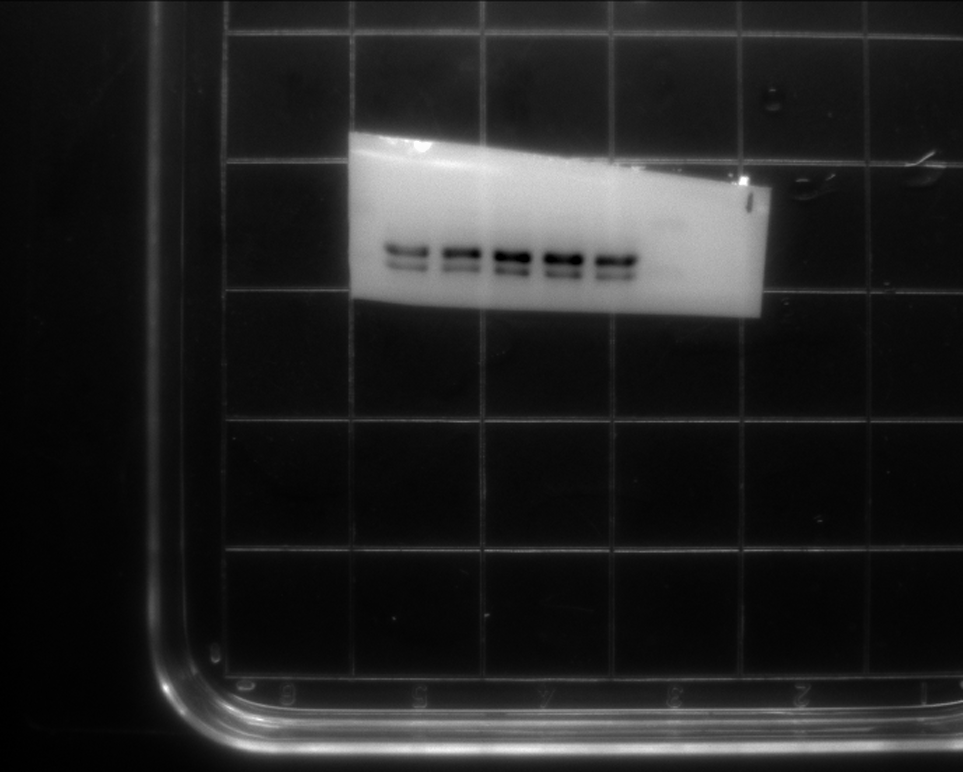

Supplement: Supplementary file 35 [file DataSheet7.ZIP › ERK-1/ERK q.tif]

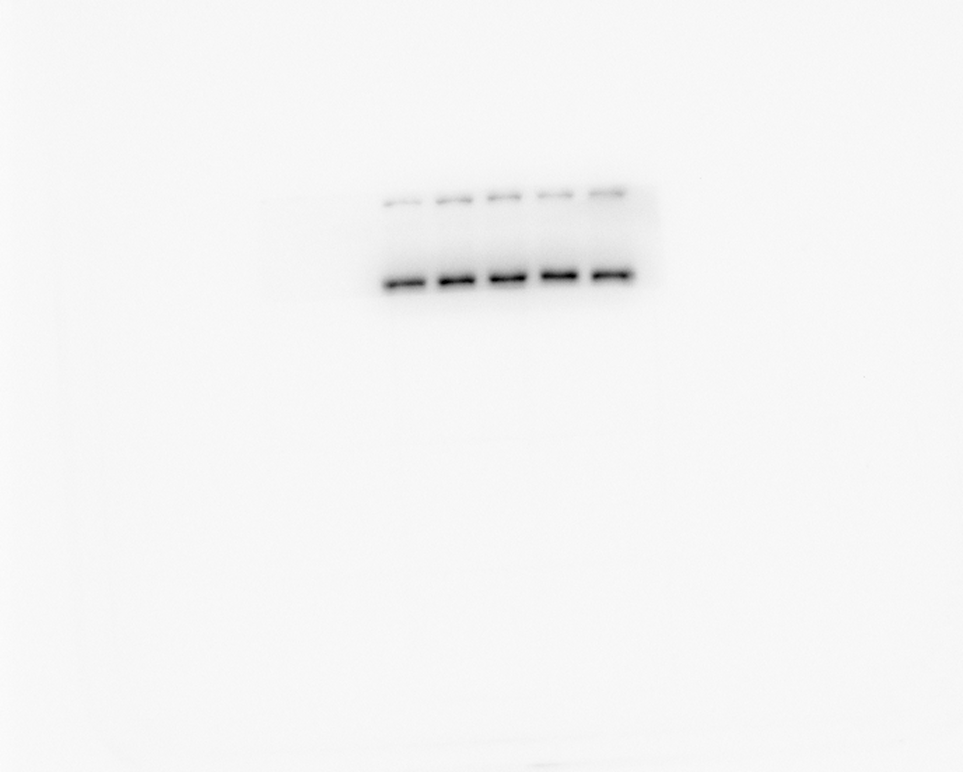

Supplement: Supplementary file 35 [file DataSheet7.ZIP › ERK-1/ERK tublin 1.tif]

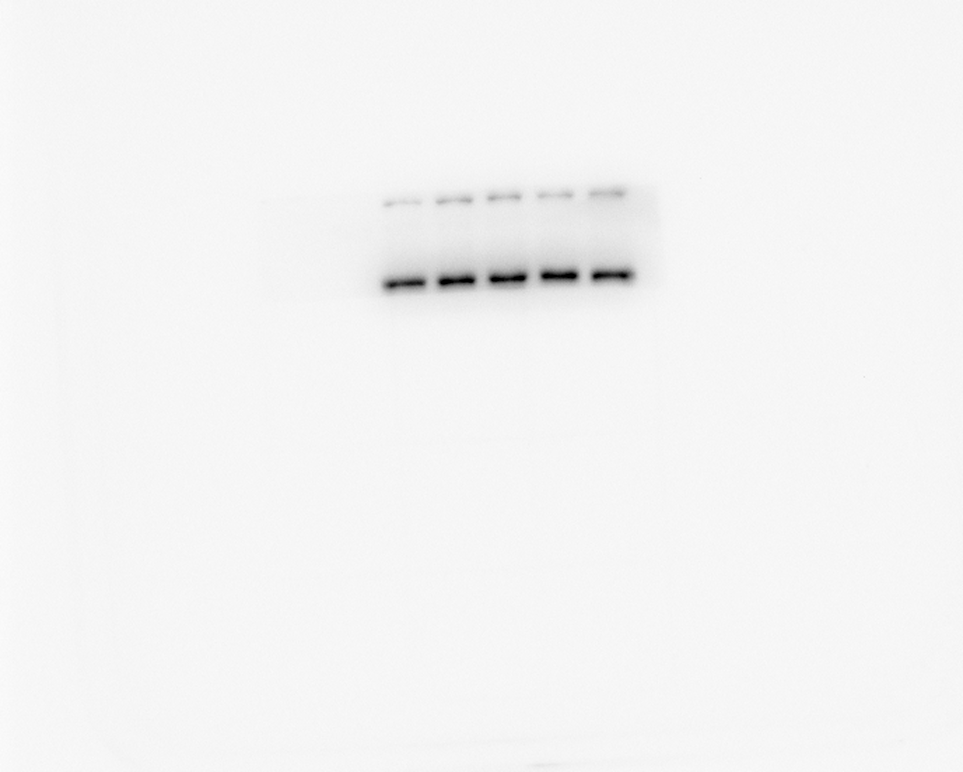

Supplement: Supplementary file 35 [file DataSheet7.ZIP › ERK-1/ERK tublin 2.tif]

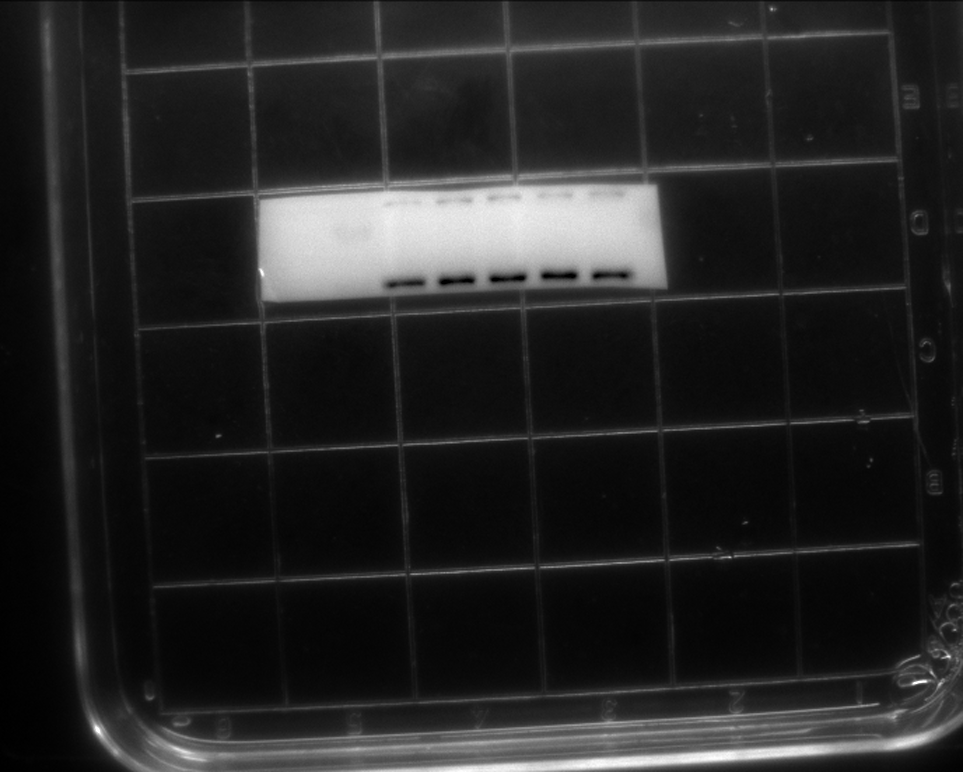

Supplement: Supplementary file 35 [file DataSheet7.ZIP › ERK-1/ERK tublin.tif]

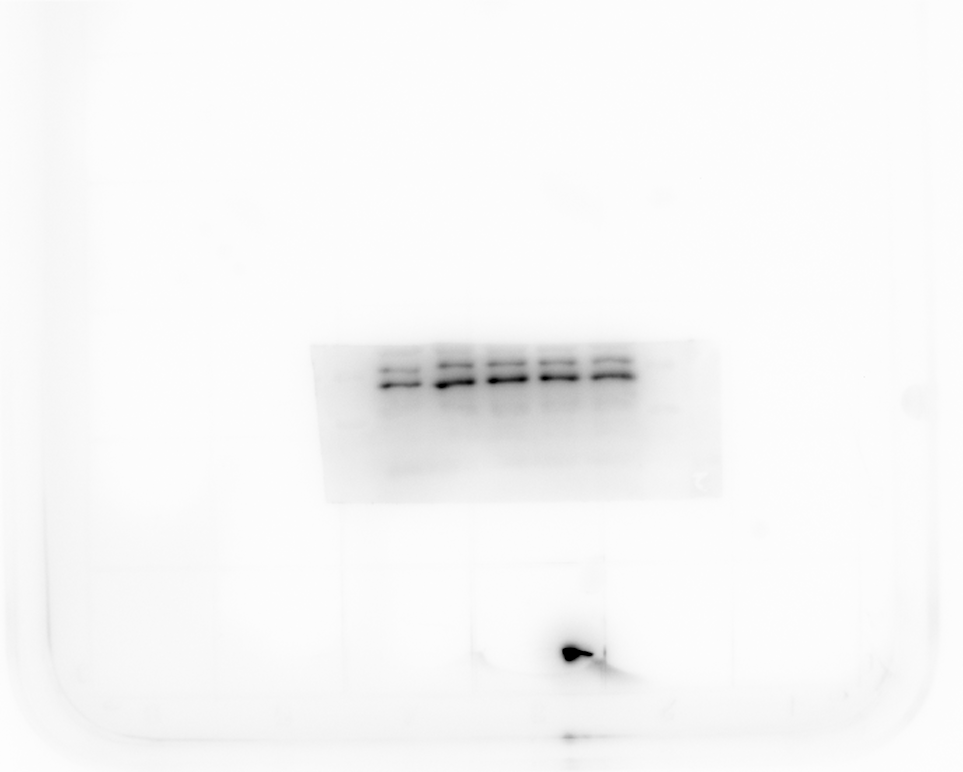

Supplement: Supplementary file 35 [file DataSheet7.ZIP › ERK-1/P-ERK 1.tif]

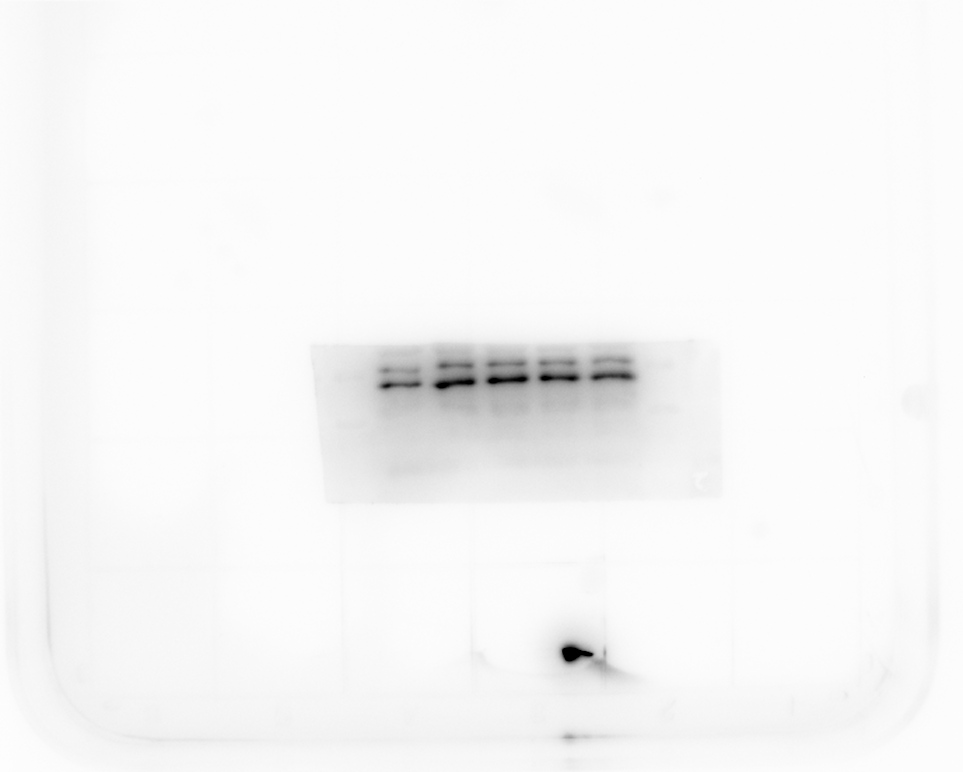

Supplement: Supplementary file 35 [file DataSheet7.ZIP › ERK-1/P-ERK 2.tif]

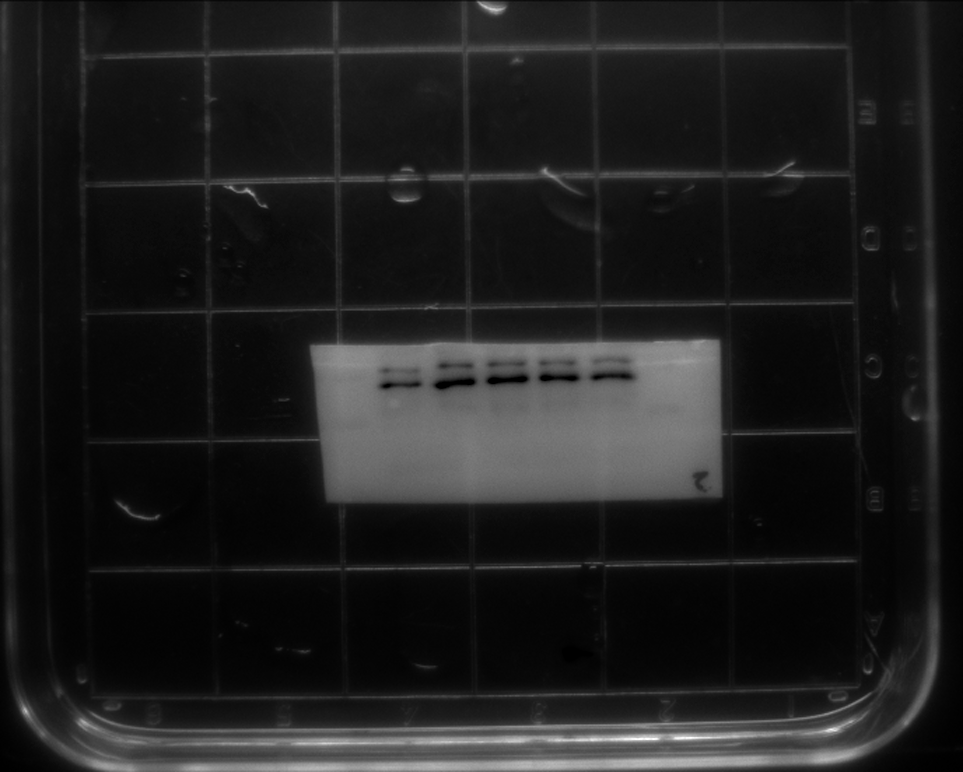

Supplement: Supplementary file 35 [file DataSheet7.ZIP › ERK-1/P-ERK Q.tif]

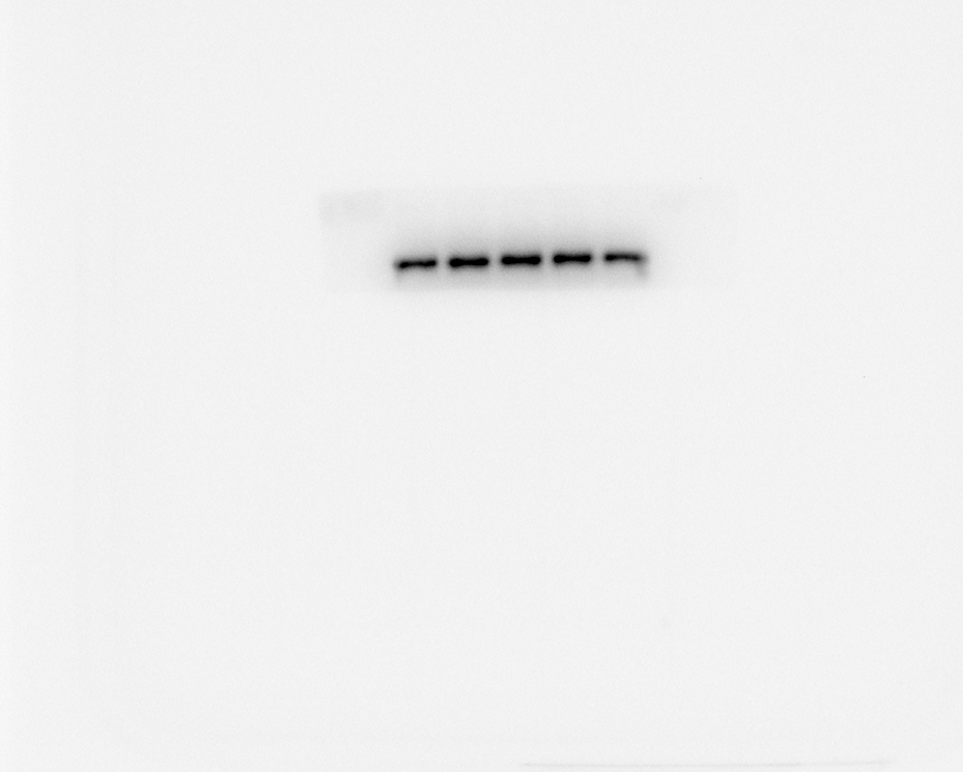

Supplement: Supplementary file 35 [file DataSheet7.ZIP › ERK-1/P-ERK tublin 2.tif]

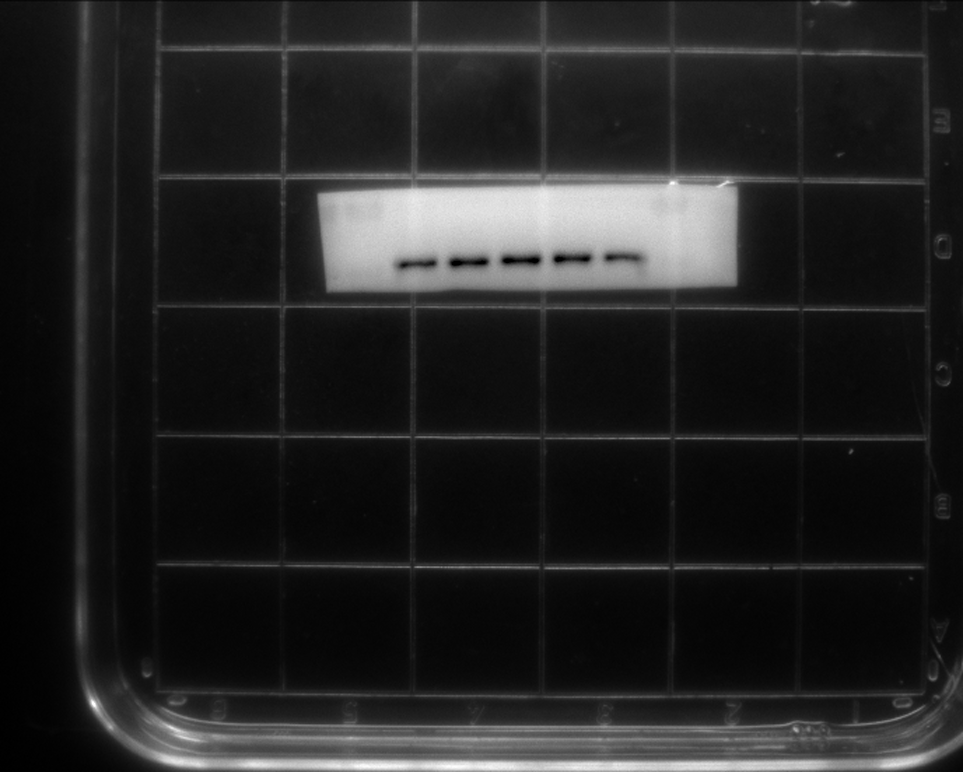

Supplement: Supplementary file 35 [file DataSheet7.ZIP › ERK-1/P-ERK tublin q.tif]

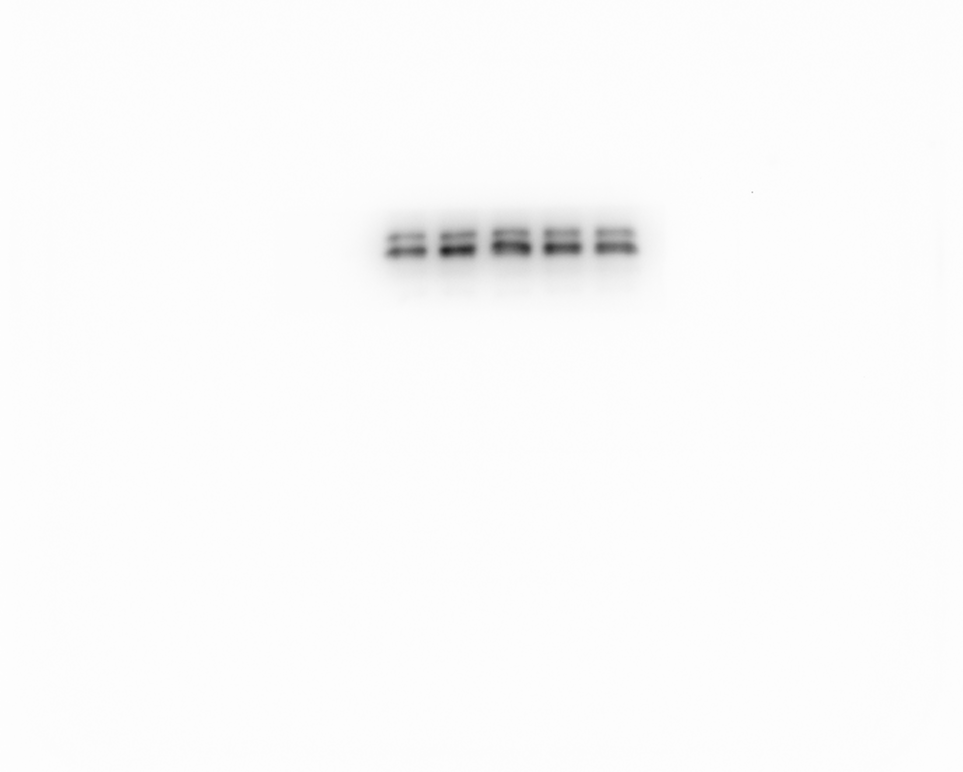

Supplement: Supplementary file 35 [file DataSheet7.ZIP › ERK-2/ERK 1.tif]

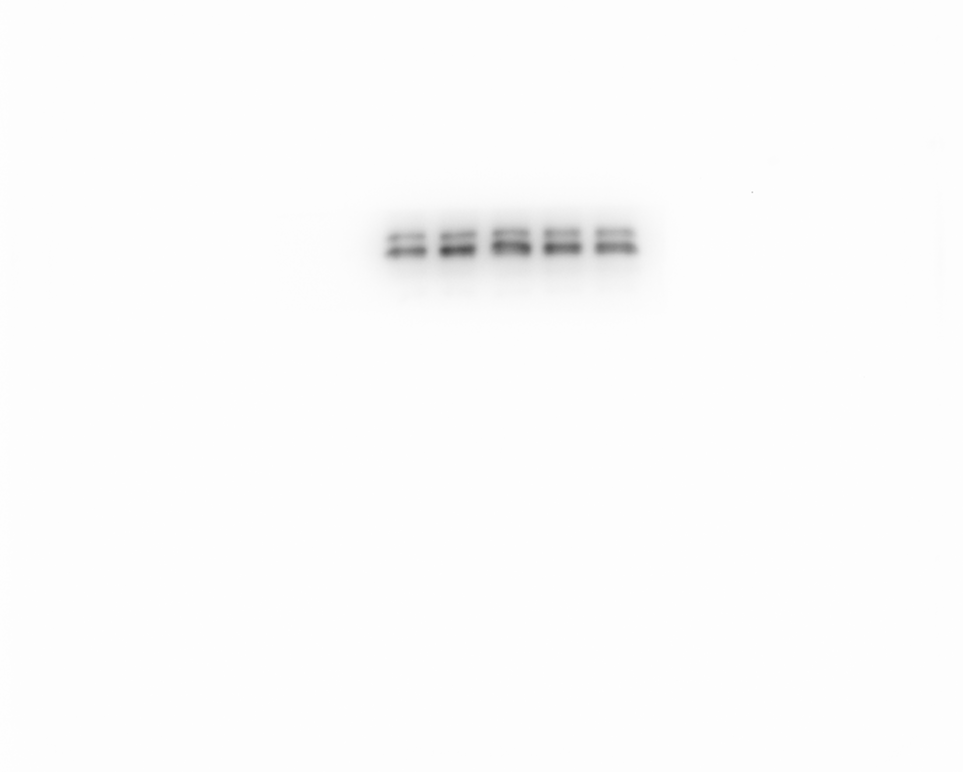

Supplement: Supplementary file 35 [file DataSheet7.ZIP › ERK-2/ERK 2.tif]

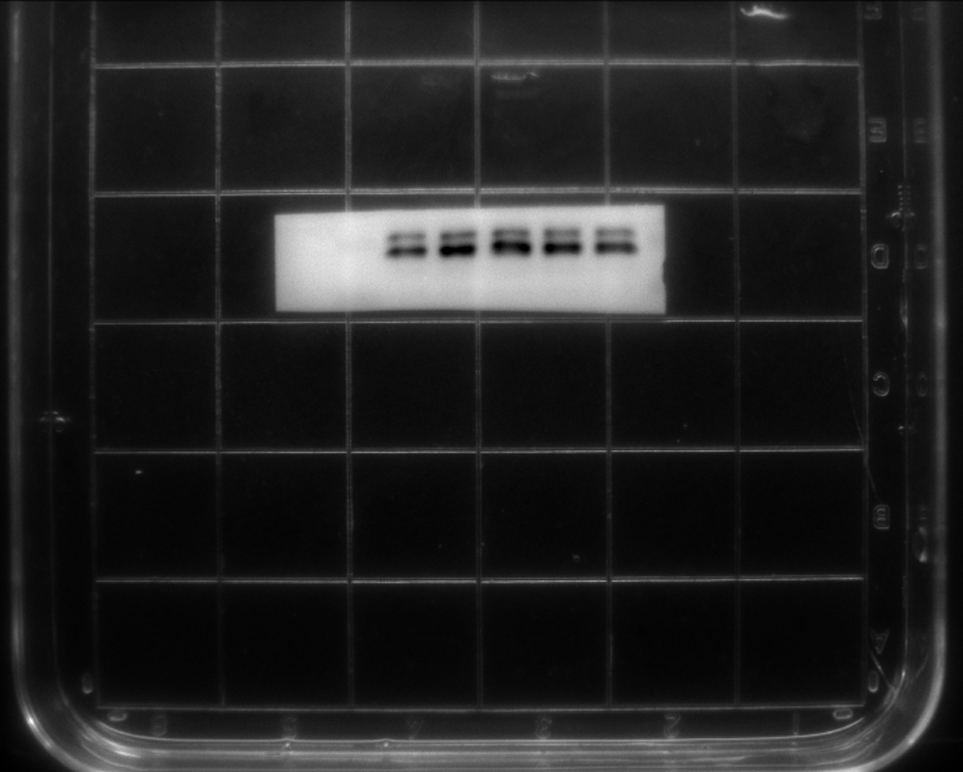

Supplement: Supplementary file 35 [file DataSheet7.ZIP › ERK-2/ERK q.tif]

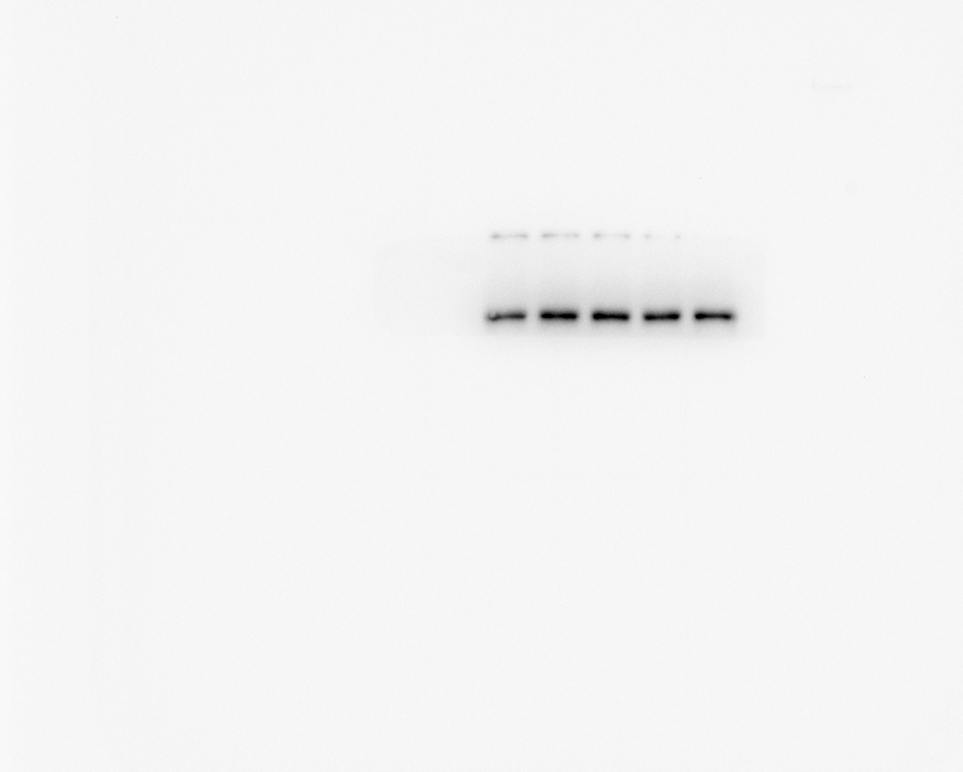

Supplement: Supplementary file 35 [file DataSheet7.ZIP › ERK-2/ERK tublin 2.tif]

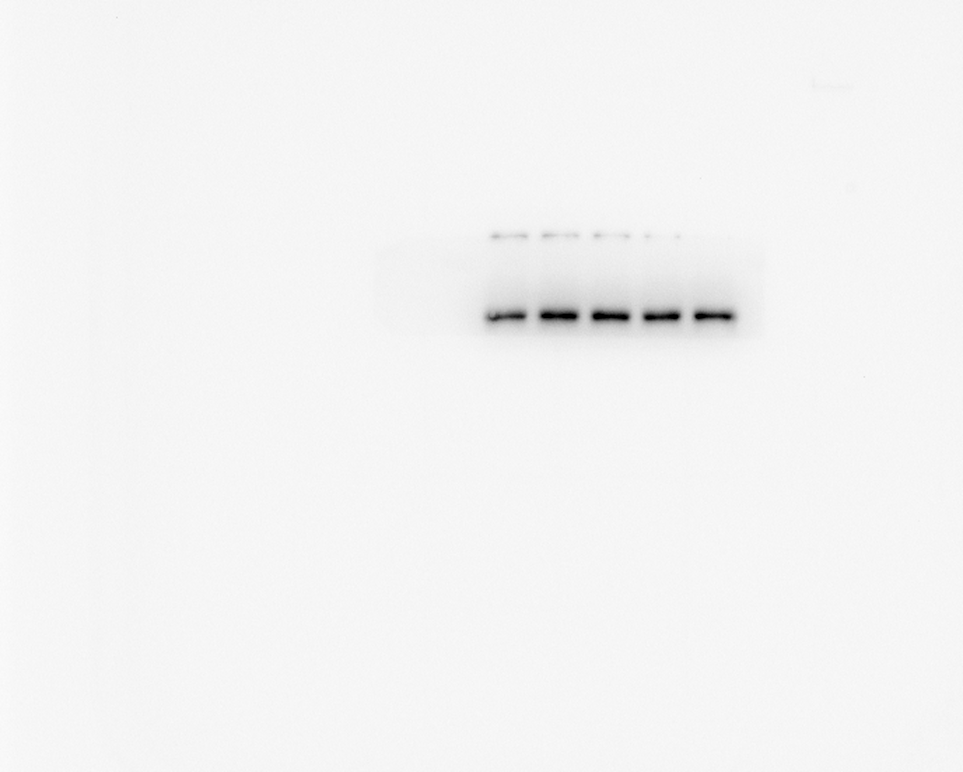

Supplement: Supplementary file 35 [file DataSheet7.ZIP › ERK-2/ERK tublin 3.tif]

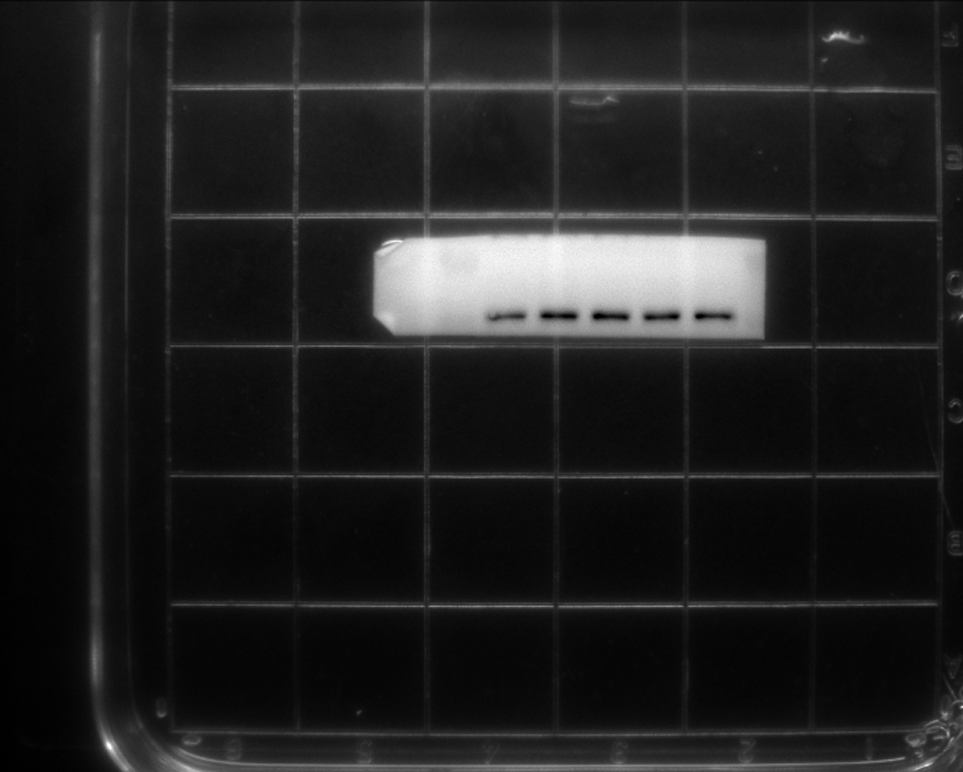

Supplement: Supplementary file 35 [file DataSheet7.ZIP › ERK-2/ERK tublin q.tif]

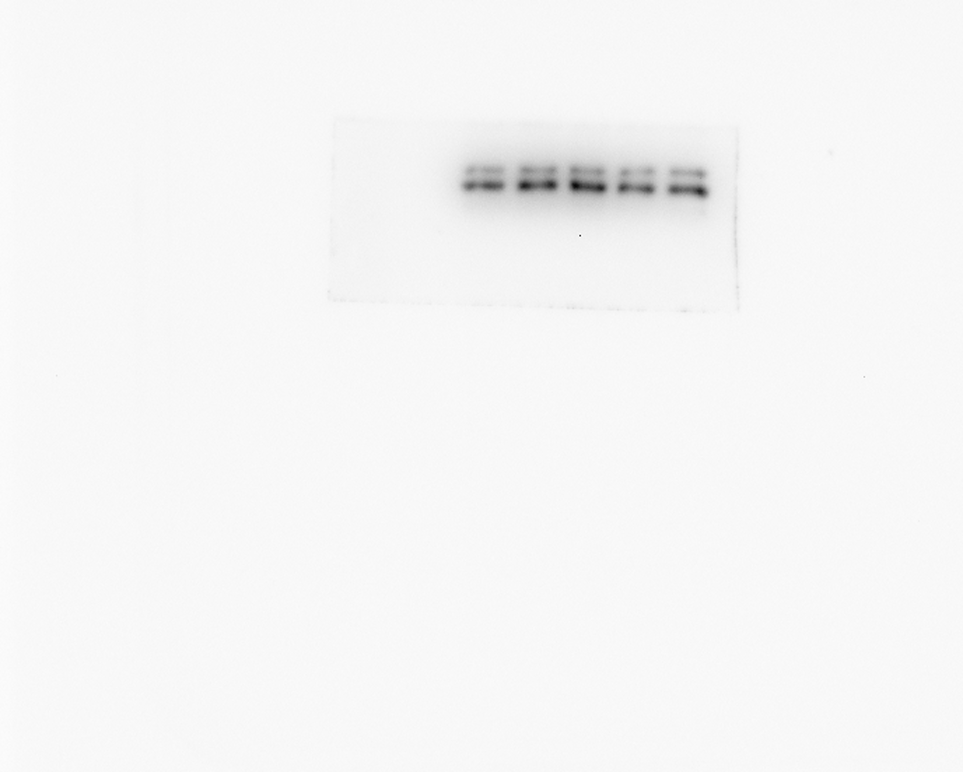

Supplement: Supplementary file 35 [file DataSheet7.ZIP › ERK-2/p-ERK 1.tif]

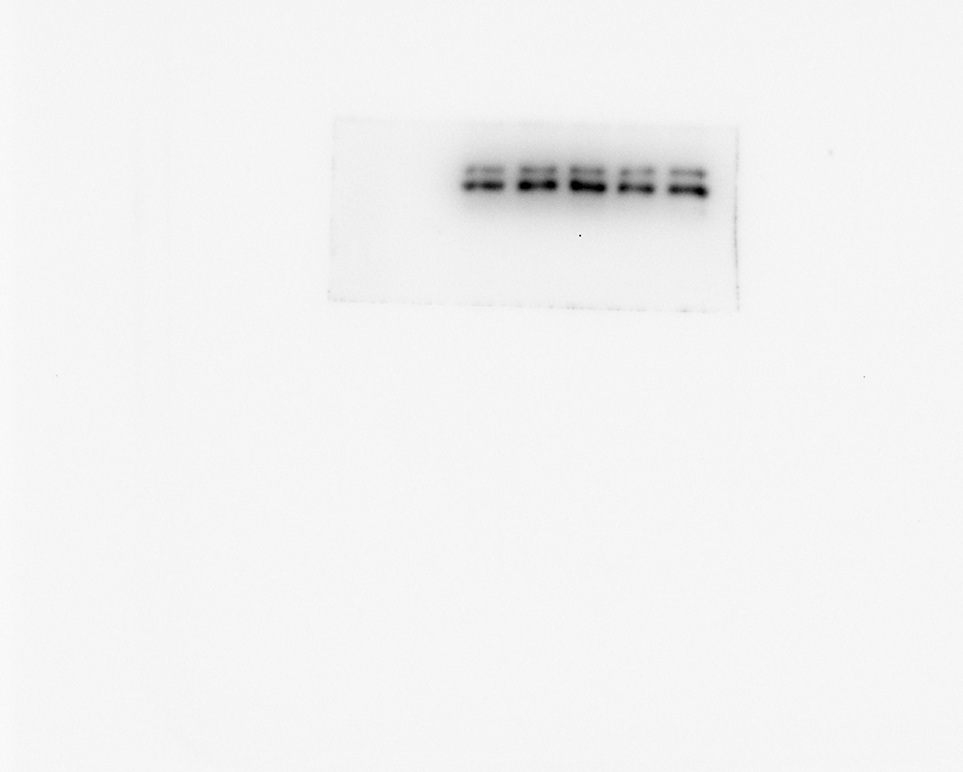

Supplement: Supplementary file 35 [file DataSheet7.ZIP › ERK-2/p-ERK 2.tif]

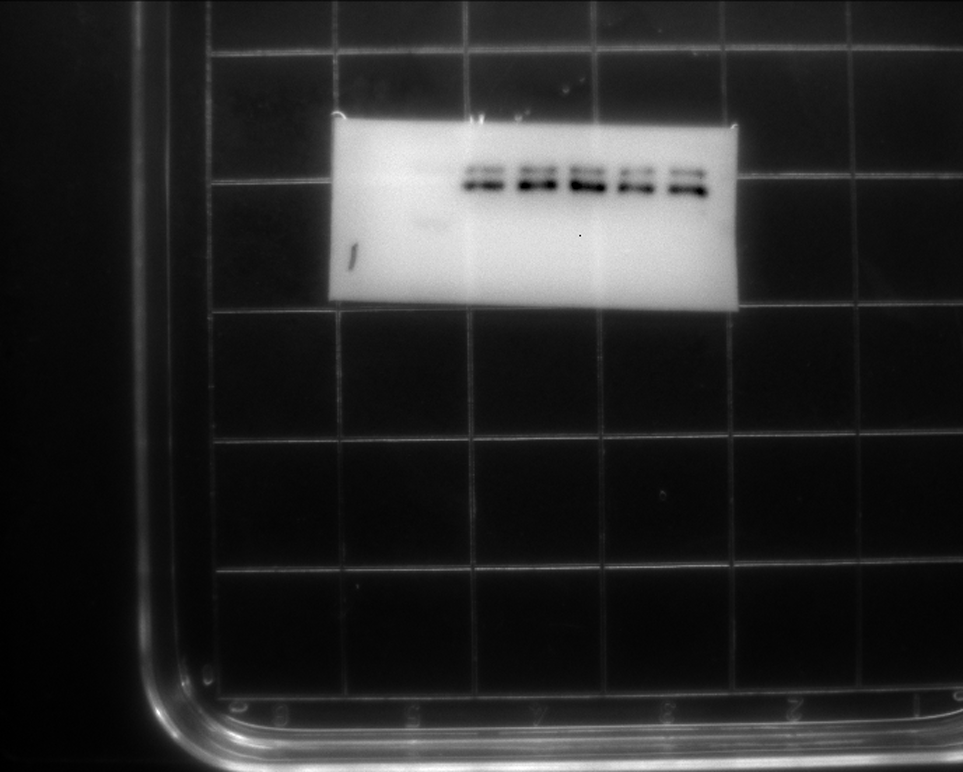

Supplement: Supplementary file 35 [file DataSheet7.ZIP › ERK-2/p-ERK q.tif]

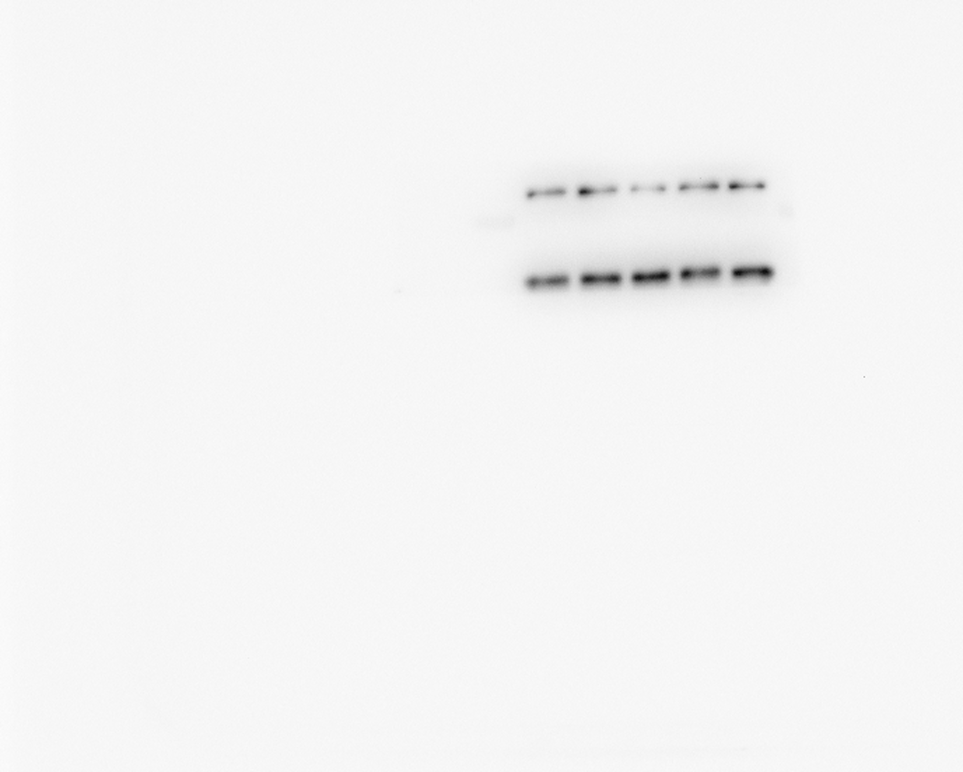

Supplement: Supplementary file 35 [file DataSheet7.ZIP › ERK-2/p-ERK tublin 1.tif]

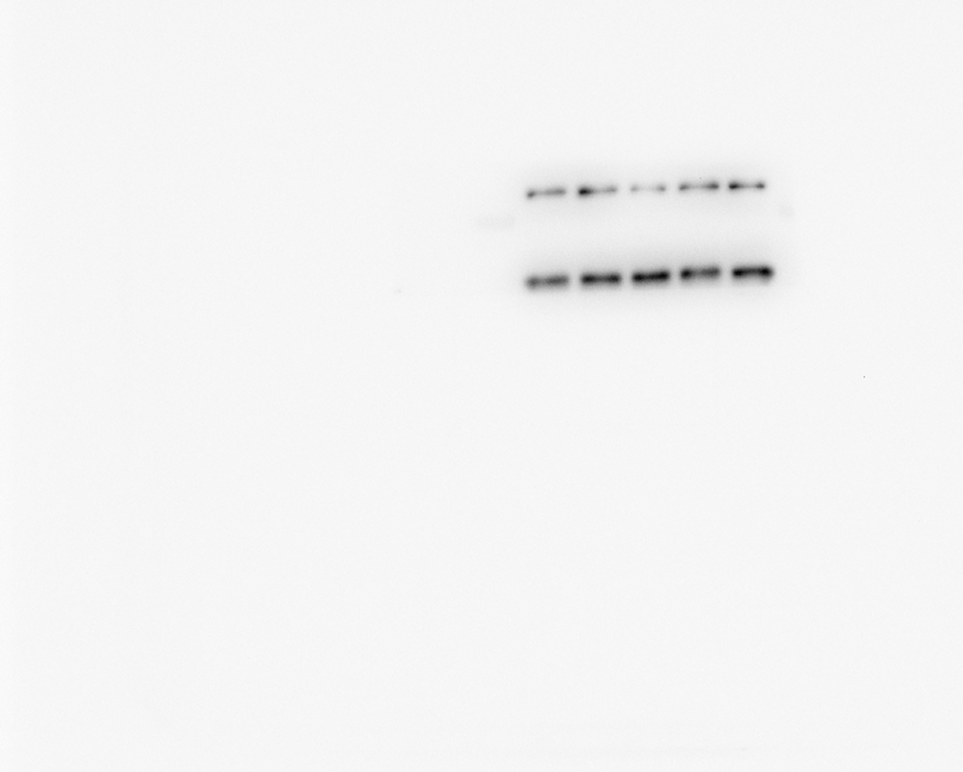

Supplement: Supplementary file 35 [file DataSheet7.ZIP › ERK-2/p-ERK tublin 2.tif]

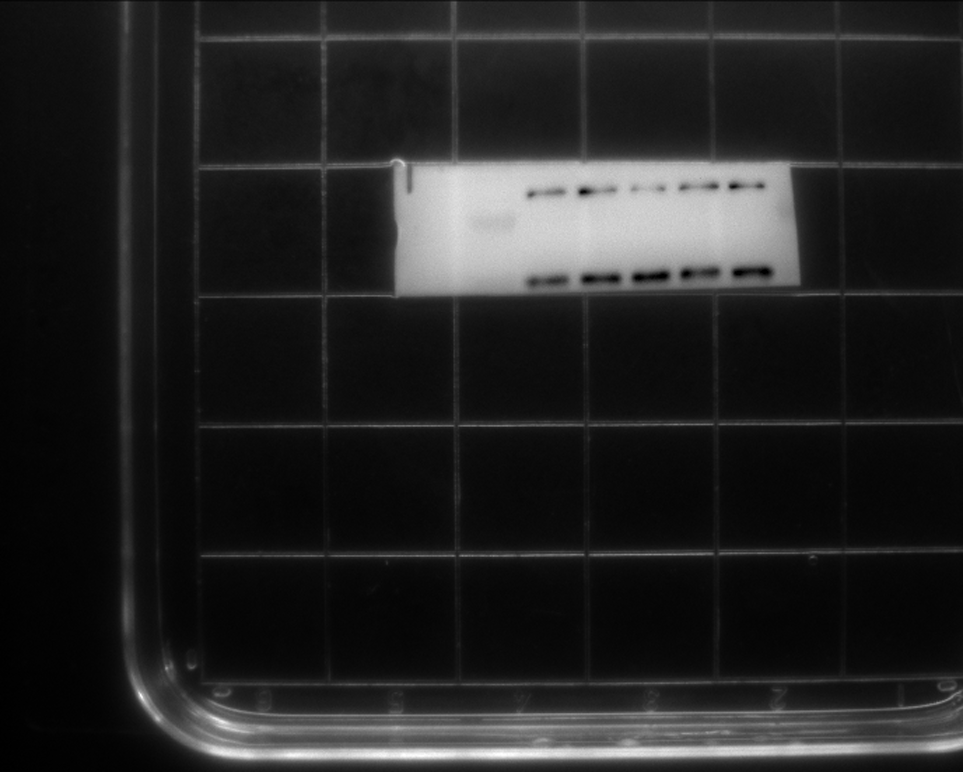

Supplement: Supplementary file 35 [file DataSheet7.ZIP › ERK-2/p-ERK tublin q.tif]

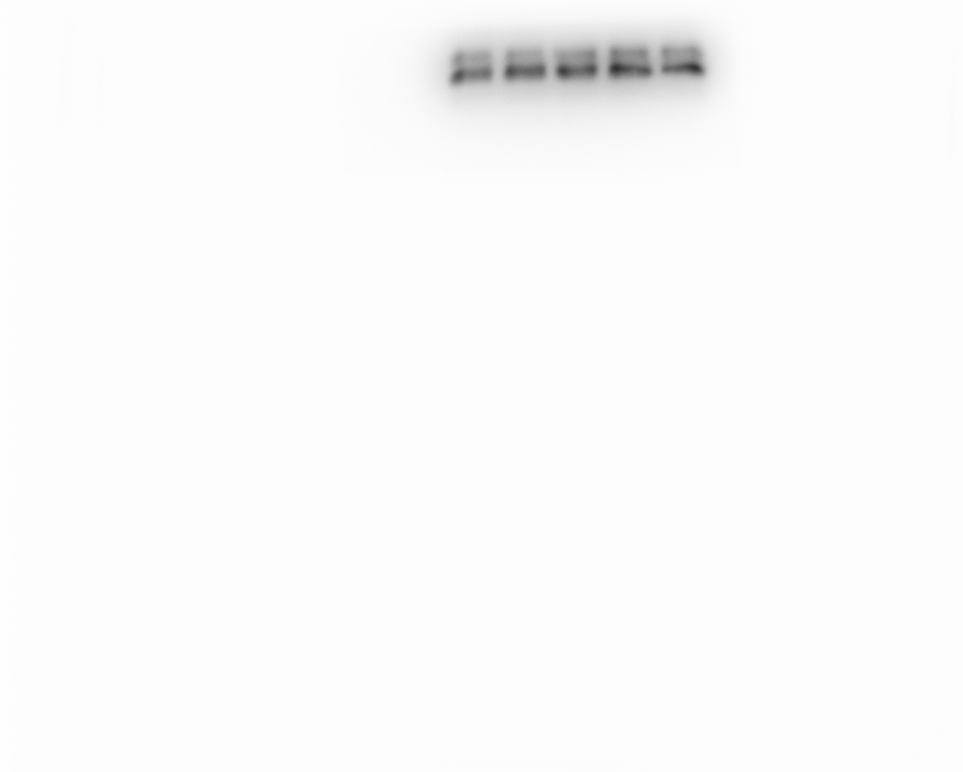

Supplement: Supplementary file 35 [file DataSheet7.ZIP › ERK-3/ERK 1.tif]

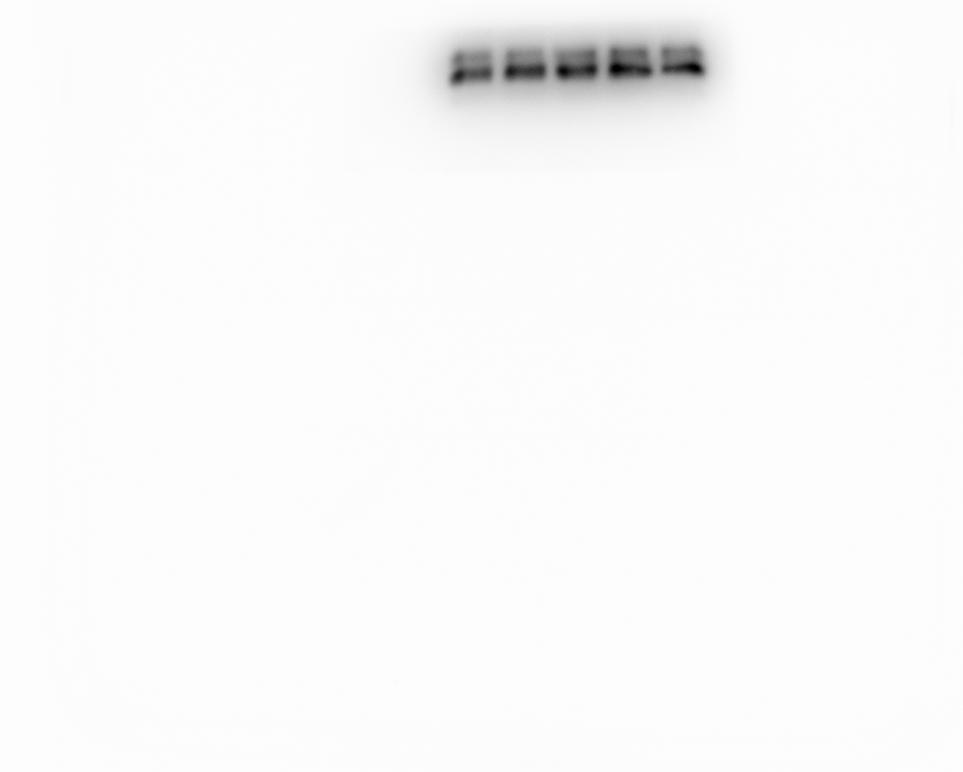

Supplement: Supplementary file 35 [file DataSheet7.ZIP › ERK-3/ERK 2.tif]

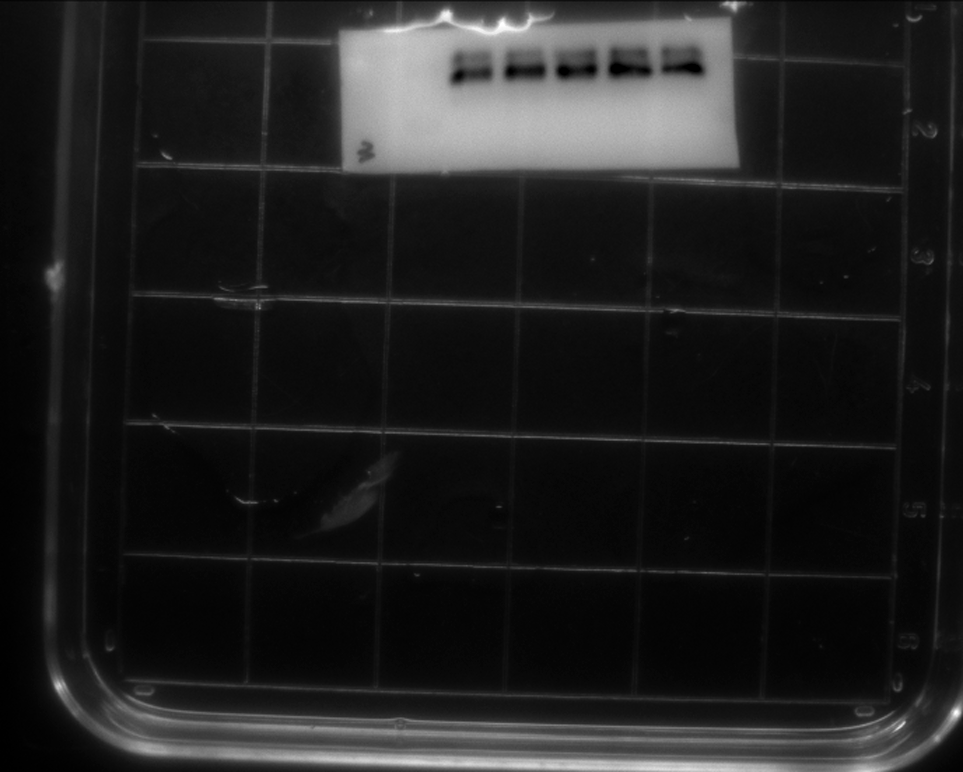

Supplement: Supplementary file 35 [file DataSheet7.ZIP › ERK-3/ERK Q.tif]

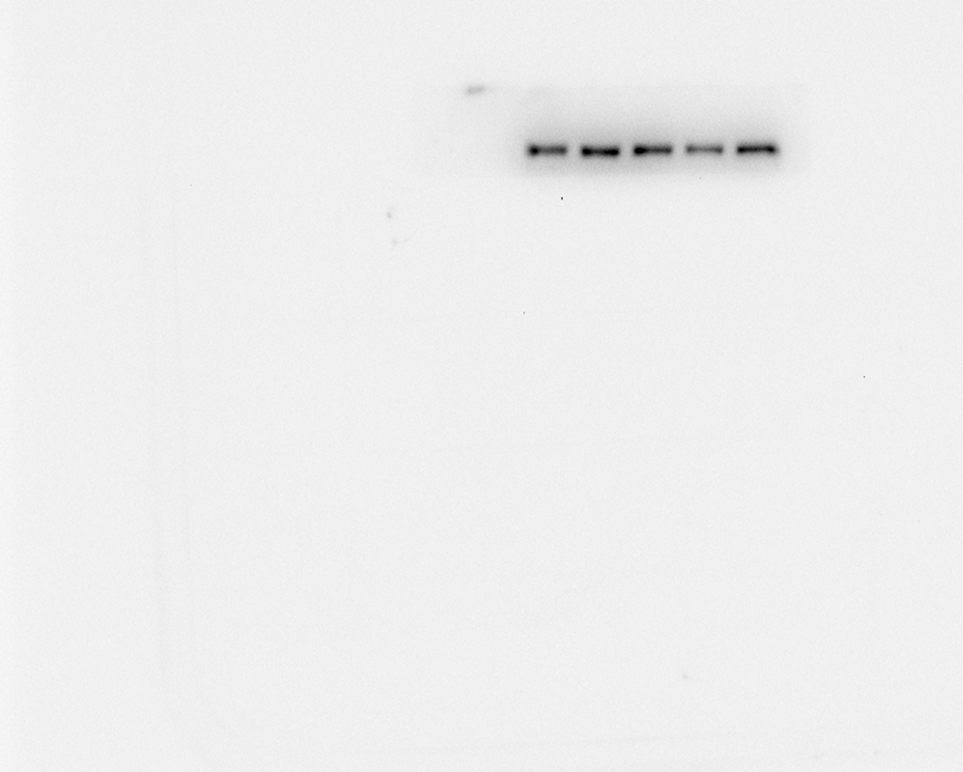

Supplement: Supplementary file 35 [file DataSheet7.ZIP › ERK-3/ERK tublin 1.tif]

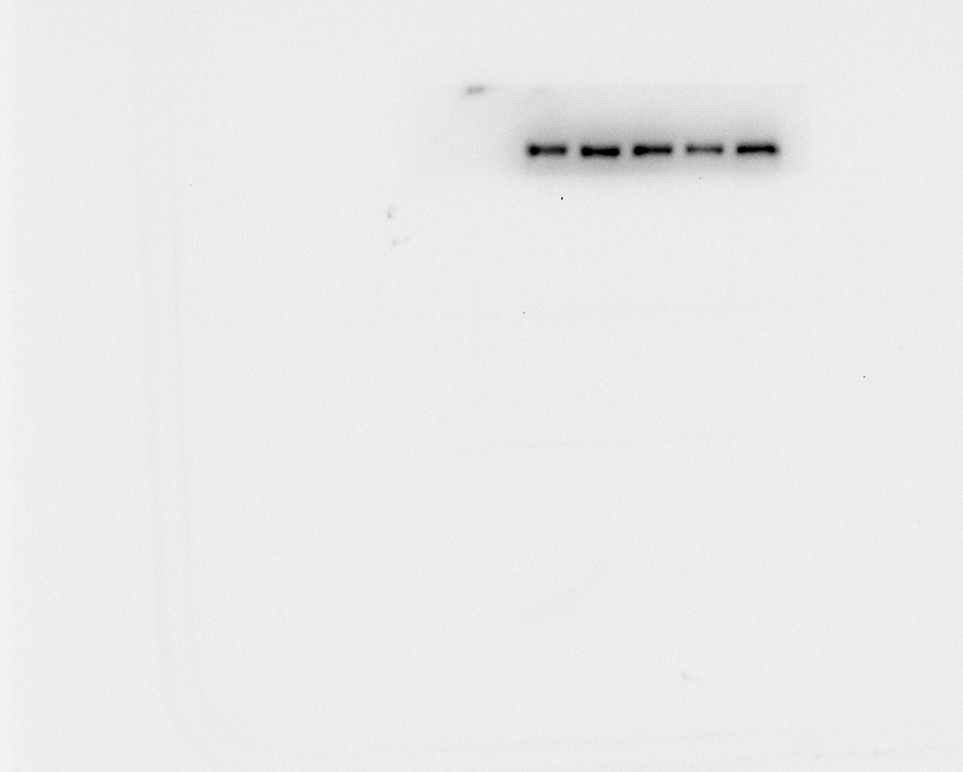

Supplement: Supplementary file 35 [file DataSheet7.ZIP › ERK-3/ERK tublin 2.tif]

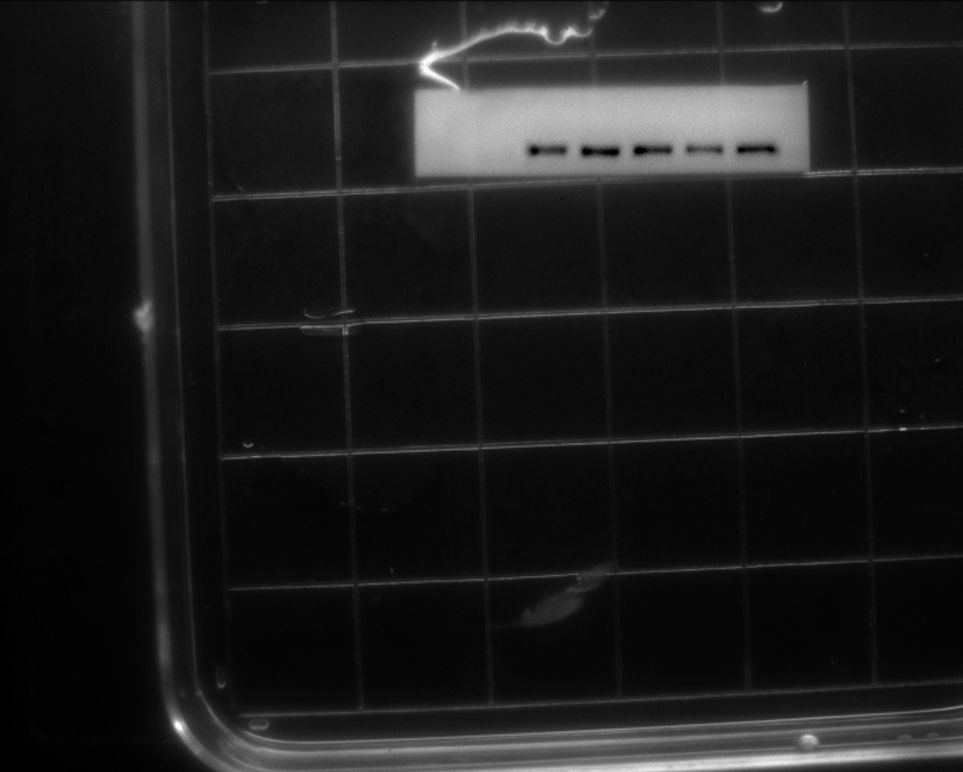

Supplement: Supplementary file 35 [file DataSheet7.ZIP › ERK-3/ERK tublin q.tif]

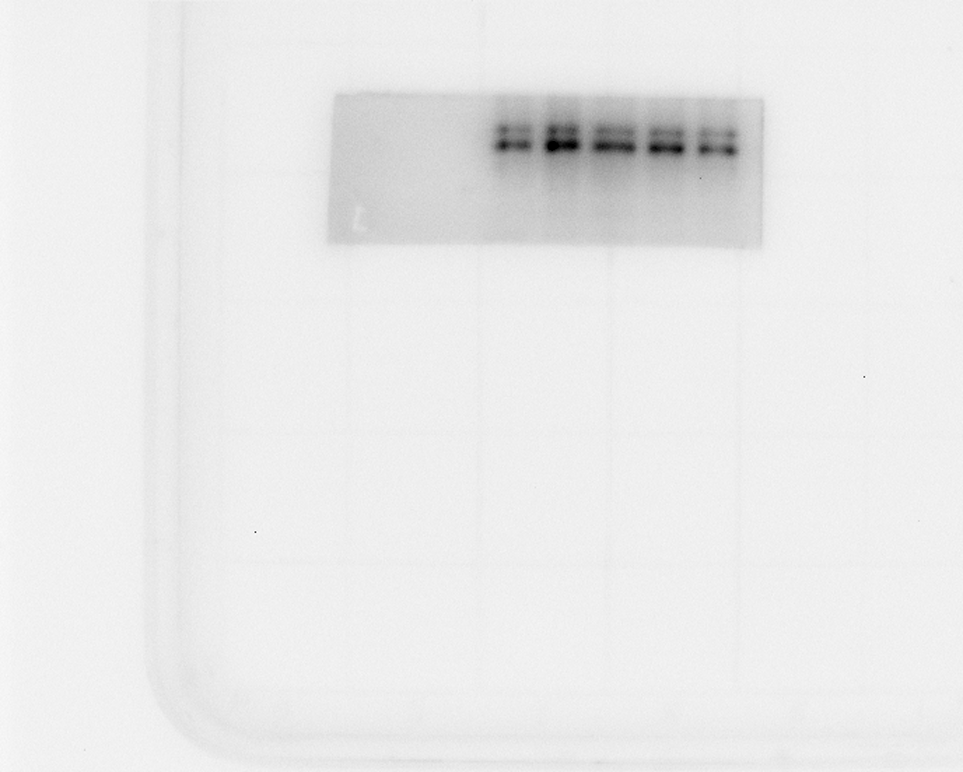

Supplement: Supplementary file 35 [file DataSheet7.ZIP › ERK-3/p-erk 1.tif]

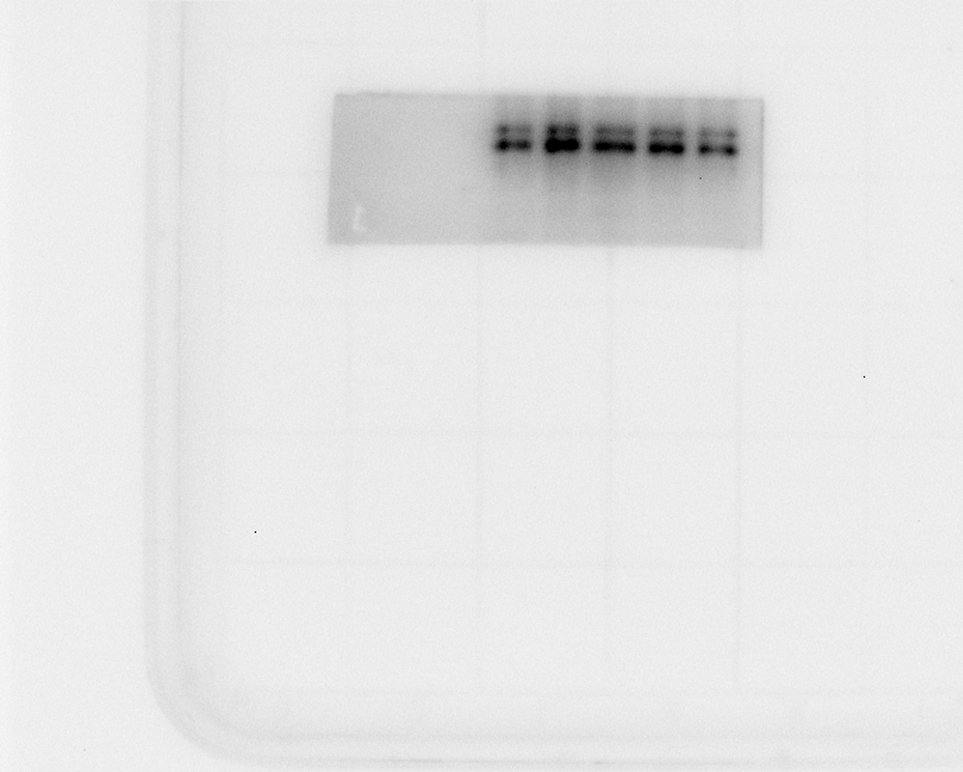

Supplement: Supplementary file 35 [file DataSheet7.ZIP › ERK-3/p-erk 2.tif]

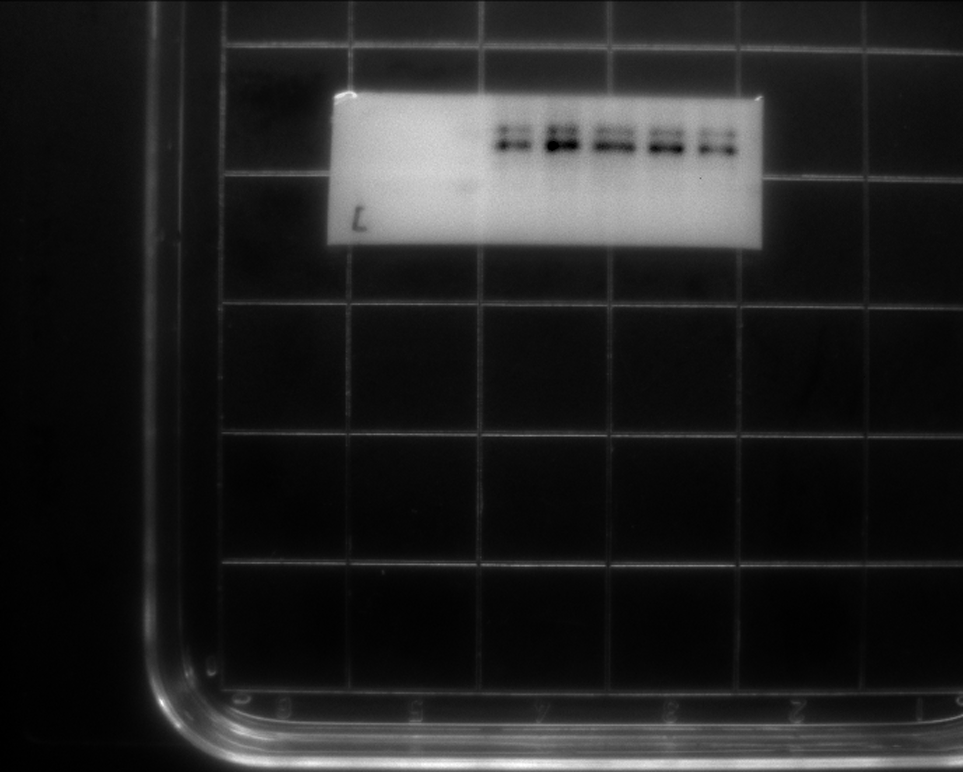

Supplement: Supplementary file 35 [file DataSheet7.ZIP › ERK-3/p-erk q.tif]

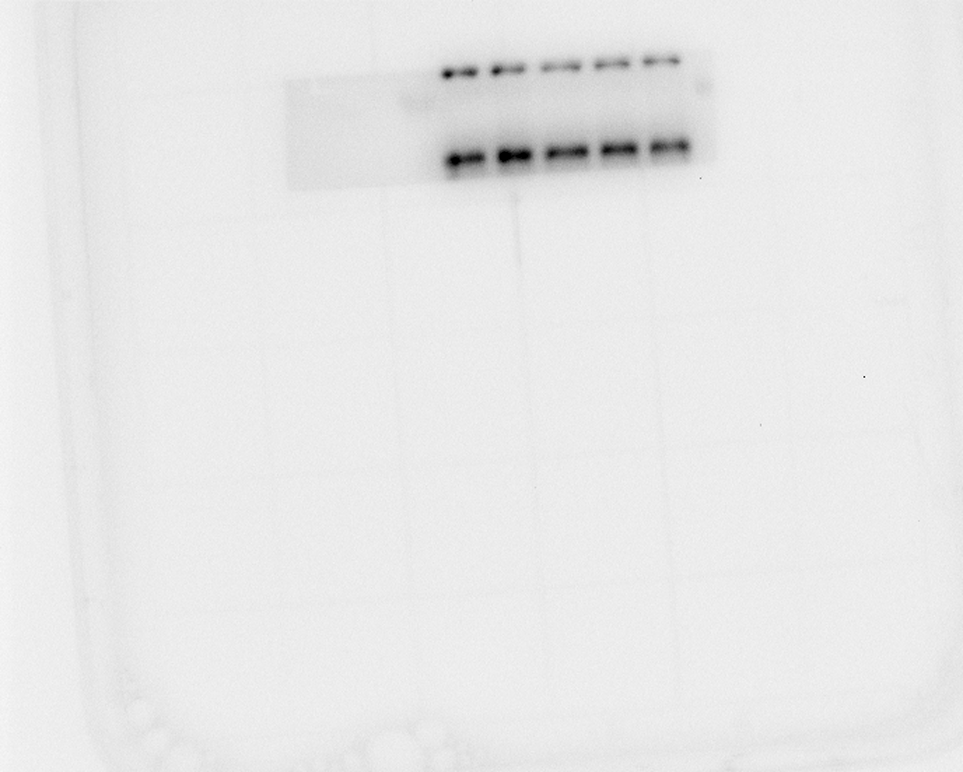

Supplement: Supplementary file 35 [file DataSheet7.ZIP › ERK-3/p-erk tublin 1.tif]

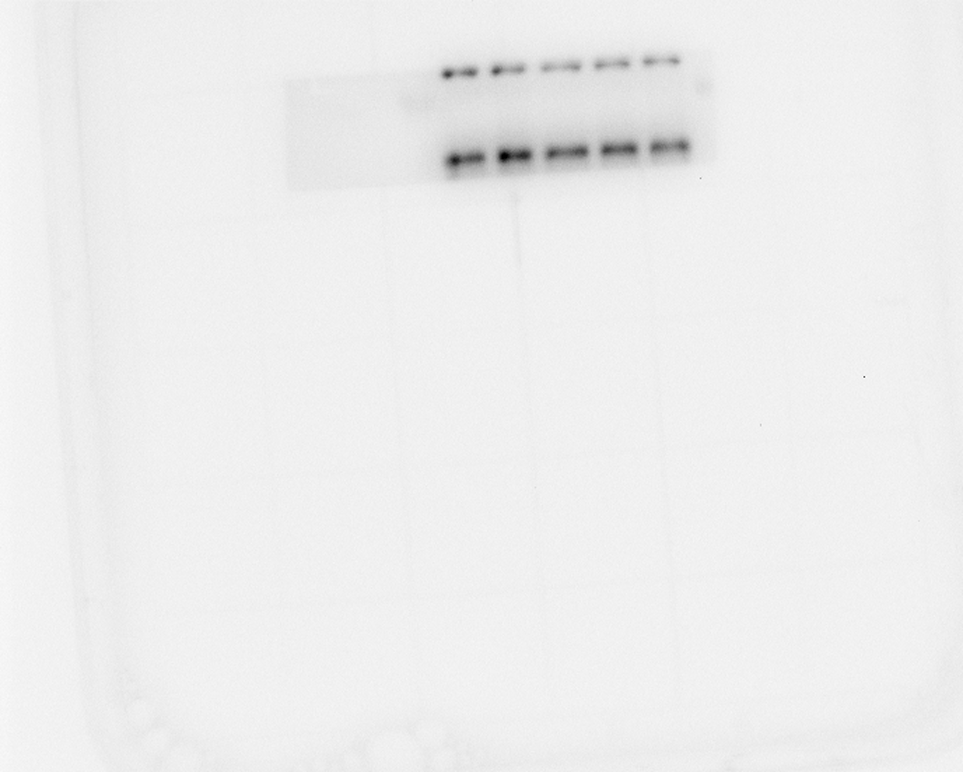

Supplement: Supplementary file 35 [file DataSheet7.ZIP › ERK-3/p-erk tublin 2.tif]

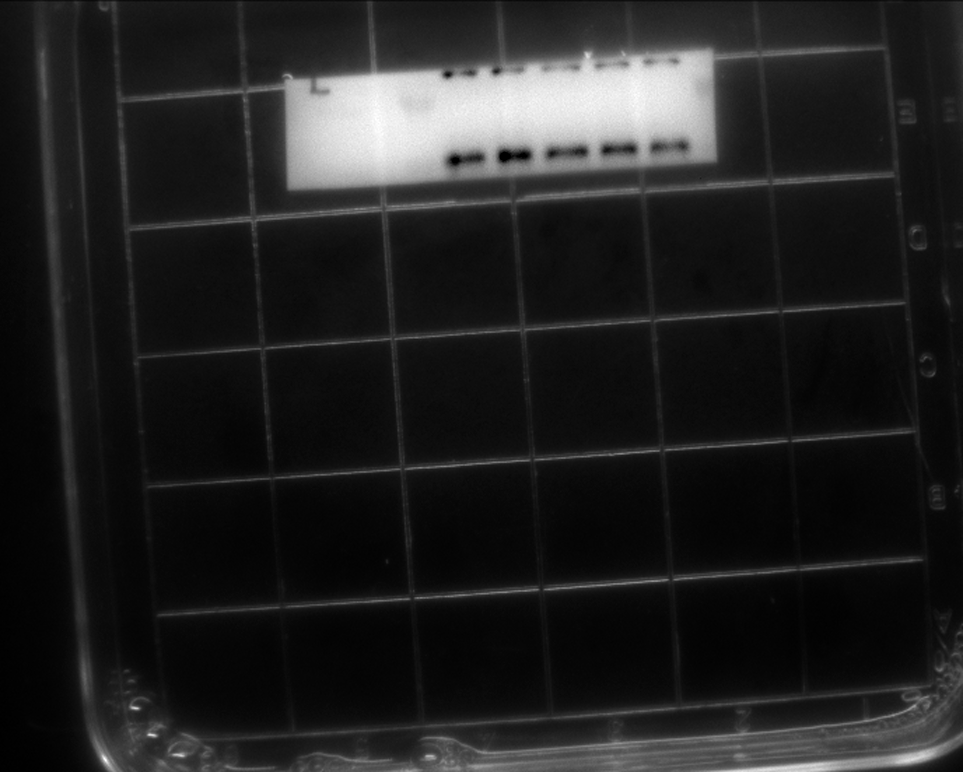

Supplement: Supplementary file 35 [file DataSheet7.ZIP › ERK-3/p-erk tublin q.tif]

## Slide 1
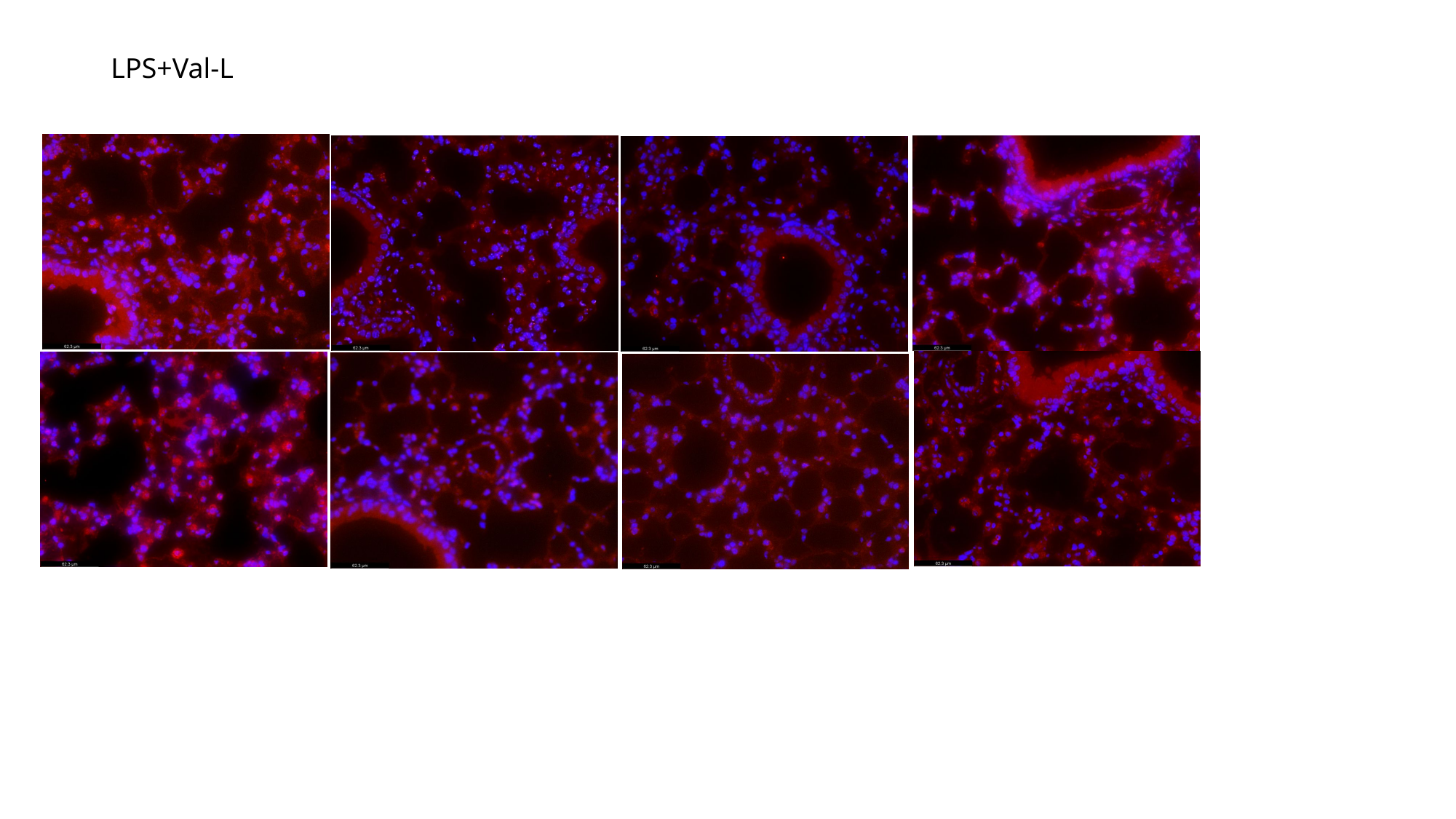

LPS+Val-L

Supplement: Supplementary file 36 [file Presentation5.PPTX]
